# Strain‐Modulated Reactivity: An Acidic Silane

**DOI:** 10.1002/anie.202015960

**Published:** 2021-03-12

**Authors:** Serhii Tretiakov, Léon Witteman, Martin Lutz, Marc‐Etienne Moret

**Affiliations:** ^1^ Utrecht University Organic Chemistry & Catalysis Institution Debye Institute for Nanomaterials Science Faculty of Science 3584 CG Utrecht The Netherlands; ^2^ Utrecht University Structural Biochemistry Bijvoet Centre for Biomolecular Research Faculty of Science 3584 CH Utrecht The Netherlands

**Keywords:** acidity, silanes, silicon, strained molecules, zwitterions

## Abstract

Compounds of main‐group elements such as silicon are attractive candidates for green and inexpensive catalysts. For them to compete with state‐of‐the‐art transition‐metal complexes, new reactivity modes must be unlocked and controlled, which can be achieved through strain. Using a tris(2‐skatyl)methylphosphonium ([TSMPH_3_]^+^) scaffold, we prepared the strained cationic silane [TSMPSiH]^+^. In stark contrast with the generally hydridic Si−H bond character, it is acidic with an experimental p*K*_a_^DMSO^ within 4.7–8.1, lower than in phenol, benzoic acid, and the few hydrosilanes with reported p*K*_a_ values. We show that ring strain significantly contributes to this unusual acidity along with inductive and electrostatic effects. The conjugate base, TSMPSi, activates a THF molecule in the presence of CH‐acids to generate a highly fluxional alkoxysilane via trace amounts of [TSMPSiH]^+^ functioning as a strain‐release Lewis acid. This reaction involves a formal oxidation‐state change from Si^II^ to Si^IV^, presenting intriguing similarities with transition‐metal‐mediated processes.

## Graphical abstract

The reactivity of a ≡SiH group can be greatly altered by enclosing it into a strained bicyclic scaffold. The resulting silane is acidic with an experimental p*K*_a_^DMSO^ of 4.7–8.1, lower than phenol, benzoic acid, and most hydrosilanes. It also shows strain‐release Lewis acidity in the form of coordination of a THF molecule with its subsequent activation towards weak nucleophiles. The resulting ring‐opening product shows high geometric fluxionality.

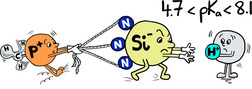

## Introduction

The main‐group elements, sometimes also named s‐ and p‐block elements, are a diverse part of the periodic table. They are the most prevalent components of the Earth's crust and have enormous economic, industrial and environmental significance. Despite all that, their catalytic applications are still scarce compared to those of transition metals,^[1]^ although strategies aiming to bridge this gap have recently emerged. In particular, incorporating a main‐group element into a strained ring system can unlock unusual reactivity. For example, forcing non‐trigonal geometries at a phosphorus(III) centre can give it an electrophilic character in addition to the natural nucleophilicity arising from the lone pair.^[2]^ Such *biphilic* compounds have been shown to engage in unusual bond activation pathways such as (reversible) oxidative addition of E−H bonds (E=OR, NR_2_, Ru).^[3]^

**Figure 1:**
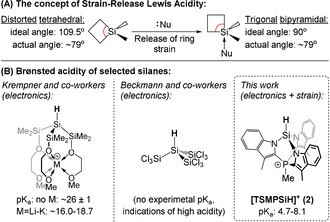
A) An example of strain‐release Lewis acidity for siletanes.^[1l]^ B) Selected acidic silanes[^12^, ^13^] and the silane discussed in this work (acidic protons are shown in bold). Experimental p*K*_a_ values are given as projected onto the DMSO scale.

Both Lewis^[4]^ and Brønsted[^5^, ^6^] acid‐base properties can also be manipulated using strain. In particular, Denmark's “strain‐release Lewis acidity”^[4]^ is based on the fact that the angle strain is partially relieved upon binding of a nucleophile (Figure 1 A). It has been most extensively studied for silicon[^7^, ^8^, ^9^] resulting in a number of highly‐enantioselective synthetic protocols (most often C−C bond forming) that employ strained silanes as directing groups,^[10]^ but also extends to other elements such as germanium^[4]^ and aluminium.^[11]^

Because of the generally hydridic character of Si−H bonds, Brønsted SiH‐acids are rare in general. Recent examples by Krempner and co‐workers^[12]^ and Beckmann and co‐workers,^[13]^ shown in Figure 1 B, mostly rely on electronic effects. In this paper, we show that ring strain can significantly contribute to the acidic character of a Si−H bond.

Herein, we report the silanide‐silane acid‐base pair TSMPSi (**1**)/[TSMPSiH]^+^ (**2**), with an unusually low solution p*K*_a_^DMSO^ (Figure 1 B). It is experimentally shown to lie between 4.7 and 8.1, which is more acidic than phenol, benzoic acid (p*K*_a_^DMSO^ of 18.0^[14]^ and 11.1,^[15]^ respectively), and other silanes of which the p*K*_a_^DMSO^ was reported. We analyse the physicochemical origins of this unusual acidity in terms of inductive and electrostatic effects and confirm its link to ring strain. In addition, the reactivity of both TSMPSi (**1**) and [TSMPSiH]^+^ (**2**) is investigated. The increase in strain that generally accompanies quaternization of the silicon atom in **1**, together with charge separation effects, render it a weaker nucleophile than typical silicon anions. Moreover, we provide evidence that, under the influence of strain, quaternized **1** can transfer a methyl group thus engaging into “strain‐release methyl transfer”. Finally, **2** shows strain‐release Lewis acidity that manifests itself in the coordination and activation of a THF molecule towards attack by weak nucleophiles, such as highly‐delocalized aromatic anions. The product of THF ring‐opening exhibits a high degree of fluxionality, which is analysed using a combination of spectroscopic and computational tools.

## Results and Discussion

### Synthesis and Characterization of the Acid–Base Pair

**Scheme 1:**
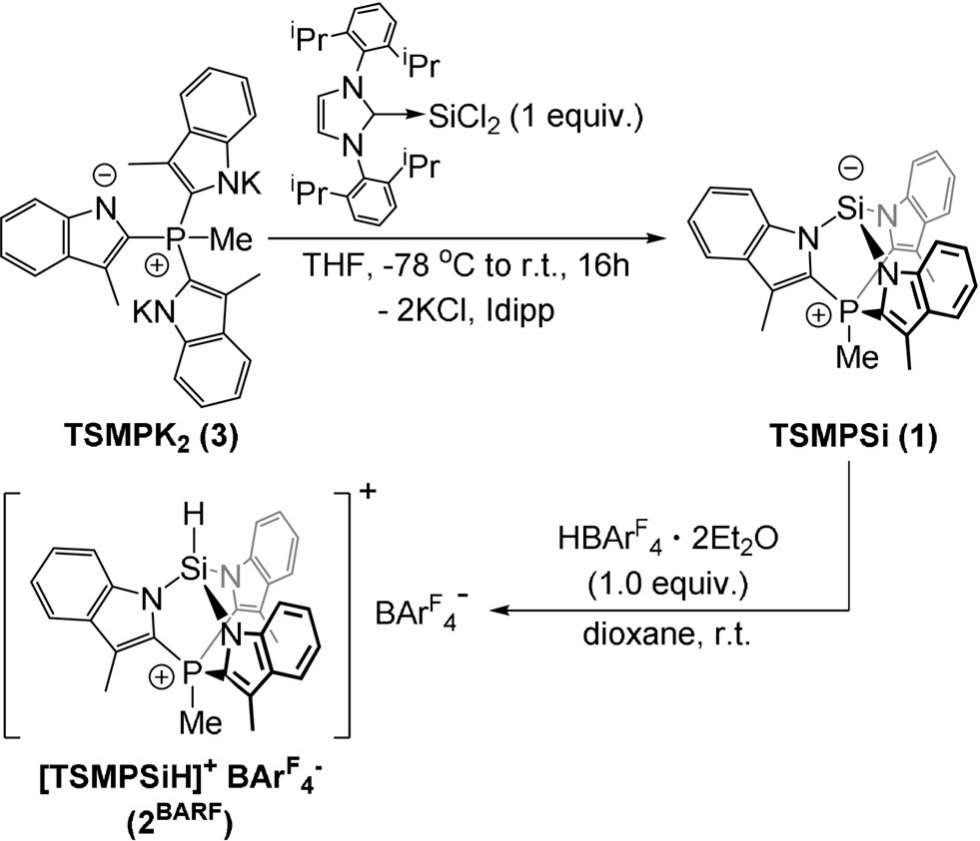
Synthesis of zwitterion **1** and its protonated salt **2^BARF^**.

Zwitterionic Si^II^ silanide TSMPSi (**1**) was isolated from the reaction of the tris‐indolide salt TSMPK_2_ (**3**)^[16]^ with Idipp→SiCl_2_ used as a Si^II^ source (Scheme 1).^[17]^ The corresponding cationic Si^IV^ silane, [TSMPSiH]^+^BAr^F^_4_^−^ (**2^BARF^**), was synthesized by protonation with an equimolar amount of Brookhart's acid (HBAr^F^_4_⋅2 Et_2_O). Attempted protonation with HCl led to an intractable mixture of insoluble products, highlighting the importance of a non‐coordinating anion in this reaction (Section S5.1.1).

The ^1^H NMR spectra of TSMPSi (**1**) in [D_8_]THF and [TSMPSiH]^+^BArF_4_^−^ (**2^BARF^**) in [D_2_]DCM each show a single set of four aromatic signals indicating C_3_ symmetry that corresponds to heterobicyclo[2.2.2]octane topology. The ^29^Si NMR spectrum of **1** shows a doublet at −48.0 ppm with ^3^ *J*_Si,P_=4.3 Hz, which is consistent with the DFT‐calculated values of −56.2 ppm and 7.9 Hz, respectively (Section S4.2). Upon protonation of **1** into **2^BARF^**, the ^29^Si NMR signal becomes a doublet of doublets at −46.4 ppm with ^1^ *J*_Si,H_=318.4 Hz and ^3^ *J*_Si,P_=8.0 Hz, consistent with a tetrahedral tertiary silane. The respective DFT‐calculated NMR parameters, −59.0 ppm, −342.3 and −7.5 Hz (Section S4.2), are in a good agreement with the experiment. Even though the Si−H signal in ^1^H NMR of **2^BARF^** is obscured by aromatic multiplets, its position at 7.52 ppm can be clearly determined from ^1^H‐^29^Si ASAP‐HMQC spectra.

**Figure 2:**
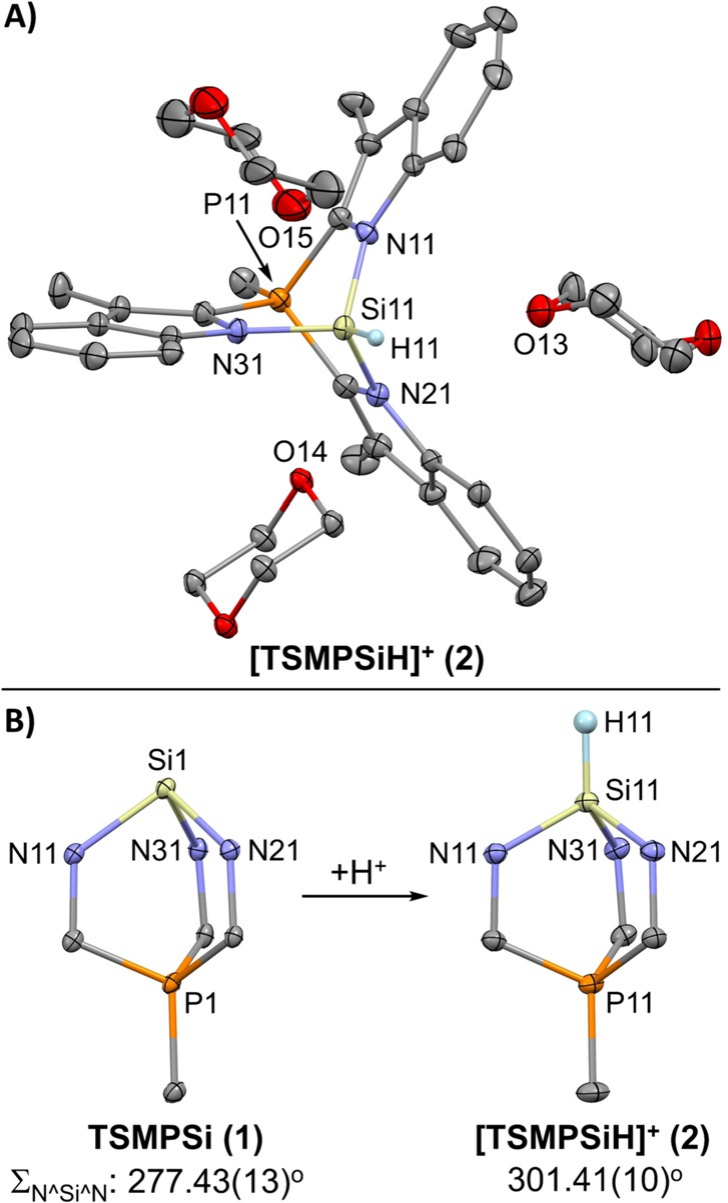
A) Molecular structure of a cationic unit of **2^BARF^** from X‐ray crystal structure determination. Displacement ellipsoids are drawn at 30 % probability level. The BAr^F^_4_^−^ counterion and hydrogen atoms, except for Si−H, are omitted for clarity. B) Structural changes in the bicyclic cage before and after protonation according to X‐ray crystallography. Only one independent molecule of TSMPSi (**1**) is shown. Selected bond distances and angles are given in Section S3.^[51]^

The structures of both **1** and **2^BARF^** were determined by X‐ray crystallography (Figure 2) using crystals grown from benzene/*n*‐hexane and DCM/1,4‐dioxane, respectively. The asymmetric unit of **1** contains two independent molecules, each featuring a tricoordinate anionic silicon center. A sum of the N^Si^N angles close to 270°, namely 277.43(13)/277.51(14)°, indicates a high s‐character of the anionic lone pair.

The structure of **2^BARF^** features a tetrahedral Si center; the Si‐bound H atom could be located from the difference Fourier maps. Interestingly, every cationic unit of **2^BARF^** is surrounded by three 1,4‐dioxane molecules^[18]^ with Si−O distances within 2.7807(13)–3.2260(16) Å, which is shorter than the sum of van der Waals radii (3.62 Å),^[19]^ suggesting weak Si⋅⋅⋅O interactions. The latter are best described as interactions between the O‐centered lone pairs and the σ‐hole opposite the polar Si−N bonds.^[20]^ The silane hydrogen atom shows rather short contacts to the dioxane oxygens, but the Si^H^O angles of 82.7(10)–92.0(10)° are unfavourable for hydrogen bonding. The above suggests that the silicon centre has a Lewis acidic character, while the Si−H bond remains polarized towards hydrogen and is hence not prone to engage in hydrogen bonding.

It is worth pointing out that protonation causes marked geometrical changes around Si (Figure 2 B), wherein the Si−N distances shorten from 1.840(2)–1.864(2) Å in **1** to 1.752(1)–1.762(2) Å in **2^BARF^**. Concomitantly, the sum of N^Si^N angles increases from 277.43(13)/277.51(14)° in **1** to 301.41(10)° in **2^BARF^**, while the intracyclic Si^N^C angles decrease from 125.05(13)–126.36(14)° in **1** to 119.11(10)–119.32(9)° in **2^BARF^**. No other bond in the molecule changes by >0.02 Å, and no flat angle deforms by >2.9°. In other words, structural perturbations due to proton addition are mostly confined to the SiN_3_ fragment. The observed changes are consistent with an increased s‐character of the Si−N bonding orbitals as the Si‐centred electron pair in **1** acquires higher p‐character upon protonation, which is in line with Bent's rule.^[21]^

### Experimental Solution p*K*_a_ Determination

Examining the solid‐state structure of TSMPSi (**1**) as well as κ^3^ complexes of Fe^II^, Ni^II^, and Cu^I^ with the TMSP scaffold,^[16]^ it becomes apparent that the latter favors N^E^N angles that are close to 90°. Hence, the N^Si^N angles of about 100° in [TSMPSiH]^+^ (**2**) are expected to generate strain within the cage structure. In addition, the ^1^ *J*_Si,H_ coupling constant in **2^BARF^** (318.4 Hz) is significantly larger than that in the electronically similar yet unstrained tris‐*N*‐pyrrolylsilane (284.5 Hz).^[22]^ Similar increased ^1^ *J*_C,H_ coupling constants in strained hydrocarbons^[6]^ have been correlated to a high acidity of the corresponding C−H bonds. These considerations, combined with the overall positive charge of [TSMPSiH]^+^ (**2**), led us to anticipate its high SiH‐acidity.

**Figure 3:**
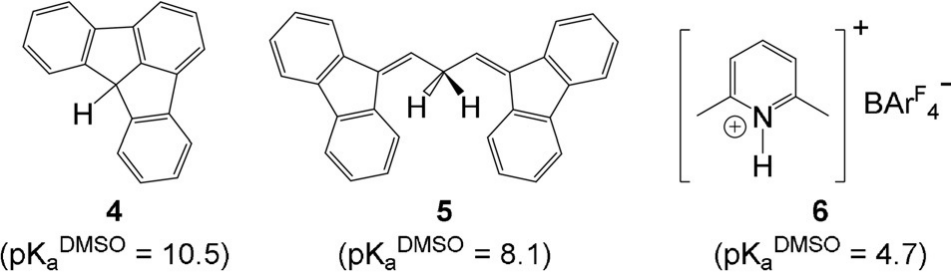
Acids used in experimental studies.[^25^, ^26^]

To test this, we undertook experimental p*K*_a_ measurements. Of the several existing ways to measure p*K*_a_ in non‐aqueous media,^[23]^ we chose a bracketing approach: the basic form **1** was dissolved together with acids of known p*K*_a_, and proton transfer was monitored using NMR. CH‐acidic fluorenes **4** and **5** as well as 2,6‐lutidinium‐BAr^F^_4_ salt **6** were used as reference acids (Figure 3). Fluorenes have previously been used to measure the acidity of silanes,[^12^, ^24^] and their conjugate bases are strongly delocalized anions which are not expected to coordinate to the protonated silicon centre. 2,6‐Lutidine is also unlikely to coordinate due to steric bulk. Because of the intrinsically low kinetic acidity of most CH‐acids, we chose [D_8_]dioxane as a solvent, reasoning that a hydrogen bond acceptor could promote fast and efficient proton transfer. It was given preference over the much more common [D_8_]THF due to its lesser sensitivity to strong acids and lower susceptibility to (catalytic) ring‐opening. To check that an acid‐base equilibrium is reached, the measurements were repeated starting from **2^BARF^** and the conjugate bases of the reference acids.

While the acidity of the reference acids that we used is known only in DMSO, cationic silane **2** cannot exist in this solvent and immediately degrades. However, given a linear correlation between acidities in different solvents (i.e. the relative acidity does not change from one solvent to another),^[27]^ one can still perform an acid‐base reaction in [D_8_]dioxane and project the result onto the DMSO acidity scale.^[28]^ Even though the projected p*K*_a_ does not reflect solvent‐specific effects (viz. degradation), it allows for comparison with p*K*_a_ of other acids most of which are only known in DMSO.[^27^, ^30^]

Silanide **1** is not protonated by fluoradene (**4**) which has p*K*_a_^DMSO^ of 10.5. With the stronger CH‐acid **5** (p*K*_a_^DMSO^=8.1), the protonation is also not observed directly. However, **5** undergoes base‐catalysed isomerization in the presence of TSMPSi (**1**) (Section S5.1.2), suggesting comparable acidities. Additionally, the solution becomes pale blue, indicating the presence of a small concentration of the delocalized anion obtained by deprotonating **5**. Finally, protonation of **1** with 2,6‐lutidinium‐BAr^F^_4_ (**6**) in [D_8_]dioxane is complete with release of free 2,6‐lutidine. Thus, one can conclude that the projected p*K*_a_ of silane **2** in DMSO lies between 4.7 and 8.1.

The high acidity of cationic silane **2** is remarkable in view of the general hydridic character of silicon‐bound hydrogen atoms. In fact, it is more acidic than phenol and benzoic acid (p*K*_a_^DMSO^ of 18.0^[14]^ and 11.1,^[15]^ respectively). It is also considerably more acidic than the few hydrosilanes whose p*K*_a_ has been measured: triphenylsilane (p*K*_a_^THF^≈35.1),^[31]^ tris(trimethylsilyl)silane (p*K*_a_^ether^≈29.4),^[24]^ as well as the cationic alkali metal‐silane complexes shown in Figure 1 B.^[12]^ In the latter series, the introduction of a cationic centre into the molecule decreases the p*K*_a_ in benzene by six to eleven orders of magnitude, suggesting that [TSMPSiH]^+^ (**2**) owes at least a part of its acidity to the presence of a positive charge in the molecule. It is additionally worth mentioning that trichlorosilane,^[32]^ tris(pentafluoroethyl)silane,^[33]^ and tris(trichlorosilyl)silane^[13]^ display acidic reactivity in solution, but no experimental p*K*_a_ has been reported.

### Computational Analysis of Acidity of [TSMPSiH]^+^ (2)

**Figure 4:**
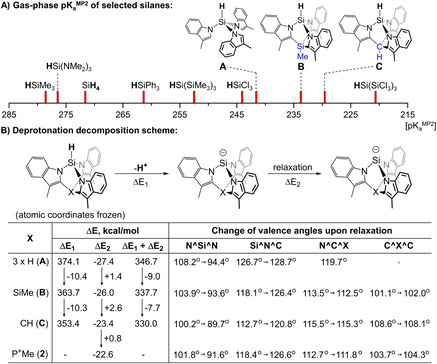
A) Comparison of MP2‐calculated gas‐phase acidities for neutral silanes, including tris(trichlorosilyl)silane, HSi(SiCl_3_)_3_, from Beckmann and co‐workers.^[13]^ The p*K*_a_^MP2^ of cationic silane **2** (177.7) is not shown on the scale along with neutral silanes since it cannot be directly compared (see text). B) Deprotonation energy decomposition Scheme at B3LYP‐GD3BJ/6–311++G** level of theory. Δ*E* reflect changes in SCF energy.

*I. Factors contributing to the acidity*. The high acidity of cationic silane [TSMPSiH]^+^ (**2**) can be ascribed to three cumulative effects: the strong electron‐withdrawing effect of indolyl nitrogens, ring strain, and the overall positive charge. The respective contributions of these effects can, in principle, be roughly estimated by comparing solution acidity of a series of silanes: SiH_4_, tris‐*N*‐skatylsilane (**A** in Figure 4 A), the neutral isoelectronic Si‐tethered analogue of **2** (**B** in Figure 4 A; see also Section S5.1.3), and **2** itself. Thus, the acidity difference between SiH_4_ and **A** would reflect the electron withdrawal, the difference between **A** and **B** would be reflective of strain, and the difference between **B** and **2** would give information about the influence of charge. Unfortunately, the solution acidities of SiH_4_, **A** and **B** are experimentally unknown. Furthermore, the lack of reference experimental data hampers the development of reliable procedures for calculating these. This problem can be partly circumvented by taking a detour via gas‐phase p*K*_a_’s, which can be calculated with high precision by modern quantum chemical methods.^[34]^ There is an empirical linear dependence between gas‐phase and solution p*K*_a_ values within the same class of acids,[^35^, ^37^, ^38^] which is possible due to the fact that the free energy difference between an acid and a base is attenuated by differential solvation effects that are specific for the acid class, electric charge and solvent. Such correlations have been documented for phenols,^[39]^ alcohols,[^38^, ^40^] amines,^[41]^ thiols,^[41]^ and CH‐acids.[^42^, ^43^] In this way, the gas‐phase p*K*_a_ of 177.7 calculated for **2** can be compared to the gas‐phase p*K*_a_ of other cationic silanes described by Krempner and co‐workers (Figure 1 B),^[12]^ which we calculate as being 205.5 for M=K and 197.8 for M=Li (Section S5.1.4). Knowing that p*K*_a_^DMSO^ of the latter two silanes is 22.3 and 19.8, respectively,^[12]^ and assuming a linear correlation, one arrives to a projected p*K*_a_^DMSO^ of 7.3 for **2**, which falls within the experimental interval of 4.7–8.1. This gives us confidence in the ability of the computed gas‐phase p*K*_a_’s to accurately reflect trends in solution for silanes as well.

Now, comparing calculated gas‐phase p*K*_a_^MP2^ of SiH_4_ (271.6) to that of tris‐*N*‐skatyl silane **A** (241.6) shows that inductive effects reduce the p*K*_a_^MP2^ by roughly 30.0 units. Constraining *N*‐skatyl moieties into the cage structure **B** (p*K*_a_^MP2^=234.5) decreases the sum of N^Si^N angles (Σ_N^Si^N_) from 324.7° in **A** to 311.6° **B**, while reducing the p*K*_a_^MP2^ by an additional 7.1 units, which is about one fourth of the electron withdrawal contribution. This trend is continued by the more constrained C‐tethered analogue **C**, which has not been observed experimentally, but its conjugate base has been isolated.[^17^, ^44^] With a Σ_N^Si^N_ of 300.5°, its gas‐phase p*K*_a_^MP2^ equals 229.6. The increased acidity of **B** and **C** is also in line with a build‐up of strain (9.0 and 16.5 kcal mol^−1^, respectively) with reference to corresponding conjugate bases as analyzed by a series of homodesmotic equations (Section S5.1.5). In other words, deprotonation is accompanied by strain release, which significantly contributes to the acidity of **B**, **C**, and, by extension, compound **2**.

As for the influence of charge, the calculated gas‐phase p*K*_a_^MP2^ of **2** (177.7) is, as expected, much lower than that of **B** (234.5) due to electrostatic effects. Because of a difference in solvation of neutral and charged species,^[34]^ these effects are expected to remain significant but be strongly attenuated in solution relative to electron withdrawal and strain. Therefore, the influence of the positive charge on the solution acidity of **2** is difficult to quantify.

To qualitatively assess the role of the positive charge in the acidity of **2**, we performed Natural Bonding Orbital (NBO) analysis of the deprotonated form **1**. It showed that the anionic lone pair on silicon is hosted in an orbital with predominant s‐character (sp^0.41^ hybridization). The corresponding Natural Localized Molecular Orbital (NLMO) is composed of Si atomic orbitals by 98.1 %, with only minor delocalization into σ*(C−N) orbitals due to hyperconjugation (Section S5.1.6). In other words, the high acidity of **2** does not originate from increased delocalization of the negative charge in its conjugate base **1**, and the interaction between the zwitterionic charges is almost entirely electrostatic.

*II. On the role of strain*. More insight into the influence of strain on acidity of silanes **A**–**C** (Figure 4 A) and **2** can be obtained by decomposing the deprotonation reaction into two formal stages (Figure 4 B).^[45]^ In the first stage, a proton is abstracted from the silane with all other atomic coordinates frozen. The energy of this process (Δ*E*_1_) gauges the penalty of charge separation in the course of heterolytic Si−H bond dissociation followed by electronic relaxation. In the second stage, the deprotonated silane is allowed to relax to the geometry of the corresponding anion, which is described by the respective energy difference (Δ*E*_2_). In essence, Δ*E*_2_ characterizes how strongly the geometry of the silane resembles that of the corresponding silanide. The larger the discrepancy between geometries, the more energy is gained during relaxation, leading to a more negative total energy balance and therefore higher acidity. It is important to note that upon relaxation of deprotonated **B**, **C** and **2**, the N^C^X and C^X^C angles barely change while most of the relaxation occurs within the SiN_3_ fragment, which is analogous to the differences between the X‐ray crystal structures of **2^BARF^** and **1** (Figure 2 B).

Δ*E*_1_ energies become progressively more negative from **A** to **C** (total difference of −20.7 kcal mol^−1^), which correlates with decreasing N^Si^N valence angles in respective silanes. Consequently, acute angles around silicon imposed by the ligand destabilize the silane allowing for easier proton abstraction, and thus higher acidity. Because of the different molecular charge, comparing Δ*E*_1_ of cationic silane **2** with that of neutral silanes **A**–**C** would be meaningless.

During the second stage, relaxation, Δ*E*_2_ energies become progressively less negative from **A** to **C** (total difference of +4.0 kcal mol^−1^), which means that constraint makes the geometry of a silane closer to that of the corresponding anion, hence reducing the acidity. While there is a similarity in valence angles between **2** and its isoelectronic analogue **B**, relaxation for the former is 3.4 kcal mol^−1^ less negative. We connect this to the fact that the distance between positive phosphonium and negative silanide atoms increases upon relaxation. This should reduce electrostatic stabilization, hence slightly increasing Δ*E*_2_ and lowering the acidity.

Even though Δ*E*_1_ and Δ*E*_2_ contribute to the gas‐phase acidity in an opposite way, changes associated with Δ*E*_1_ have larger magnitude, which leads to higher acidity with acuter N^Si^N angles. The progression of Δ*E*_1_ in Figure 4 B is related to subtle differences in electronic structure as shown by a Natural Bonding Orbital (NBO) analysis of the respective silanes (Section S5.1.7). The composition of the Si‐based hybrid in the Si−H NBO of tris‐*N*‐skatyl silane (**A**) is sp^2.04^d^0.02^. The s‐character significantly increases in the cage compounds **B** (sp^2.01^d^0.02^), **C** (sp^1.90^d^0.02^), and in the cationic silane **2** (sp^1.82^d^0.02^). This translates into a subtle shift in bond polarization: the Si−H bond remains overall polarized towards hydrogen, as was inferred from the interaction of **2** with dioxane in the solid state (*vide supra*), but the Si contribution to the Si−H bonding NBO increases from 41.3 % in **A** to 41.8 % in **B**, 42.5 % in **C**, and 43.5 % in **2**. This relative shift reduces Δ*E*_1_ and makes strained silanes more electronically similar to their respective anions with the silicon lone pair in the latter ranging in hybridization from sp^0.47^ in **B** to sp^0.41^ in TSMPSi (**1**). Overall, this makes heterolytic Si−H bond dissociation in strained silanes more favorable than in unstrained ones.

### Reactivity of the Zwitterionic Silanide TSMPSi (1)

**Scheme 2:**
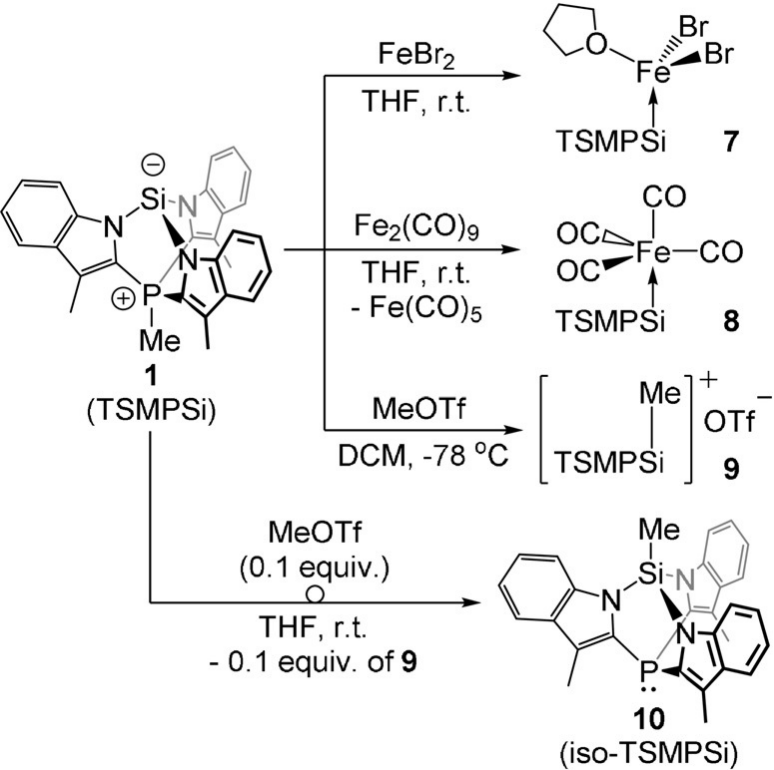
Reactivity of zwitterionic TSMPSi (**1**).

The high acidity of [TSMPSiH]^+^ (**2**) suggests that the stabilized Si anion in TSMPSi (**1**) should behave as a relatively weak nucleophile, which was assessed by a series of reactions (Scheme 2). Treatment with FeBr_2_ in THF afforded the insoluble stable complex (TSMPSi)FeBr_2_(THF) (**7**), which was identified crystallographically. This reaction is likely solubility‐driven, since no complexation was observed with Fe(OTf)_2_ and Fe(acac)_2_, suggesting only a weak interaction with the Fe^II^ centers. Reaction of **1** with Fe_2_(CO)_9_ afforded (TSMPSi)Fe(CO)_4_ (**8**) as a stable complex, suggesting that the softer (in the HSAB sense) Fe(CO)_4_ fragment is a better match for the soft Si^−^ center in **1**. Finally, reaction of **1** with 1 equiv of MeOTf resulted in methylation at silicon to form the cationic cage compound [TSMPSiMe]^+^OTf^−^ (**9**). Quaternization of the silicon atom in TSMPSi (**1**) is evidenced by a change of the ^29^Si NMR chemical shift from −48.0 ppm in **1** to 34.4 and −22.5 ppm in **8** and **9**, respectively. Additionally, the ^29^Si NMR signal in **9** shows as a pentet (*J*_Si,H_≈*J*_Si,P_=8.1 Hz) consistent with the assigned structure.

Relatively low donicity of the silicon center in TSMPSi (**1**) is also apparent from analysis of the CO‐stretching frequencies of its complexes with metal carbonyls (Section S5.2.1), which places **1** in the vicinity of silylenes and P(NMe_2_)_3_ rather than other silicon anions. An obvious explanation for this is the presence of a positive charge in the molecule, which electrostatically stabilizes the negative charge on silicon rendering it less available for bonding. The second factor that contributes to weak nucleophilicity is a build‐up of strain that occurs upon quaternization. The strain penalty can be separated from electrostatic effects and approximated by analyzing a series of homodesmotic equations involving a Si‐tethered analogue of **1** (Section S5.1.5). There, complexation with an FeBr_2_(THF) center leads to a 4.5 kcal mol^−1^ increase in strain, whereas methylation and protonation provide 8.3 and 9.0 kcal mol^−1^ of an increase, respectively, confirming a likely contribution of ring strain to the weakened nucleophilicity of **1**.

The isolated zwitterion **1** is kinetically stable in solution despite the presence of a nucleophilic Si^−^ site and an electrophilic P^+^‐Me unit in the same molecule. Unexpectedly, exposing **1** to 0.1 equiv of MeOTf was found to result in nearly complete isomerization of **1** to the charge‐neutral analogue iso‐TSMPSi (**10**) (Section S5.2.2). This suggests that the cation [TSMPSiMe]^+^ is subject to nucleophilic attack at the phosphonium methyl group by a molecule of **1**, forming **10** and generating a new molecule of [TSMPSiMe]^+^ (Scheme S7). This increased electrophilicity of the P‐bound methyl group in [TSMPSiMe]^+^ likely originates from a combination of its overall positive charge and increased ring strain. As a matter of fact, nearly complete isomerization also occurs in the presence of 0.25 equiv of HBAr^F^_4_⋅2 Et_2_O to form **2^BARF^** in situ (Section S5.2.2). The loss of the P‐bound methyl group from either [TSMPSiH]^+^ (**2**) or [TSMPSiMe]^+^ reduces the strain of the cage structure, which provides an additional driving force for this “strain‐release methyl transfer” reaction.

### Reactivity of the Cationic Silane [TSMPSiH]^+^ (2)

**Scheme 3:**
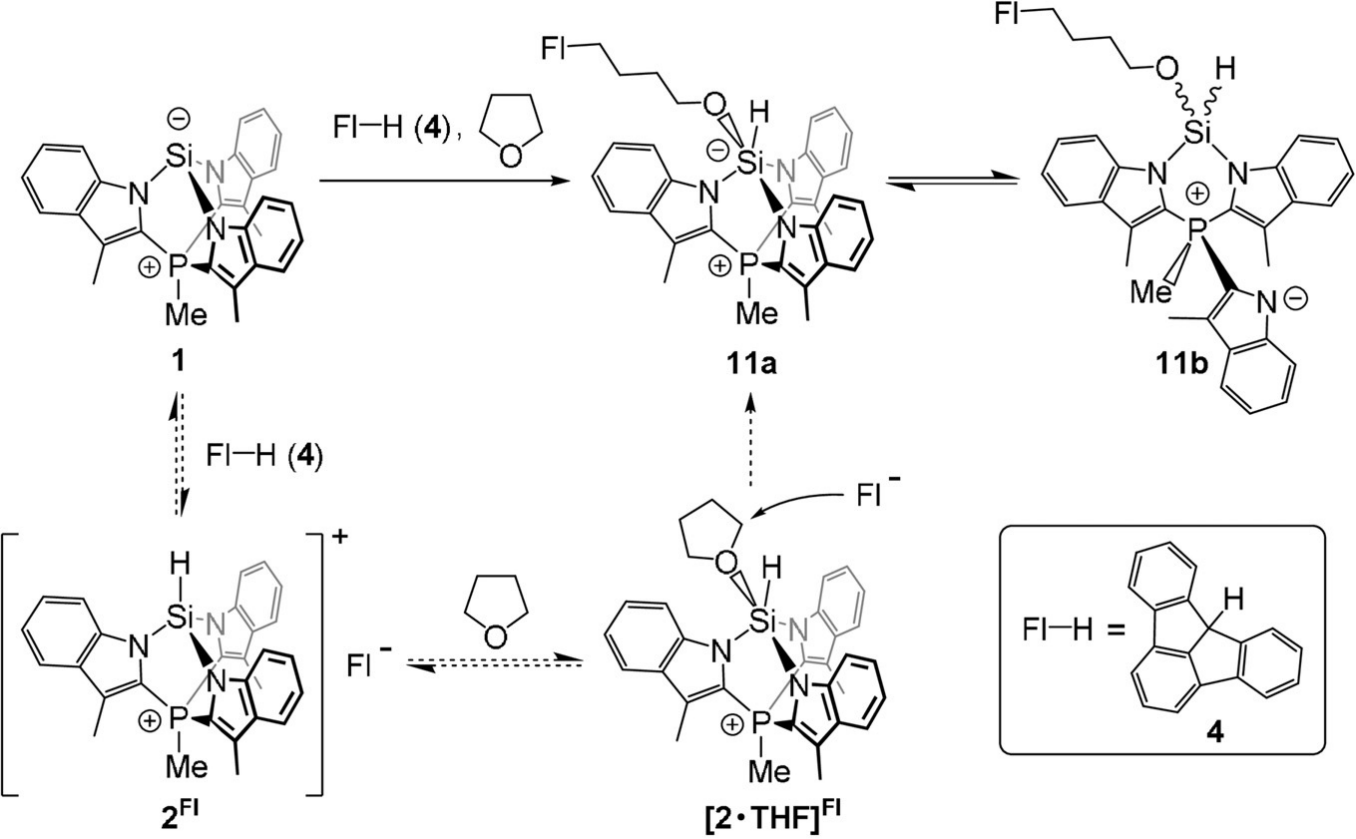
THF ring‐opening with fluoradene (**4**). Suggested reaction mechanism is shown with dashed arrows.

Interestingly, recording NMR spectra of [TSMPSiH]^+^BArF_4_^−^ (**2^BARF^**) in [D_8_]THF instead of [D_2_]DCM results in dramatic changes in the ^29^Si signal. The doublet of doublets shifts to −91.0 ppm (vs. −46.4 ppm in [D_2_]DCM) with ^1^ *J*_Si,H_=368.4 Hz (vs. 318.4 Hz) and ^3^ *J*_Si,P_=6.7 Hz (vs. 8.0 Hz). We propose that this is due to coordination of a THF molecule resulting in a pentacoordinate silicon centre (**2⋅THF** in Scheme 3). NMR parameters calculated for **2⋅THF** using DFT reproduce the experiment reasonably well, being −104.9 ppm, −392.7 and −7.3 Hz, respectively (Section S4.2). Importantly, the ^31^P NMR signal of **2^BARF^** only shifts from −7.6 ppm in [D_2_]DCM to −7.0 ppm in [D_8_]THF, ruling out THF interaction with the phosphonium center. Another point of note is that ^1^H and ^13^C spectra of **2^BARF^** in [D_8_]THF remain in line with C_3_ symmetry, in contrast with the expected breaking of symmetry upon coordination of THF. This suggests a fluxional process such as Berry‐like pseudorotation or reversible dissociation.

THF coordination to silicon in [TSMPSiH]^+^ (**2**) is key to the observed reactivity of TSMPSi (**1**) with the CH‐acidic fluoradene (**4**, p*K*_a_^DMSO^=10.5) in [H_8_]THF (Scheme 3). Next to a moderate amount of the isomerization product iso‐TSMPSi (**10**; *vide supra*), the main product was spectroscopically identified as the THF ring‐opening product **11 a**, which exists in a dynamic equilibrium with the open form **11 b**. ^1^H NMR spectra of the mixture (**11 a**/**b**) in [D_8_]THF at 25 °C (Section S5.3.1) display a number of broadened peaks corresponding to protons of the Si−H, methyl phosphonium, and aromatic groups as well as four methylene signals between 0.75 and 3.25 ppm. The latter disappear if the reaction is conducted in [D_8_]THF instead of [H_8_]THF, which indicates that they belong to a −(CH_2_)_4_− fragment from a THF ring‐opening reaction. Moreover, ^1^H COSY spectrum shows that they form an isolated spin system (Section S5.3.2), while the NOE spectra (Section S5.3.3) indicate that the termini of the −(CH_2_)_4_− fragment are located in spatial proximity to one aromatic doublet each, with the oxygen‐bound terminus also being close to a Si−H proton.

The formation of **11 a**/**b** can be explained by the generation of a small amount of the Si^IV^ cation [TMSPSiH]^+^ (**2**) as the fluoradenide salt **2^Fl^** in an acid/base equilibrium with Si^II^ compound TSMPSi (**1**) and **4**. The cation then acts as a strain‐release Lewis acid for THF, generating the complex **2⋅THF** characterized above (Scheme 3), which undergoes ring opening upon nucleophilic attack by the deprotonated fluoradene (**4**). These last steps can also be interpreted as the activation of THF by the transient frustrated Lewis pair **2^Fl^**.^[46]^ Interestingly, products similar to **11 a**/**b** form with other tested fluorenes of p*K*_a_^DMSO^ within 8.1–11.6 (Section S5.3.4), while less acidic fluorenes exclusively lead to isomerisation to iso‐TSMPSi (**10**). This can be understood from the fact that stronger acids will generate higher equilibrium concentration of the fluorenide anion, thus opening a kinetic pathway for the THF ring opening. This reaction sequence involves the insertion of both the Si center and a neutral molecule (THF) into a C−H bond coupled with a formal oxidation state change from Si^II^ in TSMPSi (**1**) to Si^IV^ in [TMSPSiH]^+^ (**2**), which presents intriguing similarities with transition metal‐mediated processes. In the overall reaction, the silicon center sequentially acts as a nucleophile (base) and an electrophile (Lewis acid), demonstrating biphilic character that can be traced back to ring strain in [TSMPSiH]^+^ (**2**).

### Fluxionality of the THF Ring‐Opening Product 11 a/b

The THF ring‐opening product **11 a**/**b** displays rich dynamic behavior in solution, which could be further elucidated by using variable‐temperature NMR spectroscopy. Between −89 and 60 °C, **11 a**/**b** displays only one ^31^P NMR signal with a temperature‐dependent chemical shift (Section S5.3.5), indicating a rapid temperature‐dependent equilibrium between two forms **11 a** and **11 b**. A regressive thermodynamic analysis shows that the transition starts above −60 °C and is 78.5 % complete at 25 °C with Δ_r_ *H*=10.4 kcal mol^−1^ and Δ_r_ *S*=37.5 cal mol^−1^ K^−1^ (Section S5.3.5). These thermodynamic parameters are consistent with a ring‐opening equilibrium between **11 a** and **11 b** involving breaking of a Si−N bond (Scheme 3). Confirming this interpretation, the ^29^Si NMR signal of **11 a**, −120.2 ppm at −89 °C indicative of a penta‐ or hexacoordinate silane,^[47]^ shifts to −74.9 ppm at 25 °C (Section S5.3.6). With consideration of the 78.5 % complete transition at 25 °C, this yields an extrapolated value of −62.5 ppm for pure **11 b**, which is typical for tetrahedral silanes.^[47]^ Our assignment of the temperature‐dependent speciation in solution is supported by DFT‐calculated ^29^Si NMR chemical shifts and ^1^ *J*_Si,H_ coupling constants for possible geometries on silicon using truncated molecular models (Section S5.3.6).

**Scheme 4:**
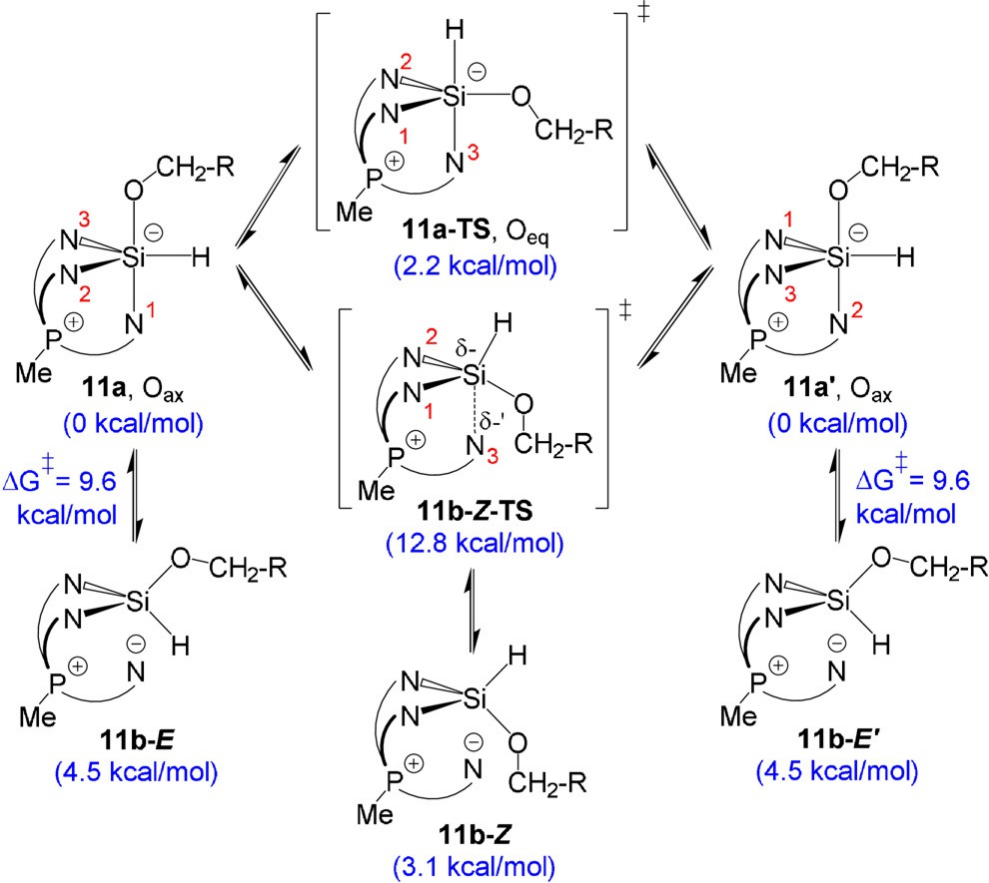
Cyclic exchange and ring opening in a truncated molecular model of **11 a** (R=H, 3‐methylindoles were reduced to pyrroles) analyzed at B3LYP‐GD3BJ/6–31+G* level of theory. Calculated Δ*G* energies are shown in blue.

An NOE correlation between the oxygen‐bound CH_2_ group and the aromatic methyl group (Section S5.3.3) indicates that **11 b** at least partially exists as the *Z*‐stereoisomer with the free indolide and the alkoxy substituent on the same side of the central six‐membered ring (**11 b‐*Z*** in Scheme 4). This may appear surprising at first sight, considering that the ring‐opening should proceed from the lowest‐energy configuration of **11 a**, which, according to DFT, has a trigonal bipyramidal silicon centre with an axial alkoxy group. There, dissociation of the most labile apical Si−N bond should lead to the **11 b‐*E*** isomer (Scheme 4). Consequently, **11 b‐*Z*** likely forms via another mechanism that involves a certain degree of geometric fluxionality around silicon in **11 a**, sampling geometries leading to **11 b‐*Z*** upon reversible dissociation. Supporting the presence of an additional dynamic process in the system, ^1^H spectra of **11 a**/**b** (Section S5.3.1) show only one aromatic methyl signal, whereas both the closed (**11 a**) and the open (**11 b**) isomers should display two signals in a ratio of 2:1 as a result of breaking of the C_3_ symmetry.

To account for these observations, we undertook a computational study on a truncated model of **11 a** (3‐methylindoles were reduced to pyrroles, R=H in Scheme 4), supporting the overall exchange and isomerization mechanisms depicted in Scheme 4. The apical and equatorial N‐atoms can swap in a single step following the M2 positional exchange mechanism^[48]^ with a low barrier of 2.2 kcal mol^−1^ (Section S5.3.7), explaining the above spectroscopic observations. This exchange mechanism can be thought of as two consecutive Berry‐type deformations,^[49a]^ the product of the first one being the transition state for the overall process, **11 a‐TS**. While dissociation of the apical N−Si bond from **11 a**/**11 a′** readily yields **11 b‐*E***/**11 b‐*E*′** with a barrier of 9.6 kcal mol^−1^, a similar process leading to **11 b‐*Z*** would have to start from the transition state structure **11 a‐TS**, in which the hydride substituent occupies an apical position. This situation may indicate the presence of a bifurcation on the potential energy surface,^[50]^ which is supported by a two‐dimensional relaxed potential energy surface scan (Section S5.3.8). The bifurcation is best understood starting from the 4‐coordinate structure **11 b‐*Z***: as the N‐atom gradually approaches the Si center, the molecule first overcomes the transition state **11 b‐*Z*‐TS** (12.8 kcal mol^−1^) and then “avoids” the second transition state **11 a‐TS** to fall directly onto either of the degenerate energy minima **11 a** or **11 a′**.

Overall, while the NOE spectra (Section S5.3.3) indicate the presence of **11 b‐*Z***, the comparable free energy of the isomer **11 b‐*E*** and low computed barriers for interconversion suggest that both stereoisomers likely coexist with **11 a** in a thermal equilibrium. These observations demonstrate that, besides its ability to support facile interconversion of Si^II^ and Si^IV^ species, the strained TSMP platform confers much conformational flexibility to the latter, as it allows virtually all thermodynamically accessible 4‐coordinate and 5‐coordinate geometries to be sampled at room temperature or below.

## Conclusion

The constrained valence angles around the silicon atom at the bridgehead position of the bicyclic cationic silane [TSMPSiH]^+^ (**2**) have a profound effect on its reactivity.

First, at odds with the general hydridic character of silicon‐bound hydrogen atoms, [TSMPSiH]^+^ (**2**) exhibits an exceptionally low p*K*_a_^DMSO^ within 4.7–8.1, which makes it more acidic than phenol, benzoic acid (p*K*_a_^DMSO^ of 18.0^[14]^ and 11.1,^[15]^ respectively) and the few silanes of which p*K*_a_^DMSO^ was reported.[^24^, ^31^] While this high acidity originates in part from the electron‐withdrawing effect of the substituents on silicon and overall positive charge of **2**, it is significantly enhanced by the unusually acute N^Si^N angles imposed by the heterobicyclo[2.2.2]octane scaffold. Namely, DFT calculations suggest that strain increases the s‐character of the Si−H bonding pair, polarizing the bond towards silicon and facilitating its heterolytic dissociation.

Then, protonation (or methylation) of the anionic Si atom in TSMPSi (**1**) increases the reactivity of the opposing methylphosphonium unit, which can transfer a methyl group with release of strain. This opens up a charge neutralization pathway to form the phosphine/methylsilane isomer iso‐TSMPSi (**10**) by intermolecular methyl transfer.

Finally, next to its high Brønsted acidity, [TSMPSiH]^+^ (**2**) also behaves as a strain‐release Lewis acid, coordinating a THF molecule so that the silicon atom assumes a trigonal bipyramidal geometry. This is accompanied by activation of α‐carbons of the THF ring, which makes it susceptible to such weak nucleophiles as highly‐stabilized aromatic anions. In particular, its conjugate base TSMPSi (**1**) reacts with fluoradene (**4**) in THF to afford the linear product **11 a**/**b**, which forms by nucleophilic ring opening of a THF molecule coordinated to [TSMPSiH]^+^ (**2**). In this process, the Si center sequentially acts as a nucleophile (base) and then as an electrophile (Lewis acid) to facilitate the activation of two relatively unreactive molecules, suggesting the potential of such cage compounds as biphilic main‐group centers. This reaction sequence also illustrates the ability of the TSMP scaffold to support both the Si^II^ and Si^IV^ states along with the processes that interconvert them under mild conditions. In addition, the trigonal bipyramidal silicon center in the addition product **11 a**/**b** undergoes several facile fluxional processes (positional exchange and reversible Si−N bond dissociation), illustrating the flexibility of the TSMPSi (**1**) platform in terms of accessible geometries.

These observations collectively illustrate how a combination of charge, inductive effects and strain can significantly manipulate the properties of a silicon atom leading to unusual reactivity. The various reactive pathways accessible to the strained cage structure [TSMPSiH]^+^ (**2**) suggest that this or a related platform may serve as an entry point into novel bond activation strategies based on the Si^II^/Si^IV^ couple.

## Conflict of interest

The authors declare no conflict of interest.

## Supporting information

As a service to our authors and readers, this journal provides supporting information supplied by the authors. Such materials are peer reviewed and may be re‐organized for online delivery, but are not copy‐edited or typeset. Technical support issues arising from supporting information (other than missing files) should be addressed to the authors.

Supplementary
