# Supplementary material for: Strain‐Modulated Reactivity: An Acidic Silane
Source: Angew Chem Int Ed Engl. 2021 Mar 12;60(17):9618–26. doi: 10.1002/anie.202015960 (PMC8252520; doi:10.1002/anie.202015960)
Supplement: Supplementary file 1 — Supplementary [file ANIE-60-9618-s001.pdf]

Angewandte Chemie
Eine Zeitschrift der Gesellschaft Deutscher Chemiker

Supporting Information

Strain-Modulated Reactivity: An Acidic Silane

*Serhii Tretiakov, Léon Witteman, Martin Lutz, and Marc-Etienne Moret**

anie_202015960_sm_miscellaneous_information.pdf

Contents[a]

S1 General remarks 4
S2 Synthesis and characterization 5
S2.1 Silicon and germanium compounds 5
TSMPSi (1) 5
$[TSMPSiH]^+BAr^F_4{}^-$ (2^{BARF}) 6
TSMPGe (12) 8
(TSMPSi)$FeBr_2$(THF) (7) 9
(TSMPSi)$Fe(CO)_4$ (8) 9
(TSMPGe)$Fe(CO)_4$ (17) 10
$[TSMPSiMe]^+OTf^-$ (9) 10
iso-TSMPSi (10) 11
S2.2 Fluorenes 12
Fluoradene (4) 12
1,3-di(9*H*-fluoren-9-ylidene)propane (5) 13
9-Phenyl-9*H*-fluorene (14) 15
9-Pentafluorophenyl-9*H*-fluorene (15) 15
9-Phenylsulfonyl-9*H*-fluorene (16) 16
9-Cyano-9*H*-fluorene (17) 16
S2.3 Additional experiments 16
Reaction of TSMPSi (1) and TSMPGe (12) with HCl 16
pK_a bracketing experiments in dioxane-d_8 17
Attempted complexation of TSMPSi (1) with $Fe(OTf)_2$ and $Fe(acac)_2$ 17
Reactivity of TSMPSi (1) with CH-acids in THF 17
S3 X-ray crystal structure determinations 19
TSMPSi (1) 19
$[TSMPSiH]^+BAr^F_4{}^-$ (2^{BARF}) 20
TSMPGe (12) 22
(TSMPSi)$FeBr_2$(THF) (7) 23
iso-TSMPSi (10) 25
(TSMP–H)GeCl (13) 26
S4 Computational studies 28

[a] For the reader's convenience, this document is enhanced with hyperlinks. Clicking on any mention of a section/table/scheme/figure/chart in the text will bring the reader to the object that was referred to.

S4.1 General remarks 28

S4.2 Calculated NMR parameters for isolated compounds 28

S5 Peripheral discussions 31

S5.1 Synthesis and Brønsted acidity of [TSMPSiH]$^{+}$ (2) 31

S5.1.1 The importance of a non-coordinating anion for 2^{BARF} 31

S5.1.2 Base-catalyzed isomerization of fluorene 5 32

S5.1.3 Comparison of geometries of P^{+}- and Si-tethered analogues 34

S5.1.4 Gas-phase acidities 35

S5.1.5 On the ring strain in bicyclic systems 37

S5.1.6 Anionic NLMO on a silicon in TSMPSi (1) 39

S5.1.7 Natural Bonding Orbital (NBO) analysis of the deprotonation 39

S5.2 Properties and reactivity of the zwitterionic TSMPSi (1) 41

S5.2.1 On donicity of TSMPSi (1) 41

S5.2.2 Remarks on isomerization into iso-TSMPSi (10) 47

S5.3 Properties and reactivity of the cationic silane [TSMPSiH]$^{+}$ (2) 48

S5.3.1 ^{1}H NMR of the THF ring-opening reaction 48

S5.3.2 ^{1}H gCOSY NMR of the THF ring-opening reaction 49

S5.3.3 ^{1}H 1D NOE NMR of the THF ring-opening reaction 50

S5.3.4 Reactions with various fluorenes 51

S5.3.5 Variable temperature ^{1}H and ^{31}P NMR of the THF ring-opening reaction 54

S5.3.6 Variable temperature ^{29}Si NMR of the THF ring-opening reaction 57

S5.3.7 Positional exchange around a silicon atom 60

S5.3.8 Relaxed 2D PES scan 62

S6 Literature references 66

S7 Spectra of isolated compounds 75

S8 Computational coordinates 186

S1 General remarks

All reactions were conducted under a nitrogen atmosphere with strict exclusion of moisture by using standard glovebox or Schlenk techniques.

Acetonitrile, diethyl ether, toluene and *n*-hexane were dried with an MBRAUN MB SPS-79 system. Acetonitrile was additionally dried by passing through a column of activated alumina after being kept over ca. 5 wt.% of 3Å molecular sieves over 48h. THF and dioxane were distilled from benzophenone/Na. DCM and aniline were distilled from CaH_2. Benzene was dried over ca. 5 wt.% of 3Å molecular sieves over 48h. Dried solvents were degassed by sparging with dry nitrogen for 30 min and stored over molecular sieves in a glovebox, except for acetonitrile which was stored without the sieves. THF-d_8, pyridine-d_5 and dioxane-d_8 were purchased from ABCR, other deuterated solvents were acquired from Cambridge Isotope Laboratories, Inc. Deuterated solvents were dried as indicated above for their proteo-analogues, except for dioxane-d_8 which was dried by passing through a column of activated alumina. Pyridine-d_5 was dried by distillation over CaH_2. Dried deuterated solvents were degassed by four freeze-pump-thaw cycles and stored in a glovebox over molecular sieves, except for acetonitrile-d_3 which was stored without the sieves.

$Fe(OTf)_2$ was purchased from ABCR and 9-cyano-9*H*-fluorene (**17**) was acquired from Enamine Ltd. All other chemicals were purchased from Sigma-Aldrich. $FeBr_2$ was acquired in anhydrous form. Potassium hydride was supplied as a 30 wt.% suspension in mineral oil, which was washed away with several portions of *n*-hexane before further use. 2,6-Lutidine was dried by distillation over CaH_2 and degassed by bubbling dry dinitrogen for 30 min, after which it was stored in a glovebox over 4Å molecular sieves. All other commercially obtained chemicals were used as received. The $FeCl_2{\cdot}1.5THF$ adduct was prepared by extraction of $FeCl_2$ with anhydrous THF using a Soxhlet extractor. Potassium graphite (KC_8) and Idipp·$SiCl_2$ were synthesized according to literature procedures.[1,2] After purification, according to ^1H NMR, Idipp·$SiCl_2$ was still contaminated with ~10 wt.% of unidentified Idipp-containing impurities. $NaBAr^F_4$ was prepared following a reported procedure.[3] Before drying, an additional recrystallization step from fluorobenzene was required in order to achieve higher purity. $HBAr^F_4{\cdot}2Et_2O$ was prepared from $NaBAr^F_4$ according to the literature procedure.[4] The TSMPK_2 salt (**3**) was synthesized following the published procedure.[5]

Unless otherwise stated, the NMR measurements were performed at 298K on a Varian VNMRS400 or Varian MRF400 spectrometer, chemicals shifts are reported relative to TMS with the residual solvent signal as internal standard.[6] All NMR experiments involving air-sensitive compounds were conducted in J. Young NMR tubes under nitrogen atmosphere. Peak multiplicity was quoted as s (singlet), d (doublet), t (triplet) and so on. In cases of unresolved couplings that strongly affected the line shape of individual components of an otherwise well-defined multiplet, the effective multiplicity was quoted as 's', 'd', 't' and so on. ASAPHMQC NMR experiments were conducted using the corresponding pulse sequence[7] as implemented in the VnmrJ 4.2 software.[8] IR spectra were recorded on a Perkin-Elmer Spectrum Two FT-IR spectrometer. The bands were classified by an absorption intensity as: very weak (VW; 0-10% of the most intense absorption in the spectrum), weak (W; 10-30%), medium (M; 30-60%), strong (S; 60-90%), very strong (VS; 90-100%). UV-Vis spectra were measured on a PerkinElmer Lambda 35 spectrometer. ESI-MS measurements were performed on a Waters LCT Premier XE KE317 spectrometer. Elemental analysis was conducted by Medac Ltd.

S2 Synthesis and characterization

S2.1 Silicon and germanium compounds

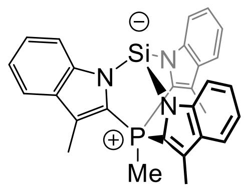

1

TSMPSi (1). A solution of Idipp·$SiCl_2$ (90 wt.% purity, 3.055 g, 5.630 mmol, 1.050 equiv.) in THF (20.0 ml) was added dropwise to dipotassium salt **3** (contains 21.5 wt.% of THF, 3.494 g, 5.360 mmol, 1.000 equiv.) in THF (140 ml) at -78 °C. The mixture was stirred at low temperature for 4h and then allowed to warm up to room temperature overnight. Next day, the reaction was filtered, and the solid was washed with THF (3 x 10.0 ml). The solvent from combined organic fractions was removed *in vacuo*. The solid was dissolved in a minimal amount of PhMe/CH_3CN 1:1 (v/v) solvent mixture, carefully topped up with an equal volume of Et_2O and placed into a freezer overnight at -36 °C. The precipitate was filtered off the next day and, in order to remove the traces of other solvents, was thrice resuspended in THF (10.0 ml) followed by drying *in vacuo*. The procedure yielded fine light-beige powder. The residual THF content of 19.0 wt.% was determined using quantitative ^{1}H NMR measurements in 4:1 benzene-d_6/acetonitrile-d_3 solvent mixture. With the product mass of 2.017 g, this gives 66.0% yield. Crystals suitable for X-ray diffraction analysis were grown by vapor diffusion of *n*-hexane into a solution of **1** in benzene at room temperature.

The ^{1}H and ^{13}C NMR spectra of **1** show a single set of signals in aromatic region indicating C_3 symmetry that corresponds to heterobicyclo[2.2.2]octane topology. Moreover, both ^{1}H and ^{29}Si spectra show a good agreement with the DFT-calculated NMR parameters (see *Section S4.2*). Some NMR spectra are contaminated with small amounts of isomer **10**, which has better solubility than **1** in most solvents. In case of an overlap with a solvent signal, where possible, integral intensities in ^{1}H NMR spectra were extracted using MNova[9] peak deconvolution tool. **^{1}H NMR** (400 MHz, Pyridine-d_5) δ 8.54 (d, $J_{H,H}$ = 8.4 Hz, 1H, Ar-H), 7.61 (d, $J_{H,H}$ = 8.0 Hz, 1H, Ar-H), 7.41 (t, $J_{H,H}$ = 7.6 Hz, 1H, Ar-H), 7.20 (t, $J_{H,H}$ = 7.3 Hz, 1H, Ar-H), 4.07 (d, $J_{H,P}$ = 16.2 Hz, 1H, P^+-CH_3), 2.52 (d, $J_{H,P}$ = 1.8 Hz, 3H, Ar-CH_3). ^{1}H NMR (400 MHz, THF-d_8) δ 8.11 (d, $J_{H,H}$ = 8.5 Hz, 1H, Ar-H), 7.46 (d, $J_{H,H}$ = 8.0, 1.0 Hz, 1H, Ar-H), 7.18 (t, $J_{H,H}$ = 7.6 Hz, 1H, Ar-H), 6.97 (t, $J_{H,H}$ = 7.4 Hz, 1H, Ar-H), 3.67 – 3.56 (m, obscured by the overlap with the THF-h_8 peak, P^+-CH_3), 2.59 (d, $J_{H,P}$ = 1.8 Hz, 3H, Ar-CH_3). ^{1}H NMR (400 MHz, Methylene Chloride-d_2) δ 8.14 (d, $J_{H,H}$ = 8.5 Hz, 1H, Ar-H), 7.52 (d, $J_{H,H}$ = 8.0 Hz, 1H, Ar-H), 7.27 (t, $J_{H,H}$ = 7.7 Hz, 1H, Ar-H), 7.06 (t, $J_{H,H}$ = 7.4 Hz, 1H, Ar-H), 3.28 (d, $J_{H,P}$ = 15.8 Hz, 1H, P^+-CH_3), 2.59 (d, $J_{H,P}$ = 1.9 Hz, 3H, Ar-CH_3). ^{1}H NMR (400 MHz, 1,4-Dioxane-d_8) δ 8.14 (d, $J_{H,H}$ = 8.5 Hz, 1H, Ar-H), 7.46 (d, $J_{H,H}$ = 7.9 Hz, 1H, Ar-H), 7.23 (t, $J_{H,H}$ = 7.7 Hz, 1H, Ar-H), 7.00 (t, $J_{H,H}$ = 7.5 Hz, 1H, Ar-H), 3.40 (d, $J_{H,P}$ = 16.3 Hz, 1H, P^+-CH_3), 2.58 (d, $J_{H,P}$ = 1.8 Hz, 3H, Ar-CH_3). ^{1}H NMR (400 MHz, Benzene-d_6) δ 8.55 ('d', $J_{H,H}$ = 8.5 Hz, 1H, Ar-H), 7.48 ('d', $J_{H,H}$ = 8.0 Hz, 1H, Ar-H), 7.23 ('t', $J_{H,H}$ = 7.6 Hz, 1H, Ar-H), 7.13 ('t', $J_{H,H}$ = 7.6 Hz, 1H, Ar-H), 2.08 – 1.98 (m, 4H, P^+-CH_3, Ar-CH_3). ^{1}H NMR (400 MHz, Acetonitrile-d_3/Benzene-d_6 1:4 (v/v); referenced against the residual benzene peak at 7.16 ppm) δ 8.21 ('d', $J_{H,H}$ = 8.5 Hz, 1H, Ar-H), 7.43 ('d', $J_{H,H}$ = 8.1 Hz, 1H, Ar-H), 7.14 ('t', J = 7.8 Hz, overlaps with residual benzene peak, Ar-H), 6.99 ('t', $J_{H,H}$ = 7.3 Hz, 1H, Ar-H), 3.02 (d, $J_{H,P}$ = 16.0 Hz, 1H, P^+-CH_3), 2.40 (d, $J_{H,P}$ = 1.9 Hz, 3H, Ar-CH_3). **^{13}C NMR** (101 MHz, Pyridine-d_5) δ 147.7 (d, $J_{C,P}$ = 10.9 Hz, indole-C8), 130.9 (d, $J_{C,P}$ = 14.2 Hz, indole-C9 or C3), 126.2 (C^{Ar}-H), 123.2 (d, $J_{C,P}$ = 17.5 Hz, indole-C3 or C9), 121.8 (d, $J_{C,P}$ = 115.7 Hz, indole-C2), 121.7 (d, $J_{C,P}$ = 1.6 Hz, C^{Ar}-H), 121.4 (C^{Ar}-H), 115.3 (d, $J_{C,P}$ = 2.0 Hz, C^{Ar}-H), 11.0 (Ar-CH_3), 2.3 (d, $J_{C,P}$ = 56.2 Hz,

P^+-CH_3). **^{31}P NMR** (162 MHz, Pyridine-d_5) δ -7.7 (q, $J_{P,H}$ = 16.1 Hz, carbon satellites: $J_{P,C}$ = 115.6, 55.9, 17.0, 13.8, 10.5 Hz). ^{31}P NMR (162 MHz, THF-d_8) δ -7.3 (q, $J_{P,H}$ = 16.2 Hz, carbon satellites: $J_{P,C}$ = 116.1 Hz). ^{31}P NMR (162 MHz, Methylene Chloride-d_2) δ -10.2 (q, $J_{P,H}$ = 16.0 Hz, additional couplings resulting into carbon satellites: $J_{P,C}$ = 117.3 Hz). ^{31}P NMR (162 MHz, Dioxane-h_8) δ -9.5. ^{31}P NMR (162 MHz, Benzene-d_6) δ -10.9 (q, $J_{P,H}$ = 16.0 Hz). ^{31}P NMR (162 MHz, Acetonitrile-d_3/Benzene-d_6 1:4 (v/v)) δ -9.70 (q, $J_{P,H}$ = 16.1 Hz). **^{29}Si NMR** (79 MHz, Pyridine-d_5) δ -47.5 (d, $J_{Si,P}$ = 4.4 Hz). ^{29}Si NMR (79 MHz, THF-d_8) δ -48.0 (d, $J_{Si,P}$ = 4.3 Hz). No satisfactory elemental analysis could be obtained due to the high reactivity of **1**.

Appended spectra: NMR in pyridine-d_5: ^{1}H (*Figure S23*), ^{13}C{^{1}H} (*Figure S24*), ^{1}H-^{13}C ASAPHMQC (*Figure S25*), ^{31}P{^{1}H} (*Figure S26*), ^{31}P (*Figure S27*), ^{29}Si{^{1}H} (*Figure S28*); NMR in THF-d_8: ^{1}H (*Figure S29*), ^{31}P{^{1}H} (*Figure S30*), ^{31}P (*Figure S31*), ^{29}Si{^{1}H} (*Figure S32*); NMR in DCM-d_2: ^{1}H (*Figure S33*), ^{31}P{^{1}H} (*Figure S34*), ^{31}P (*Figure S35*); NMR in dioxane-d_8: ^{1}H (*Figure S36*), ^{31}P{^{1}H} (*Figure S37*); NMR in benzene-d_6: ^{1}H (*Figure S38*), ^{31}P{^{1}H} (*Figure S39*); NMR in acetonitrile-d_3/benzene-d_6 1:4 (v/v): ^{1}H (*Figure S40*), ^{31}P (*Figure S41*).

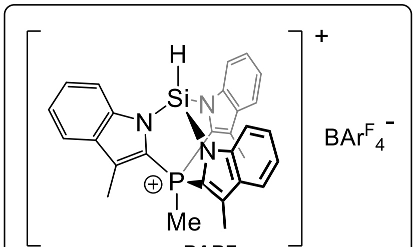

2^{BARF}

[TSMPSiH]$^+$BAr$^F{}_4^-$ (2^{BARF}). Depending on the solvent, compound **2^{BARF}** shows two different sets of NMR signals consistent with different coordination environment of the silicon. Namely, in DCM-d_2, ^{29}Si shifts are consistent with a tetrahedral silicon atom, whereas in THF-d_8 ^{29}Si spectra indicate pentacoordination. Below, we provide two alternative procedures that can be used in order to synthesize **2^{BARF}**: one which is THF-free (*Procedure A*) and another in THF as a reaction solvent (*Procedure B*). While the former, according to NMR, yields some amount of unidentified side-products and, thus, only qualifies as "generation", the latter provides clean **2^{BARF}** and can be considered preparative.

Procedure A (THF-free generation). All manipulations were performed in a J. Young NMR tube. Silanide **1** (contains 19.0 wt.% of THF, 0.035 g, 0.061 mmol, 1.0 equiv.) was suspended in 2.0 ml of dioxane. The suspension was cooled in a freezer until it solidified. This was followed by drying *in vacuo* to a constant mass, resuspending in dioxane, cooling and another cycle of drying. Solid HBAr$^F{}_4$·2Et_2O (0.062 g, 0.061 mmol, 1.0 equiv.) suspended in 1.0 ml of dioxane was then added. The suspension was quickly stirred, upon which the mixture almost cleared up and then immediately turned into a gelatinous white mass. It was, again, cooled in a freezer and dried *in vacuo* to a constant mass. The white solid was completely redissolved in ~0.5 ml of DCM-d_2 for the NMR measurements. Crystals suitable for X-ray diffraction analysis were grown by layering a DCM solution of **2^{BARF}** with dioxane in a J. Young NMR tube, and leaving it standing for 3 days.

Both ^{1}H and ^{13}C NMR spectra in DCM-d_2 are consistent with a heterobicyclo[2.2.2]octane topology of the cationic silane **2**. Even though the signal of the Si–H hydrogen atom is obscured by aromatic multiplets, its position can be clearly determined from ^{1}H-^{29}Si ASAPHMQC spectra. The NMR spectra are sufficiently clean in order to make conclusions about atomic connectivity. Yet, there is a number of unidentified trace impurities. Furthermore, NMR data suggest the presence of a small amount (ca. 15%) of a contaminating BAr$^F{}_4^-$ salt, possibly NaBAr$^F{}_4$ or HBAr$^F{}_4$. In order to assign

$BAr^F{}_4^-$-derived signals, we used the spectra of $NaBAr^F{}_4$ in THF-d_8 as a reference (*see Procedure B*). While the chemical shifts slightly differ from those in DCM-d_2, the number of signals and their multiplicity still hold and, therefore, can be used for the assignment. Experimental NMR parameters show a good agreement with the DFT-calculated values (see *Section S4.2*). **^{1}H NMR** (400 MHz, Methylene Chloride-d_2) δ 7.78 – 7.70 (m, 11H of which 8H: C(2)^{Ar}-H of $BAr^F{}_4^-$ + 3H: Ar-H of cationic silane), 7.68 (d, $J_{H,H}$ = 8.2 Hz, 3H of cationic silane), 7.58 – 7.51 (m, 8H of which 1H: Si-H of cationic silane + 4H: C(4)^{Ar}-H of $BAr^F{}_4^-$ + 3H: Ar-H of cationic silane), 7.33 (t, $J_{H,H}$ = 7.5 Hz, 3H, Ar-H of cationic silane), 3.54 (d, $J_{H,P}$ = 16.0 Hz, 3H, P^+-CH_3 of cationic silane), 2.65 (d, $J_{H,P}$ = 2.0 Hz, 9H, Ar-CH_3 of cationic silane). **^{13}C NMR** (101 MHz, Methylene Chloride-d_2) δ 162.3 (q, $J_{C,B}$ = 49.8 Hz, C^{Ar}-^{11}B of $BAr^F{}_4^-$), 143.1 (d, $J_{C,P}$ = 8.7 Hz, indole-C8 of cationic silane), 135.4 (br.s., C(2)^{Ar}-H of $BAr^F{}_4^-$), 130.2 (d, $J_{C,P}$ = 14.7 Hz, indole-C9 or C3 of cationic silane), 129.7 (d, $J_{C,P}$ = 13.8 Hz, indole-C3 or C9 of cationic silane), 129.4 (qq, $J_{C,F}$ = 31.4, 2.9 Hz, <u>C(3)^{Ar}</u>-CF_3 of $BAr^F{}_4^-$), 128.8 (C^{Ar}-H of cationic silane), 123.7 (C^{Ar}-H of cationic silane), 125.2 (q, $J_{C,F}$ = 272.4 Hz, CF_3 of $BAr^F{}_4^-$), 122.0 (d, $J_{C,P}$ = 1.4 Hz, C^{Ar}-H of cationic silane), 118.1 (hept, $J_{C,F}$ = 4.0 Hz, C(4)^{Ar}-H of $BAr^F{}_4^-$), 117.4 (d, $J_{C,P}$ = 111.3 Hz, indole-C2 of cationic silane), 113.1 (d, $J_{C,P}$ = 1.7 Hz, C^{Ar}-H of cationic silane), 10.8 (Ar-CH_3 of cationic silane), 2.3 (d, $J_{C,P}$ = 57.8 Hz, P^+-CH_3 of cationic silane). **^{31}P NMR** (162 MHz, Methylene Chloride-d_2) δ -7.6 (q, $J_{P,H}$ = 16.0 Hz, carbon satellites: $J_{P,C}$ = 111.0, 14.4, 8.4 Hz). **^{29}Si NMR** (79 MHz, Methylene Chloride-d_2) δ -46.4 (dd, $J_{Si,H}$ = 318.4 Hz, $J_{Si,P}$ = 8.0 Hz). **^{11}B NMR** (128 MHz, Methylene Chloride-d_2) δ -6.61, ^{13}C-satellites at -6.62 (d, $J_{B,C}$ = 49.9 Hz). **^{19}F NMR** (376 MHz, Methylene Chloride-d_2) δ -62.83, ^{13}C-satellites at -62.84 (d, $J_{F,C}$ = 30.9 Hz) and -62.96 (d, $J_{F,C}$ = 272.4 Hz).

<u>Appended spectra:</u> NMR in DCM-d_2: ^{1}H (*Figure S42*), ^{13}C{^{1}H} (*Figure S43*), ^{1}H-^{13}C ASAPHMQC (*Figure S44*), ^{31}P{^{1}H} (*Figure S45*), ^{31}P (*Figure S46*), ^{29}Si INEPT (*Figure S47*), ^{1}H-^{29}Si ASAPHMQC (*Figure S48*), ^{11}B{^{1}H} (*Figure S49*), ^{19}F (*Figure S50*).

Procedure B (preparative procedure in THF). Silanide **1** (contains 19.0 wt.% of THF, 0.067 g, 0.12 mmol, 1.1 equiv.) was dissolved in THF (18.0 ml) and cooled to -78 °C. Under vigorous stirring, a freshly prepared solution of $HBAr^F{}_4$·$2Et_2O$ (0.107 g, 0.106 mmol, 1.00 equiv.) in THF (12.0 ml) was added dropwise. The reaction mixture was stirred for 10 min and then allowed to warm to room temperature. After removal of THF under vacuum, the solid residue was extracted with ether, which was removed *in vacuo* giving 0.150 g of amorphous white powder.

According to NMR data, protonation is quantitative. The solid contains an undetermined amount of solvating THF that precludes an accurate determination of the reaction yield. Additionally, NMR data suggest the presence of a small amount (ca. 10%) of a contaminating $BAr^F{}_4^-$ salt, possibly $NaBAr^F{}_4$ or $HBAr^F{}_4$. In order to assign $BAr^F{}_4^-$-derived signals, we used the spectra of $NaBAr^F{}_4$ for a reference (see *Appended spectra*). Compared to the measurements in DCM-d_2 (*see Procedure A*), the NMR spectra of **2^{BARF}** in THF-d_8 undergo a dramatic change. While ^{1}H and ^{13}C signals remain in line with C_3 symmetry, ^{29}Si spectra indicate a pentacoordinate silicon centre with THF molecule as the fifth ligand, which is also in agreement with the DFT-calculated NMR parameters (see *Section S4.2*). The retention of C_3 symmetry in both ^{1}H and ^{13}C spectra, despite breaking of such symmetry upon coordination of THF, hints at a fluxional process, which may be either positional exchange or reversible dissociation. **^{1}H NMR** (400 MHz, THF-d_8) δ 7.90 – 7.82 (m, 8H, C(2)^{Ar}-H of $BAr^F{}_4^-$), 7.77 (d, $J_{H,H}$ = 8.5 Hz, 3H, Ar-H of cationic silane), 7.69 (d, $J_{H,H}$ = 8.1 Hz, 3H, Ar-H of cationic silane), 7.63 (s, 4H, C(4)^{Ar}-H of $BAr^F{}_4^-$), 7.51 (s, 1H, Si-H of cationic silane), 7.42 (t, $J_{H,H}$ = 7.7 Hz, 3H, Ar-H of cationic silane), 7.22

(t, $J_{H,H}$ = 7.5 Hz, 3H, Ar-H of cationic silane), 3.94 (d, $J_{H,P}$ = 16.6 Hz, 3H, P^+-CH_3 of cationic silane), 2.70 (d, $J_{H,P}$ = 2.0 Hz, 9H, Ar-CH_3 of cationic silane). **^{13}C NMR** (101 MHz, THF-*d_8*) δ 162.9 (hept, $J_{C,B}$ = 16.8 Hz, C^{Ar}-^{10}B of $BAr^F{}_4{}^-$), 162.9 (q, $J_{C,B}$ = 49.8 Hz, C^{Ar}-^{11}B of $BAr^F{}_4{}^-$), 144.0 (d, $J_{C,P}$ = 9.3 Hz, indole-C8 of cationic silane), 135.7 (br.s., $C(2)^{Ar}$-H of $BAr^F{}_4{}^-$), 130.22 (d, $J_{C,P}$ = 14.0 Hz, indole-C9 or C3 of cationic silane), 130.15 (qq, $J_{C,F}$ = 31.6, 2.9 Hz, $\underline{C(3)^{Ar}}$-CF_3 of $BAr^F{}_4{}^-$), 127.2 (C^{Ar}-H of cationic silane), 126.7 (d, $J_{C,P}$ = 15.5 Hz, indole-C3 or C9 of cationic silane), 125.6 (q, $J_{C,F}$ = 272.3 Hz, CF_3 of $BAr^F{}_4{}^-$), 122.4 (C^{Ar}-H of cationic silane), 121.7 (d, $J_{C,P}$ = 1.5 Hz, C^{Ar}-H of cationic silane), 120.3 (d, $J_{C,P}$ = 110.5 Hz, indole-C2 of cationic silane), 118.3 (hept, $J_{C,F}$ = 3.6 Hz, $C(4)^{Ar}$-H of $BAr^F{}_4{}^-$), 114.0 (d, $J_{C,P}$ = 1.9 Hz, C^{Ar}-H of cationic silane), 9.6 (Ar-CH_3 of cationic silane), 0.6 (d, $J_{C,P}$ = 55.5 Hz, P^+-CH_3 of cationic silane). **^{31}P NMR** (162 MHz, THF-*d_8*) δ -7.0 (q, $J_{P,H}$ = 16.7 Hz, carbon satellites: $J_{P,C}$ = 110.3, 14.7, 8.5 Hz). **^{29}Si NMR** (79 MHz, THF-*d_8*) δ -91.0 (dd, $J_{Si,H}$ = 368.4 Hz, $J_{Si,P}$ = 6.7 Hz). **^{11}B NMR** (128 MHz, THF-*d_8*) δ -4.61, ^{13}C-satellites at -4.62 (d, $J_{B,C}$ = 49.8 Hz). **^{19}F NMR** (376 MHz, THF-*d_8*) δ -61.55, ^{13}C-satellites at -61.56 (d, $J_{F,C}$ = 31.2 Hz) and -61.68 (d, $J_{F,C}$ = 272.3 Hz). No satisfactory elemental analysis could be obtained due to high reactivity of **2^{BARF}**.

Appended spectra: NMR of $NaBAr^F{}_4$ in THF-*d_8*: ^{1}H (*Figure S51*), ^{13}C{^{1}H} (*Figure S52*), ^{11}B{^{1}H} (*Figure S53*), ^{19}F (*Figure S54*); NMR of **2^{BARF}** in THF-*d_8*: ^{1}H (*Figure S55*), ^{13}C{^{1}H} (*Figure S56*), ^{1}H-^{13}C ASAPHMQC (*Figure S57*), ^{31}P{^{1}H} (*Figure S58*), ^{31}P (*Figure S59*), ^{29}Si{^{1}H} (*Figure S60*), ^{29}Si INEPT (*Figure S61*), ^{11}B{^{1}H} (*Figure S62*), ^{19}F (*Figure S63*).

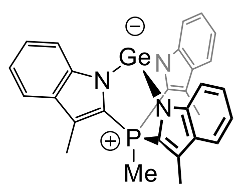

12

TSMPGe (12). Dipotassium salt **3** (contains 21.5 wt.% of THF, 0.385 g, 0.590 mmol, 1.00 equiv.) was suspended in THF (6.0 ml) and cooled down to -78 °C. With vigorous stirring, a solution of $GeCl_2$·dioxane adduct (0.274 g, 1.18 mmol, 2.00 equiv.) in THF (2.0 ml) was added dropwise over a minute. The reaction was stirred at -78 °C over 4h and then allowed to warm up to room temperature overnight. Next morning, the grey-yellow suspension was filtered to yield a bright-yellow solution. The solvent was removed *in vacuo*, the solid was dissolved in DCM, filtered and the solvent was, again, removed *in vacuo* to yield a fine white powder (0.243 g, 81.4%). Crystals suitable for X-ray diffraction analysis were grown by vapor diffusion of *n*-hexane into a solution of **12** in THF at room temperature.

The ^{1}H and ^{13}C NMR spectra of **12** show a single set of signals in aromatic region indicating C_3 symmetry that corresponds to heterobicyclo[2.2.2]octane topology. **^{1}H NMR** (400 MHz, Pyridine-*d_5*) δ 8.15 (d, $J_{H,H}$ = 8.4 Hz, 1H, Ar-H), 7.65 (d, $J_{H,H}$ = 8.0 Hz, 1H, Ar-H), 7.38 (t, $J_{H,H}$ = 7.6 Hz, 1H, Ar-H), 7.20 (t, $J_{H,H}$ = 7.4 Hz, 1H, Ar-H), 3.94 (d, $J_{H,P}$ = 15.8 Hz, 1H, P^+-CH_3), 2.57 (d, $J_{H,P}$ = 1.8 Hz, 3H, Ar-CH_3). **^{13}C NMR** (101 MHz, Pyridine-*d_5*) δ 146.7 (d, $J_{C,P}$ = 12.0 Hz, indole-C8), 130.0 (d, $J_{C,P}$ = 13.8 Hz, indole-C9 or C3), 125.0 (C^{Ar}-H), 121.1 (d, $J_{C,P}$ = 18.7 Hz, indole-C3 or C9), 120.9 (d, $J_{C,P}$ = 1.5 Hz, C^{Ar}-H), 120.6 (d, $J_{C,P}$ = 117.1 Hz, indole-C2), 120.2 (C^{Ar}-H), 114.2 (d, $J_{C,P}$ = 2.2 Hz, C^{Ar}-H), 10.3 (Ar-CH_3), 2.5 (d, $J_{C,P}$ = 56.4 Hz, P^+-CH_3). **^{31}P NMR** (162 MHz, Pyridine-*d_5*) δ -8.5 (q, $J_{P,H}$ = 15.9 Hz, carbon satellites: $J_{P,C}$ = 116.9, 56.6, 18.5, 12.5 Hz). **ESI-TOF-MS** in THF with KCl: found 508.1019 $[M+H]^+$, 582.0355 $[M+H+KCl]^+$ (calcd. 508.1004 $[M+H]^+$, 582.0325 $[M+H+KCl]^+$). No satisfactory elemental analysis could be obtained due to high reactivity of **12**.

Appended spectra: NMR in pyridine-d_5: ^{1}H (*Figure S64*), ^{13}C{^{1}H} (*Figure S65*), ^{1}H-^{13}C ASAPHMQC (*Figure S66*), ^{31}P{^{1}H} (*Figure S67*), ^{31}P (*Figure S68*).

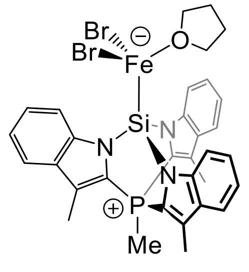

7

(TSMPSi)FeBr$_2$(THF) (7). Crystals suitable for X-ray diffraction analysis were grown in a J. Young NMR tube by layering saturated THF solution of silanide **1** over a saturated THF solution of $FeBr_2$ and PPh_3. The thin plates that form in the course of slow diffusion are insoluble in all conventional solvents, both polar and non-polar, i.e. *n*-hexane, toluene, diethyl ether, DCM, dioxane, THF and acetonitrile. No satisfactory elemental analysis could be obtained due to high reactivity of **7**.

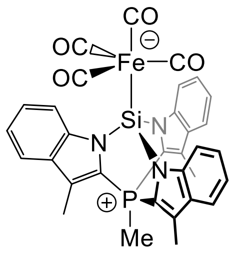

8

(TSMPSi)Fe(CO)$_4$ (8). Solid diiron nonacarbonyl (0.044 g, 0.12 mmol, 1.0 equiv.) and silanide **1** (contains 19.0 wt.% of THF, 0.068 g, 0.12 mmol, 1.0 equiv.) were mixed in a vial equipped with a stirring bar. THF (8.0 ml) was added and the reaction was stirred overnight. As the reaction progressed, the orange color of the mixture shifted into red. Afterwards, the solvent was removed *in vacuo*, the beige powder was washed with DCM (2.0 ml) and THF (1.0 ml). Drying *in vacuo* afforded iron tetracarbonyl silanide complex **8** with 79% yield (0.060 g).

Both ^{1}H and ^{13}C NMR spectra of **8** are consistent with C_3 symmetry that corresponds to heterobicyclo[2.2.2]octane topology. Experimental NMR parameters show a moderate agreement with the DFT-calculated values (see *Section S4.2*). The ^{13}C resonances corresponding to the carbonyls come as two distinct singlets, which indicates slow axial-equatorial exchange at room temperature, analogously to the related phosphine complexes.[10] Solution IR spectrum of **8** in THF shows three strong absorptions at around 2000 cm^{-1} consistent with trigonal bipyramidal geometry of the carbonyl complex.[10,11] **^{1}H NMR** (400 MHz, Pyridine-d_5) δ 9.37 (d, $J_{H,H}$ = 9.0 Hz, 1H, Ar-H), 7.65 – 7.52 (m, 2H+solvent peak, Ar-H), 7.29 (t, $J_{H,H}$ = 7.5 Hz, 1H, Ar-H), 4.21 (d, $J_{H,P}$ = 16.1 Hz, 1H, P^+-CH_3), 2.54 (d, $J_{H,P}$ = 1.9 Hz, 3H, Ar-CH_3). **^{13}C NMR** (101 MHz, Pyridine-d_5) δ 223.2 (Fe-CO, axial), 212.8 (Fe-CO, equatorial), 145.5 (d, $J_{C,P}$ = 10.0 Hz, indole-C8), 130.7 (d, $J_{C,P}$ = 14.2 Hz, indole-C9 or C3), 126.6 (C^{Ar}-H), 125.5 (d, $J_{C,P}$ = 15.9 Hz, indole-C3 or C9), 121.8 (C^{Ar}-H), 120.87 (C^{Ar}-H), 119.3 (d, $J_{C,P}$ = 111.3 Hz, indole-C2), 117.0 (C^{Ar}-H), 9.7 (d, $J_{C,P}$ = 7.7 Hz, Ar-CH_3), 1.6 (d, $J_{C,P}$ = 56.7 Hz, P^+-CH_3). **^{31}P NMR** (162 MHz, Pyridine-d_5) δ -9.5 (q, $J_{P,H}$ = 16.1 Hz, carbon satellites: $J_{P,C}$ = 111.6, 14.8, 9.9 Hz). **^{29}Si NMR** (79 MHz, Pyridine-d_5) δ 34.4 (d, J = 4.6 Hz). **IR** (THF solution) ṽ (cm^{-1}): 1926, 1961, 2038. **ESI-TOF-MS** in acetonitrile: found 628.0607 [*M*-H]$^-$ (calcd. 628.0546 [*M*-H]$^-$). No satisfactory elemental analysis could be obtained due to the high reactivity of **8**.

Appended spectra: NMR in pyridine-d_5: ^{1}H (*Figure S69*), ^{13}C{^{1}H} (*Figure S70*), ^{1}H-^{13}C ASAPHMQC (*Figure S71*), ^{31}P{^{1}H} (*Figure S72*), ^{31}P (*Figure S73*), ^{29}Si (*Figure S74*); IR in THF solution (*Figure S75*).

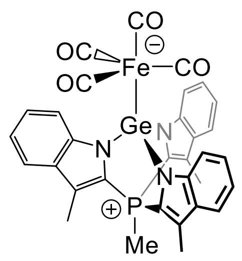

17

(TSMPGe)Fe(CO)$_4$ (17). Solid diiron nonacarbonyl (0.044 g, 0.12 mmol, 1.0 equiv.) and germanide **12** (0.061 g, 0.12 mmol, 1.0 equiv.) were mixed in a vial equipped with a stirring bar. Dry THF (8.0 ml) was added, and the reaction was stirred overnight. As the reaction progressed, the orange color of the mixture became deeper. Afterwards, the solvent was removed *in vacuo*, and the resulting beige powder was washed with DCM (2.0 ml) and THF (1.0 ml). Drying *in vacuo* afforded iron tetracarbonyl germanide complex **17** with 85% yield (0.069 g).

Both ^{1}H and ^{13}C NMR spectra of **17** are consistent with C_3 symmetry that corresponds to heterobicyclo[2.2.2]octane topology. The ^{13}C resonances corresponding to the carbonyls come as a broad signal, which indicates slow axial-equatorial exchange at room temperature, analogously to the related phosphine complexes.[10] Solution IR spectrum of **17** in THF shows three strong absorptions at around 2000 cm^{-1} consistent with trigonal bipyramidal geometry of the carbonyl complex.[10,11] **^{1}H NMR** (400 MHz, Pyridine-*d*$_5$) δ 9.04 (d, $J_{H,H}$ = 8.6 Hz, 1H, Ar-H), 7.63 (d, $J_{H,H}$ = 8.0 Hz, 1H, Ar-H), 7.59 (t, $J_{H,H}$ = 7.8 Hz, 1H, Ar-H), 7.26 (t, $J_{H,H}$ = 7.4 Hz, 1H, Ar-H), 4.12 (d, $J_{H,P}$ = 16.0 Hz, 1H, P^{+}-CH$_3$), 2.56 (d, $J_{H,P}$ = 1.9 Hz, 3H, Ar-CH$_3$). **^{13}C NMR** (101 MHz, Pyridine-*d*$_5$) δ 212.6 (br.s, Fe-(CO)$_4$), 146.9 (d, $J_{C,P}$ = 10.5 Hz, indole-C8), 131.1 (d, $J_{C,P}$ = 14.0 Hz, indole-C9 or C3), 127.2 (C^{Ar}-H), 122.1 (C^{Ar}-H), 122.0 (C^{Ar}-H), 120.0 (d, $J_{C,P}$ = 112.9 Hz, indole-C2), 116.0 (C^{Ar}-H), 10.8 (d, $J_{C,P}$ = 6.0 Hz, Ar-CH$_3$), 3.2 (d, $J_{C,P}$ = 57.2 Hz, P^{+}-CH$_3$). One signal is missing: according to ^{31}P satellites, there should be another doublet with $J_{P,C}$ = 16.3 Hz. It likely overlaps with a solvent peak. **^{31}P NMR** (162 MHz, Pyridine-*d*$_5$) δ -9.1 (q, $J_{P,H}$ = 16.0 Hz, carbon satellites: $J_{P,C}$ = 113.1, 16.3, 14.1, 10.5 Hz). **IR** (THF solution) ṽ (cm^{-1}): 1941, 1970, 2047. **ESI-TOF-MS** in THF: found 692.0002 [*M*+OH]$^{-}$ (calcd. 692.0102 [*M*+OH]$^{-}$). No satisfactory elemental analysis could be obtained due to the high reactivity of **17**.

Appended spectra: NMR in pyridine-*d*$_5$: ^{1}H (*Figure S76*), ^{13}C{^{1}H} (*Figure S77*), ^{1}H-^{13}C ASAPHMQC (*Figure S78*), ^{31}P{^{1}H} (*Figure S79*), ^{31}P (*Figure S80*); IR in THF solution (*Figure S81*).

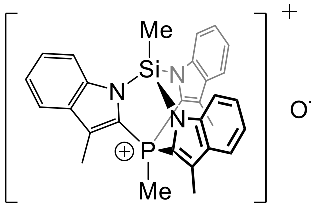

9

[TSMPSiMe]$^{+}$OTf^{-} (9). Silanide **1** (contains 19.0 wt.% of THF, 0.040 g, 0.070 mmol, 1.0 equiv.) was dissolved in DCM (15.0 ml), and the solvent was evaporated *in vacuo*. This operation was repeated two extra times to remove the majority of THF trapped in **1**. The resulting solid was dissolved in DCM (15.0 ml) for the fourth time, and the solution was cooled down to -78 °C. Methyl triflate (8µl, 0.01 g, 0.07 mmol, 1 equiv.) in DCM (0.2 ml) was added dropwise over 0.5 min with vigorous stirring. The reaction was allowed to warm up to room temperature, and the solvent was removed *in vacuo* to give white powder (99%, 0.044 g). Without further purification, the solid was analyzed by NMR, which showed >95% purity.

The ^{1}H and ^{13}C NMR spectra of **9** show a single set of signals in aromatic region indicating C_3 symmetry that corresponds to heterobicyclo[2.2.2]octane topology. Experimental NMR parameters show a good agreement with the DFT-calculated values (see *Section S4.2*). **^{1}H NMR** (400 MHz, Methylene Chloride-*d*$_2$) δ 7.62 – 7.53 (m, 2H, Ar-H), 7.46 (t, $J_{H,H}$ = 7.7 Hz, 1H, Ar-H), 7.25 (t, $J_{H,H}$ =

7.5 Hz, 1H, Ar-H), 3.82 (d, $J_{H,P}$ = 15.8 Hz, 1H, P^+-CH_3), 2.68 (d, $J_{H,P}$ = 2.0 Hz, 3H, Ar-CH_3), 2.47 (s, 1H, Si-CH_3). **^{13}C NMR** (101 MHz, Methylene Chloride-d_2) δ 143.2 (d, J = 9.0 Hz, indole-C8), 130.0 (d, J = 14.0 Hz, indole-C9 or C3), 129.6 (d, J = 15.0 Hz, indole-C3 or C9), 128.1 (C^{Ar}-H), 122.9 (d, J = 1.3 Hz, C^{Ar}-H), 121.9 (d, J = 1.3 Hz, C^{Ar}-H), 118.0 (d, J = 110.1 Hz, indole-C2), 112.9 (d, J = 1.7 Hz, C^{Ar}-H), 10.5 (d, J = 1.0 Hz, Ar-CH_3), 1.9 (d, J = 56.1 Hz, P^+-CH_3), -3.5 (Si-CH_3). **^{31}P NMR** (162 MHz, Methylene Chloride-d_2) δ -8.25 (q, $J_{P,H}$ = 16.0 Hz, carbon satellites: $J_{P,C}$ = 110.1, 55.7, 14.3, 8.4 Hz). **^{29}Si NMR** (79 MHz, Methylene Chloride-d_2) δ -22.5 (p, $J_{Si,H}$ = $J_{Si,P}$ =8.1 Hz). **^{19}F NMR** (376 MHz, Methylene Chloride-d_2) δ -78.8. No satisfactory elemental analysis could be obtained due to high reactivity of **9**.

Appended spectra: NMR in DCM-d_2: ^{1}H (*Figure S82*), ^{13}C{^{1}H} (*Figure S83*), ^{1}H-^{13}C ASAPHMQC (*Figure S84*), ^{31}P{^{1}H} (*Figure S85*), ^{31}P (*Figure S86*); ^{29}Si{^{1}H} (*Figure S87*); ^{29}Si (*Figure S88*); ^{29}Si INEPT (*Figure S89*); ^{19}F (*Figure S90*).

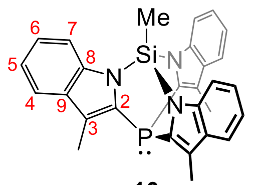

10

iso-TSMPSi (10). Silanide **1** (contains 19.0 wt.% of THF, 0.020 g, 0.035 mmol, 1.0 equiv.) was dissolved in benzene (16.0 ml), and a suspension of KC_8 (1.2 mg, 0.0088 mmol, 0.25 equiv.) in benzene (5.0 ml) was added with vigorous stirring at room temperature. After 5 min of stirring, the bronze coloration of KC_8 disappeared and yellow-grey suspension was stirred for 16h. Afterwards, the reaction was filtered, and the filtrate was freed from solvent *in vacuo* to yield an amorphous yellow solid in a quantitative yield with respect to **1** (99%, 0.016 g). Crystals suitable for X-ray diffraction analysis were grown by vapor diffusion of *n*-hexane into a solution of **10** in pyridine at room temperature.

The ^{1}H and ^{13}C NMR spectra of **10** show a single set of signals in aromatic region indicating C_3 symmetry that corresponds to heterobicyclo[2.2.2]octane topology. Experimental NMR parameters show a good agreement with the DFT-calculated values (see *Section S4.2*). **^{1}H NMR** (400 MHz, THF-d_8) δ 7.56 (d, $J_{H,H}$ = 8.3 Hz, 1H, H^7), 7.45 (d, $J_{H,H}$ = 7.8 Hz, 1H, H^4), 7.18 (ddd, $J_{H,H}$ = 8.3, 7.0, 1.3 Hz, 1H, H^6), 7.03 (t, $J_{H,H}$ = 7.4 Hz, 1H, H^5), 2.51 (d, $J_{H,P}$ = 1.1 Hz, 3H, Ar-CH_3), 2.34 (s, 1H, Si-CH_3). ^{1}H NMR (400 MHz, Benzene-d_6) δ 7.43 (dd, $J_{H,H}$ = 6.7, 1.2 Hz, 1H, Ar-H), 7.23 – 7.15 (m, overlaps with a solvent peak, Ar-H), 7.14 – 7.10 (m, 1H, Ar-H), 2.46 (d, $J_{H,P}$ = 1.1 Hz, 3H, Ar-CH_3), 1.22 (s, 1H, Si-CH_3). **^{13}C NMR** (101 MHz, THF-d_8) δ 142.6 (indole-C8), 133.6 (d, $J_{C,P}$ = 9.9 Hz, indole-C3 or C9), 131.5 (d, $J_{C,P}$ = 7.5 Hz, indole-C9 or C3), 124.6 (indole-C6), 124.2 (d, $J_{C,P}$ = 29.8 Hz, indole-C2), 121.0 (indole-C5), 120.5 (d, $J_{C,P}$ = 1.9 Hz, indole-C4), 113.0 (indole-C7), 10.1 (d, $J_{C,P}$ = 10.4 Hz, Ar-CH_3), -4.3 (Si-CH_3). ^{13}C NMR (101 MHz, Benzene-d_6) δ 142.0 (indole-C8), 133.1 (d, $J_{C,P}$ = 10.2 Hz, indole-C9 or C3), 131.3 (d, $J_{C,P}$ = 7.3 Hz, indole-C3 or C9), 124.3 (d, $J_{C,P}$ = 29.9 Hz, indole-C2), 124.2 (C^{Ar}-H), 120.8 (C^{Ar}-H), 120.7 (d, $J_{C,P}$ = 1.9 Hz, C^{Ar}-H), 112.4 (C^{Ar}-H), 10.2 (d, $J_{C,P}$ = 10.0 Hz, Ar-CH_3), -4.8 (Si-CH_3). **^{31}P NMR** (162 MHz, THF-d_8) δ -124.8. ^{31}P NMR (162 MHz, Benzene-d_6) δ -124.1. **^{29}Si NMR** (79 MHz, THF-d_8) δ -25.7 (qd, $J_{Si,H}$ = 8.4 Hz; $J_{Si,P}$ = 2.9 Hz). ^{29}Si NMR (79 MHz, Benzene-d_6) δ -26.8 (qd, $J_{Si,H}$ = 8.2 Hz; $J_{Si,P}$ = 2.7 Hz). No satisfactory elemental analysis could be obtained due to high reactivity of **10**.

Appended spectra: NMR in THF-d_8: ^{1}H (*Figure S91*), ^{1}H DQF-COSY (*Figure S92*), ^{1}H NOE (*Figure S93*, *Figure S94*), ^{13}C{^{1}H} (*Figure S95*), ^{1}H-^{13}C ASAPHMQC (*Figure S96*), ^{31}P (*Figure S97*), ^{29}Si{^{1}H} (*Figure S98*), ^{29}Si INEPT (*Figure S99*), ^{1}H-^{29}Si gHMBC (*Figure S100*); NMR in benzene-d_6: ^{1}H (*Figure S101*), ^{13}C{^{1}H} (*Figure S102*), ^{1}H-^{13}C ASAPHMQC (*Figure S103*), ^{31}P (*Figure S104*),^{29}Si INEPT (*Figure S105*).

S2.2 Fluorenes

Fluoradene (4). Compound **4** was synthesized following the synthetic route in *Scheme S1*. Intermediates **4a** and **4b** as well as the final product **4** were prepared using reported procedures.[12–14]

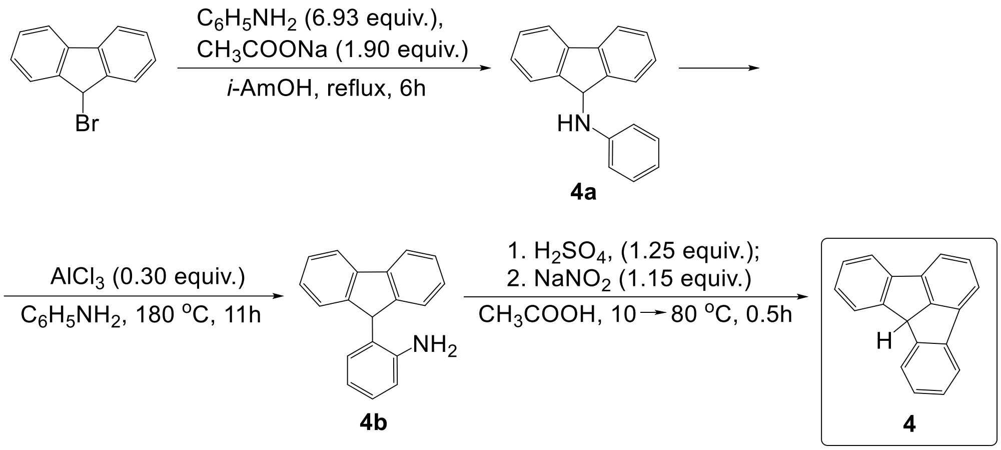

Scheme S1. Synthesis of fluoradene (**4**).

To the best of our knowledge, there are no published characterization data on **4** beyond elemental analysis and electronic spectra.[14] Below, we provide NMR and ESI-MS data that are fully consistent with the structure of **4**. **^{1}H NMR** (400 MHz, Chloroform-*d*) δ 7.85 (dd, $J_{H,H}$ = 7.4, 0.9 Hz, 2H, Ar-H), 7.75 (d, $J_{H,H}$ = 7.6 Hz, 2H, Ar-H), 7.52 – 7.43 (m, 3H, Ar-H), 7.40 ('t', $J_{H,H}$ = 7.5 Hz, 2H, Ar-H), 7.31 (dd, $J_{H,H}$ = 7.5, 1.3 Hz, 2H, Ar-H), 5.21 (s, 1H, C(sp^3)-H). ^{1}H NMR (400 MHz, THF-d_8) δ 7.89 ('d', $J_{H,H}$ = 7.5 Hz, 1H, Ar-H), 7.74 ('d', $J_{H,H}$ = 7.6 Hz, 1H, Ar-H), 7.48 ('d', $J_{H,H}$ = 7.4 Hz, 1H, Ar-H), 7.39 (ddd, $J_{H,H}$ = 8.0, 6.6, 0.9 Hz, 1H, Ar-H), 7.34 ('t', $J_{H,H}$ = 7.6 Hz, 1H, Ar-H), 7.26 (ddd, $J_{H,H}$ = 7.5, 1.2 Hz, 1H, Ar-H), 5.21 (s, 1H, C(sp^3)-H). ^{1}H 1D zTOCSY spectra show two aromatic spin systems in 8:3 ratio. **^{13}C NMR** (101 MHz, Chloroform-*d*) δ 161.3, 147.4, 144.91, 139.9, 130.2, 127.5, 126.4, 125.5, 122.0, 119.1, 55.3 (C(sp^3)-H). ^{13}C NMR (101 MHz, THF-d_8) δ 162.4, 148.5, 145.9, 140.9, 131.0, 128.3, 127.2, 126.4, 122.70, 119.8, 56.3 (C(sp^3)-H). ^{13}C and ^{1}H-^{13}C ASAPHMQC spectra show four quaternary carbon atoms. **ESI-TOF-MS** in THF with NEt_3: found 239.0848 [*M*-H]$^-$ (calcd. 239.0861 [*M*-H]$^-$).

Appended spectra: NMR in chloroform-*d*: ^{1}H (*Figure S106*), ^{13}C{^{1}H} (*Figure S107*), 1D zTOCSY (*Figure S108*), ^{1}H-^{13}C ASAPHMQC (*Figure S109*); NMR in THF-d_8: ^{1}H (*Figure S110*), ^{13}C{^{1}H} (*Figure S111*).

1,3-di(9*H*-fluoren-9-ylidene)propane (5). Compound **5** was prepared following the synthetic route outlined in *Scheme S2*. Only intermediates **5a**, **5c** and well as the final product **5** were isolated and characterized. Intermediates **5b** and **5d** were used without purification.

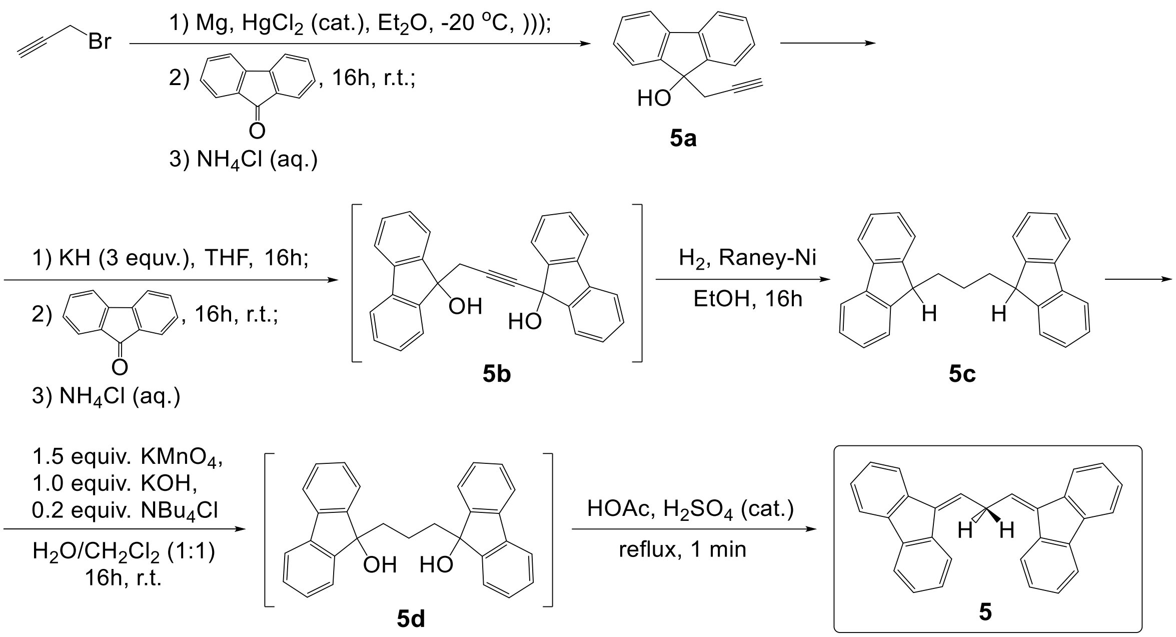

Scheme S2. Synthesis of 1,3-di(9*H*-fluoren-9-ylidene)propane (**5**).

*9-(2-Propynyl)-9H-fluoren-9-ol (**5a**).* An oven-dried Schlenk tube was charged with $HgCl_2$ (0.014 g, 0.052 mmol, $2.9 \cdot 10^{-4}$ equiv.), magnesium turnings (3.37 g, 139 mmol, 5.00 equiv.) and dry ether (30.0 ml) under nitrogen. The mixture was sonicated for 5 min., and a solution of propargyl bromide in toluene (80 wt.% solution, 6.0 ml, 55 mmol, 2.0 equiv.) was added in two parts. The first part (0.20 ml) was added in one portion, upon which the reaction started warming up as was indicated by gentle boiling of ether. The rest of the propargyl bromide solution was added dropwise at -20 °C over an hour. After stirring at -20 °C for another hour, a solution of fluorenone (5.00 g, 27.8 mmol, 1.00 equiv.) in THF (40.0 ml) was added dropwise at -10 °C over an hour. The reaction was allowed to warm up to room temperature, stirred for another hour, after which it was quenched with saturated aqueous solution of NH_4Cl (~100 ml) until all magnesium dissolved. The organic layer was separated, and the aqueous layer was extracted with ether (3 x 50.0 ml). The combined organic layers were dried over anhydrous Na_2SO_4. The solvents were removed *in vacuo* to yield a light-yellow oil that, upon trituration with hexane, crystallized into an off-white powder. The yield is 85.1% (5.20 g) with respect to fluorenone, >95% purity according to NMR spectroscopy.

^{1}**H NMR** (400 MHz, Chloroform-*d*) δ 7.68 (d, $J_{H,H}$ = 7.3 Hz, 2H, Ar-H), 7.62 (d, $J_{H,H}$ = 7.4 Hz, 2H, Ar-H), 7.39 (ddd, $J_{H,H}$ = 7.5, 7.5, 1.2 Hz, 2H, Ar-H), 7.32 (ddd, $J_{H,H}$ = 7.4, 7.4, 1.1 Hz, 2H, Ar-H), 2.88 (d, $J_{H,H}$ = 2.6 Hz, 2H, CH_2), 2.53 (s, 1H, OH), 2.05 (t, J = 2.6 Hz, 1H, C(sp)-H). ^{13}**C NMR** (101 MHz, Chloroform-*d*) δ 147.6, 139.4, 129.5, 128.1, 123.8, 120.1, 80.0 (C(sp)), 79.7 (C(sp)), 71.1 (qC(sp^3)), 30.6 (CH_2). **ESI-TOF-MS** in THF with HCOOH: found 203.0801 [*M*-OH]$^+$ (calcd. 203.0861 [*M*-OH]$^+$).

Appended spectra: NMR in chloroform-*d*: ^{1}H (*Figure S112*), ^{1}H gCOSY (*Figure S113*), ^{13}C{^{1}H} (*Figure S114*), ^{1}H-^{13}C ASAPHMQC (*Figure S115*).

1,3-di(9H-fluoren-9-yl)propane (5c). An oven-dried Schlenk tube under nitrogen was charged with 9-(2-propynyl)-9*H*-fluoren-9-ol (**5a**, 5.20 g, 23.6 mmol, 1.00 equiv.) in THF (50.0 ml). Slowly and under stirring, solid KH (2.84 g, 70.8 mmol, 3.00 equiv.) was added. The reaction was left to stir overnight to yield a dark-red solution. Solid fluorenone (4.25 g, 23.6 mmol, 1.00 equiv.) was added to the mixture, and stirring continued for another night. The resulting brown reaction mixture was then carefully poured into brine (~50 ml) followed by extraction with toluene (3 x 50 ml). The combined organic layers were dried over anhydrous Na_2SO_4, and the solvents were removed *in vacuo* to yield a yellow oil (crude **5b**). Without any further purification, the oil was added to a suspension of Raney nickel (27.7 g) in ethanol (~300 ml) and stirred under H_2 (1 atm.) gas overnight. In the morning, an abundant white flaky precipitate had appeared. The suspension was filtered through a celite pad; the solid residue was extracted with DCM until white flakes had completely dissolved. *(Caution: Raney nickel must be never left dry, otherwise it self-ignites in air!)* After filtration, the solid residue was immediately suspended in water, and Raney nickel was dissolved by careful addition of concentrated sulfuric acid and disposed of. The combined organic filtrate and washings were dried *in vacuo*, which yielded a colourless oil. Upon recrystallization from hexane, white needles were obtained with 71.2% yield (3.13 g) and >95% purity according to NMR spectroscopy.

^{1}H NMR (400 MHz, Chloroform-*d*) δ 7.80 (d, $J_{H,H}$ = 7.5 Hz, 4H, Ar-H), 7.47 – 7.37 (m, 8H, Ar-H), 7.32 (td, $J_{H,H}$ = 7.4, 7.4, 1.2 Hz, 4H, Ar-H), 3.95 (t, $J_{H,H}$ = 6.1 Hz, 2H, C(sp^{3})-H), 2.09 – 1.90 (m, 4H, 1,3-CH_2), 1.48 – 1.27 (m, 2H, 2-CH_2). **^{13}C NMR** (101 MHz, Chloroform-*d*) δ 147.6, 141.1, 127.0, 126.9, 124.5, 119.9, 47.3 (C(sp^{3})-H), 33.4 (1,3-CH_2), 22.9 (2-CH_2). **ESI-TOF-MS** in THF with $AgNO_3$: found 479.0768 [*M*+Ag]$^{+}$ (calcd. 479.0929 [*M*+Ag]$^{+}$).

Appended spectra: NMR in chloroform-*d*: ^{1}H (*Figure S116*), ^{1}H gCOSY (*Figure S117*), ^{13}C{^{1}H} (*Figure S118*), ^{1}H-^{13}C ASAPHMQC (*Figure S119*).

1,3-di(9H-fluoren-9-ylidene)propane (5). Oxidation step was adapted from Gannon and Krause,[15] *dehydration was adapted from Kuhn et al.*[16] To 1,3-di(9*H*-fluoren-9-yl)propane (**5c**, 0.40 g, 1.1 mmol, 1.0 equiv.) in DCM (5.0 ml) was added a solution of $KMnO_4$ (0.255 g, 1.61 mmol, 1.50 equiv.), KOH (0.060 g, 1.1 mmol, 1.0 equiv.) and tetrabutylammonium chloride (0.060 g, 0.22 mmol, 0.20 equiv.) in water (5.0 ml). The mixture was stirred for 16h, after which it was acidified with HOAc (0.5 ml), and solid $NaHSO_3$ was added until the brown coloration disappeared. The organic layer was separated, and the mixture was extracted with DCM (3 x 5.0 ml). The combined organic layers were dried over anhydrous Na_2SO_4 followed by removal of DCM on a rotary evaporator to yield a yellow oil (crude **5d**). Without any further purification, the oil was dissolved in boiling HOAc (10.0 ml) and 1 drop of concentrated H_2SO_4 was added. Immediately after, the reaction was cooled down to room temperature and diluted with water, resulting into grey precipitate. The latter was washed with water, dried and recrystallized from toluene/ethanol to furnish fibrous yellowish needles with 69.8% yield (0.275 g) and >95% purity according to NMR spectroscopy.

To the best of our knowledge, there are no published characterization data on **5** beyond elemental analysis and electronic spectra.[16] Below, we provide NMR and ESI-MS data that are fully consistent with the structure of **5**, as well as electronic spectra that are identical to the published ones. **^{1}H NMR**

(400 MHz, Chloroform-*d*) δ 7.91 (d, $J_{H,H}$ = 7.6 Hz, 2H, Ar-H), 7.80 (d, $J_{H,H}$ = 7.5 Hz, 2H, Ar-H), 7.73 (d, $J_{H,H}$ = 7.5 Hz, 2H, Ar-H), 7.69 (d, $J_{H,H}$ = 7.5 Hz, 2H, Ar-H), 7.45 – 7.26 (m, 8H, Ar-H), 6.94 (t, $J_{H,H}$ = 7.3 Hz, 2H, =CH), 4.36 (t, $J_{H,H}$ = 7.3 Hz, 2H, CH_2). ^{1}H NMR (400 MHz, THF-d_8) δ 8.01 (d, *J* = 7.6 Hz, 2H, Ar-H), 7.82 (d, *J* = 7.3 Hz, 2H, Ar-H), 7.75 (d, *J* = 7.7 Hz, 4H, Ar-H), 7.45 – 7.20 (m, 8H, Ar-H), 7.05 (t, *J* = 7.3 Hz, 2H, =CH), 4.42 (t, *J* = 7.3 Hz, 2H, CH_2). ^{1}H NMR (400 MHz, 1,4-Dioxane-h_8) δ 8.00 (d, $J_{H,H}$ = 7.5 Hz, 2H, Ar-H), 7.83 (d, $J_{H,H}$ = 7.1 Hz, 2H, Ar-H), 7.80 – 7.72 (m, 4H, Ar-H), 7.48 – 7.20 (m, 8H, Ar-H), 7.03 (t, $J_{H,H}$ = 7.3 Hz, 2H, =CH), 4.41 (t, $J_{H,H}$ = 7.3 Hz, 2H, CH_2). ^{1}H 1D zTOCSY spectra in chloroform-*d* show two aromatic and one mixed spin system in a ratio of 4:4:2. **^{13}C NMR** (101 MHz, Chloroform-*d*) δ 141.3, 139.3, 139.0, 137.3, 137.2, 128.4, 128.0, 127.4, 127.2, 126.9 (=CH), 125.3, 120.2, 120.1, 119.7, 29.59 (CH_2). ^{13}C NMR (101 MHz, THF-d_8) δ 142.2, 140.3, 139.9, 138.1, 138.0, 129.0, 128.6, 128.0, 128.0, 127.8, 126.1, 120.9, 120.7, 120.3, 30.30 (CH_2). ^{13}C and ^{1}H-^{13}C ASAPHMQC spectra in chloroform-*d* shows 5 quaternary carbon signals. **UV-Vis** (diethyl ether): λ_{max} (ε)=316 (3.6·10^4), 303 (2.6·10^4), 286 (2.8·10^4), 257 (8.1·10^4), 248 nm (6.4·10^4 cm^{-1}M^{-1}). **ESI-TOF-MS** in THF with KCl: found 407.2058 [*M*+K]$^+$ (calcd. 407.1202 [*M*+K]$^+$).

Appended spectra: NMR in chloroform-*d*: ^{1}H (*Figure S120*), ^{1}H 1D zTOCSY (*Figure S121*, *Figure S122*, *Figure S123*), ^{13}C{^{1}H} (*Figure S124*), ^{1}H-^{13}C ASAPHMQC (*Figure S125*); NMR in THF-d_8: ^{1}H (*Figure S126*), ^{13}C{^{1}H} (*Figure S127*); NMR in dioxane-d_8: ^{1}H (*Figure S128*); UV-Vis in diethyl ether (*Figure S129*).

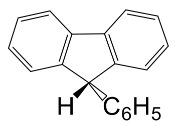

14

9-Phenyl-9*H*-fluorene (14). Compound **14** was synthesized following a literature procedure.[17] ^{1}H and ^{13}C NMR spectra in chloroform-*d* match the reported ones. As a reference for THF ring-opening studies, below we provide the list of ^{1}H NMR signals of **14** in THF-d_8.

^{1}H NMR (400 MHz, THF-d_8) δ 7.82 (d, *J* = 7.6 Hz, 2H, Ar-H), 7.34 (t, *J* = 7.4 Hz, 2H, Ar-H), 7.30 – 7.14 (m, 7H, Ar-H), 7.06 (d, *J* = 7.1 Hz, 2H, Ar-H), 5.08 (s, 1H, C(sp^3)-H).

Appended spectra: NMR in THF-d_8: ^{1}H (*Figure S130*).

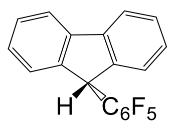

15

9-Pentafluorophenyl-9*H*-fluorene (15). Compound **15** was synthesized following a literature procedure.[18] ^{1}H and ^{13}C NMR spectra in chloroform-*d* match the reported ones. As a reference for THF ring-opening studies, below we provide the list of ^{1}H NMR signals of **15** in THF-d_8.

^{1}H NMR (400 MHz, THF-d_8) δ 7.87 (d, *J* = 7.7 Hz, 2H, Ar-H), 7.41 (t, *J* = 7.5 Hz, 2H, Ar-H), 7.33 (d, *J* = 7.5 Hz, 2H, Ar-H), 7.27 (t, *J* = 7.4 Hz, 2H, Ar-H), 5.53 (s, 1H, C(sp^3)-H).

Appended spectra: NMR in THF-d_8: ^{1}H (*Figure S131*).

9-Phenylsulfonyl-9*H*-fluorene (16). Compound **16** was synthesized following the synthetic route shown in *Scheme S3*. Intermediate **16a** as well as the final product **16** were prepared following reported procedures.[19,20]

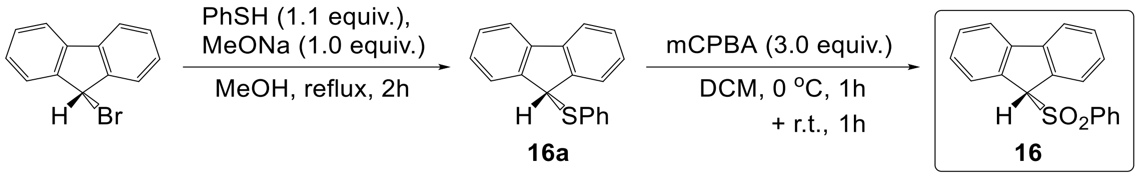

Scheme S3. Synthesis of 9-phenylsulfonyl-9*H*-fluorene (**16**).

^{1}H and ^{13}C NMR spectra in chloroform-*d* match the reported ones.[20] As a reference for THF ring-opening studies, below we provide the list of ^{1}H NMR signals of **16** in THF-d_8.

^{1}H NMR (400 MHz, THF-d_8) δ 8.01 – 7.91 (m, 2H, Ar-H), 7.55 – 7.47 (m, 2H, Ar-H), 7.40 – 7.32 (m, 4H, Ar-H), 7.32 – 7.25 (m, 1H, Ar-H), 7.11 – 6.99 (m, 4H, Ar-H), 5.63 (s, 1H, C(sp^3)-H).

Appended spectra: NMR in THF-d_8: ^{1}H (*Figure S132*).

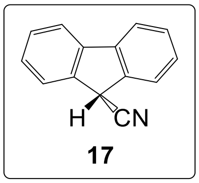

9-Cyano-9*H*-fluorene (17). Compound **17** was purchased commercially from Enamine Ltd. As a reference for THF ring-opening studies, below we provide the list of ^{1}H NMR signals of **17** in THF-d_8.

^{1}H NMR (400 MHz, THF-d_8) δ 7.86 (d, J = 7.5 Hz, 2H, Ar-H), 7.72 (d, J = 7.5 Hz, 2H, Ar-H), 7.47 (t, J = 7.4 Hz, 2H, Ar-H), 7.40 (t, J = 7.4 Hz, 2H, Ar-H), 5.23 (s, 1H, C(sp^3)-H).

Appended spectra: NMR in THF-d_8: ^{1}H (*Figure S133*).

S2.3 Additional experiments

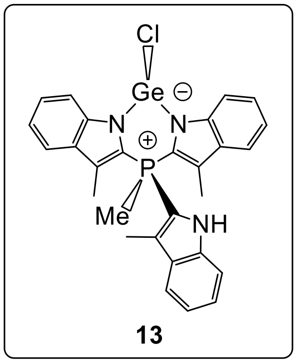

Reaction of TSMPSi (1) and TSMPGe (12) with HCl. Silanide **1** or germanide **12** (0.052 mmol, 1.0 equiv.) was dissolved in THF (10.0 ml), and the solution was cooled down to -78 °C. Ethereal HCl was added dropwise (0.10N, 0.52 ml, 0.052 mmol, 1.0 equiv.). The reaction was left to warm to room temperature overnight. In the morning, the clear colourless solution was divided in two parts. The first part was placed into a vapor diffusion chamber *vs. n*-hexane. For silanide **1**, this yielded a light-beige amorphous powder. For germanide **12**, this yielded colourless blocky crystals identified as **13** by X-ray diffraction. The other part of the reaction mixture was freed of solvent *in vacuo* in order to be redissolved in THF-d_8 for NMR analysis. However, for both **1** and **12**, removal of solvent yielded powders that are completely insoluble in all conventional solvents (benzene, DCM, THF, acetonitrile, pyridine). No satisfactory elemental analysis could be obtained due to the high reactivity of **13**.

pK_a bracketing experiments in dioxane-d_8. As discussed in the main text, in order to make sure that protonation experiments reflected a thermodynamic equilibrium, they were performed in two configurations. *Procedure A* employed zwitterion **1** as a base and **4**, **5** or **6** as an acid. *Procedure B* used the cationic silane $\mathbf{2}^{\mathbf{BARF}}$ (conjugate acid to **1**) as well as a conjugate base to **4**, **5** or **6**. The zwitterionic silanide **1** used for the experiments contained THF, which stood a risk of undergoing cationic polymerization in the presence of acids, thus, complicating the experiments. In order to exclude THF, **1** (contains 19.0 wt.% of THF, 0.010 g, 0.017 mmol, 1.0 equiv.) was twice dissolved in dioxane-h_8 (3.0 ml) followed by evaporation *in vacuo*. This amount of **1** was used for both *Procedures A and B*, and is referred to as "pre-treated zwitterionic silanide **1**" below.

Procedure A. All manipulations were performed in a J. Young NMR tube. Florene **4** or **5** (0.017 mmol, 1.0 equiv.) was dissolved in ca. 0.3 ml of dioxane-d_8. Alternatively, in order to generate 2,6-lutidinium salt **6** *in situ*, to a suspension of $HBAr^F{}_4{\cdot}2Et_2O$ (0.018 g, 0.017 mmol, 1.0 equiv.) in ca. 0.3 ml of dioxane-d_8 was added 2,6-lutidine (2.0 µl, 0.017 mmol, 1.0 equiv.). This was followed by the addition of a solution of pre-treated zwitterionic silanide **1** (1.0 equiv.) in ca. 0.3 ml of dioxane-d_8. The NMR tube was then sealed, shaken for 2 min., and NMR spectra were measured.

Procedure B. In order to prepare conjugated bases of fluorenes **4** and **5**, the fluorenes (0.017 mmol, 1.0 equiv.) were mixed with solid KHMDS (0.004 g, 0.02 mmol, 1 equiv.) and dissolved in dioxane-h_8 (2.0 ml). The solvent was then evaporated *in vacuo*, and the solid what washed with *n*-hexane (3 x 2.0 ml) followed by additional drying. Cationic silane $\mathbf{2}^{\mathbf{BARF}}$ was generated in a J. Young NMR tube by mixing solutions of pre-treated zwitterionic silanide **1** (1.0 equiv.) and $HBAr^F{}_4{\cdot}2Et_2O$ (0.018 g, 0.017 mmol, 1.0 equiv.) in dioxane-d_8 (ca. 0.3 ml for each). A solution of a previously generated conjugate base to **4** or **5** in dioxane-d_8 (ca. 0.6 ml) was then added. Alternatively, a solution of 2,6-lutidine (2.0 µl, 0.017 mmol, 1.0 equiv.) in dioxane-d_8 (ca. 0.6 ml) was added. The NMR tube was then sealed, shaken for 2 min., and NMR spectra were measured. The spectra were identical to those obtained in *Procedure A*, provided the related starting materials were used.

Attempted complexation of TSMPSi (1) with $Fe(OTf)_2$ and $Fe(acac)_2$. Solid $Fe(OTf)_2$ or $Fe(acac)_2$ (0.02 mmol, 1 equiv.) and silanide **1** (0.009 g, 0.02 mmol, 1 equiv.) were mixed in a vial equipped with a stirring bar. Dry THF (10.0 ml) was added, and the clear solution was stirred overnight. The solvent was evaporated *in vacuo* and ~5 mg of the resulting solid was dissolved in THF-d_8 to be analyzed by NMR spectroscopy. Except for some degree of line-broadening, the spectra were identical to those of free **1**.

Reactivity of TSMPSi (1) with CH-acids in THF. Reaction of **1** with fluoradene (**4**) was conducted in both THF-h_8 and THF-d_8, whereas the reactions with other CH-acids (**5**, **14**-**17**) were only undertaken in THF-d_8. Hence, two procedures.

Procedure A (with fluoradene (4) in THF-h_8). THF-h_8 (3.0 ml) was added to a mixture of solid silanide **1** (contains 19.0 wt.% of THF, 0.010 g, 0.017 mmol, 1.0 equiv.) and fluoradene (**4**; 0.004 g, 0.02 mmol, 1 equiv.). The mixture was stirred for the next 4h, upon which the solvent was evaporated *in vacuo*.

The yellow oily residue was redissolved in THF-d_8 (ca. 0.6 ml) and transferred into a J. Young NMR tube. From that point on, if the sample is kept at room temperature, the spectra do not change within at least the next 72h.

Procedure B (with all CH-acids in THF-d_8). THF-d_8 (ca. 0.6 ml) was added to a mixture of solid silanide **1** (contains 19.0 wt.% of THF, 0.010 g, 0.017 mmol, 1.0 equiv.) and CH-acid **4**, **5**, **14**-**17** (1.0 equiv.). The mixture was stirred until dissolution of all solids and transferred into a J. Young NMR tube. Within the next 72h, the reaction was periodically monitored using NMR spectroscopy. The final ^{1}H and ^{31}P NMR spectra with CH-acids **4**, **5**, **14**-**17** are shown in *Figure S15* and *Figure S16*.

S3 X-ray crystal structure determinations

CCDC 2046873-2046878 contain the supplementary crystallographic data for this paper. These data can be obtained free of charge from The Cambridge Crystallographic Data Centre *via* www.ccdc.cam.ac.uk/data_request/cif.

TSMPSi (1). $C_{28}H_{24}N_3PSi \cdot 0.75C_6H_6$, Fw = 520.14, colourless block, 0.34 × 0.25 × 0.17 mm^3, triclinic, $P\overline{1}$ (no. 2), a = 13.2558(8), b = 13.7172(7), c = 15.3872(9) Å, α = 83.192(2), β = 72.330(3), γ = 88.256(3)°, V = 2647.0(3) Å^3, Z = 4, D_x = 1.305 g/cm^3, μ = 0.18 mm^{-1}. The diffraction experiment was performed on a Bruker Kappa ApexII diffractometer with sealed tube and Triumph monochromator (λ = 0.71073 Å) at a temperature of 100(2) K up to a resolution of $(\sin \theta/\lambda)_{max}$ = 0.65 Å^{-1}. The crystal appeared to be cracked with an angle of 2.42° between the two fragments. Consequently, two orientation matrices were used for the integration with the Eval15 software.[21] A multi-scan absorption correction and scaling was performed with TWINABS[22,23] (correction range 0.60-0.75). A total of 93259 reflections was measured, 12185 reflections were unique (R_{int} = 0.053), 8995 reflections were observed [I>2σ(I)]. The structure was solved with Patterson superposition methods using SHELXT.[24] Structure refinement was performed with SHELXL-2016[25] on F^2 of all reflections based on an HKLF-5 file.[26] Non-hydrogen atoms were refined freely with anisotropic displacement parameters. Hydrogen atoms were introduced in calculated positions and refined with a riding model. 685 Parameters were refined with no restraints. R1/wR2 [I > 2σ(I)]: 0.0492 / 0.1151. R1/wR2 [all refl.]: 0.0756 / 0.1284. S = 1.029. Batch scale factor for the two crystal fragments BASF = 0.5383(17). Residual electron density between -0.36 and 0.66 e/Å^3. Geometry calculations and checking for higher symmetry was performed with the PLATON program.[27]

Discussion: The derived molecular structure is shown in *Figure S1*. The asymmetric unit of TSMPSi (**1**) has two independent molecules, however, despite the approximate molecular C_{3v} symmetry, they are located on general positions without symmetry. The molecules feature "naked" anionic centers with the sum of the surrounding N^Si^N angles close to 270°, i.e. 277.43(13)/277.51(14)°. This indicates a high s-character of the anionic lone pairs. In some of the rings, the P and Si deviate significantly from the aromatic indole plane (*Table S1*).

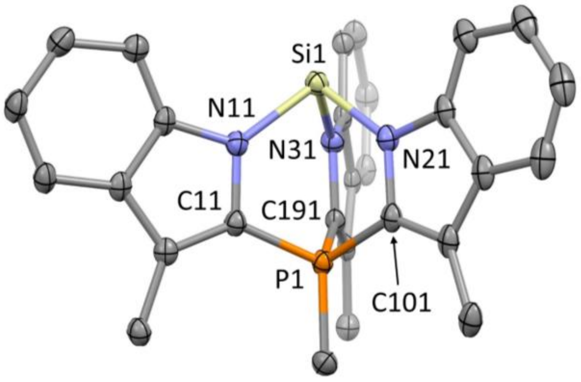

Figure S1. Molecular structure of zwitterionic TSMPSi (**1**) in the crystal. Displacement ellipsoids are drawn at the 50% probability level. Benzene solvent molecules and hydrogen atoms are omitted for clarity. Selected bond distances (Å) and angles (°): two molecules in the asymmetric unit, molecule 1:

Si1-N11 1.8404(18), Si1-N21 1.8581(17), Si1-N31 1.8465(17), N11^Si^N21 92.13(8), N21^Si^N31 93.34(7), N31^Si^N11 91.96(8); C11^P1^C101 104.51(9), C11^P1^C191 105.15(9), C101^P1^C191 101.40(10). molecule 2: N12-Si2 1.8552(18), N22-Si2 1.8441(18), N32-Si2 1.8639(18), N12^Si2^N22 91.87(8), N22^Si2^N32 92.74(8), N32^Si2^N12 92.90(8); C12^P2^C102 104.10(9), C12^P2^C192 104.03(10), C102^P2^C192 103.33(9).

Table S1. Crystallographic dihedral angles between least-squares planes in **1** as calculated with the PLATON software.[27]

**Plane 1**	**Plane 2**	**Angle, °**
N11,C11,C21,C31,C41,C51,C61,C71,C81	P1,Si1,N11,C11	10.84(8)
N21,C101,C111,C121,C131,C141,C151,C161,C171	P1,Si1,N21,C101	15.40(8)
N31,C191,C201,C211,C221,C231,C241,C251,C261	P1,Si1,N31,C191	5.24(7)
N12,C12,C22,C32,C42,C52,C62,C72,C82	P2,Si2,N12,C12	2.35(7)
N22,C102,C112,C122,C132,C142,C152,C162,C172	P2,Si2,N22,C102	0.59(7)
N32,C192,C202,C212,C222,C232,C242,C252,C262	P2,Si2,N32,C192	3.56(7)

$[TSMPSiH]^+BAr^F{}_4^-$ (2^{BARF}). $[C_{28}H_{25}N_3PSi](C_{32}H_{12}BF_{24}) \cdot 2C_4H_8O_2$ + disordered solvent, Fw = 1502.00[a], colourless block, 0.67 × 0.45 × 0.22 mm^3, triclinic, $P\overline{1}$ (no. 2), a = 13.38545(16), b = 15.8323(2), c = 18.8391(2) Å, α = 76.347(1), β = 72.440(1), γ = 70.700(1)°, V = 3551.01(8) Å^3, Z = 2, D_x = 1.405 g/cm^3[a], μ = 0.17 mm^{-1}[a]. The diffraction experiment was performed on a Bruker Kappa ApexII diffractometer with sealed tube and Triumph monochromator (λ = 0.71073 Å) at a temperature of 150(2) K up to a resolution of $(\sin \theta/\lambda)_{max}$ = 0.72 Å^{-1}. The crystal appeared to be cracked into two fragments. Consequently, two orientation matrices were used for the integration with the Eval15 software.[21] A multi-scan absorption correction and scaling was performed with TWINABS[22,23] (correction range 0.67-0.75). A total of 187169 reflections was measured, 21735 reflections were unique (R_{int} = 0.040), 17594 reflections were observed [I>2σ(I)]. The structure was solved with Patterson superposition methods using SHELXT.[24] Structure refinement was performed with SHELXL-2018[25] on F^2 of all reflections based on an HKLF-5 file.[26] The crystal structure contains large voids (265 Å^3/unit cell) filled with severely disordered CH_2Cl_2 solvent molecules. Their contribution to the structure factors was secured by the SQUEEZE algorithm[28] resulting in 84 electrons / unit cell. Non-hydrogen atoms were refined freely with anisotropic displacement parameters. The Si–H hydrogen atom was located in difference Fourier maps and refined freely with an isotropic displacement parameter. C–H hydrogen atoms were introduced in calculated positions and refined with a riding model. Six of the eight CF_3 groups were refined with a disorder model. Slight disorder in the co-crystallized dioxane molecules was not resolved. Minor presence of *n*-hexane on the dioxane locations cannot be ruled out. 1096 Parameters were refined with 3483 restraints (distances, angles and displacement parameters in the CF_3 groups). R1/wR2 [I > 2σ(I)]: 0.0542 / 0.1531. R1/wR2 [all refl.]: 0.0660 / 0.1620. S = 1.050. Fraction of the second crystal fragment BASF = 0.0780(15). Residual electron density between -0.69 and 2.34 e/Å^3 (positive peak in proximity of dioxane).

[a] Derived values do not contain the contribution of the disordered solvent.

Geometry calculations and checking for higher symmetry was performed with the PLATON program.[27]

<u>Discussion:</u> The derived molecular structure is shown in *Figure S2*. The cation of $[TSMPSiH]^+BArF_4^-$ ($\mathbf{2^{BARF}}$) is located on a general position without symmetry. However, there is an approximate, non-crystallographic C_3 symmetry in the cation with an r.m.s. deviation of 0.1231 Å. The silicon-bound hydrogen can be located from the difference Fourier maps. In some of the rings, the P and Si deviate significantly from the aromatic indole plane (*Table S2*).

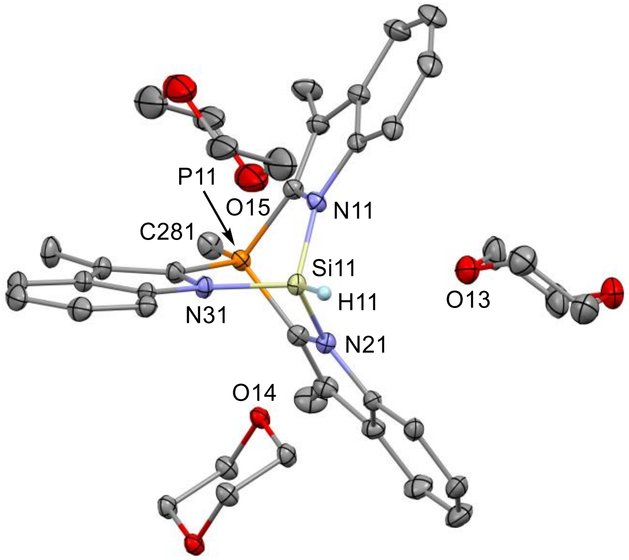

Figure S2. Molecular structure of cationic silane **2** in the crystal of $[TSMPSiH]^+BArF_4^-$ ($\mathbf{2^{BARF}}$). Displacement ellipsoids are drawn at the 30% probability level. The $BAr^F{}_4^-$ counterion, severely disordered CH_2Cl_2, and hydrogen atoms, except for Si–H, are omitted for clarity. The dioxane molecules involving O14 and O15, respectively, are located on inversion centers. Selected bond distances (Å) and angles (°): Si11-H11 1.319(19), Si11-N11 1.7621(13), Si11-N21 1.7565(12), Si11-N31 1.7525(12), Si11-O13 3.2260(16), Si11-O14 2.7807(13), Si11-O15 3.067(3), H11-O13 2.90(2), H11-O14 2.62(2), H11-O15 2.73(2), N11^Si11^N21 99.42(6), N11^Si11^N31 100.33(6), N21^Si11^N31 101.66(6), N11^Si11^H11 115.1(8), N21^Si11^H11 117.1(8), N31^Si11^H11 119.9(8), C11^P11^C101 102.58(7), C101^P11^C191 104.28(7), C11^P11^C191 102.66(7), Si11^H11^O13 92.0(10), Si11^H11^O14 82.7(10), Si11^H11^O15 91.8(10).

Table S2. Crystallographic dihedral angles between least-squares planes in $\mathbf{2^{BARF}}$ as calculated with the PLATON software.[27]

**Plane 1**	**Plane 2**	**Angle, °**
N11,C11,C21,C31,C41,C51,C61,C71,C81	P11,Si11,N11,C11	3.35(6)
N21,C101,C111,C121,C131,C141,C151,C161,C171	P11,Si11,N21,C101	3.00(6)
N31,C191,C201,C211,C221,C231,C241,C251,C261	P11,Si11,N31,C191	5.14(7)

Upon protonation of TSMPSi (**1**) into [TSMPSiH]$^+$ (**2**), the structural changes are mostly confined to the SiN_3 fragment. No other bond in the molecule changes by >0.02 Å, and no flat angle deforms by >2.9°, which is illustrated in *Figure S3* and *Table S3*.

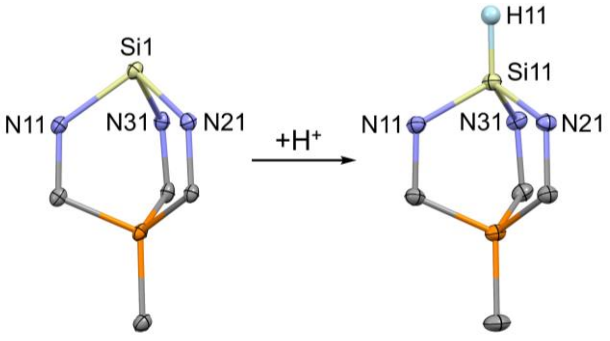

TSMPSi (1) **[TSMPSiH]$^+$ (2)**

Figure S3. Structural changes within the bicyclic cage upon protonation according to the X-ray crystal structure. Only one independent molecular fragment of TSMPSi (**1**) is shown.

Table S3. Structural changes within the bicyclic fragment upon protonation according to the X-ray crystal structure. Since a crystal of TSMPSi (**1**) has two independent molecules in the unit cell, both sets of geometric parameters are given separated by a slash.

**Geometric parameter**	**Value, Å or °**	
	**TSMPSi (1)**	**[TSMPSiH]$^+$BAr$^F_4$$^-$ (2^{BARF})**
Si-N	1.8404(18)-1.8639(18)	1.7525(12)-1.7621(13)
N-C^{Ar}	1.400(3)-1.408(2)	1.4174(18)-1.4195(18)
C^{Ar}-P	1.768(2)-1.7782(19)	1.7751(15)-1.7753(15)
P^+-CH_3	1.780(2)/1.782(2)	1.7756(16)
N^Si^N	91.87(8)-93.34(7)	99.42(6)-101.66(6)
$\Sigma_{N\hat{}Si\hat{}N}$	277.43(13)/277.51(14)	301.41(10)
Si^N^C	125.05(13)-126.36(14)	119.11(10)-119.32(9)
N^C^P	111.00(14)-112.14(14)	113.15(10)-113.40(10)
C^{Ar}^P^C^{Ar}	101.40(10)-105.15(9)	102.58(7)-104.28(7)

TSMPGe (12). $C_{28}H_{24}GeN_3P$ + disordered solvent, Fw = 506.06[a], colourless block, 0.35 × 0.31 × 0.13 mm^3, monoclinic, $P2_1/c$ (no. 14), a = 14.9354(10), b = 8.8055(5), c = 22.7168(15) Å, β = 108.505(4)°, V = 2833.1(3) Å^3, Z = 4, D_x = 1.186 g/cm^3[a], μ = 1.16 mm^{-1}[a]. The diffraction experiment was performed on a Bruker Kappa ApexII diffractometer with sealed tube and Triumph monochromator (λ = 0.71073 Å) at a temperature of 150(2) K up to a resolution of $(\sin \theta/\lambda)_{max}$ = 0.65 Å^{-1}. The Eval15 software[21] was used for the intensity integration. For the prediction of the reflection profiles a large isotropic mosaicity of 1.8° was used. Additional weak crystal fragments and diffuse scattering were ignored during the integration. A multi-scan absorption correction and scaling was performed with

---

[a] Derived values do not contain the contribution of the disordered solvent.

SADABS[22,23] (correction range 0.58-0.75). A total of 58172 reflections was measured, 6527 reflections were unique (R_{int} = 0.045), 5379 reflections were observed [I>2σ(I)]. The structure was solved with Patterson superposition methods using SHELXT.[24] Structure refinement was performed with SHELXL-2016[25] on F^2 of all reflections. The crystal structure contains large voids (654 $Å^3$/unit cell) filled with severely disordered *n*-hexane solvent molecules. Their contribution to the structure factors was secured by the SQUEEZE algorithm[28] resulting in 200 electrons / unit cell. Non-hydrogen atoms were refined freely with anisotropic displacement parameters. Hydrogen atoms were introduced in calculated positions and refined with a riding model. 302 Parameters were refined with no restraints. R1/wR2 [I > 2σ(I)]: 0.0540 / 0.1336. R1/wR2 [all refl.]: 0.0663 / 0.1389. S = 1.190. Residual electron density between -0.41 and 2.90 e/$Å^3$. Geometry calculations and checking for higher symmetry was performed with the PLATON program.[27]

Discussion: The derived molecular structure is shown in *Figure S4*. Despite the approximate molecular C_{3v} symmetry, the molecule is located on a general position without symmetry in the crystal packing. The system features a "naked" anionic center with the sum of the surrounding N^Ge^N angles close to 270°, i.e. 270.04(19)°. This indicates a high s-character of the anionic lone pair. In some of the rings, the P and Si deviate significantly from the aromatic indole plane (*Table S4*).

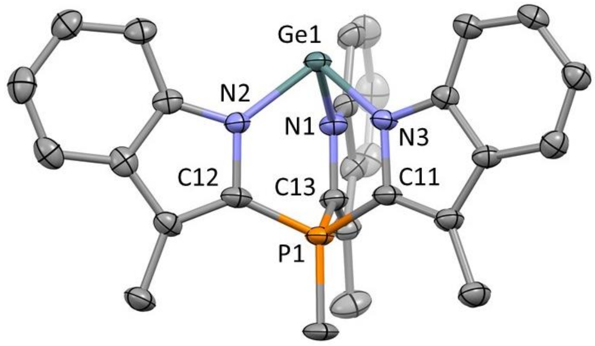

Figure S4. Molecular structure of **12** in the crystal. Displacement ellipsoids are drawn at the 50% probability level. Solvent molecules and hydrogen atoms are omitted for clarity. Selected bond distances (Å) and angles (°): Ge1-N1 1.967(3), Ge1-N2 1.976(3), Ge1-N3 1.972(3), N1^Ge1^N2 89.73(11), N2^Ge1^N3 90.25(11), N3^Ge1^N1 90.06(11); C11^P1^C12 106.65(15), C12^P1^C13 103.52(15), C11^P1^C13 103.69(15).

Table S4. Crystallographic dihedral angles between least-squares planes in **12** as calculated with the PLATON software.[27]

Plane 1	Plane 2	Angle, °
N1,C11,C21,C31,C41,C51,C61,C71,C81	P1,Ge1,N1,C11	12.51(11)
N2,C12,C22,C32,C42,C52,C62,C72,C82	P1,Ge1,N2,C12	11.29(11)
N3,C13,C23,C33,C43,C53,C63,C73,C83	P1,Ge1,N3,C13	0.71(12)

(TSMPSi)FeBr₂(THF) (7). $C_{32}H_{32}Br_2FeN_3OPSi \cdot 3C_4H_8O$, Fw = 965.64, yellow block, 0.46 × 0.20 × 0.10 mm^3, monoclinic, $P2_1/c$ (no. 14), a = 16.5476(8), b = 12.1769(5), c = 21.8288(9) Å, β = 94.147(2)°, V = 4387.0(3) $Å^3$, Z = 4, D_x = 1.462 g/cm^3, μ = 2.27 mm^{-1}. The diffraction experiment

was performed on a Bruker Kappa ApexII diffractometer with sealed tube and Triumph monochromator (λ = 0.71073 Å) at a temperature of 150(2) K up to a resolution of $(\sin \theta/\lambda)_{max}$ = 0.65 Å^{-1}. The Eval15 software[21] was used for the intensity integration. A numerical absorption correction and scaling was performed with SADABS[22,23] (correction range 0.48-0.84). A total of 60843 reflections was measured, 10071 reflections were unique (R_{int} = 0.046), 7928 reflections were observed [$I>2\sigma(I)$]. The structure was solved with Patterson superposition methods using SHELXT.[24] Structure refinement was performed with SHELXL-2016[25] on F^2 of all reflections. Non-hydrogen atoms were refined freely with anisotropic displacement parameters. Hydrogen atoms were introduced in calculated positions and refined with a riding model. One non-coordinated THF solvent molecule was refined with a disorder model. 519 Parameters were refined with 245 restraints (distances, angles and displacement parameters in the non-coordinated THF molecules). R1/wR2 [$I > 2\sigma(I)$]: 0.0372 / 0.0886. R1/wR2 [all refl.]: 0.0548 / 0.0973. S = 1.015. Residual electron density between -0.60 and 1.00 e/Å^3. Geometry calculations and checking for higher symmetry was performed with the PLATON program.[27]

Discussion: The derived molecular structure is shown in *Figure S5*. The Fe(II) center has a tetrahedral configuration typical for an S = 2 spin state. Fe–Br bond lengths of 2.3857(5) and 2.3976(5)Å are also rather typical for a high-spin iron(II) center.[29–36] The Fe–Si bond distance of 2.4711(7) Å is somewhat longer than that in an analogous complex of $FeCl_2$(THF) and C-tethered analogue of **1**, 2.4482(12)/2.4589(12).[37] This indicates a slightly weaker bonding in the case of **7**. As expected, the geometry around silicon is sensitive to coordination. Thus, the sum of crystallographic N^Si^N angles changes from 277.43(13)/277.51(14)° in free TSMPSi (**1**) to 289.42(17)° in complex (TSMPSi)$FeBr_2$(THF) (**7**). This change is consistent with Bent's rule.[38] In some of the rings, the P and Si deviate significantly from the aromatic indole plane (*Table S5*).

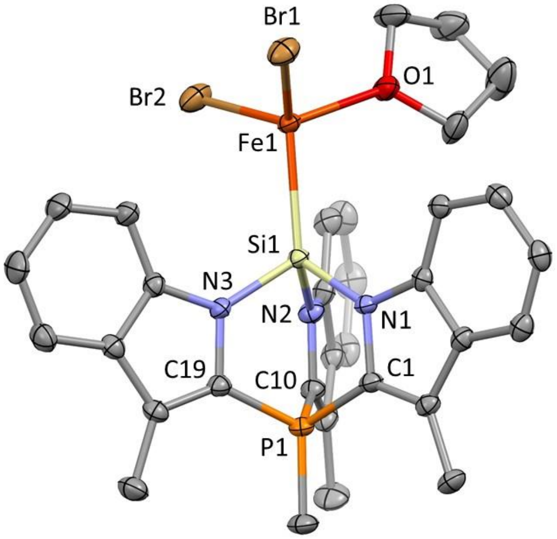

Figure S5. Molecular structure of (TSMPSi)$FeBr_2$(THF) (**7**) in the crystal. Displacement ellipsoids are drawn at the 50% probability level. Hydrogens and non-coordinated THF molecules are omitted for clarity. Selected bond (Å) and angles (°): Si1-N1 1.798(2), Si1-N2 1.812(2), Si1-N3 1.806(2), Fe1-Si1 2.4711(7), Fe1-Br1 2.3976(5), Fe1-Br2 2.3857(5), Fe1-O1 2.0727(19), N1^Si1^N2 96.55(10),

N2^Si1^N3 96.37(10), N3^Si1^N1 96.50(10), C1^P1^C10 102.62(12), C1^P1^C19 104.07(11), C10^P1^C19 103.70(12).

Table S5. Crystallographic dihedral angles between least-squares planes in **7** as calculated with the PLATON software.[27]

Plane 1	Plane 2	Angle, °
N1,C1,C2,C3,C4,C5,C6,C7,C8	P1,Si1,N1,C1	2.93(9)
N2,C10,C11,C12,C13,C14,C15,C16,C17	P1,Si1,N2,C10	2.10(9)
N3,C19,C20,C21,C22,C23,C24,C25,C26	P1,Si1,N3,C19	2.67(9)

iso-TSMPSi (10). $C_{28}H_{24}N_3PSi \cdot C_6H_6$, Fw = 547.73, colourless block, 0.45 × 0.25 × 0.06 mm^3, triclinic, $P\overline{1}$ (no. 2), a = 8.8398(4), b = 12.4251(6), c = 14.7566(7) Å, α = 71.073(3), β = 89.907(2), γ = 71.483(2)°, V = 1444.48(12) $Å^3$, Z = 2, D_x = 1.259 g/cm^3, μ = 0.17 mm^{-1}. The diffraction experiment was performed on a Bruker Kappa ApexII diffractometer with sealed tube and Triumph monochromator (λ = 0.71073 Å) at a temperature of 150(2) K up to a resolution of $(\sin \theta/\lambda)_{max}$ = 0.65 $Å^{-1}$. The Eval15 software[21] was used for the intensity integration. A multi-scan absorption correction and scaling was performed with SADABS[22,23] (correction range 0.69-0.75). A total of 33226 reflections was measured, 6648 reflections were unique (R_{int} = 0.026), 5760 reflections were observed [$I>2\sigma(I)$]. The structure was solved with Patterson superposition methods using SHELXT.[24] Structure refinement was performed with SHELXL-2018[25] on F^2 of all reflections. Non-hydrogen atoms were refined freely with anisotropic displacement parameters. Hydrogen atoms of the Si compound were located in difference Fourier maps. Hydrogen atoms of the hexane were introduced in calculated positions. All hydrogen atoms were refined with a riding model. The *n*-hexane solvent molecule was refined with a disorder model. The solvent disorder could not be fully resolved. 411 Parameters were refined with 144 restraints (distances, angles and displacement parameters in the hexane molecule). R1/wR2 [$I > 2\sigma(I)$]: 0.0389 / 0.1073. R1/wR2 [all refl.]: 0.0451 / 0.1114. S = 1.062. Residual electron density between -0.37 and 0.44 e/$Å^3$. Geometry calculations and checking for higher symmetry was performed with the PLATON program.[27]

Discussion: The derived molecular structure is shown in *Figure S6*. It features a tetrahedral methylated silicon atom and a tricoordinate phosphorus atom. Due to the absence of the methyl group, the phosphorus atom in iso-TSMPSi (**10**) is more pyramidalized as compared to that in the parent zwitterion TSMPSi (**1**) ($\Sigma_{C^\wedge P^\wedge C}$ of 290.67(12)° *vs.* 311.06(16)/311.46(16)°, respectively). The opposite applies to the silicon atom, which now features wider N^Si^N angles ($\Sigma_{N^\wedge Si^\wedge N}$ of 306.97(10)° *vs.* 277.43(13)/277.51(14)° in **1**). All these changes comply with Bent's rule.[38] In some of the rings, the P and Si deviate significantly from the aromatic indole plane (*Table S6*).

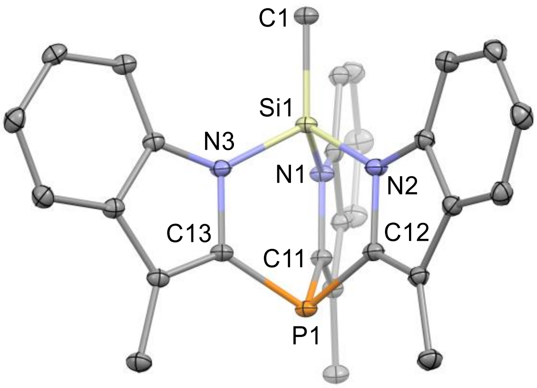

Figure S6. Molecular structure of iso-TSMPSi (**10**) in the crystal. Displacement ellipsoids are drawn at the 30% probability level. Hydrogens and *n*-hexane solvent molecules are omitted for clarity. Selected bond lengths (Å) and angles (°): Si1-N1 1.7473(13), Si1-N2 1.7379(13), Si1-N3 1.7416(13), C1-Si1 1.8284(14), N1^Si1^N2 101.67(6), N2^Si1^N3 101.87(6), N3^Si1^N1 103.43(6), C11^P1^C12 97.91(6), C12^P1^C13 98.57(7), C11^P1^C13 94.19(7).

Table S6. Crystallographic dihedral angles between least-squares planes in **10** as calculated with the PLATON software.[27]

**Plane 1**	**Plane 2**	**Angle, °**
N1,C11,C21,C31,C41,C51,C61,C71,C81	P1,Si1,N1,C11	9.28(5)
N2,C12,C22,C32,C42,C52,C62,C72,C82	P1,Si1,N2,C12	0.97(5)
N3,C13,C23,C33,C43,C53,C63,C73,C83	P1,Si1,N3,C13	11.44(6)

(TSMP–H)GeCl (13). $C_{28}H_{25}ClGeN_3P \cdot C_4H_8O$, Fw = 614.62, colourless block, 0.39 × 0.23 × 0.07 mm^3, monoclinic, $P2_1/n$ (no. 14), a = 9.4061(3), b = 26.6171(13), c = 12.4032(6) Å, β = 111.723(3)°, V = 2884.8(2) Å^3, Z = 4, D_x = 1.415 g/cm^3, μ = 1.24 mm^{-1}. The diffraction experiment was performed on a Bruker Kappa ApexII diffractometer with sealed tube and Triumph monochromator (λ = 0.71073 Å) at a temperature of 150(2) K up to a resolution of $(\sin \theta/\lambda)_{max}$ = 0.65 Å^{-1}. The Eval15 software[21] was used for the intensity integration. A multi-scan absorption correction and scaling was performed with SADABS[22,23] (correction range 0.65-0.75). A total of 66423 reflections was measured, 6640 reflections were unique (R_{int} = 0.025), 6078 reflections were observed [I>2σ(I)]. The structure was solved with Patterson superposition methods using SHELXT.[24] Structure refinement was performed with SHELXL-2016[25] on F^2 of all reflections. Non-hydrogen atoms were refined freely with anisotropic displacement parameters. All hydrogen atoms were located in difference Fourier maps. The N–H hydrogen atoms was refined freely with an isotropic displacement parameter. The C–H hydrogen atoms were refined with a riding model. 360 Parameters were refined with no restraints. R1/wR2 [I > 2σ(I)]: 0.0294 / 0.0769. R1/wR2 [all refl.]: 0.0331 / 0.0786. S = 1.071. Residual electron density between -0.33 and 0.56 e/Å^3. Geometry calculations and checking for higher symmetry was performed with the PLATON program.[27]

<u>Discussion:</u> The derived molecular structure is shown in *Figure S7*. The proton at N1 (H1N) is clearly visible in the difference Fourier map. It can be refined freely in the least-squares refinement of the structure. Additionally, the short N1…O14 distance indicates the presence of a hydrogen bond.

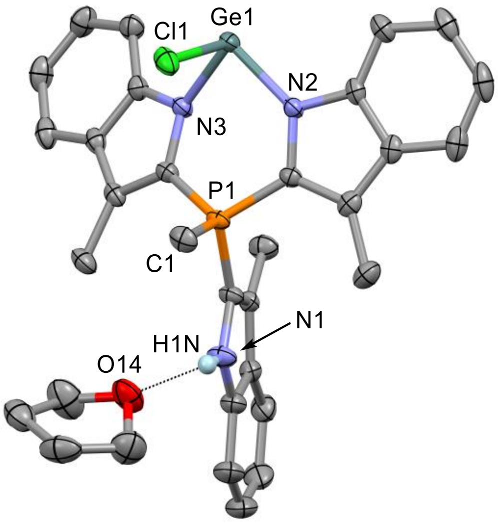

Figure S7. Molecular structure of (TSMP–H)GeCl (**13**) in the crystal. Displacement ellipsoids are drawn at the 50% probability level. Hydrogens, except for H1N, are omitted for clarity. A THF molecule that forms a hydrogen bond with H1N is shown explicitly. Selected bond distances (Å) and angles (°): Ge1-Cl1 2.3549(5), Ge1-N2 1.9524(15), Ge1-N3 1.9569(14), N1-H1N 0.88(3), H1N…O14 2.14(3), N1…O14 2.913(2), Cl1^Ge1^N2 94.01(4), N2^Ge1^N3 90.96(6), N3^Ge1^Cl1 92.36(4), N1^H1N^O14 146(3).

S4 Computational studies

S4.1 General remarks

All calculations were performed using Gaussian 16, Revision A.03.[39] Routine geometry optimizations were carried out in vacuum at the B3LYP-GD3BJ/6-311++G** level of theory. The absence of imaginary frequencies was confirmed for all structures. In case of several possible conformers, the one with the lowest energy was chosen for further calculations. NMR parameters were calculated using GIAO method from stationary geometries at B3LYP-GD3BJ level of theory with IGLO-III basis set for C, H, P, Si, O atoms and 6-311++G** basis set for Fe atoms. Implicit solvation was treated within the Polarizable Continuum Model (PCM).

Gas-phase acidities were derived using both B3LYP-GD3BJ and MP2 with 6-311++G(d,p) basis set. For MP2 calculations, thermal corrections for Gibbs free energy were taken from B3LYP-GD3BJ/6-311++G(d,p) level of theory. It has been shown[40,41] that MP2-derived gas-phase acidities tend to give a better agreement with the experiment than the B3LYP-derived ones for a wide range of acids; nevertheless, both methods give identical trends. This was also true in our calculations. However, due to the higher precision, we chose MP2-calculated values for a further discussion.

Even though deprotonation decomposition schemes are closely related to the gas-phase acidities, given that B3LYP-GD3BJ and MP2 show identical trends, deprotonation decomposition was treated at B3LYP-GD3BJ/6-311++G** level of theory in order to save computational time.

Carbonyl stretching frequencies in IrCp(CO)L model complexes were calculated as described by Gusev.[42] Namely, geometry optimizations and frequency calculations were performed using MPW1PW91 functional with SDD basis set (associated with ECP) for Ir and 6-311+G(d,p) for all other atoms. Tight geometry optimizations and ultrafine integration grid were employed. The absence of imaginary frequencies was confirmed for all structures.

Relaxed potential energy surface (PES) scans were carried out in vacuum at B3LYP-GD3BJ/6-31+G* level of theory. Related stationary points were optimized at the same level of theory. The absence of imaginary frequencies was confirmed for all structures, except for transition states: those had one negative vibrational mode, implying a negative force constant.

NBO analysis was done using NBO 6.0 program.[43] NLMOs were visualized using VMD 1.9.3.[44–46]

Geometric coordinates along with the corresponding energies are provided in *Section S8*.

S4.2 Calculated NMR parameters for isolated compounds

Experimental and calculated ^{29}Si and ^{31}P NMR parameters of selected silicon compounds are shown in *Table S7*. Experimental spin-spin coupling constants *J* are given as absolute values. Regardless of the starting geometry, optimization of THF-coordinated silane **2·THF** always yielded a trigonal bipyramidal silicon center with an axial oxygen atom, which is consistent with high apicophilicity of the latter.

Table S7. Calculated ^{29}Si and ^{31}P NMR parameters of selected silicon compounds. Experimental spin-spin coupling constants *J* are given as absolute values.

Compound	NMR parameters			
	δ, ppm		*J*, Hz	
	**Experimental**	**Calculated**	**Experimental**	**Calculated**
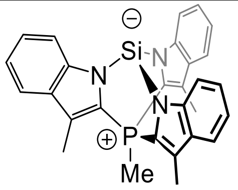<br>**TSMPSi (1)**	THF-d_8:<br>δ(^{31}P)=-7.6<br>δ(^{29}Si)=-48.0<br>Pyridine-d_5:<br>δ(^{31}P)=-7.7<br>δ(^{29}Si)=-47.5	THF-d_8:<br>δ(^{31}P)=-14.8<br>δ(^{29}Si)=-56.2<br>Pyridine-d_5:<br>δ(^{31}P)=-14.7<br>δ(^{29}Si)=-54.2	THF-d_8:<br>$J_{Si,P}$=4.3<br>Pyridine-d_5:<br>$J_{Si,P}$=4.4	THF-d_8:<br>$J_{Si,P}$=7.9<br>Pyridine-d_5:<br>$J_{Si,P}$=7.8
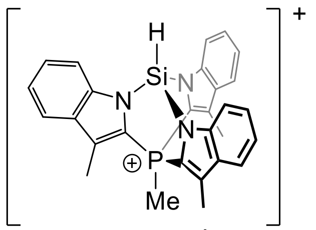<br>**[TSMPSiH]$^+$ (2)**	**2**^{BARF} in<br>DCM-d_2:<br>δ(^{31}P)=-7.6<br>δ(^{29}Si)=-46.4	DCM-d_2:<br>δ(^{31}P)=-10.8<br>δ(^{29}Si)=-59.0	**2**^{BARF} in<br>DCM-d_2:<br>$J_{Si,P}$=8.0<br>$J_{Si,H}$=318.4	DCM-d_2:<br>$J_{Si,P}$=-7.5<br>$J_{Si,H}$=-342.3
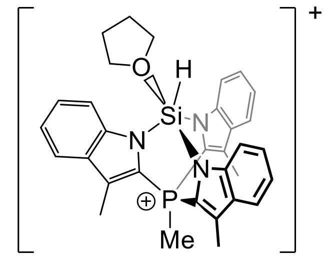<br>**[TSMPSiH]$^+$•THF (2•THF)**	**2**^{BARF} in<br>THF-d_8:<br>δ(^{31}P)=-7.0<br>δ(^{29}Si)=-91.0	THF-d_8:<br>δ(^{31}P)=-13.3<br>δ(^{29}Si)=-104.9	**2**^{BARF} in<br>THF-d_8:<br>$J_{Si,P}$=6.7<br>$J_{Si,H}$=368.4	THF-d_8:<br>$J_{Si,P}$=-7.3<br>$J_{Si,H}$=-392.7
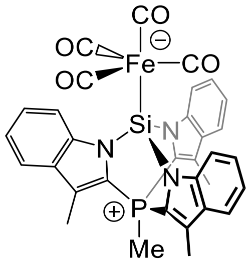<br>**(TSMPSi)Fe(CO)$_4$ (8)**	Pyridine-d_5:<br>δ(^{31}P)=-9.5<br>δ(^{29}Si)=34.4[a]	Pyridine-d_5:<br>δ(^{31}P)=-15.7<br>δ(^{29}Si)=53.2[a]	Pyridine-d_5:<br>$J_{Si,P}$=4.6	Pyridine-d_5:<br>$J_{Si,P}$=-3.7

[a] The discrepancy between calculated and experimental ^{29}Si shifts is likely due to improper treatment of relativistic effects.[103]

Table S7 (continued). Calculated ^{29}Si and ^{31}P NMR parameters of selected silicon compounds. Experimental spin-spin coupling constants J are given as absolute values.

**Compound**	**NMR parameters**			
	**δ, ppm**		***J*, Hz**	
	**Experimental**	**Calculated**	**Experimental**	**Calculated**
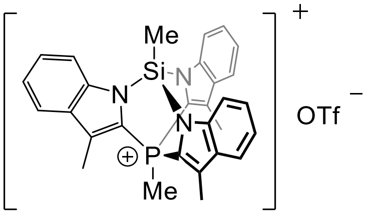<br>**[TSMPSiMe]$^{+}$OTf^{-} (9)**	DCM-d_2:<br>$\delta(^{31}P)$=-8.3<br>$\delta(^{29}Si)$=-22.5	DCM-d_2:<br>$\delta(^{31}P)$=-11.8<br>$\delta(^{29}Si)$=-29.1	DCM-d_2:<br>$J_{Si,P}$=7.9<br>$J_{Si,H}$=7.9	DCM-d_2:<br>$J_{Si,P}$=-7.6<br>$J_{Si,H}$=7.5
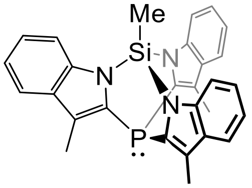<br>**iso-TSMPSi (10)**	THF-d_8:<br>$\delta(^{31}P)$=-124.8<br>$\delta(^{29}Si)$=-25.7	THF-d_8:<br>$\delta(^{31}P)$=-126.6<br>$\delta(^{29}Si)$=-33.7	THF-d_8:<br>$J_{Si,P}$=2.9<br>$J_{Si,H}$=8.4	THF-d_8:<br>$J_{Si,P}$=-1.8<br>$J_{Si,H}$=7.4

S5 Peripheral discussions

S5.1 Synthesis and Brønsted acidity of [TSMPSiH]$^+$ (2)

S5.1.1 The importance of a non-coordinating anion for 2^{BARF}

Initial protonation attempts of TSMPSi (**1**) were performed by reacting with an equimolar amount of ethereal hydrogen chloride in THF, although this gave intractable mixtures of products. In view of generally similar reactivity trends between silicon and germanium compounds, and also higher stability of the latter, we then attempted the protonation of a zwitterionic germanium analogue TSMPGe (**12**; *Scheme S4*). This was anticipated to help us identify the intermediates responsible for degradation in case of TSMPSi (**1**). Germanide TSMPGe (**12**) was prepared using $TSMPK_2$ (**3**) and following a procedure for the synthesis of an analogous C-tethered compound.[11]

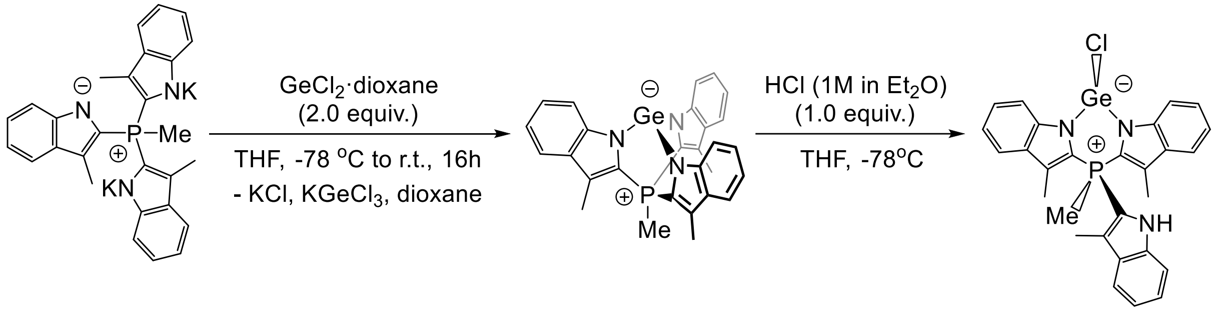

$TSMPK_2$ (3) **TSMPGe (12)** **(TSMP-H)GeCl (13)**

Scheme S4. Synthesis and attempted protonation of the zwitterionic germanide TSMPGe (**12**).

Interestingly, the reaction yielded a zwitterionic germanide (TSMP–H)GeCl (**13**), which can be formally considered a product of hydrogen chloride addition to a Ge–N bond in **12**. The structure of insoluble **13** was elucidated by X-ray crystallography (see *Section S3*) and has a chloride and a methyl group in a *syn* configuration with respect to a six-membered heterocyclic ring.

Taking into account the stereochemistry of **13**, we speculate that its origin goes back to the cationic germane (**A** in *Scheme S5*) that is analogous to the desired silane, [TSMPSiH]$^+$ (**2**). Namely, **13** can be viewed as an isomer of germane **C**, which may form due to the opening of the trigonal bipyramidal intermediate **B**, where the apicophilic chloride occupies an axial position, thus labilizing the bond between germanium and a leaving axial indolide. In turn, intermediate **B** may form as a result of coordination of a chloride anion to germane **A**, the driving force of the coordination being strain-release Lewis acidity.

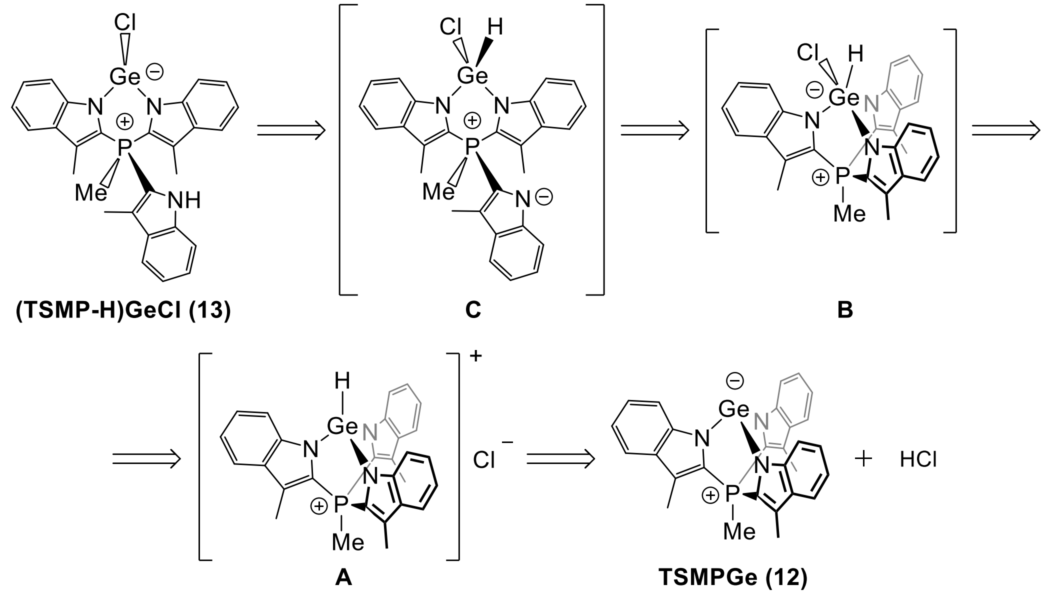

Scheme S5. Retrosynthetic analysis of the proposed path towards **13**.

Regarding the origin of the strain-release Lewis acidity of **A**, our reasoning is as follows. By examining the solid-state structures of TSMPSi (**1**) (see *Section S3*) as well as κ^3 complexes of Fe(II), Ni(II), Cu(I) with the TMSP scaffold,[5] it becomes apparent that the latter prefers N^E^N angles that are close to 90°. Coincidentally, this is the angles between bidirectional p_x, p_y and p_z atomic orbitals. Hence, the higher p-character of E–N bonds, the lesser the strain. In this respect, germanide **12** is a rather unstrained system since, according to computational NBO analysis, its E–N bonding orbitals have 93.8% of p-character on average, and its crystallographic N^Ge^N angles are around 90° (see *Section S3*). Protonation of its anionic lone pair, yielding **A**, should steeply increase its p-character, in accordance with Bent's rule.[38] Consequently, this should lead to a larger s-character the Ge–N bonding orbitals, thus requiring more obtuse N^Ge^N angles. Such a change should be in conflict with the preference of the TSMP scaffold, thus generating strain within the cage structure. This strain could be relieved by coordination of a chloride anion (intermediate **B**). In other words, germane **A** should exhibit strain-release Lewis acidity. It is reasonable to assume that protonation of silanide TSMPSi (**1**) with HCl proceeds in a similar fashion, yet, due to generally higher reactivity of Si compounds, degradation occurs at some stage after coordination of a chloride anion.

If the above reasoning is correct, protonated TSMPSi (**1**) may be possible to obtain using acids with non-coordinating anions. Indeed, reacting **1** with an equimolar amount of Brookhart's acid ($HBAr^F{}_4{\cdot}2Et_2O$) resulted in successful protonation and formation of the desired cationic silane $[TSMPSiH]^+BAr^F{}_4{}^-$ (**2^BARF^**).

S5.1.2 Base-catalyzed isomerization of fluorene 5

^{1}H NMR shows no direct protonation of zwitterionic TSMPSi (**1**) by CH-acid **5** (pK_a^{DMSO} = 8.1[47]), however the spectrum displays a number of new peaks that do not correspond to any of the starting materials (labelled with asterisks in *Figure S8*).

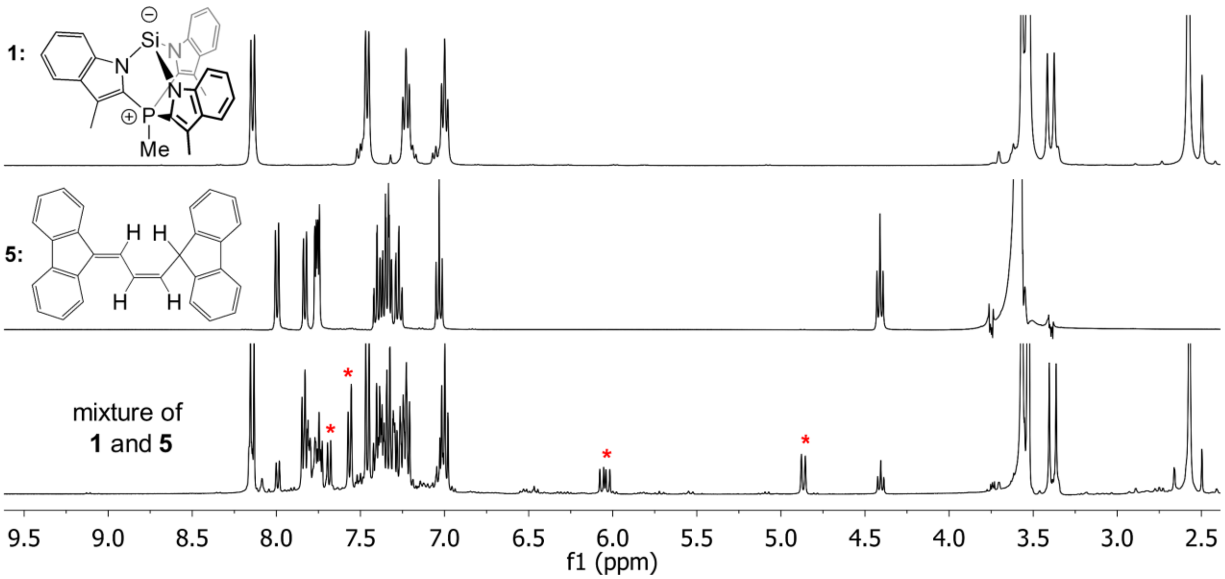

Figure S8. ^{1}H NMR (400 MHz) spectra of silanide **1**, fluorene **5** as well their mixture in dioxane-d_8. Spectrum of **5** was recorded dioxane-h$_8$ with WET solvent suppression. In the bottom panel, asterisks indicate some of the new peaks.

It is known from the literature[47,48] that **5** can convert into the tautomeric form **5-iso** with a close pK_a^{DMSO} (*Scheme S6*). Unfortunately, the authors did not publish NMR spectra of the tautomer that we could use for a reference.

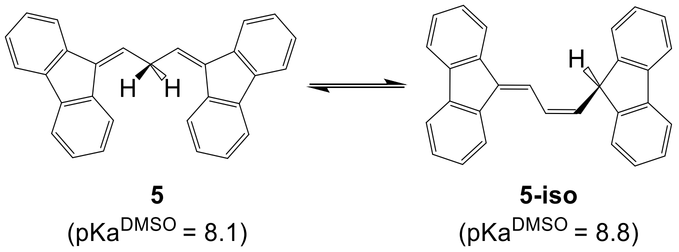

Scheme S6. Tautomerization of fluorene **5**.[47,48]

However, 1D zTOSCY experiments performed with selective excitation of a new doublet at 4.87 ppm (**A** in *Figure S9*) show four signals with chemical shifts and multiplicities consistent with the non-aromatic spin system of **5-iso**. According to NMR, pure **5** does not tautomerize in dioxane for at least a month. This implies that, even though proton transfer does not occur to a measurable extent, zwitterion **1** may act as a catalytic base that provides a pathway for tautomerization of **5**. A steady concentration of deprotonated **5** in solution is indirectly confirmed by a pale-blue coloration of the mixture of **1** and **5** in dioxane-d_8, which is characteristic of the presence of deprotonated **5**. Consequently, the pK_a of protonated zwitterion **1** is not much lower than that of fluorene **5**.

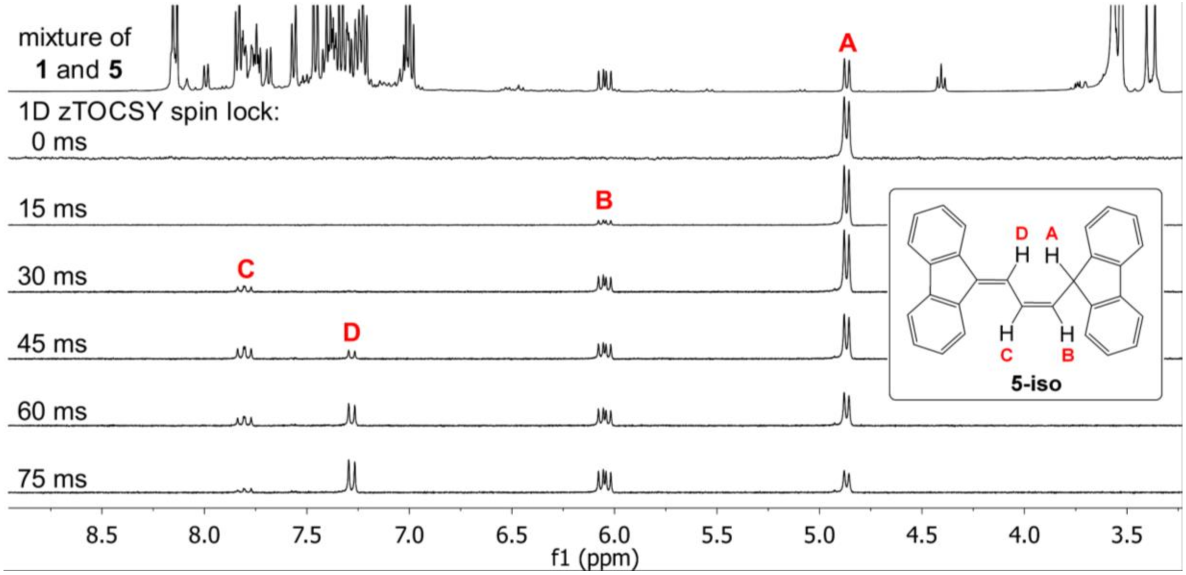

Figure S9. ^{1}H 1D zTOCSY NMR (400 MHz) of a mixture of silanide **1** and fluorene **5** in dioxane-d_8. Top panel is a regular ^{1}H NMR spectrum for a reference. Signal **A** is selectively excited.

S5.1.3 Comparison of geometries of P^{+}- and Si-tethered analogues

Calculated selected geometric parameters of the zwitterion TSMPSi (**1**), corresponding cationic silane [TSMPSiH]$^{+}$ (**2**) and their isoelectronic Si-tethered analogues are shown in *Table S8*. Geometries were optimized in vacuum at B3LYP-GD3BJ/6-311++G(d,p) level of theory. Whereas most of the bond lengths and angles match closely, the positive phosphonium atom is slightly smaller than the isoelectronic silicon (longer Si–C^{Ar} bonds compared to P^{+}–C^{Ar}), which should lead to a small difference in strain.

Table S8. Comparison of the calculated geometries.

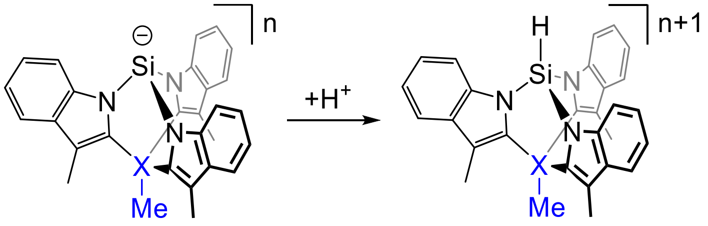

Structure	Average distances, Å					Average angles, degrees			
	N–Si	C^{Ar}–N	X–C^{Ar}	X–CH_3	Si–H	N^Si^N	Si^N^C	N^C^X	C^{Ar}^X^C^{Ar}
**A**, X=P^+ (**1**)	1.89	1.40	1.78	1.80	-	91.6	126.6	111.8	104.3
**A**, X=Si	1.88	1.41	1.87	1.87	-	93.6	126.4	112.5	102.0
**B**, X=P^+ (**2**)	1.76	1.42	1.79	1.80	1.46	101.8	118.4	112.7	103.7
**B**, X=Si	1.75	1.43	1.88	1.86	1.46	103.9	118.1	113.5	101.1

The table above shows that in terms of bond lengths and angles, both before and after quaternization, the analogy between TSMPSi (**1**) and its Si-tethered analogue is maintained.

S5.1.4 Gas-phase acidities

Gas-phase acidities (ΔG) were calculated in vacuum using both B3LYP-GD3BJ and MP2 with the 6-311++G(d,p) basis set. For MP2, thermal corrections for Gibbs free energy were taken from B3LYP-GD3BJ/6-311++G(d,p) calculations in vacuum. It has been shown[40,41] that MP2-derived gas-phase acidities tend to give a better agreement with the experiment than B3LYP for a wide range of acids; nevertheless, both methods show identical trends. This was also true in our calculations. Due to higher precision, we chose MP2-calculated values for a further discussion. Gas-phase pK_a's at 298K were calculated as 0.733ΔG. Calculated and available experimental gas-phase acidities are shown in *Table S9*.

Table S9. Calculated and experimentally available free energies and pK_a for spontaneous dissociation of the first silicon-bound proton in the gas phase.

SiH_4	$HSiCl_3$	$HSi(SiCl_3)_3$	$HSi(SiMe_3)_3$	$HSi(NMe_2)_3$	$HSiPh_3$	$HSiMe_3$
**A1**	**A2**	**A3**	**A4**	**A5**	**A6**	**A7**

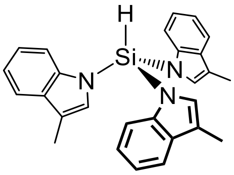

A8

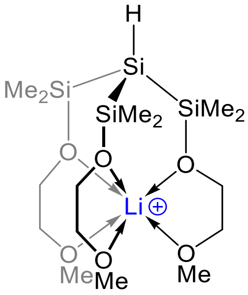

A9

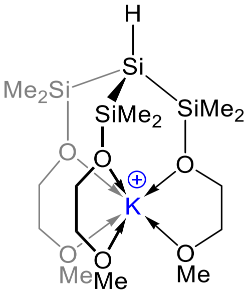

A10

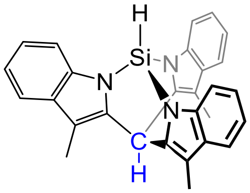

A11

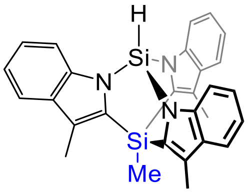

A12

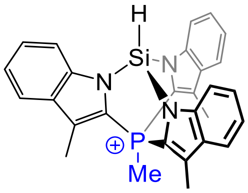

2

**Acid**	**AH → H⁺ + B**				
	**ΔG, kcal/mol**			**pK_a**	
	**ΔG^{DFT}**	**ΔG^{MP2}**	**ΔG^{exp}**	**pK_a^{DFT}**	**pK_a^{MP2}**
A1	363.9	370.4	365.7 ± 2.0[49]	266.9	271.6
A2	319.9	332.8	<353.3 ± 2.5[49]	234.6	244.1
A3	294.9	300.8	-	216.3	220.6
A4	347.8	344.3	346 ± 3[49]	255.0	252.5
A5	375.4	377.0	-	275.3	276.4
A6	357.8	356.4	-	262.4	261.4
A7	377.8	379.9	-	277.1	278.6
A8	333.7	329.4	-	244.7	241.6
A9	272.8	269.7	-	200.1	197.8
A10	278.2	276.2	-	204.0	202.5
A11	316.9	313.0	-	232.4	229.6
A12	324.5	319.8	-	238.0	234.5
**2**	248.9	242.4	-	182.5	177.7

S5.1.5 On the ring strain in bicyclic systems

Quaternization of a silicon atom in the discussed bicyclic silanes results in a build-up of ring strain. The latter originates from rehybridization of the silicon as its lone pair gets bound by the acceptor. In accordance with Bent's rule,[38] this binding increases p-character of the lone pair, which in turn leads to a larger s-character of the Si–N bonding orbitals giving more obtuse N^Si^N angles. These angles might differ from what is preferred by the rest of the cage structure, resulting in strain. In order to get a quantitative estimate of this effect, and how it differs depending on the size of a bridgehead atom, we resorted to DFT calculations.

The homodesmotic reaction scheme in *Table S10* enables to evaluate the strain depending on the nature of a bridgehead atom R and the fourth substituent on silicon R'. It should be noted that this scheme cannot be applied to the silanide TSMPSi (**1**) due to its zwitterionic nature, because otherwise it would lead to a different number of charged species on both sides of the equation. Instead of **1**, we used its isoelectronic Si-tethered analogue. The latter roughly retains the bond lengths and angles compared to the original phosphonium compound (see *Section S5.1.3*), and therefore the steric effects. Nevertheless, its use conserves the number of charges, thus allowing to evaluate the emergent strain without ion destabilization effects in vacuum. Finally, as a measure of strain we chose the reaction enthalpy (ΔH) since it has been used for this purpose historically.[50]

Table S10. Strain in the Si- and C-tethered tricyclic silanes as well as in their quaternized forms. Level of theory: B3LYP-GD3BJ/6-311++G** in vacuum.[a] Notations: n=0,-1; LP=lone pair.

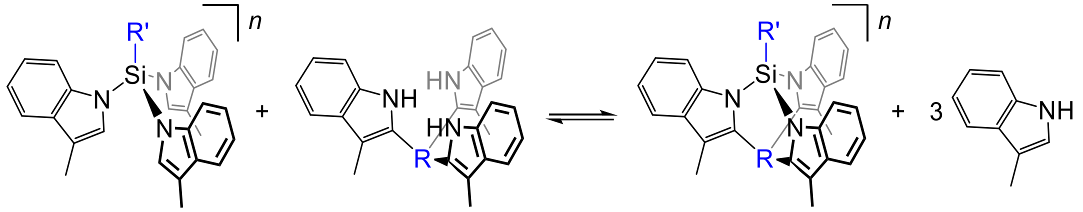

**R'**	**R=SiMe**		**R=CH**	
	**$\Sigma_{N\text{^}Si\text{^}N}$, °**	**ΔH, kcal/mol**	**$\Sigma_{N\text{^}Si\text{^}N}$, °**	**ΔH, kcal/mol**
LP	280.9	22.4	268.9	14.5
	↓ +12.2	↓ +4.5	↓ +12.1	↓ +4.2
$FeBr_2$(THF)	293.1	26.9	281.0	18.7
	↓ +16.7	↓ +3.8	↓ +17.3	↓ +9.8
Me	309.8	30.7	298.3	28.5
	↓ -6.4	↓ +0.7	↓ +2.2	↓ +2.5
H	303.4	31.4	300.5	31.0

Following the above analysis, complexation of the Si-tethered silane (R=SiMe) with an $FeBr_2$(THF) center leads to 4.5 kcal/mol of an increase in strain, whereas methylation and protonation give 8.3 and 9.0 kcal/mol, respectively, confirming a likely contribution of ring strain to the weakened nucleophilicity of TSMPSi (**1**). Derivatives of the C-tethered silane (R=CH) are at least as much or more strained than the corresponding Si-tethered analogues due to a smaller size of a bridging C-atom.

---

[a] In a previous paper from our group,[37] TPSS/TZVP level of theory was used in order to estimate the strain of cage structures. Herein, we reconsider this choice in favor of B3LYP-GD3BJ/6-311++G(d,p). Primarily, because it involves an empirical dispersion correction (GD3BJ) that favors the side of a homodesmotic equation with the least number of particles. Furthermore, geometries optimized at B3LYP-GD3BJ/6-311++G(d,p) level of theory show a good agreement with the data obtained from the X-ray crystal structure (*Table S11*).

Table S11. Selected crystal structure (cryst.) and computationally-optimized (theor.) bond distances and angles for silanide **1**. For the crystal structure, both independent molecules are considered (see *Section S3*).

	**Distances, Å**				**Angles, °**			
	**Si-N**	**N-C^{Ar}**	**C^{Ar}-P**	**P^+-CH_3**	**N^Si^N**	**Si^N^C**	**N^C^P**	**C^{Ar}^P^C^{Ar}**
cryst.	1.8404(18)-<br>1.8639(18)	1.400(3)-<br>1.408(2)	1.768(2)-<br>1.7782(19)	1.780(2),<br>1.782(2)	91.87(8)-<br>93.34(7)	125.05(13)-<br>126.36(14)	111.00(14)-<br>112.14(14)	101.40(10)-<br>105.15(9)
theor.	1.890	1.401	1.780	1.803	91.60	126.6	111.8	104.3

Despite the protonated Si-tethered cage being more strained than the methylated one, its sum of N^Si^N angles ($\Sigma_{N\text{^}Si\text{^}N}$) is smaller than in the methylated structure. This suggests that the precise relationship between $\Sigma_{N\text{^}Si\text{^}N}$ and ring strain depends on the fourth substituent on the silicon atom.

S5.1.6 Anionic NLMO on a silicon in TSMPSi (1)

Natural Localized Molecular Orbital (NLMO) analysis within the NBO framework shows that the anionic lone pair in TSMPSi (**1**) is by 98.1% localized on a silicon atom, with minor delocalization into σ*(C-N) due to hyperconjugation (*Figure S10*). In other words, the Lewis structure **1** used to denote the zwitterionic silanide is a good description of its electronic structure. This also means that interaction between zwitterionic charges is almost entirely of electrostatic nature.

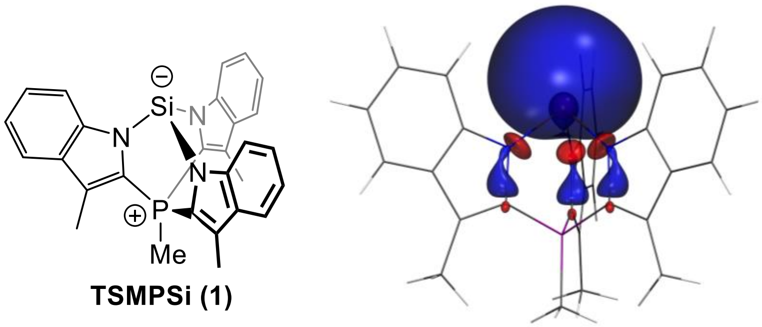

Figure S10. NLMO plot of anionic lone pair in TSMPSi (**1**) (isocontour=0.02). Calculations were performed in vacuum at B3LYP-GD3BJ/6-311++G** level of theory.

S5.1.7 Natural Bonding Orbital (NBO) analysis of the deprotonation

In order to establish the electronic reasons why imposing acute N^Si^N angles increases acidity, we resorted to Natural Bonding Orbital (NBO) analysis. It was performed according to the deprotonation decomposition scheme used in *Figure 4B* (see main text) for the same structures. The results are shown in *Table S12*. The latter contains the orbital composition of the Si-based hybrid in the Si–H NBO which transforms into a lone pair NBO upon deprotonation (B/LP on Si). Additionally, the table shows compositions of hybrids on silicon and nitrogen in Si–N NBO as well as Natural Hybrid Orbital (NHO) bending angles for the respective hybrids.

Table S12. NBO analysis according to deprotonation energy decomposition scheme in *Figure 4B* (see main text). Deprotonated states are shown in square brackets. Calculations were performed in vacuum at B3LYP-GD3BJ/6-311++G** level of theory.

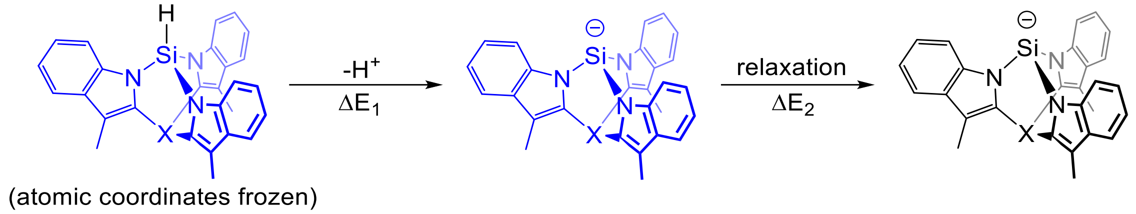

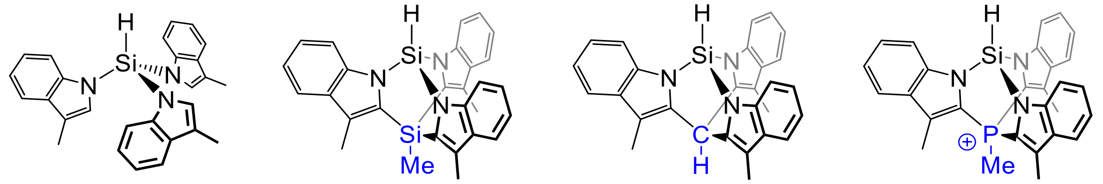

A (X = 3 x H) **B** (X = SiMe) **C** (X = CH) **2** (X = P$^+$Me)

Structure	NBO composition			NHO bending angles	
	B/LP on Si	Si in Si-N	N in Si-N	Si → N	N → Si
**A**	$sp^{2.04}d^{0.02}$	$sp^{3.40}d^{0.06}$	$sp^{1.93}$	2.5°	<1.0°
**[A-H$^+$]$^-$ (frozen)**	$sp^{1.01}$	$sp^{4.86}d^{0.08}$	$sp^{1.76}$	13.9°	3.3°
**[A-H$^+$]$^-$ (relaxed)**	$sp^{0.46}$	$sp^{8.33}d^{0.13}$	$sp^{2.05}$	1.4°	5.9°
**B**	$sp^{2.01}d^{0.02}$	$sp^{3.42}d^{0.06}$	$sp^{2.04}$	5.7°	4.4°
**[B-H$^+$]$^-$ (frozen)**	$sp^{1.03}$	$sp^{4.82}d^{0.08}$	$sp^{1.80}$	7.3°	3.0°
**[B-H$^+$]$^-$ (relaxed)**	$sp^{0.47}$	$sp^{8.18}d^{0.12}$	$sp^{2.11}$	2.9°	≤1.0°
**C**	$sp^{1.90}d^{0.02}$	$sp^{3.50}d^{0.06}$	$sp^{2.04}$	12.2°	7.9°
**[C-H$^+$]$^-$ (frozen)**	$sp^{0.83}$	$sp^{5.51}d^{0.09}$	$sp^{1.78}$	≤1.0°	6.1°
**[C-H$^+$]$^-$ (relaxed)**	$sp^{0.43}$	$sp^{8.71}d^{0.12}$	$sp^{2.16}$	7.7°	2.9°
**2**	$sp^{1.82}d^{0.02}$	$sp^{3.57}d^{0.06}$	$sp^{1.98}$	6.9°	4.1°
**[2-H$^+$]^0 (frozen)**	$sp^{0.88}$	$sp^{5.31}d^{0.09}$	$sp^{1.76}$	5.2°	2.3°
**[2-H$^+$]^0 (relaxed)**	$sp^{0.41}$	$sp^{9.11}d^{0.14}$	$sp^{2.07}$	4.0°	1.2°

The analysis shows that the s-orbital fraction in the Si-based hybrid of Si–H NBO increases from **A** to **C** as the sum of N^Si^N angles ($\Sigma_{N^\wedge Si^\wedge N}$) decreases. Thus, hybridization in tris-*N*-skatyl silane (**A**; $\Sigma_{N^\wedge Si^\wedge N}$=324.7°) is $sp^{2.04}d^{0.02}$, whereas in increasingly more constrained Si- and C-tethered analogues **B** ($\Sigma_{N^\wedge Si^\wedge N}$=311.6°) and **C** ($\Sigma_{N^\wedge Si^\wedge N}$=300.5°) it is $sp^{2.01}d^{0.02}$ and $sp^{1.90}d^{0.02}$, respectively. This makes strained silanes more prone to heterolytic dissociation since Si–H bond polarization shifts towards silicon (better illustrated in *Table S13*).

The increasing strain from **A** to **C** is also reflected in the Si–N NHO bending angles in *Table S12*. While the bending is rather low in **A** (2.5°/<1.0°), it significantly increases in **B** (5.7°/4.4°) and **C** (12.2°/7.9°), which is in line with the trend we found using homodesmotic schemes to analyse the

strain in **B** and **C** (see *Section S5.1.5*). The bending in **2** (6.9°/4.1°) is rather similar to that in **B**, which justifies our choice of **B** as a neutral analogue of **2** (see *Section S5.1.3*).

Upon proton abstraction with nuclear coordinates frozen, hybridization in all three silanes changes dramatically towards a greater s-contribution in accordance with Bent's rule.[38] Final geometry relaxation of all deprotonated silanes maximizes contribution of the 3s-orbital to the lone pair on silicon as well as minimises strain (see corresponding NHO bending angles).

As for [TSMPSiH]$^+$ (**2**; $\Sigma_{N^\wedge Si^\wedge N}$=305.5°), while it is rather geometrically similar to **B** (see *Section S5.1.3*), the 3s-contribution to the Si–H bonding pair ($sp^{1.82}d^{0.02}$) is larger than for any other discussed silane, which we associate with the presence of a positive charge in the molecule. Other than that, upon deprotonation, its orbital compositions and NHO bending angles evolve in the same way as outlined above for **A**-**C**.

Table S13. NBO analysis for a Si–H bond in silanes in *Table S12*. Calculations were performed in vacuum at B3LYP-GD3BJ/6-311++G** level of theory.

Silane	Si-H NBO			Atomic contributions to Si-H NLMO	
	Hybridization on Si	Atomic contributions			
		%Si	%H	%Si	%H
**A**	$sp^{2.04}d^{0.02}$	41.34	58.66	41.35	57.83
**B**	$sp^{2.01}d^{0.02}$	41.80	58.20	41.72	57.56
**C**	$sp^{1.90}d^{0.02}$	42.49	57.51	42.58	56.93
**2**	$sp^{1.82}d^{0.02}$	43.50	56.50	43.45	55.98

S5.2 Properties and reactivity of the zwitterionic TSMPSi (1)

S5.2.1 On donicity of TSMPSi (1)

The assessment of the donicity is often done experimentally by comparing $\tilde{\nu}$(CO) of the infrared band corresponding to the carbonyl A1 stretching mode in $LNi(CO)_3$ complexes, as originally suggested by Tolman.[51] He also pointed out that meaningful data could be obtained with other carbonyls, and $LNi(CO)_3$ complexes were chosen primarily due to the ease of preparation. Indeed, reasonable results have been obtained using carbonyl complexes of manganese,[52] chromium,[53–56] vanadium,[57] molybdenum,[57–61] tungsten,[53,57,61–64] iron,[10,65–69] rhodium[70–76] and iridium.[73,74,77] For our purpose, we chose iron carbonyls with a general formula of $LFe(CO)_4$. This was done following the work of Barnard and Mason[10] who used this approach to characterize structurally-related phosphines.

The respective $LFe(CO)_4$ complexes, (TSMPSi)$Fe(CO)_4$ (**8**) and the germanium analogue (TSMPGe)$Fe(CO)_4$ (**17**) used for the comparison, were prepared by reaction of the corresponding zwitterions, TSMPSi (**1**) and TSMPGe (**12**; see *Section S5.1.1*), with $Fe_2(CO)_9$ in THF. Solution IR spectra of both **8** and **17** in THF show three strong absorptions at around 2000 cm^{-1} consistent with trigonal bipyramidal geometry of the carbonyl complexes.[10,11] The experimental carbonyl stretching mode frequencies in compounds **8**, **17** as well as some other reported $LFe(CO)_4$ complexes are given

in a *Table S14*. The sequence of fully symmetrical (A1) carbonyl stretching mode frequencies, ṽ$(CO)_1$, is plotted in *Chart S1*.

As expected, the silanides and germanides are more donating than neutral phosphines. Also, germanides tend to be less electron-rich than corresponding silanides due to the slightly higher electronegativity of germanium. It is interesting, however, that steric constraint leads to less donation as exemplified by phosphines **C** and **D**. In their original paper,[10] Barnard and Mason propose that this stems from increased s-character of the lone pair in **D** due to smaller N^P^N angles. Since s-orbitals are closer to the nucleus than the p-orbitals, the higher s-contribution results into less donation. This explanation is in line with the modern understanding of a connection between acid-base properties and strain.[78,79] In this vein, TSMPSi (**1**) and TSMPGe (**12**) should be more donating than their C-tethered counterparts **A** and **B**. Thus, the sum of N^Si^N angles in the crystal structure of **1** is 277.43(13)/277.51(14)° (see *Section S3*) which is larger than 272.58(8)° [37] in **A**; the same applies for the N^Ge^N sum in **12** , 270.04(19)° (see *Section S3*), *vs.* that in **B**, 263.6(4)°.[11] However, judging from the carbonyl stretching frequencies, the opposite is true. Our interpretation is that a positive charge in formally neutral **1** and **12** makes them less donating than anionic **A** and **B**.

Table S14. Solution carbonyl stretching frequencies in $LFe(CO)_4$ complexes according to infrared spectroscopy. Complexes **III**-**VIII** were measured in hexane, while the rest were measured in THF due to low solubility in hexane. For the sake of comparison, the solvent effects were assumed to be negligible. References in the table show the literature source where a complex was first characterized.

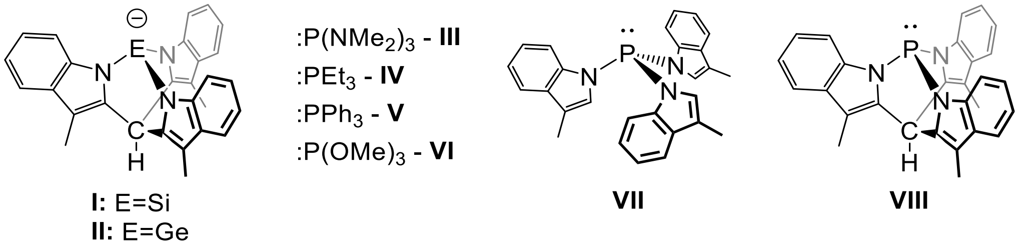

L	ṽ(CO), cm^{-1}			L	ṽ(CO), cm^{-1}		
	ṽ$(CO)_1$	ṽ$(CO)_2$	ṽ$(CO)_3$		ṽ$(CO)_1$	ṽ$(CO)_2$	ṽ$(CO)_3$
**I**[37]	2029	N/A	1920	**IV**[80]	2051	1976	1938
**II**[11]	2037	1954	1933	**V**[81]	2052	1979	1947
**TSMPSi (1)**	2039	1960	1926	**VI**[82]	2065	1992	1966
**TSMPGe (12)**	2047	1970	1941	**VII**[10]	2065	1997	1966
**III**[83]	2048	1973	1936	**VIII**[10]	2076	2006	1977

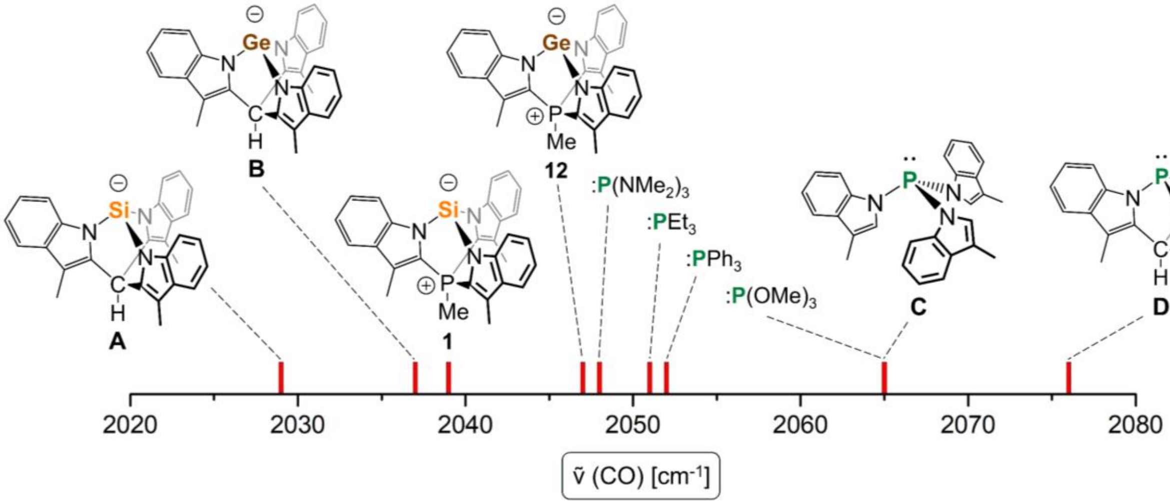

Chart S1. Experimental ṽ(CO) of carbonyl A1 stretching modes in $LFe(CO)_4$ complexes for different L.

This analysis, however, comes with two caveats. Firstly, the span of the comparison is limited to the ligands the IR data for the corresponding complexes of which are available, which is very few. Secondly, according to a number of authors,[42,84] not all carbonyl complexes are equally suitable for making conclusions about the donor strength. It has been shown that carbonyl stretching modes in nickel, iron and molybdenum carbonyls are contaminated by mode-mode coupling. The same applies for the derivatives.[84] In very simple terms, coupling arises when not all vibrational energy is concentrated within one bond but is rather delocalized among several bonds of similar energies/force constants and reduced masses. For $LNi(CO)_3$ complexes, the contribution associated with mode-mode coupling can reach as much as 100 cm^{-1} for $L=F^-$.[84] The stretching modes can also be sensitive to sterics and trans effects, which leads to additional contributions to ṽ(CO) not associated with electronic properties of the ligand. These problems can be overcome by calculating a local Tolman's electronic parameter (LTEP) based on the inherent bond strength defined by the magnitude of the local bond force constant.[84] An alternative computational solution was suggested by Gusev.[42] His argument stems from the fact that there should be a linear correlation between ṽ(CO) and C–O bonding distance for a complex that is used to gauge electronic properties of a ligand. In $LNi(CO)_3$, a good correlation is found for phosphines and some carbenes but not O- and N-donor ligands. Gusev then suggests a model complex, IrCp(CO)L, in which a linear correlation is observed for a range of ligand families. In essence, this solves the problem of mode-mode coupling, sterics and *trans*-effects in a very general way, since a linear correlation is only observed if these are minimized.

Calculated ṽ(CO) for the model IrCp(CO)L complexes of TSMPSi (**1**), TSMPGe (**12**) and other ligands with a range of donor strengths, along with corresponding C–O bonding distances, d(CO), are given in *Table S15* and plotted in *Chart S2*. There is a linear correlation between ṽ(CO) and d(CO) for all featured ligands (see *Figure S11*). In general, the order of donor strength is the same as derived from the experimental data for the $LFe(CO)_4$ complexes, which reinforces prior conclusions. Additional data points with silanide **A** and germanide **C** confirm that steric constraints lead to the reduction of donor strength for silicon and germanium compounds just like for phosphines. Furthermore,

introduction of a positive charge makes TSMPSi (**1**) more alike to silylenes rather than silicon anions in terms of donor strength. Out of all considered simple phosphines, **1** is most similar to $P(NMe_2)_3$.

We choose to limit our discussion of the donicity of TSMPSi (**1**) to the IrCp(CO)L model since the latter provides comparison to a larger number of ligands and is expected to be more precise because of minimized mode-mode coupling, sterics and trans effect contributions. Yet, as noted by Gusev,[42] quantifying ligand donor properties is only useful as long as it helps to rationalize and predict the reactivity patterns in coordination chemistry and catalysis. He also argues that ligand properties are not always adequately represented as a single parameter for different ligand types.

Table S15. Calculated carbonyl stretching frequencies in IrCp(CO)L model complexes for different L. MPW1PW91 functional with SDD basis set (associated with ECP) for Ir and 6-311+G(d,p) for all other atoms was employed, along with tight geometry optimizations and ultrafine integration grid. References in the table show the literature source where a ligand was first discussed.

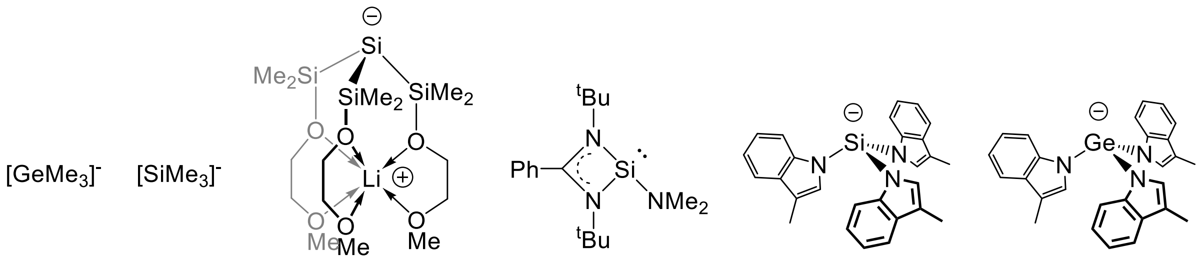

I **II** **III** **IV** **V** **VI**

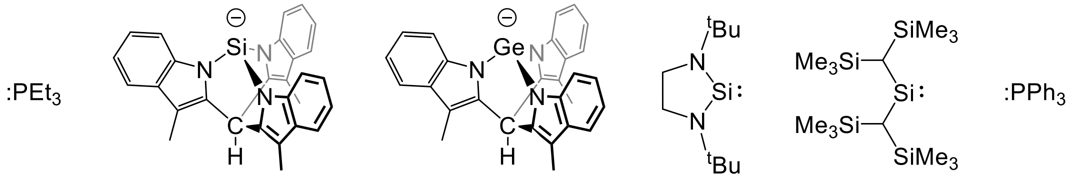

VII **VIII** **IX** **X** **XI** **XII**

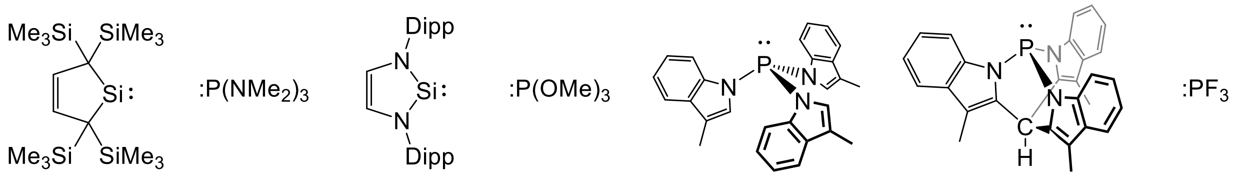

XIII **XIV** **XV** **XVI** **XVII** **XVIII** **XIX**

L	ṽ(CO), cm^{-1}	d(CO), Å	L	ṽ(CO), cm^{-1}	d(CO), Å	L	ṽ(CO), cm^{-1}	d(CO), Å
**I**	1955.52	1.17509	**VIII**[37]	2037.65	1.15981	**1**	2053.29	1.15690
**II**	1956.26	1.17521	**IX**[11]	2038.47	1.15933	**12**	2055.97	1.15614
**III**[85]	1988.81	1.16801	**X**[86]	2044.39	1.15835	**XV**[87]	2068.56	1.15334
**IV**[88]	2002.02	1.16554	**XI**	2045.98	1.15710	**XVI**	2078.12	1.15255
**V**	2020.98	1.16267	**XII**	2048.73	1.15728	**XVII**[10]	2086.11	1.15057
**VI**	2024.77	1.16163	**XIII**	2050.72	1.15618	**XVIII**[10]	2100.43	1.14838
**VII**	2035.29	1.15984	**XIV**	2050.74	1.15740	**XIX**	2114.81	1.14648

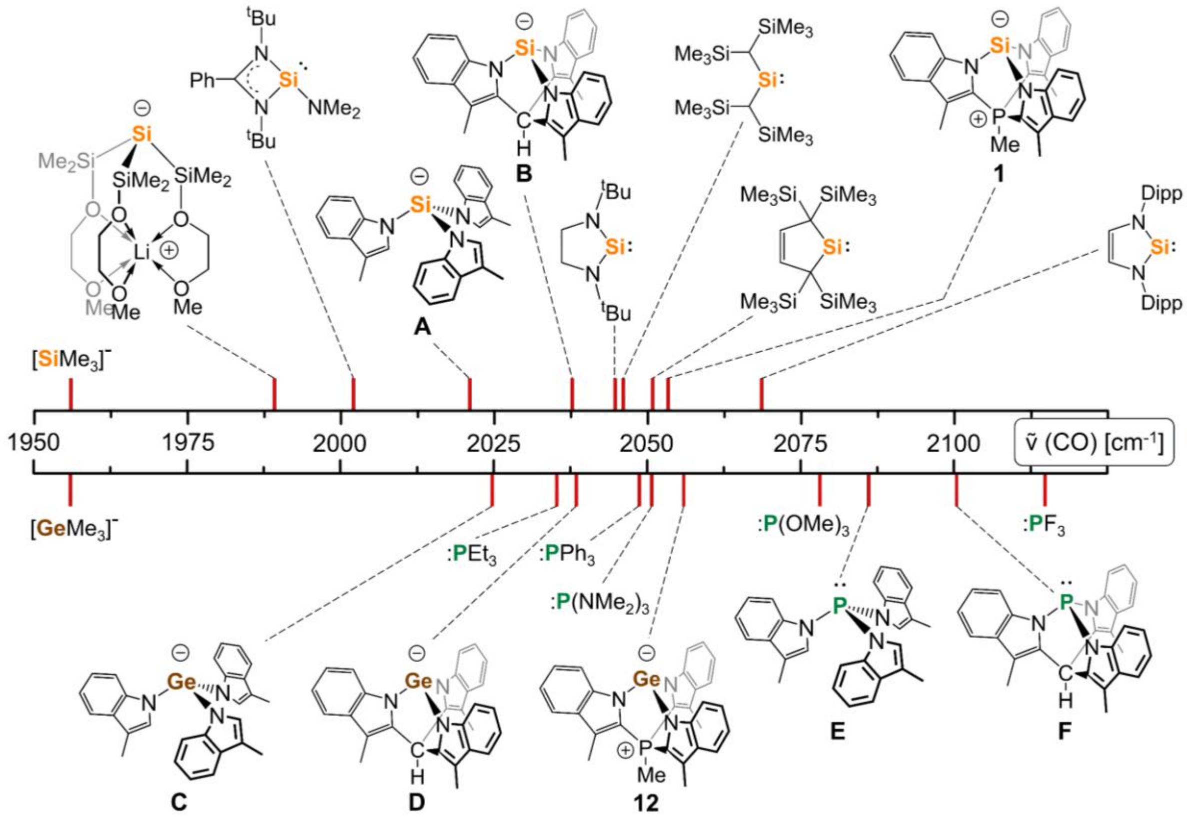

Chart S2. Calculated ṽ(CO) of the carbonyl stretching mode in IrCp(CO)L complexes for different L.

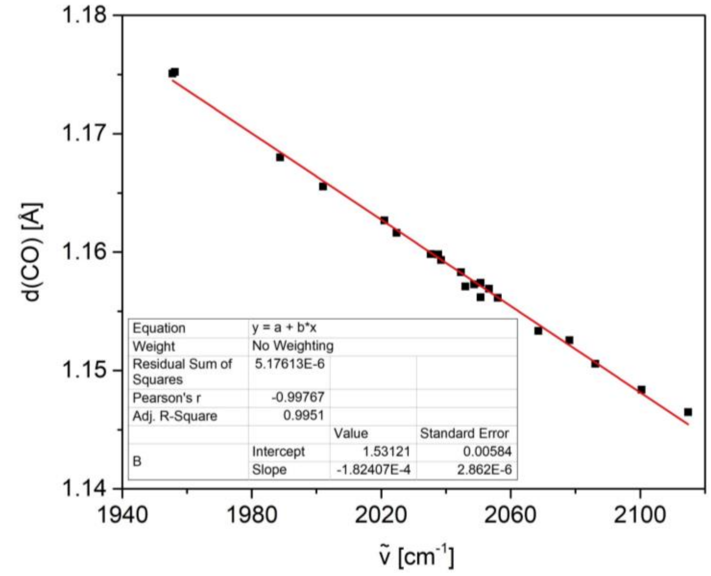

Figure S11. Linear correlation between calculated C-O bonding distances and stretching frequencies for IrCp(CO)L model complexes.

S5.2.2 Remarks on isomerization into iso-TSMPSi (10)

^{1}H NMR spectroscopic observations in THF-d_8 show that complete isomerization of an otherwise kinetically stable TSMPSi (**1**) into iso-TSMPSi (**10**) occurs in the presence of MeOTf (0.1 equiv.) or $HBAr^F_4{\cdot}2Et_2O$ (0.25 equiv.) and after formation of the respective quaternization products, $[TSMPSiMe]^+OTf^-$ (**9**) or $[TSMPSiH]^+BArF_4^-$ ($\mathbf{2}^{\mathbf{BARF}}$). The isomerization also occurs in the presence of CH-acids (see *Section S5.3.4*). This leads one to conclude that the isomerization is catalytic in nature and is linked to partial quaternization of anionic silicon in zwitterion **1**. Based on this, we suggest a possible mechanism for this process in *Scheme S7*.

Initiation:

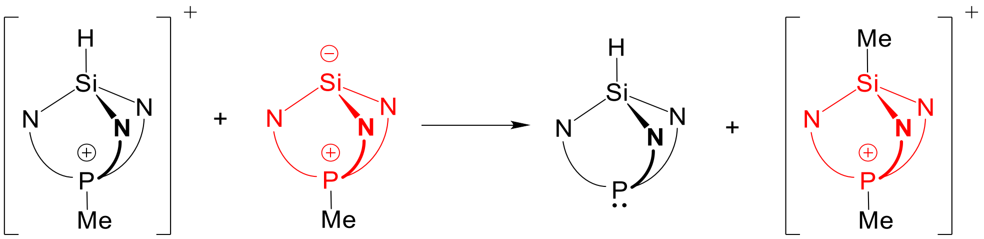

[TSMPSiH]⁺ (2) **TSMPSi (1)** **[TSMPSiMe]⁺ (A)**

Catalysis:

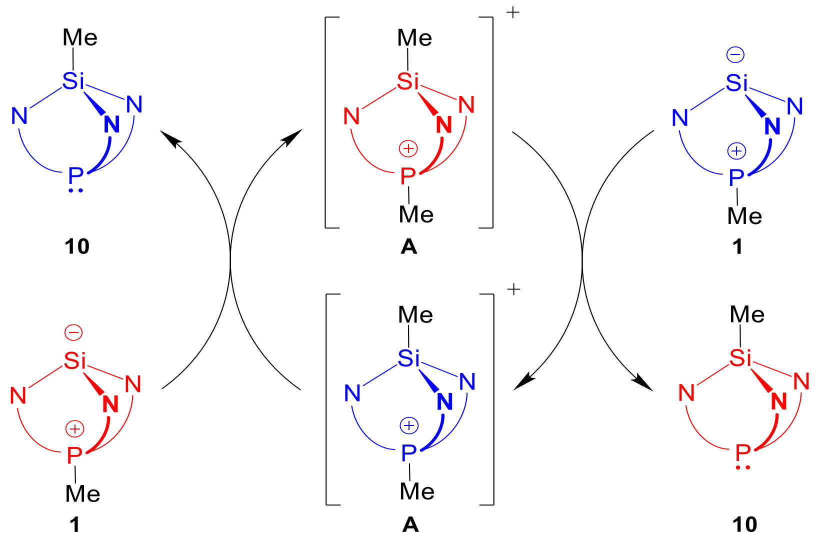

Scheme S7. Suggested mechanism for isomerization of TSMPSi (**1**). The path of an individual molecule, **1**→**A**→**10**, is coded in colour.

The first stage of the mechanism, initiation, only applies if TSMPSi (**1**) is exposed to a protonating agent, i.e. $HBAr^F_4{\cdot}2Et_2O$ or a CH-acid (see *Section S5.3.4*), and is meant to generate a catalytically active species – $[TSMPSiMe]^+$ (**A**). The latter also directly forms in a reaction of **1** with MeOTf. Initiation involves transfer of a methyl group from silane $[TSMPSiH]^+$ (**2**) to a molecule of **1**. This transfer is likely possible due to overall positive charge of **2** along with its ring strain (see *Section S5.1.5*). The second stage is catalytic and employs **A**, which transfers its phosphonium methyl group to a molecule of **1**, thus generating another molecule of **A** and the final electroneutral product iso-TSMPSi

(**10**). Again, methyl transfer from **A** is likely possible due to strain, analogously to that in **2**. The driving force for isomerization is cancellation of the separated charges in zwitterion **1**.

Pure iso-TSMPSi (**10**; >98% by ^{1}H NMR) can be quantitatively prepared by stirring zwitterion TSMPSi (**1**) with 0.25 equiv. of potassium graphite in benzene. Whereas the reaction mechanism is unclear in this case, this method was used to prepare clean samples of **10** for full characterization.

S5.3 Properties and reactivity of the cationic silane [TSMPSiH]$^{+}$ (2)

S5.3.1 ^{1}H NMR of the THF ring-opening reaction

In contrast with the absence of reactivity observed in dioxane, attempted protonation of zwitterionic TSMPSi (**1**) in THF-h_8 with an approximately equimolar amount of fluoradene (**4**) led to a rather complex ^{1}H NMR spectrum (*Figure S12*). Thus, it shows the presence of the original CH-acid **4** and isomeric iso-TSMPSi (**10**) (confirmed by ^{29}Si and ^{31}P NMR spectra as well). Four other signals, $CH_2^{A\text{-}D}$, do not appear if the reaction is run in THF-d_8 instead of THF-h_8, implying that they originate from THF ring-opening. These signals, as well as some others below 6.5 ppm, display line broadening indicative of a dynamic process on the measurement timescale. It is reasonable to assume that they belong to the same species. We assign these resonances to a Si–H proton, a phosphonium methyl group, and aromatic methyl groups. This is consistent with the formation a THF ring-opening product **11a/b**.

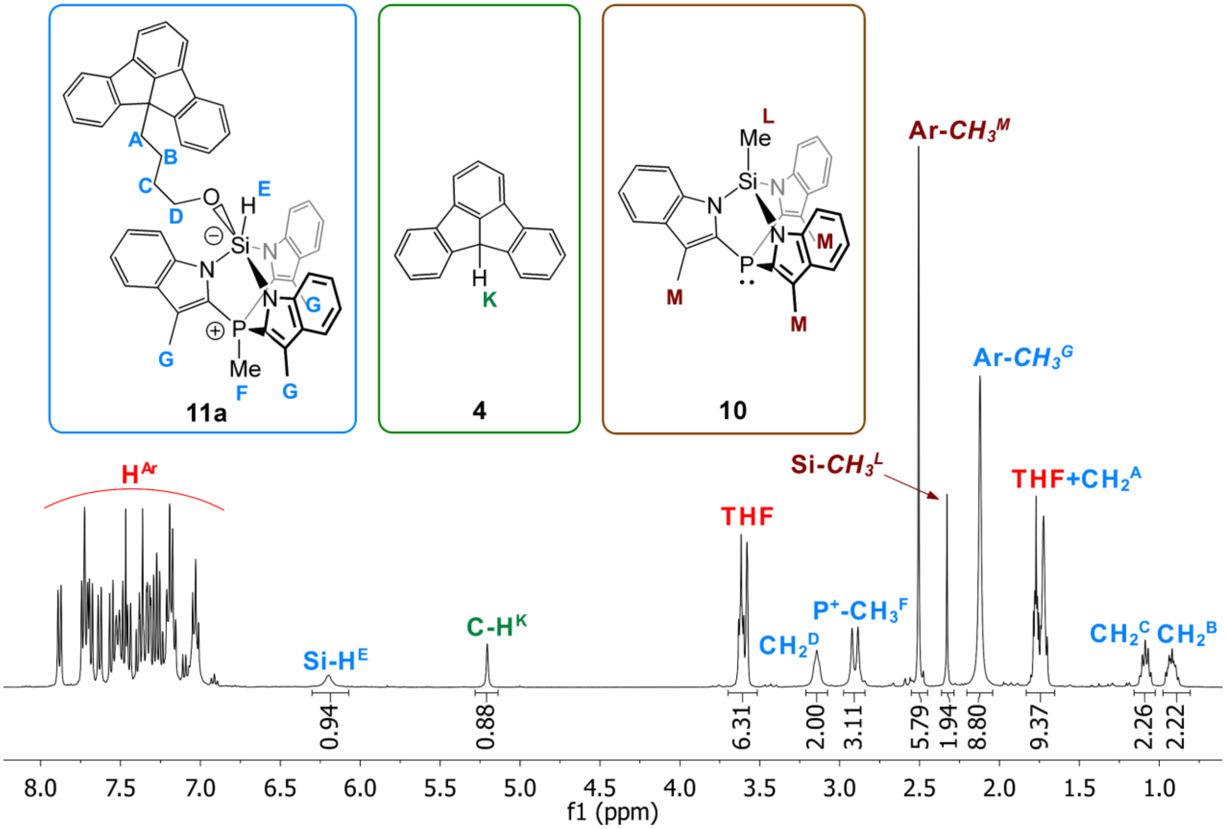

Figure S12. ^{1}H NMR (400 MHz) spectrum of the reaction between TSMPSi (**1**), fluoradene (**4**) and THF-h_8, recorded in THF-d_8 at 25 °C. Even though **11a** is in thermal equilibrium with the cage-opening product **11b**, only the structure of **11a** is shown for simplicity.

S5.3.2 ^{1}H gCOSY NMR of the THF ring-opening reaction

^{1}H COSY spectrum in *Figure S13* shows that the methylene protons of the THF ring-opening product constitute an isolated spin system.

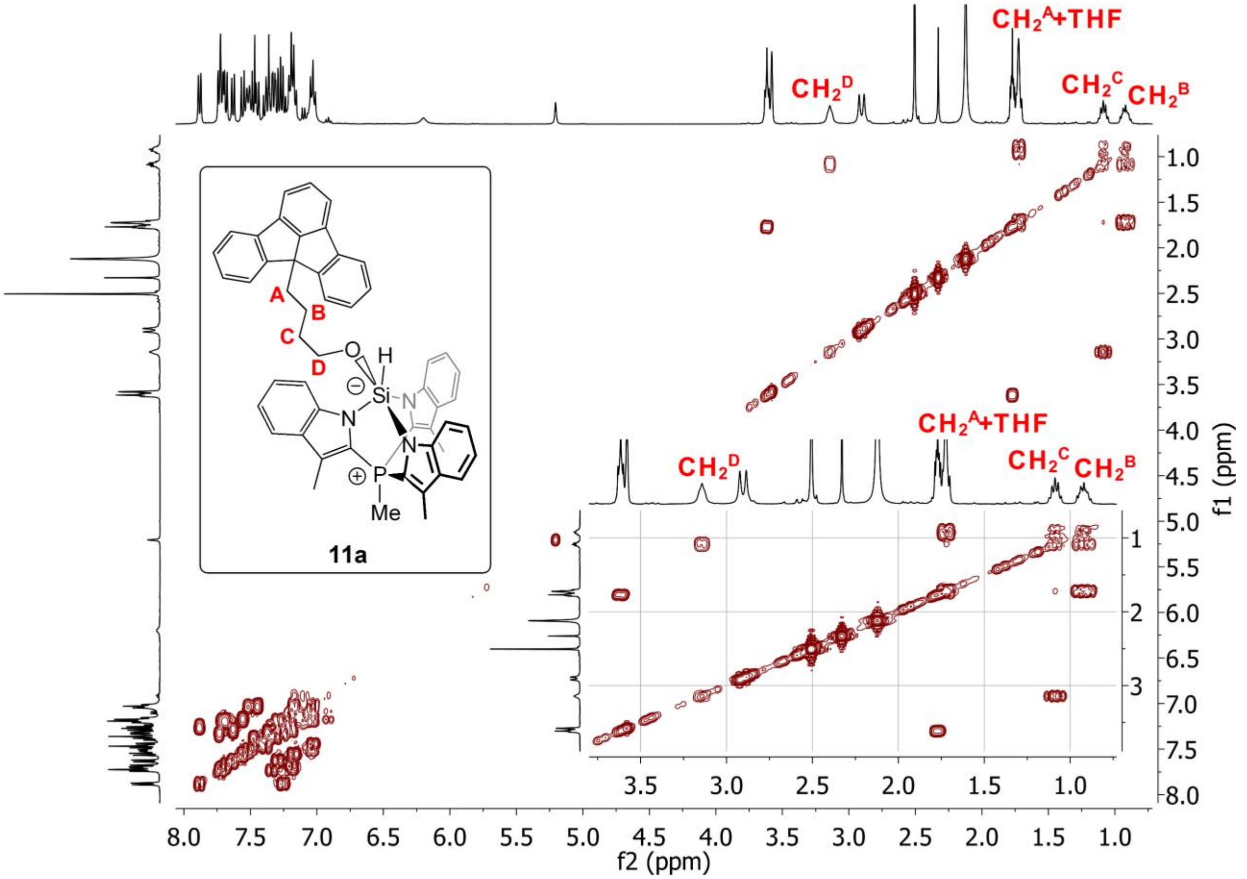

Figure S13. ^{1}H gCOSY spectrum (400 MHz) of the reaction between TSMPSi (**1**), fluoradene (**4**) and THF-h_8, recorded in THF-d_8 at 25 °C. Even though **11a** is in thermal equilibrium with the cage-opening product **11b**, only the structure of **11a** is shown for simplicity.

S5.3.3 ^{1}H 1D NOE NMR of the THF ring-opening reaction

The connection between the broadened ^{1}H signals assigned to **11a/b** is confirmed by NOE experiments (*Figure S14*). More specifically, saturation of CH_2^A methylene yields a number of expected correlations such as broad $CH_2^{B\text{-}D}$ signals, H^P doublet and some minor peaks from further along fluoradene spin system (labelled with asterisk). Similarly, upon saturation of CH_2^D protons, the excited resonances are silane Si–H^E, aromatic doublet H^N as well as methylenes $CH_2^{A\text{-}C}$. Interestingly, a correlation is also observed with an aromatic methyl group, CH_3^G, which cannot happen for the closed structure **11a** due to a large distance between the resonating protons. It can only be the case if one of indolyl arms dissociates from silicon and engages into free rotation around C–P bond, giving an open form **11b**. The occurrence of this dynamic process is also consistent with observed line-broadening in the ^{1}H NMR spectrum (see *Section S5.3.1*). On a related note, despite breaking of equivalence of the aromatic methyl groups, they still show as one signal in the ^{1}H spectrum. This apparent contradiction can be resolved if there is an additional dynamic process in **11a** that exchanges these positions (see *Section S5.3.7*).

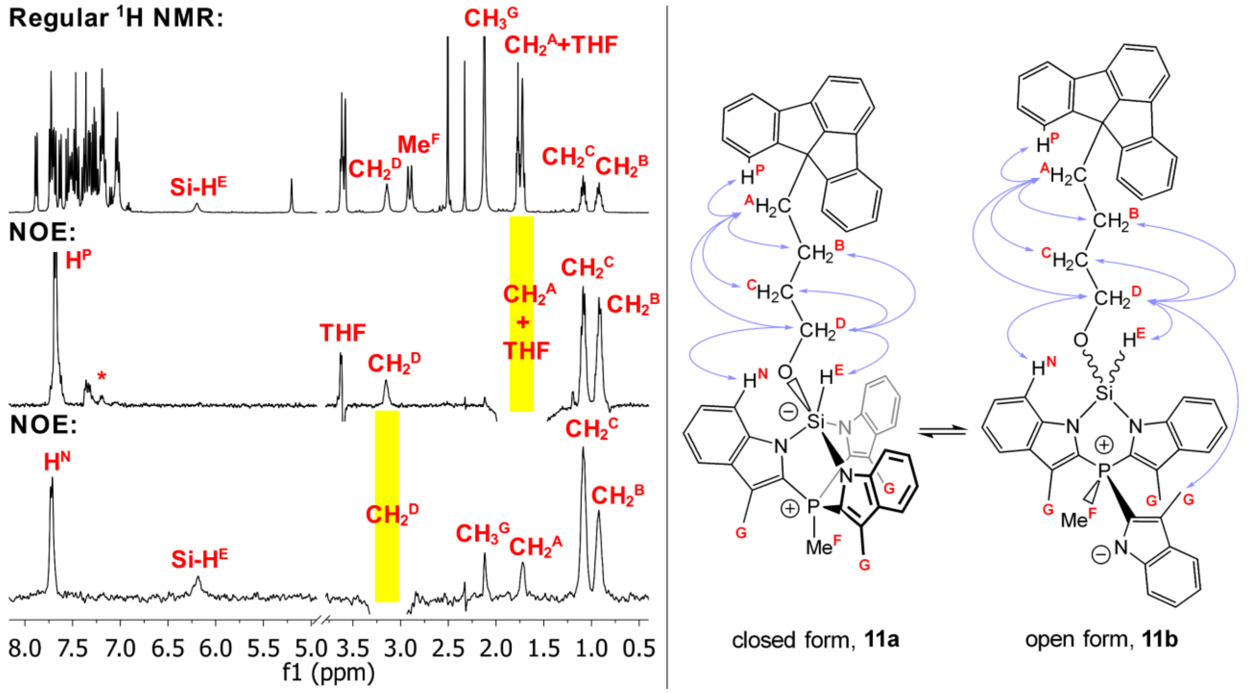

Figure S14. Left panel: ^{1}H NMR (400 MHz) and 1D NOE spectra of the reaction between TSMPSi (**1**), fluoradene (**4**) and THF-h_8, recorded in THF-d_8 at 25 °C. Assignments are shown only for the solvent and THF ring-opening product **11a/b**. Yellow rectangles show saturation windows. *Right panel*: NOE correlations mapped on the structural formulae of isomeric forms.

S5.3.4 Reactions with various fluorenes

We have tested a reaction of TSMPSi (**1**) in THF-d_8 with a number of fluorenes with pK_a^{DMSO} between 8.1 and 17.9 (*Chart S3*).

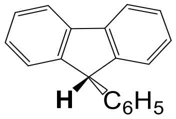

14
(pK_a^{DMSO} = 17.9)[89]

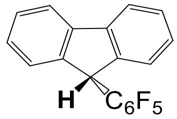

15
(pK_a^{DMSO} = 14.7)[18]

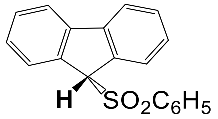

16
(pK_a^{DMSO} = 11.6)[90]

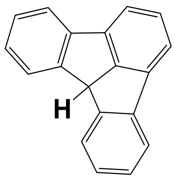

4
(pK_a^{DMSO} = 10.5)[47]

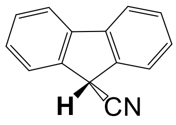

17
(pK_a^{DMSO} = 8.3)[89]

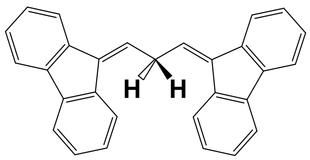

5
(pK_a^{DMSO} = 8.1)[47]

Chart S3. CH-acids used in the study. Acidic protons are shown in bold.

As shown by ^{1}H and ^{31}P NMR spectra (*Figure S15* and *Figure S16*) and regardless of pK_a^{DMSO}, all fluorenes lead to isomerization of **1** into iso-TSMPSi (**10**) (pathway **A** in *Scheme S8*). This is consistent with a catalytic nature of this transformation, which is discussed in *Section S5.2.2* in more detail. Additionally, THF ring-opening (pathway **B** in *Scheme S8*) occurs for fluorenes with pK_a^{DMSO} in the range of 8.1-11.6 (*Figure S15* and *Figure S16*). This fact suggests that the THF ring-opening is dependent on the equilibrium concentration of fluorenide anions in solution, which supports the mechanism proposed in *Scheme 3* (see main text).

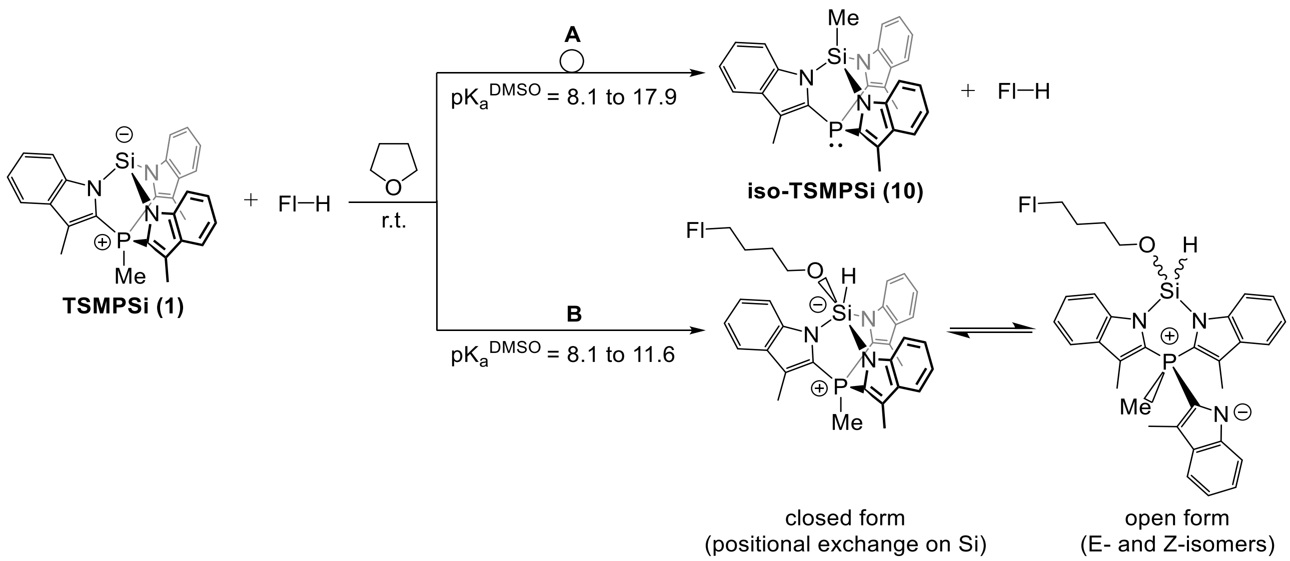

Scheme S8. Full reaction manifold of TSMPSi (**1**) with tested fluorenes.

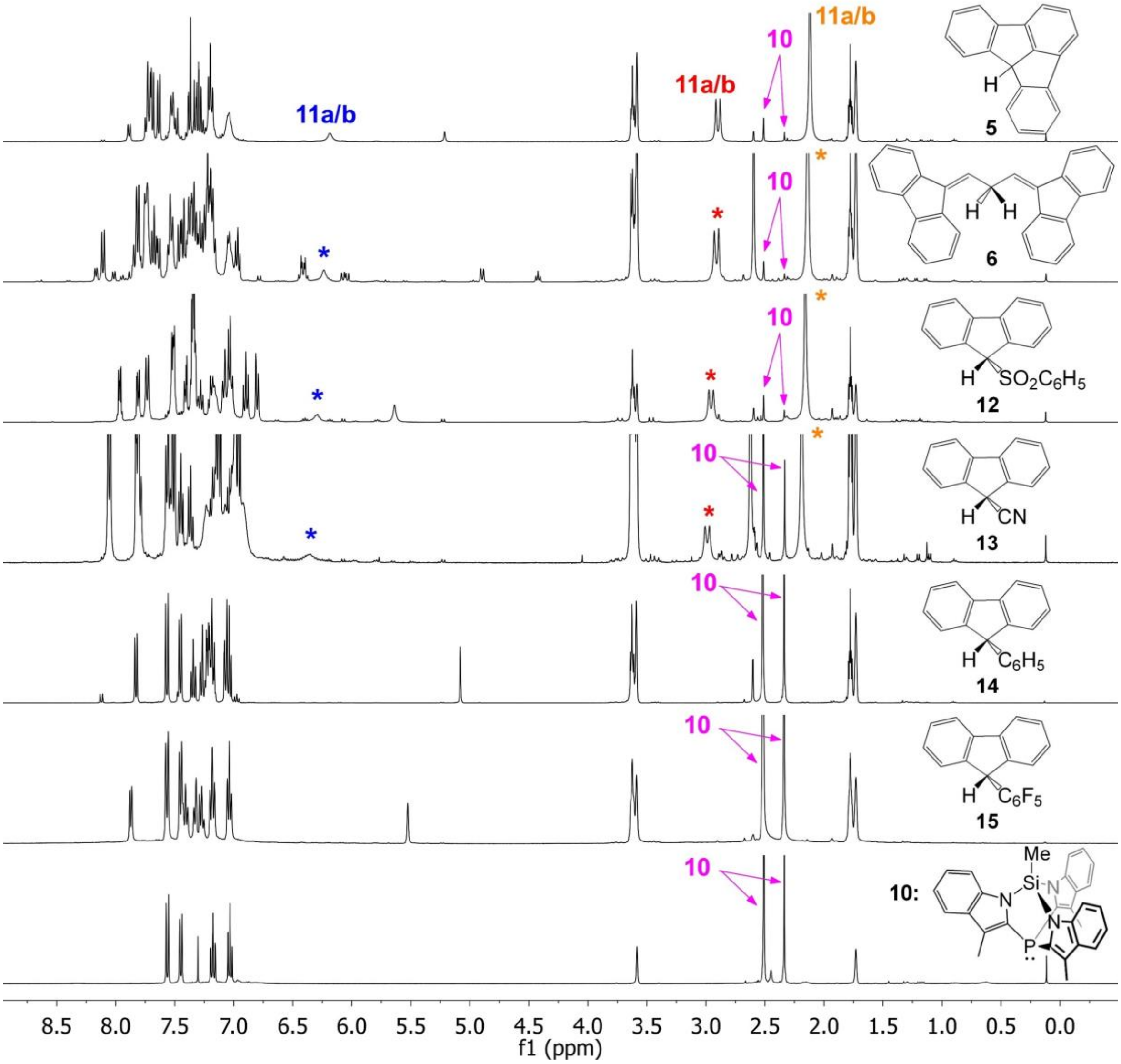

Figure S15. ^{1}H NMR (400 MHz) spectra of a reaction of TSMPSi (**1**) in THF-d_8 with a number of fluorenes at 25 °C. Used fluorenes are shown on the right, a spectrum of **10** is given for a reference. Coloured asterisks indicate peaks analogous to those of the THF ring-opening product **11a/b**.

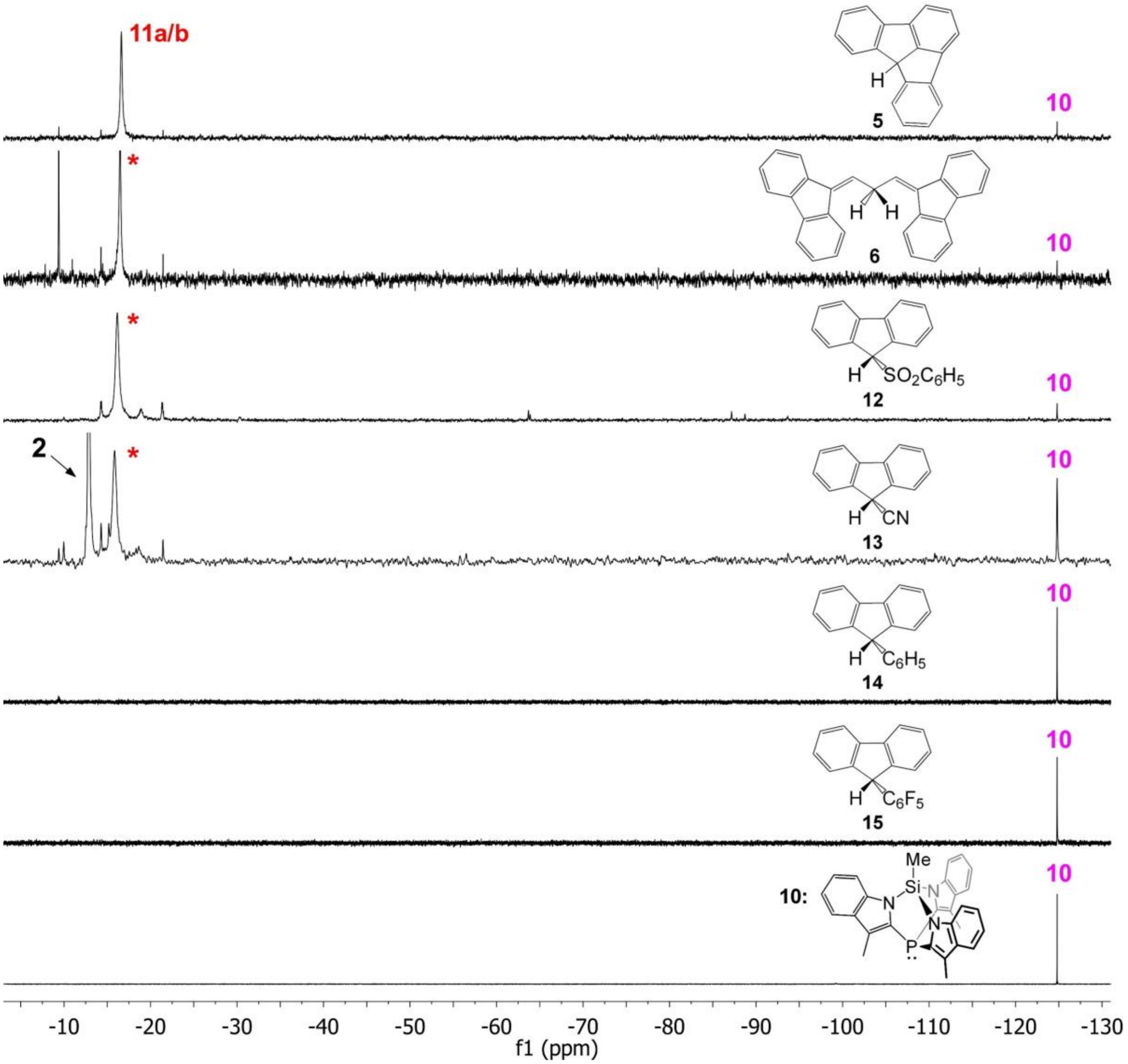

Figure S16. ^{31}P NMR (162 MHz) spectra of a reaction of TSMPSi (**1**) in THF-d_8 with a number of fluorenes at 25 °C. Used fluorenes are shown on the right, a spectrum of **10** is given for a reference. Coloured asterisks indicate peaks analogous to those of the THF ring-opening product **11a/b**.

S5.3.5 Variable temperature ^{1}H and ^{31}P NMR of the THF ring-opening reaction

The solution-state dynamics of the reaction mixture was further investigated by variable-temperature ^{1}H and ^{31}P NMR experiments (*Figure S17*). Both nuclei show notable and simultaneous changes with temperature. For instance, at 60 °C, all peaks assigned to the THF ring-opening product **11a/b** are sharp. As temperature decreases, these peaks move around and broaden, almost coalescing with the baseline between 0 and -20 °C. At lower temperature, they reappear (albeit broadened) at different chemical shifts which barely change between -60 and -89 °C.

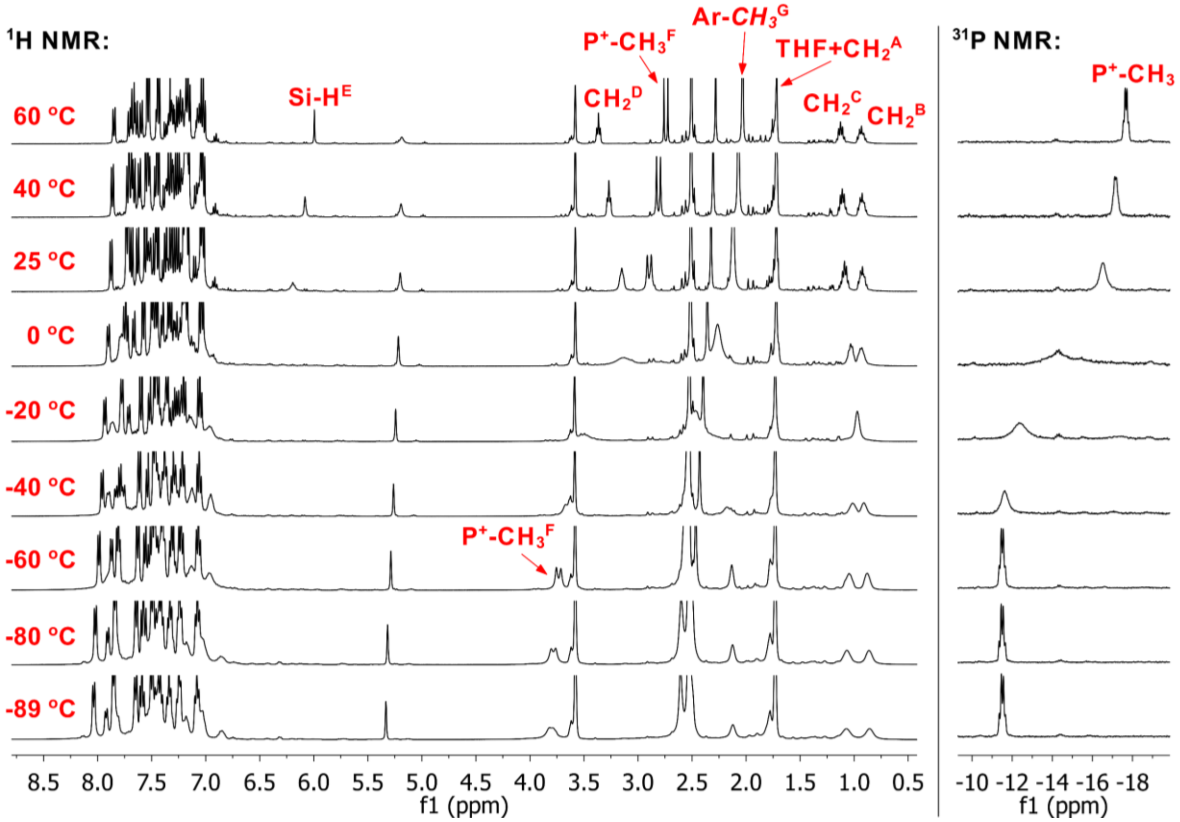

Figure S17. Variable-temperature ^{1}H (400 MHz) and ^{31}P (162 MHz) NMR spectra of the reaction between TSMPSi (**1**), fluoradene (**5**) and THF-h_8, measured in THF-d_8. All labeled peaks belong to ring-opening product **11a/b**. In ^{31}P spectra, the signal of isomer iso-TSMPSi (**10**) at ca.-125 ppm is not shown.

This behaviour is diagnostic of a dynamic equilibrium between two chemical entities in solution, which is consistent with the NOE spectra in *Section S5.3.3*. The fact that, in ^{31}P NMR spectra, there is only one quaternary phosphonium peak visible per measurement implies that equilibrium between the two forms **11a/b** is fast, and the observable chemical shift is a population-weighted average of the shifts of two individual forms. In this case, it is possible to regressively find thermodynamic parameters of the interconversion *via* temperature-dependent equilibrium constant.

An equilibrium between the low- (**11a**) and the high-temperature (**11b**) forms can be written down as follows:

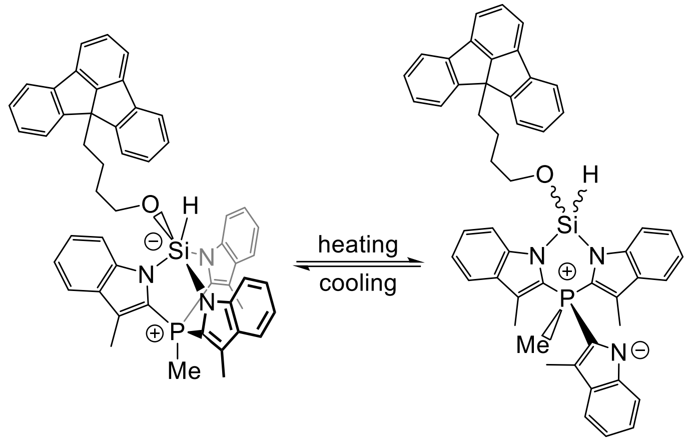

closed form, **11a**   open form, **11b**

molar fraction: *(1-x)*   *x*

$$K = \frac{x}{1 - x}$$

In this case, the observable chemical shift δ can be represented as a weighted average of individual shifts δ_A and δ_B (assuming the shifts are temperature-independent):

$$\delta = (1 - x)\delta_A + x\delta_B.$$

The equilibrium constant K can be expressed *via* δ as:

$$\delta = (1 - x)(\delta_A + K\delta_B)$$

$$\delta = \frac{\delta_A + K\delta_B}{1 + K}.$$

Assuming that $\Delta_r H$ and $\Delta_r S$ are temperature-independent, K can also be written as:

$$-RTlnK = \Delta_r H - T\Delta_r S$$

$$K = \exp\left(\frac{T\Delta_r S - \Delta_r H}{RT}\right),$$

where T is temperature, R is a gas constant, $\Delta_r H$ and $\Delta_r S$ are enthalpy and entropy of the interconversion. Thus, one can get a final expression for observed chemical shift:

$$\delta = \frac{\delta_A + \delta_B \exp\left(\frac{\Delta_r S}{R} - \frac{\Delta_r H}{RT}\right)}{1 + \exp\left(\frac{\Delta_r S}{R} - \frac{\Delta_r H}{RT}\right)} \qquad (Eq.S1)$$

However, in order to use the regression model in *Eq. S1*, one may need to know the individual chemical shifts of the low- and high-temperature forms, δ_A and δ_B respectively. Whereas for the low-temperature form it is obvious since ^{31}P shift stops evolving below -60°C, it is not clear whether the transition into an open form is complete at 60 °C. ^{31}P NMR measurement at a higher temperature would not be possible since it would boil the solvent (THF-d_8). Since there are nine data points, in addition to $\Delta_r H$ and $\Delta_r S$ of the interconversion, one can also fit chemical shift of the open form δ_B (*Figure S18*).

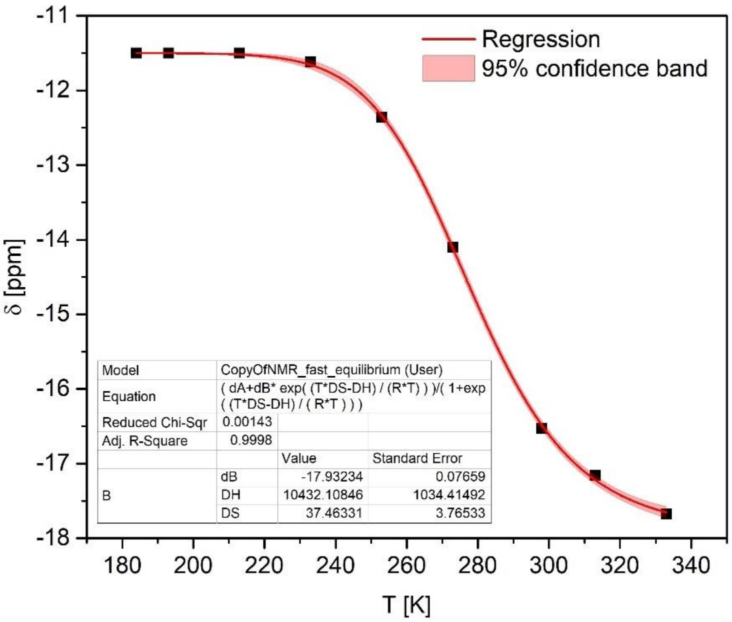

Figure S18. Regression analysis of variable-temperature ^{31}P chemical shifts.

As can be seen, the derived model adequately describes evolution of ^{31}P chemical shift with temperature. Regression analysis shows that the transition is almost complete (96%) at 60 °C (333K). The limiting ^{31}P shift δ_B is -17.9 ppm. Positive enthalpy of 10.4 kcal/mol indicates that transition into an open form is endothermic, which is consistent with breaking of a Si–N bond. Positive entropy of 37.5 cal/(mol*K) is also in line with such a process since cage-opening creates additional degrees of freedom.

S5.3.6 Variable temperature ^{29}Si NMR of the THF ring-opening reaction

A confirmation as to the nature of the breaking bond can be derived from variable-temperature ^{29}Si NMR spectra (*Figure S19*). At both -89 and 25 °C, one can observe two signals. One of them weakly depends on temperature and belongs to iso-TSMPSi (**10**), as can be shown by comparison with the spectra of the pure compound (see *Section S2.1*). The chemical shift of peak **11a/b**, on the other hand, is strongly temperature-dependent and changes from -120.2 to -74.9 ppm upon heating from -89 to 25 °C. Such a significant difference is diagnostic of a geometry change on silicon. Knowing from ^{31}P NMR spectroscopy (*Section S5.3.5*) that the transition occurs above -60 °C, one can consider -120.2 ppm at -89 °C to be the pure shift of the closed form (**11a**). This value is typical for either a penta- or hexacoordinate silicon atom.[91] Unfortunately, no ^{29}Si resonance was observed at elevated temperature (60 °C), likely due to reduced signal intensity because of the smaller population difference between nuclear magnetic energy levels at a higher temperature. However, from the analysis of temperature-dependent ^{31}P shifts in *Section S5.3.5* , it is possible to extrapolate the ^{29}Si shift to the limiting high-temperature value. Thus, at 25 °C, the transition is 78.5% complete, therefore the full transition should give -62.5 ppm, which is typical for tetrahedral silanes.[91]

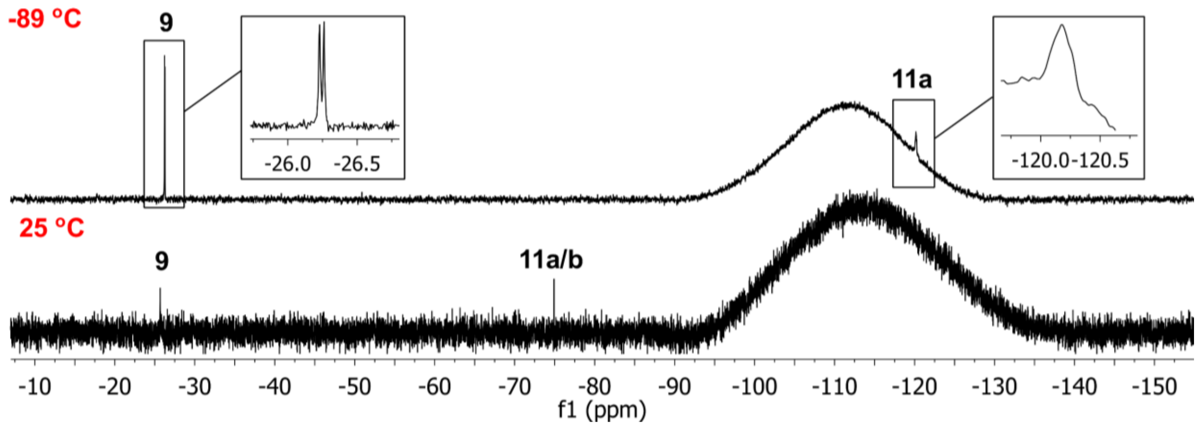

Figure S19. Variable-temperature ^{29}Si NMR (79.5 MHz) spectra of the reaction between TSMPSi (**1**), fluoradene (**4**) and THF-h_8, recorded in THF-d_8.

As for $^1J_{Si,H}$ spin-spin coupling constants, both the ^{1}H (see *Section S5.3.5*) and ^{29}Si spectra of **11a** at -89 °C show the Si–H peaks that are too broad for the coupling effects to be detected. However, the $^1J_{Si,H}$ coupling constant for **11b** was extracted by superimposing ^{1}H{^{13}C} and ^{1}H{^{29}Si} spectra at 60 °C (*Figure S20*), which gave 310.1 Hz.

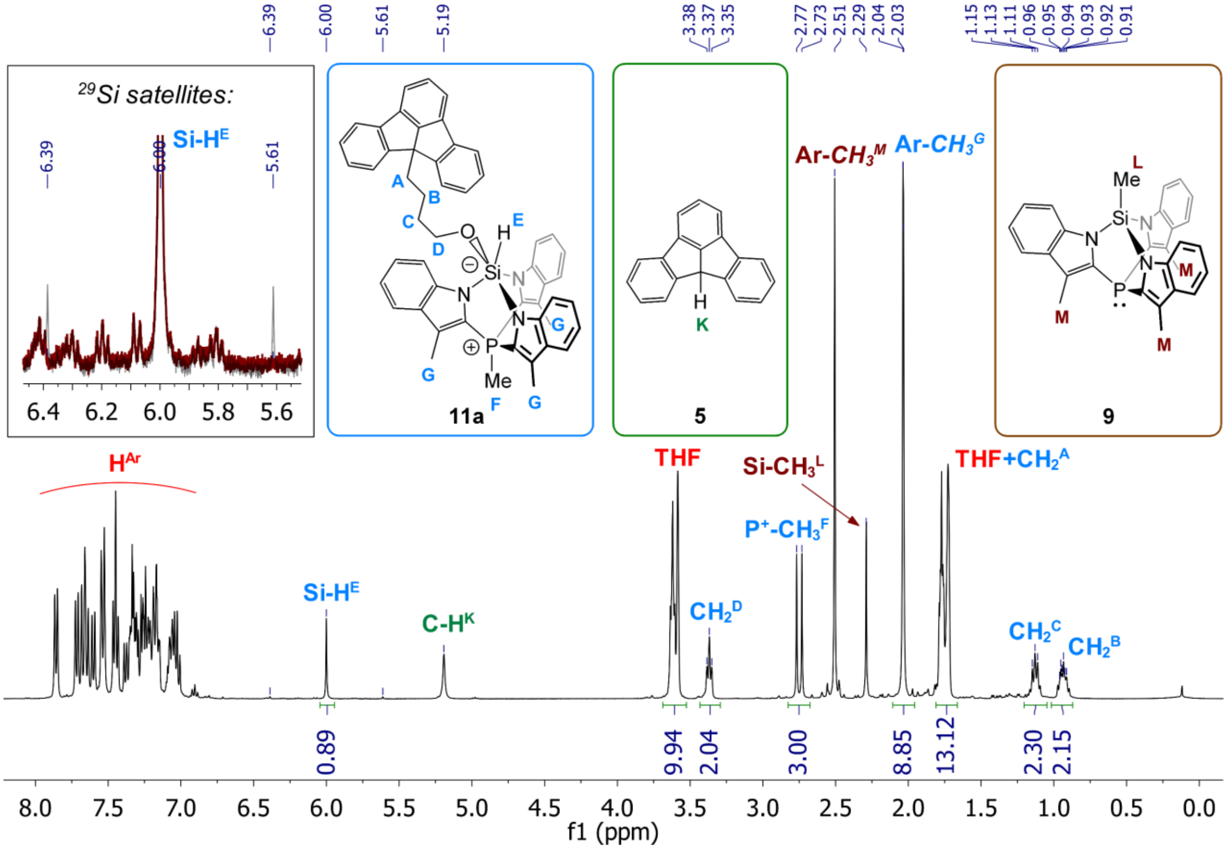

Figure S20. ^{1}H{^{13}C} NMR (400 MHz) spectrum of the reaction between TSMPSi (**1**), fluoradene (**4**) and THF-h_8, recorded in THF-d_8 at 60 °C. An insert in the upper-left corner shows an overlay of ^{1}H{^{13}C} (grey) and ^{1}H{^{29}Si} (maroon) spectra, thus allowing to identify $^1J_{Si,H}$.

In order to reinforce our assignments of temperature-dependent speciation in solution, we calculated ^{29}Si NMR chemical shifts and $^{1}J_{Si,H}$ coupling constants for possible geometries on silicon using truncated molecular models (*Table S16*). The fluoradene moiety and four methylenes were reduced to a methyl group as it was not expected to significantly influence the values of interest and greatly reduced computational time. Optimization of pentacoordinate geometries always led to a minimum with alkoxy group in an axial position of a trigonal bipyramid due to high apicophilicity of oxygen.

Table S16. Comparison of calculated and experimental assigned chemical shifts (δ) and spin-spin coupling constants (*J*) for different geometries on silicon. Experimental spin-spin coupling constant $^{1}J_{Si,H}$ is given as an absolute value. Geometry optimizations were performed in vacuum at B3LYP-GD3BJ/6-311++G** level of theory, while the NMR parameters were calculated in THF (PCM solvation) using B3LYP-GD3BJ/IGLO-III for C, H, P, Si, O atoms and 6-311++G** for Fe atoms.

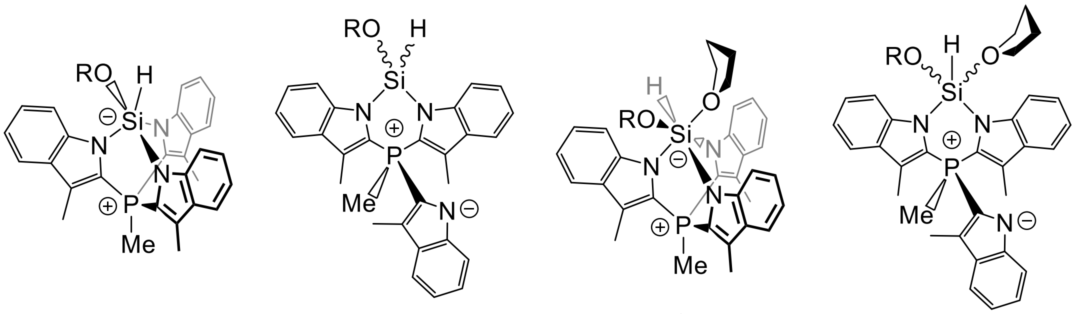

A Trigonal bipyramidal (closed form) — **B** Tetrahedral (open form) — **C** Octahedral (closed form•THF) — **D** Trigonal bipyramidal (open form•THF)

Geometry on silicon	NMR parameters	
	**R = Me (calculated, truncated model)**	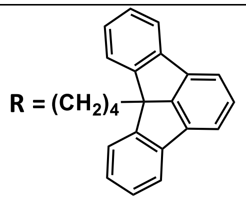 **(experimental, assigned)**
**A**: Trigonal bipyramidal (closed form)	δ(^{29}Si) = -124.9 ppm<br>$^{1}J_{Si,H}$ = -372.0 Hz	**11a**: δ(^{29}Si) = -120.2 ppm[a]
**B**: Tetrahedral (open form)	(values for E/Z geometry)<br>δ(^{29}Si) = -56.9/-57.5 ppm<br>$^{1}J_{Si,H}$ = -309.1/-333.2 Hz	**11b**: δ(^{29}Si) = -62.5 ppm[b]<br>$^{1}J_{Si,H}$ = 310.1 Hz
**C**: Octahedral (closed form·THF)	δ(^{29}Si) = -171.33 ppm<br>$^{1}J_{Si,H}$ = -357.8 Hz	-
**D**: Trigonal bipyramidal (open form·THF)	(values for E/Z geometry)<br>δ(^{29}Si) = -62.5/-57.8 ppm<br>$^{1}J_{Si,H}$ = -349.0/-358.7 Hz	-

[a] The signal is too broad for any $^{1}J_{Si,H}$-coupling to be observed.
[b] Extrapolated (see explanation in the text).

As can be seen, the observed ^{29}Si chemical shift for **11a** corresponds well to the calculated value for the closed trigonal bipyramidal geometry **A**. The situation with **11b** is somewhat more complicated: while the observed $^{1}J_{Si,H}$ coupling constant is consistent with the calculated value for the tetrahedral open form **B**, ^{29}Si chemical shifts do not allow to formally exclude the trigonal bipyramidal THF-coordinated open form **D**. Yet, the reaction entropy for a hypothetical transition from **A** into **D** should have a negative sign due to the fact that THF coordination, which reduces the number of particles in the system, should overwhelm the entropic balance compared to a smaller positive contribution of the cage opening. This contradicts the experimental Δ_rS of 37.5 cal/(mol*K) found based on variable-temperature ^{31}P NMR chemical shifts (see *Section S5.3.5*). Therefore, we assign the species observed in solution to geometries **A** and **B**.

S5.3.7 Positional exchange around a silicon atom

In order to explore the possibility of a positional exchange around a silicon atom, we undertook a relaxed potential energy surface (PES) scan in a truncated model of **11a** (**Heq1** in *Chart S4*; ΔE_{SCF} taken as 0.0 kcal/mol for a reference), which avoids complications associated with local conformational energy minima of the tetramethylene chain and saves computational time at the expense of aromatic methyl groups and fused rings. During the scan, the N^{1}^Si^OMe angle was gradually varied from 169.4° to 89.4° with all other coordinates optimized at each point. The resulting cyclic interchange of one axial and two equatorial nitrogen atoms gave the degenerate structure **Heq2**. Due to its complexity, this transformation can be represented in two different ways (*Chart S4*). Most obviously, it can be viewed as a threefold cyclic permutation – one of the five possible general permutation types derived by Muetterties[92,93] based on topological analysis (type 4 stereomutation in his own classification, or M2 stereomutation according to Lammertsma and co-workers[94]). Alternatively, it can be represented as two consecutive Berry-type deformations,[94] each having its own pivot (a substituent that does not move in the course of permutation). In the course of this permutation, the potential energy curve exhibits two maxima. A smaller maximum (92.9°, 0.2 kcal/mol) labelled with an asterisk is likely an artifact of the PES algorithm.[a] Point **TS1** (130.6°, 1.0 kcal/mol) corresponds to an apicophilic methoxy group in an equatorial position and can be regarded as an approximation of a transition state for the cyclic interchange. Its optimization using the Berny algorithm ('opt=ts' option in the Gaussian 16 program[39]) gives ΔE_{SCF} of 1.0 kcal/mol and $\Delta G^{\ddagger}$ of 2.2 kcal/mol, which is an extremely low barrier. Overall, this analysis shows that the TSMP ligand scaffold is flexible enough to allow for low-energy positional exchange on silicon. Unfortunately, we were not able to connect the optimized transition state with the degenerate minimal geometries **Heq1** and **Heq2** using the intrinsic reaction coordinate (IRC).[95] This is presumably due to the overall flatness of the potential energy hypersurface and, thus, the IRC algorithm failing to find a gradient.

---

[a] *Note on the relaxed PES scans*: the scanning algorithm in the Gaussian 16 is not a full pointwise optimization of the potential energy surface, but it gives a feeling where maxima may exist. Various discontinuities observed during the relaxed scans are known artifacts and may arise from sudden reorientations of atomic groups in the course of optimization.

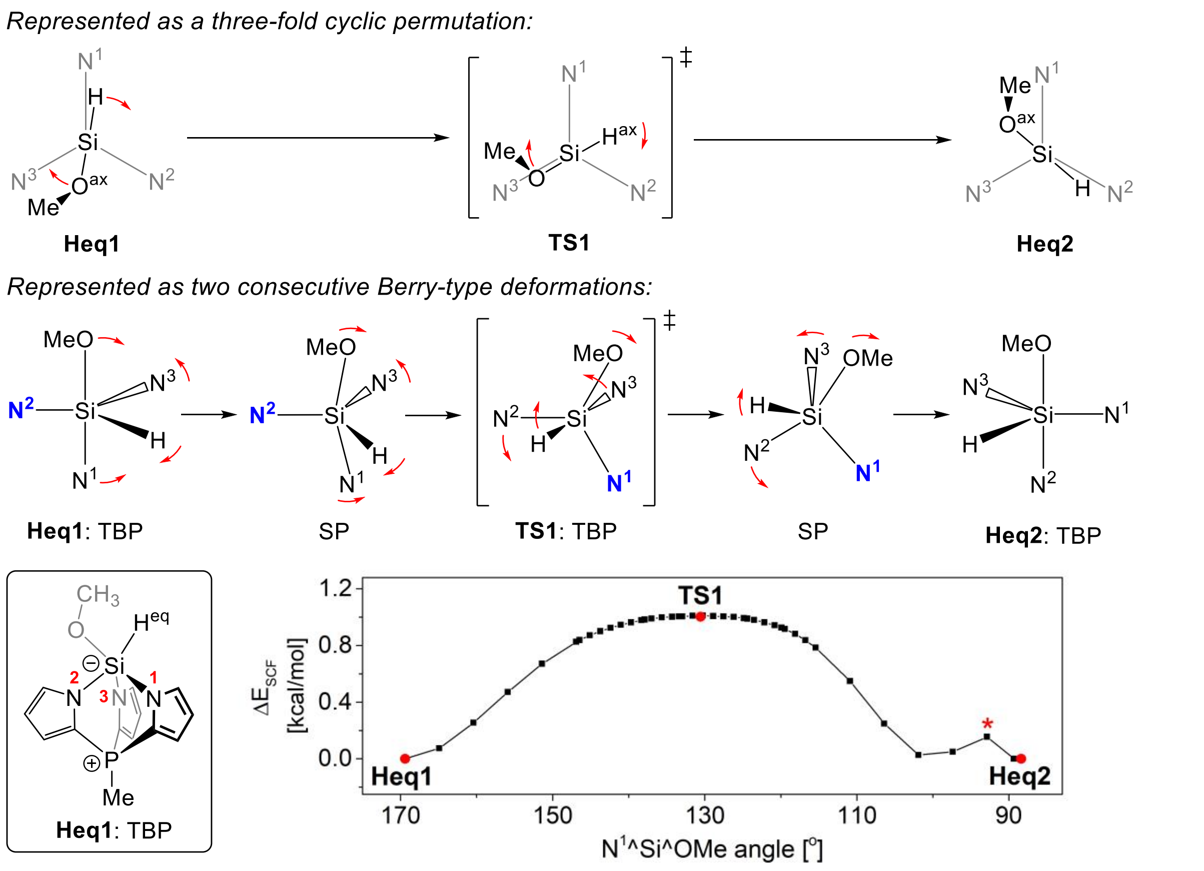

Chart S4. Computational analysis of positional exchange in a truncated model of **11a** (**Heq1** in the scheme) in a vacuum at B3LYP-GD3BJ/6-31+G* level of theory. *Top panel*: two different representations of the positional exchange; pivots for Berry-type deformations[94] are labelled in blue; TBP = trigonal bipyramid, SP = square pyramid. *Bottom-left panel*: structural formula of the truncated model. *Bottom-right panel*: potential energy in the course of the stereomutation, depending on N^1^Si^OMe.

In order to test whether the truncation of the methyl groups and fused rings affected the potential energy profile, we performed a relaxed PES scan on a more complete model of **11a** (**Heq1'** in *Chart S5*). The resulting potential energy curve bears the same extremal character as that in *Chart S4*: there are two maxima at 93.9°, 0.1 kcal/mol and 129.9°, 1.1 kcal/mol. However, the curve is less flat at the top, likely due to the presence of the aromatic methyl groups interfering with the internal rotation. Optimization of a transition state from point **TS1'** using the Berny algorithm ('opt=ts' option in the Gaussian 16 program[39]) gives ΔE_{SCF} of 1.1 kcal/mol and $\Delta G^{\ddagger}$ of 2.0 kcal/mol, which is similar to the values obtained for the simpler model in *Chart S4*. Overall, the truncation of the aromatic rings and methyl groups does not strongly affect the potential energy profile while significantly reducing the computational time. This made us confident that we could use this model for a relaxed 2D PES scan (see *Section S5.3.8*).

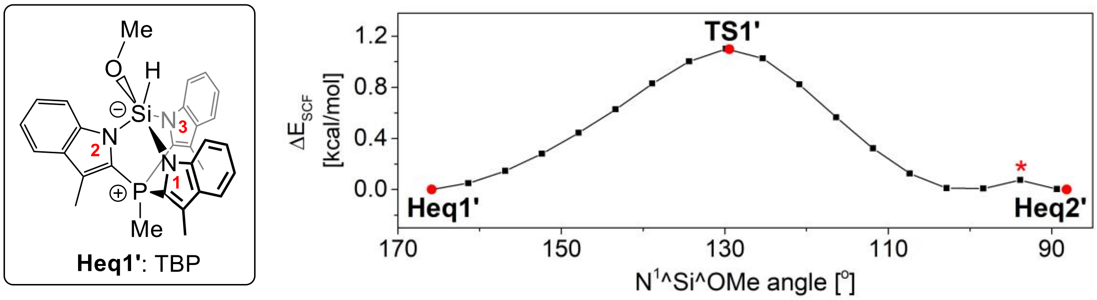

Chart S5. Relaxed PES scan in a less truncated model of **11a** (**Heq1'** in the scheme) in a vacuum at B3LYP-GD3BJ/6-31+G* level of theory. TBP = trigonal bipyramid.

It should be noted that the calculated barrier of ~2 kcal/mol is likely an underestimation for the experimental system. One should remember that the barrier was calculated for a truncated model in the gas phase. The value for the real molecule **11a** in solution is expected to be higher due to the larger mass and conformational constraints of the aryltetramethylene chain as opposed to a methoxy group. Besides, for precise treatment, one might also need to consider solvation effects in THF.

S5.3.8 Relaxed 2D PES scan

In order to investigate the origins of a *Z*-isomer, **11b-*Z***, we undertook a two-dimensional relaxed PES scan in a truncated model of **11a** (**Heq1** in *Chart S4*). As in the one-dimensional scans in *Section S5.3.7*, the N^1^Si^OMe angle was gradually varied as to correspond to a transition between degenerate minimum energy geometries **Heq1** and **Heq2** (*Scheme S9*). Additionally, for every angle, the distance between the silicon atom and the N^3 nitrogen atom, dissociation of which leads to a *Z*-isomer, was incrementally increased. The results are shown in *Figure S21*. The two-dimensional PES scan shows several stationary points that correspond to either minimum energy geometries (**Heq1**, **Heq2** and ***Z*-form**) or transition states (**TS1** and **TS2**). These were optimized separately, and their energies are shown in *Scheme S9*.

Now, owing to the principle of microscopic reversibility, let us change the perspective and consider the reverse reaction – the closure of the ***Z*-form**. It appears that the corresponding **TS2** is directly followed by **TS1** with no energy minimum in-between. Such a disposition is known[96,97] to give rise to a post-transition-state bifurcation where a reaction path splits into two branches leading to the alternative products, **Heq1** and **Heq2** in our case. The branching occurs at the valley-ridge inflection point (**VRI**) where the PES valley changes into a dynamically-unstable ridge.[98] This reaction type cannot be described as stepwise or concerted and is instead referred to as a two-step-no-intermediate mechanism.[99]

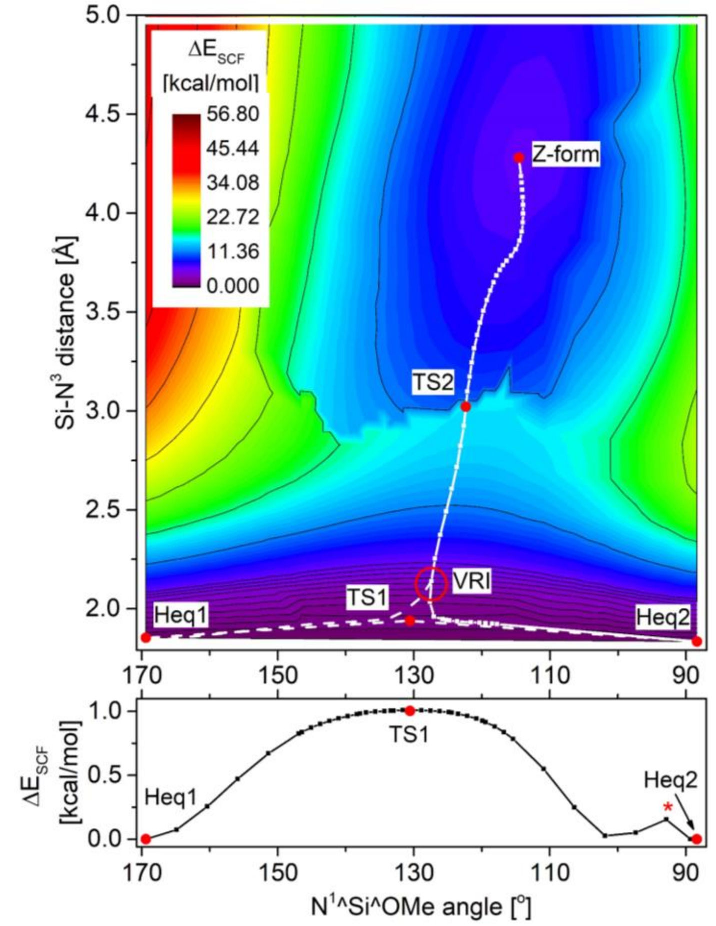

Figure S21. Two- (*top plot*) and one-dimensional (*bottom plot*; adapted from *Chart S4*) relaxed PES scans at B3LYP-GD3BJ/6-31+G* level of theory in vacuum. Stationary points as well as the hypothetical **VRI** region are indicated explicitly. Reaction paths are shown in white. Solid line was used for the paths derived by the IRC with the separate IRC steps marked along. Dashed line was used for the hypothetical paths. In the two-dimensional PES scan, contour lines are shown for every 5.68 kcal/mol increment, except for the lowest ten, which are shown for every 0.568 kcal/mol.

In order to confirm the connection between the transition states and the corresponding PES minima, as well as to visualize the reaction path, we resorted to IRC.[95] As discussed in *Section S5.3.7*, we could not connect **Heq1** and **Heq2** *via* **TS1.** Presumably, due to the flatness of PES between these points, and, therefore, the IRC algorithm failing to find a gradient. While we were able to easily find a path between **TS2** and ***Z*-form**, an IRC calculation from **TS2** led to **Heq2** only (*Figure S22*). It is not surprising since, mathematically, the IRC path is a unique solution of an autonomous system of differential equations and, therefore, no branching can occur before reaching the next stationary point.[100,101] Oftentimes, in such situations, IRC simply connects **TS1** and **TS2** giving a dynamically unstable reaction path.[97] This is not the case for our system because of another fundamental property of IRC: while on the intrinsic reaction path, a molecular system can never loose symmetry. Whereas **TS2** and **Heq2** are asymmetric (C_1 group), **TS1** possesses a mirror plane (C_s group), therefore, the path connects the former two. Precisely locating the point where the branching towards **Heq1** occurs, i.e. **VRI**, is a challenging problem.[96,97] The position of a valley-ridge inflection point, in general, depends

on the chosen coordinate system.[102] For the purpose of our discussion, the exact location of **VRI** plays a minor role, therefore we did not expend effort for finding it. Instead, we make an educated guess about the region where it should be found based on the shape of the PES. As mentioned above, a **VRI** appears when two transition states follow one another with no energy minima in-between. Along the reaction path from **TS2** to **TS1**, the curvature of the PES changes from negative to positive. Since **TS2** is much more energetic than **TS1** (ΔE_{SCF} of 14.8 *vs.* 1.0 kcal/mol), the contribution of its curvature along the path is also larger, shifting the inflection point (**VRI**) closer to **TS1**. Indeed, a visual inspection of the two-dimensional PES scan in *Figure S21* reveals a flat region next to **TS1**. This is where the intrinsic reaction path between **TS2** and **Heq2** should split off towards **Heq1**. Hence, we draw an approximate subpath that connects the **VRI** region and **Heq1**.

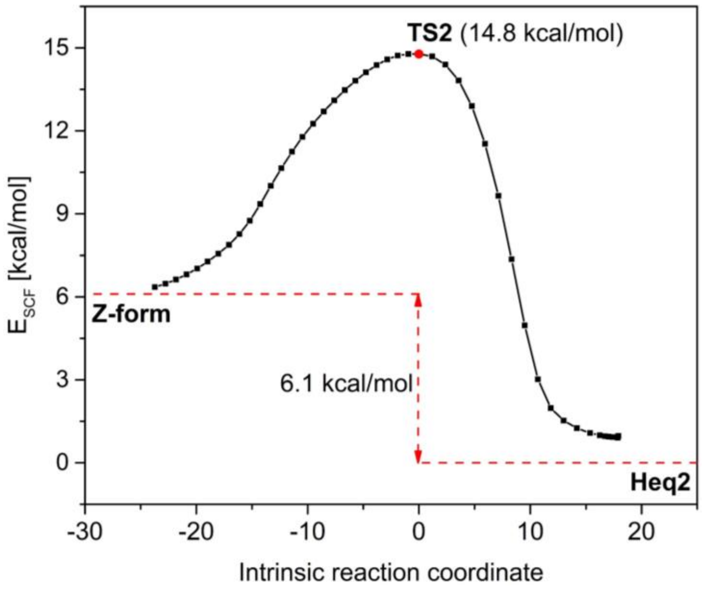

Figure S22. IRC path that connects ***Z*-form** and **Heq2** via **TS2.** Geometry optimization at the end of each branch leads directly to the respective forms. Level of theory: B3LYP-GD3BJ/6-31+G*.

Now, let us look at interpretation of the points and paths on PES in terms of molecular geometry (*Scheme S9*). With decreasing the N^1^Si^OMe angle, roughly trigonal bipyramidal **Heq1** (ΔG of 0.0 kcal/mol taken as a reference) can undergo a three-fold cyclic permutation. The same in reverse applies for **Heq2**. The transition state for this process is very low-lying (**TS1**; $\Delta G^{\ddagger}$ of 2.2 kcal/mol). However, shortly before reaching **TS1**, the system can take another path where the Si-N^3 bond starts dissociating. At the initial stages of this process, the molecular geometry closely resembles that of **TS1**. Whether the reaction path starts at **Heq1** or **Heq2**, it anyway ends up at **VRI**, which is the point of convergence of the two branches. There, the molecular system has a rough mirror symmetry with the O-Si-H, N^3 and P atoms approximately in the same plane. From there, *via* further elongation of the Si-N^3 bond, the system proceeds to the asymmetric **TS2** ($\Delta G^{\ddagger}$ of 12.8 kcal/mol) and to the final product, ***Z*-form** (ΔG of 3.1 kcal/mol). For comparison, a transition barrier from **Heq1** to an ***E*-form**

(not shown in *Scheme S9*) has $\Delta G^{\ddagger}$ of 9.6 kcal/mol,[a] whereas the form itself has ΔG of 4.5 kcal/mol. Overall, this implies that both geometric isomers of the open form are thermally accessible at room temperature. Projecting these conclusions onto the experimental system can explain our observations.

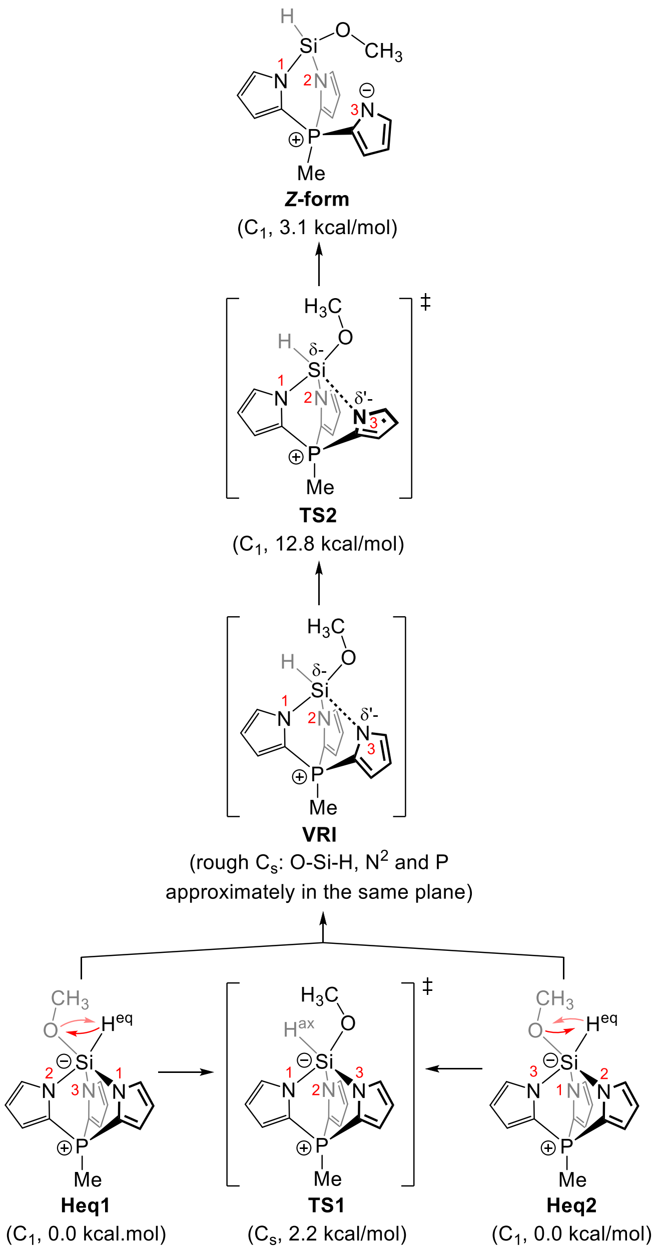

Scheme S9. Interpretation of the points and paths in relaxed PES scans in *Figure S21* in terms of molecular geometry at B3LYP-GD3BJ/6-31+G* level of theory in vacuum. Where applicable, symmetry point group and ΔG are indicated in parenthesis.

---

[a] The corresponding transition state was located using QST3 algorithm in Gaussian 16.[39] Due to the high curvature and overall flatness of the corresponding PES, IRC algorithm fails to connect the transition state with **Heq1** and ***E*-form**. Still, minor manual displacement of the transition state along the imaginary normal mode followed by the geometry optimization yields both **Heq1** and ***E*-form**. This indirectly confirms that the transition state in question lies on the minimal energy path between the two geometries.

S6 Literature references

(1) Fürstner, A. Potassium-Graphite Laminate. In *Encyclopedia of Reagents for Organic Synthesis*; John Wiley & Sons, Ltd: Chichester, UK, 2001. https://doi.org/10.1002/047084289X.rp217.

(2) Ghadwal, R. S.; Roesky, H. W.; Merkel, S.; Henn, J.; Stalke, D. Lewis Base Stabilized Dichlorosilylene. *Angew. Chemie Int. Ed.* **2009**, *48* (31), 5683–5686. https://doi.org/10.1002/anie.200901766.

(3) Yakelis, N. A.; Bergman, R. G. Safe Preparation and Purification of Sodium Tetrakis[(3,5-Trifluoromethyl)Phenyl]Borate ($NaBArF_{24}$): Reliable and Sensitive Analysis of Water in Solutions of Fluorinated Tetraarylborates. *Organometallics* **2005**, *24* (14), 3579–3581. https://doi.org/10.1021/om0501428.

(4) Brookhart, M.; Grant, B.; Volpe, A. F. $[(3,5\text{-}(CF_3)_2C_6H_3)_4B]^-[H(OEt_2)_2]^+$: A Convenient Reagent for Generation and Stabilization of Cationic, Highly Electrophilic Organometallic Complexes. *Organometallics* **1992**, *11* (11), 3920–3922. https://doi.org/10.1021/om00059a071.

(5) Tretiakov, S.; Damen, J. A. M.; Lutz, M.; Moret, M.-E. A Dianionic C_3-Symmetric Scorpionate: Synthesis and Coordination Chemistry. *Dalt. Trans.* **2020**, *49* (39), 13549–13556. https://doi.org/10.1039/D0DT02601H.

(6) Fulmer, G. R.; Miller, A. J. M.; Sherden, N. H.; Gottlieb, H. E.; Nudelman, A.; Stoltz, B. M.; Bercaw, J. E.; Goldberg, K. I. NMR Chemical Shifts of Trace Impurities: Common Laboratory Solvents, Organics, and Gases in Deuterated Solvents Relevant to the Organometallic Chemist. *Organometallics* **2010**, *29* (9), 2176–2179. https://doi.org/10.1021/om100106e.

(7) Kupče, E.; Freeman, R. Fast Multidimensional NMR by Polarization Sharing. *Magn. Reson. Chem.* **2007**, *45* (1), 2–4. https://doi.org/10.1002/mrc.1931.

(8) VnmrJ 4.2, Revision A. Agilent Technologies: Santa Clara, USA 2014.

(9) MestReNova v.9.0.1-13254. Mestrelab Research S.L. 2014.

(10) Barnard, T. S.; Mason, M. R. Hindered Axial−Equatorial Carbonyl Exchange in an $Fe(CO)_4(PR_3)$ Complex of a Rigid Bicyclic Phosphine. *Inorg. Chem.* **2001**, *40* (19), 5001–5009. https://doi.org/10.1021/ic001372b.

(11) Witteman, L.; van Beek, C. B.; van Veenhuizen, O. N.; Lutz, M.; Moret, M.-E. Synthesis and Complexation of a Free Germanide Bearing a Tridentate *N*-Heterocyclic Substituent. *Organometallics* **2019**, *38* (2), 231–239. https://doi.org/10.1021/acs.organomet.8b00630.

(12) Hailwood, A. J.; Robinson, R. A Reaction of Fluorene. *J. Chem. Soc.* **1932**, 1292–1295. https://doi.org/10.1039/jr9320001292.

(13) Sugawara, T.; Nakashima, N.; Yoshihara, K.; Iwamura, H. Low-Temperature and Time-Resolved Absorption Spectral Studies on the Sp- and Ap-2-(9-Fluorenyl)Phenylnitrenes Generated from 1-Azatriptycene and 2-(9-Fluorenyl)Phenyl Azide. *J. Am. Chem. Soc.* **1983**, *105* (4), 858–862. https://doi.org/10.1021/ja00342a036.

(14) Rapoport, H.; Smolinsky, G. Fluoradene. *J. Am. Chem. Soc.* **1960**, *82* (4), 934–941. https://doi.org/10.1021/ja01489a038.

(15) Gannon, S. M.; Krause, J. G. Phase-Transfer Permanganate Oxidation of Unfunctionalized Benzylic Positions. *Synthesis (Stuttg).* **1987**, *1987* (10), 915–917. https://doi.org/10.1055/s-1987-28121.

(16) Kuhn, R.; Fischer, H.; Neugebauer, F. A.; Fischer, H. Über Hochacide Kohlenwasserstoffe. *Justus Liebigs Ann. Chem.* **1962**, *654* (1), 64–81. https://doi.org/10.1002/jlac.19626540109.

(17) Vougioukalakis, G. C.; Roubelakis, M. M.; Orfanopoulos, M. Radical Reactivity of Aza[60]Fullerene: Preparation of Monoadducts and Limitations. *J. Org. Chem.* **2010**, *75* (12), 4124–4130. https://doi.org/10.1021/jo100277v.

(18) Bordwell, F. G.; Branca, J. C.; Bares, J. E.; Filler, R. Enhancement of the Equilibrium Acidities of Carbon Acids by Polyfluoroaryl Substituents. *J. Org. Chem.* **1988**, *53* (4), 780–782. https://doi.org/10.1021/jo00239a016.

(19) Bavin, P. M. G. ALIPHATIC CHEMISTRY OF FLUORENE:PART IV. PREPARATION AND ALKYLATION OF SOME SULPHIDES AND SULPHONES. *Can. J. Chem.* **1960**, *38* (6), 917–922. https://doi.org/10.1139/v60-129.

(20) Jakab, G.; Tancon, C.; Zhang, Z.; Lippert, K. M.; Schreiner, P. R. (Thio)Urea Organocatalyst Equilibrium Acidities in DMSO. *Org. Lett.* **2012**, *14* (7), 1724–1727. https://doi.org/10.1021/ol300307c.

(21) Schreurs, A. M. M.; Xian, X.; Kroon-Batenburg, L. M. J. EVAL15 : A Diffraction Data Integration Method Based on Ab Initio Predicted Profiles. *J. Appl. Crystallogr.* **2010**, *43* (1), 70–82. https://doi.org/10.1107/S0021889809043234.

(22) Sheldrick, G. M. SADABS. Universität Göttingen, Germany 2014.

(23) Sheldrick, G. M. TWINABS. Universität Göttingen, Germany 2014.

(24) Sheldrick, G. M. SHELXT – Integrated Space-Group and Crystal-Structure Determination. *Acta Crystallogr. Sect. A Found. Adv.* **2015**, *71* (1), 3–8. https://doi.org/10.1107/S2053273314026370.

(25) Sheldrick, G. M. Crystal Structure Refinement with SHELXL. *Acta Crystallogr. Sect. C Struct. Chem.* **2015**, *71* (1), 3–8. https://doi.org/10.1107/S2053229614024218.

(26) Herbst-Irmer, R.; Sheldrick, G. M. Refinement of Twinned Structures with SHELXL97. *Acta Crystallogr. Sect. B Struct. Sci.* **1998**, *54* (4), 443–449. https://doi.org/10.1107/S0108768197018454.

(27) Spek, A. L. Structure Validation in Chemical Crystallography. *Acta Crystallogr. Sect. D Biol. Crystallogr.* **2009**, *65* (2), 148–155. https://doi.org/10.1107/S090744490804362X.

(28) Spek, A. L. PLATON SQUEEZE: A Tool for the Calculation of the Disordered Solvent Contribution to the Calculated Structure Factors. *Acta Crystallogr. Sect. C Struct. Chem.* **2015**, *71*

(1), 9–18. https://doi.org/10.1107/S2053229614024929.

(29) Müller, J.; Heinl, S.; Schwarzmaier, C.; Balázs, G.; Keilwerth, M.; Meyer, K.; Scheer, M. Rearrangement of a P_4 Butterfly Complex-The Formation of a Homoleptic Phosphorus-Iron Sandwich Complex. *Angew. Chemie Int. Ed.* **2017**, *56* (25), 7312–7317. https://doi.org/10.1002/anie.201703175.

(30) Langer, R.; Bönisch, F.; Maser, L.; Pietzonka, C.; Vondung, L.; Zimmermann, T. P. Substitutional Lability of Diphosphine Ligands in Tetrahedral Iron(II) Chloro Complexes. *Eur. J. Inorg. Chem.* **2015**, *2015* (1), 141–148. https://doi.org/10.1002/ejic.201402859.

(31) Benito-Garagorri, D.; Kirchner, K.; Mereiter, K. Tetraphenylphosphonium Acetonitriletribromoferrate(II). *Acta Crystallogr. Sect. E Struct. Reports Online* **2006**, *E62*, m1136–m1138. https://doi.org/10.1107/S1600536806014504.

(32) LePichon, L.; Stephan, D. W.; Gao, X.; Wang, Q. Iron Phosphinimide and Phosphinimine Complexes: Catalyst Precursors for Ethylene Polymerization. *Organometallics* **2002**, *21* (7), 1362–1366. https://doi.org/10.1021/om011041m.

(33) Chard, E. F.; Thompson, J. R.; Dawe, L. N.; Kozak, C. M. Synthesis and Structure of Iron(III) Complexes of Amine-Bis(Phenolate) Ligands. *Can. J. Chem.* **2014**, *92* (8), 758–764. https://doi.org/10.1139/cjc-2014-0043.

(34) Holzhacker, C.; Stöger, B.; Carvalho, M. D.; Ferreira, L. P.; Pittenauer, E.; Allmaier, G.; Veiros, L. F.; Realista, S.; Gil, A.; Calhorda, M. J.; Müller, D.; Kirchner, K. Synthesis and Reactivity of TADDOL-Based Chiral Fe(II) PNP Pincer Complexes-Solution Equilibria between K^2P,N- and K^3P,N,P-Bound PNP Pincer Ligands. *Dalt. Trans.* **2015**, *44* (29), 13071–13086. https://doi.org/10.1039/C5DT00832H.

(35) Kiernicki, J. J.; Norwine, E. E.; Zeller, M.; Szymczak, N. K. Tetrahedral Iron Featuring an Appended Lewis Acid: Distinct Pathways for the Reduction of Hydroxylamine and Hydrazine. *Chem. Commun.* **2019**, *55* (79), 11896–11899. https://doi.org/10.1039/C9CC05720J.

(36) Govor, E. V.; Sanakis, Y.; Raptis, R. G. Synthesis of Pyrazole (Hemi)Aminals via the Cleavage of Saturated Aliphatic Ether C–O Bonds in the Presence of Ferric Halides. *New J. Chem.* **2017**, *41* (6), 2220–2223. https://doi.org/10.1039/C6NJ03822K.

(37) Witteman, L.; Evers, T.; Lutz, M.; Moret, M.-E. A Free Silanide from Nucleophilic Substitution at Silicon(II). *Chem. Eur. J.* **2018**, *24* (47), 12236–12240. https://doi.org/10.1002/chem.201801435.

(38) Weinhold, F.; Landis, C. R. *Valency and Bonding: A Natural Donor-Acceptor Perspective*, 1st ed.; Cambridge University Press: Cambridge, 2005. https://doi.org/10.1017/CBO9780511614569.

(39) Frisch, M. J.; Trucks, G. W.; Schlegel, H. B.; Scuseria, G. E.; Robb, M. A.; Cheeseman, J. R.; Scalmani, G.; Barone, V.; Petersson, G. A.; Nakatsuji, H.; Li, X.; Caricato, M.; Marenich, A. V.; Bloino, J.; Janesko, B. G.; Gomperts, R.; Mennucci, B.; Hratchian, H. P.; Ortiz, J. V.; Izmaylov, A. F.; Sonnenberg, J. L.; Williams-Young, D.; Ding, F.; Lipparini, F.; Egidi, F.;

Goings, J.; Peng, B.; Petrone, A.; Henderson, T.; Ranasinghe, D.; Zakrzewski, V. G.; Gao, J.; Rega, N.; Zheng, G.; Liang, W.; Hada, M.; Ehara, M.; Toyota, K.; Fukuda, R.; Hasegawa, J.; Ishida, M.; Nakajima, T.; Honda, Y.; Kitao, O.; Nakai, H.; Vreven, T.; Throssell, K.; Montgomery Jr., J. A.; Peralta, J. E.; Ogliaro, F.; Bearpark, M. J.; Heyd, J. J.; Brothers, E. N.; Kudin, K. N.; Staroverov, V. N.; Keith, T. A.; Kobayashi, R.; Normand, J.; Raghavachari, K.; Rendell, A. P.; Burant, J. C.; Iyengar, S. S.; Tomasi, J.; Cossi, M.; Millam, J. M.; Klene, M.; Adamo, C.; Cammi, R.; Ochterski, J. W.; Martin, R. L.; Morokuma, K.; Farkas, O.; Foresman, J. B.; Fox, D. J. Gaussian 16, Revision A.03. Gaussian, Inc.: Wallingford CT 2016.

(40) Ding, F.; Smith, J. M.; Wang, H. First-Principles Calculation of pK_a Values for Organic Acids in Nonaqueous Solution. *J. Org. Chem.* **2009**, *74* (7), 2679–2691. https://doi.org/10.1021/jo802641r.

(41) Fu, Y.; Liu, L.; Li, R. Q.; Liu, R.; Guo, Q. X. First-Principle Predictions of Absolute pK_a's of Organic Acids in Dimethyl Sulfoxide Solution. *J. Am. Chem. Soc.* **2004**, *126* (3), 814–822. https://doi.org/10.1021/ja0378097.

(42) Gusev, D. G. Donor Properties of a Series of Two-Electron Ligands. *Organometallics* **2009**, *28* (3), 763–770. https://doi.org/10.1021/om800933x.

(43) Glendening, E. D.; Badenhoop, J. K.; Reed, A. E.; Carpenter, J. E.; Bohmann, J. A.; Morales, C. M.; Landis, C. R.; Weinhold, F. NBO 6.0. Theoretical Chemistry Institute, University of Wisconsin: Madison, WI 2013.

(44) Humphrey, W.; Dalke, A.; Schulten, K. VMD: Visual Molecular Dynamics. *J. Mol. Graph.* **1996**, *14* (1), 33–38. https://doi.org/10.1016/0263-7855(96)00018-5.

(45) Stone, J. E. An Efficient Library for Parallel Ray Tracing and Animation, University of Missouri-Rolla, 1998.

(46) Stone, J. E.; Vandivort, K. L.; Schulten, K. GPU-Accelerated Molecular Visualization on Petascale Supercomputing Platforms. In *Proceedings of the 8th International Workshop on Ultrascale Visualization - UltraVis '13*; ACM Press: New York, New York, USA, 2013; pp 1–8. https://doi.org/10.1145/2535571.2535595.

(47) Ritchie, C. D. Acidity in Nonaqueous Solvents. VII. Proton Transfers in Dipolar Aprotic Solvents. 5. Solvation and Geometric Factors in the Rates of Proton Transfer Reactions. *J. Am. Chem. Soc.* **1969**, *91* (24), 6749–6753. https://doi.org/10.1021/ja01052a036.

(48) Kuhn, R.; Rewicki, D. Die pK-Werte Doppelbindungsisomerer Kohlenwasserstoffe Mit Gemeinsamem Carbanion. *Tetrahedron Lett.* **1965**, *6* (39), 3513–3522. https://doi.org/10.1016/S0040-4039(01)89335-7.

(49) Linstrom, P. J.; Mallard, W. G., E. NIST Standard Reference Database 69, October 2018 Release http://webbook.nist.gov/chemistry/ (accessed May 1, 2019). https://doi.org/10.18434/T4D303.

(50) Anslyn, E. V.; Dougherty, D. A. *Modern Physical Organic Chemistry*; University Science Books: Sausalito, United States, 2011.

(51) Tolman, C. A. Steric Effects of Phosphorus Ligands in Organometallic Chemistry and Homogeneous Catalysis. *Chem. Rev.* **1977**, *77* (3), 313–348. https://doi.org/10.1021/cr60307a002.

(52) Strohmeier, W.; Guttenberger, J. F. S-Haltige Verbindungen Als Liganden in Photochemisch Hergestellten Derivaten Des Cyclopentadienylmangantricarbonyls. *Chem. Ber.* **1964**, *97* (7), 1871–1876. https://doi.org/10.1002/cber.19640970714.

(53) Horrocks, W. D.; Taylor, R. C. Infrared Spectroscopic Study of Derivatives of Cobalt Tricarbonyl Nitrosyl. *Inorg. Chem.* **1963**, *2* (4), 723–727. https://doi.org/10.1021/ic50008a013.

(54) Magee, T. A.; Matthews, C. N.; Wang, T. S.; Wotiz, J. H. Organic Derivatives of Chromium, Molybdenum and Tungsten Carbonyls. *J. Am. Chem. Soc.* **1961**, *83* (15), 3200–3203. https://doi.org/10.1021/ja01476a008.

(55) Bond, A. M.; Carr, S. W.; Colton, R. Study of Substituent Effects, Isomerization and Cross Redox Reactions Associated with Electrochemical Oxidation of $Mo(CO)_3P_3$ Systems. *Organometallics* **1984**, *3* (4), 541–548. https://doi.org/10.1021/om00082a005.

(56) Grim, S. O.; Wheatland, D. A.; McFarlane, W. Phosphorus-31 Nuclear Magnetic Resonance Study of Tertiary Phosphine Derivatives of Group VI Metal Carbonyls. *J. Am. Chem. Soc.* **1967**, *89* (22), 5573–5577. https://doi.org/10.1021/ja00998a012.

(57) Strohmeier, W.; Müller, F. J. Notizen: π-Acceptorstärke von Phosphinen Als Liganden in Cyclopentadienylmangantricarbonyl Und Nickelcarbonyl. *Zeitschrift für Naturforsch. B* **1967**, *22* (4), 451–452. https://doi.org/10.1515/znb-1967-0416.

(58) Cotton, F. A. Vibrational Spectra and Bonding in Metal Carbonyls. III. Force Constants and Assignments of CO Stretching Modes in Various Molecules; Evaluation of CO Bond Orders. *Inorg. Chem.* **1964**, *3* (5), 702–711. https://doi.org/10.1021/ic50015a024.

(59) Alyea, E. C.; Ferguson, G.; Somogyvari, A. Crystal and Molecular Structures of $Mo(CO)_5P$(p-Tol)$_3$ and $Mo_2(CO)_8P$(p-Tol)$_3$ and Spectral Properties of Related .Sigma.- and .Pi.-Bonded Phosphine Complexes. *Organometallics* **1983**, *2* (5), 668–674. https://doi.org/10.1021/om00077a016.

(60) Dalton, J.; Paul, I.; Smith, J. G.; Stone, F. G. A. Spectroscopic Studies on Organometallic Compounds. Part XI. Infrared Spectra of Pentacarbonyl Complexes of Metals of the Chromium and Manganese Sub-Groups in the Carbonyl Stretching Region. *J. Chem. Soc. A Inorganic, Phys. Theor.* **1968**, 1195. https://doi.org/10.1039/j19680001195.

(61) Brown, R. A.; Dobson, G. R. Octahedral Metal Carbonyls. XXI. Carbonyl and Metal-Carbon Stretching Spectra of Monosubstituted Group VIB Metal Carbonyls. *Inorganica Chim. Acta* **1972**, *6*, 65–71. https://doi.org/10.1016/S0020-1693(00)91760-2.

(62) Woodard, S. S.; Angelici, R. J.; Dombek, B. D. Carbon-13 Nuclear Magnetic Resonance, Infrared, and Equilibrium Studies of Cis- and Trans-$W(CO)_4(CS)L$ Complexes. *Inorg. Chem.* **1978**, *17* (6), 1634–1639. https://doi.org/10.1021/ic50184a049.

(63) Angelici, R. J.; Sister Mary Diana Malone. Infrared Studies of Amine, Pyridine, and Phosphine Derivatives of Tungsten Hexacarbonyl. *Inorg. Chem.* **1967**, *6* (9), 1731–1736. https://doi.org/10.1021/ic50055a026.

(64) Bancroft, G. M.; Dignard-Bailey, L.; Puddephatt, R. J. Spectroscopic Study of the Effect of Methyl and Phenyl Substituents on the Basicity of Phosphine Ligands in Tungsten Carbonyl Derivatives. *Inorg. Chem.* **1986**, *25* (20), 3675–3680. https://doi.org/10.1021/ic00240a031.

(65) Darensbourg, D. J.; Nelson, H. H.; Hyde, C. L. Detailed Analysis of the Carbonyl Stretching Vibrations in Axial and Equatorial Substituted Iron Carbonyl Compounds. Absolute Infrared Intensities and Force Constants of the Carbonyl Ligands. *Inorg. Chem.* **1974**, *13* (9), 2135–2145. https://doi.org/10.1021/ic50139a017.

(66) Cunningham, D.; Goldschmidt, Z.; Gottlieb, H. E.; Hezroni-Langerman, D. Influence of Phosphine Conformation on the Structure and Stereodynamics of Tetracarbonyl(Tri-*o*-Tolylphosphine)Iron and Pentacarbonyl(Tri-*o*-Tolylphosphine)Chromium. *Inorg. Chem.* **1991**, *30* (25), 4683–4685. https://doi.org/10.1021/ic00025a001.

(67) Inoue, H.; Nakagome, T.; Kuroiwa, T.; Shirai, T.; Fluck, E. Infrared, ^{57}Fe Mössbauer, and ^{31}P NMR Spectroscopic Characterization of $Fe(CO)_4L$ (L = Phosphine and Phosphite). *Zeitschrift für Naturforsch. B* **1987**, *42* (5), 573–578. https://doi.org/10.1515/znb-1987-0510.

(68) Smith, R. T.; Baird, M. C. Metal Carbonyl Derivatives of a Water Soluble Phosphine. *Inorganica Chim. Acta* **1982**, *62*, 135–139. https://doi.org/10.1016/S0020-1693(00)88492-3.

(69) Howell, J. A. .; Lovatt, J. D.; McArdle, P.; Cunningham, D.; Maimone, E.; Gottlieb, H. E.; Goldschmidt, Z. The Effect of Fluorine, Trifluoromethyl and Related Substitution on the Donor Properties of Triarylphosphines towards [$Fe(CO)_4$]. *Inorg. Chem. Commun.* **1998**, *1* (3), 118–120. https://doi.org/10.1016/S1387-7003(98)00021-5.

(70) Otto, S.; Roodt, A. Quantifying the Electronic Cis Effect of Phosphine, Arsine and Stibine Ligands by Use of Rhodium(I) Vaska-Type Complexes. *Inorganica Chim. Acta* **2004**, *357* (1), 1–10. https://doi.org/10.1016/S0020-1693(03)00436-5.

(71) Serron, S.; Huang, J.; Nolan, S. P. Solution Thermochemical Study of Tertiary Phosphine Ligand Substitution Reactions in the Rh(Acac)(CO)(PR_3) System. *Organometallics* **1998**, *17* (4), 534–539. https://doi.org/10.1021/om970766g.

(72) Roodt, A.; Otto, S.; Steyl, G. Structure and Solution Behaviour of Rhodium(I) Vaska-Type Complexes for Correlation of Steric and Electronic Properties of Tertiary Phosphine Ligands. *Coord. Chem. Rev.* **2003**, *245* (1–2), 121–137. https://doi.org/10.1016/S0010-8545(03)00069-9.

(73) Fürstner, A.; Alcarazo, M.; Krause, H.; Lehmann, C. W. Effective Modulation of the Donor Properties of N-Heterocyclic Carbene Ligands by "Through-Space" Communication within a Planar Chiral Scaffold. *J. Am. Chem. Soc.* **2007**, *129* (42), 12676–12677. https://doi.org/10.1021/ja076028t.

(74) Canac, Y.; Lepetit, C.; Abdalilah, M.; Duhayon, C.; Chauvin, R. Diaminocarbene and

Phosphonium Ylide Ligands: A Systematic Comparison of Their Donor Character. *J. Am. Chem. Soc.* **2008**, *130* (26), 8406–8413. https://doi.org/10.1021/ja801159v.

(75) Nonnenmacher, M.; Buck, D. M.; Kunz, D. Experimental and Theoretical Investigations on the High-Electron Donor Character of Pyrido-Annelated N-Heterocyclic Carbenes. *Beilstein J. Org. Chem.* **2016**, *12*, 1884–1896. https://doi.org/10.3762/bjoc.12.178.

(76) Huynh, H. V. Electronic Properties of N-Heterocyclic Carbenes and Their Experimental Determination. *Chem. Rev.* **2018**, *118* (19), 9457–9492. https://doi.org/10.1021/acs.chemrev.8b00067.

(77) Kelly III, R. A.; Clavier, H.; Giudice, S.; Scott, N. M.; Stevens, E. D.; Bordner, J.; Samardjiev, I.; Hoff, C. D.; Cavallo, L.; Nolan, S. P. Determination of N-Heterocyclic Carbene (NHC) Steric and Electronic Parameters Using the [(NHC)Ir$(CO)_2$Cl] System. *Organometallics* **2008**, *27* (2), 202–210. https://doi.org/10.1021/om701001g.

(78) Dicks, A. P. Using Hydrocarbon Acidities To Demonstrate Principles of Organic Structure and Bonding. *J. Chem. Educ.* **2003**, *80* (11), 1322. https://doi.org/10.1021/ed080p1322.

(79) Konishi, S.; Iwai, T.; Sawamura, M. Synthesis, Properties, and Catalytic Application of a Triptycene-Type Borate-Phosphine Ligand. *Organometallics* **2018**, *37* (12), 1876–1883. https://doi.org/10.1021/acs.organomet.8b00113.

(80) Van Rentergem, M.; Van Der Kelen, G. P.; Claeys, E. C. Study of the Co-Stretching Frequencies and Intensities in Metal-Carbonyl Compounds. Part I. The Compounds LFe$(CO)_4$ with L an Organophosphorous Ligand. *J. Mol. Struct.* **1982**, *80*, 317–324. https://doi.org/10.1016/0022-2860(82)87249-9.

(81) Martin, L. R.; Einstein, F. W. B.; Pomeroy, R. K. Axial-Equatorial Isomerism in the Pentacoordinate Complexes M$(CO)_4$(EPh$_3$) (M = Fe, Ru, Os; E = P, As, Sb). *Inorg. Chem.* **1983**, *22* (14), 1959–1961. https://doi.org/10.1021/ic00156a001.

(82) Udovich, C. A.; Clark, R. J.; Haas, H. Stereochemical Nonrigidity in Iron Carbonyl Fluorophosphine Compounds. *Inorg. Chem.* **1969**, *8* (5), 1066–1072. https://doi.org/10.1021/ic50075a009.

(83) Kroshefsky, R. D.; Verkade, J. G.; Pipal, J. R. COORDINATION PROPERTIES OF CONSTRAINED AMINOPHOSPHINES. *Phosphorous Sulfur Relat. Elem.* **1979**, *6* (3), 377–389. https://doi.org/10.1080/03086647908069897.

(84) Cremer, D.; Kalescky, R.; Kraka, E. New Approach to Tolman's Electronic Parameter Based on Local Vibrational Modes. *Inorg. Chem.* **2014**, *53* (1), 478–495. https://doi.org/10.1021/ic4024663.

(85) Li, H.; Aquino, A. J. A.; Cordes, D. B.; Hase, W. L.; Krempner, C. Electronic Nature of Zwitterionic Alkali Metal Methanides, Silanides and Germanides – a Combined Experimental and Computational Approach. *Chem. Sci.* **2017**, *8* (2), 1316–1328. https://doi.org/10.1039/C6SC02390H.

(86) Denk, M.; Green, J. C.; Metzler, N.; Wagner, M. Electronic Structure of a Stable Silylene: Photoelectron Spectra and Theoretical Calculations of Si(NRCHCHNR), $Si(NRCH_2CH_2NR)$ and $SiH_2(NRCHCHNR)$. *J. Chem. Soc. Dalt. Trans.* **1994**, No. 16, 2405–2410. https://doi.org/10.1039/dt9940002405.

(87) Kong, L.; Zhang, J.; Song, H.; Cui, C. *N*-Aryl Substituted Heterocyclic Silylenes. *Dalt. Trans.* **2009**, No. 28, 5444–5446. https://doi.org/10.1039/b905905a.

(88) So, C.-W.; Roesky, H. W.; Gurubasavaraj, P. M.; Oswald, R. B.; Gamer, M. T.; Jones, P. G.; Blaurock, S. Synthesis and Structures of Heteroleptic Silylenes. *J. Am. Chem. Soc.* **2007**, *129* (39), 12049–12054. https://doi.org/10.1021/ja074019e.

(89) Matthews, W. S.; Bares, J. E.; Bartmess, J. E.; Bordwell, F. G.; Cornforth, F. J.; Drucker, G. E.; Margolin, Z.; McCallum, R. J.; McCollum, G. J.; Vanier, N. R. Equilibrium Acidities of Carbon Acids. VI. Establishment of an Absolute Scale of Acidities in Dimethyl Sulfoxide Solution. *J. Am. Chem. Soc.* **1975**, *97* (24), 7006–7014. https://doi.org/10.1021/ja00857a010.

(90) Bordwell, F. G.; Branca, J. C.; Hughes, D. L.; Olmstead, W. N. Equilibriums Involving Organic Anions in Dimethyl Sulfoxide and *N*-Methylpyrrolidin-2-One: Acidities, Ion Pairing, and Hydrogen Bonding. *J. Org. Chem.* **1980**, *45* (16), 3305–3313. https://doi.org/10.1021/jo01304a034.

(91) Lindon, J. C.; Tranter, G. E.; Koppenaal, D. *Encyclopedia of Spectroscopy and Spectrometry*; Elsevier Science, 2016.

(92) Muetterties, E. L. Topological Representation of Stereoisomerism. I. Polytopal Rearrangements. *J. Am. Chem. Soc.* **1969**, *91* (7), 1636–1643. https://doi.org/10.1021/ja01035a009.

(93) Muetterties, E. L. Topological Representation of Stereoisomerism. II. The Five-Atom Family. *J. Am. Chem. Soc.* **1969**, *91* (15), 4115–4122. https://doi.org/10.1021/ja01043a017.

(94) Couzijn, E. P. A. A.; Slootweg, J. C.; Ehlers, A. W.; Lammertsma, K. Stereomutation of Pentavalent Compounds: Validating the Berry Pseudorotation, Redressing Ugi's Turnstile Rotation, and Revealing the Two- and Three-Arm Turnstiles. *J. Am. Chem. Soc.* **2010**, *132* (51), 18127–18140. https://doi.org/10.1021/ja105306s.

(95) Fukui, K. The Path of Chemical Reactions - the IRC Approach. *Acc. Chem. Res.* **1981**, *14* (12), 363–368. https://doi.org/10.1021/ar00072a001.

(96) Taketsugu, T.; Tajima, N.; Hirao, K. Approaches to Bifurcating Reaction Path. *J. Chem. Phys.* **1996**, *105* (5), 1933–1939. https://doi.org/10.1063/1.472063.

(97) Ess, D. H.; Wheeler, S. E.; Iafe, R. G.; Xu, L.; Çelebi-Ölçüm, N.; Houk, K. N. Bifurcations on Potential Energy Surfaces of Organic Reactions. *Angew. Chemie Int. Ed.* **2008**, *47* (40), 7592–7601. https://doi.org/10.1002/anie.200800918.

(98) Valtazanos, P.; Ruedenberg, K. Bifurcations and Transition States. *Theor. Chim. Acta* **1986**, *69*, 281–307. https://doi.org/10.1007/BF00527705.

(99) Singleton, D. A.; Hang, C.; Szymanski, M. J.; Meyer, M. P.; Leach, A. G.; Kuwata, K. T.; Chen, J. S.; Greer, A.; Foote, C. S.; Houk, K. N. Mechanism of Ene Reactions of Singlet Oxygen. A Two-Step No-Intermediate Mechanism. *J. Am. Chem. Soc.* **2003**, *125* (5), 1319–1328. https://doi.org/10.1021/ja027225p.

(100) Quapp, W.; Hirsch, M.; Heidrich, D. An Approach to Reaction Path Branching Using Valley-Ridge Inflection Points of Potential-Energy Surfaces. *Theor. Chem. Accounts Theory, Comput. Model. (Theoretica Chim. Acta)* **2004**, *112*, 40–51. https://doi.org/10.1007/s00214-003-0558-8.

(101) QUAPP, W. REDUCED GRADIENT METHODS AND THEIR RELATION TO REACTION PATHS. *J. Theor. Comput. Chem.* **2003**, *02* (03), 385–417. https://doi.org/10.1142/S0219633603000604.

(102) Wales, D. J. Potential Energy Surfaces and Coordinate Dependence. *J. Chem. Phys.* **2000**, *113* (9), 3926–3927. https://doi.org/10.1063/1.1288003.

(103) Truflandier, L. A.; Brendler, E.; Wagler, J.; Autschbach, J. ^{29}Si DFT/NMR Observation of Spin-Orbit Effect in Metallasilatrane Sheds Some Light on the Strength of the Metal→Silicon Interaction. *Angew. Chemie Int. Ed.* **2011**, *50* (1), 255–259. https://doi.org/10.1002/anie.201005431.

S7 Spectra of isolated compounds

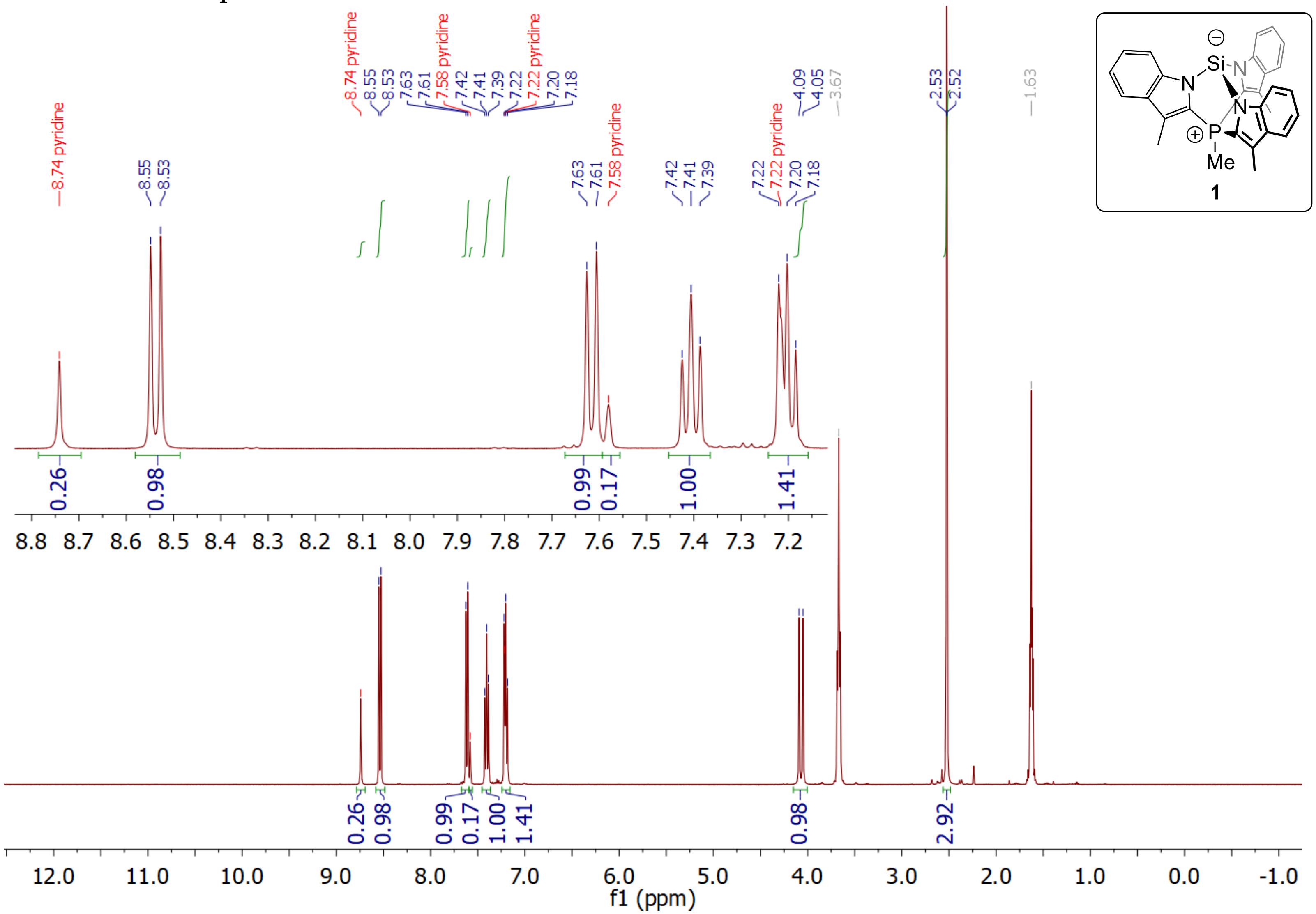

Figure S23. ^{1}H NMR (400 MHz) spectrum of compound **1** in pyridine-d_5. Impurities: 1.63 and 3.67 ppm – THF, other minor peaks are unassigned.

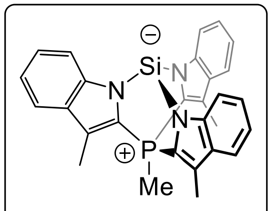

1

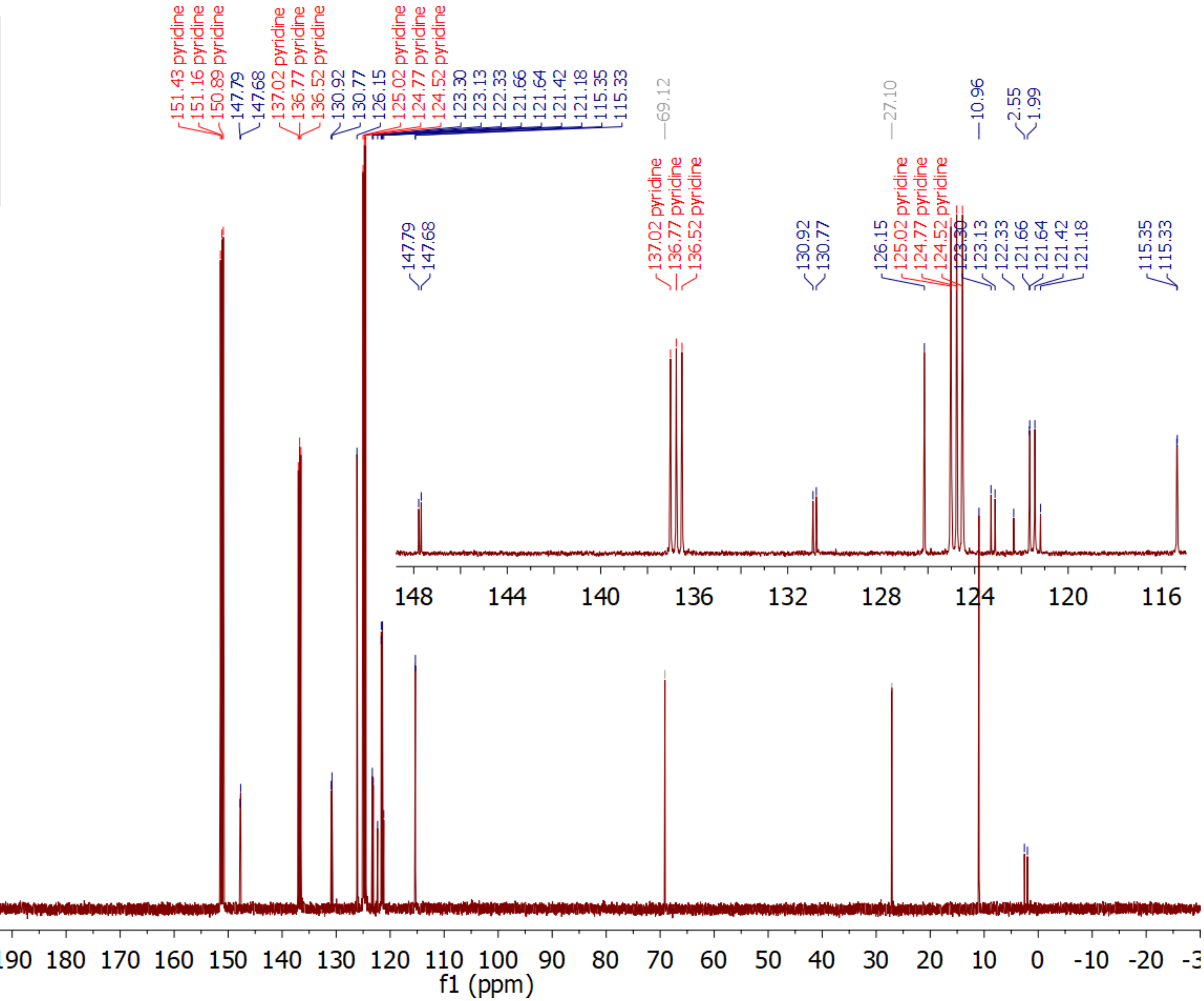

Figure S24. $^{13}C\{^{1}H\}$ NMR (101 MHz) spectrum of compound **1** in pyridine-d_5. Impurities: 27.10 and 69.12 ppm – THF.

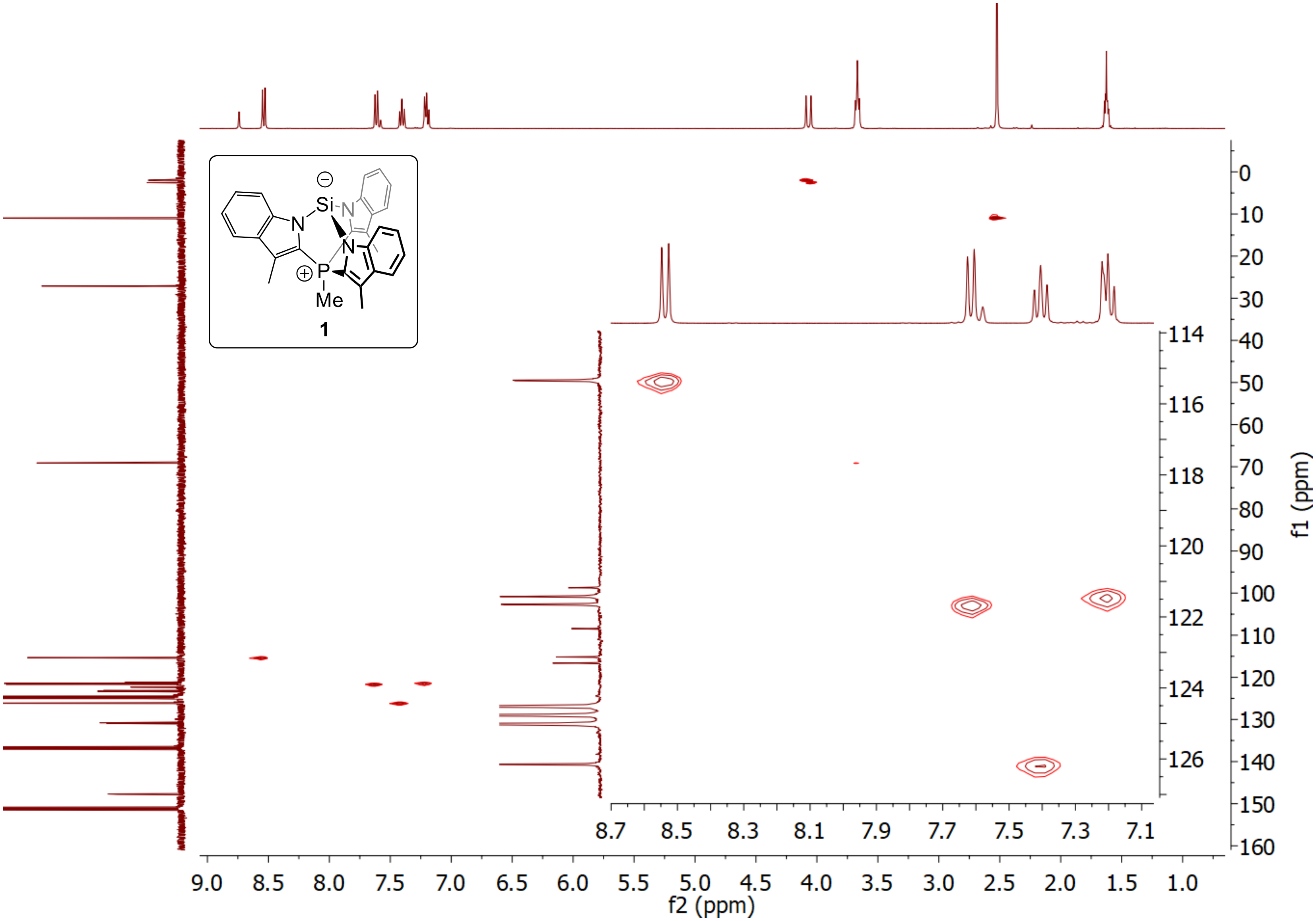

Figure S25. ^{1}H-^{13}C ASAPHMQC spectrum of compound **1** in pyridine-d_5.

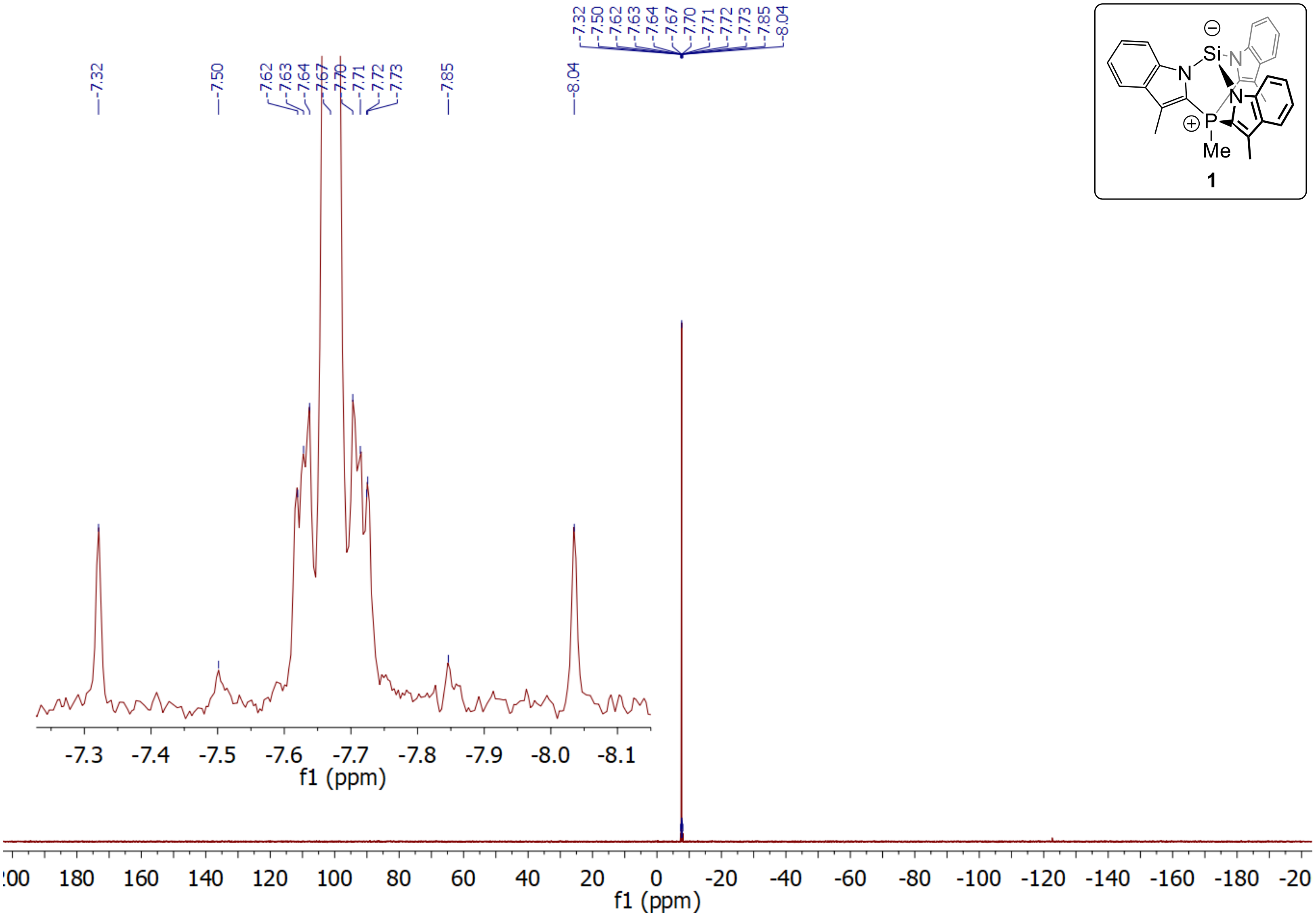

Figure S26. $^{31}P\{^{1}H\}$ NMR (162 MHz) spectrum of compound **1** in pyridine-d_5.

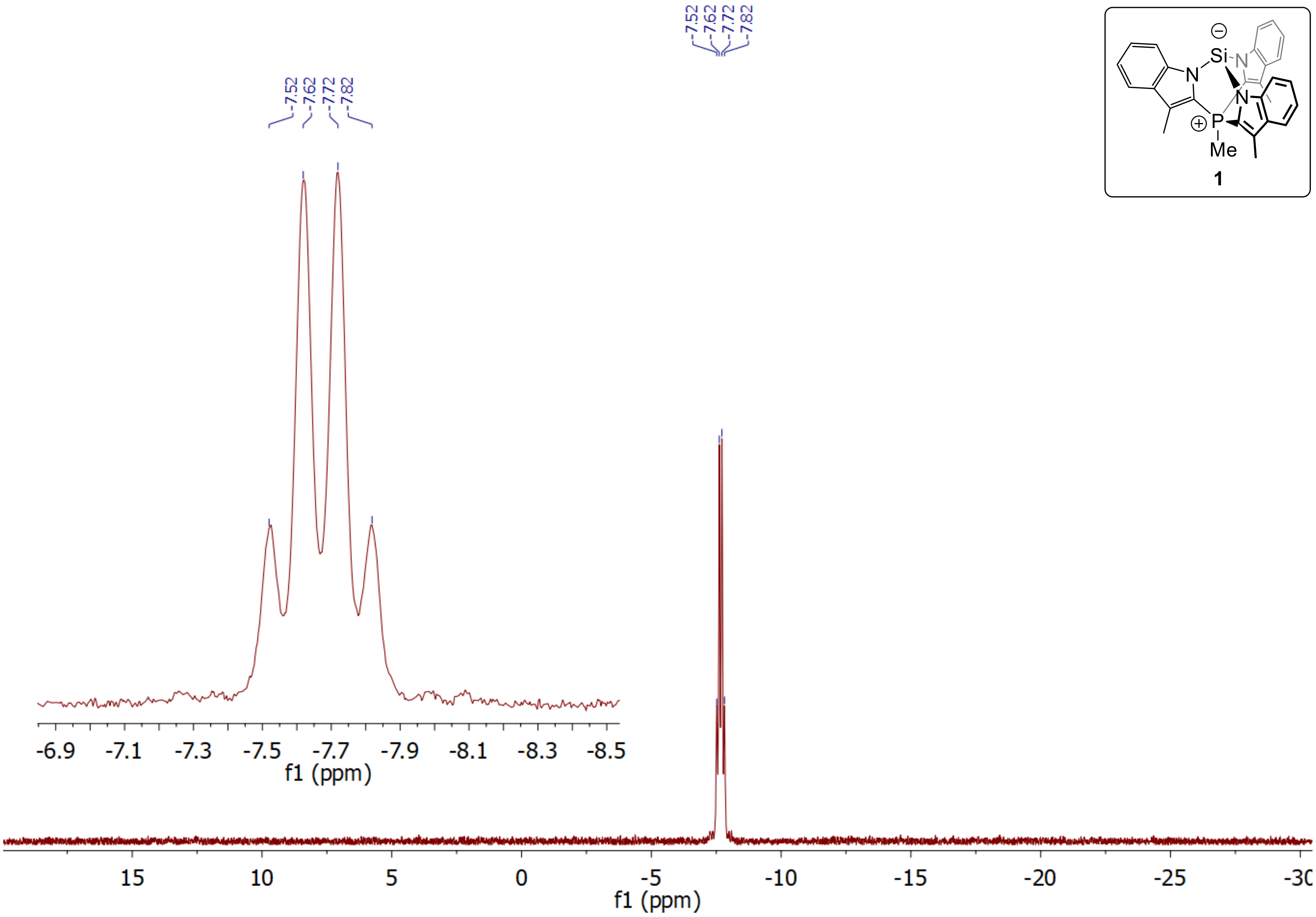

Figure S27. ^{31}P NMR (162 MHz) spectrum of compound **1** in pyridine-d_5.

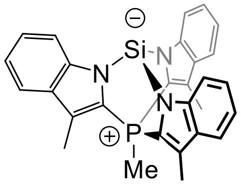

1

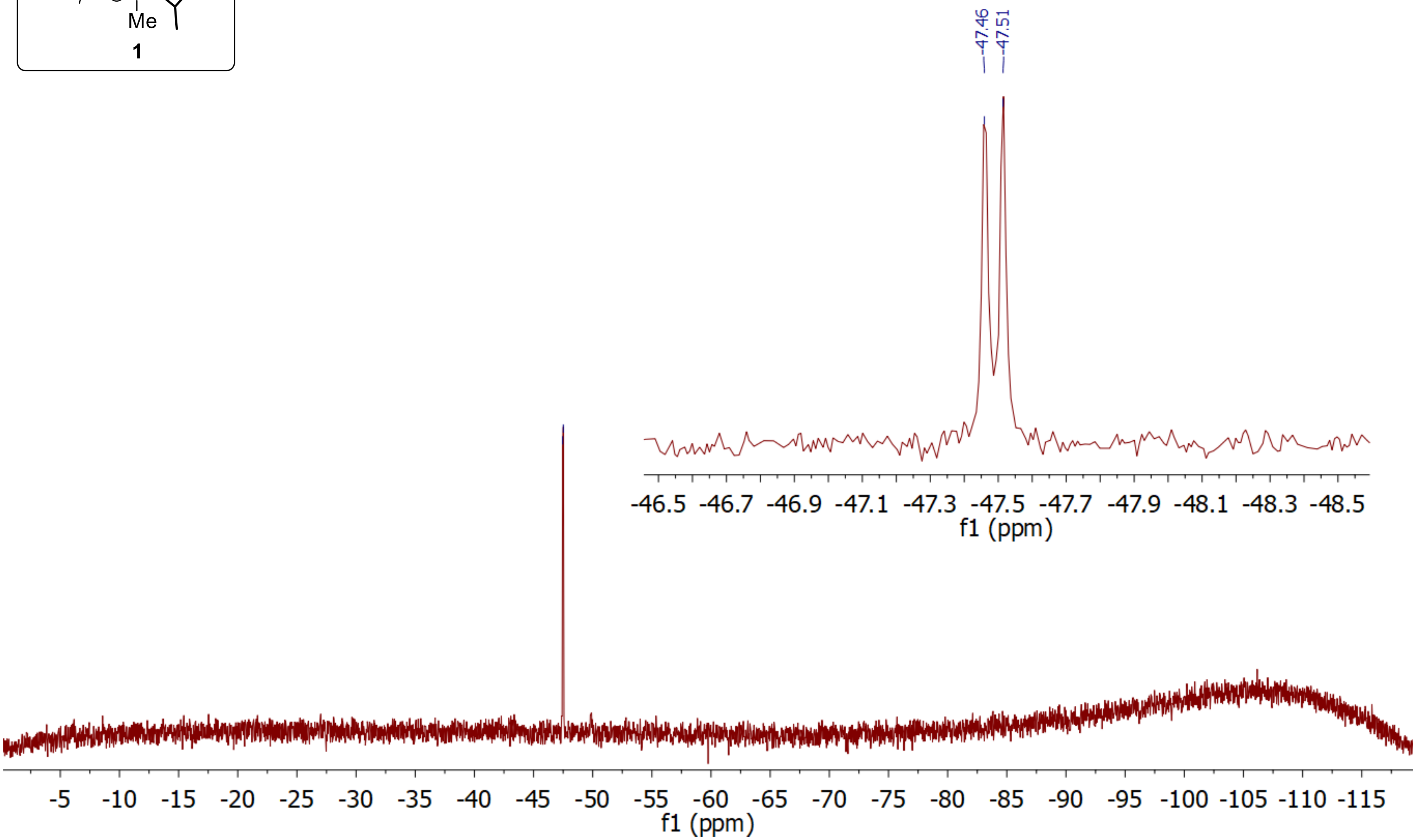

Figure S28. $^{29}Si\{^{1}H\}$ NMR (79 MHz) spectrum of compound **1** in pyridine-d_5.

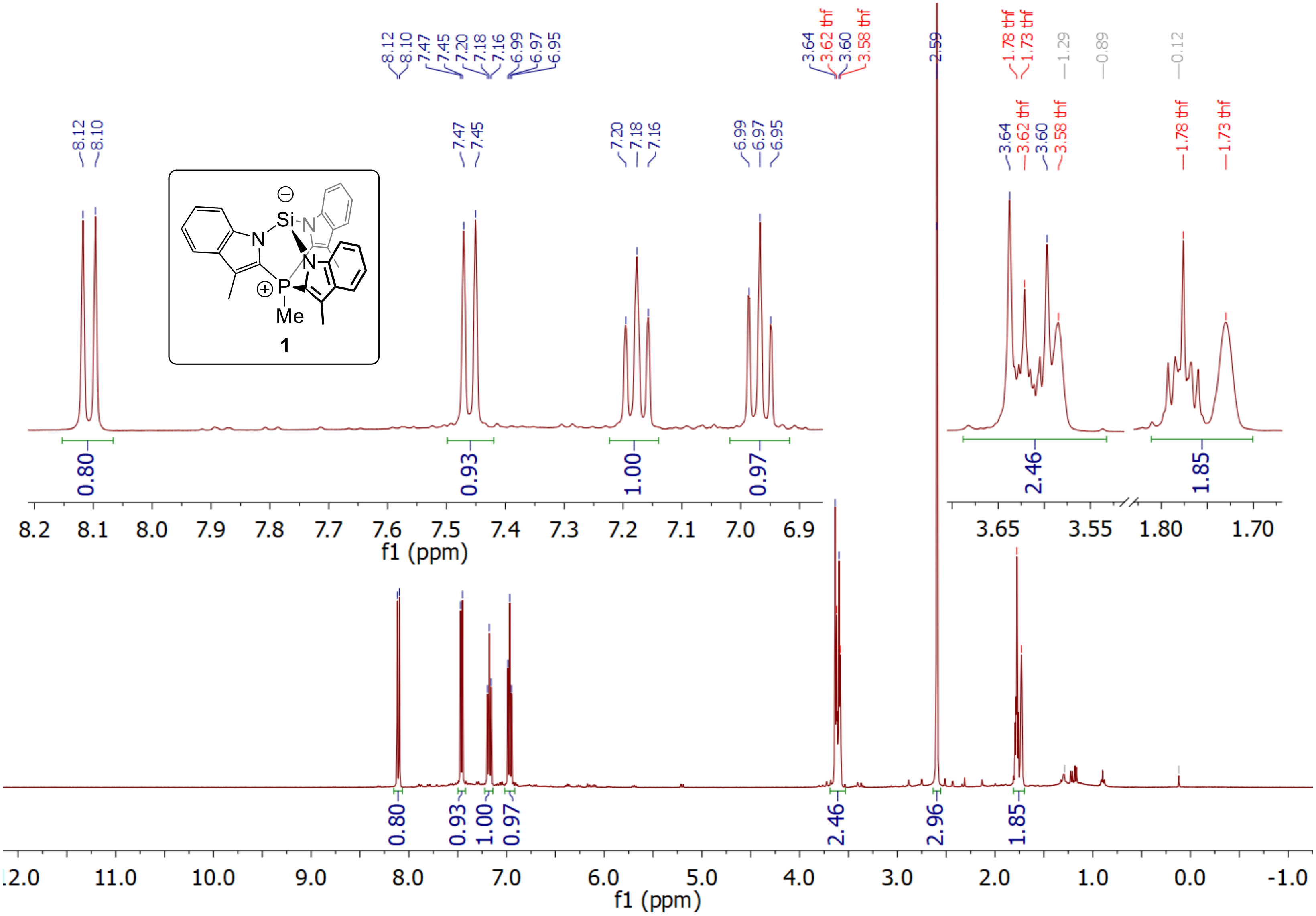

Figure S29. ^{1}H NMR (400 MHz) spectrum of compound **1** in THF-d_8. Impurities: 0.12 ppm – grease, 0.89 and 1.29 ppm – hexane, other minor peaks are unassigned.

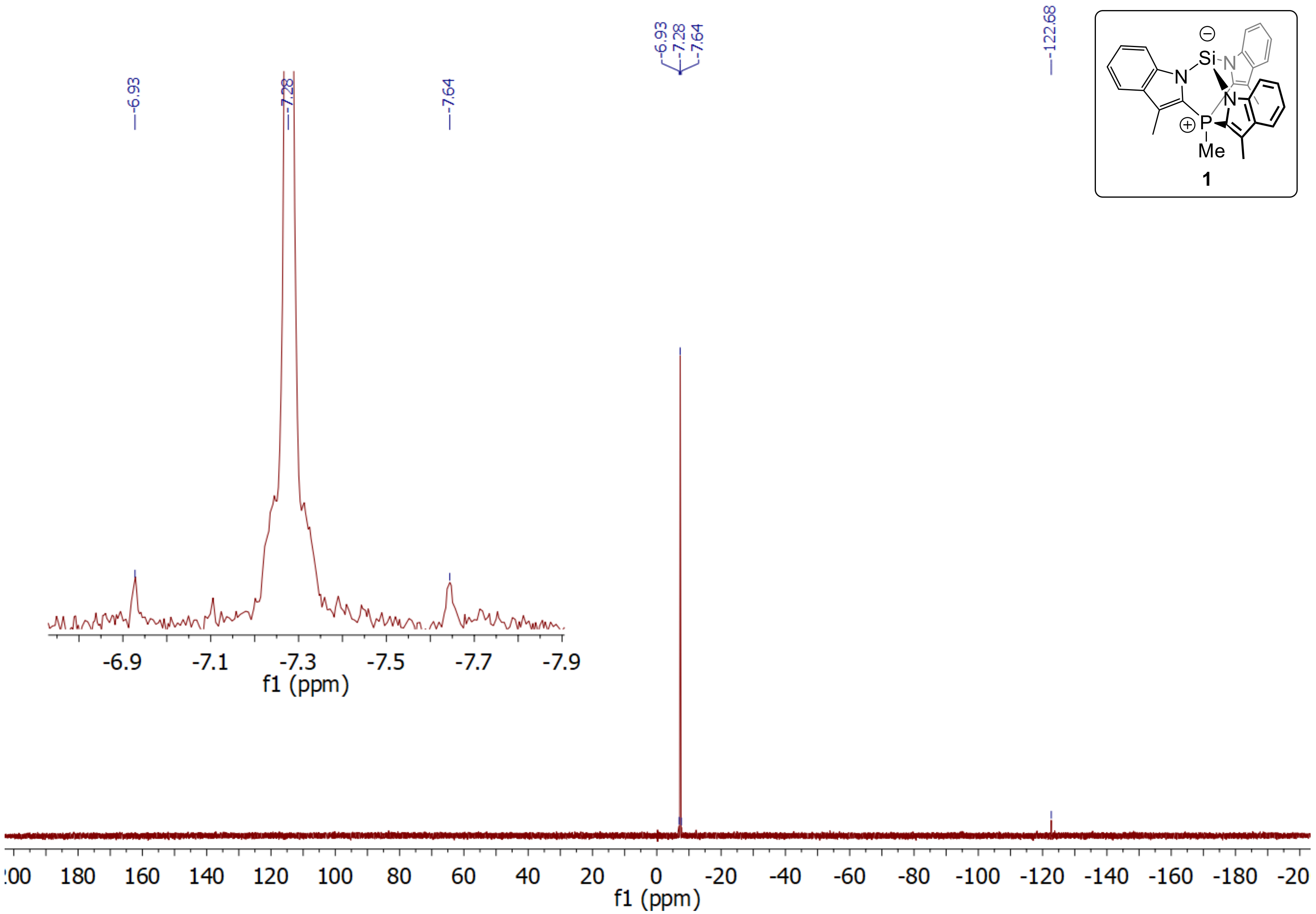

Figure S30. $^{31}P\{^{1}H\}$ NMR (162 MHz) spectrum of compound **1** in THF-d_8. Impurities: -122.68 ppm – is a trace of isomer **10**.

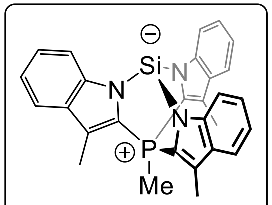

1

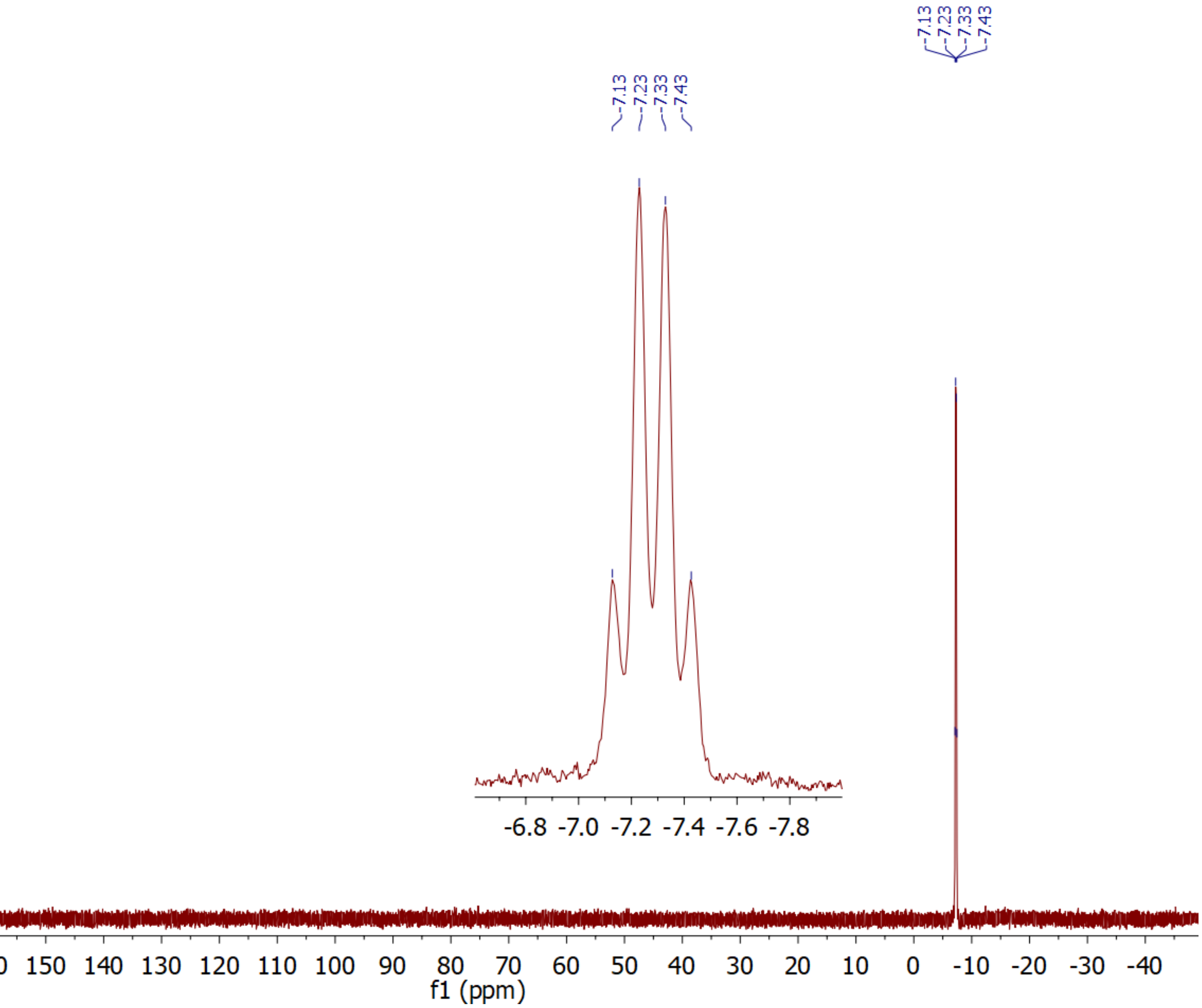

Figure S31. ^{31}P NMR (162 MHz) spectrum of compound **1** in THF-d_8.

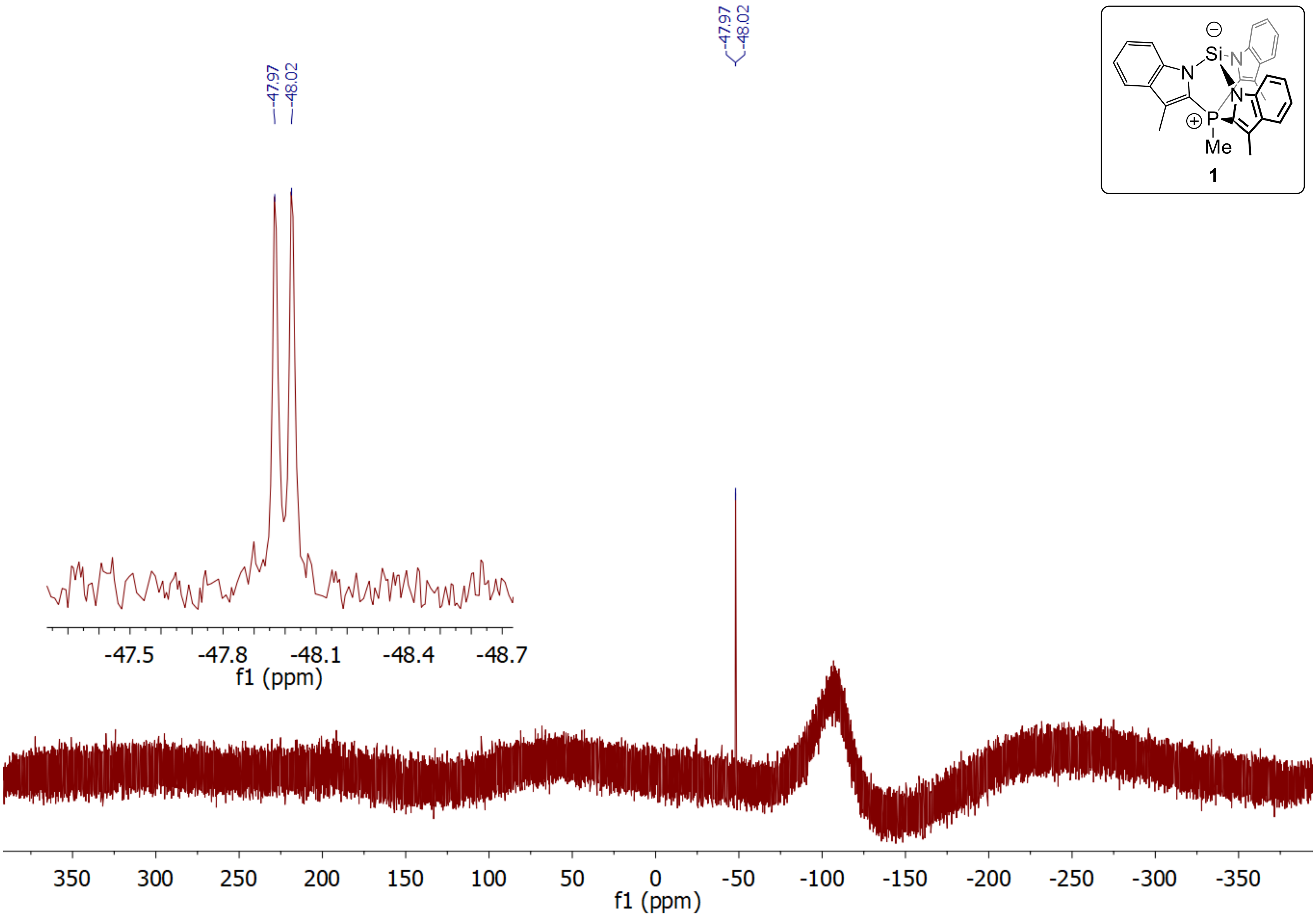

1

Figure S32. $^{29}Si\{^1H\}$ NMR (79 MHz) spectrum of compound **1** in THF-d_8.

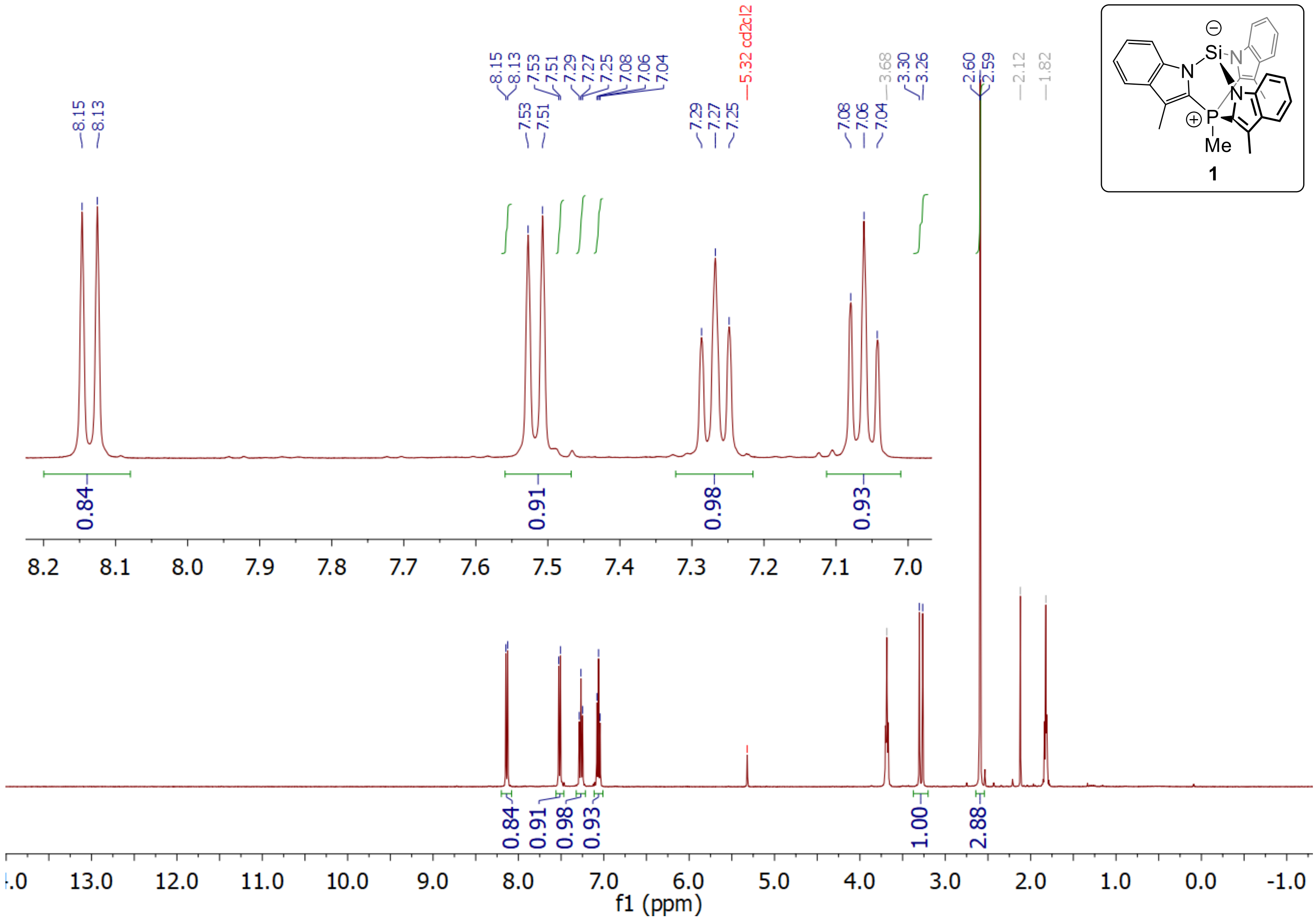

Figure S33. ^{1}H NMR (400 MHz) spectrum of compound **1** in DCM-d_2. Impurities: 1.82 and 3.68 ppm – THF, 2.12 – unidentified.

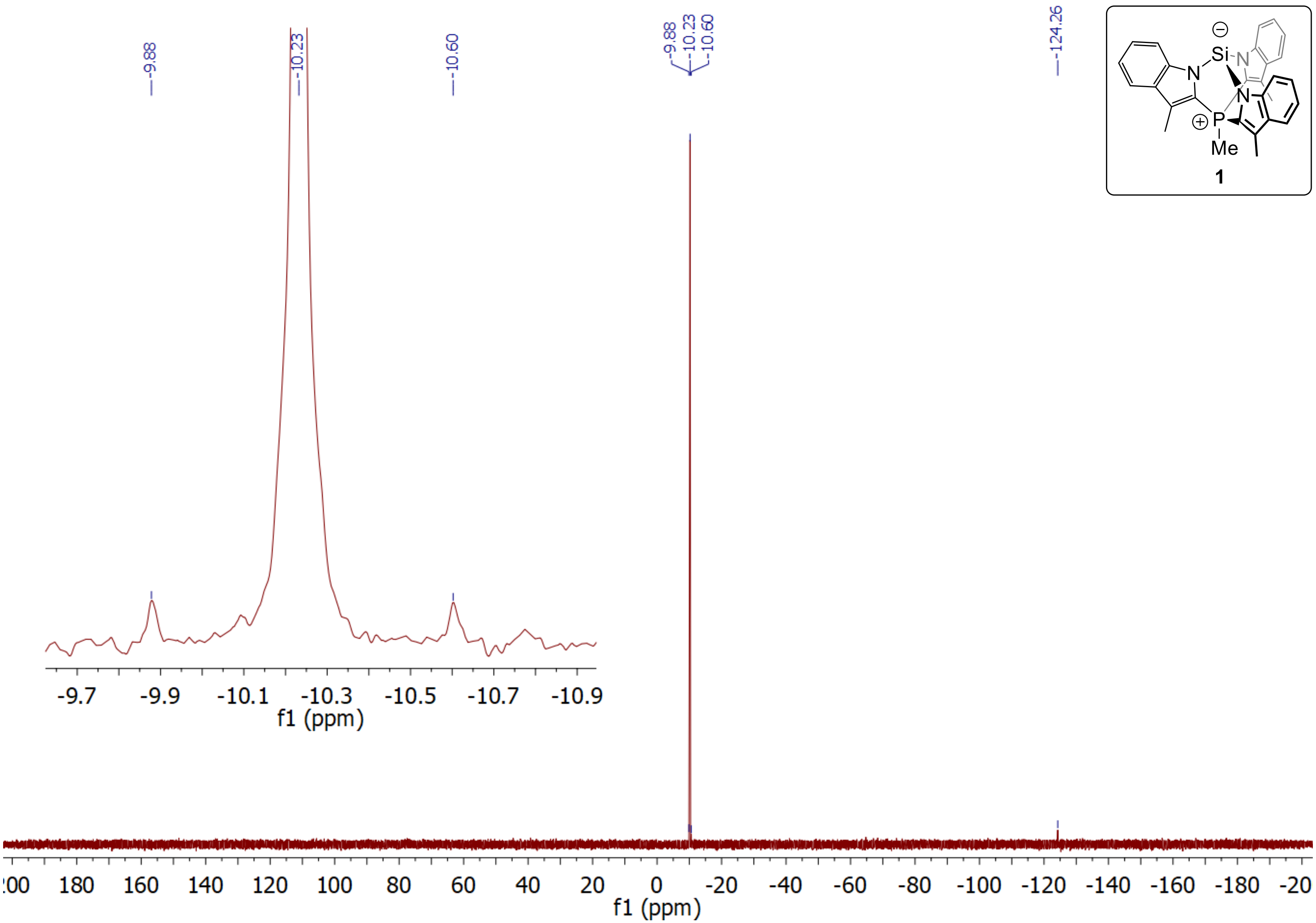

Figure S34. $^{31}P\{^1H\}$ NMR (162 MHz) spectrum of compound **1** in DCM-d_2. Impurities: -124.26 ppm – traces of isomer **10**.

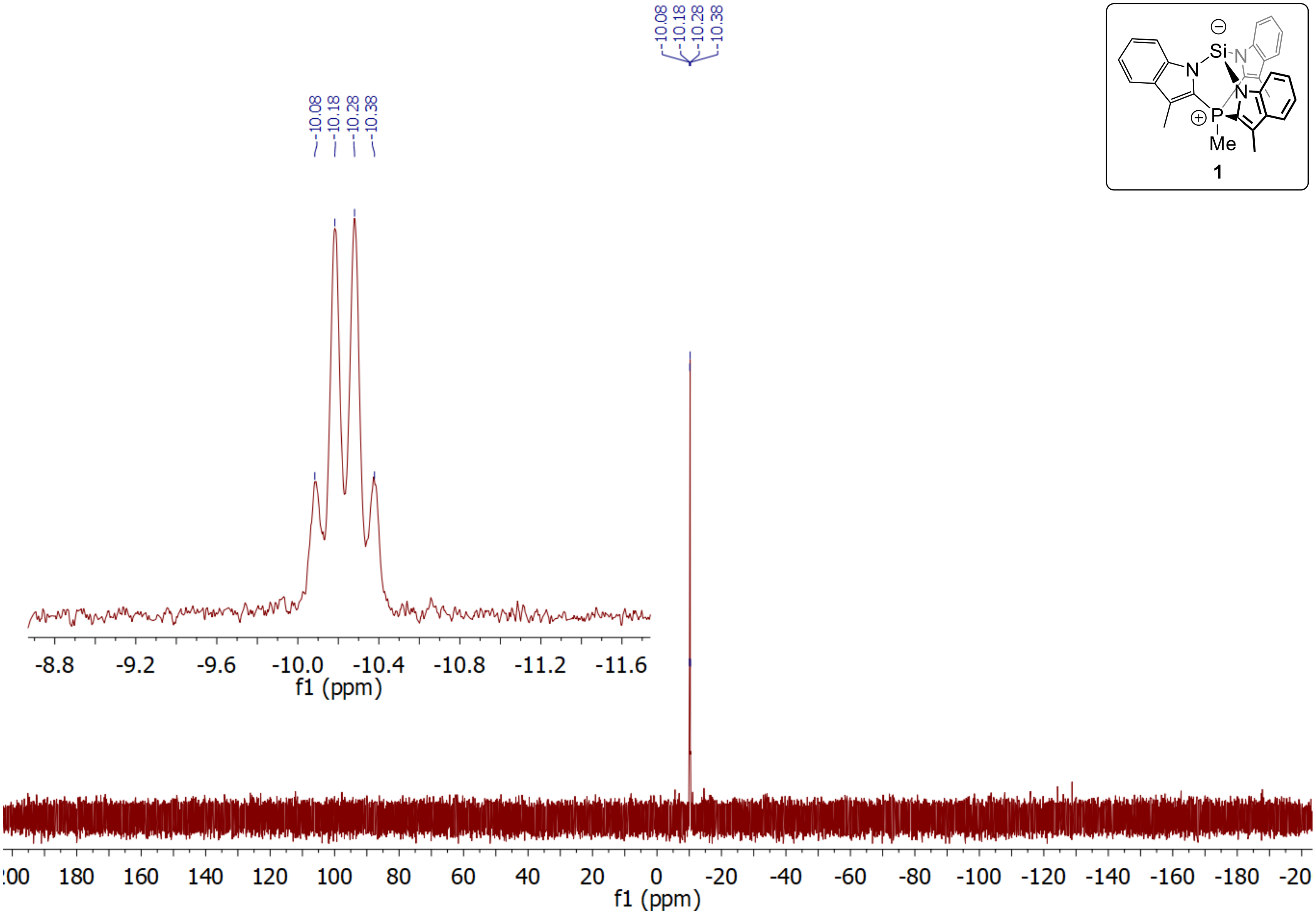

Figure S35. ^{31}P NMR (162 MHz) spectrum of compound **1** in DCM-d_2.

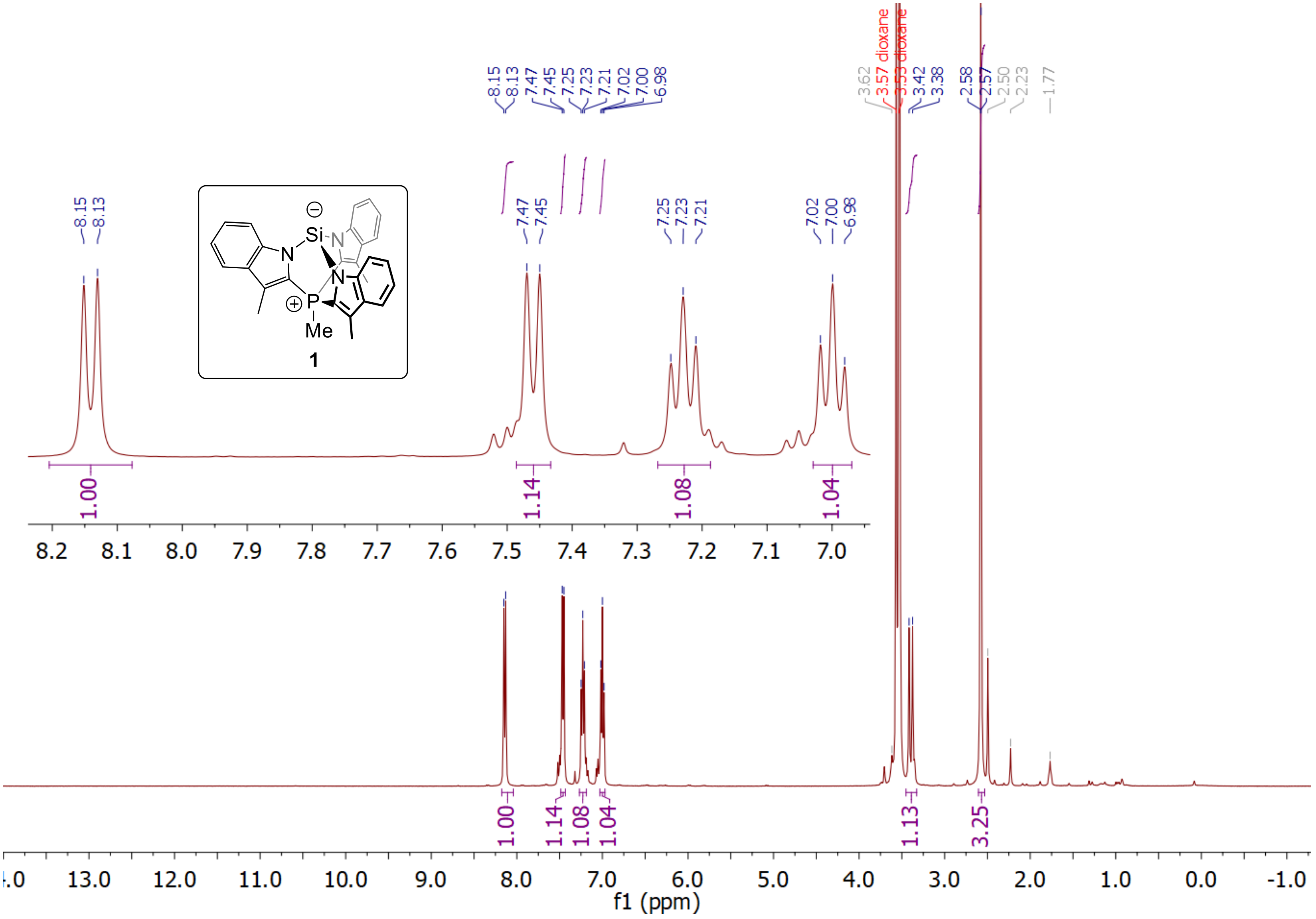

Figure S36. ^{1}H NMR (400 MHz) spectrum of compound **1** in dioxane-d_8. Impurities: 2.23 and 2.50 ppm – traces of isomer **10**, other minor peaks are unidentified.

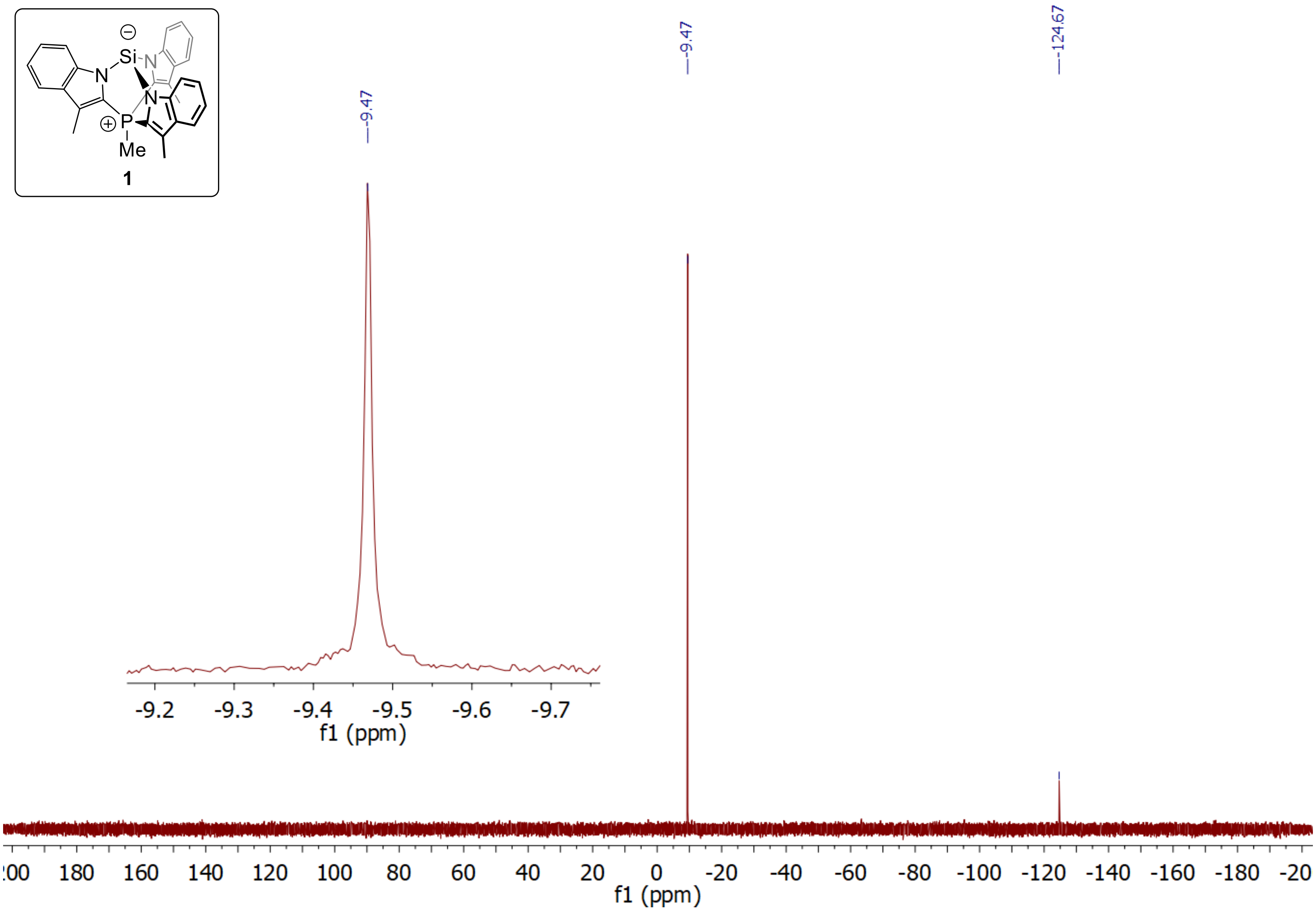

Figure S37. $^{31}P\{^1H\}$ NMR (162 MHz) spectrum of compound **1** in dioxane-d_8. Impurities: -124.67 ppm – traces of isomer **10**.

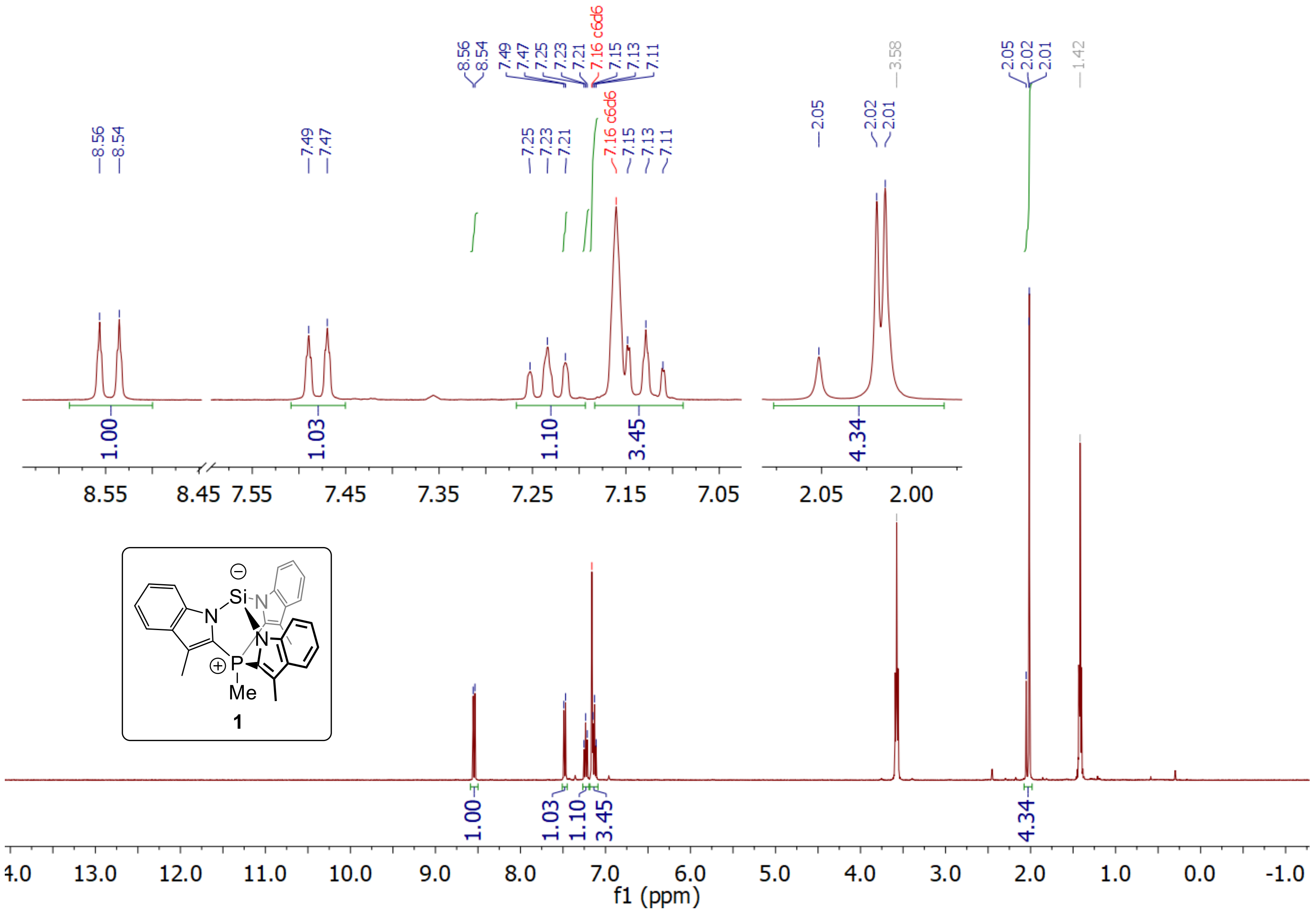

Figure S38. ^{1}H NMR (400 MHz) spectrum of compound **1** in benzene-d_6. Impurities: 1.42 and 3.58 ppm – THF, other minor peaks are unidentified.

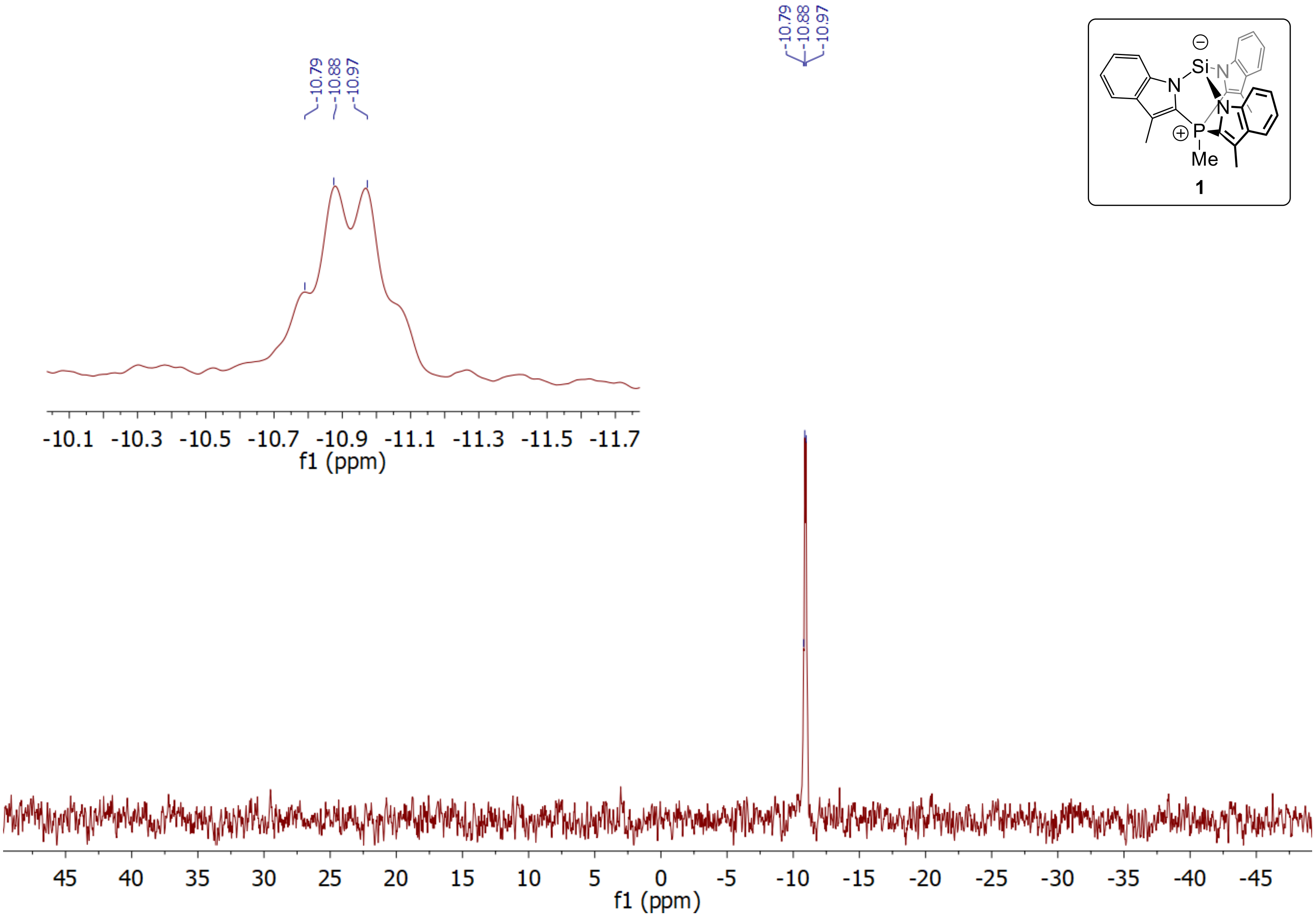

Figure S39. ^{31}P NMR (162 MHz) spectrum of compound **1** in benzene-d$_6$.

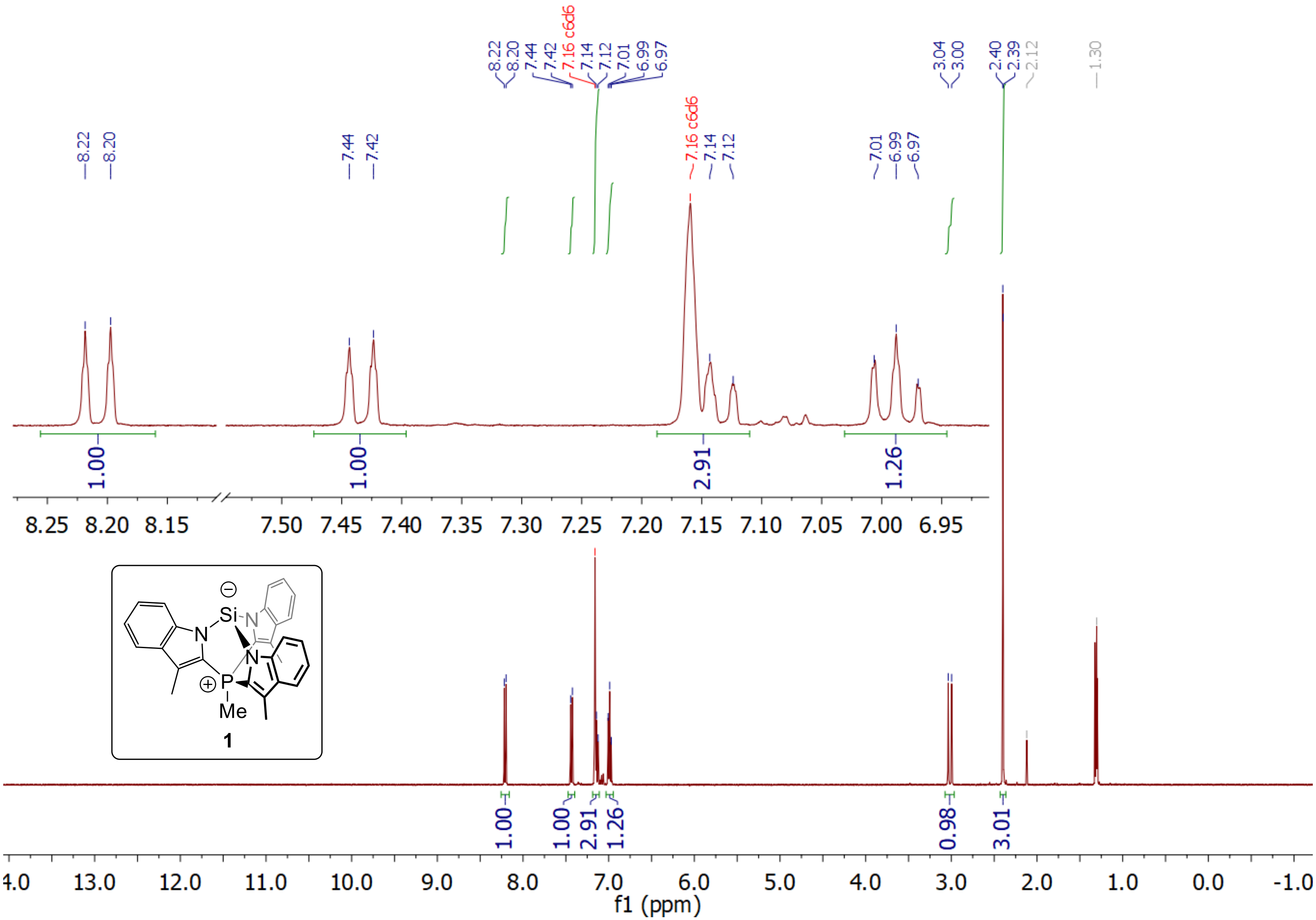

Figure S40. ^{1}H NMR (400 MHz) spectrum of compound **1** in acetonitrile-d_3/benzene-d_6 1:4 (v/v). Impurities: 1.30 ppm – acetonitrile, 2.12 ppm - unidentified

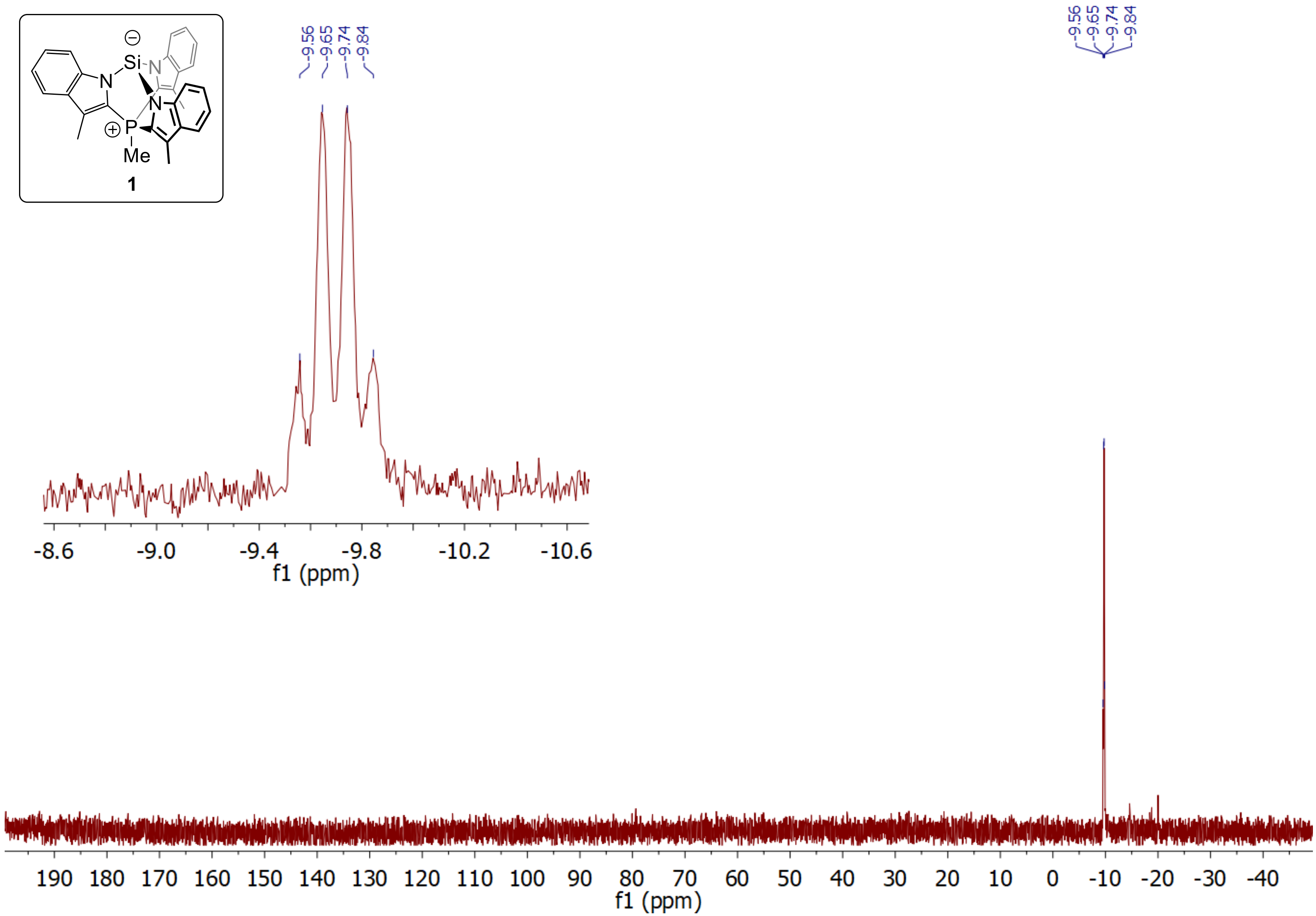

Figure S41. ^{31}P NMR (162 MHz) spectrum of compound **1** in acetonitrile-d_3/benzene-d_6 1:4 (v/v).

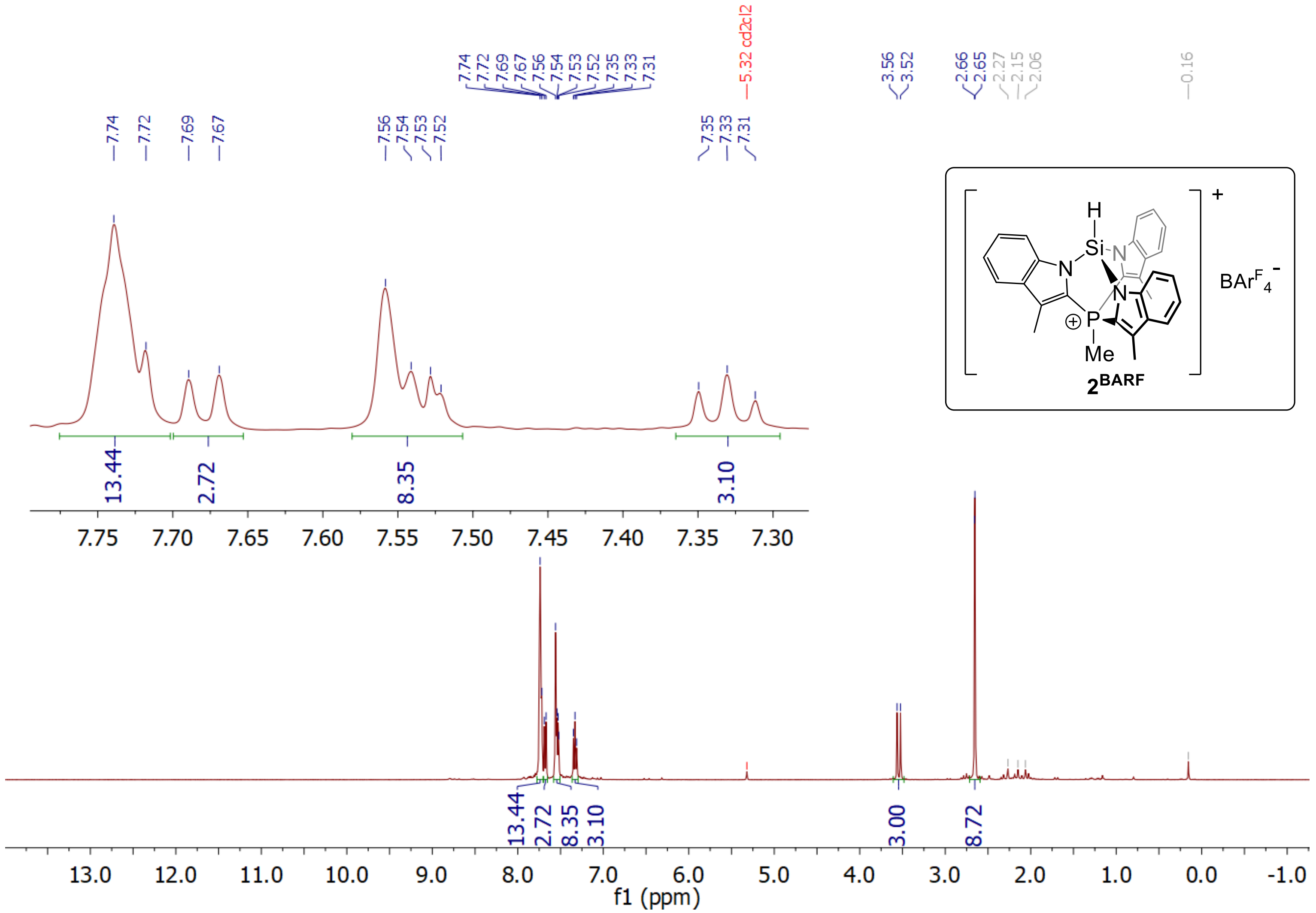

Figure S42. ^{1}H NMR (400 MHz) spectrum of compound **2^{BARF}** in DCM-d_2. Impurities: 0.16 ppm – grease, other minor peaks are unidentified. Increased intensity of BAr$^F{}_4{}^-$ -derived peaks at 7.56 and 7.74 ppm suggests the presence of a small amount (ca. 15%) of a contaminating BAr$^F{}_4{}^-$ salt, possibly NaBAr$^F{}_4$ or HBAr$^F{}_4$.

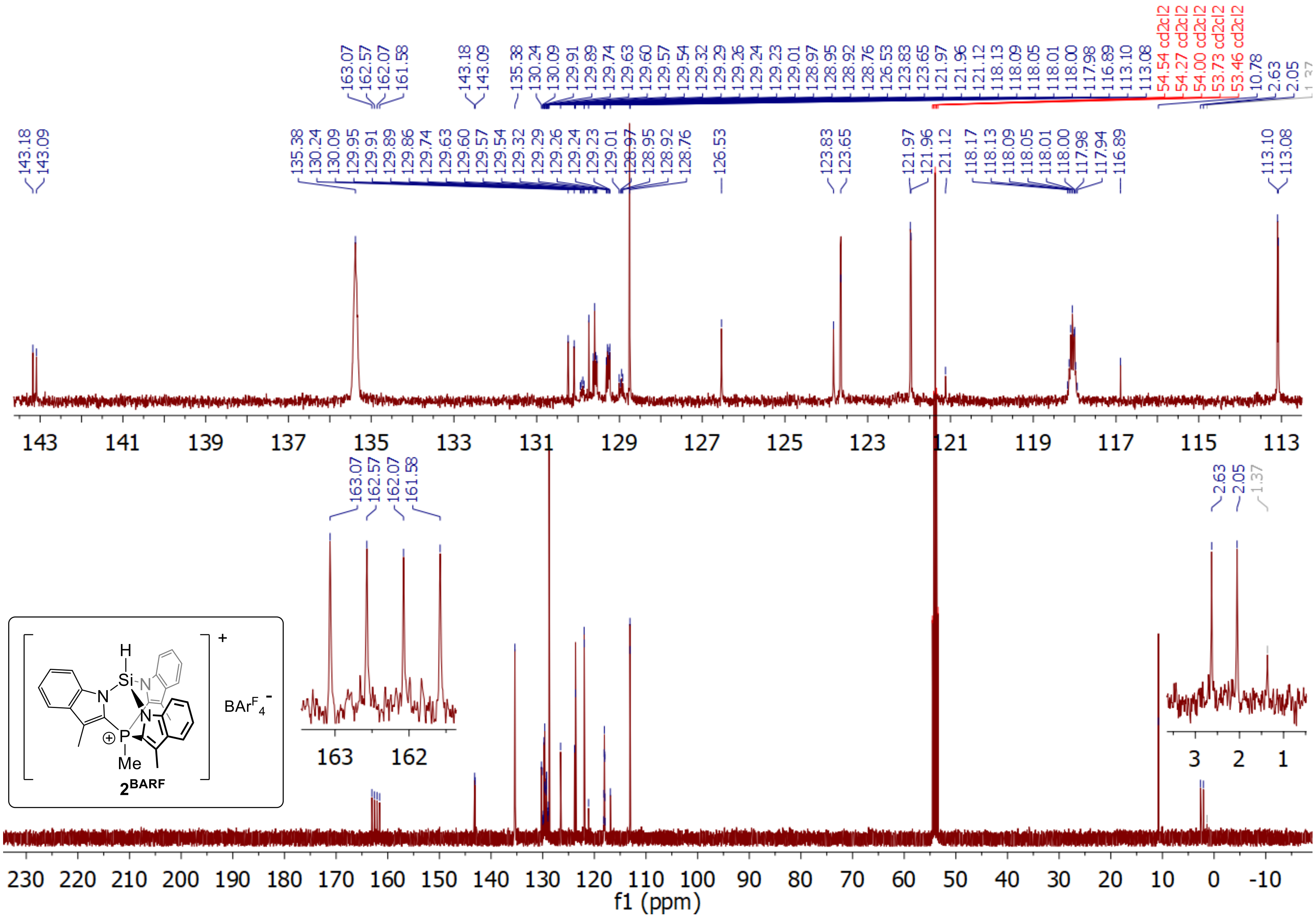

Figure S43. $^{13}C\{^{1}H\}$ NMR (101 MHz) spectrum of compound **2**$^{\mathbf{BARF}}$ in DCM-d_2. Impurities: 1.37 ppm – grease.

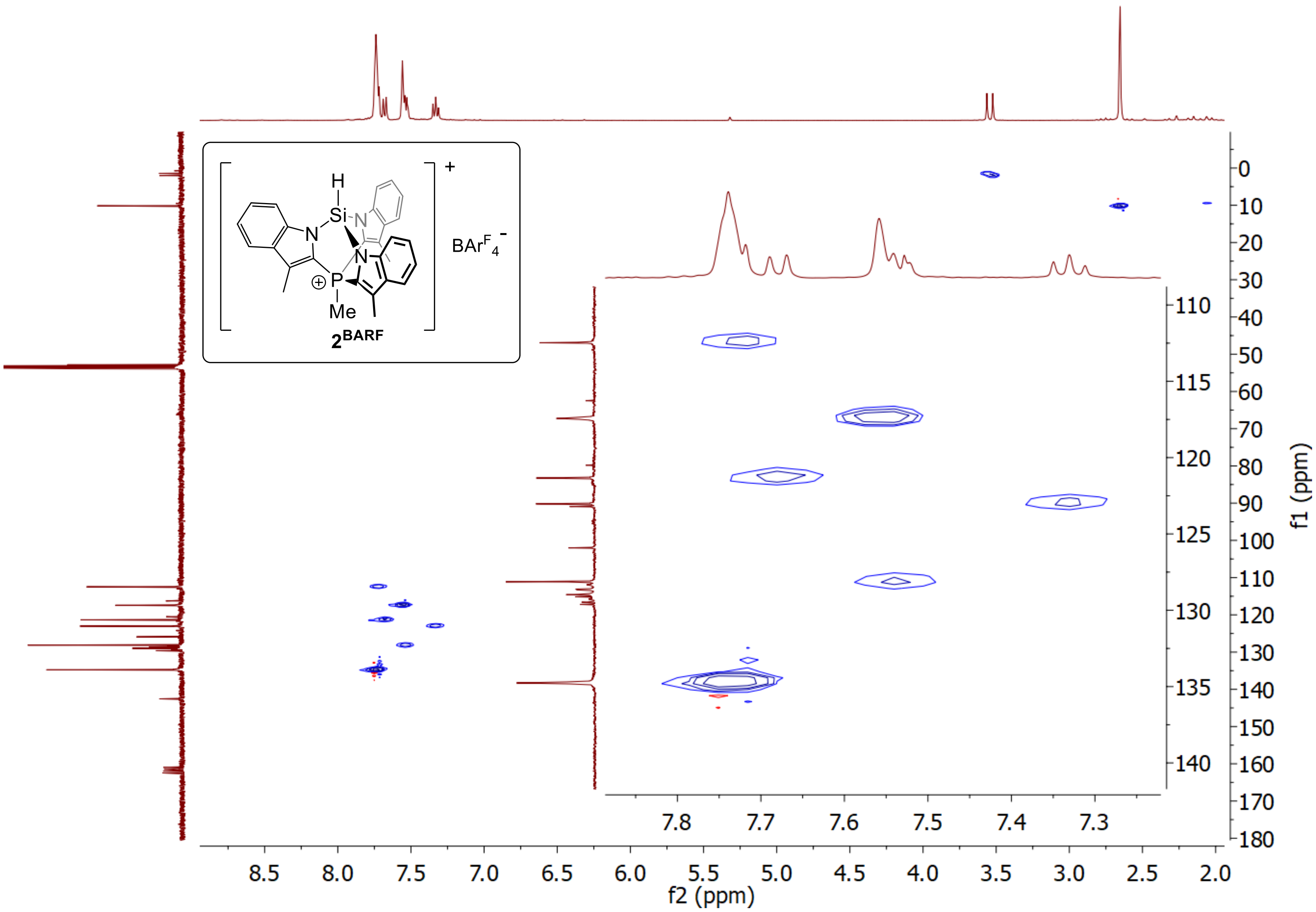

Figure S44. ^{1}H-^{13}C ASAPHMQC spectrum of compound **2^{BARF}** in DCM-d_2.

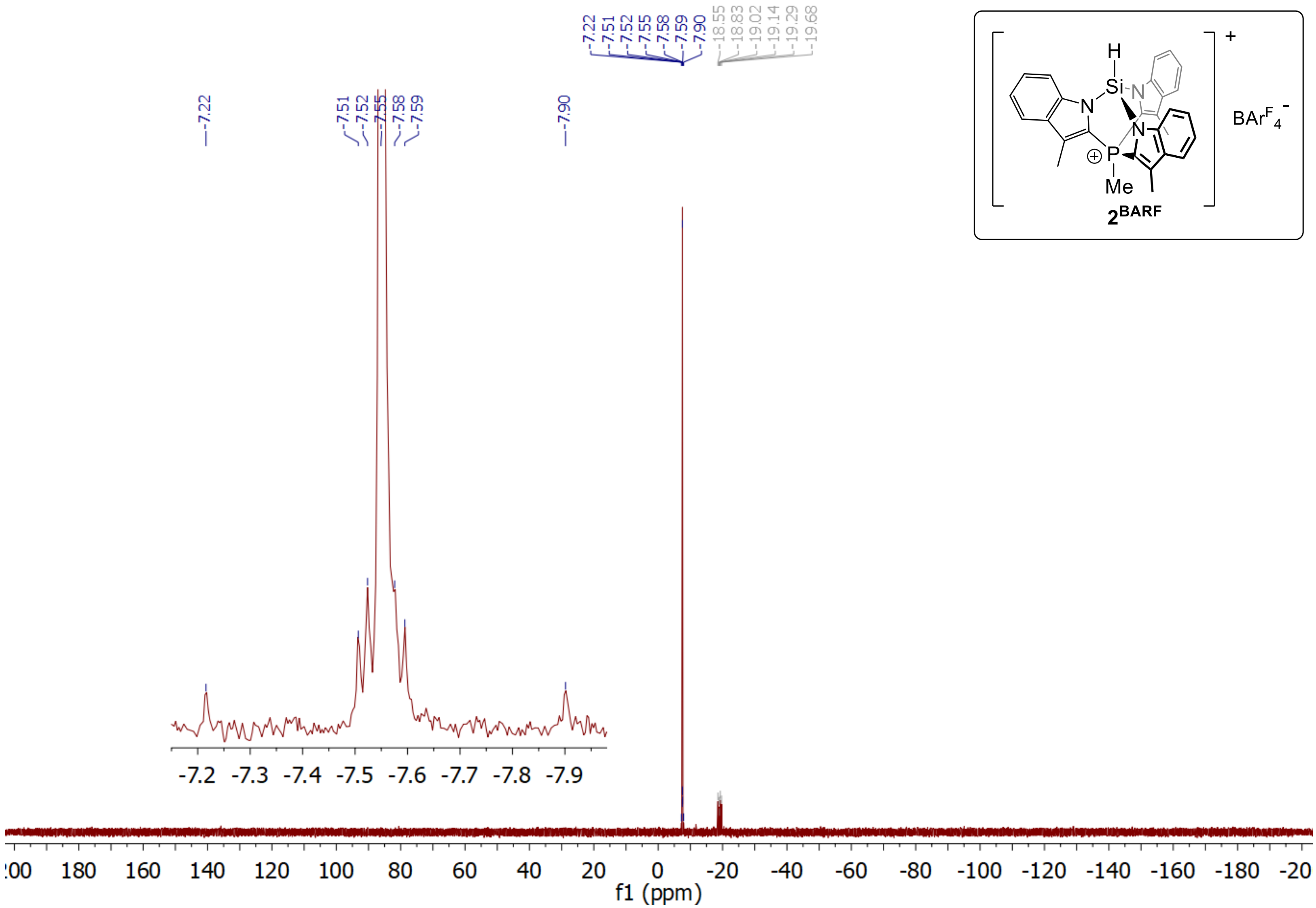

Figure S45. $^{31}P\{^1H\}$ NMR (162 MHz) spectrum of compound $\mathbf{2^{BARF}}$ in DCM-d_2. Minor peaks are unidentified impurities.

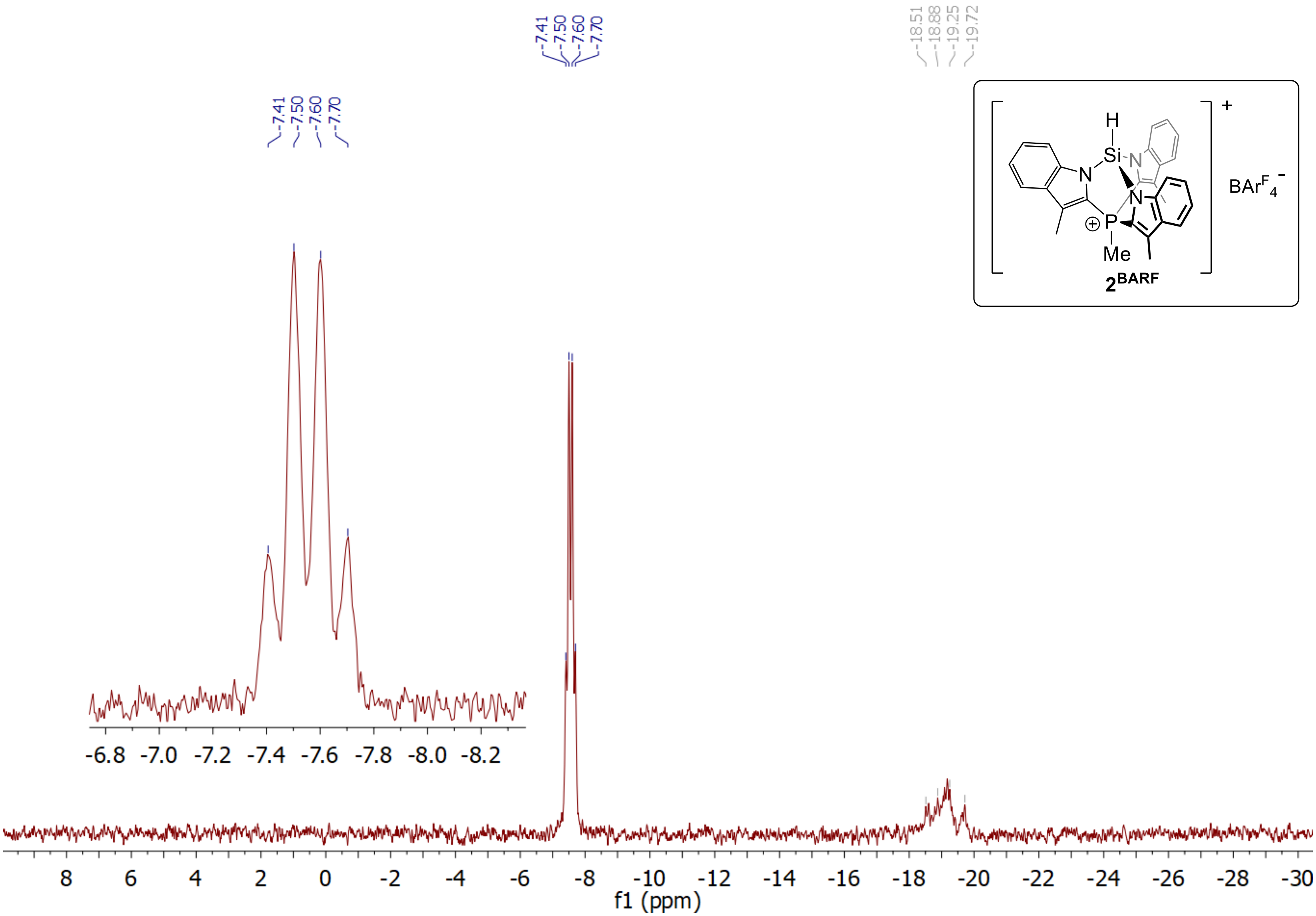

Figure S46. ^{31}P NMR (162 MHz) spectrum of compound **2**$^{\mathbf{BARF}}$ in DCM-d_2. Minor peaks are unidentified impurities.

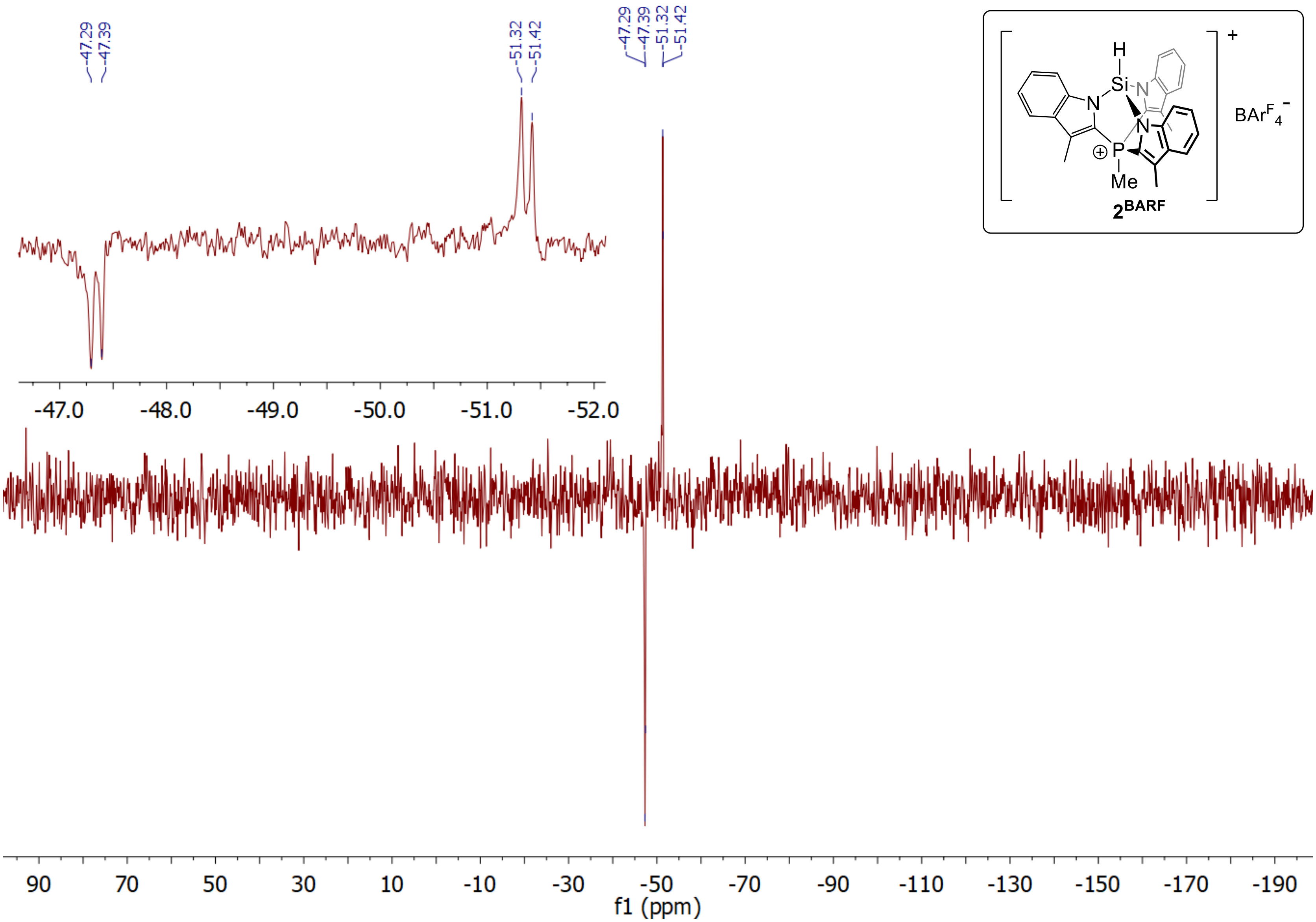

Figure S47. Non-refocused ^{29}Si INEPT (79 MHz) spectrum of compound **2^{BARF}** in DCM-d_2.

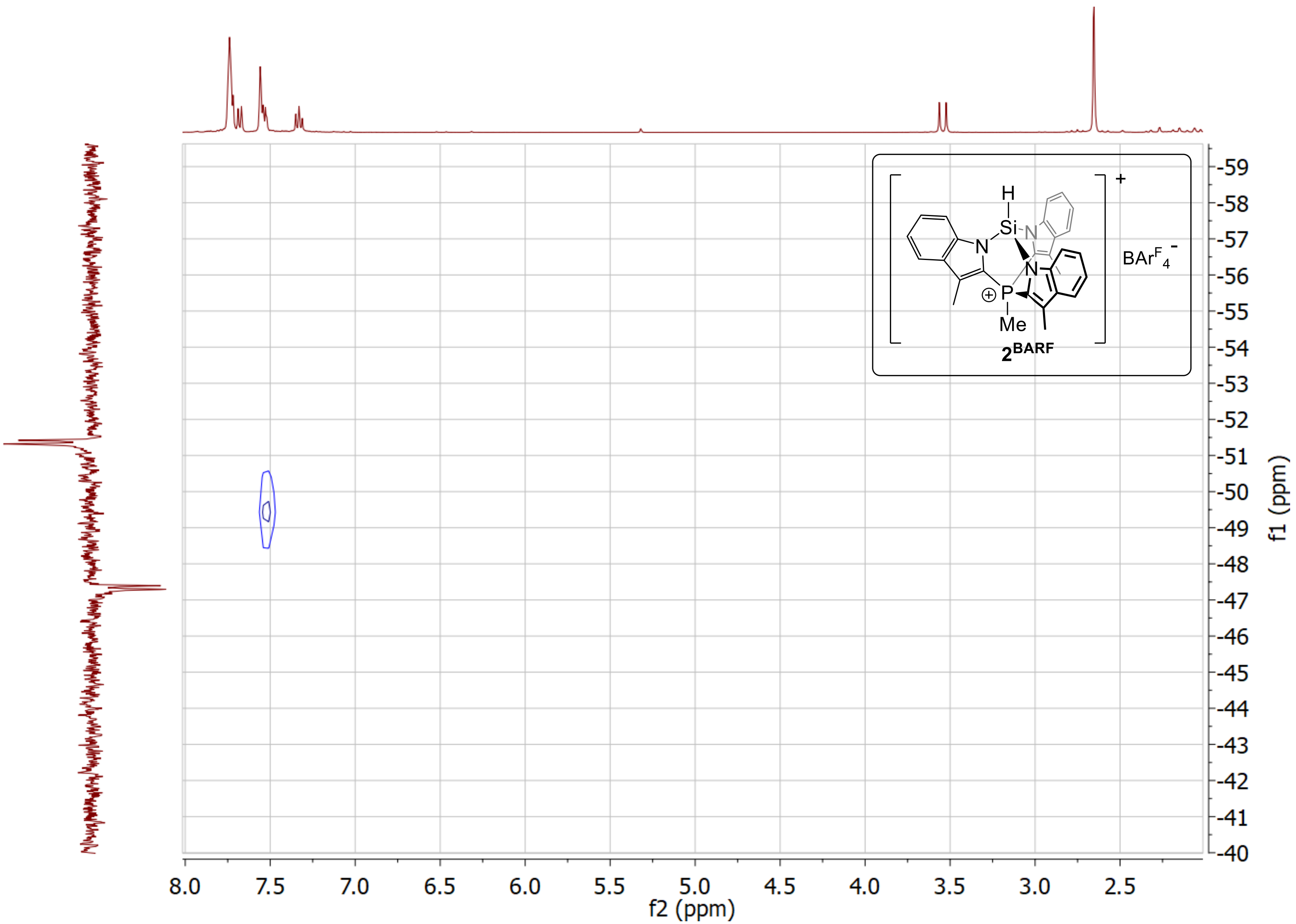

Figure S48. ^{1}H-^{29}Si ASAPHMQC spectrum of compound **2^{BARF}** in DCM-d_2.

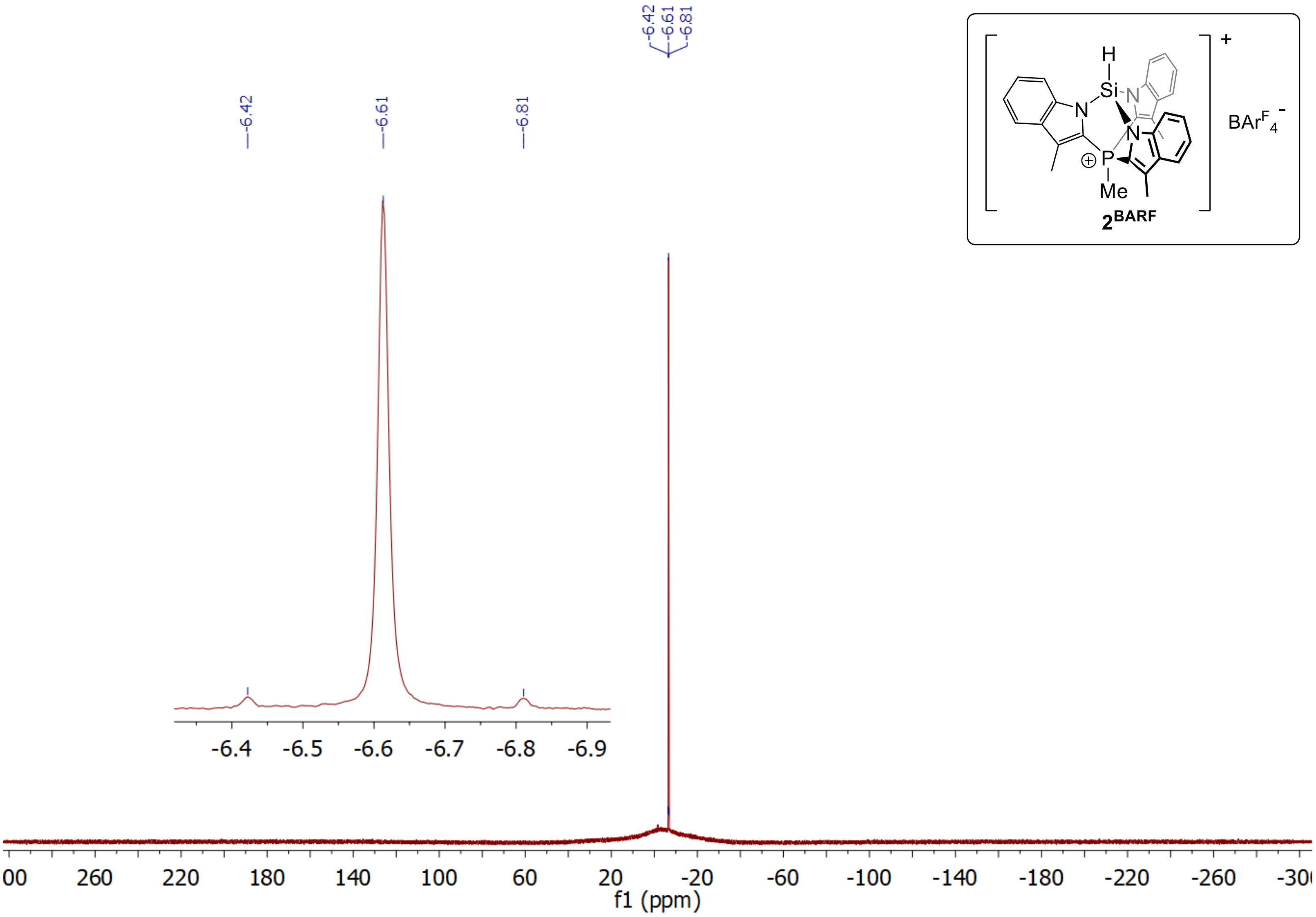

Figure S49. $^{11}B\{^{1}H\}$ NMR (128 MHz) spectrum of compound **2**$^{\mathbf{BARF}}$ in DCM-d_2.

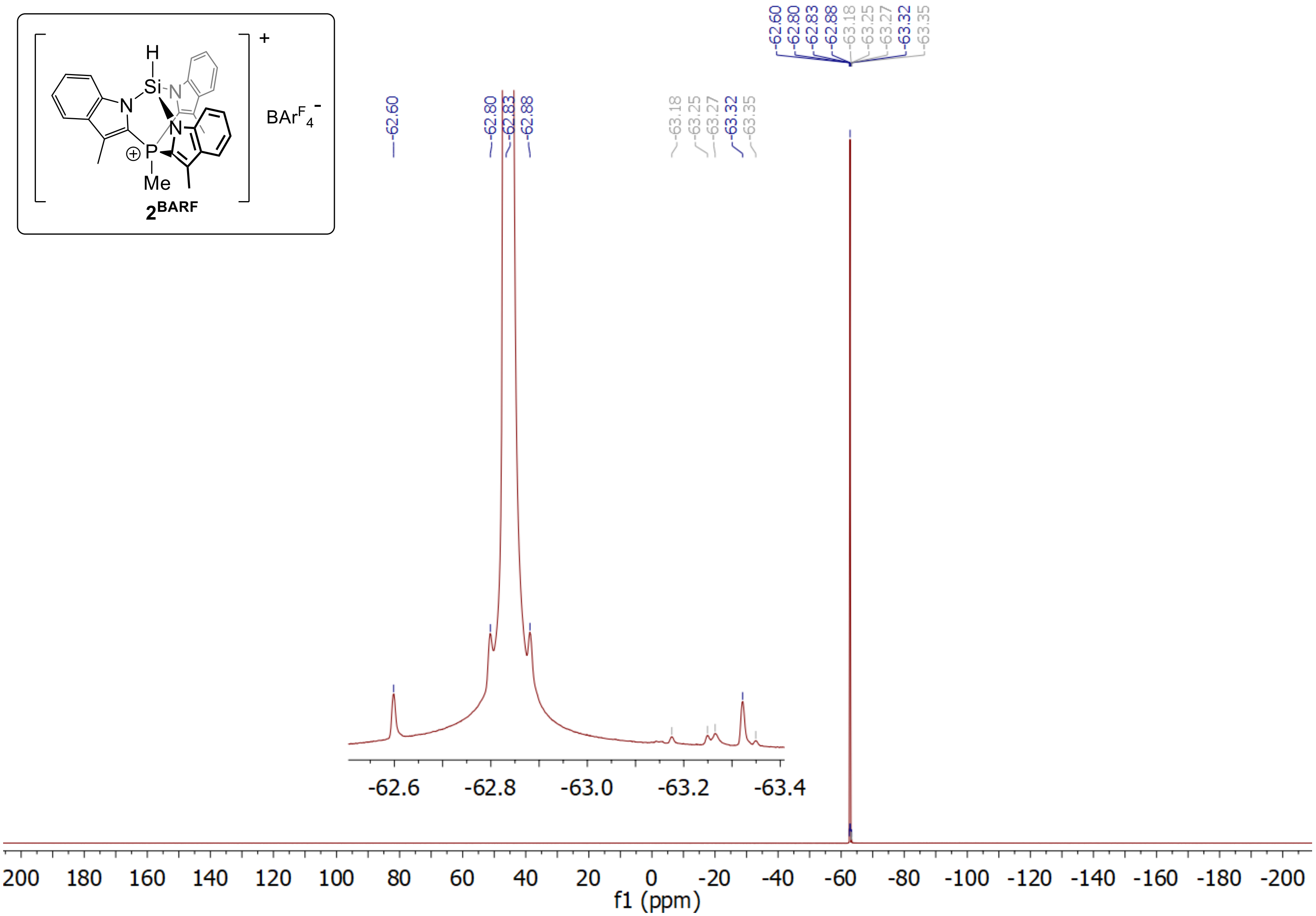

Figure S50. ^{19}F NMR (376 MHz) spectrum of compound **2**$^{\mathbf{BARF}}$ in DCM-d_2. Minor peaks are unidentified impurities.

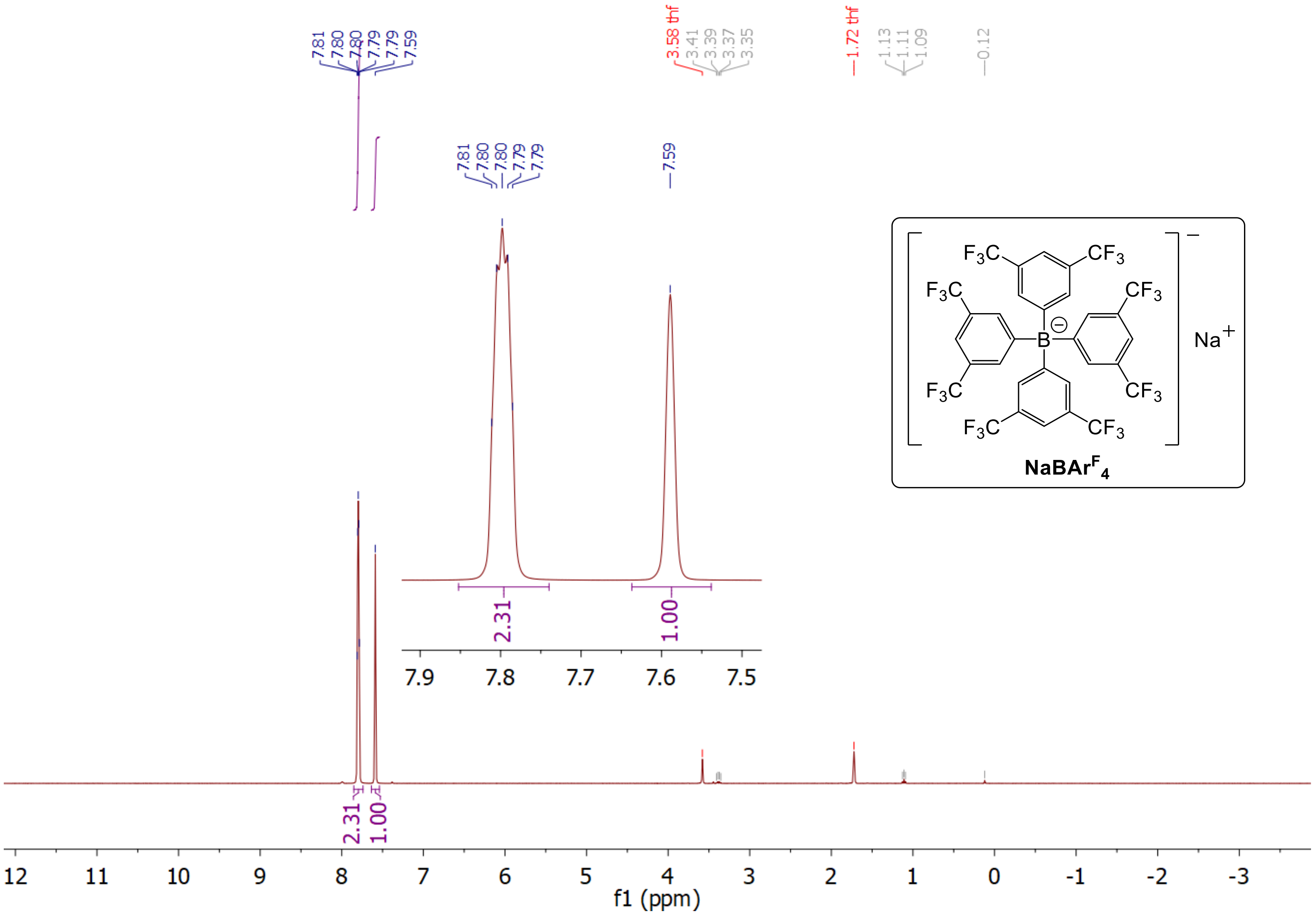

Figure S51. ^{1}H NMR (400 MHz) spectrum of $NaBAr^{F}_{4}$ in THF-d_8: δ 7.85 – 7.74 (m, 2H, C(2)^{Ar}-H), 7.59 (s, 1H, C(4)^{Ar}-H). Impurities: 0.12 ppm – grease, 1.09-1.13, 3.35-3.41 ppm – traces of diethyl ether.

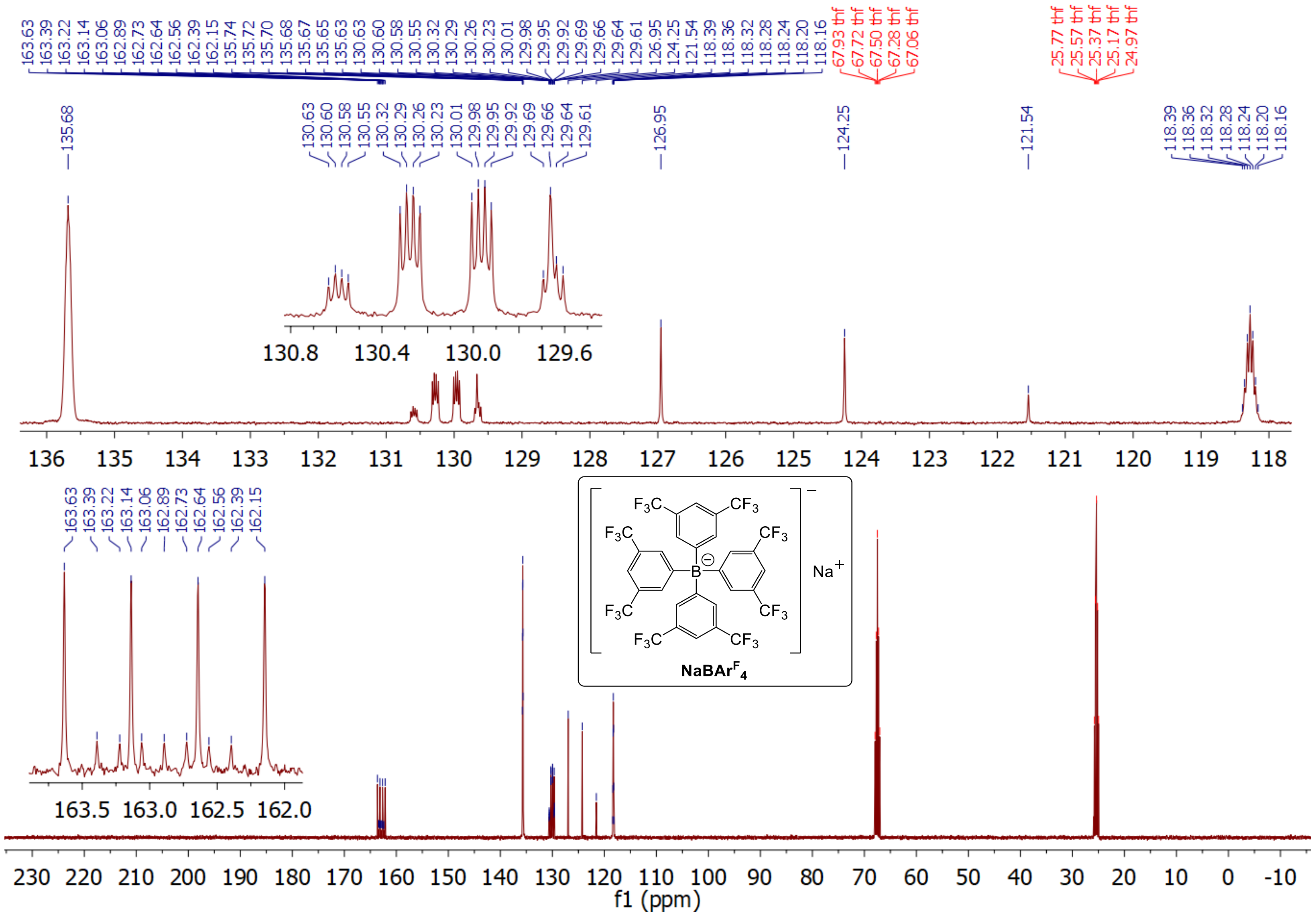

Figure S52. $^{13}C\{^{1}H\}$ NMR (101 MHz) spectrum of $NaBAr^{F}_4$ in THF-d_8: δ 162.9 (hept, $J_{C,B}$ = 16.7 Hz, C^{Ar}-^{10}B), 162.9 (q, $J_{C,B}$ = 49.8 Hz, C^{Ar}-^{11}B), 135.7 (br.s., $C(2)^{Ar}$-H), 130.1 (qq, $J_{C,F}$ = 31.5, 2.8 Hz, $\underline{C(3)^{Ar}}$-CF_3), 125.6 (q, $J_{C,F}$ = 272.3 Hz, CF_3), 118.3 (hept, $J_{C,F}$ = 3.5 Hz, $C(4)^{Ar}$-H).

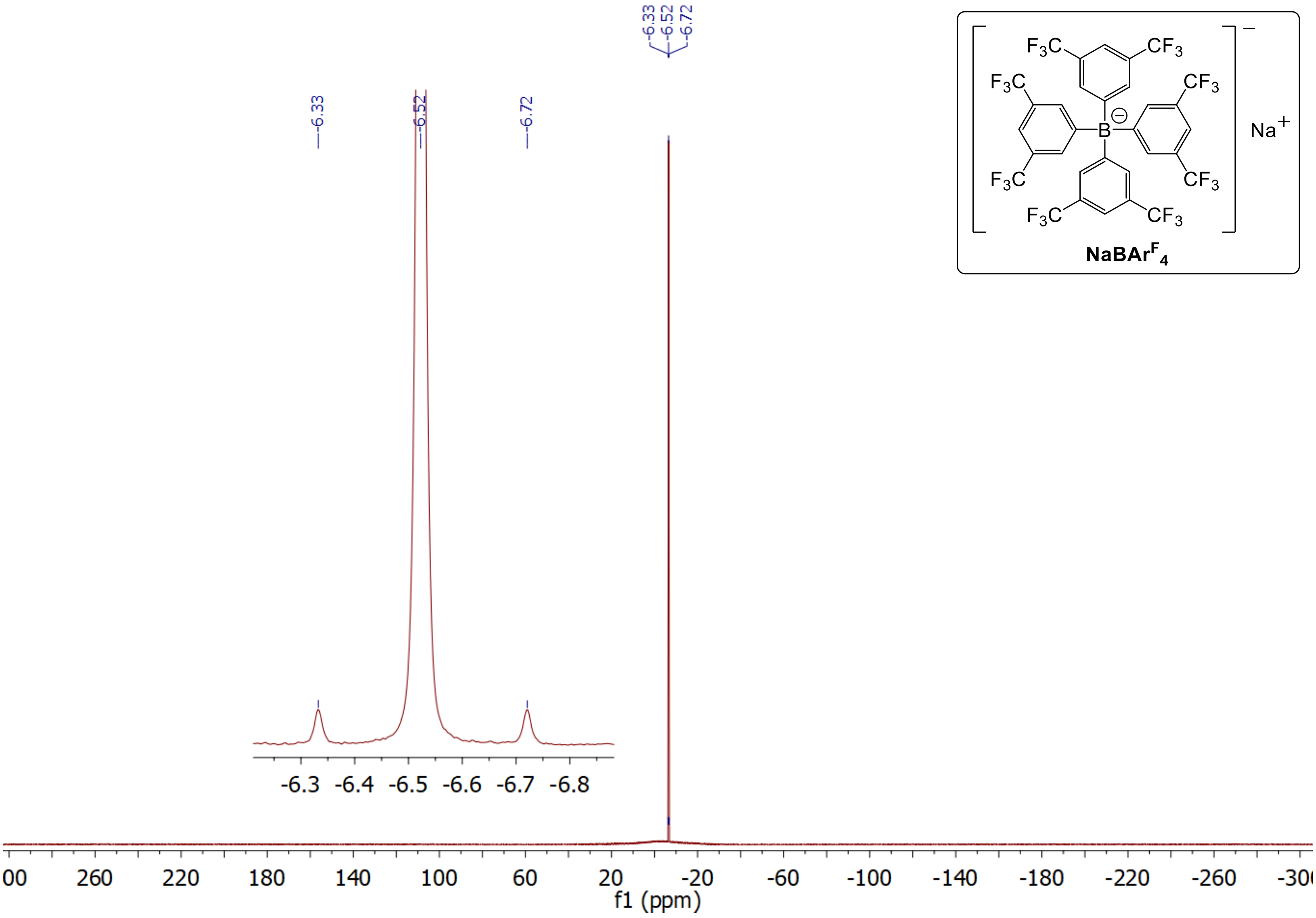

Figure S53. $^{11}B\{^1H\}$ (128 MHz) NMR spectrum of NaBAr$^F{}_4$ in THF-d_8: δ -6.52, ^{13}C-satellites at -6.53 (d, $J_{B,C}$ = 49.9 Hz).

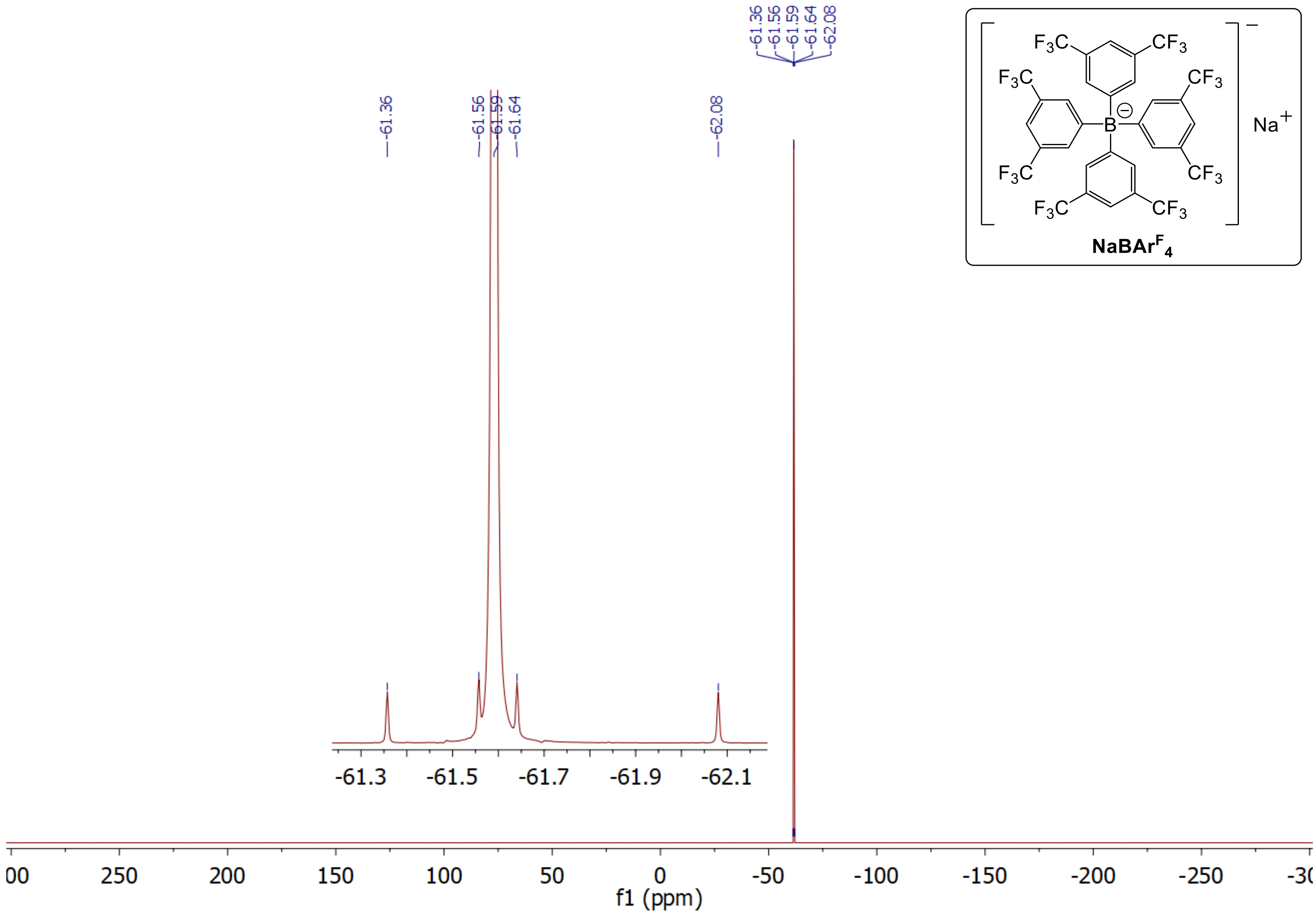

Figure S54. ^{19}F NMR (376 MHz) spectrum of NaBAr$^{F}{}_{4}$ in THF-d_8: δ -61.59, ^{13}C-satellites at -61.60 (d, $J_{F,C}$ = 31.4 Hz) and -61.72 (d, $J_{F,C}$ = 272.2 Hz).

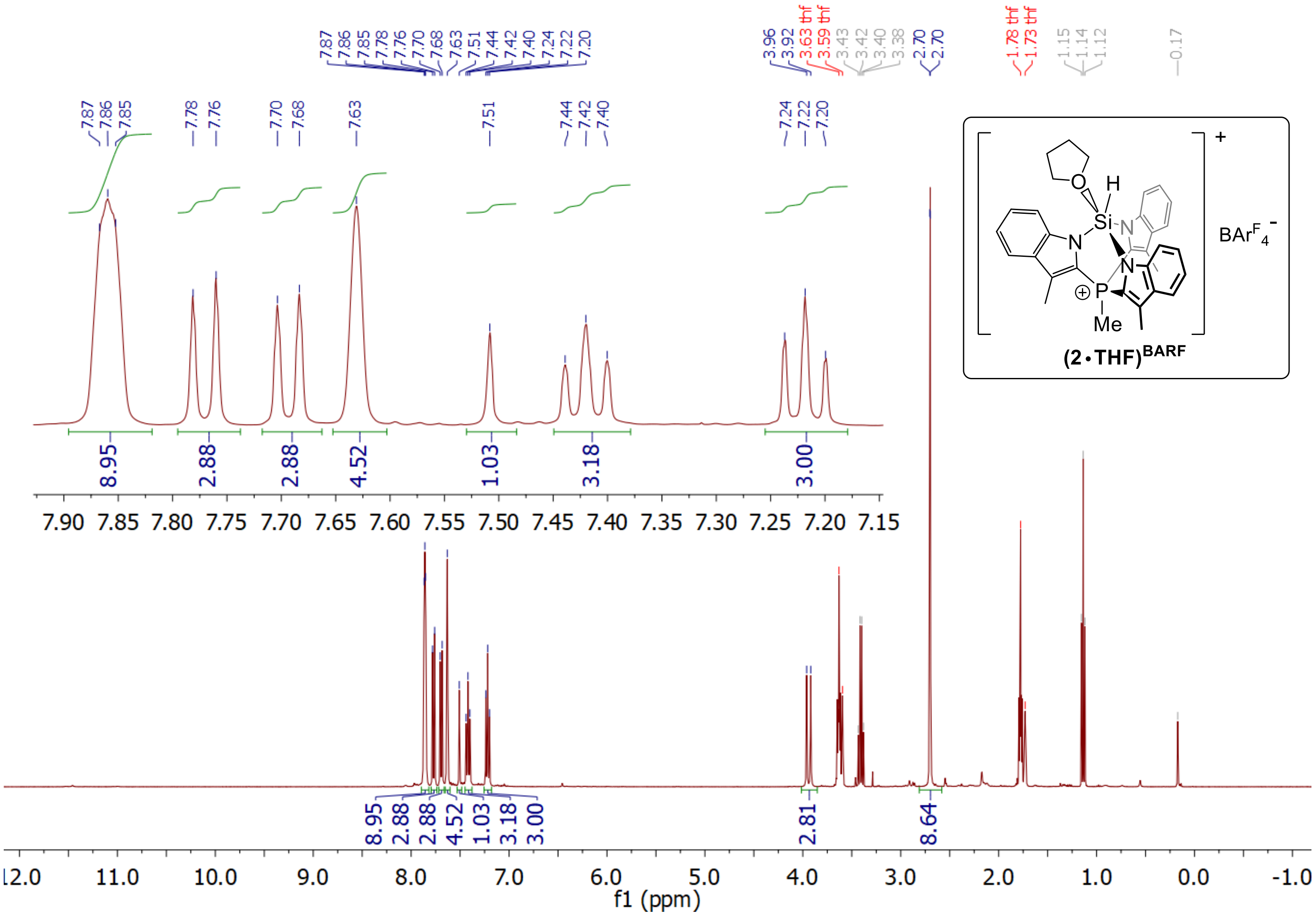

Figure S55. ^{1}H NMR (400 MHz) spectrum of compound **2**$^{\mathbf{BARF}}$ in THF-d_8. Impurities: 0.17 ppm – grease, 1.12-1.15 and 3.38-3.43 ppm – diethyl ether, other minor peaks are unidentified. Increased intensity of $BAr^F{}_4^-$ -derived peaks at 7.63 and 7.86 ppm suggests the presence of a small amount (ca. 10%) of a contaminating $BAr^F{}_4^-$ salt, possibly $NaBAr^F{}_4$ or $HBAr^F{}_4$.

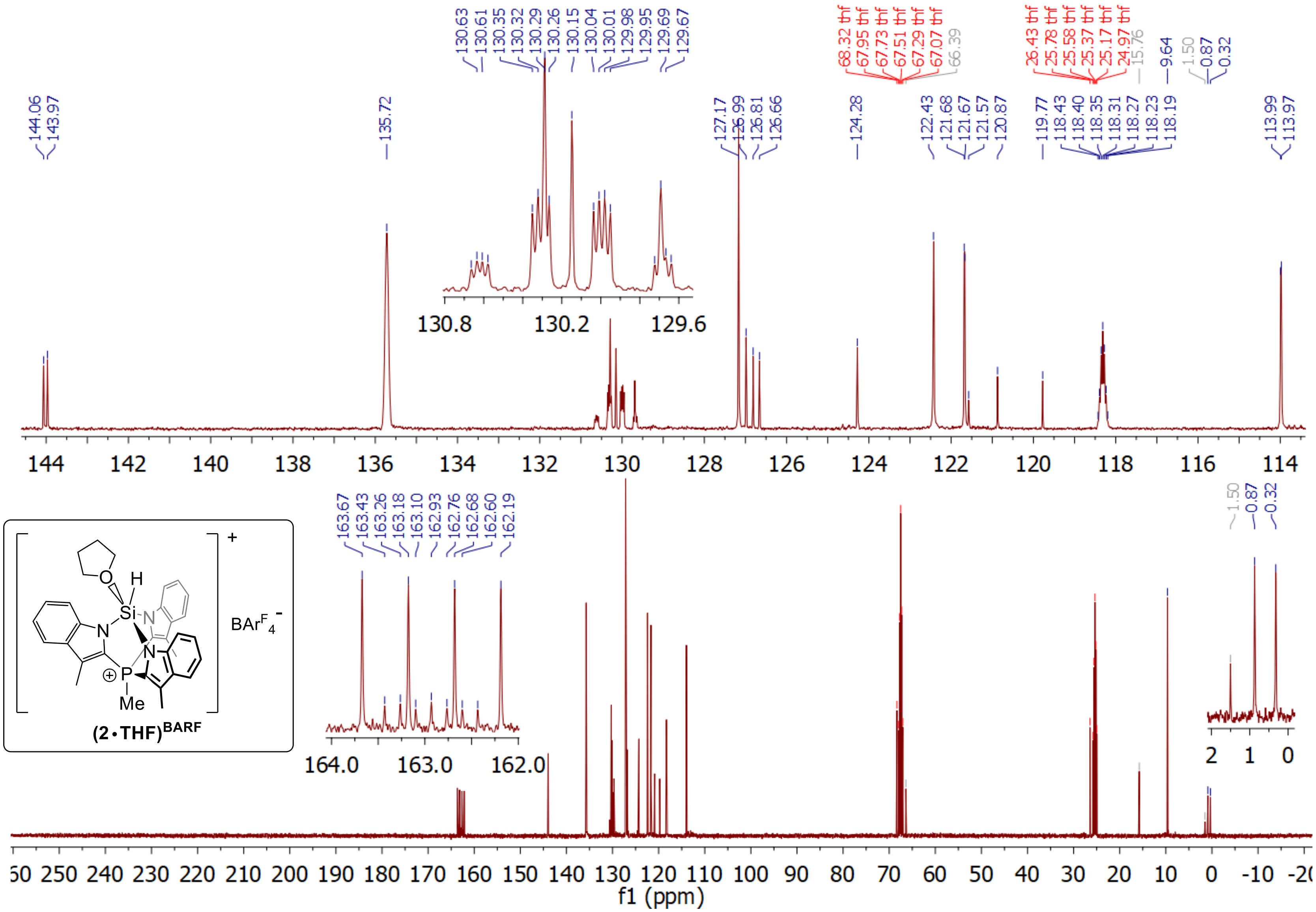

Figure S56. $^{13}C\{^1H\}$ NMR (101 MHz) spectrum of compound **2**^{BARF} in THF-d_8. Impurities: 1.50 ppm – grease, 15.76 and 66.39 ppm – diethyl ether.

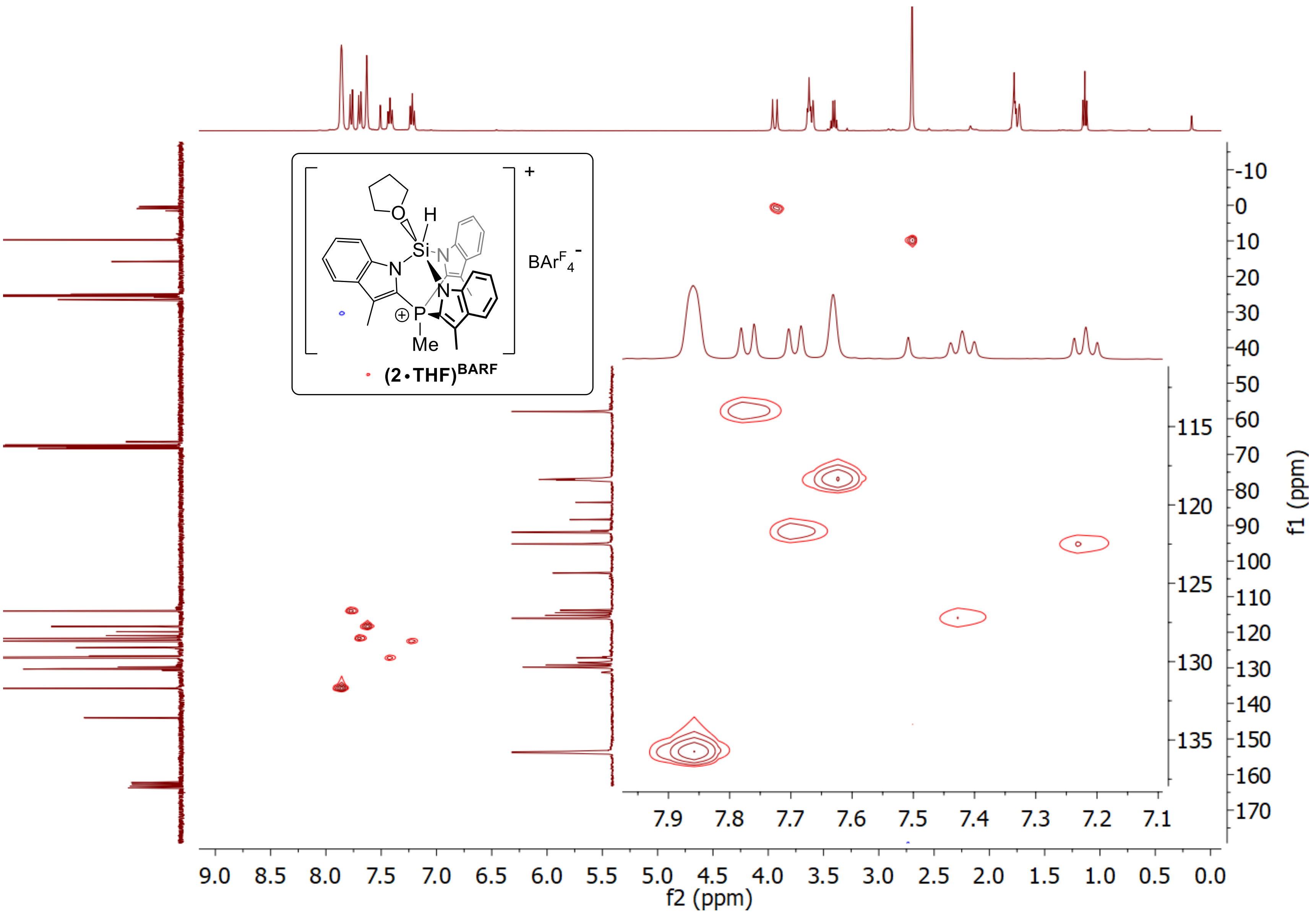

Figure S57. ^{1}H-^{13}C ASAPHMQC spectrum of compound **2^{BARF}** in THF-d_8.

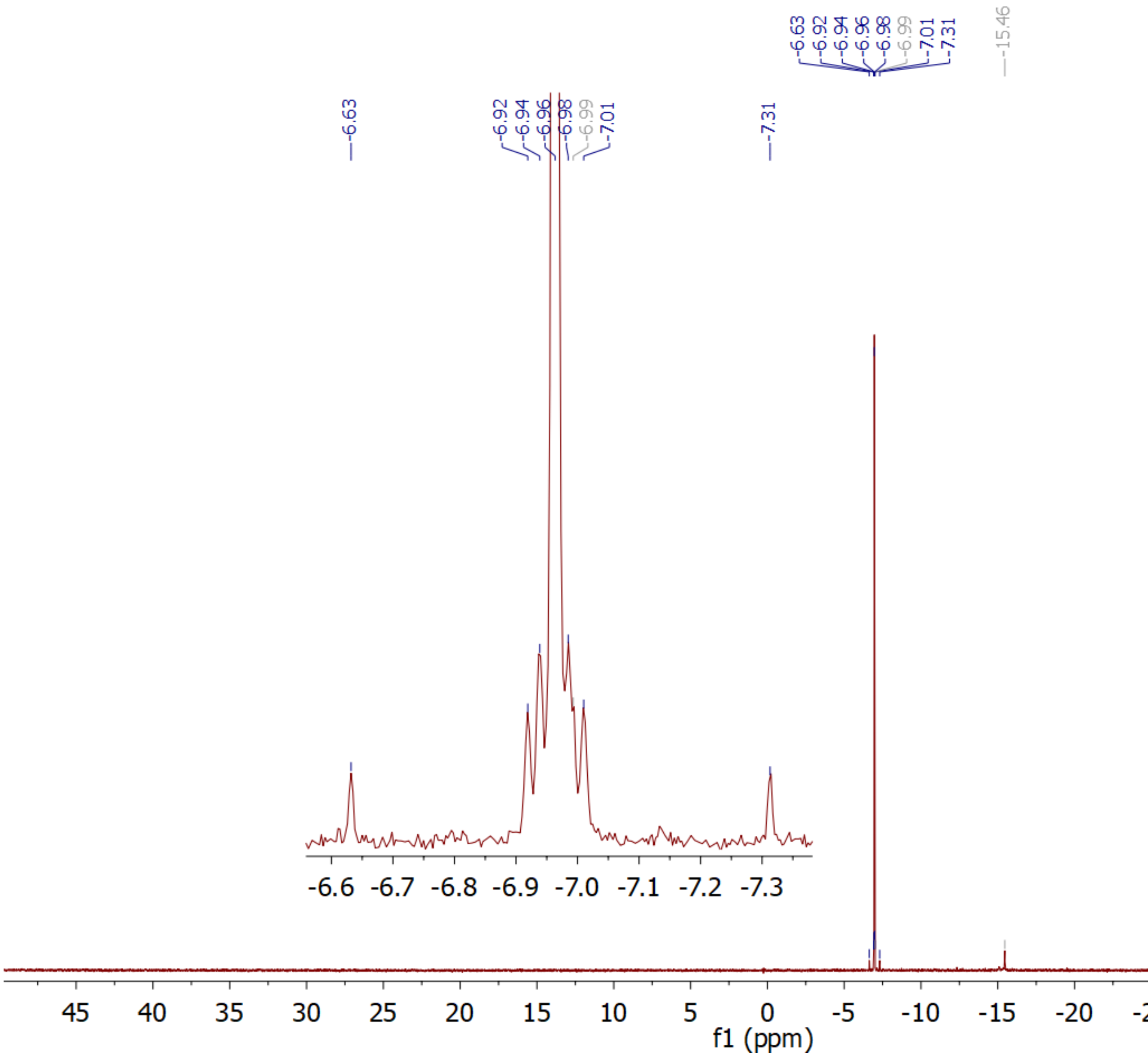

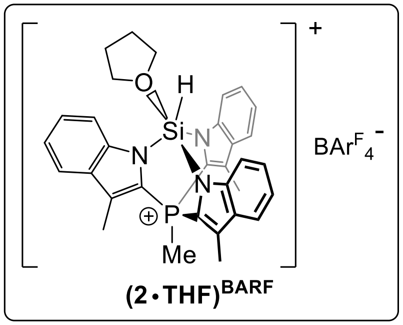

Figure S58. $^{31}P\{^{1}H\}$ NMR (162 MHz) spectrum of compound $\mathbf{2}^{\mathbf{BARF}}$ in THF-d_8. Impurities: -15.46 and -6.99 ppm – unidentified.

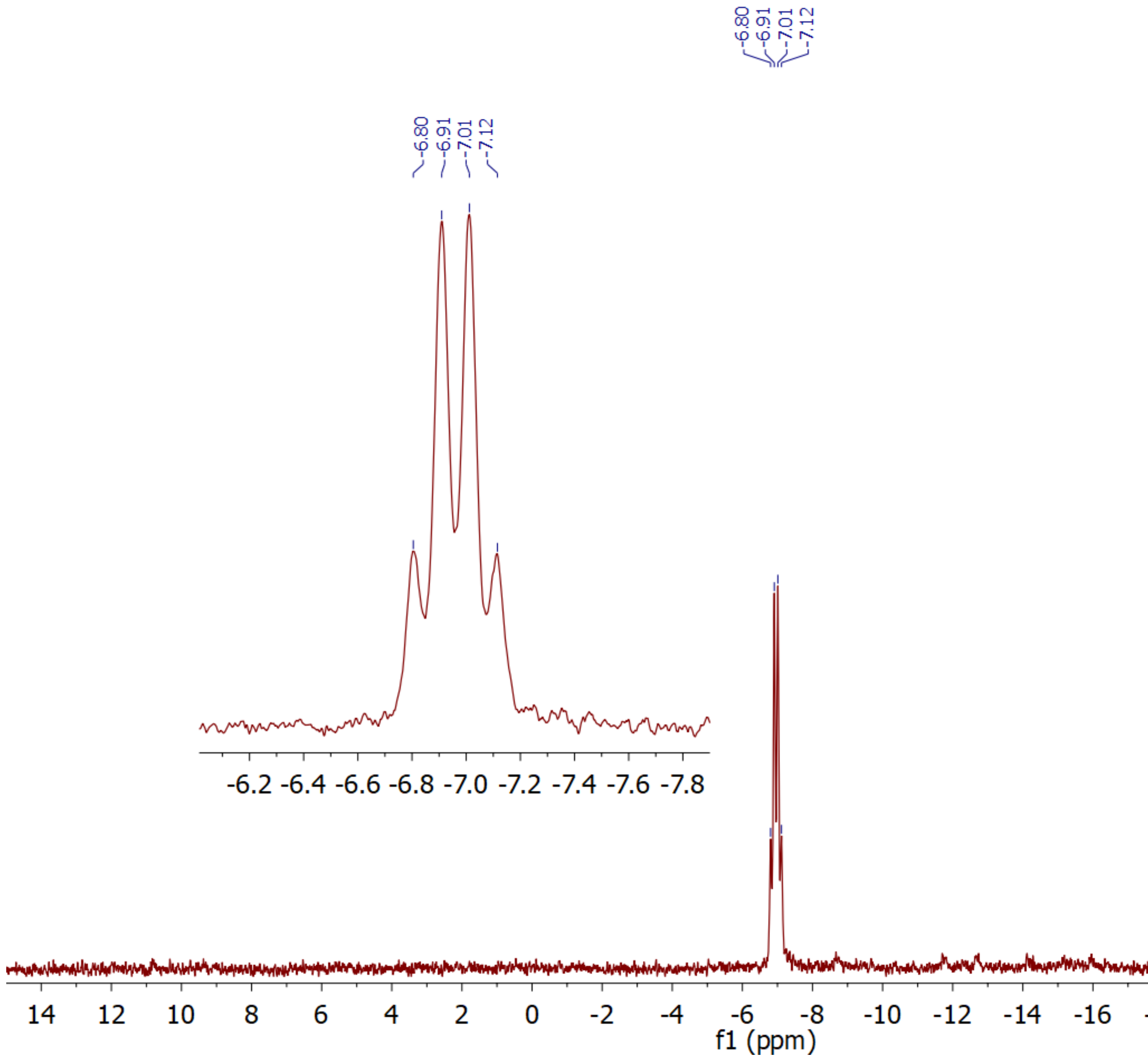

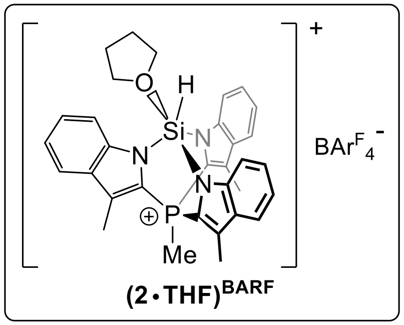

Figure S59. ^{31}P NMR (162 MHz) spectrum of compound **2^{BARF}** in THF-d_8.

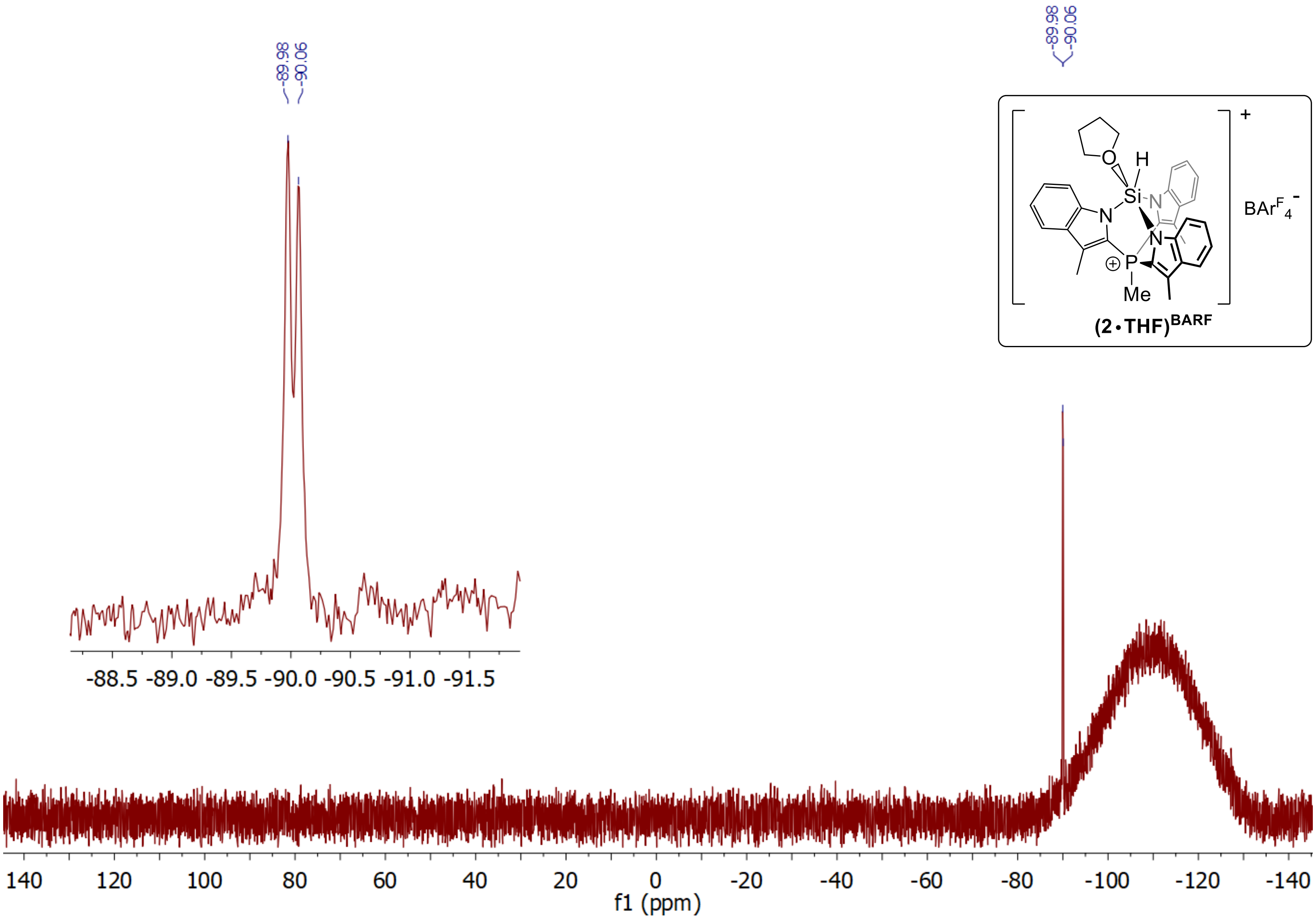

Figure S60. $^{29}Si\{^1H\}$ NMR (79 MHz) spectrum of compound **2**$^{\mathbf{BARF}}$ in THF-d_8.

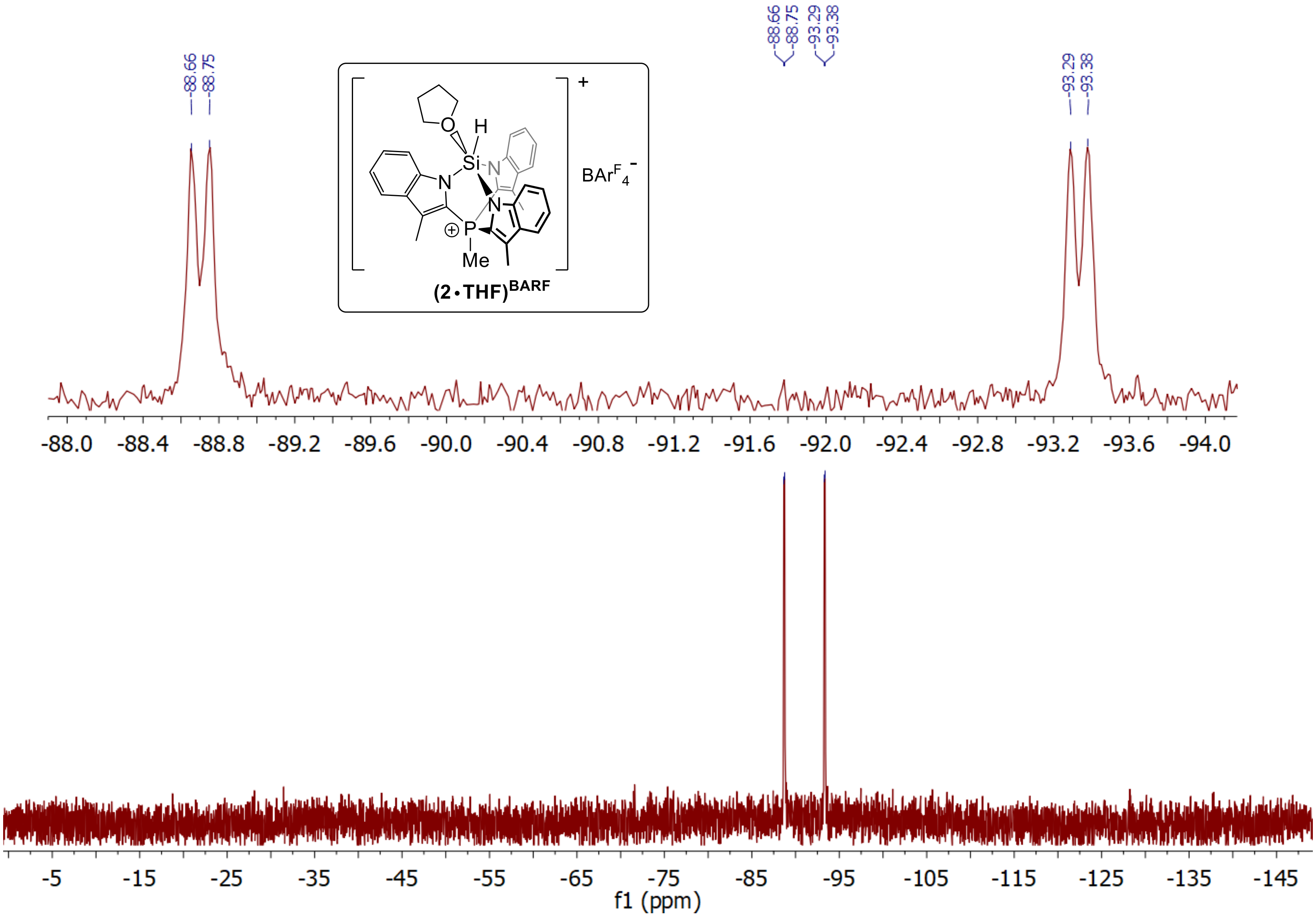

Figure S61. Refocused ^{29}Si INEPT (79 MHz) spectrum of compound **2**$^{\mathbf{BARF}}$ in THF-d_8.

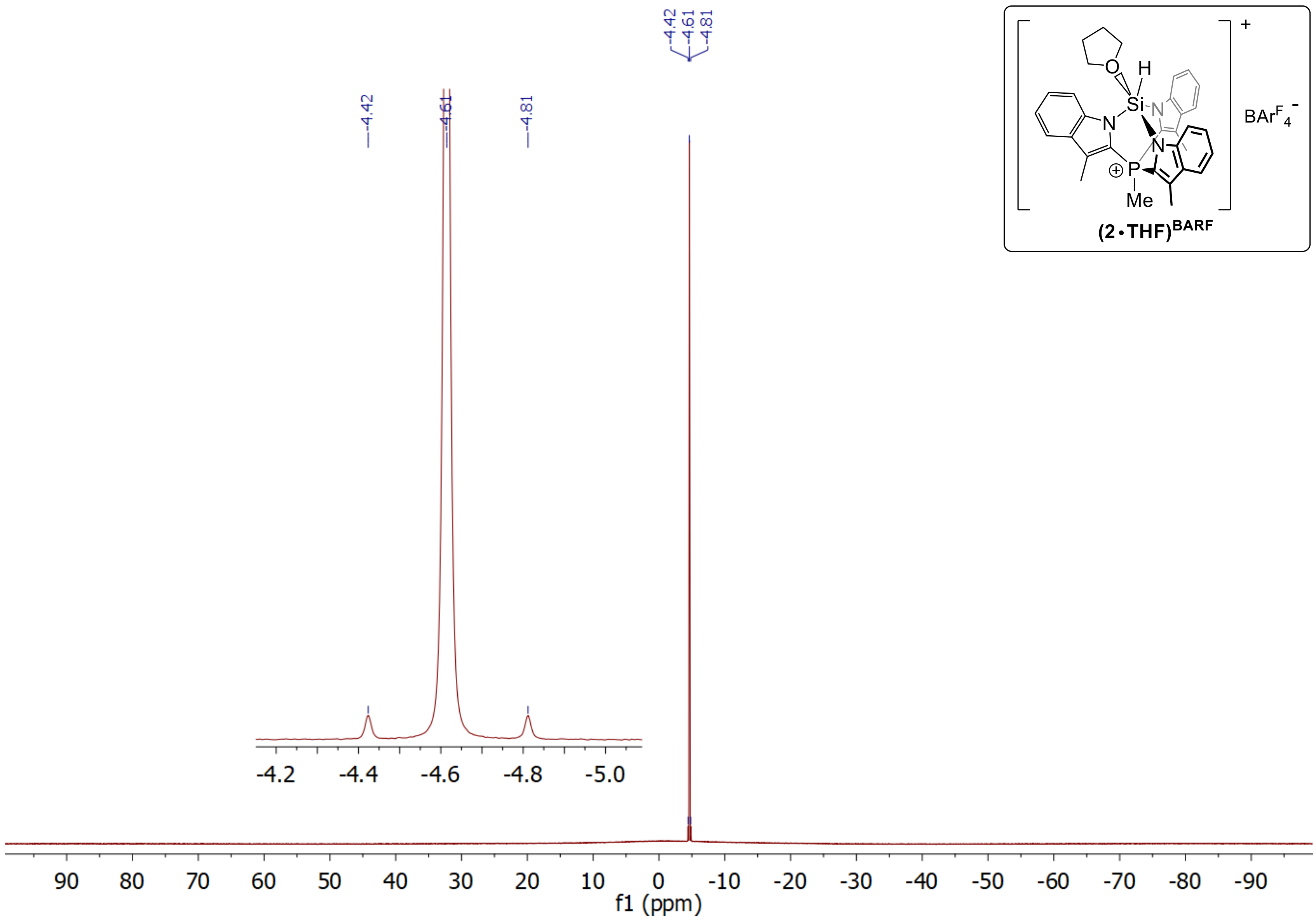

Figure S62. $^{11}B\{^1H\}$ NMR (128 MHz) spectrum of compound $\mathbf{2}^{\mathbf{BARF}}$ in THF-d_8.

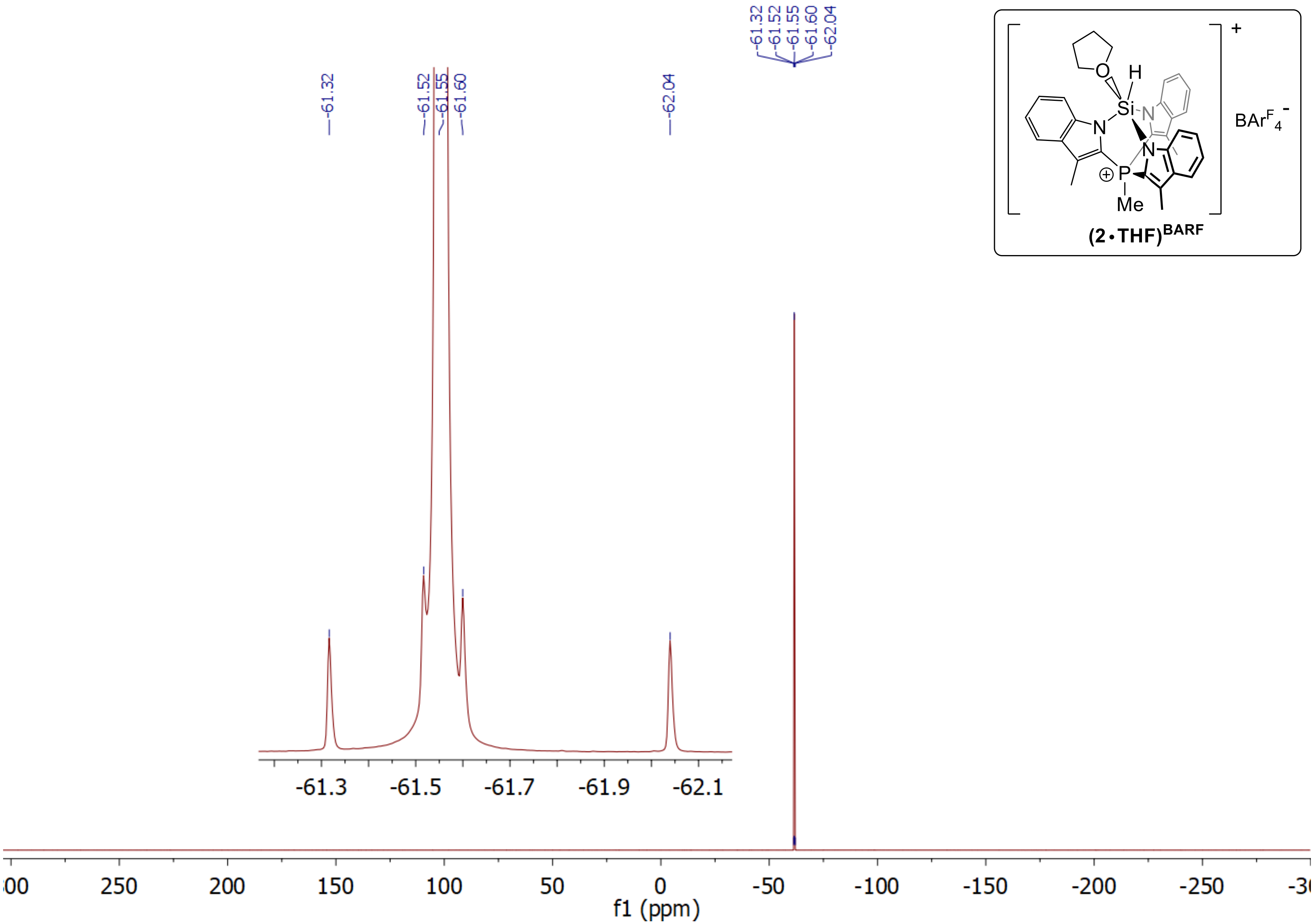

Figure S63. ¹⁹F NMR (376 MHz) spectrum of compound **2**[BARF] in THF-d_8.

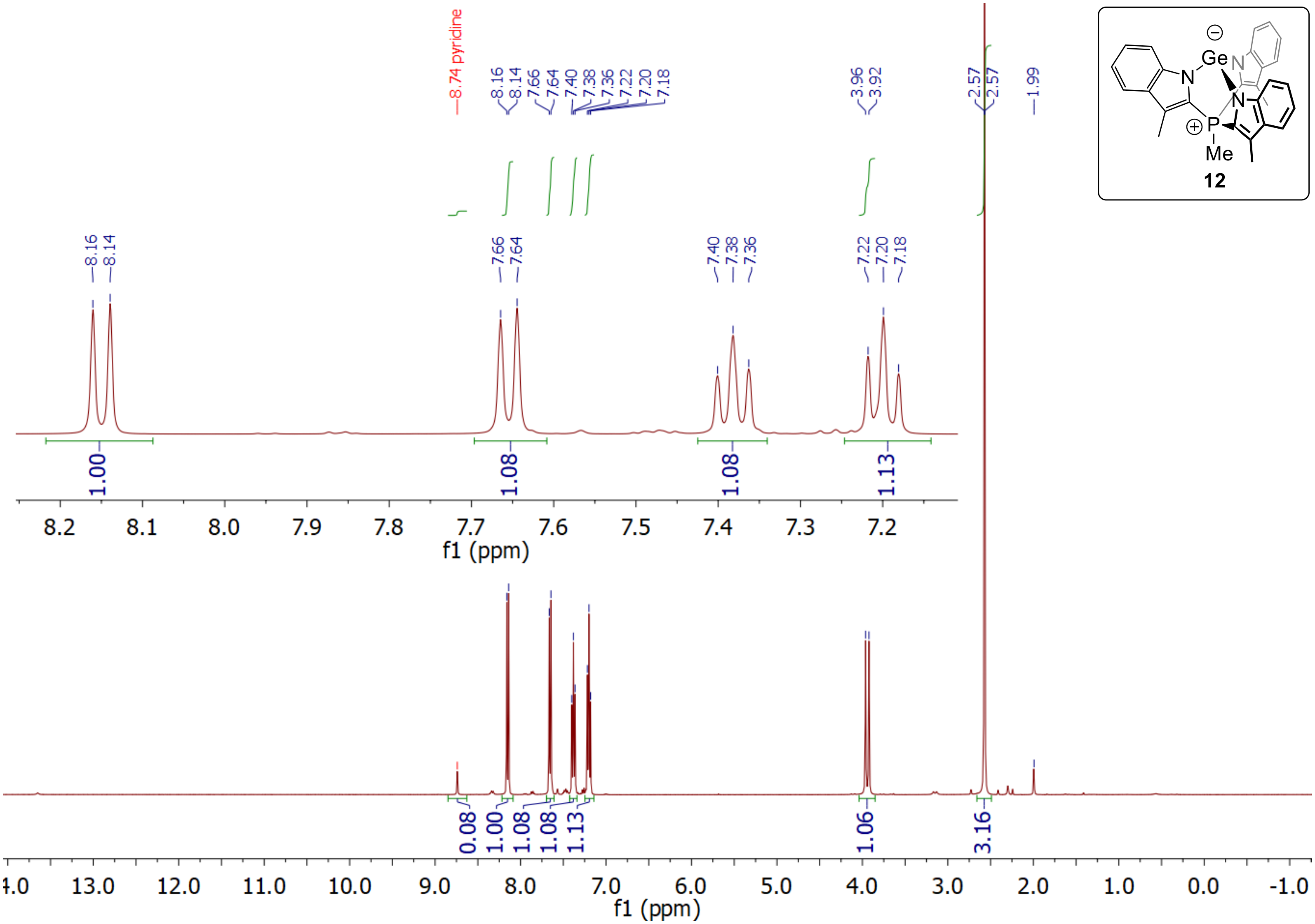

Figure S64. ^{1}H NMR (400 MHz) spectrum of compound **12** in pyridine-d_5. Impurities: 1.99 ppm – unidentified.

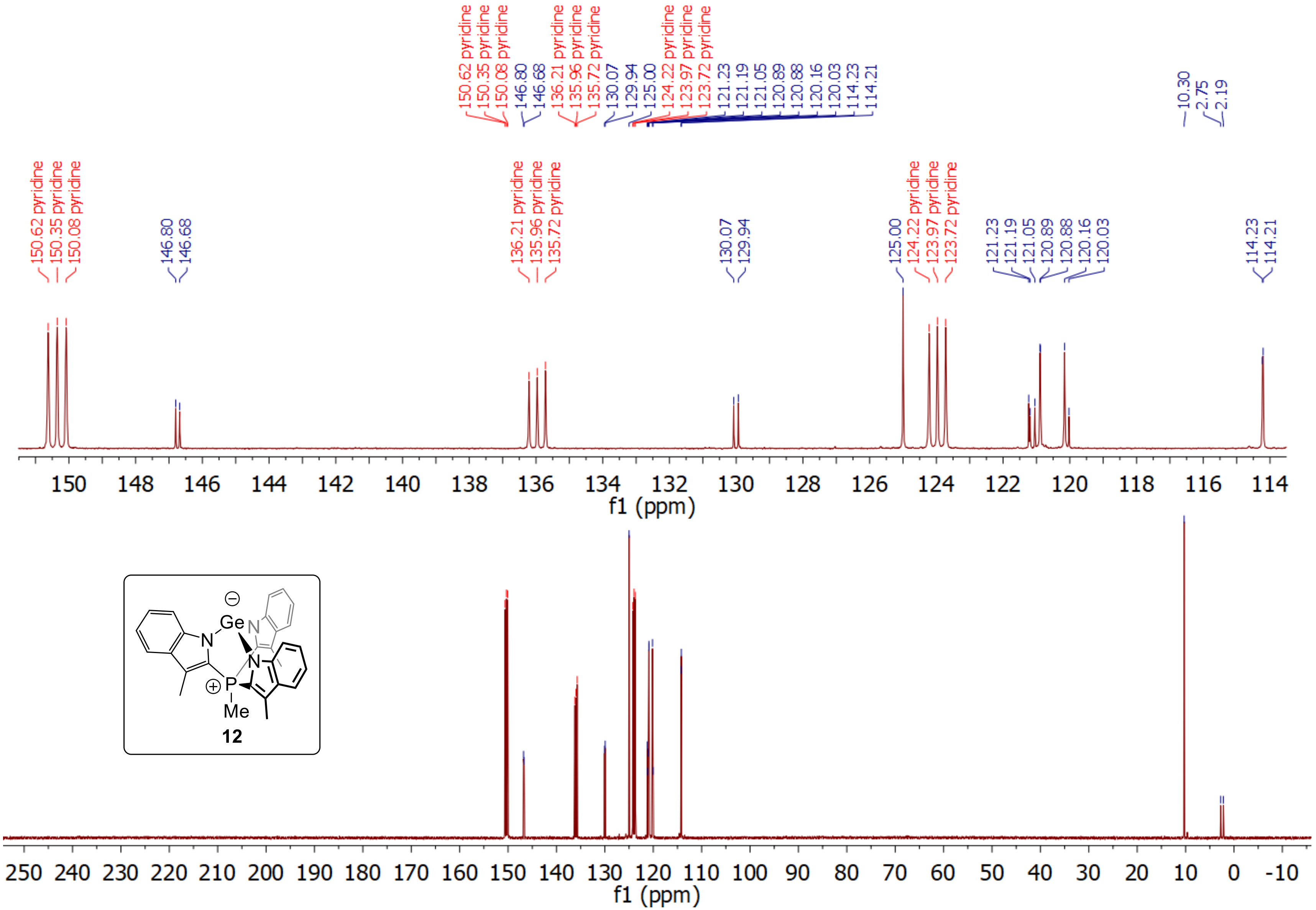

Figure S65. $^{13}C\{^{1}H\}$ NMR (101 MHz) spectrum of compound **12** in pyridine-d_5.

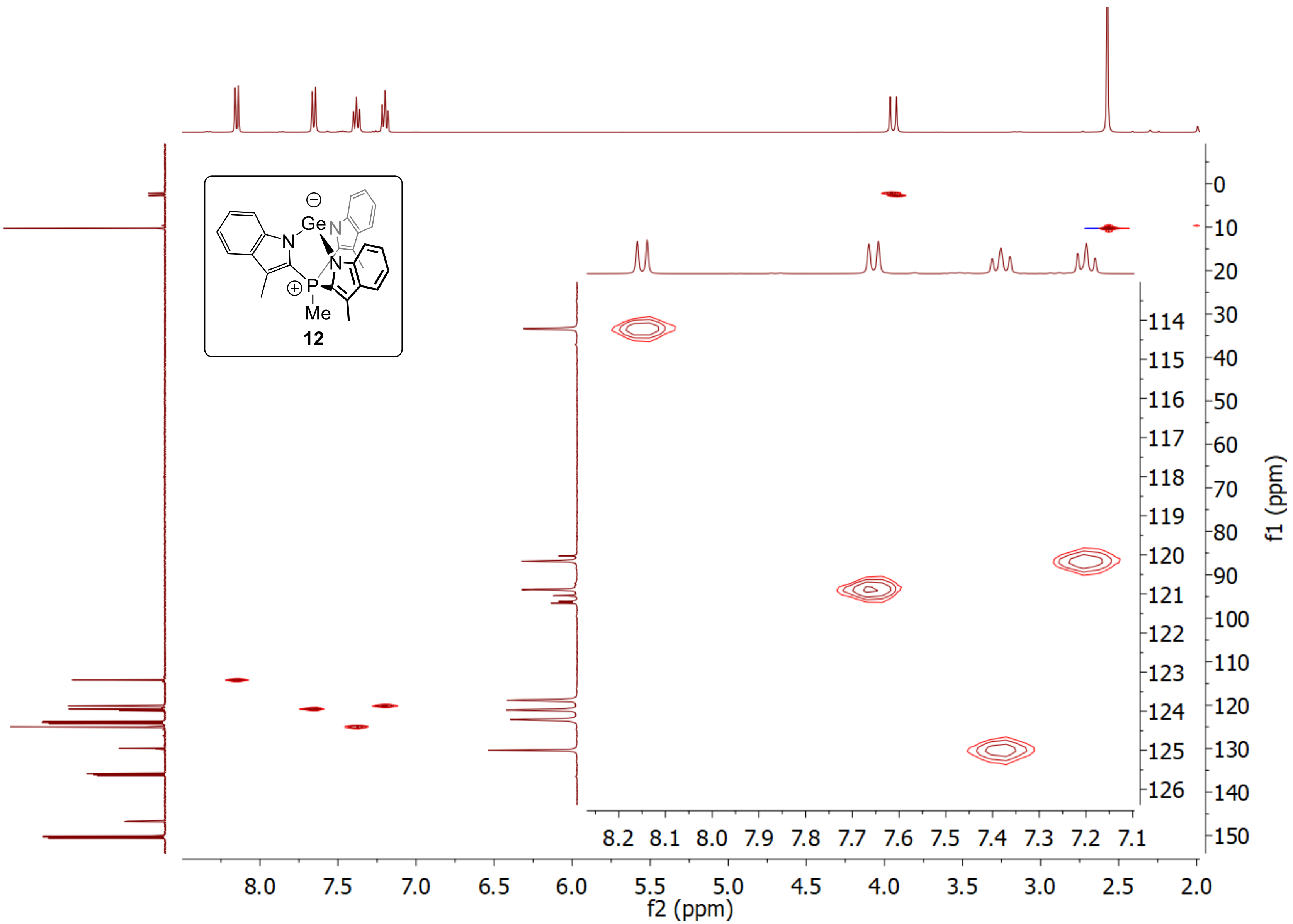

Figure S66. ^{1}H-^{13}C ASAPHMQC spectrum of compound **12** in pyridine-d_5.

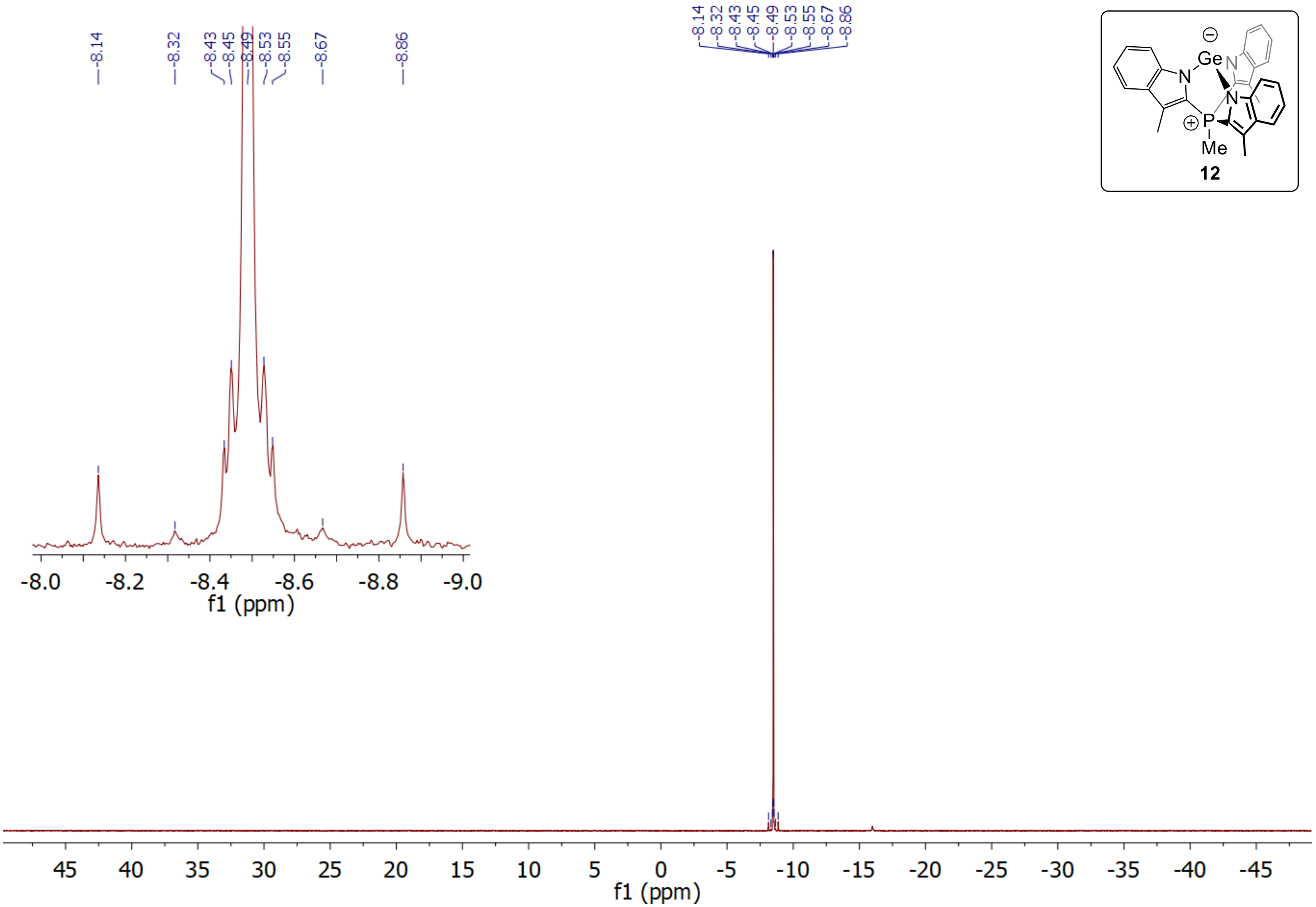

Figure S67. $^{31}P\{^{1}H\}$ NMR (162 MHz) spectrum of compound **12** in pyridine-d_5.

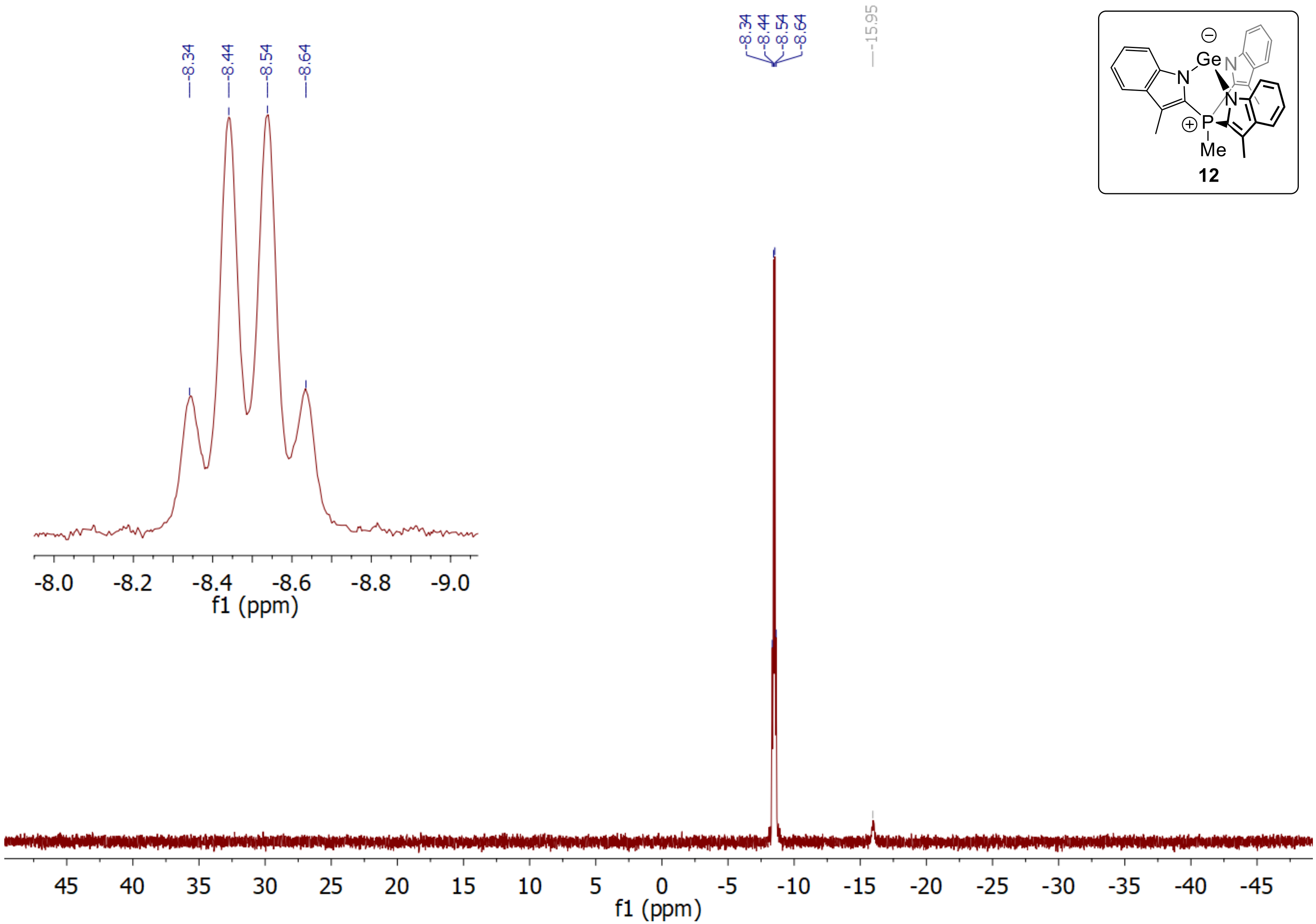

Figure S68. ^{31}P NMR (162 MHz) spectrum of compound **12** in pyridine-d_5. Impurities: -15.95 ppm – unidentified.

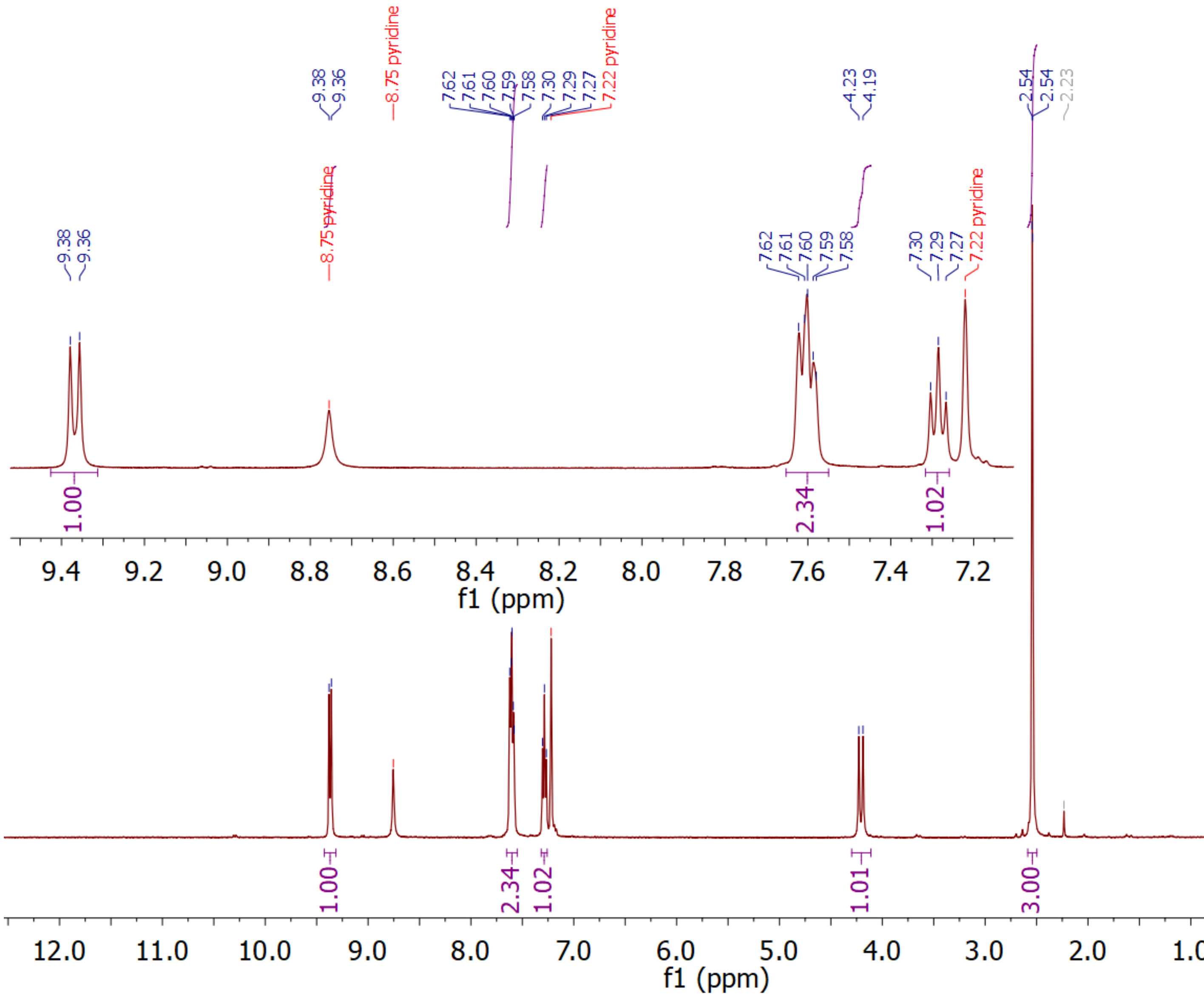

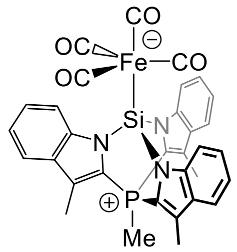

8

Figure S69. ^{1}H NMR (400 MHz) spectrum of compound **8** in pyridine-d_5. Impurities: 2.23 ppm – unidentified.

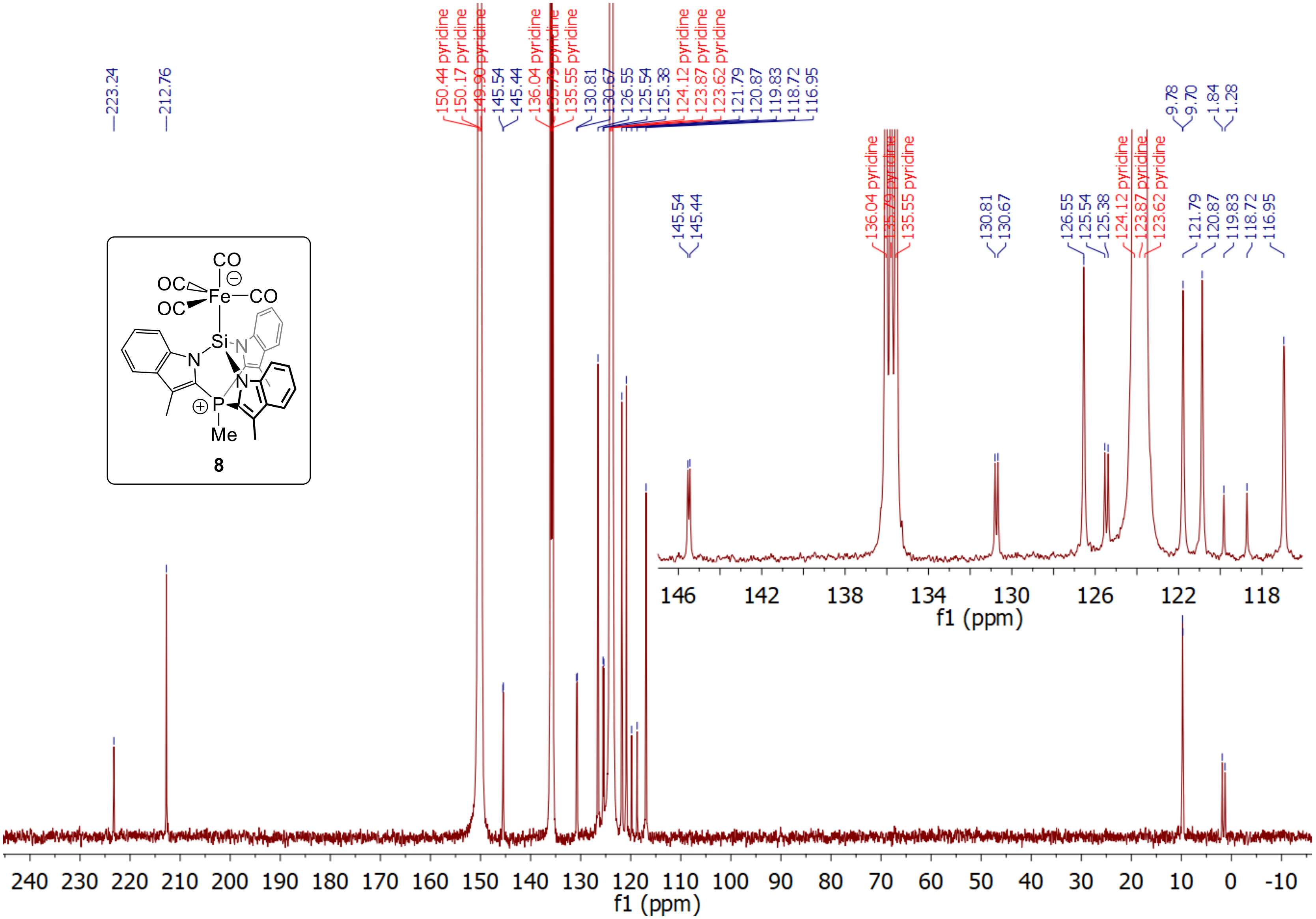

Figure S70. $^{13}C\{^{1}H\}$ NMR (101 MHz) spectrum of compound **8** in pyridine-d_5.

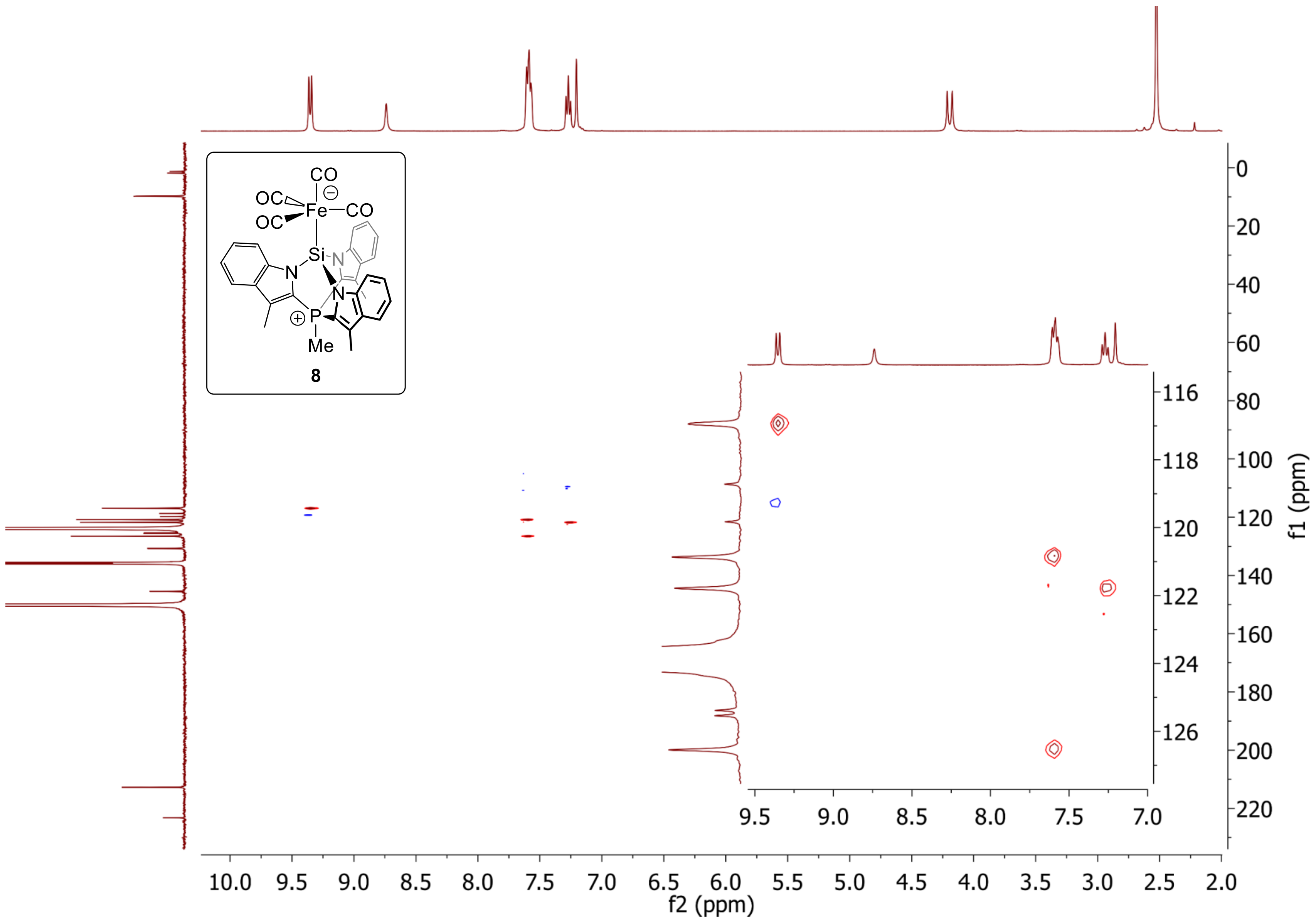

Figure S71. ^{1}H-^{13}C ASAPHMQC spectrum of compound **8** in pyridine-d_5.

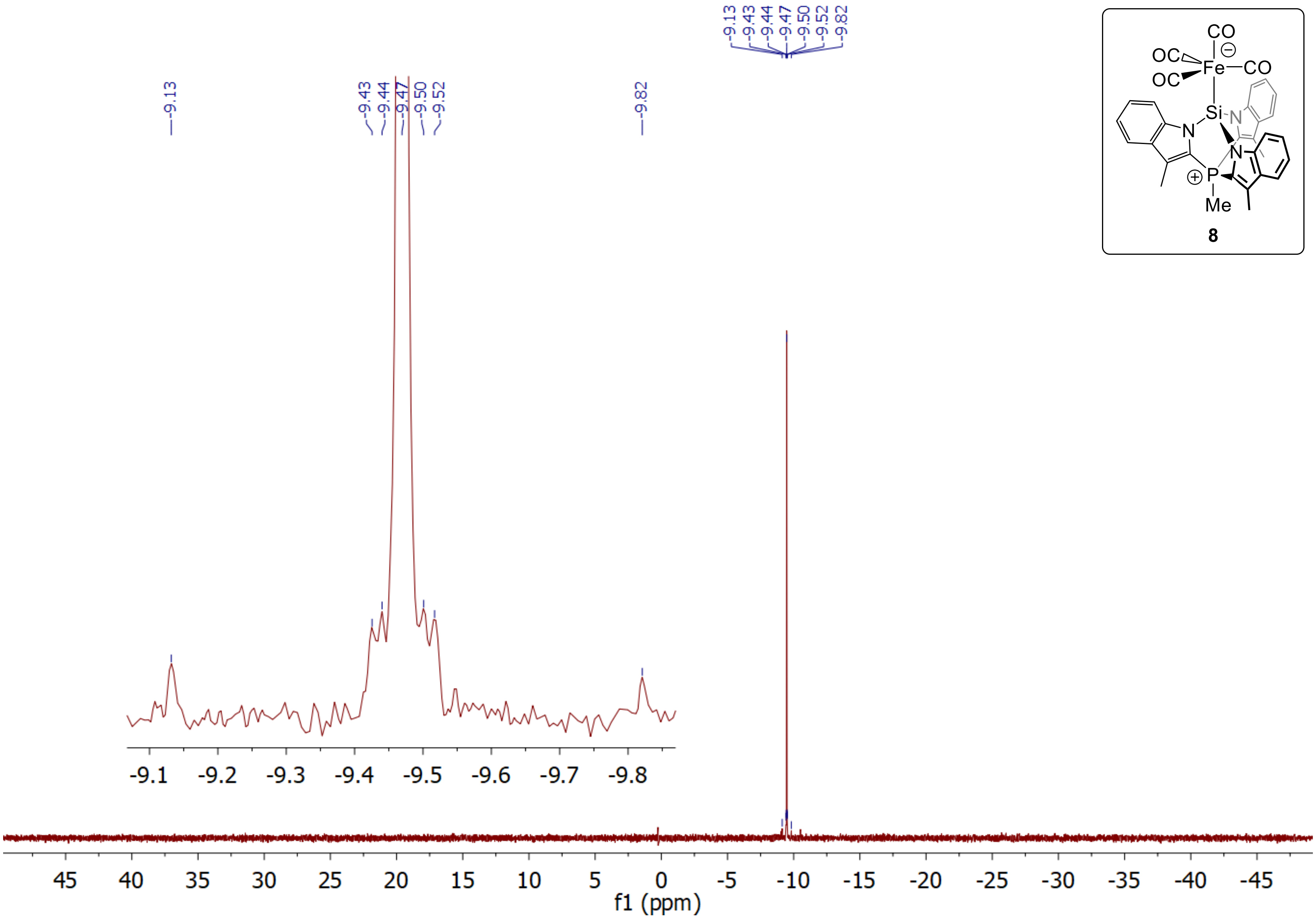

8

Figure S72. $^{31}P\{^{1}H\}$ NMR (162 MHz) spectrum of compound **8** in pyridine-d_5.

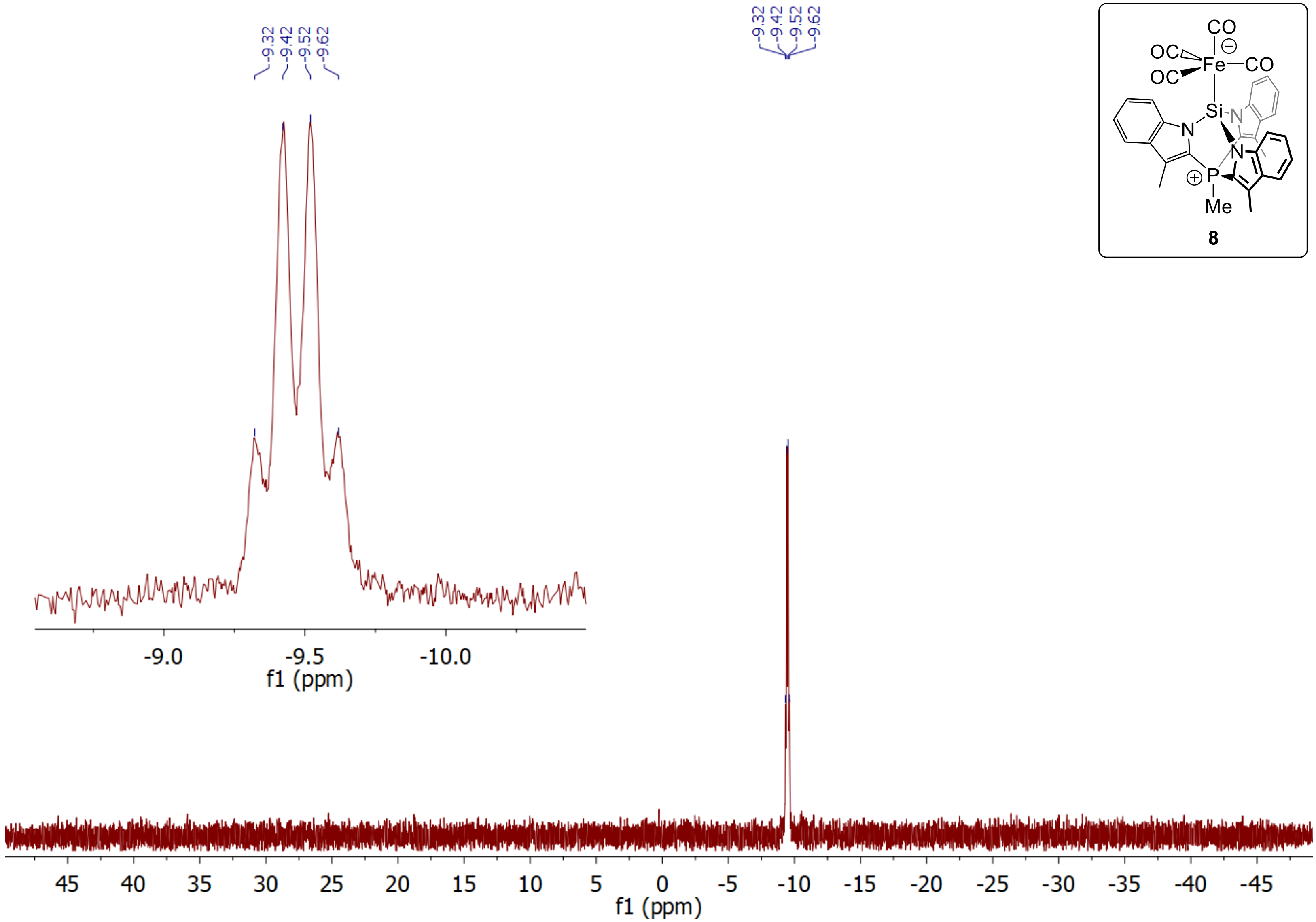

8

Figure S73. ^{31}P NMR (162 MHz) spectrum of compound **8** in pyridine-d_5.

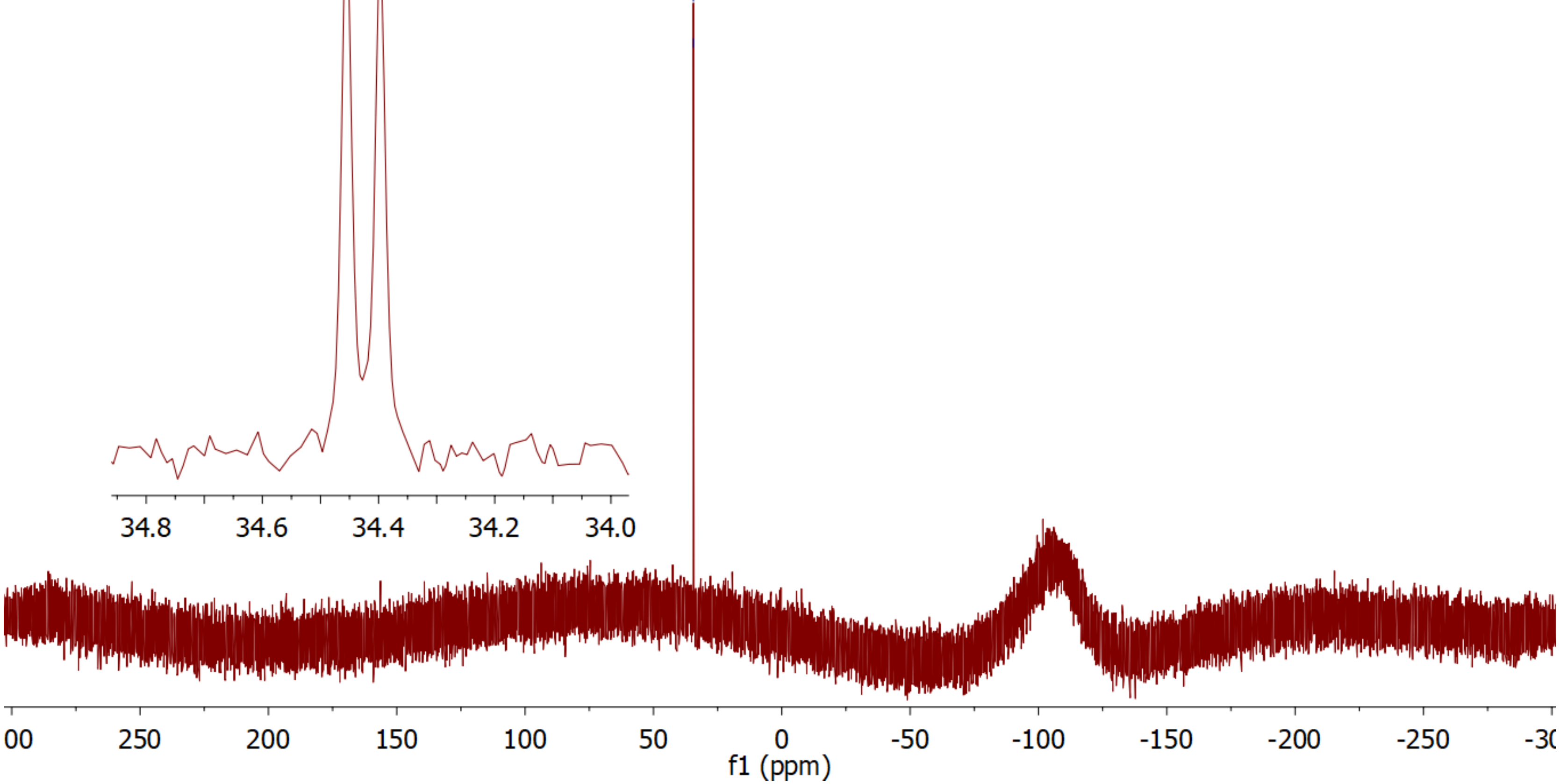

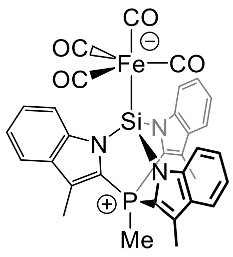

8

Figure S74. ^{29}Si NMR (79 MHz) spectrum of compound **8** in pyridine-d_5.

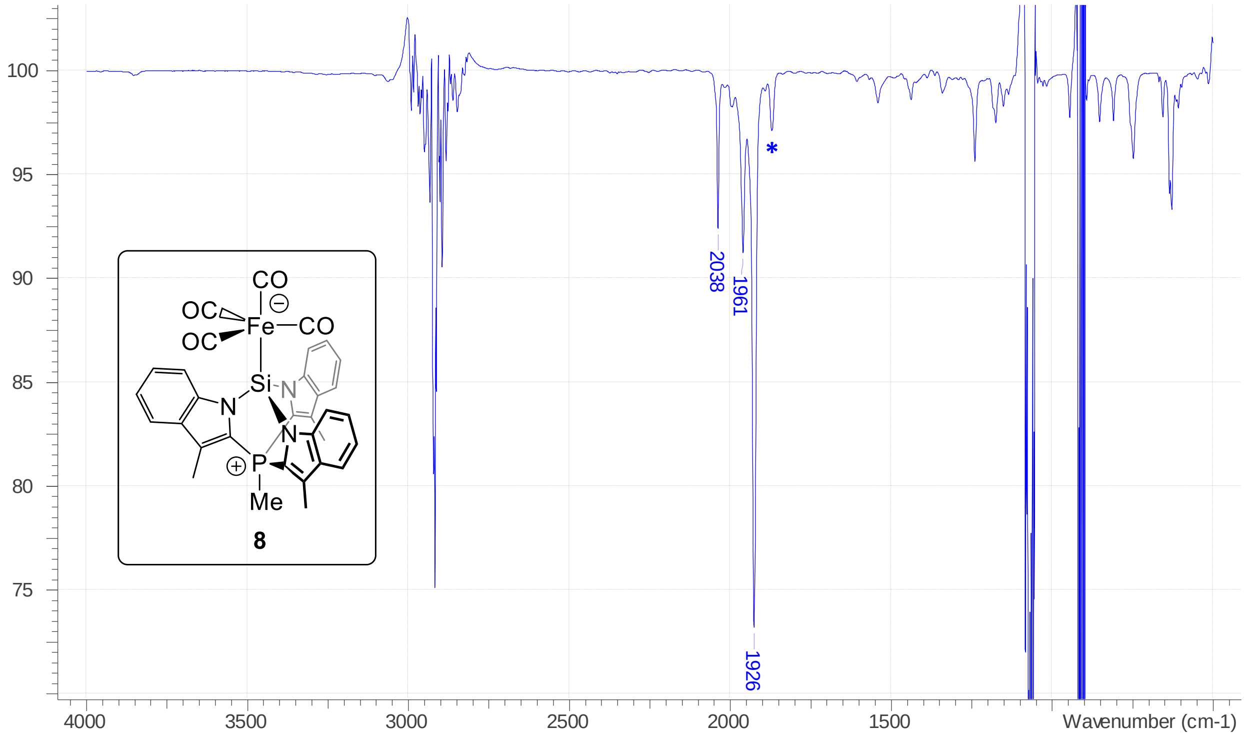

Figure S75. IR (THF solution) spectrum of compound **8**. Asterisk shows an unidentified trace impurity.

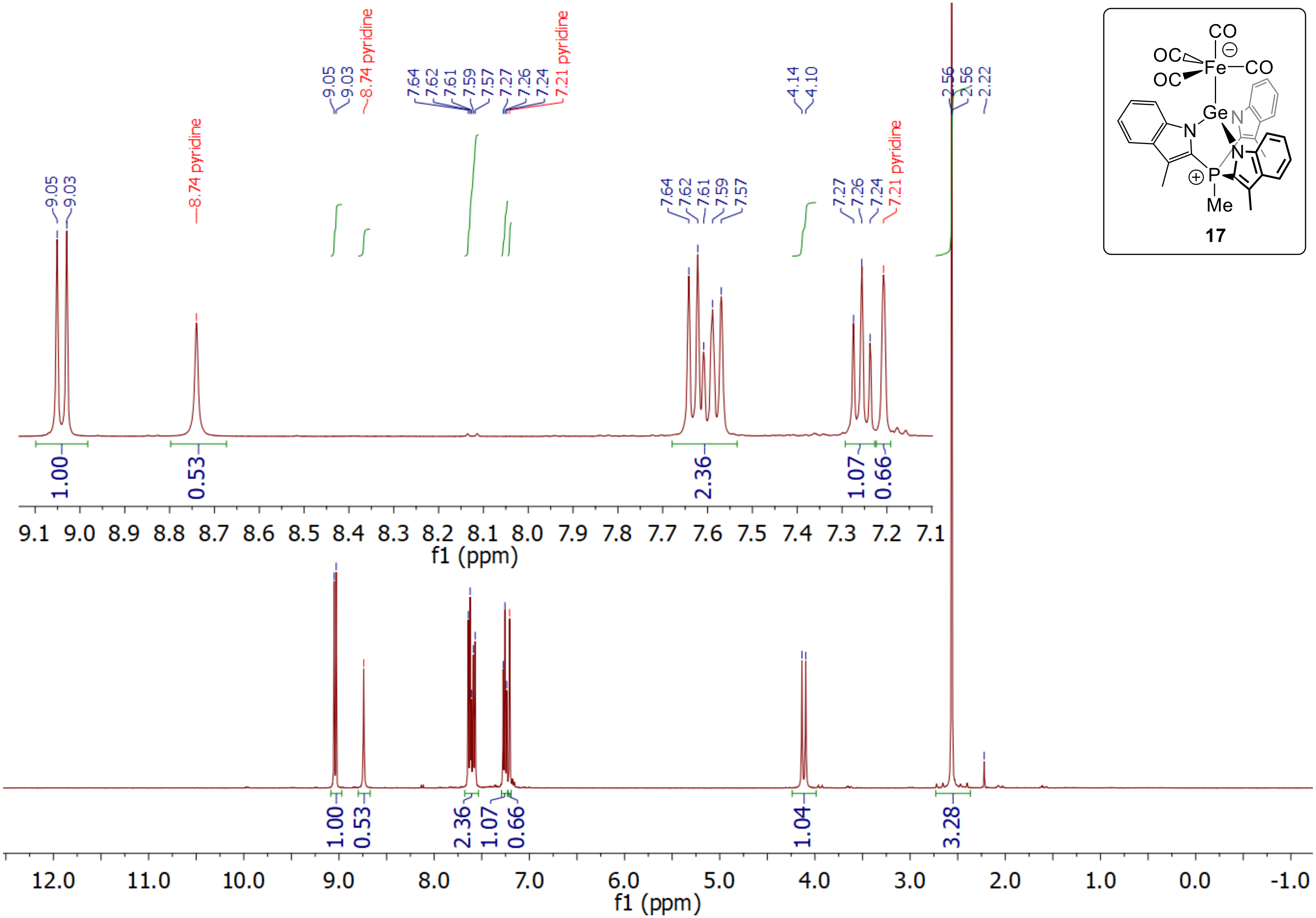

Figure S76. ¹H NMR (400 MHz) spectrum of compound **17** in pyridine-d_5. Impurities: 2.22 ppm – unidentified.

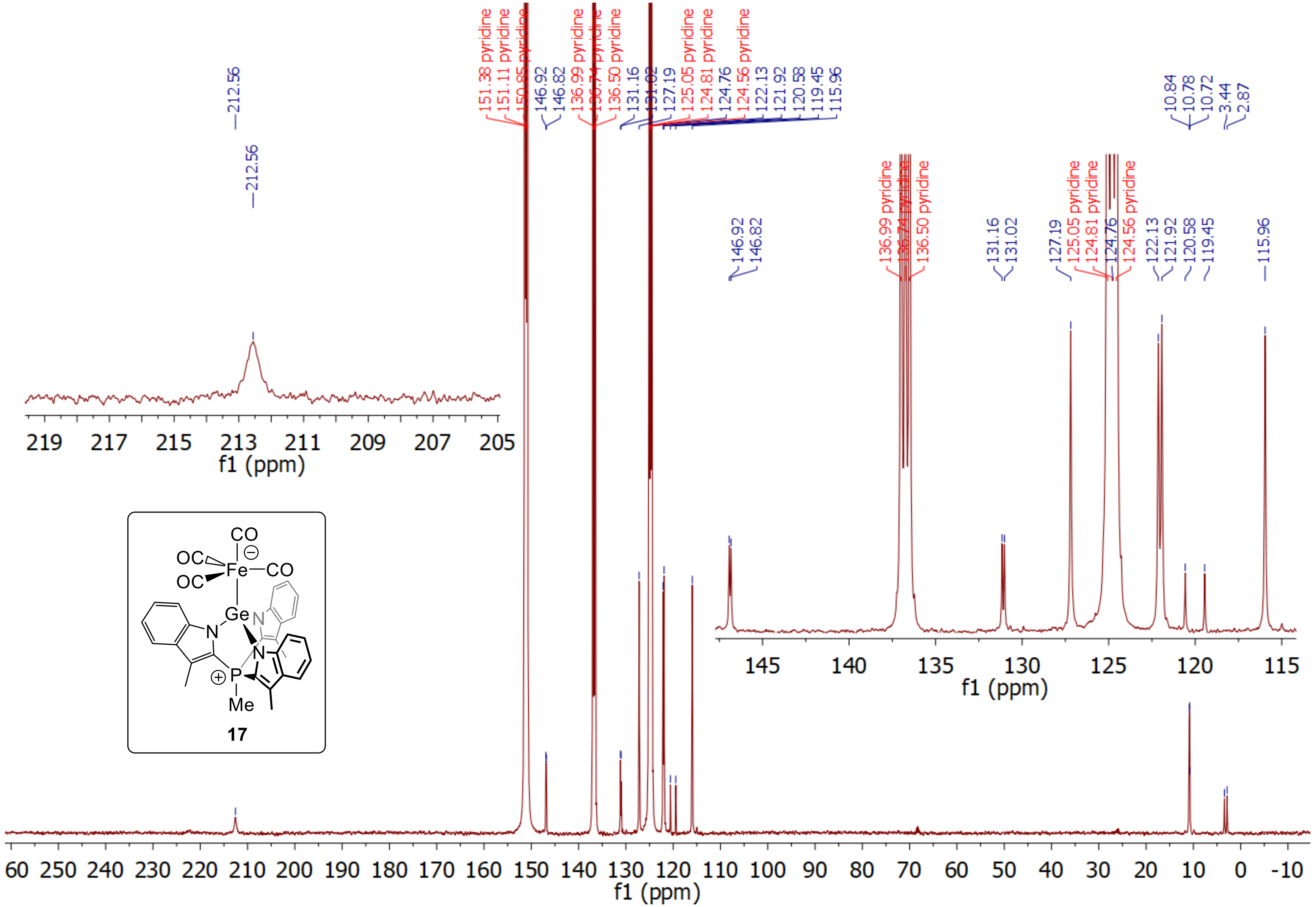

Figure S77. $^{13}C\{^1H\}$ NMR (101 MHz) spectrum of compound **17** in pyridine-d_5.

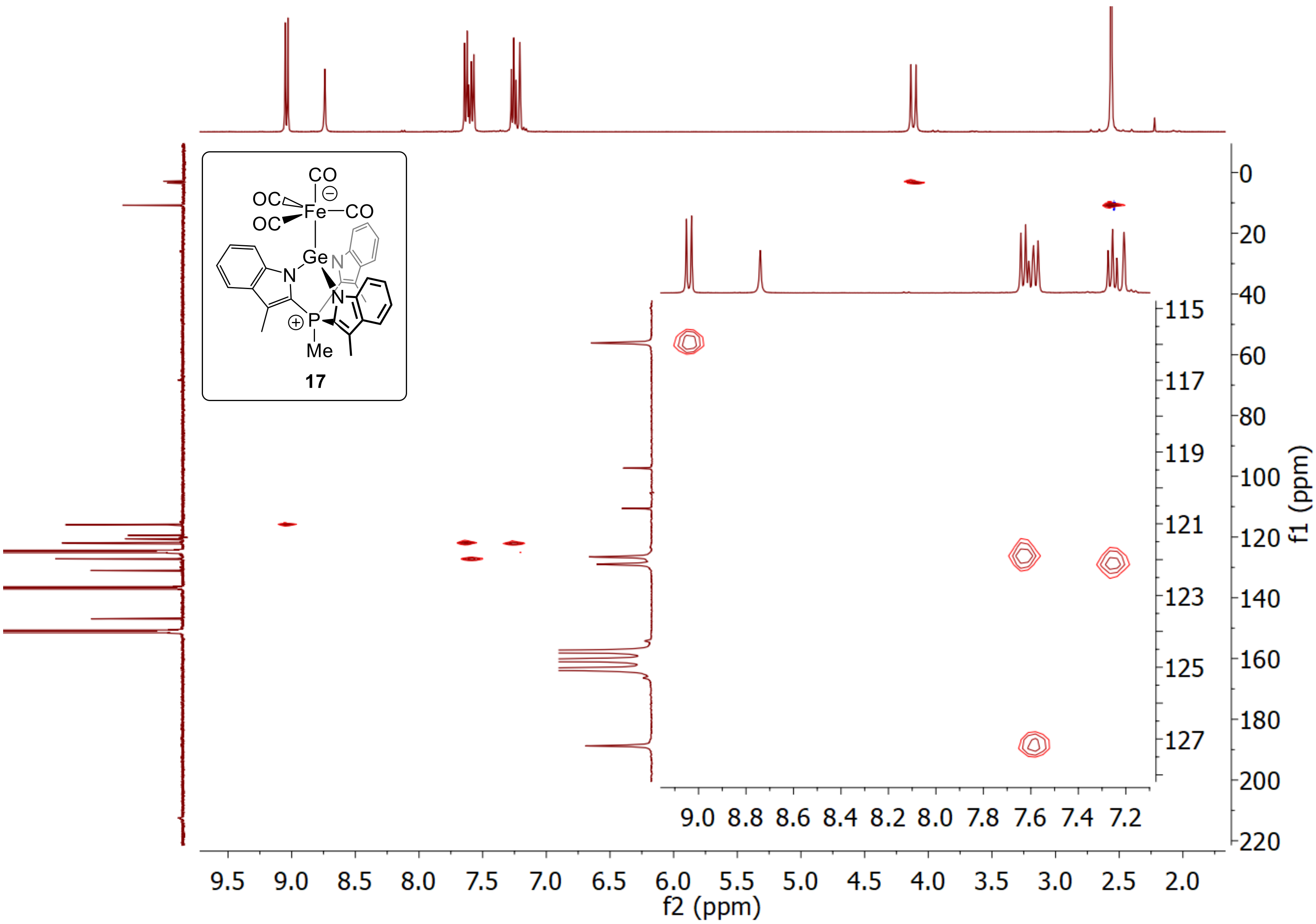

Figure S78. ^{1}H-^{13}C ASAPHMQC spectrum of compound **17** in pyridine-d_5.

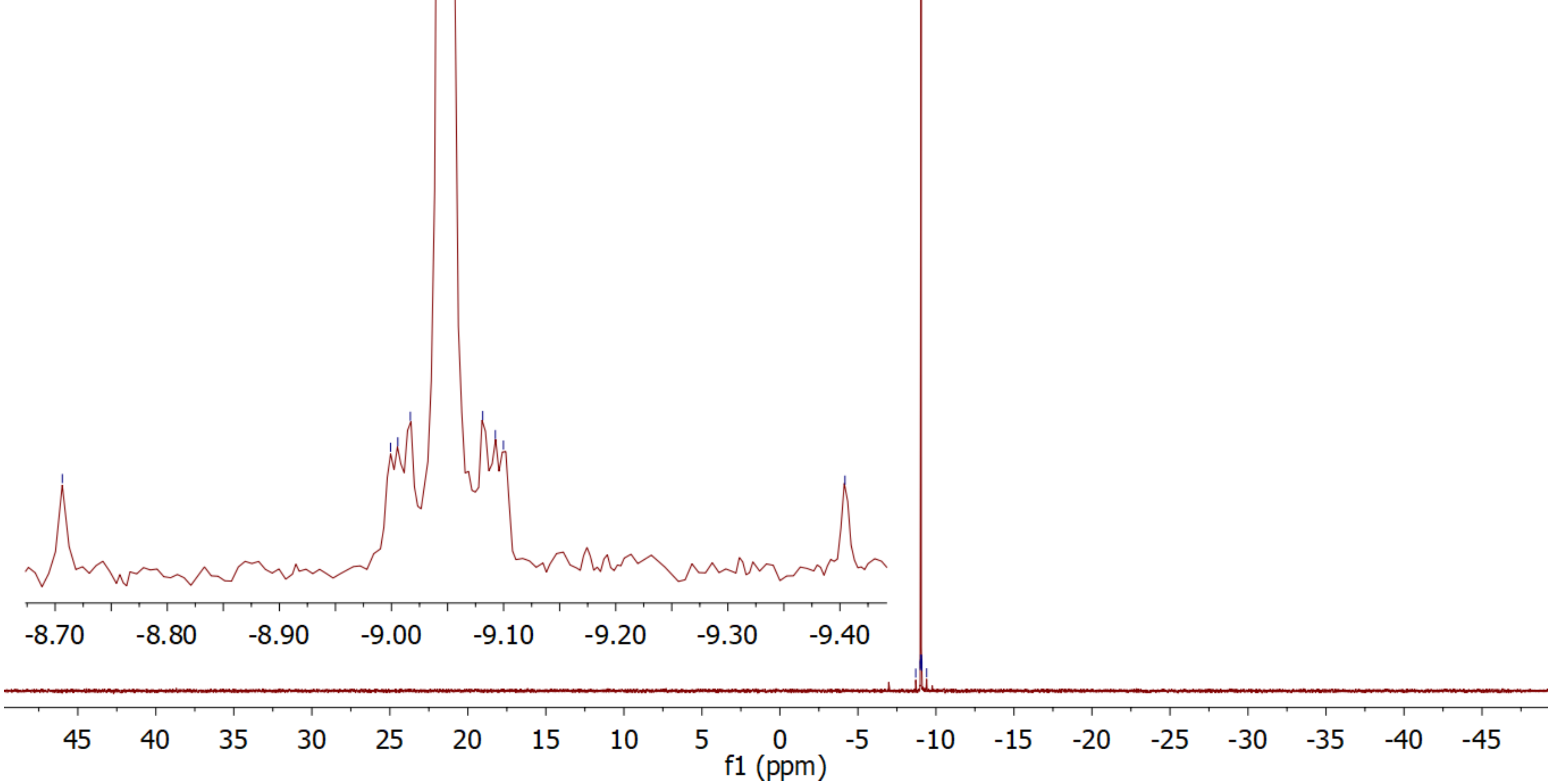

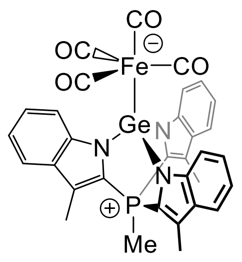

17

Figure S79. $^{31}P\{^{1}H\}$ NMR (162 MHz) spectrum of compound **17** in pyridine-d_5.

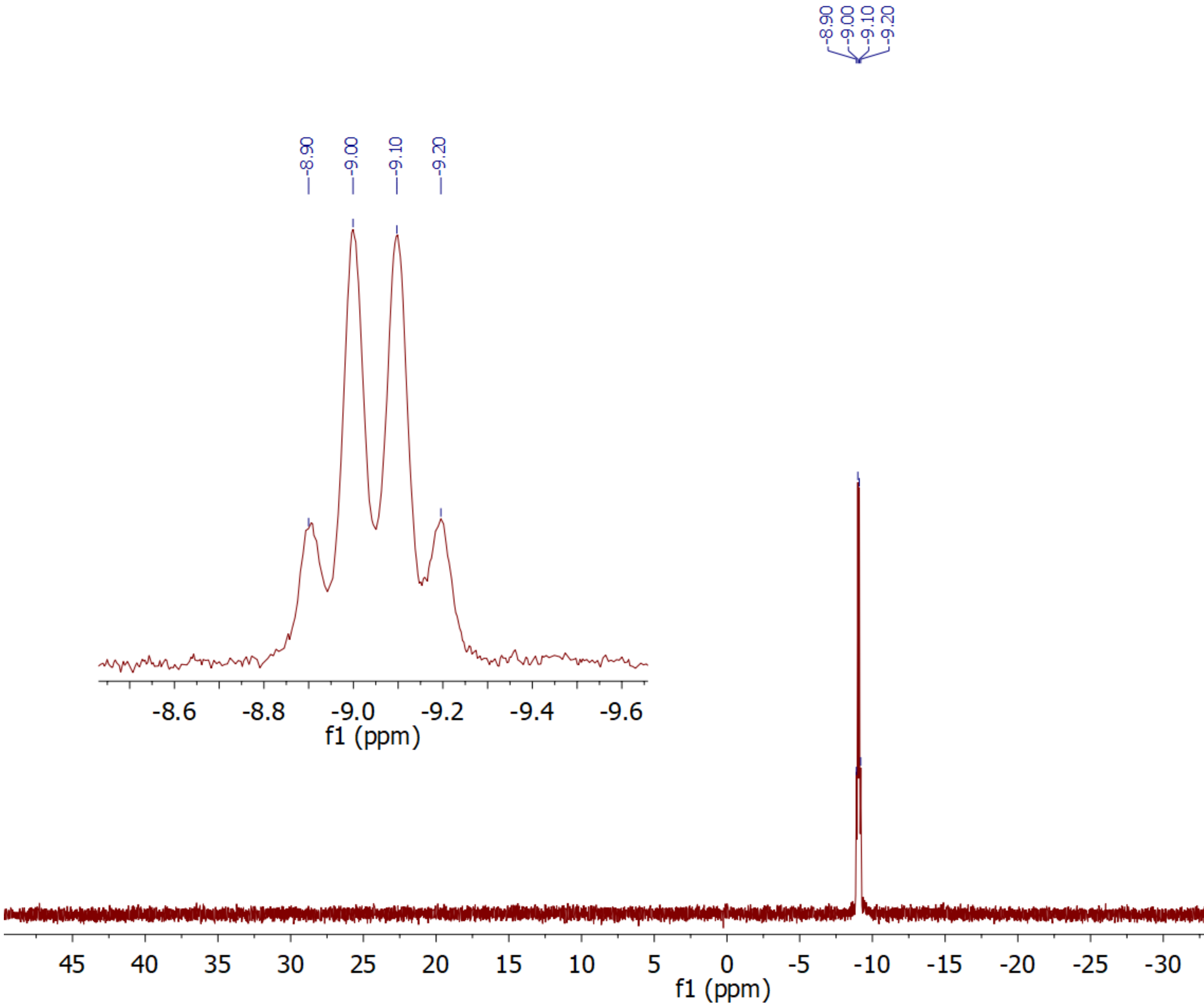

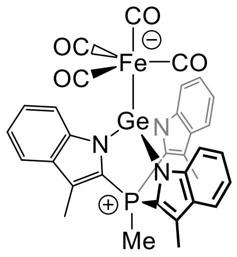

17

Figure S80. ^{31}P NMR (162 MHz) spectrum of compound **17** in pyridine-d_5.

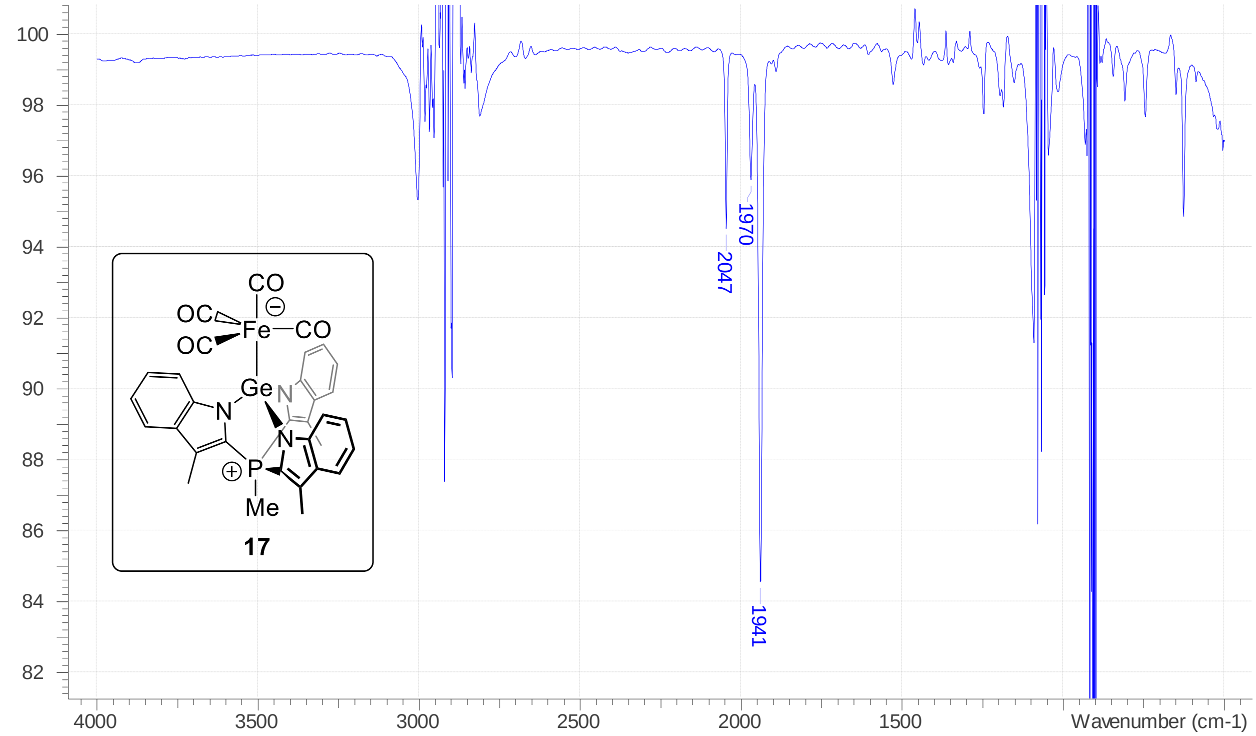

Figure S81. IR (THF solution) spectrum of compound **17**.

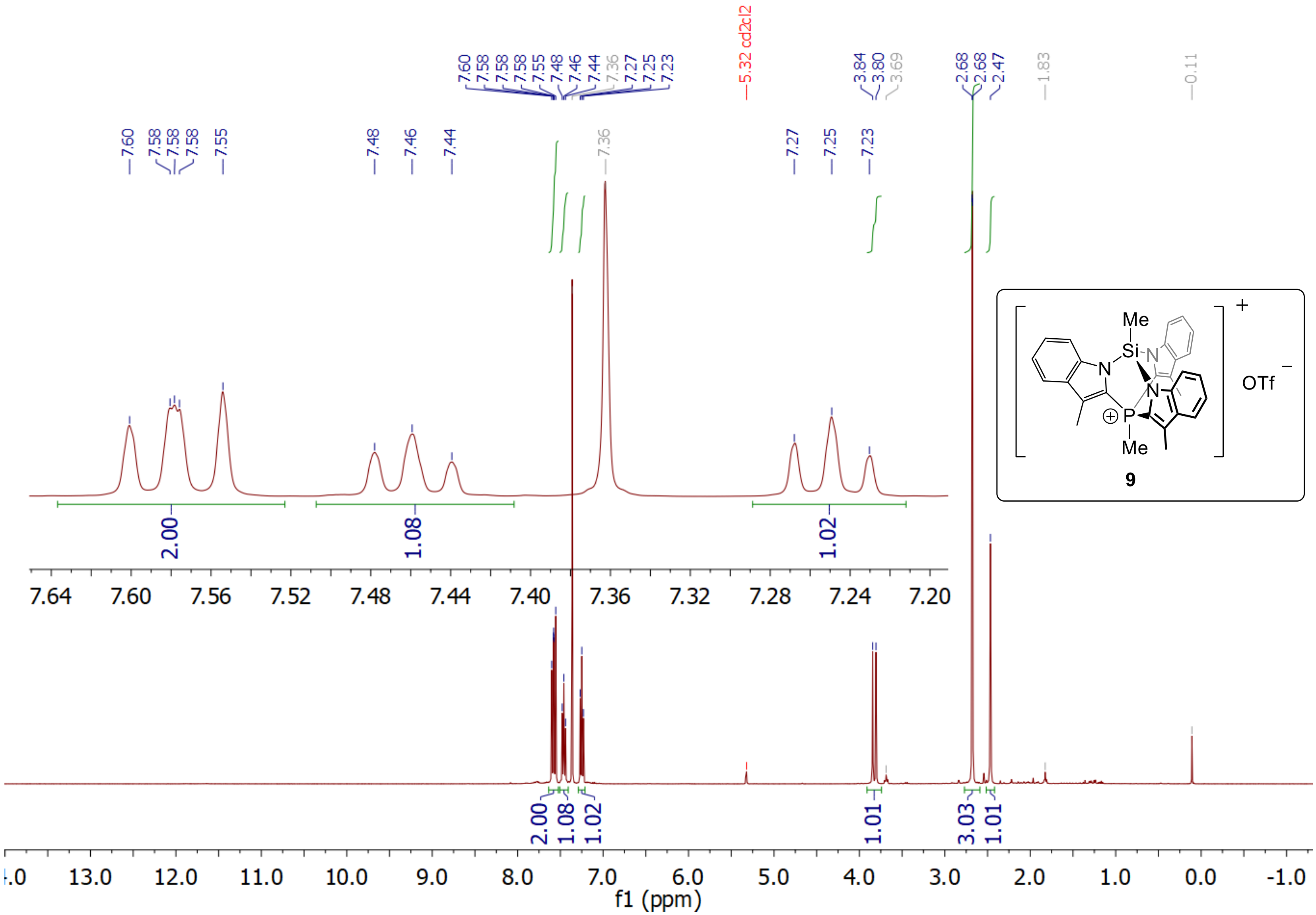

Figure S82. ^{1}H NMR (400 MHz) spectrum of compound **9** in DCM-d_2. Impurities: 0.11 ppm – grease, 1.83 and 3.69 ppm – THF, 7.36 ppm - benzene. Other minor peaks are unidentified.

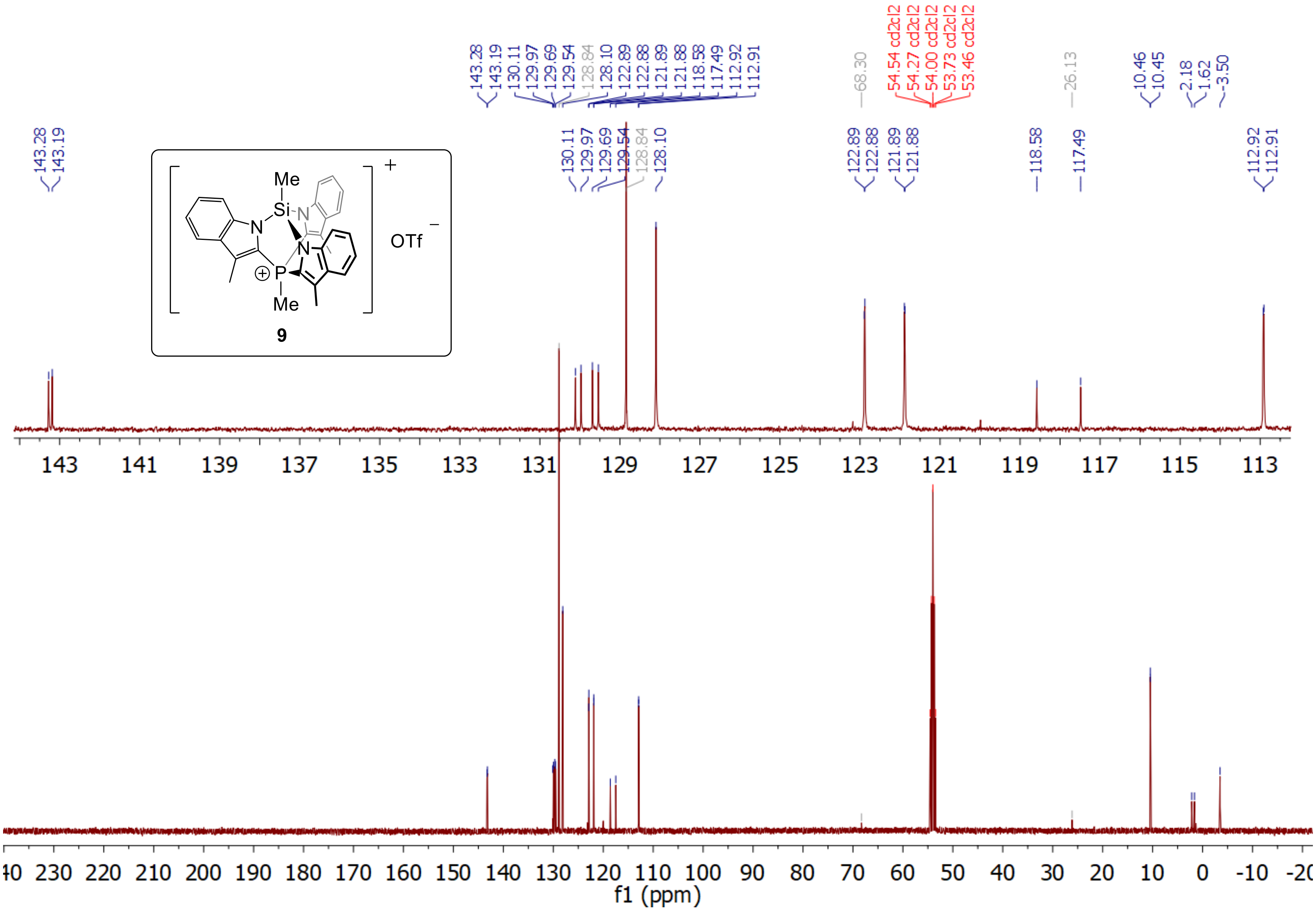

Figure S83. $^{13}C\{^{1}H\}$ NMR (101 MHz) spectrum of compound **9** in DCM-d_2. Peaks at 26.13 and 68.30 ppm belong to THF.

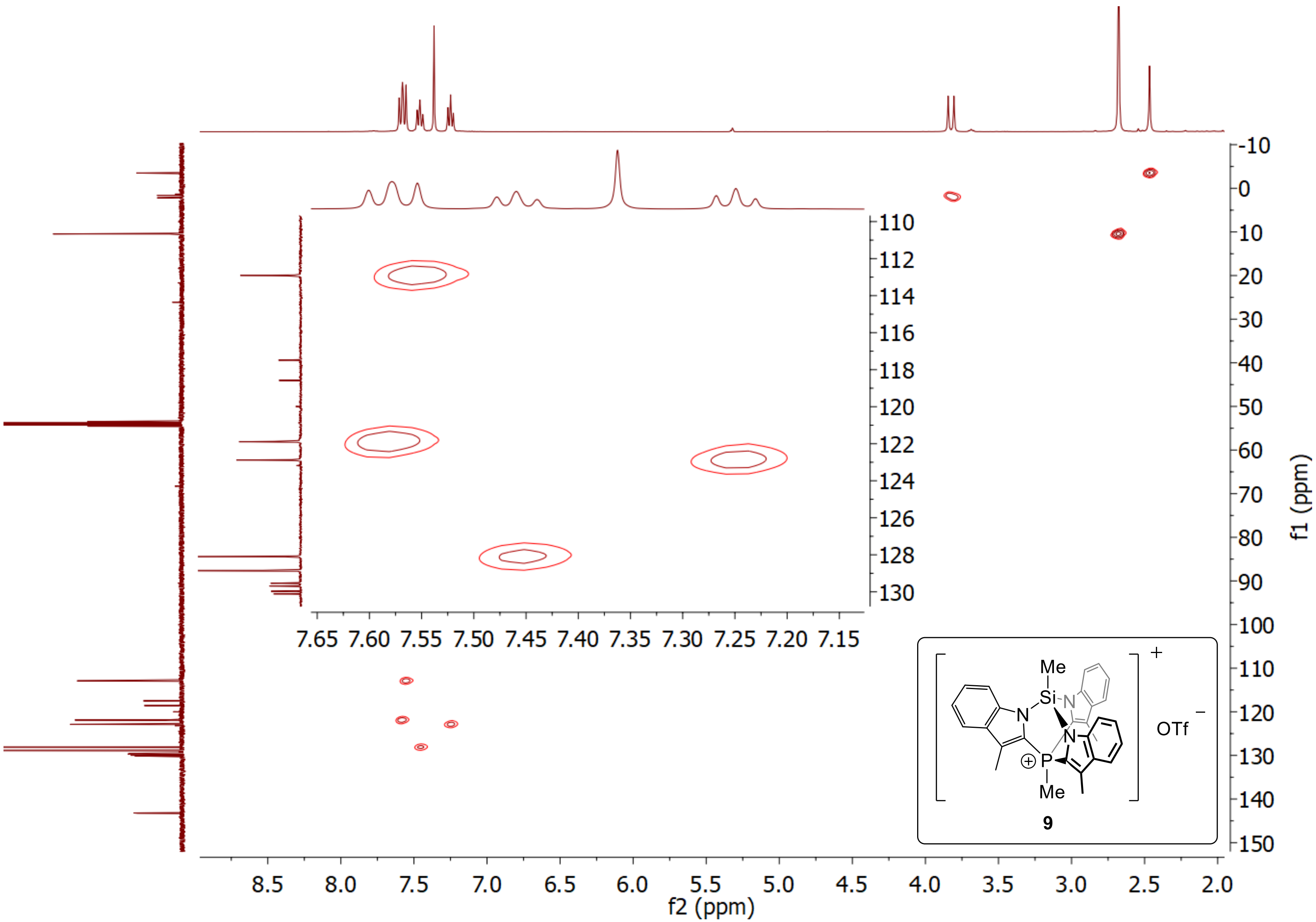

Figure S84. ^{1}H-^{13}C ASAPHMQC spectrum of compound **9** in DCM-d_2.

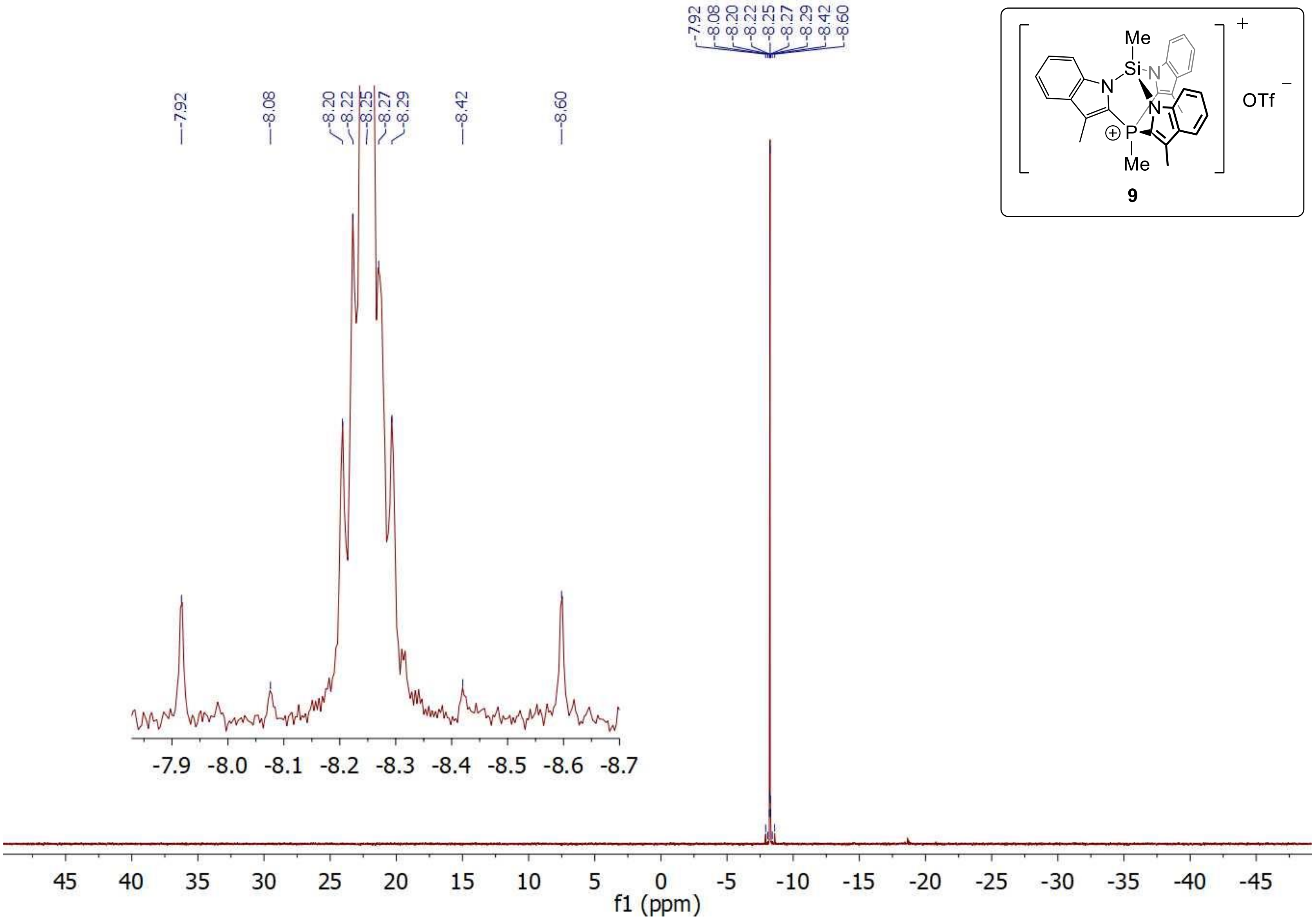

Figure S85. $^{31}P\{^{1}H\}$ NMR (162 MHz) spectrum of compound **9** in DCM-d_2.

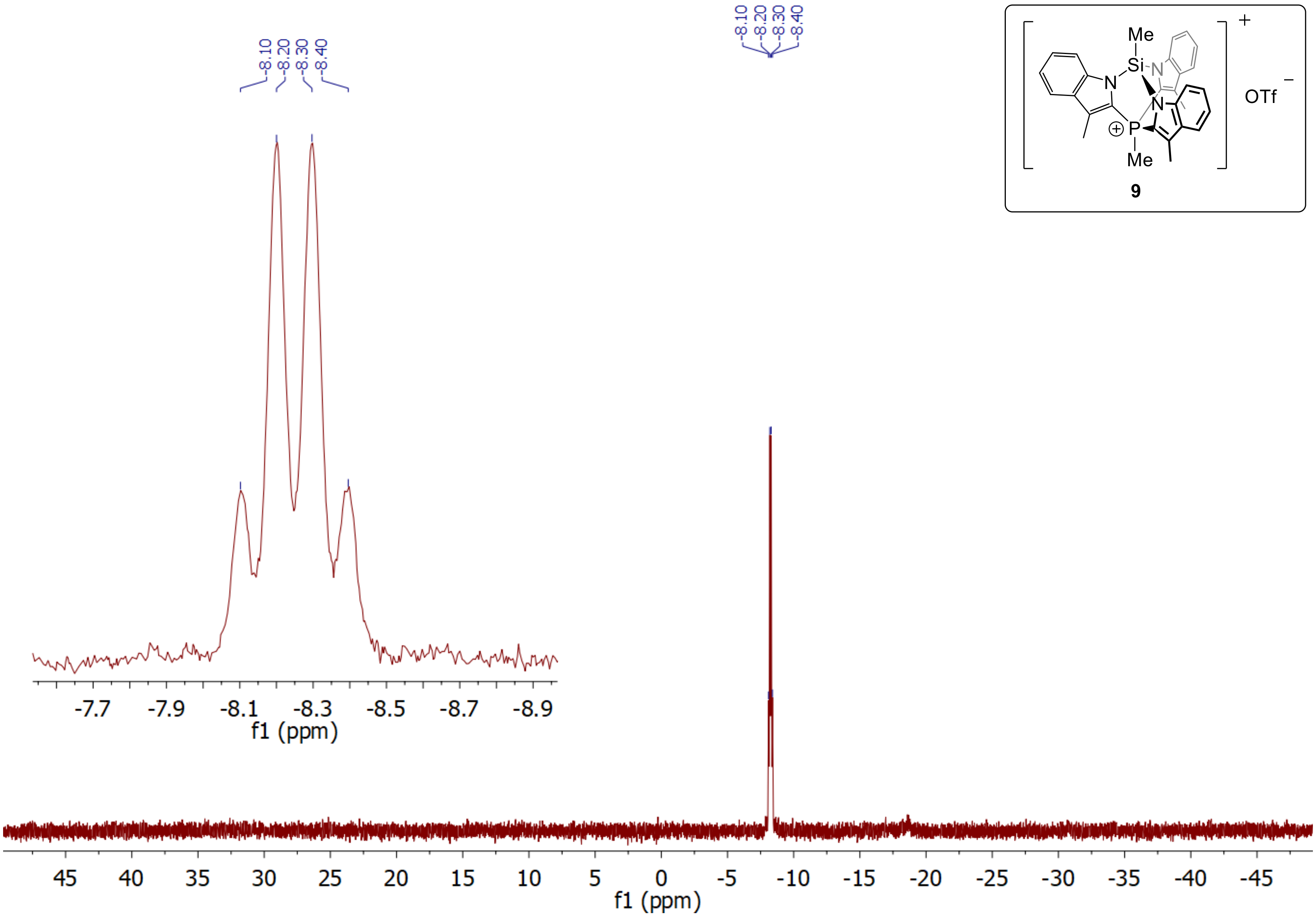

Figure S86. ^{31}P NMR (162 MHz) spectrum of compound **9** in DCM-d_2.

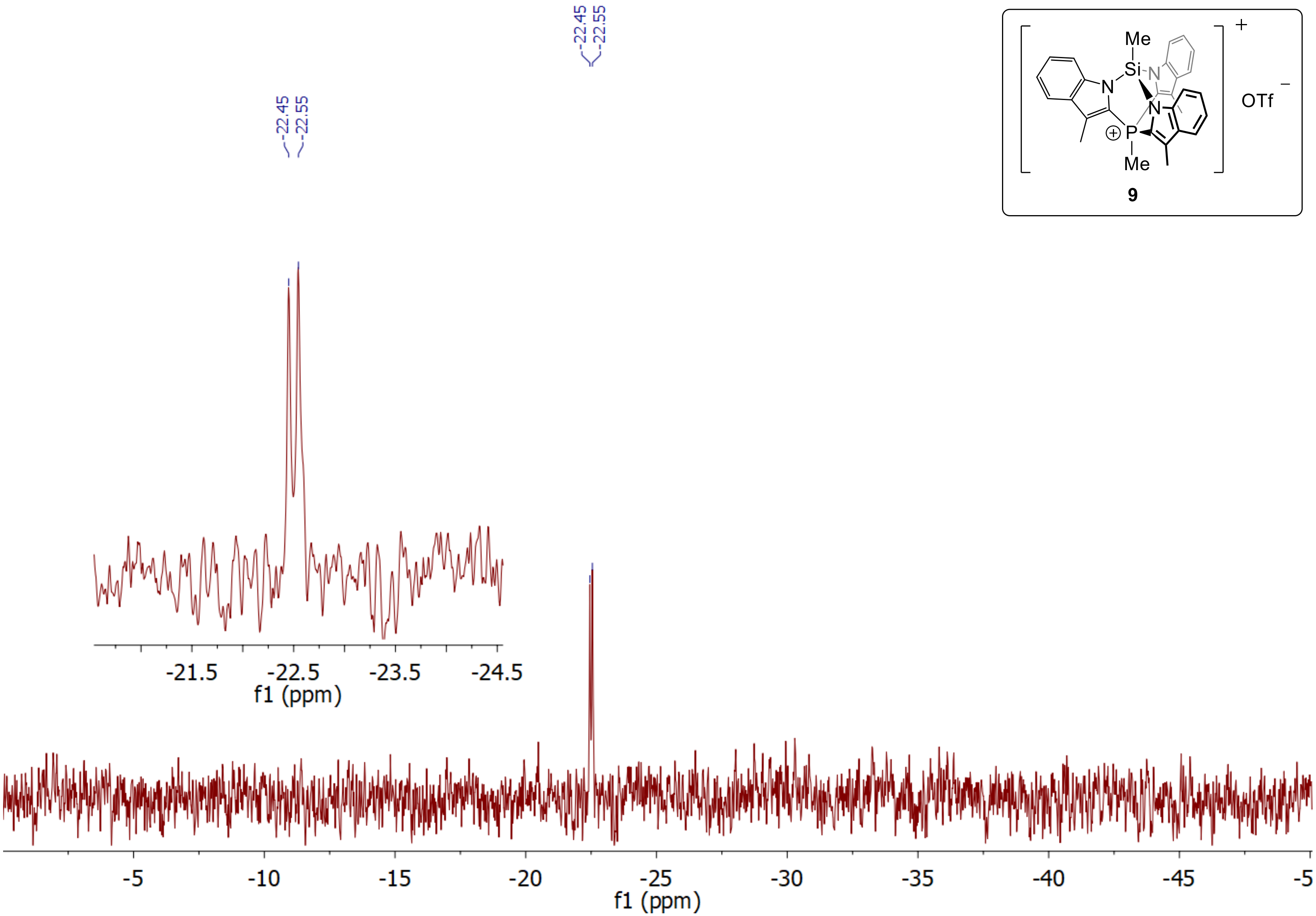

9

Figure S87. $^{29}Si\{^1H\}$ NMR (79 MHz) spectrum of compound **9** in DCM-d_2.

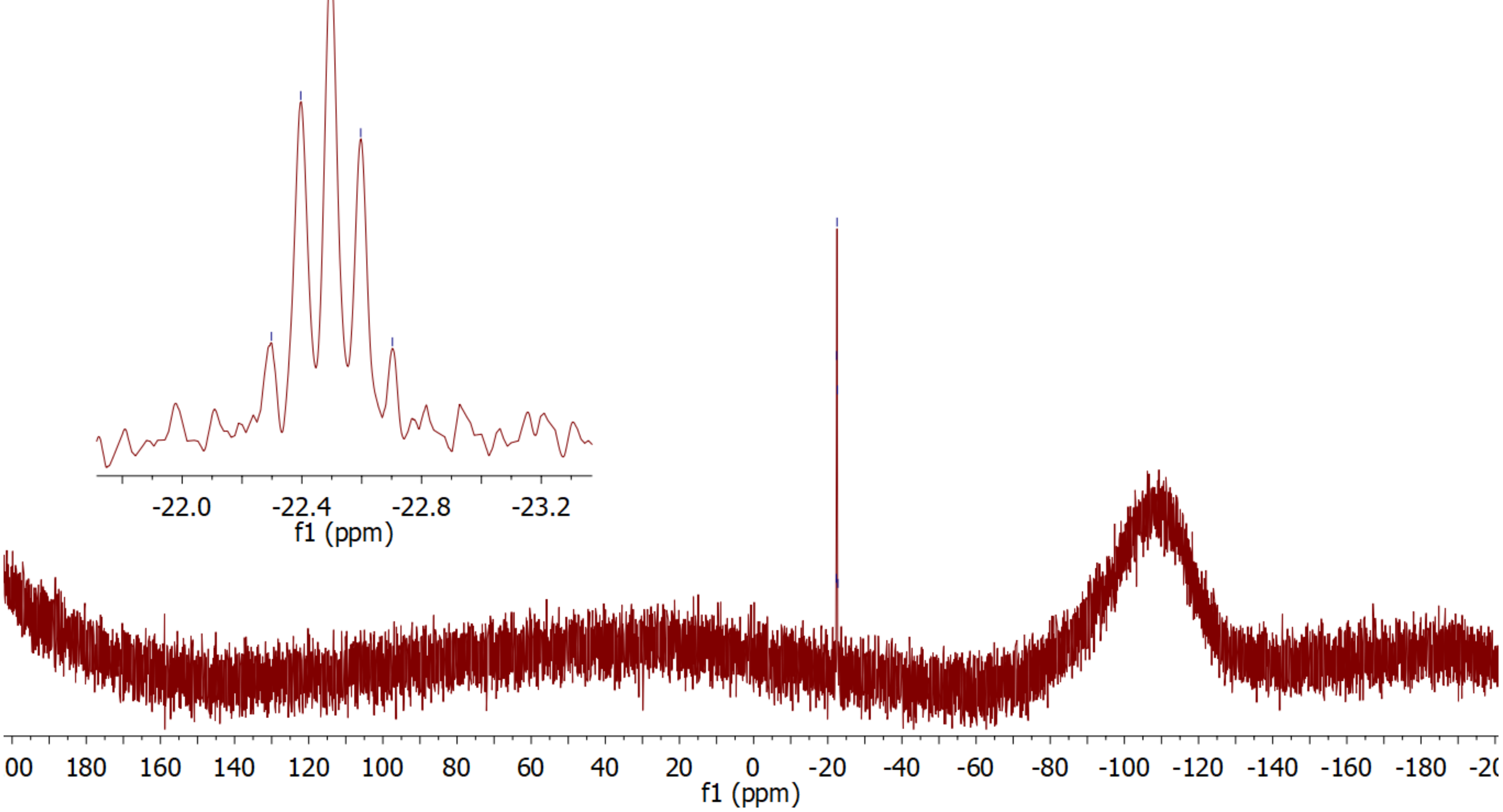

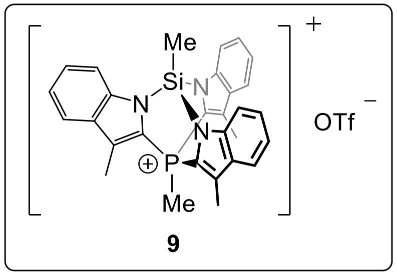

Figure S88. ^{29}Si NMR (79 MHz) spectrum of compound **9** in DCM-d$_2$.

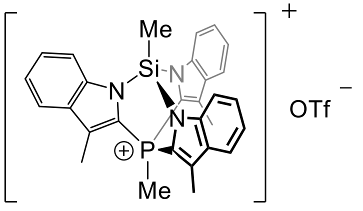

9

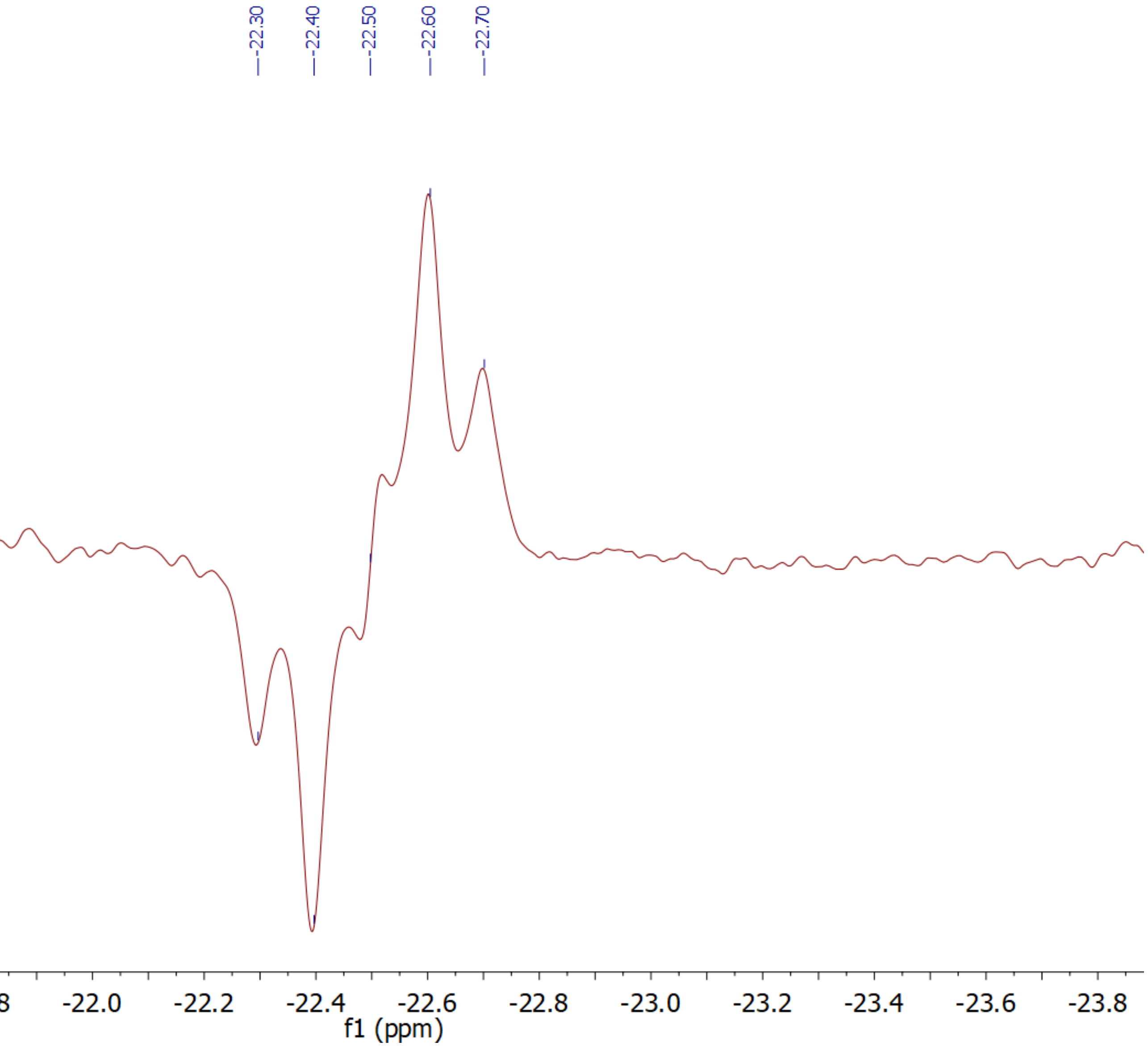

Figure S89. Non-refocused ^{29}Si INEPT (79 MHz) spectrum of compound **9** in DCM-d_2.

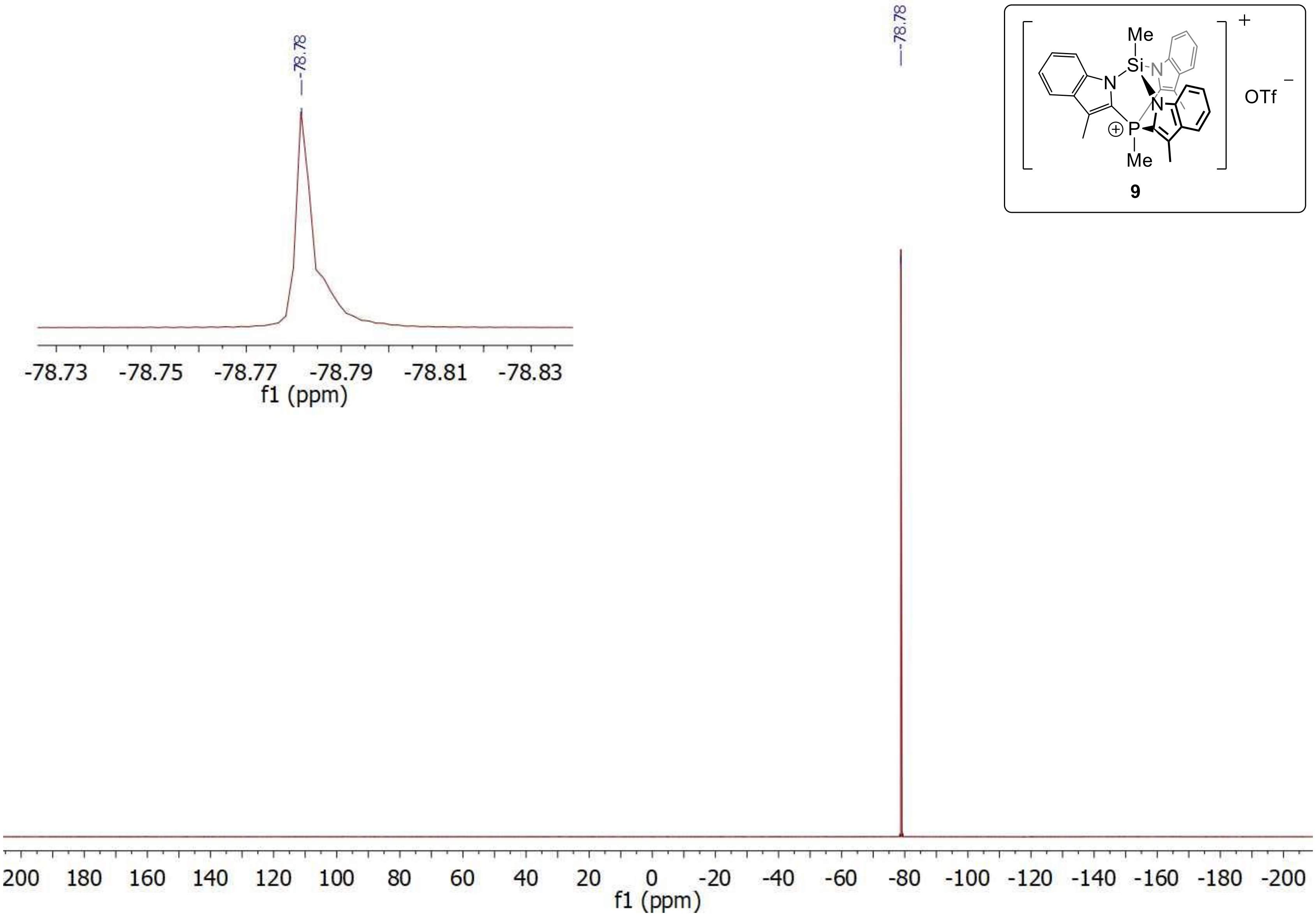

9

Figure S90. ^{19}F NMR (376 MHz) spectrum of compound **9** in DCM-d_2.

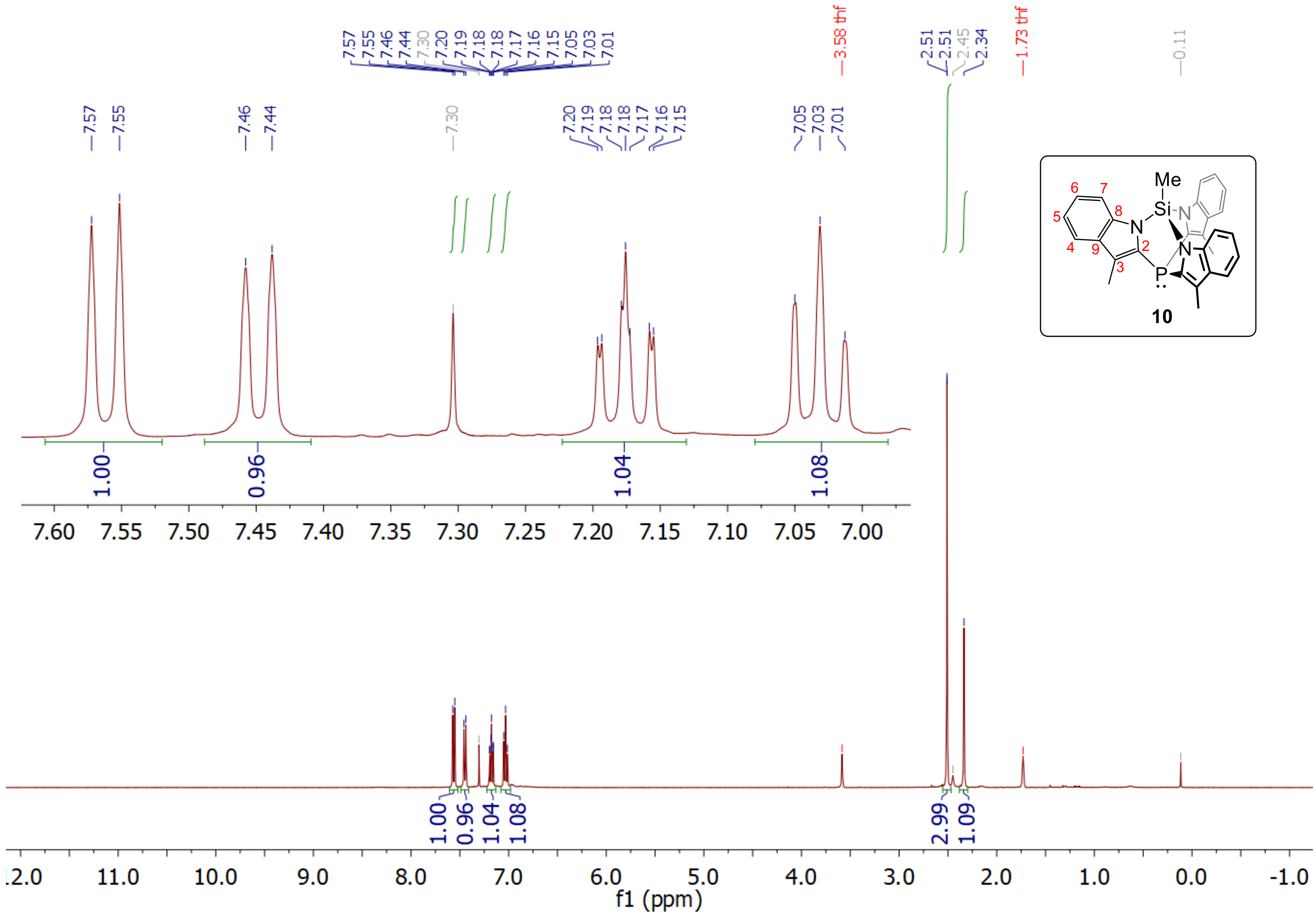

Figure S91. ^{1}H NMR (400 MHz) spectrum of compound **10** in THF-d_8. Impurities: 0.11 ppm – grease, 2.45 ppm – unidentified, 7.30 ppm – benzene.

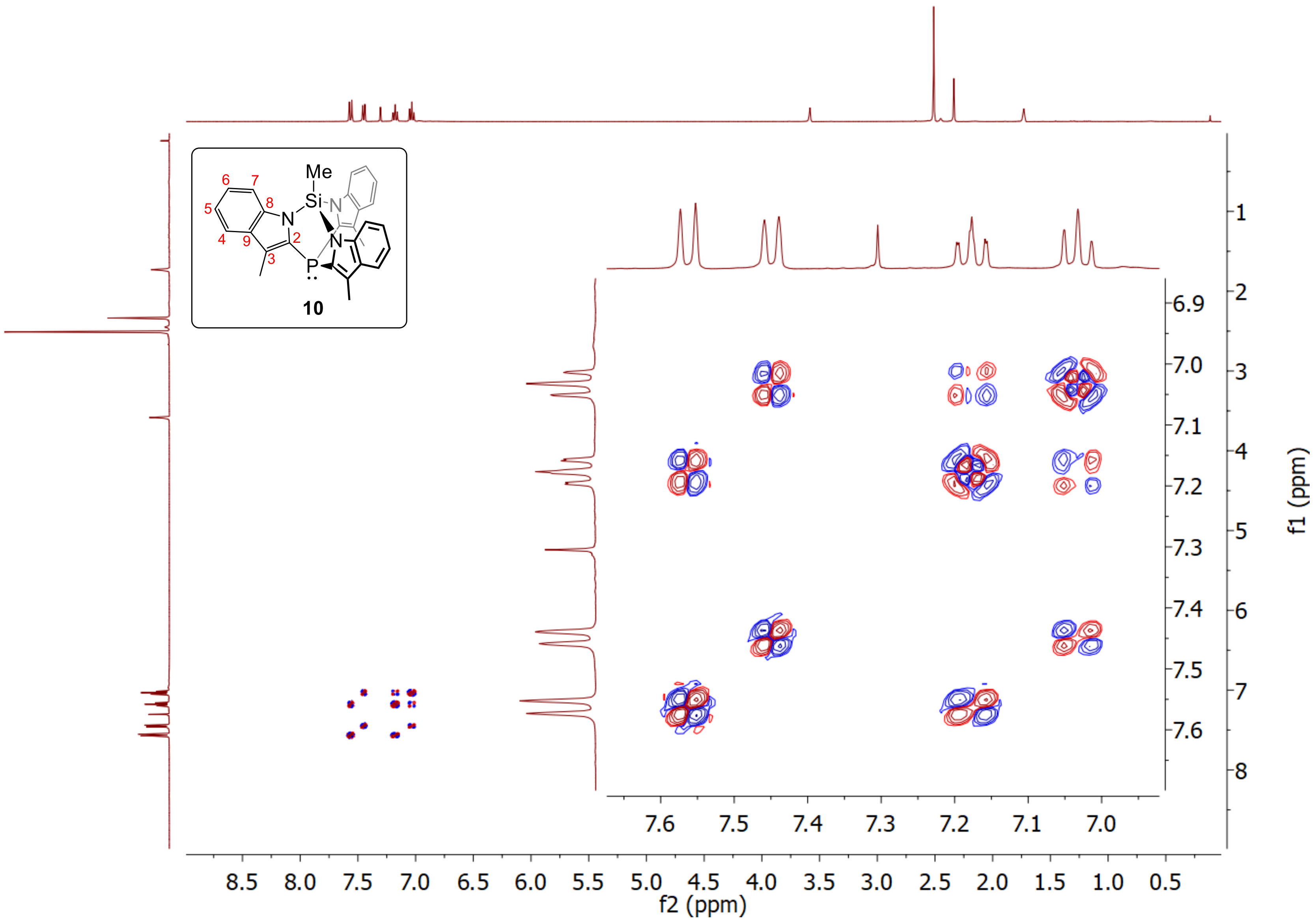

Figure S92. ^{1}H DQF-COSY (400 MHz) spectrum of compound **10** in THF-d_8.

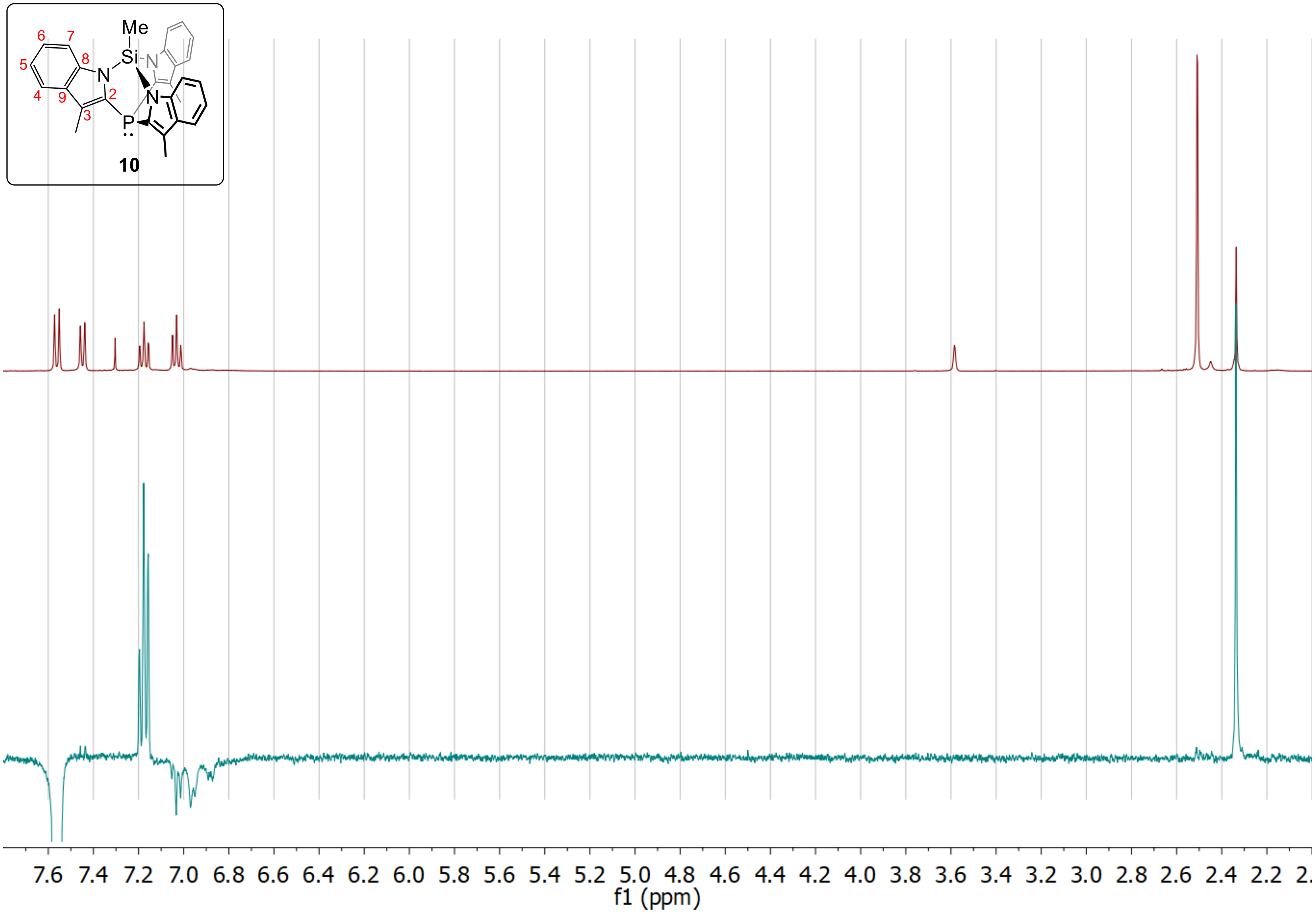

Figure S93. [1]H NOE (400 MHz) spectrum of compound **10** in THF-d_8 (bottom panel, top panel: regular ^{1}H spectrum for the reference). The signal at 7.56 ppm is being saturated.

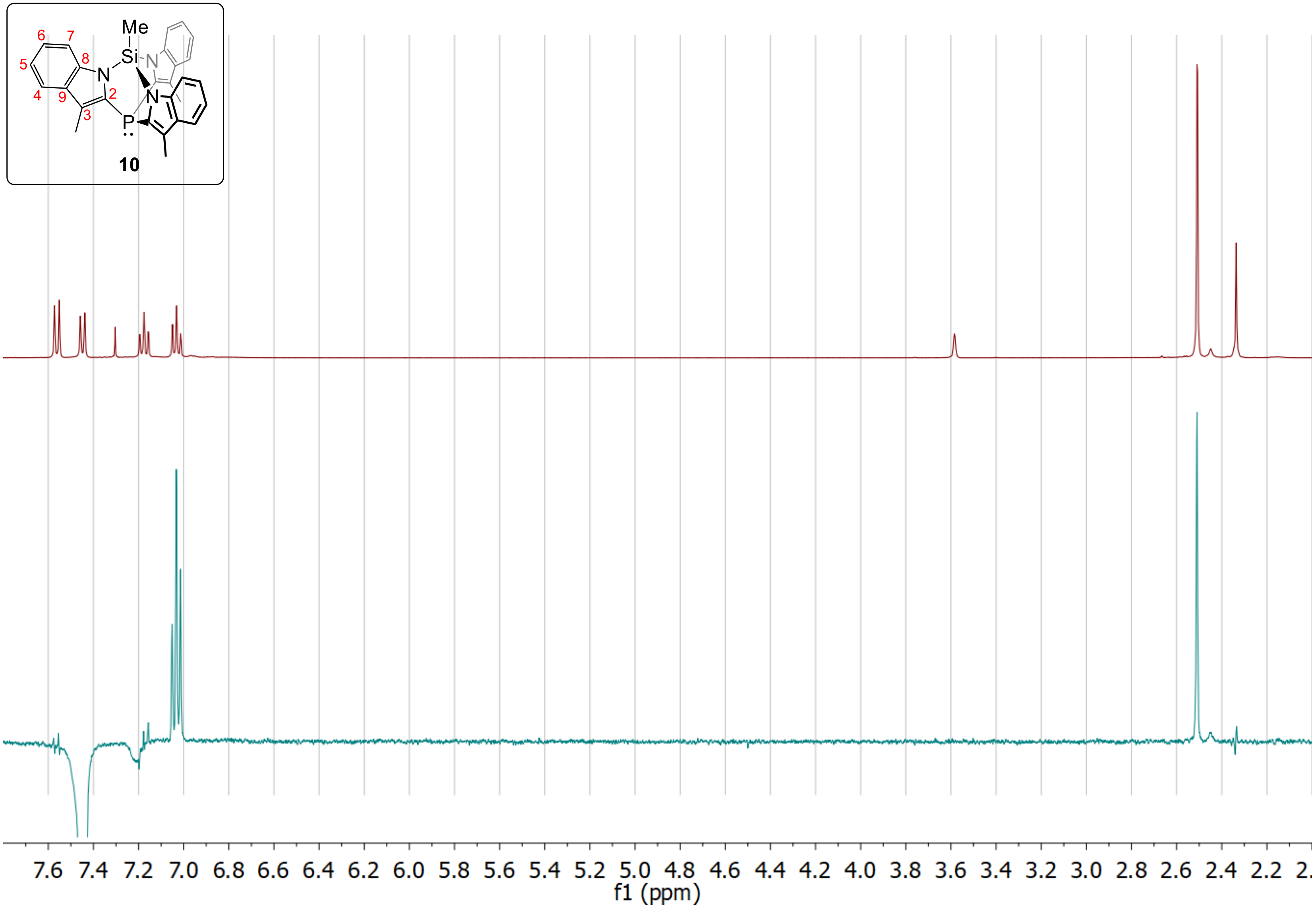

Figure S94. ^{1}H NOE (400 MHz) spectrum of compound **10** in THF-d_8 (bottom panel, top panel: regular ^{1}H spectrum for the reference). The signal at 7.45 ppm is being saturated.

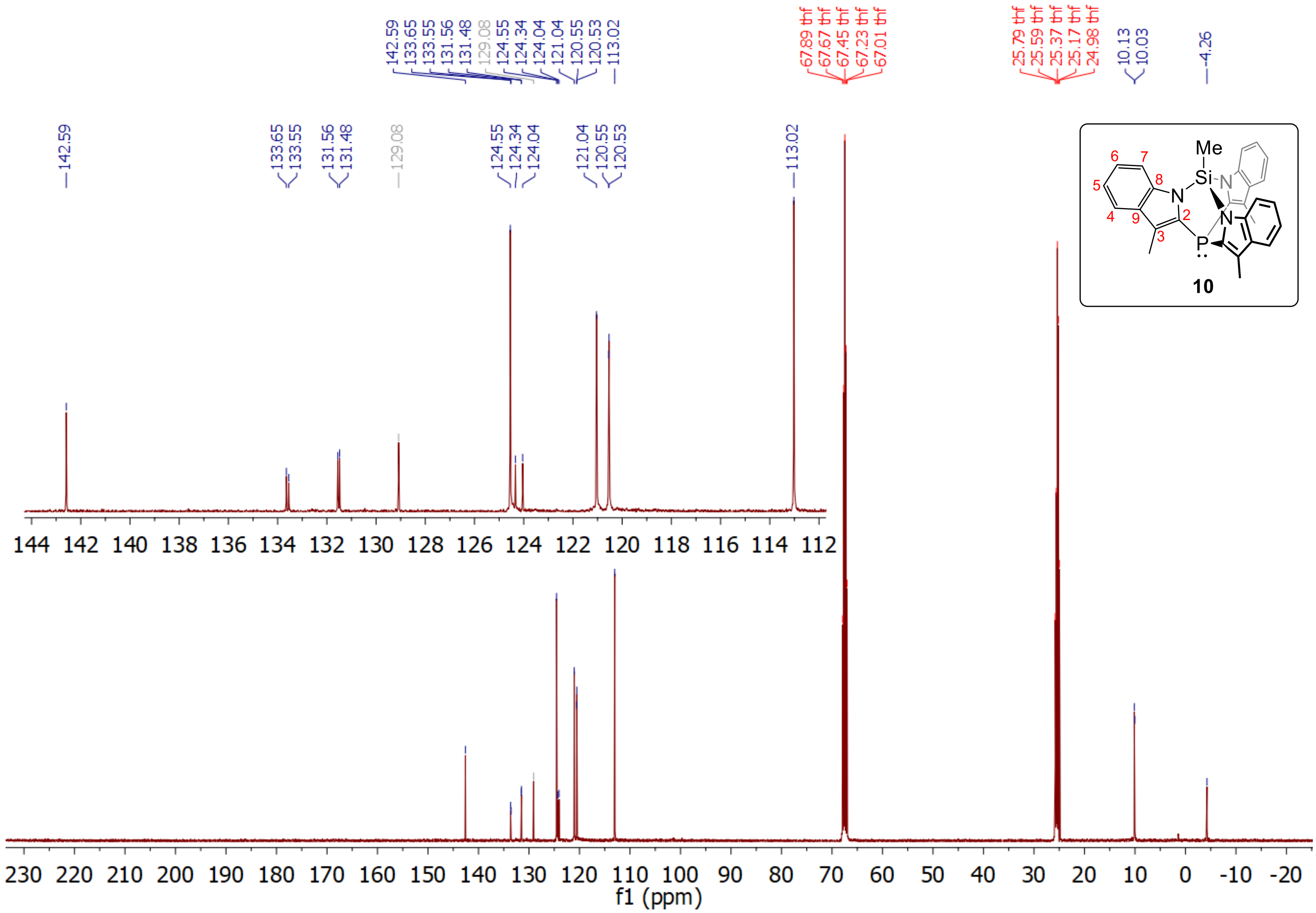

Figure S95. $^{13}C\{^{1}H\}$ NMR (101 MHz) spectrum of compound **10** in THF-d_8. Impurities: 129.08 ppm – benzene.

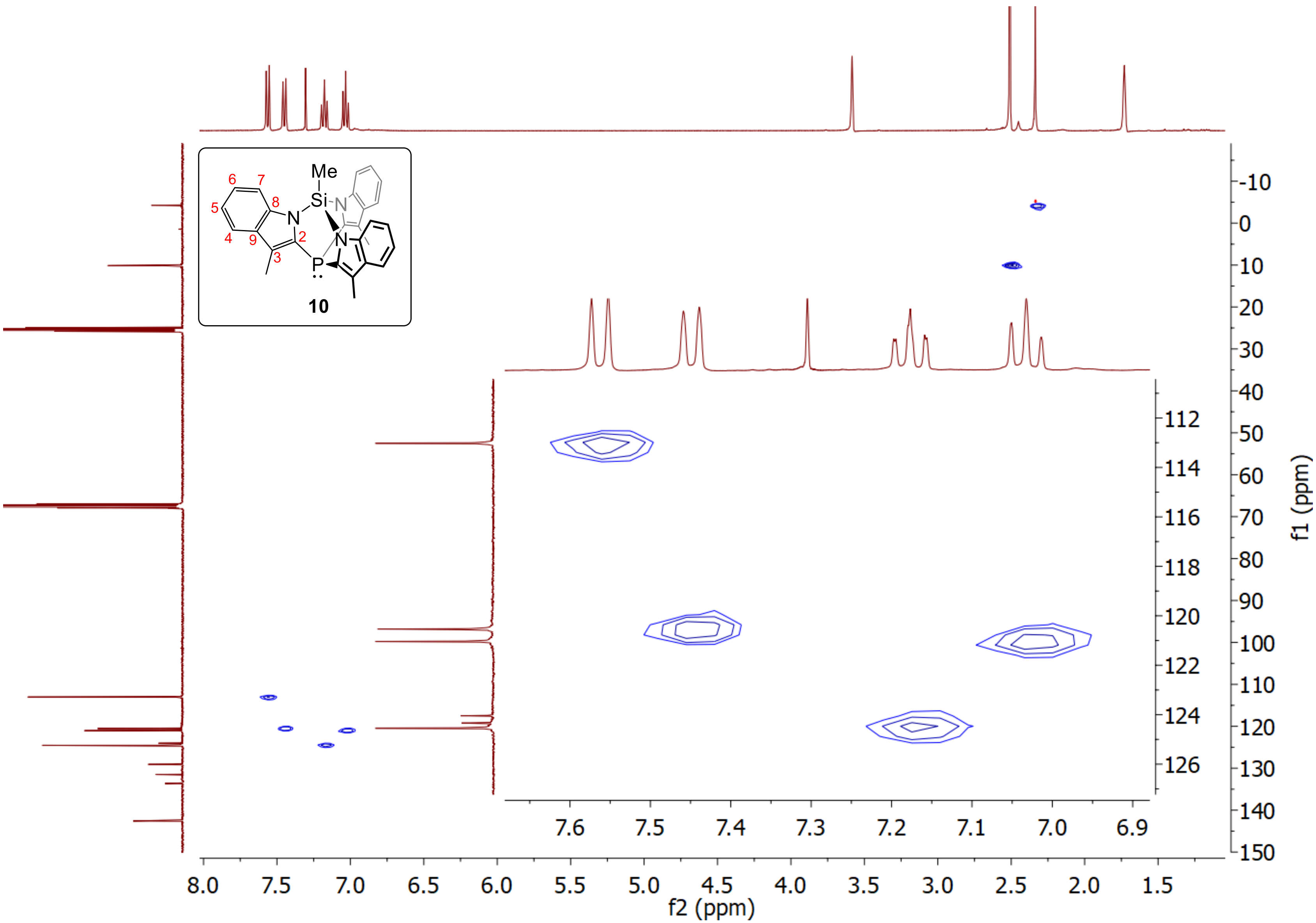

Figure S96. ^{1}H-^{13}C ASAPHMQC spectrum of compound **10** in THF-d_8.

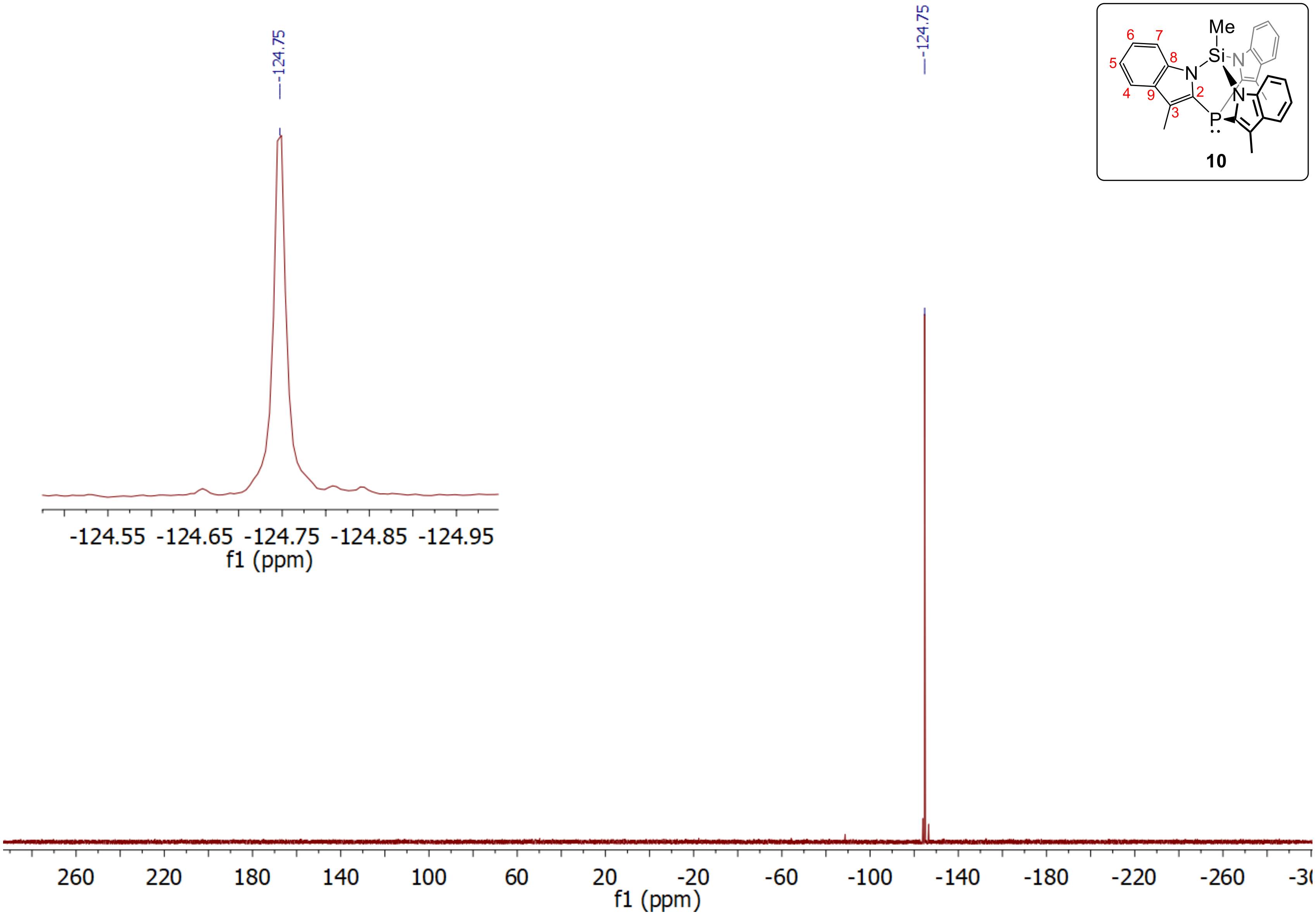

10

Figure S97. ^{31}P NMR (162 MHz) spectrum of compound **10** in THF-d_8.

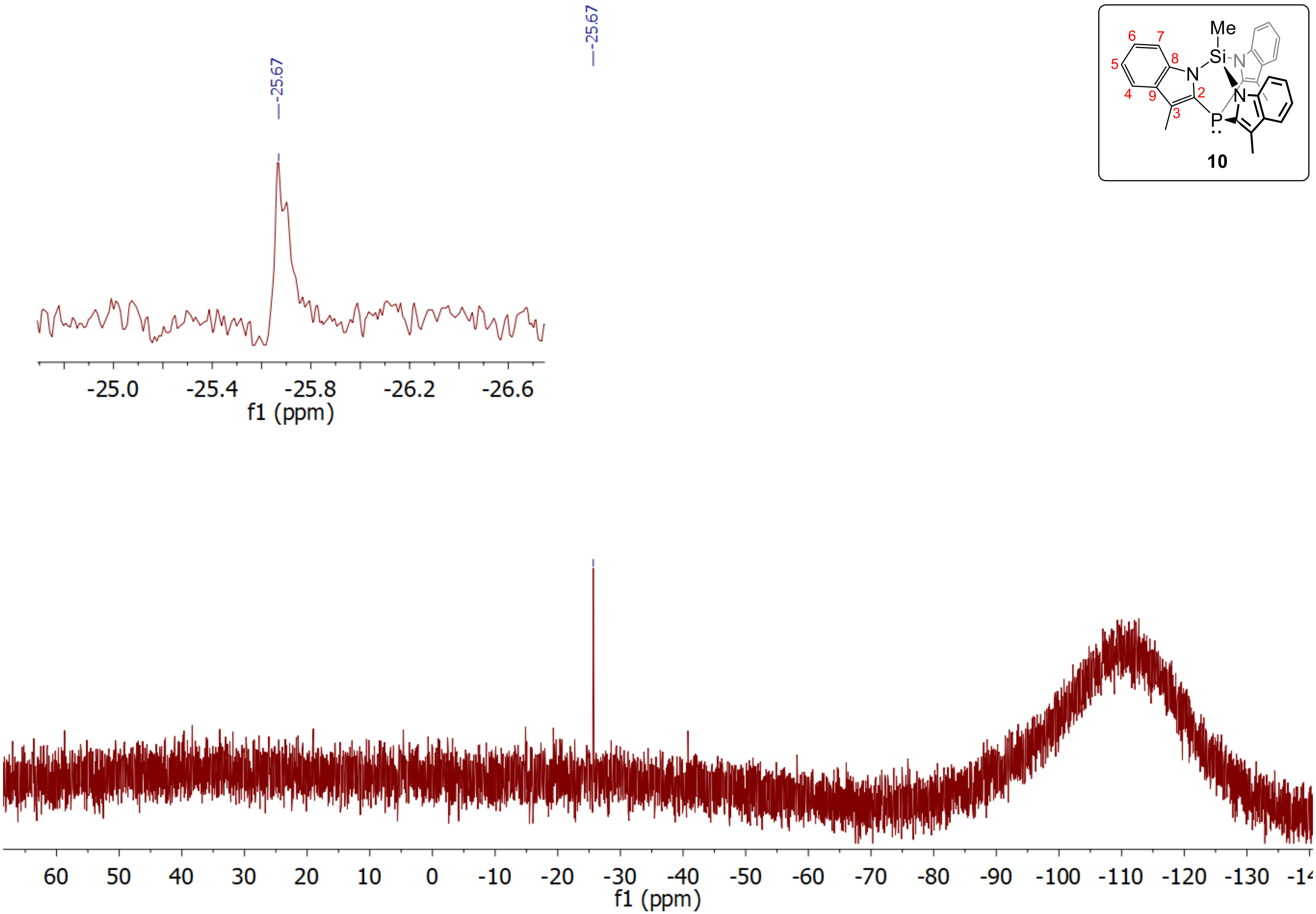

Figure S98. $^{29}Si\{^{1}H\}$ NMR (79 MHz) spectrum of compound **10** in THF-d_8.

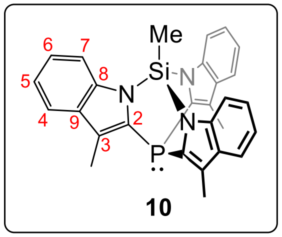

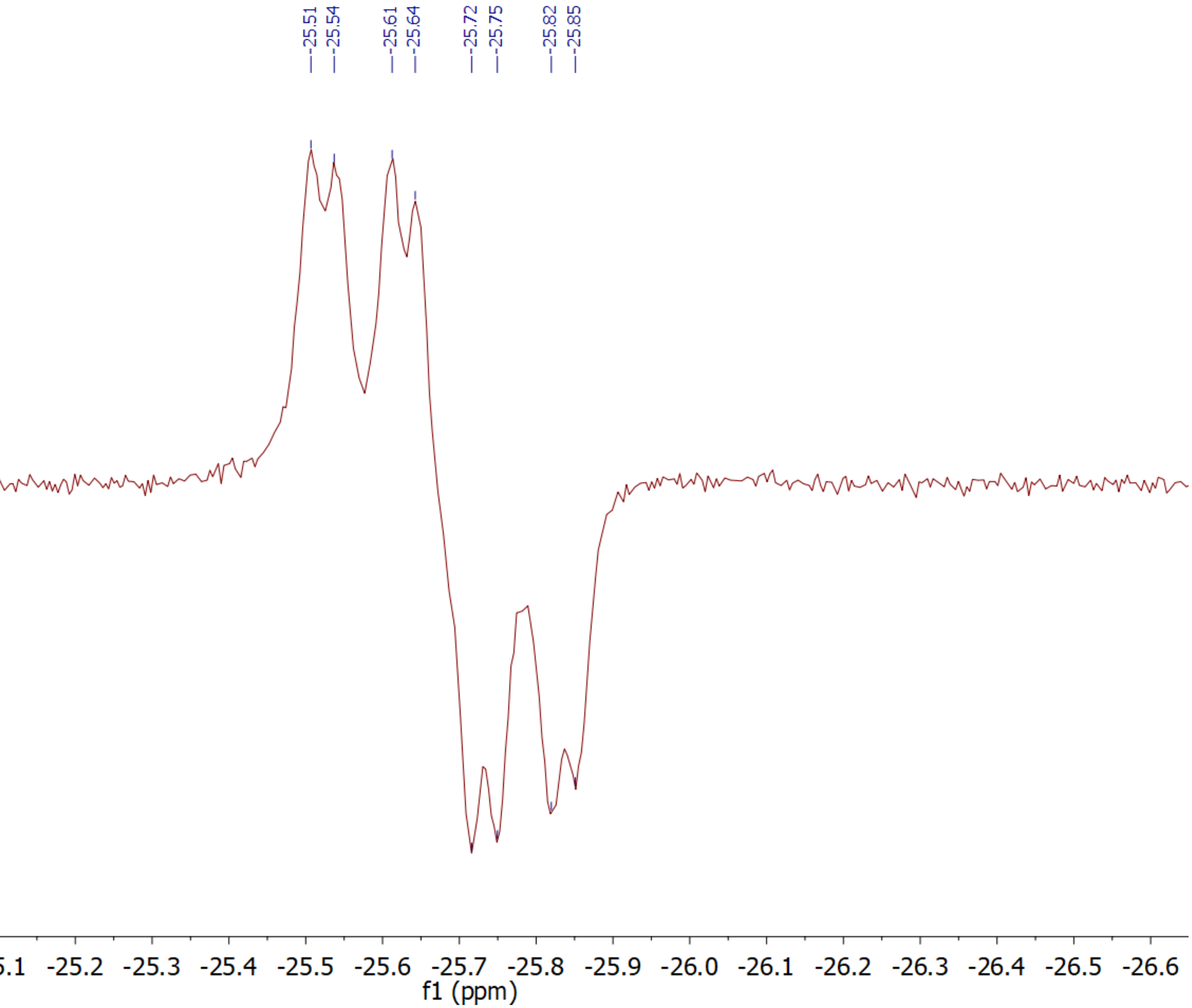

Figure S99. Non-refocused ^{29}Si INEPT (79 MHz) spectrum of compound **10** in THF-d_8.

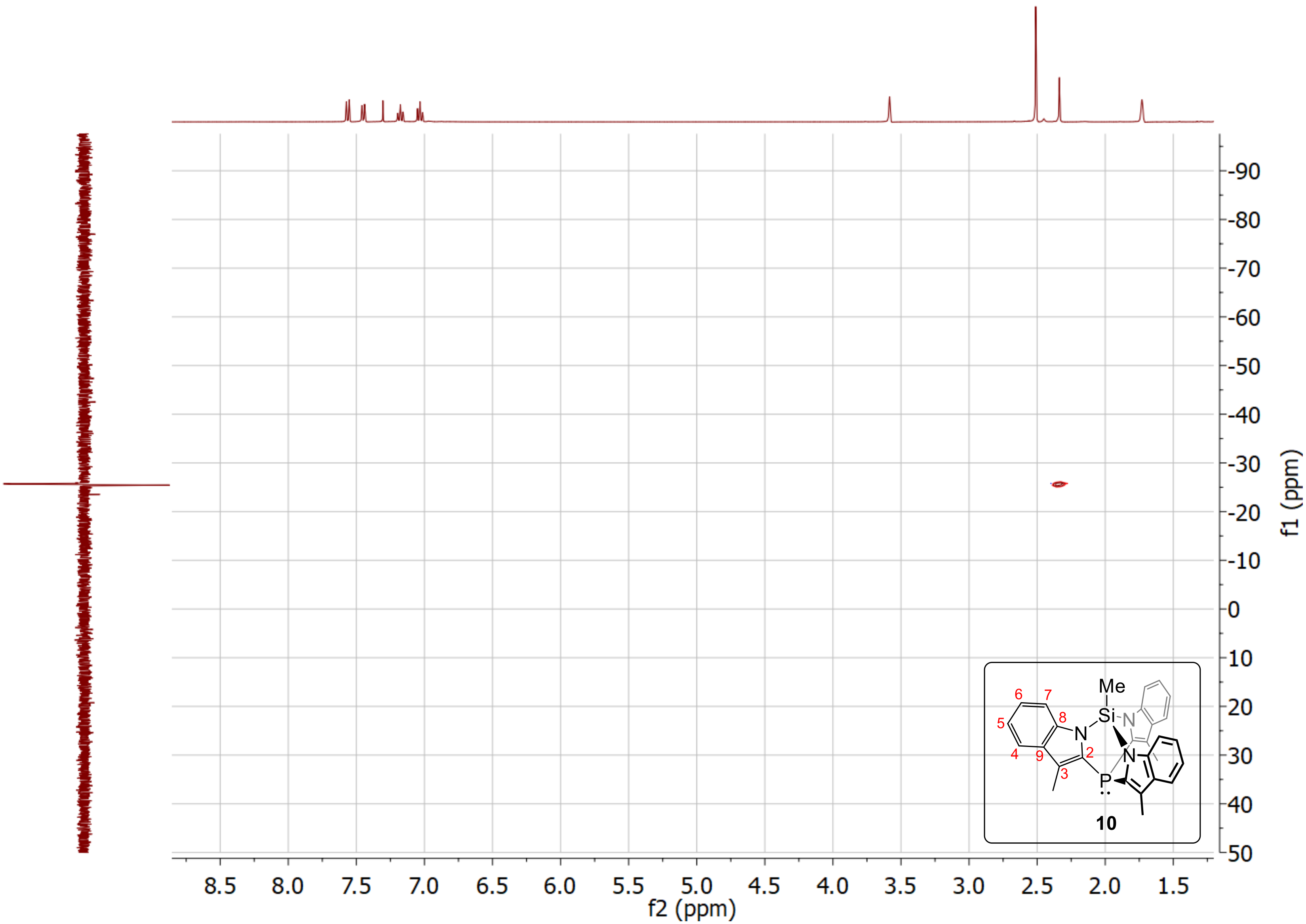

Figure S100. ^{1}H-^{29}Si gHMBC spectrum of compound **10** in THF-d_8 (vertical trace: non-refocused ^{29}Si INEPT, *Figure S99*).

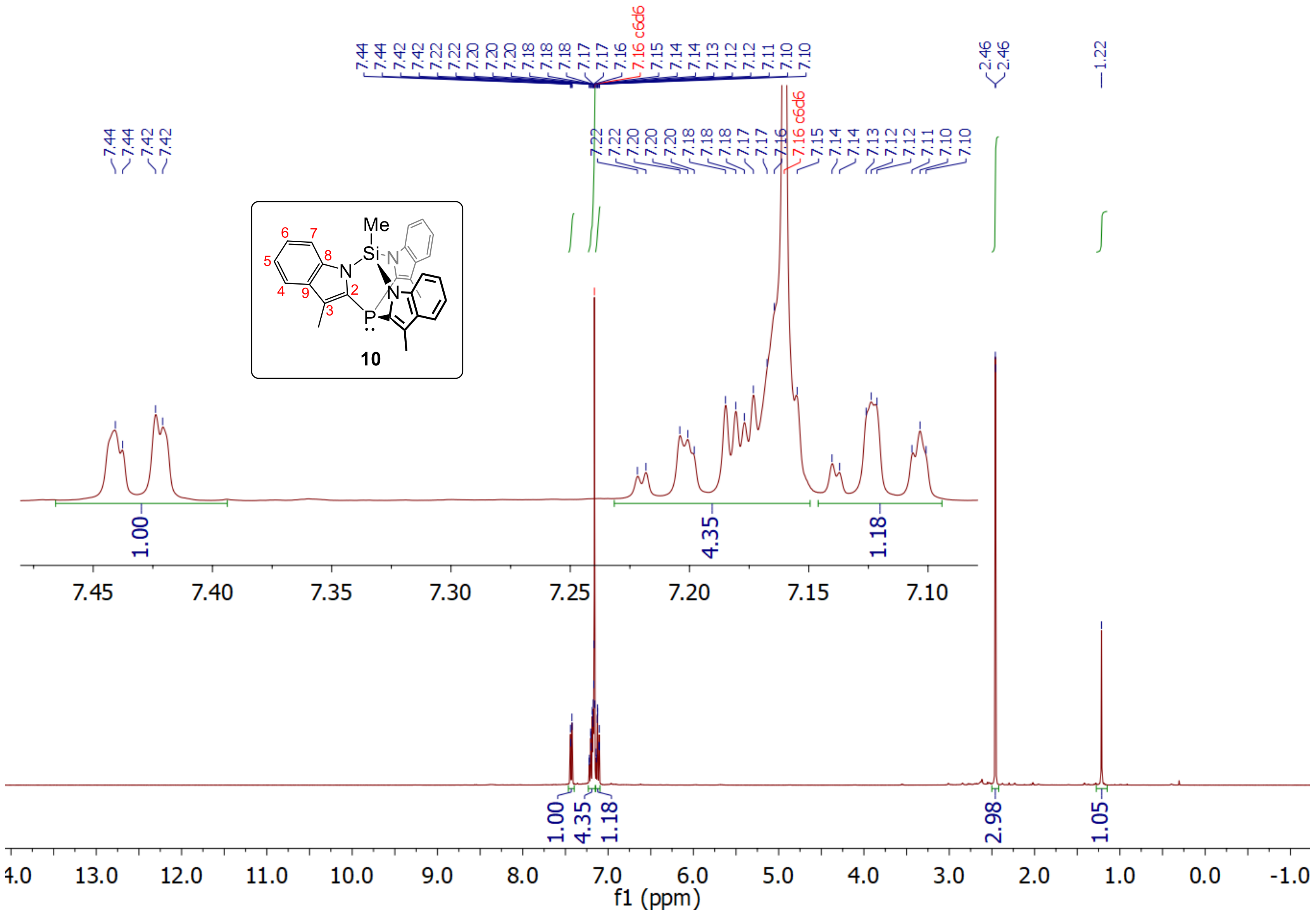

Figure S101. ^{1}H NMR (400 MHz) spectrum of compound **10** in benzene-d_6.

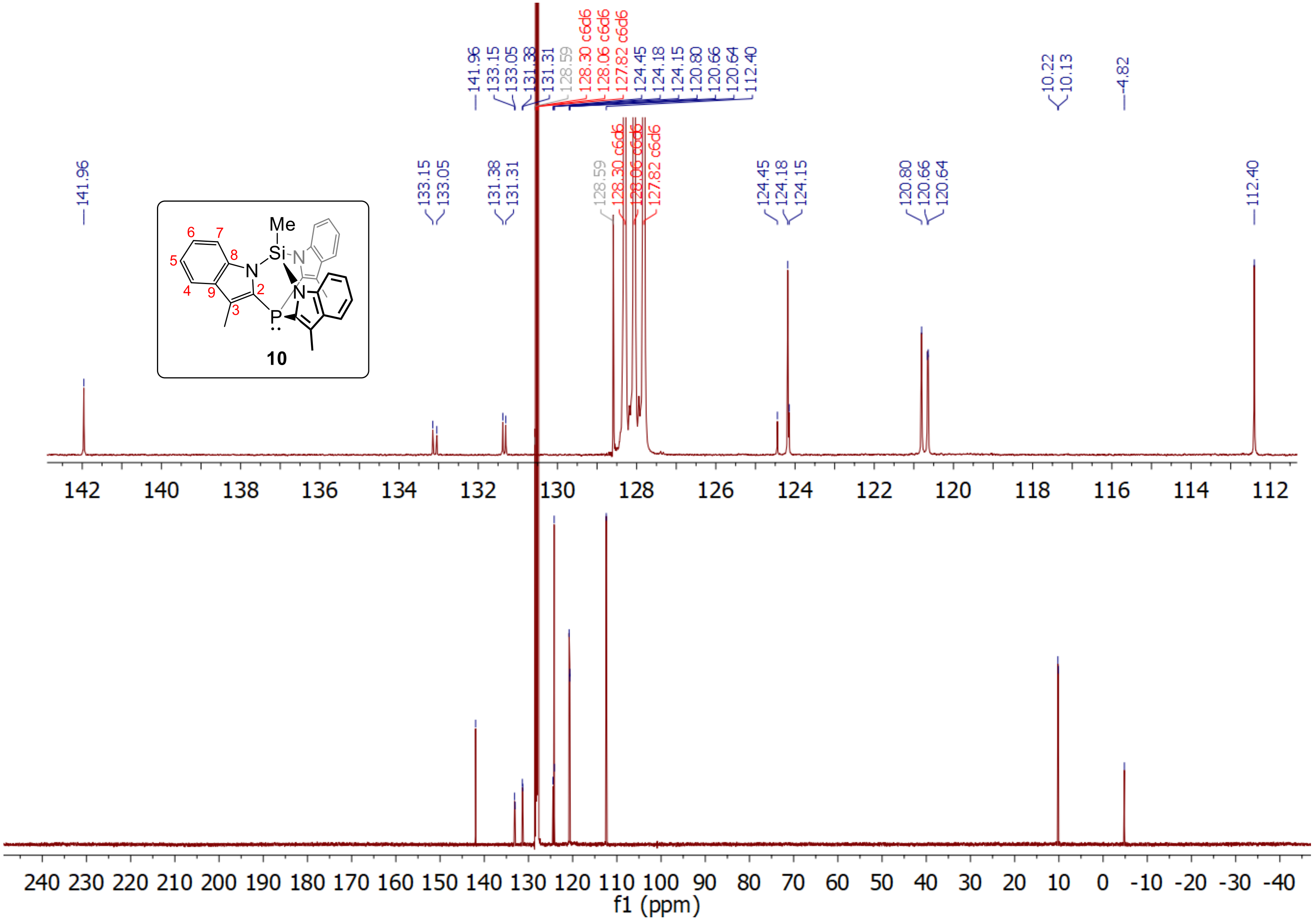

Figure S102. $^{13}C\{^{1}H\}$ NMR (101 MHz) spectrum of compound **10** in benzene-d_6. Impurities: 128.59 ppm – benzene-h_6.

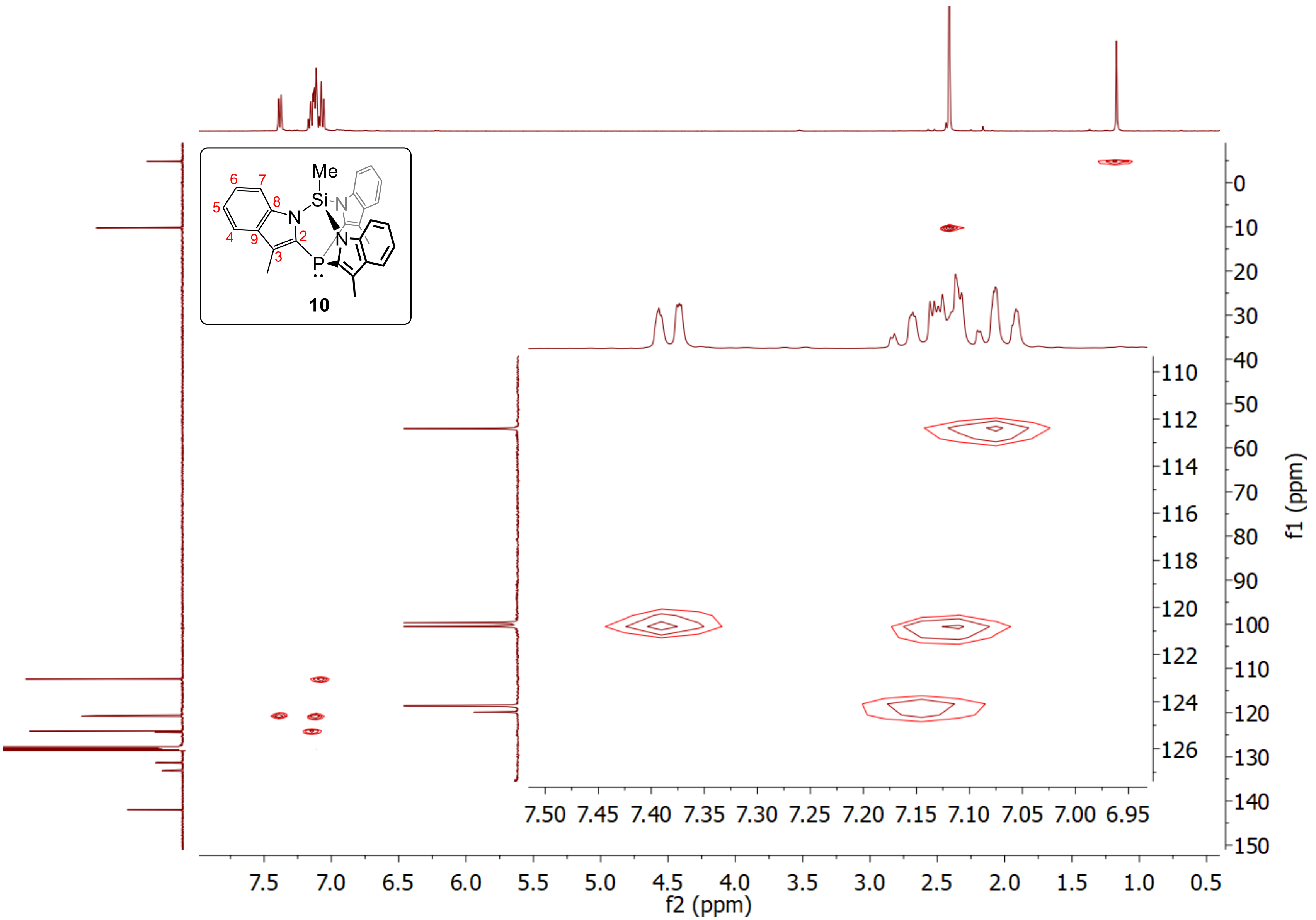

Figure S103. [1]H-^{13}C ASAPHMQC spectrum of compound **10** in benzene-d_6.

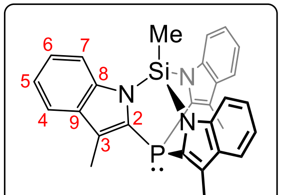

10

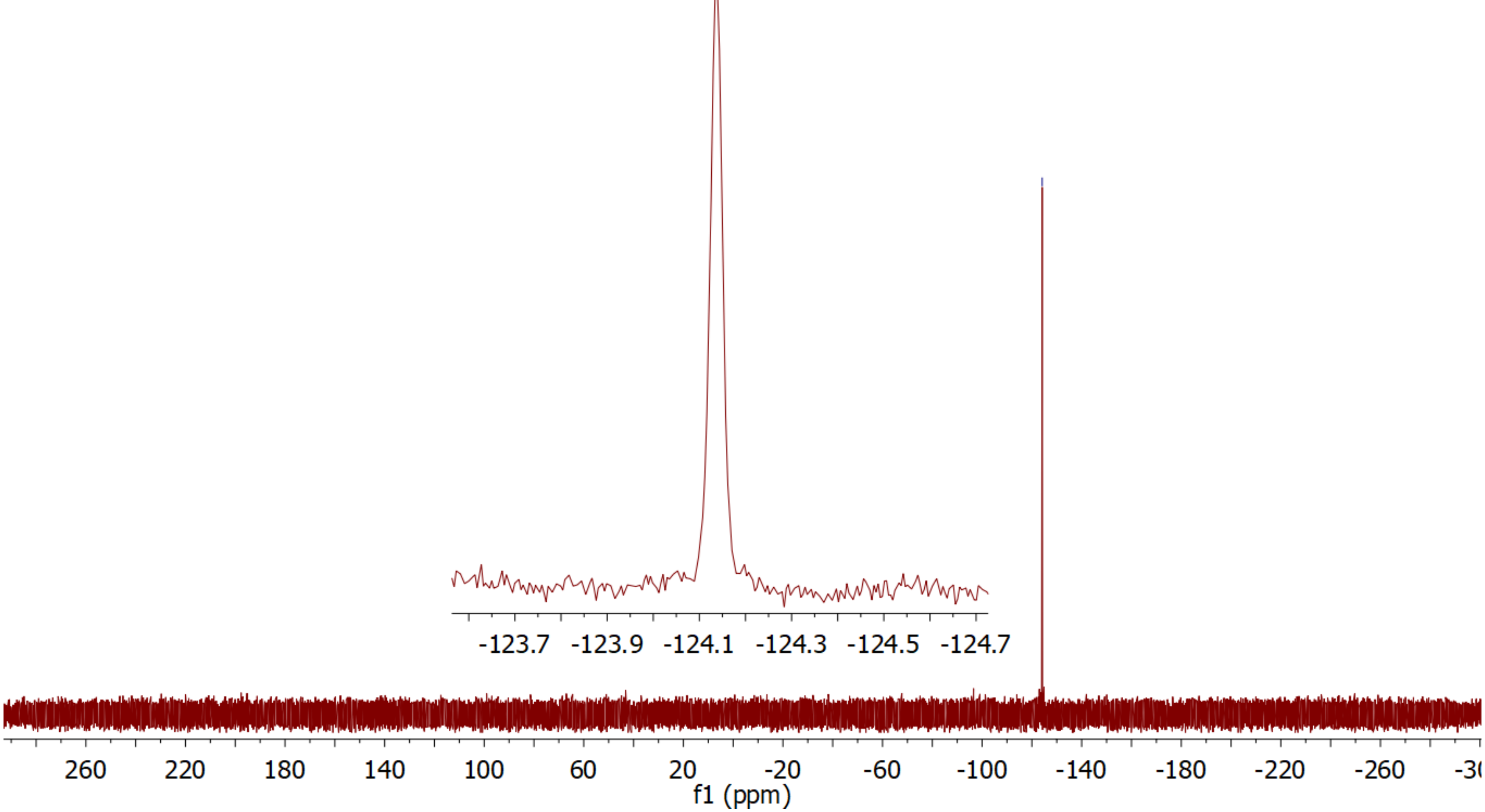

Figure S104. ^{31}P NMR (162 MHz) spectrum of compound **10** in benzene-d_6.

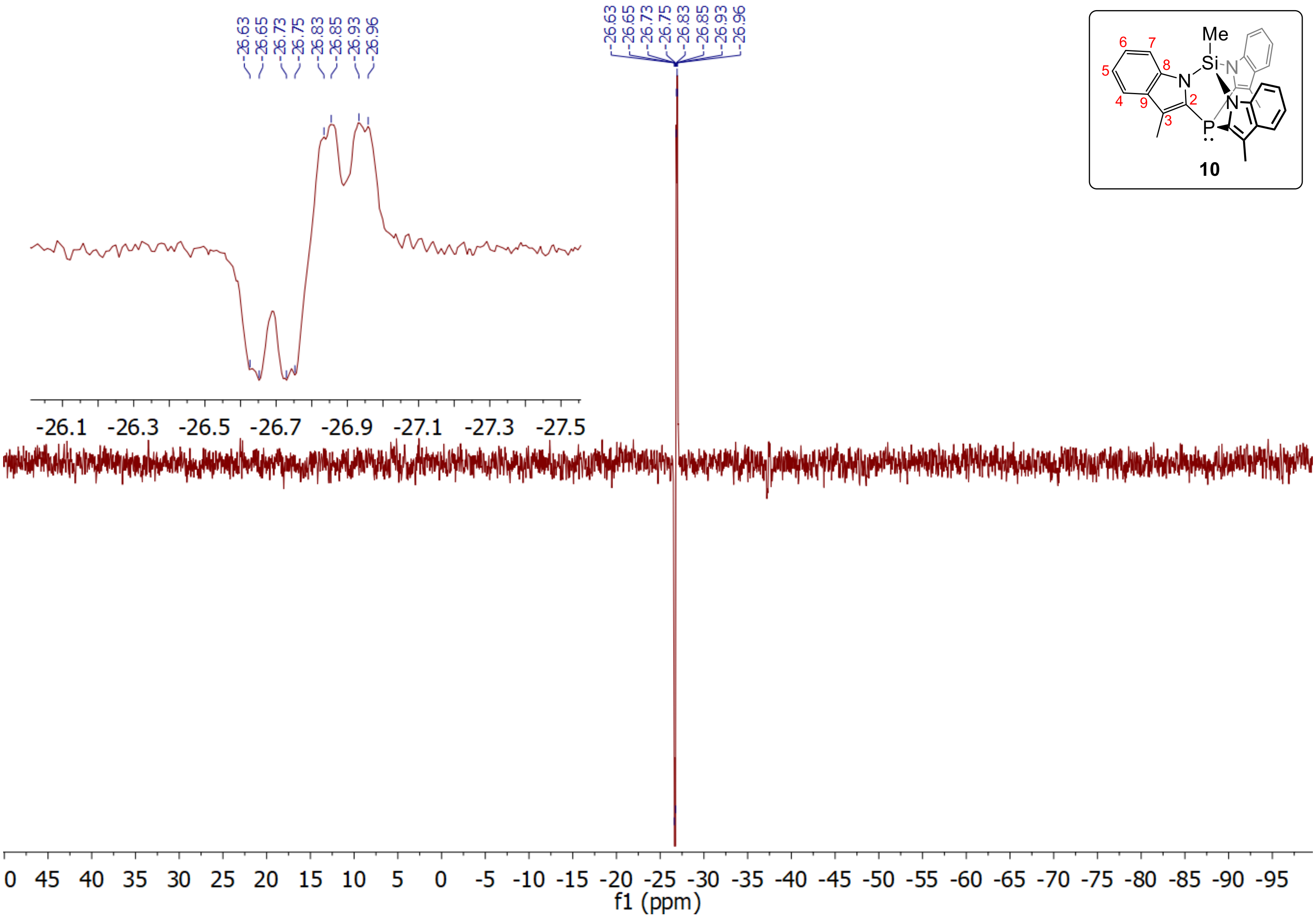

Figure S105. Non-refocused ^{29}Si INEPT (79 MHz) spectrum of compound **10** in benzene-d_6.

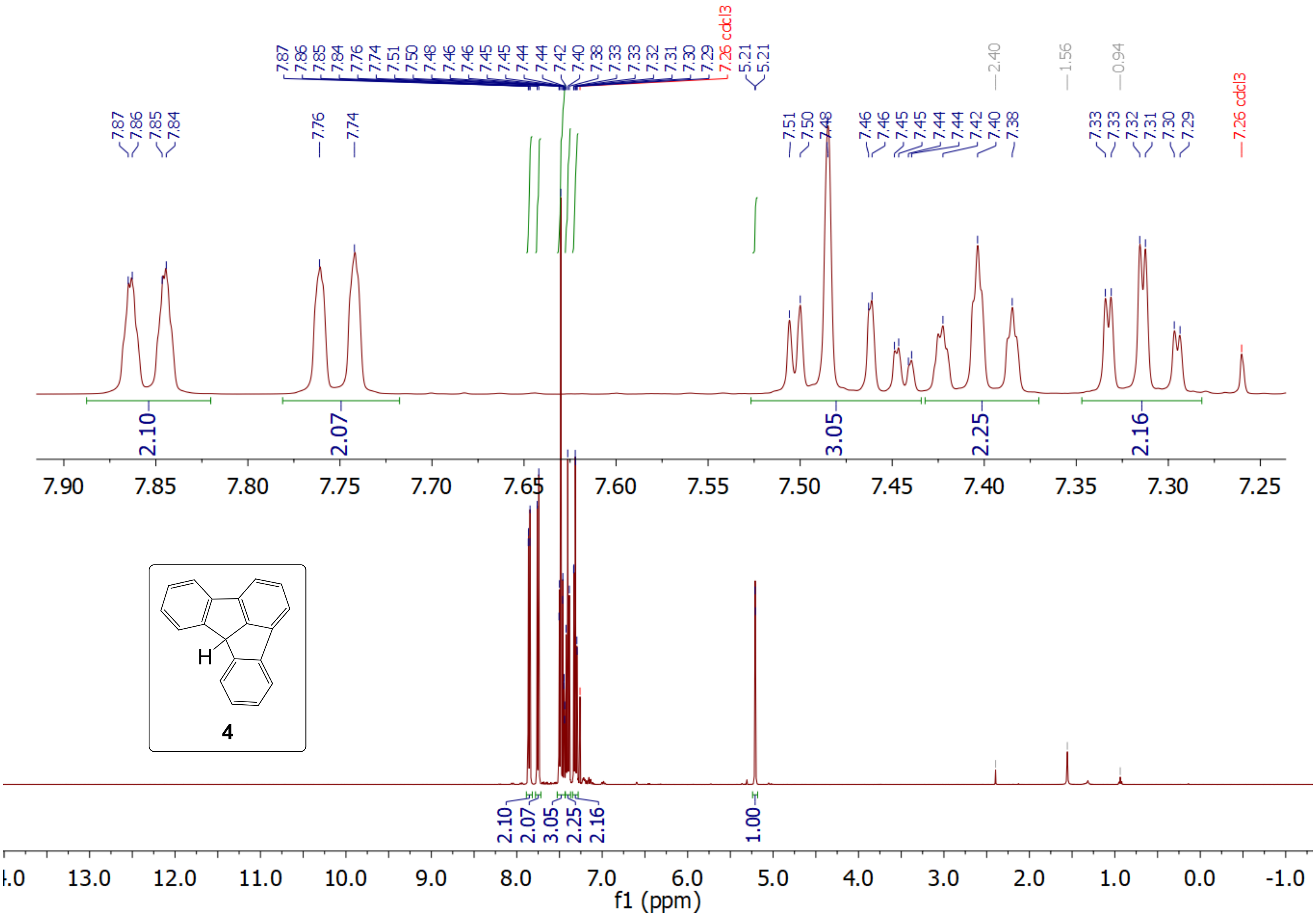

Figure S106. ^{1}H NMR (400 MHz) spectrum of fluoradene (**4**) in chloroform-*d*. Impurities: 1.56 ppm – water, other minor peaks are unidentified.

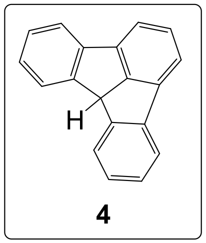

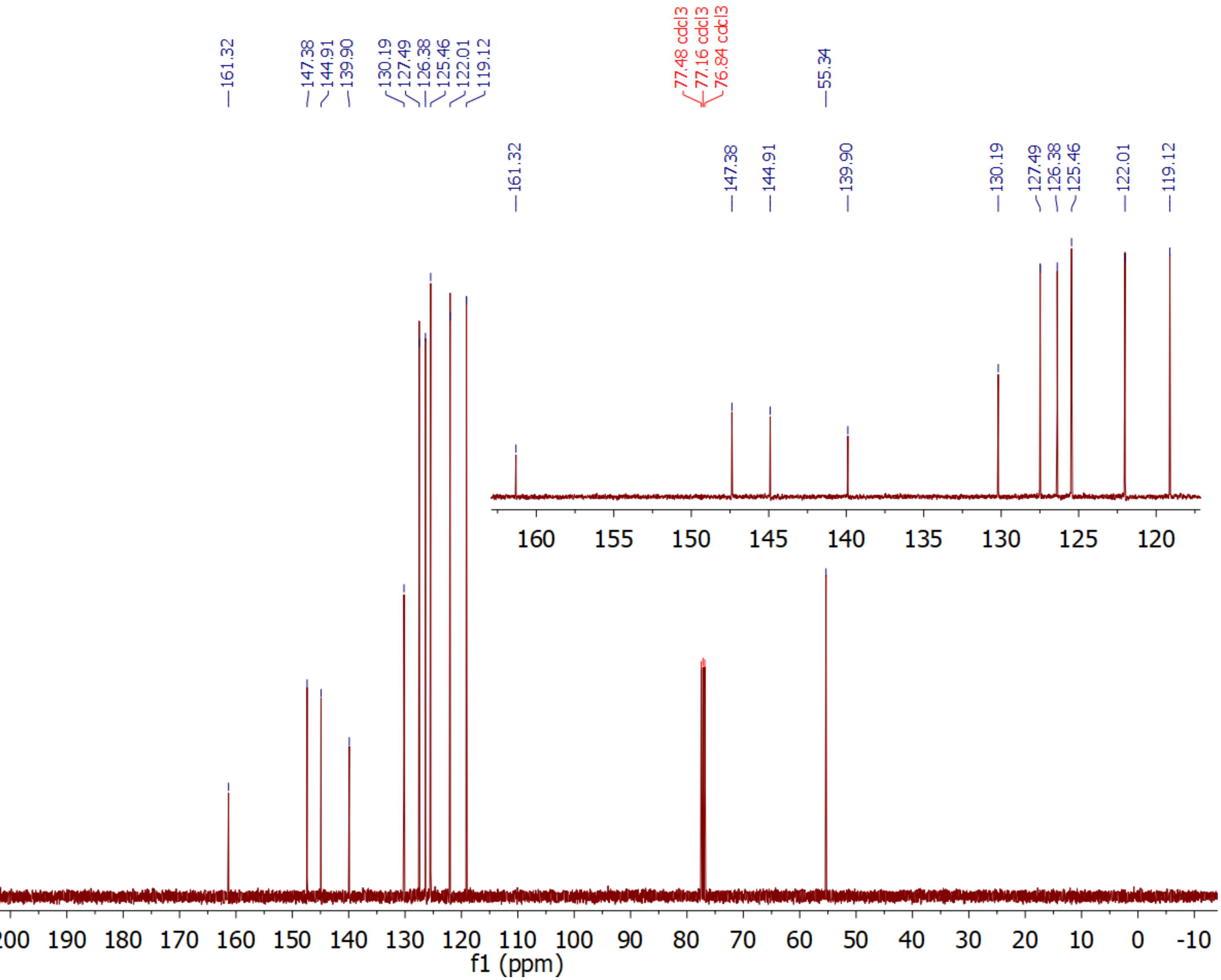

Figure S107. $^{13}C\{^{1}H\}$ NMR (101 MHz) spectrum of fluoradene (**4**) in chloroform-*d*.

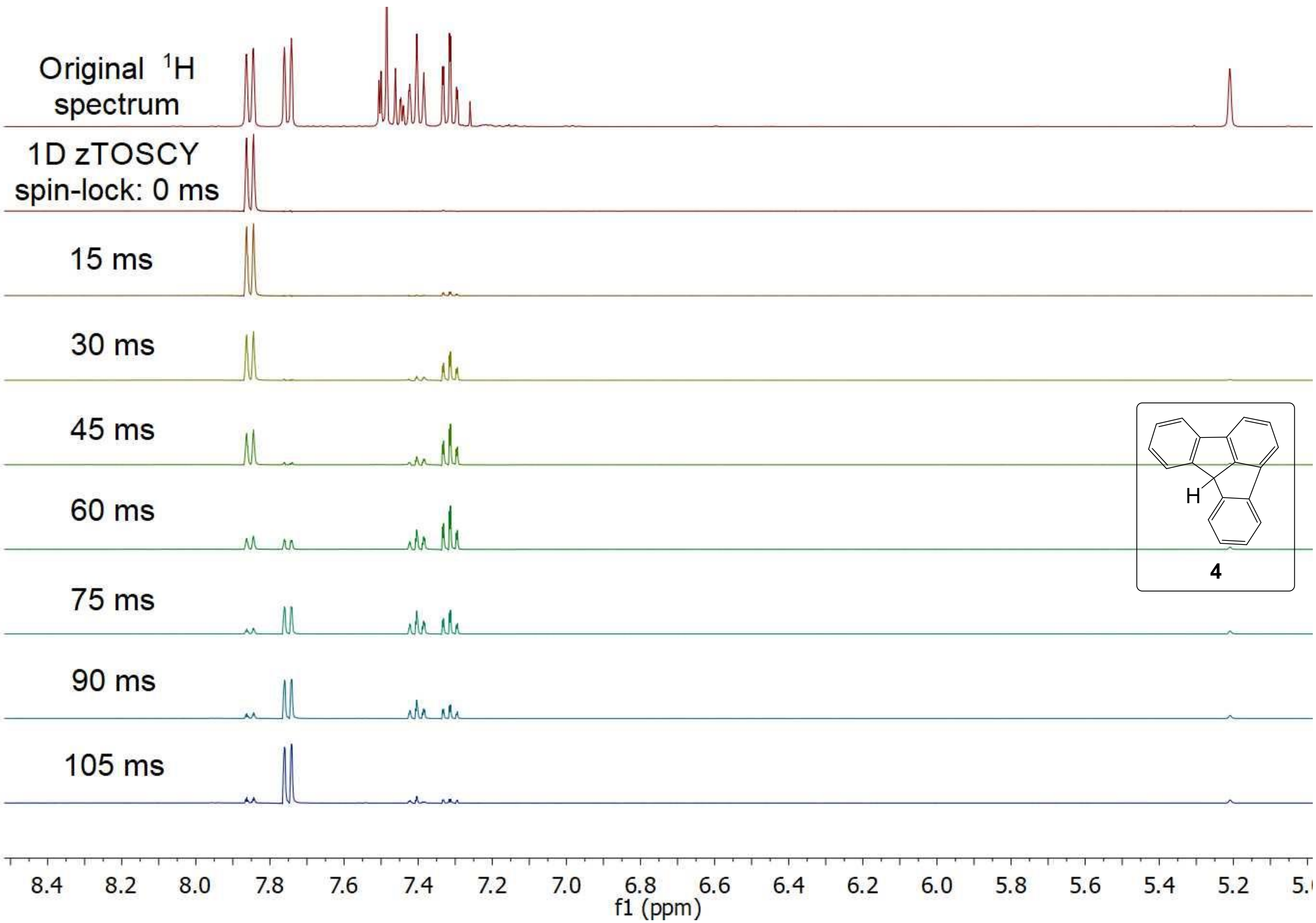

Figure S108. [1]H 1D zTOCSY (400 MHz) spectrum of fluoradene (**4**) in chloroform-*d*.

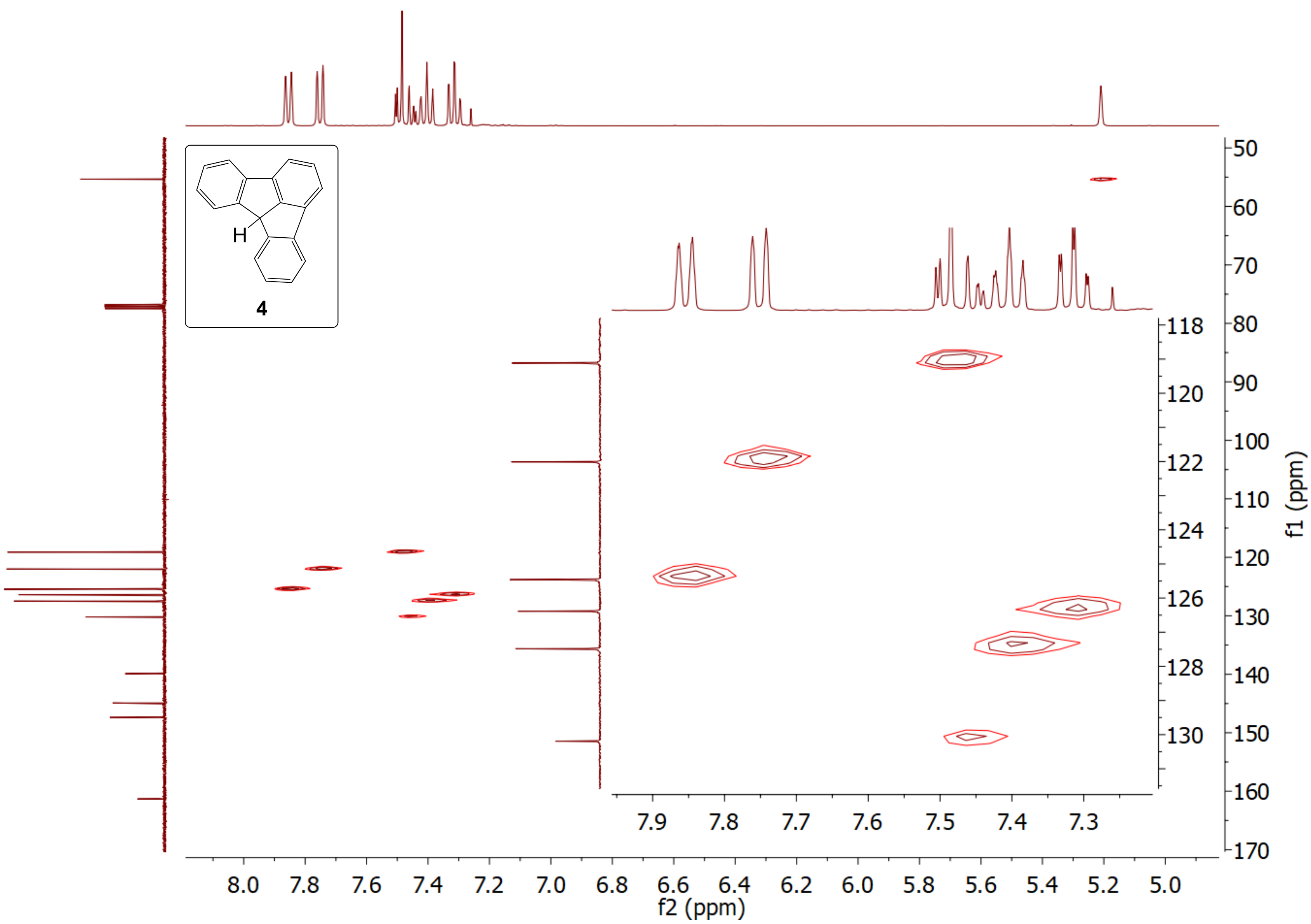

Figure S109. ^{1}H-^{13}C ASAPHMQC NMR spectrum of fluoradene (**4**) in chloroform-*d*.

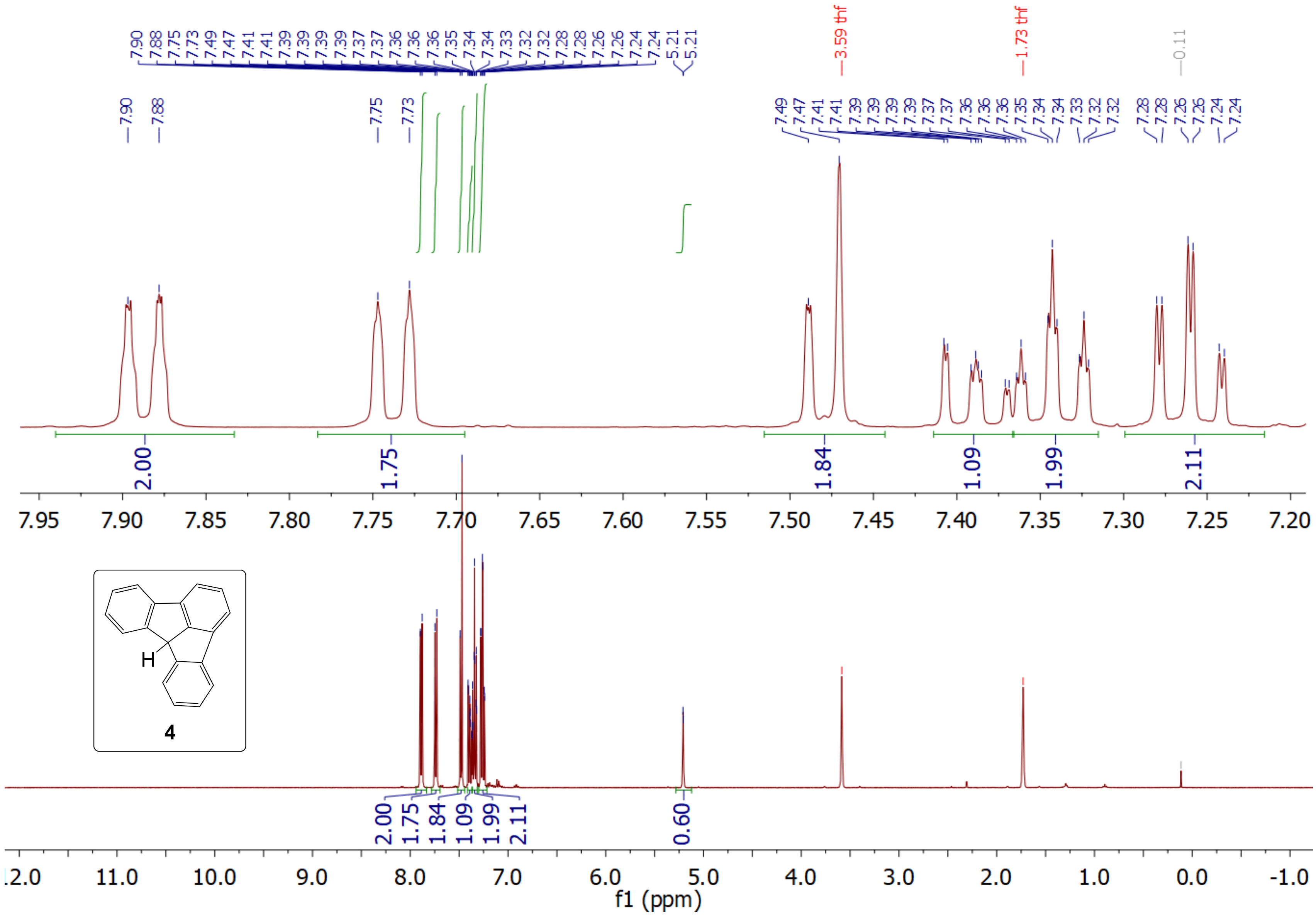

Figure S110. ^{1}H NMR (400 MHz) spectrum of fluoradene (**4**) in THF-d_8. Impurities: 0.11 ppm – grease.

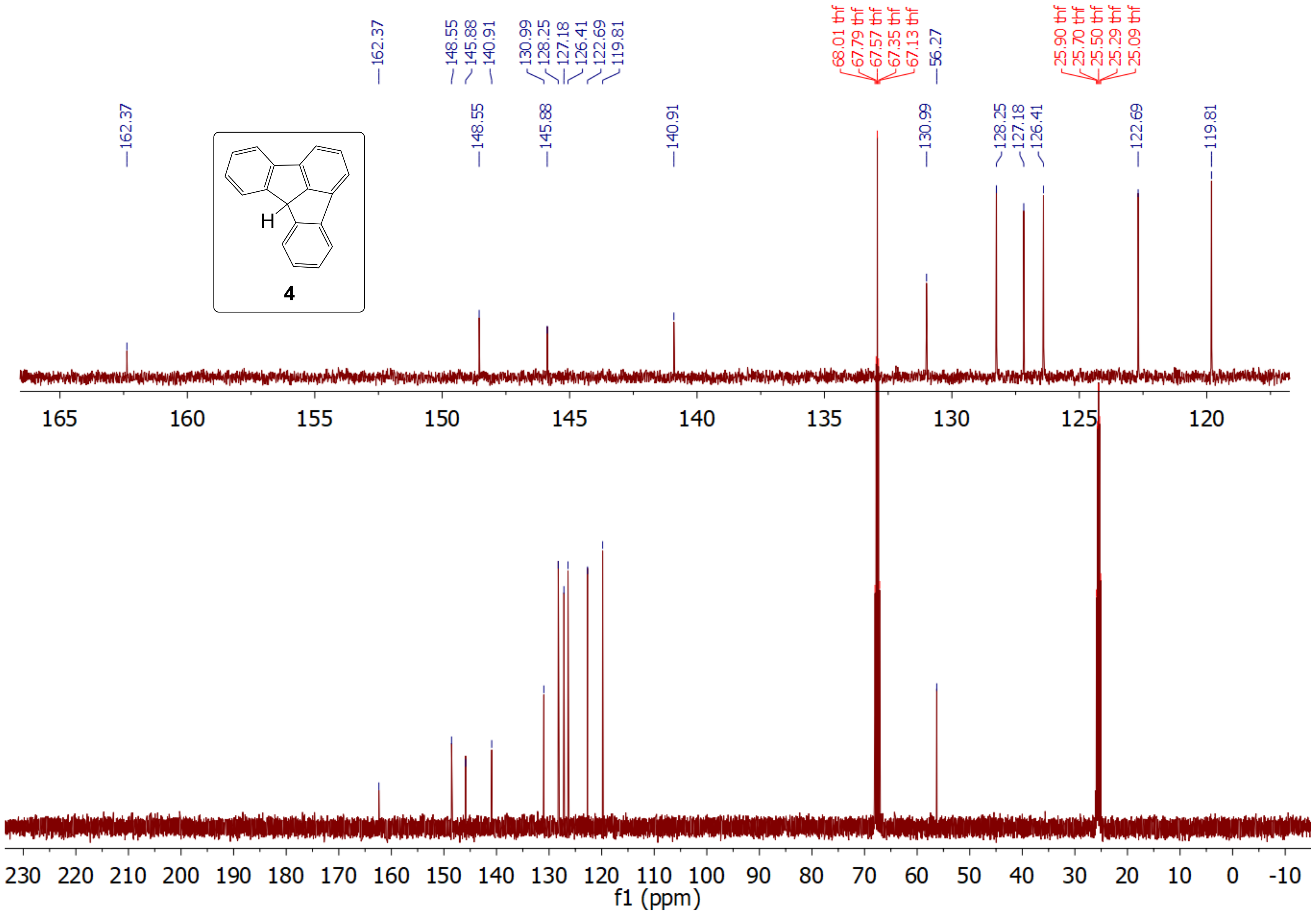

Figure S111. $^{13}C\{^{1}H\}$ NMR (101 MHz) spectrum of fluoradene (**4**) in THF-d_8.

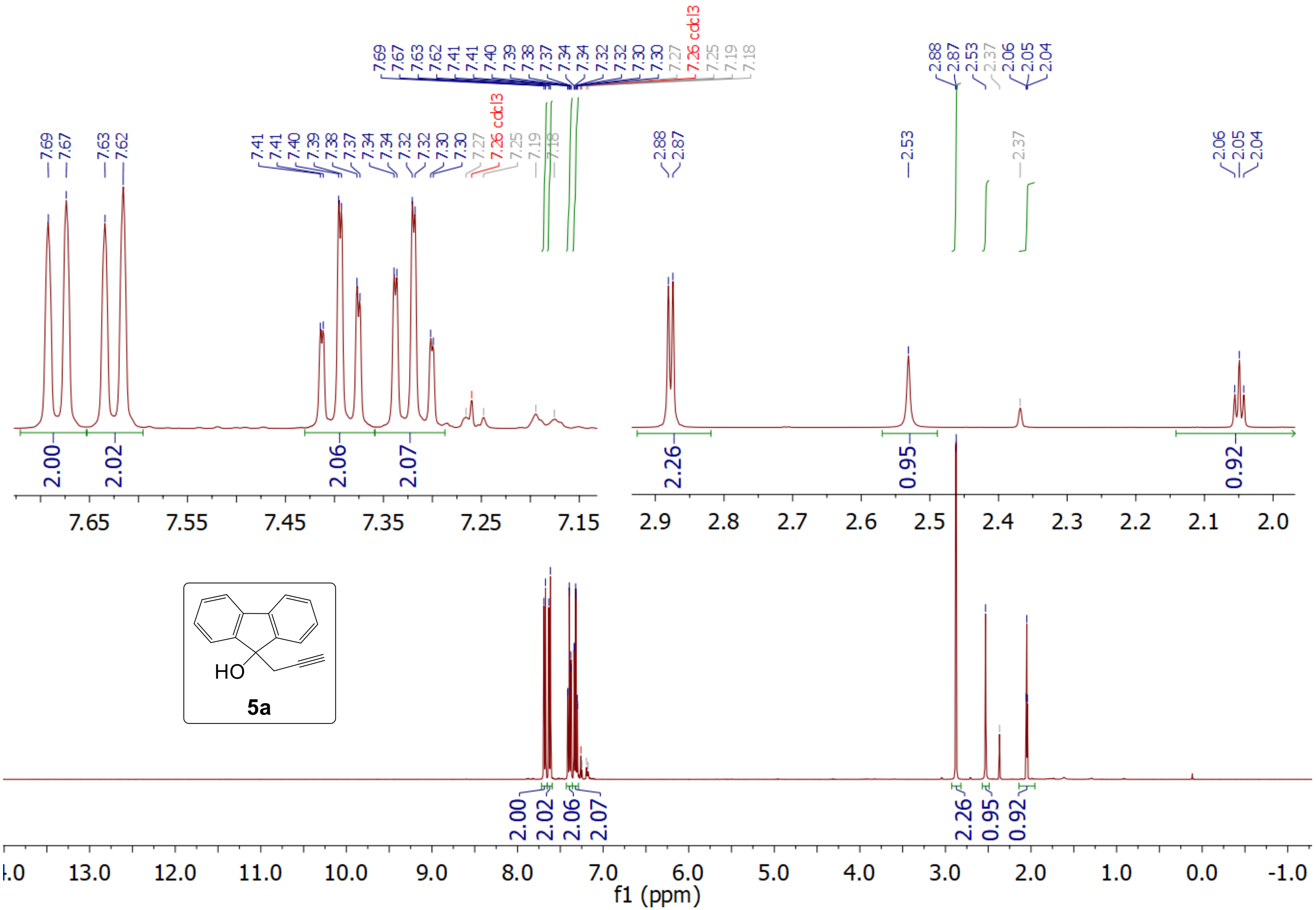

Figure S112. ^{1}H NMR (400 MHz) spectrum of compound **5a** in chloroform-*d*. Impurities: 2.37, 7.18-7.27 ppm – traces of toluene.

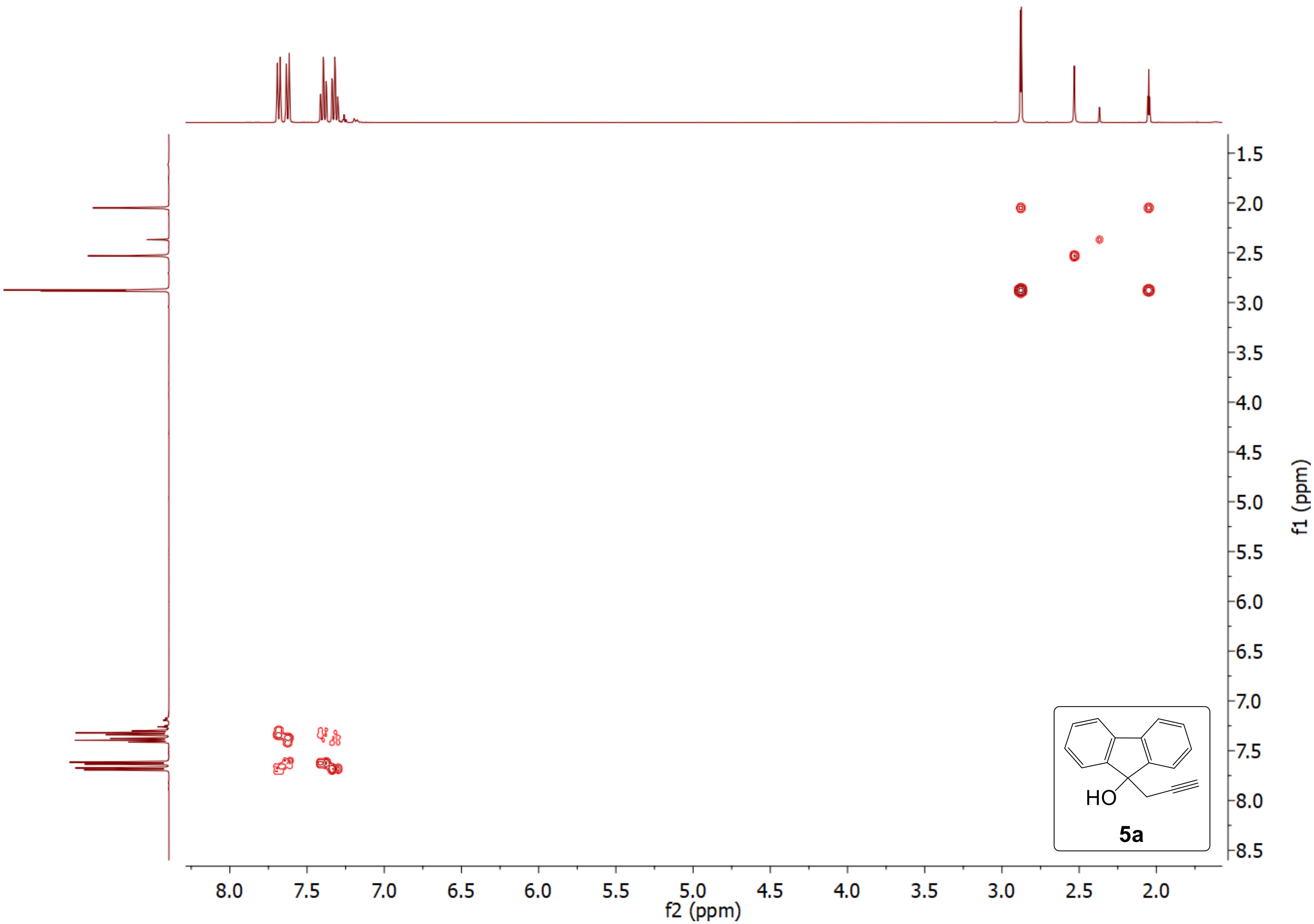

Figure S113. ^{1}H gCOSY (400 MHz) spectrum of compound **5a** in chloroform-*d*.

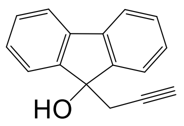

5a

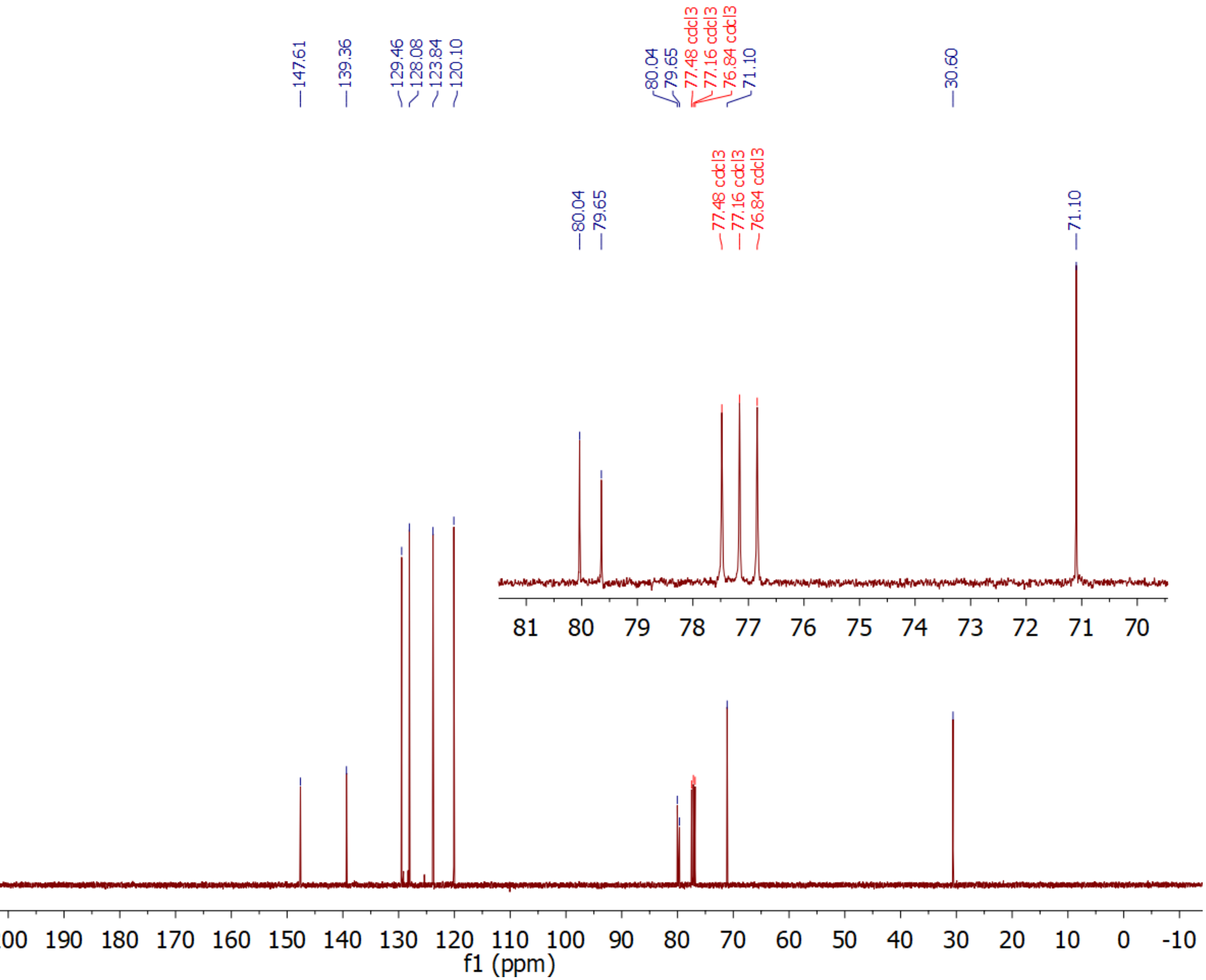

Figure S114. $^{13}C\{^{1}H\}$ NMR (101 MHz) spectrum of compound **5a** in chloroform-*d*.

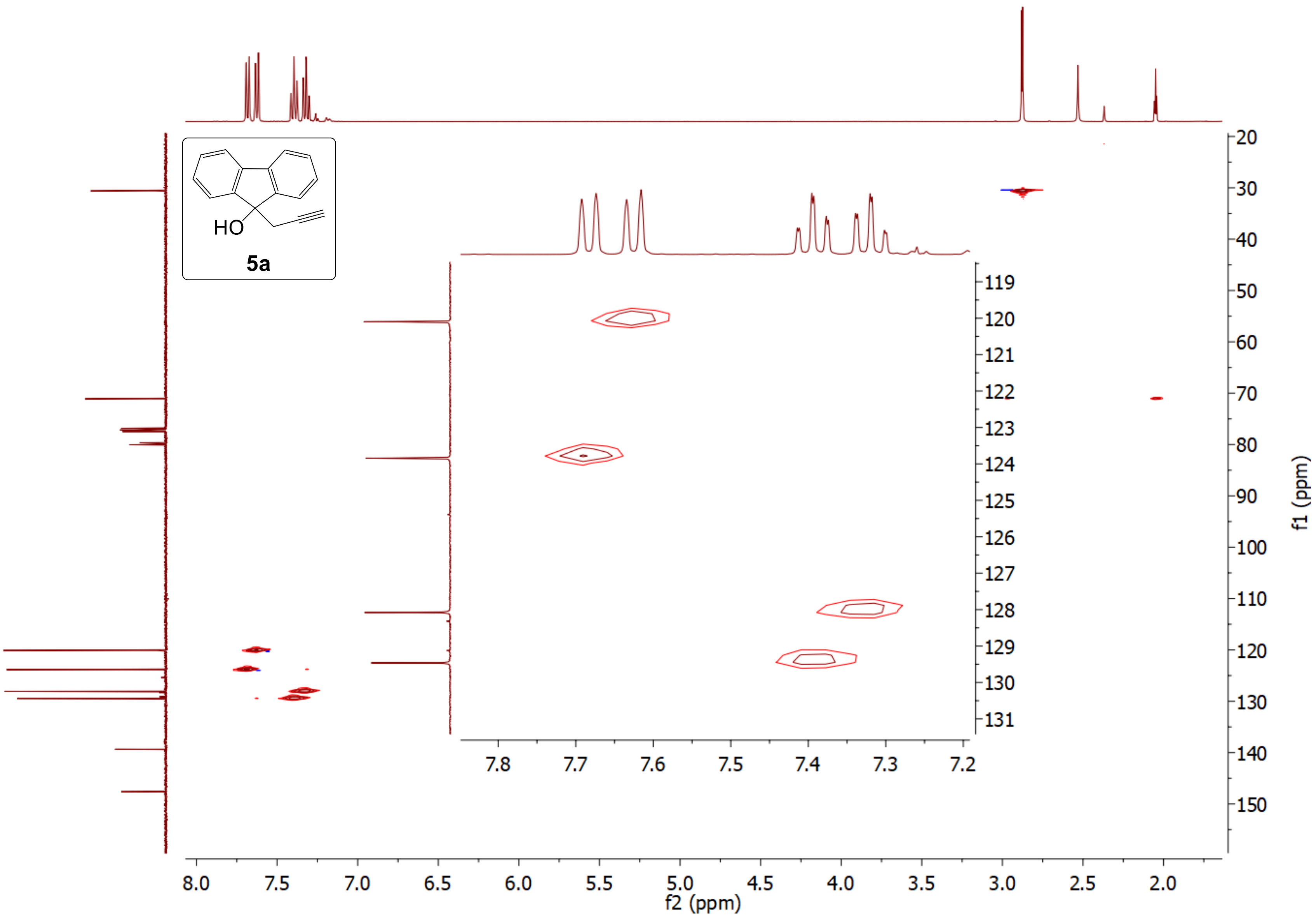

Figure S115. ^{1}H-^{13}C ASAPHMQC spectrum of compound **5a** in chloroform-*d*.

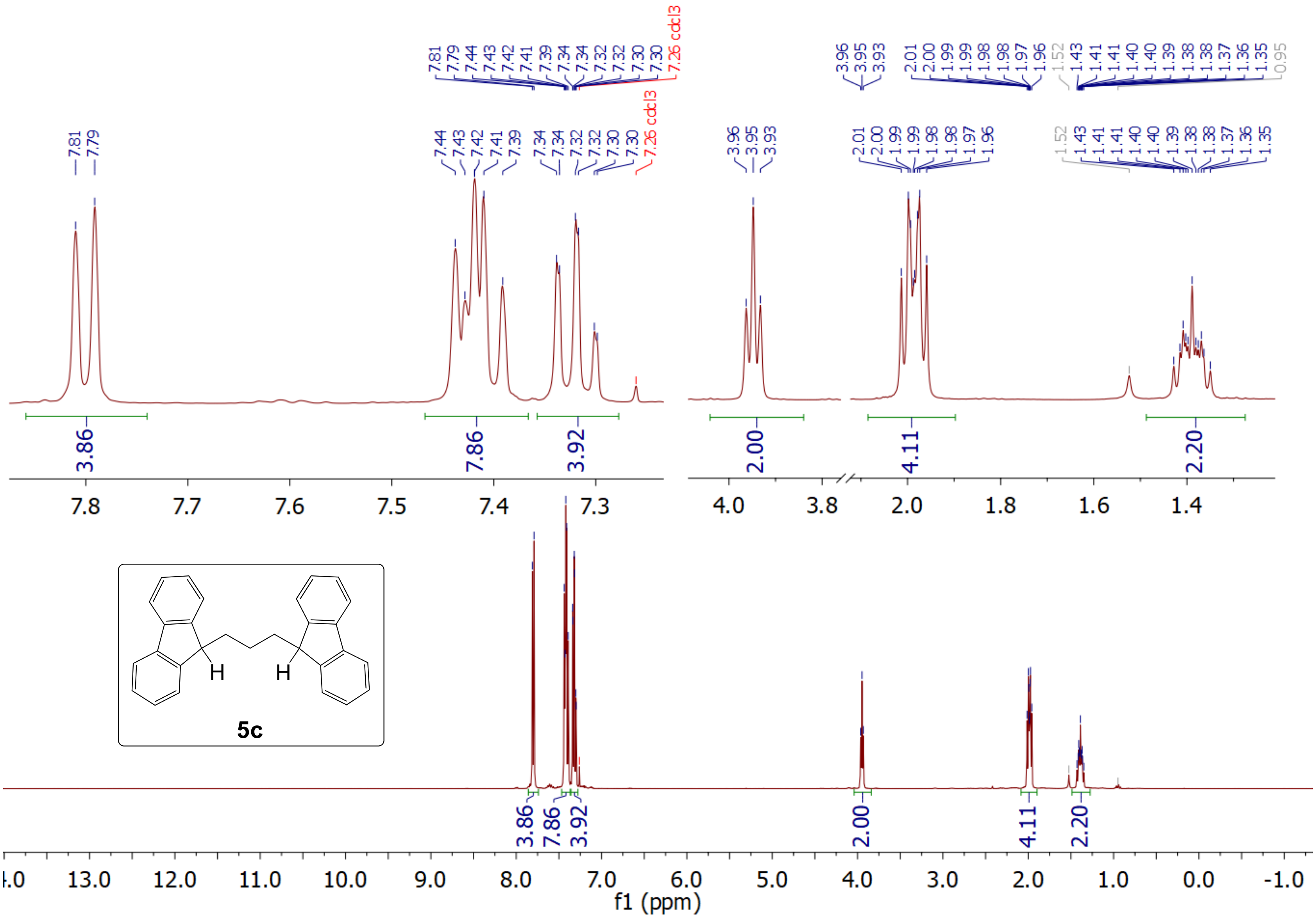

Figure S116. ^{1}H NMR (400 MHz) spectrum of compound **5c** in chloroform-*d*. Impurities: 1.52 ppm – traces of water.

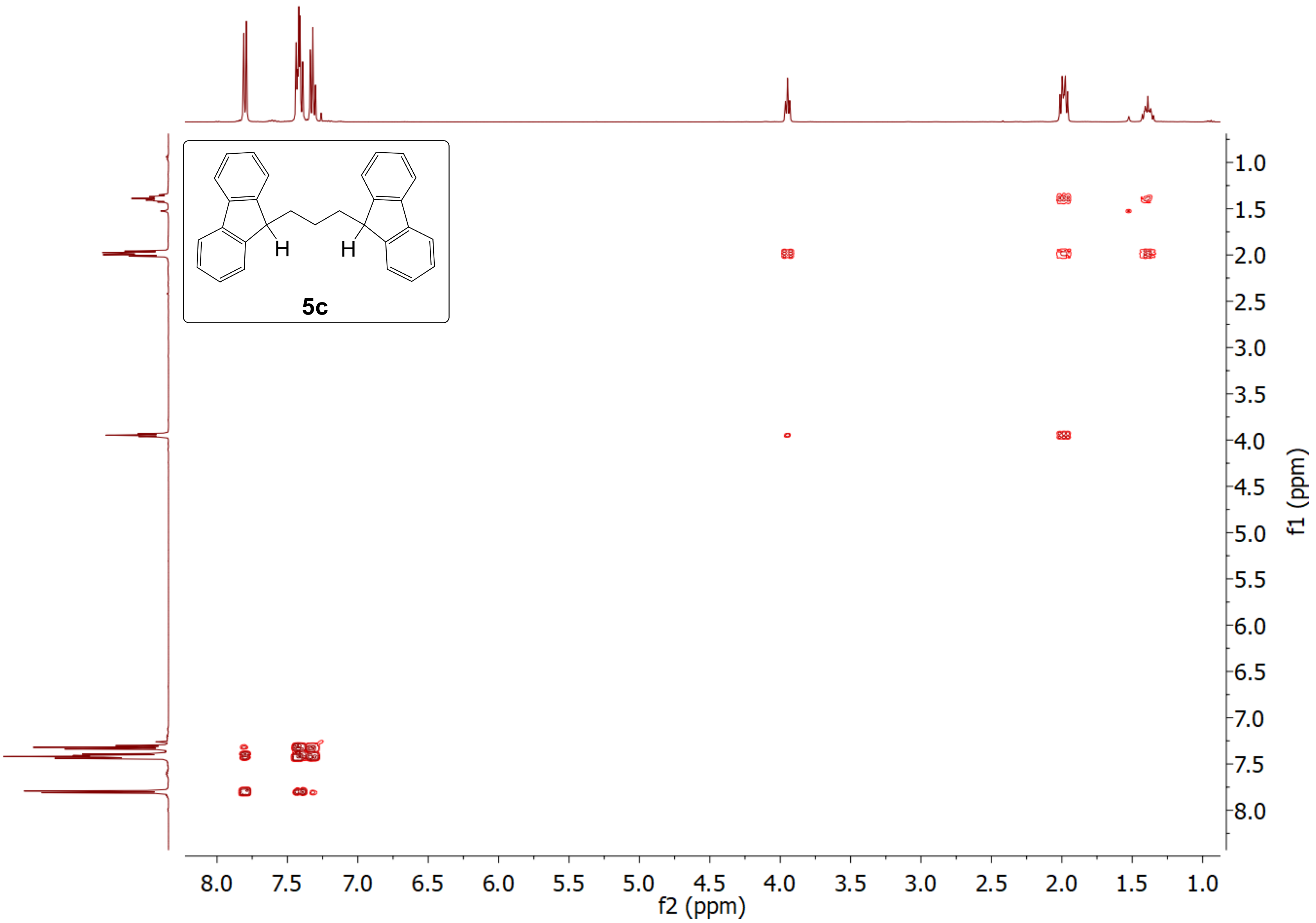

Figure S117. ¹H gCOSY (400 MHz) spectrum of compound **5c** in chloroform-*d*.

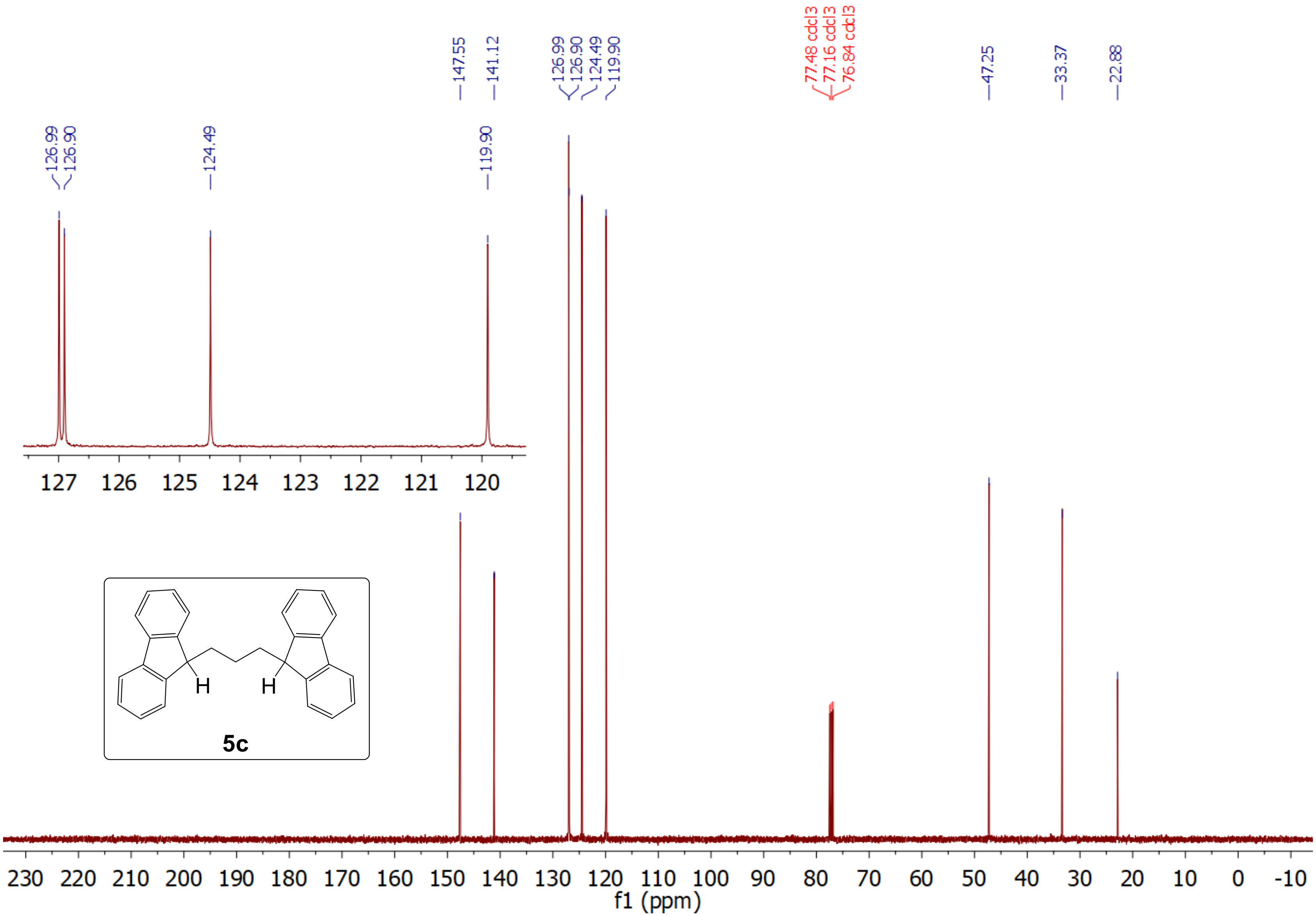

Figure S118. $^{13}C\{^1H\}$ NMR (101 MHz) spectrum of compound **5c** in chloroform-*d*.

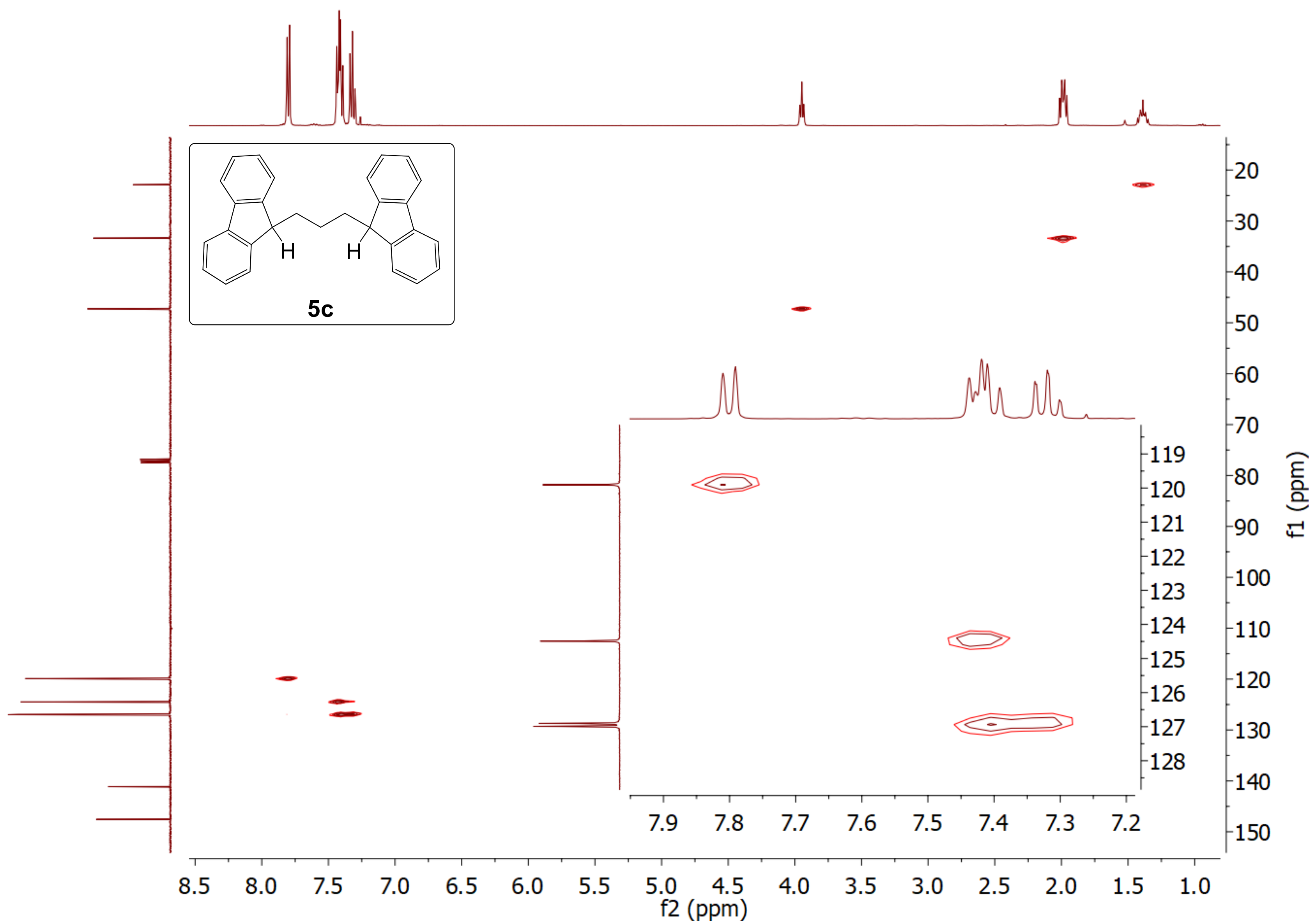

Figure S119. ^{1}H-^{13}C ASAPHMQC spectrum of compound **5c** in chloroform-*d*.

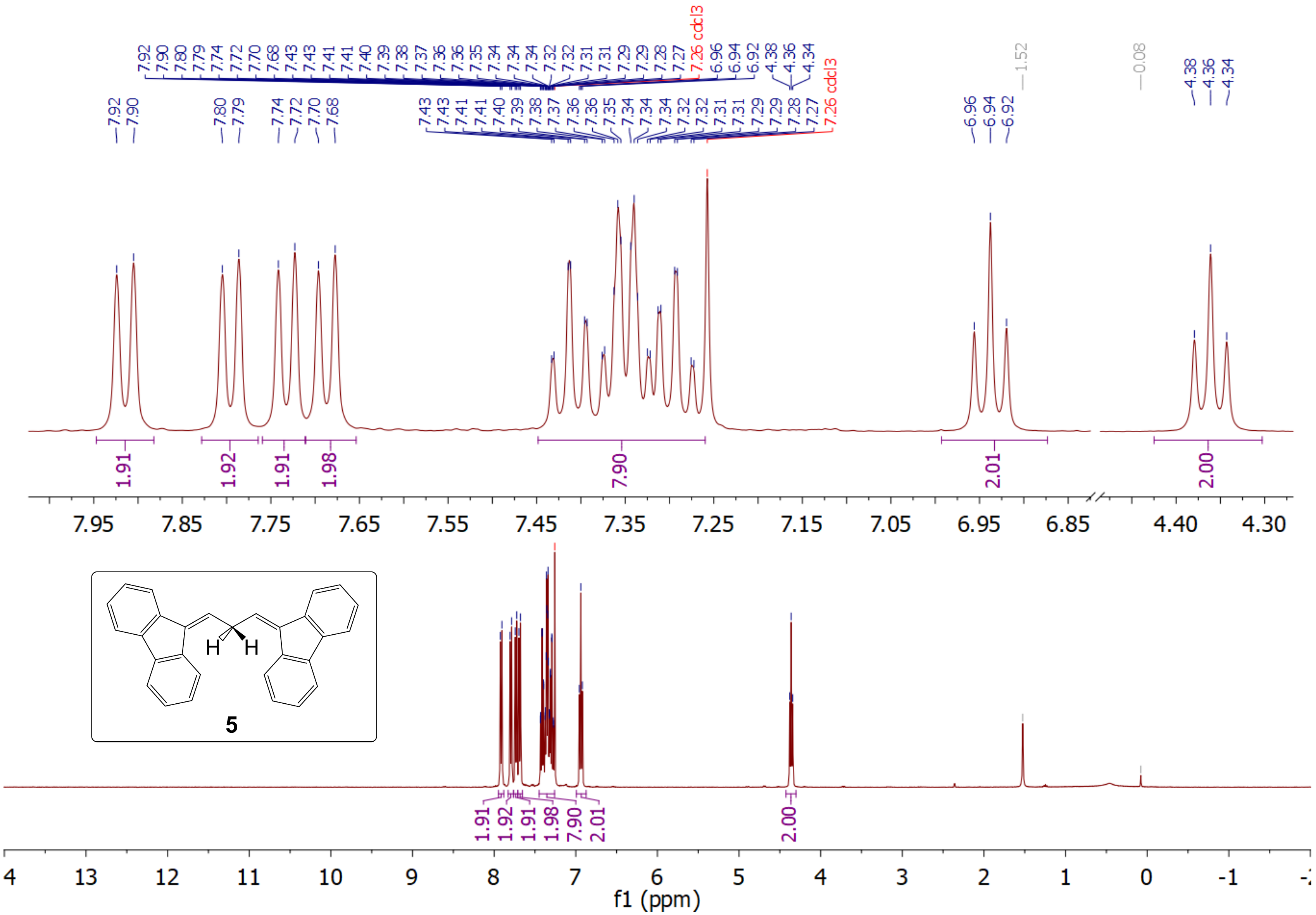

Figure S120. ^{1}H NMR (400 MHz) spectrum of compound **5** in chloroform-*d*. Impurities: 0.08 ppm – grease, 1.52 ppm – traces of water.

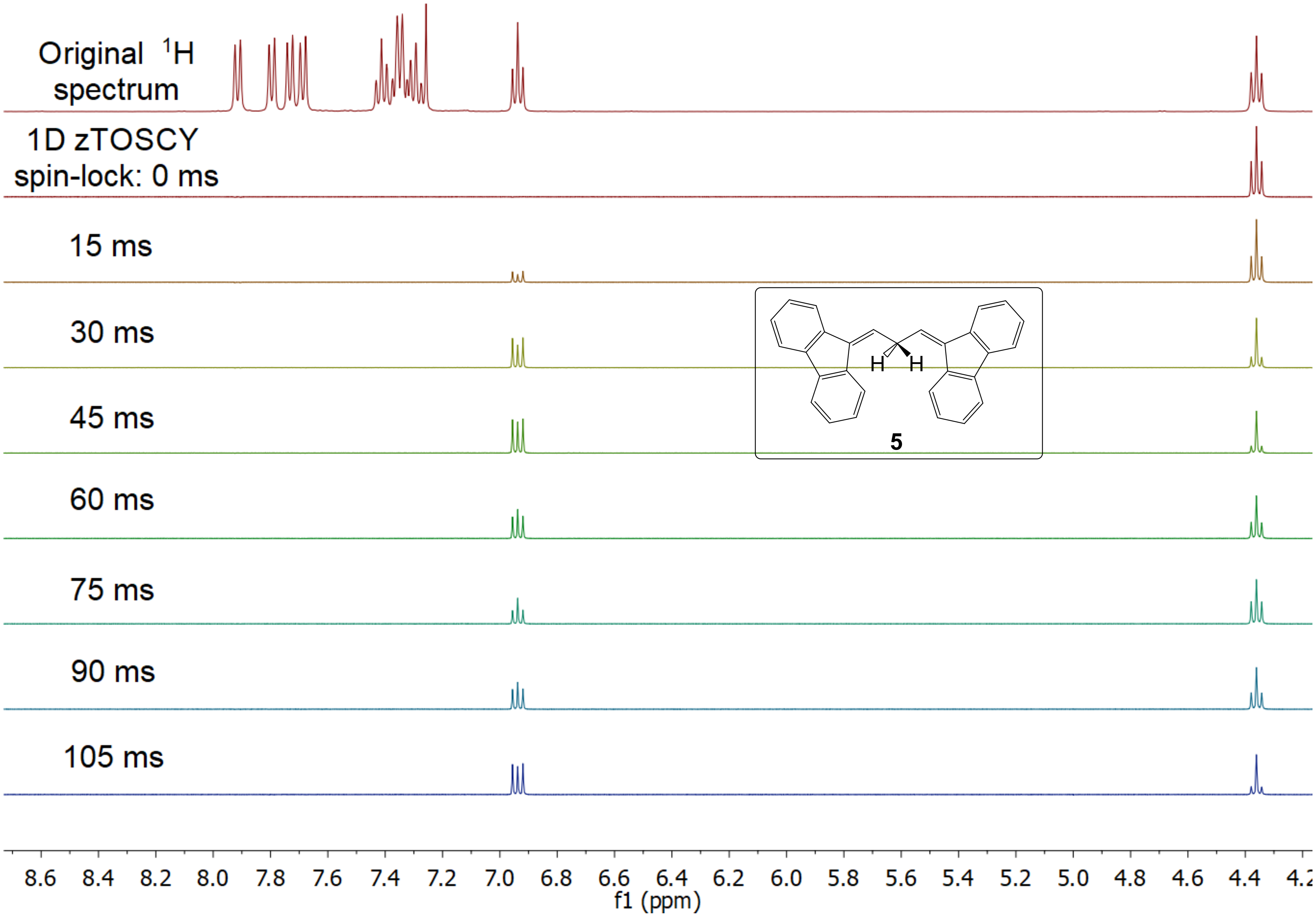

Figure S121. ^{1}H 1D zTOCSY (400 MHz) spectrum of compound **5** in chloroform-*d*.

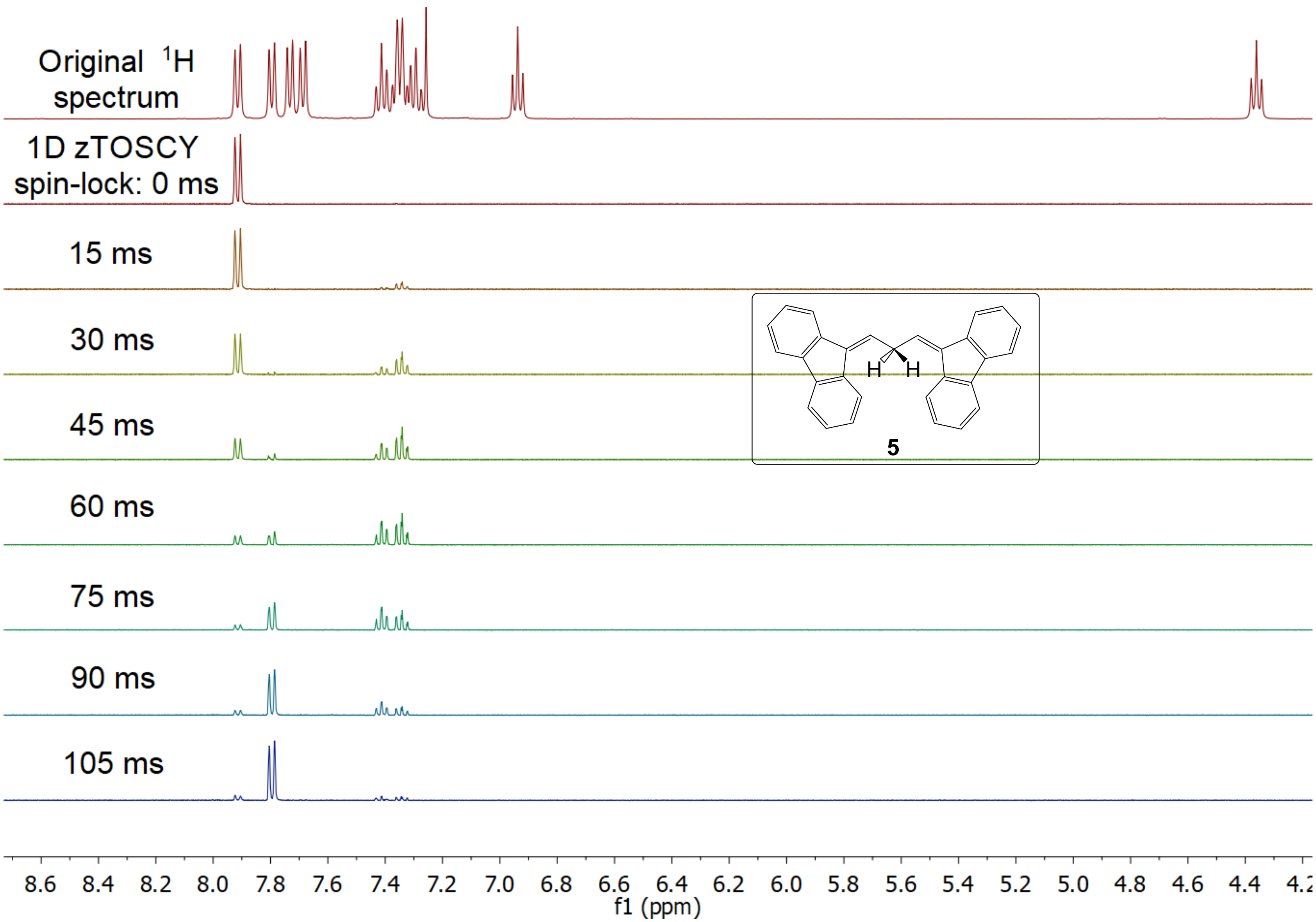

Figure S122. [1]H 1D zTOCSY (400 MHz) spectrum of compound **5** in chloroform-*d*.

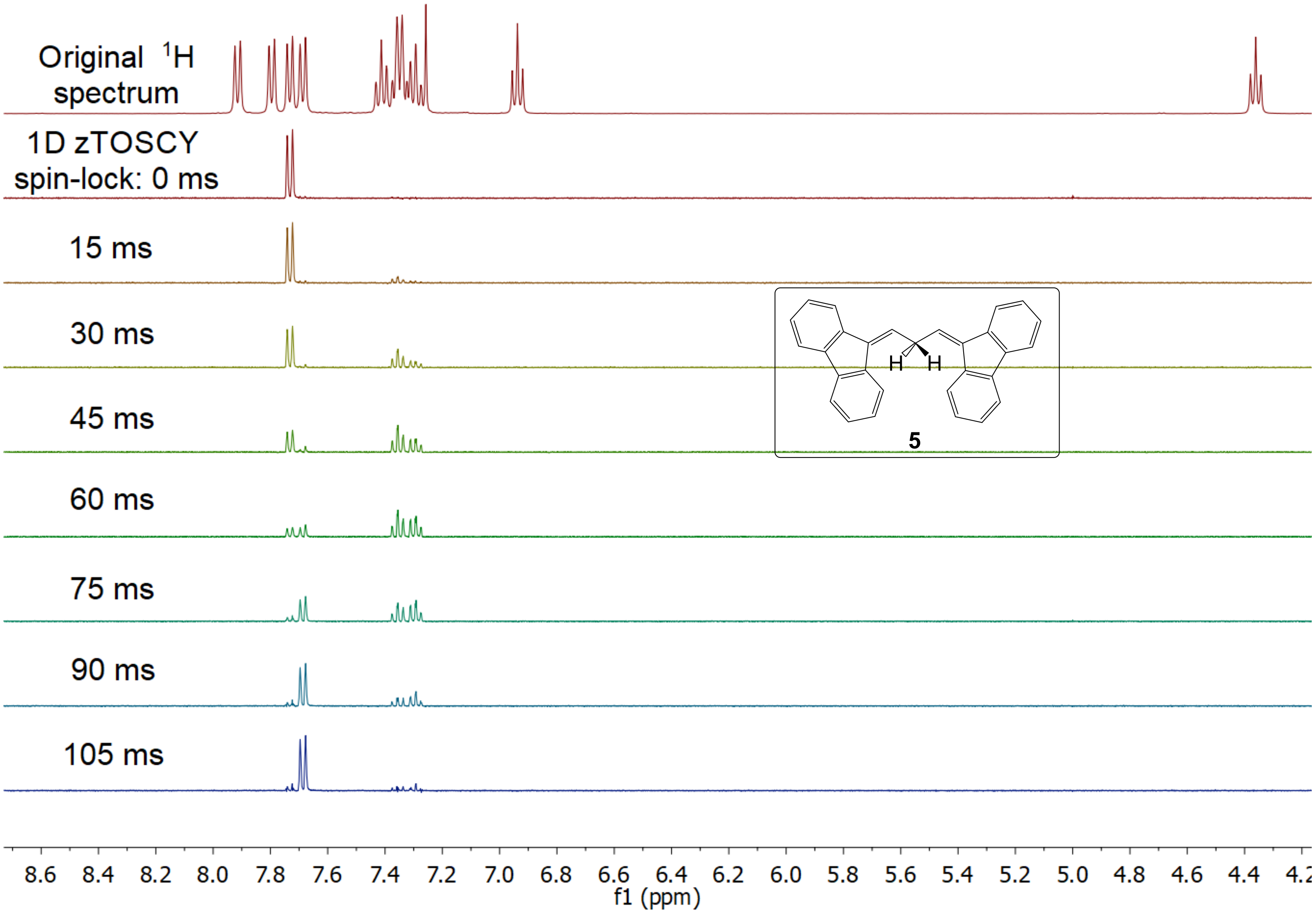

Figure S123. ^{1}H 1D zTOCSY (400 MHz) spectrum of compound **5** in chloroform-*d*.

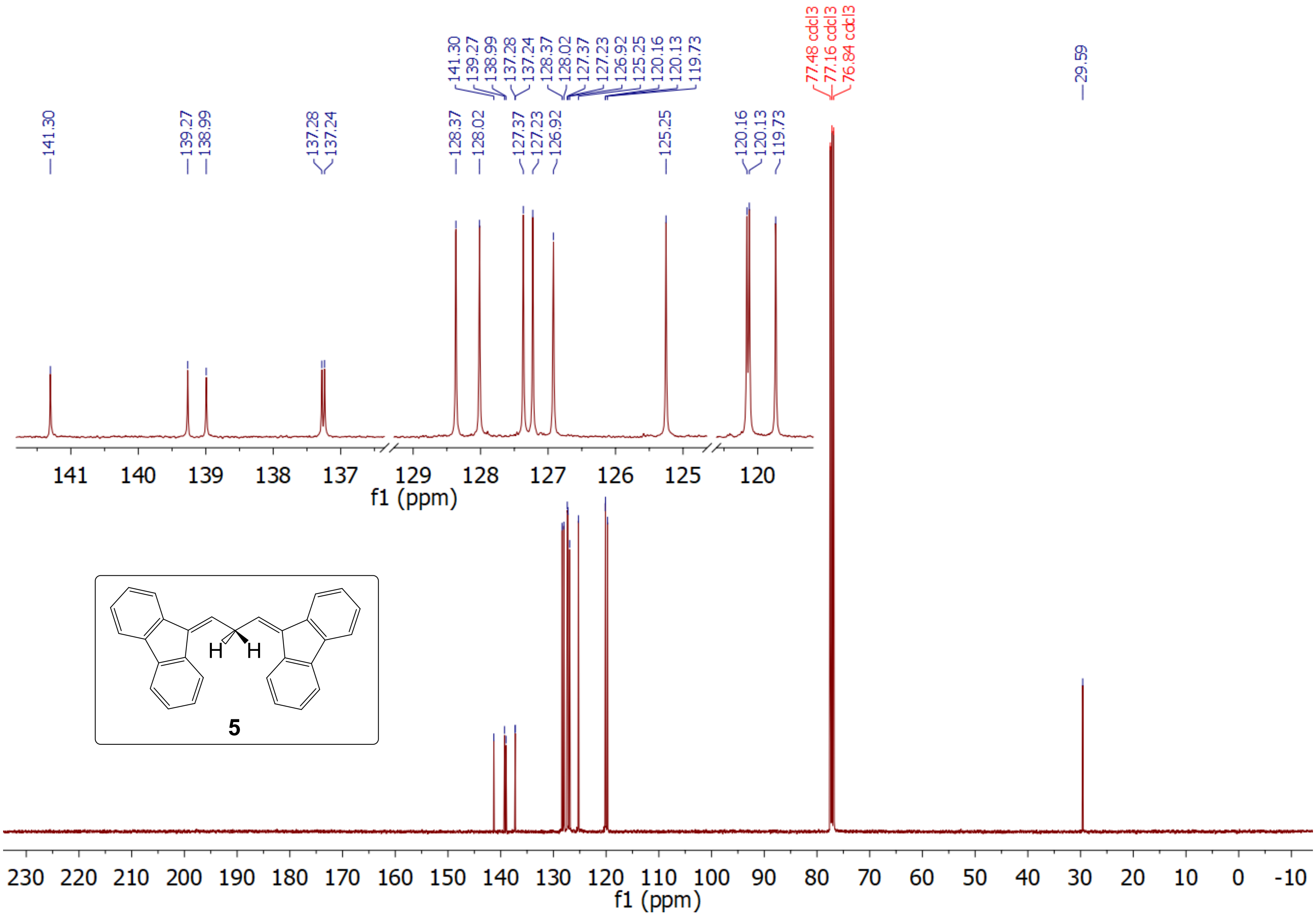

Figure S124. $^{13}C\{^1H\}$ NMR (101 MHz) spectrum of compound **5** in chloroform-*d*.

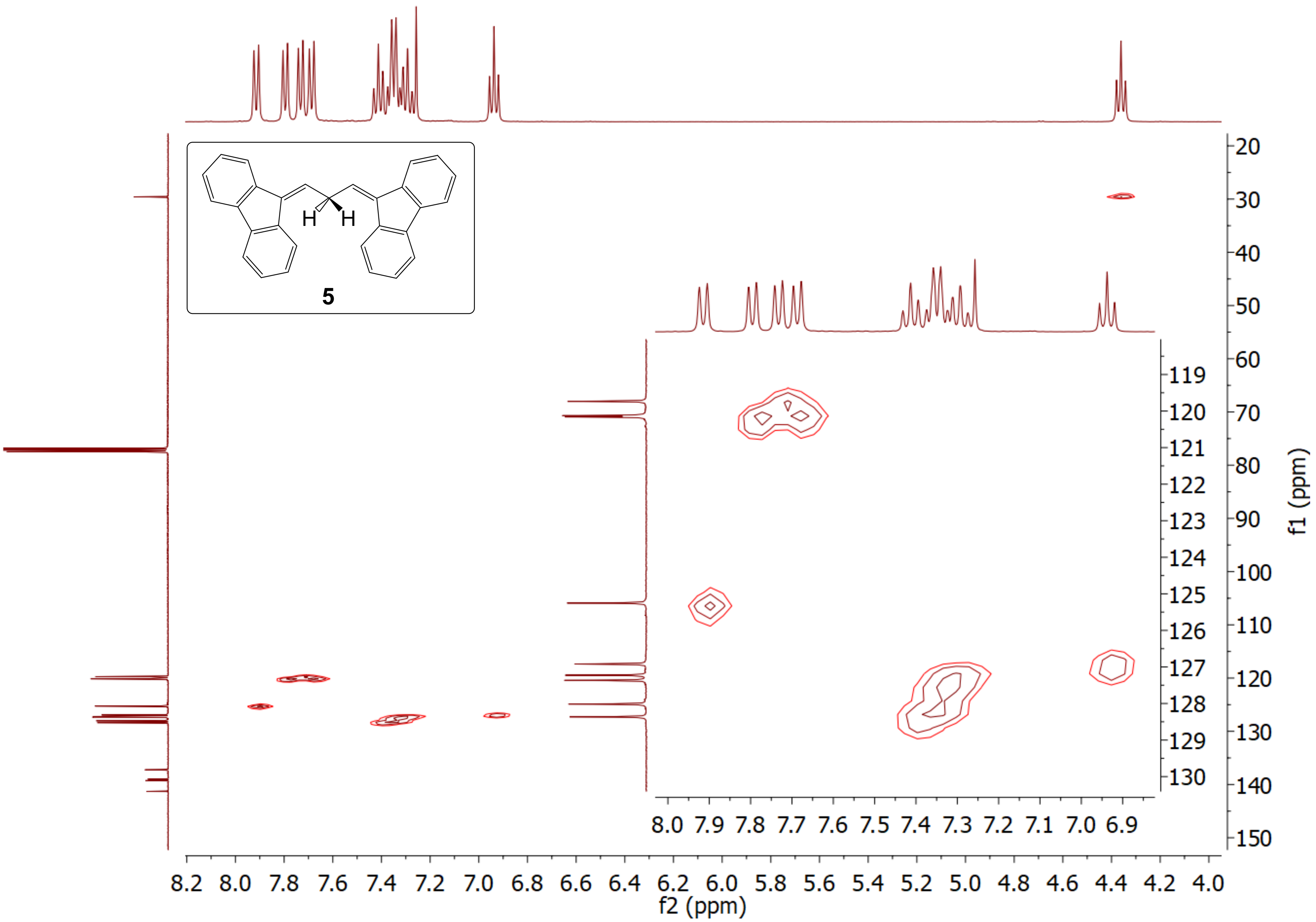

Figure S125. [1]H-^{13}C ASAPHMQC spectrum of compound **5** in chloroform-*d*.

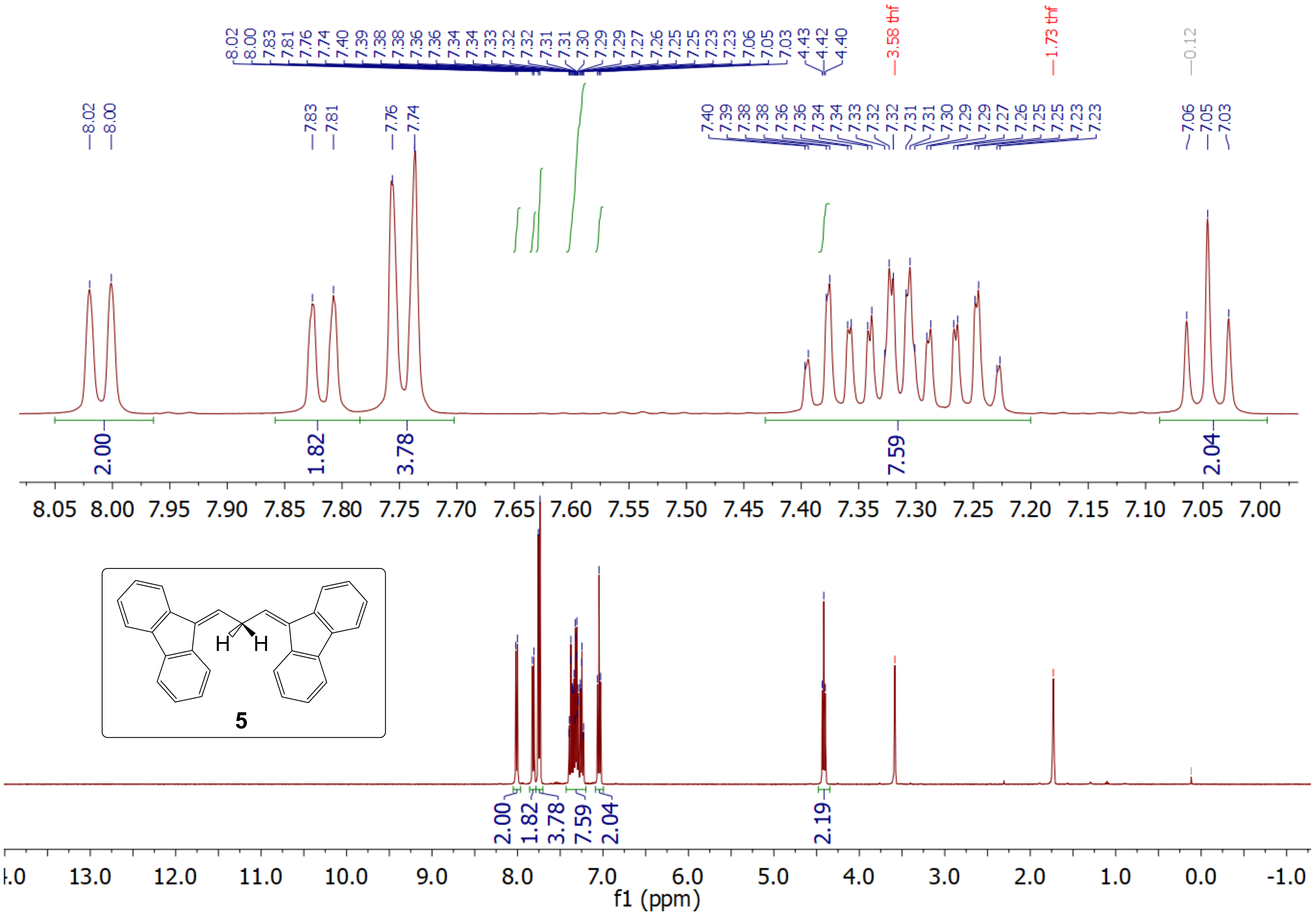

Figure S126. ^{1}H NMR (400 MHz) spectrum of compound **5** in THF-d_8. Impurities: 0.12 ppm – traces of grease.

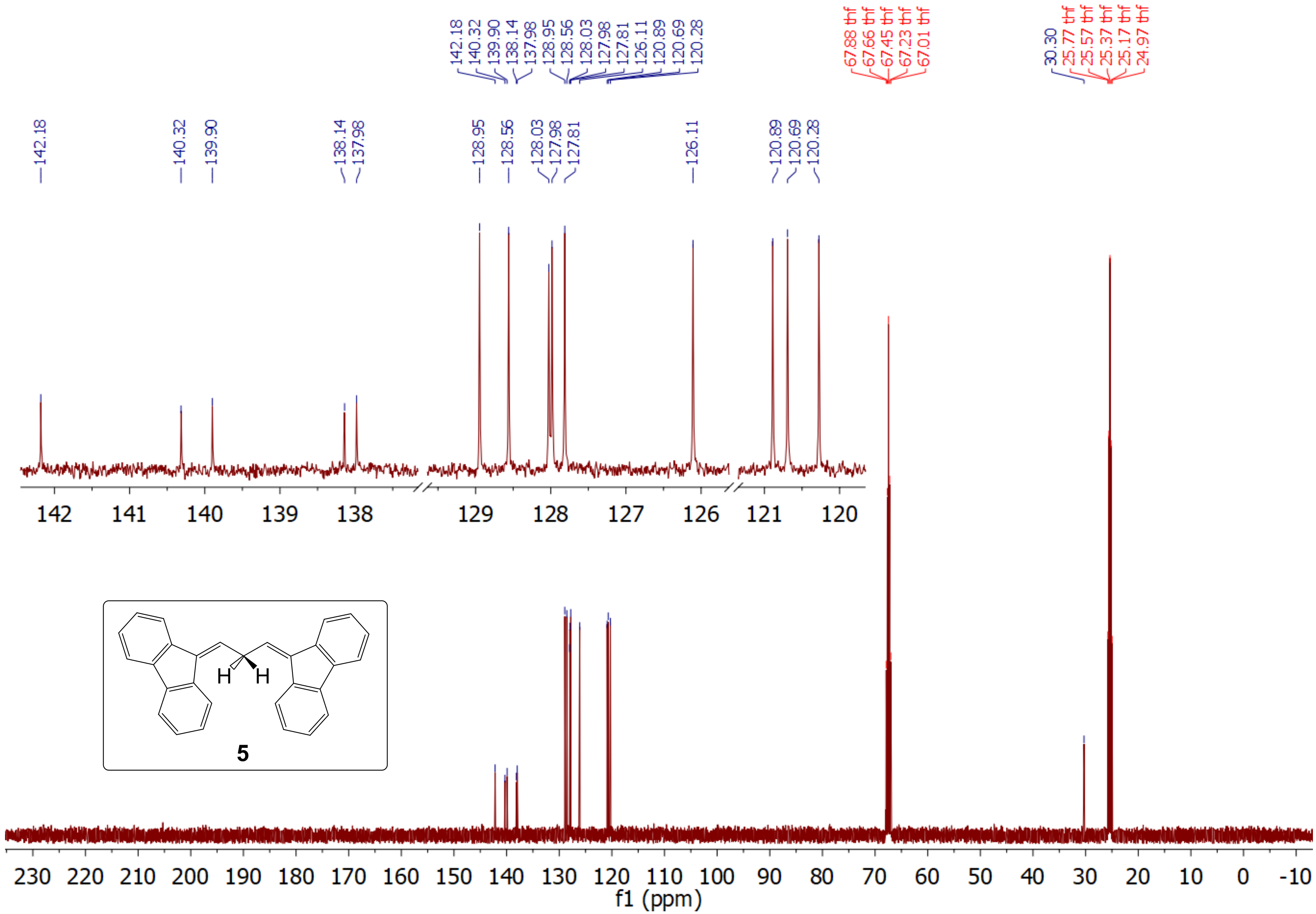

Figure S127. $^{13}C\{^1H\}$ NMR (101 MHz) spectrum of compound **5** in THF-d_8.

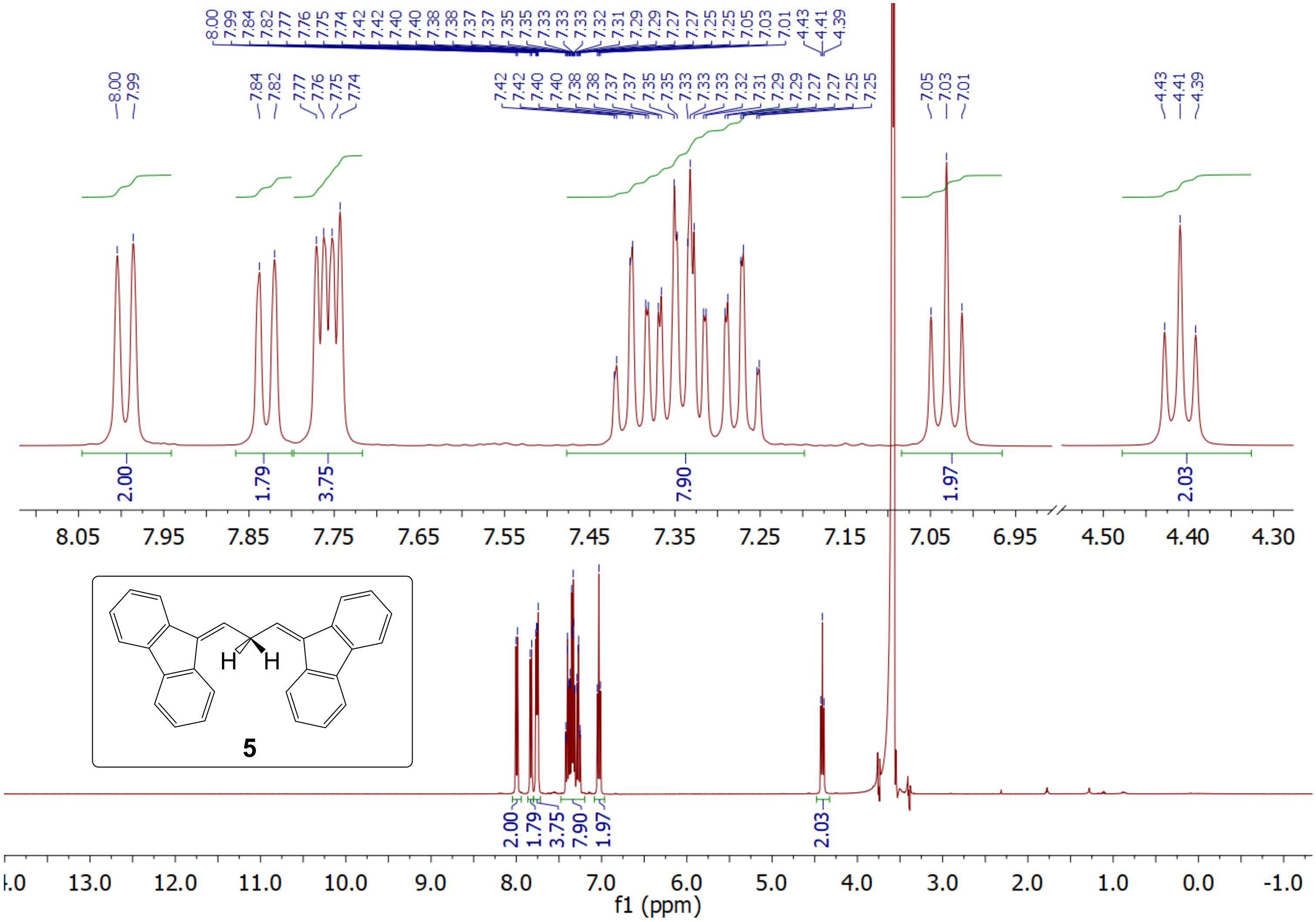

Figure S128. ^{1}H NMR (400 MHz) spectrum of compound **5** in dioxane-d_8. The spectrum was measured with suppression of 1,4-dioxane-h_8 signal. Secondary referencing was done using chemical shift of the doublet, which comes at 8.00 ppm in 1,4-dioxane-h_8 solution without solvent suppression.

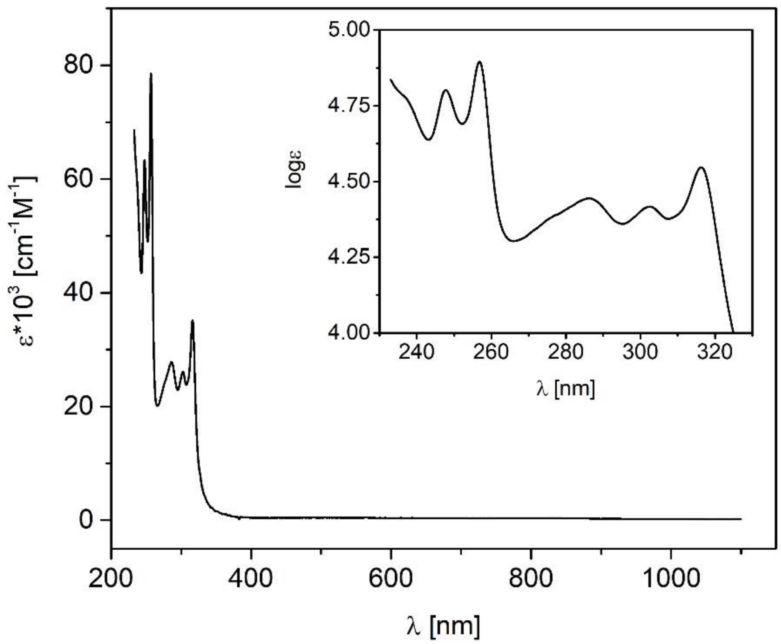

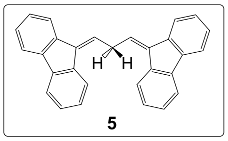

Figure S129. UV-Vis spectrum of compound **5** in diethyl ether.

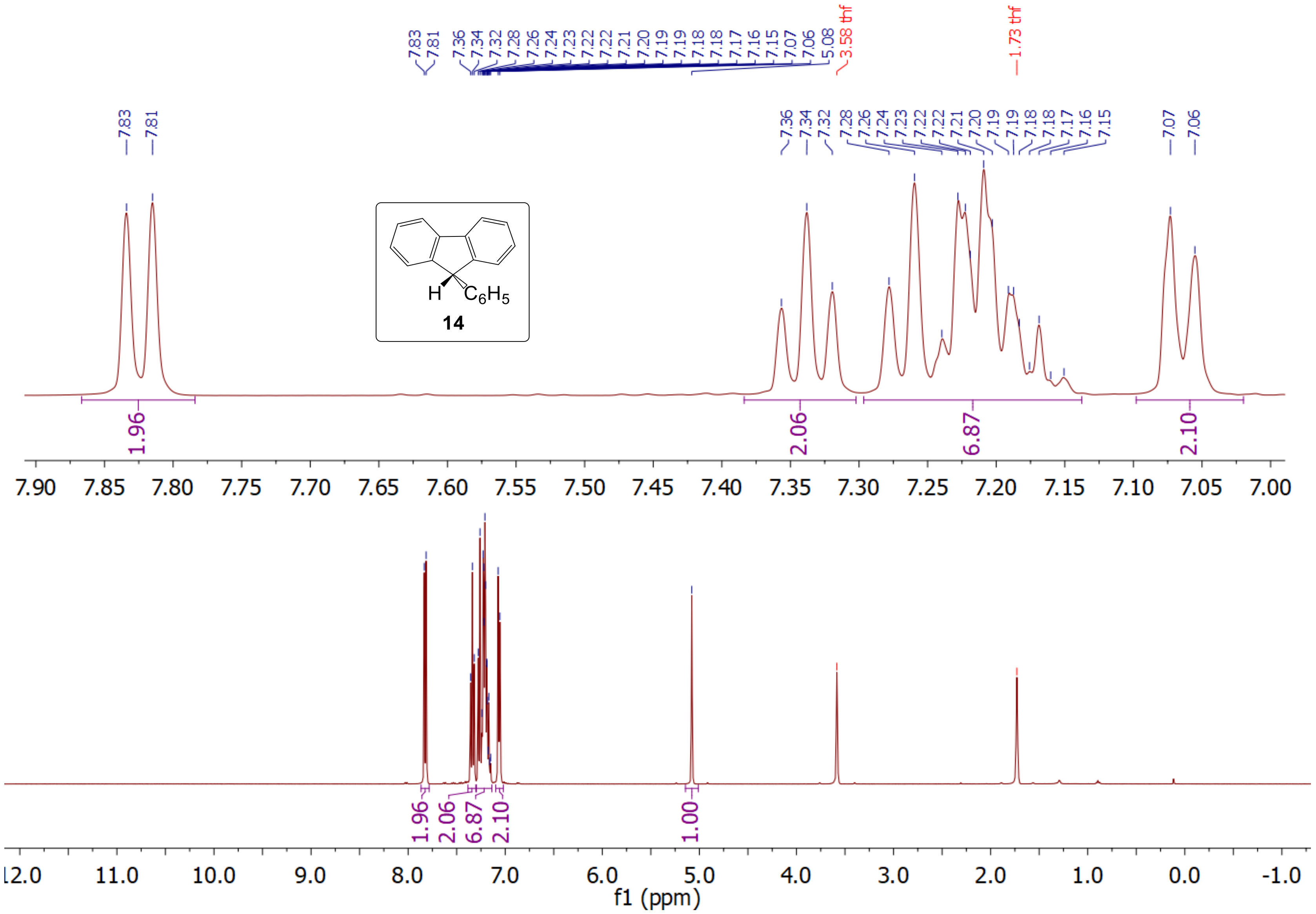

Figure S130. ^{1}H NMR (400 MHz) spectrum of compound **14** in THF-d_8.

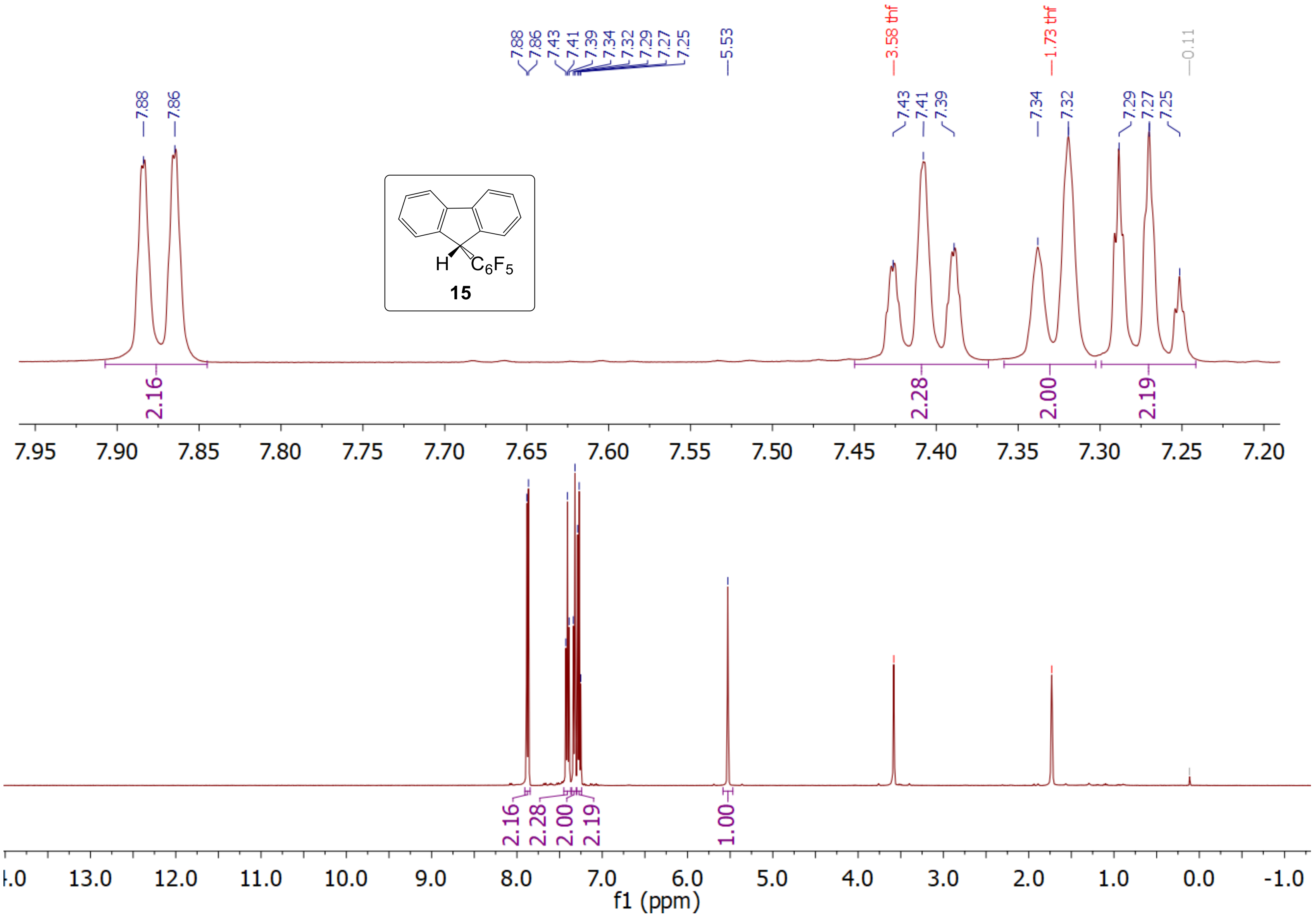

Figure S131. ^{1}H NMR (400 MHz) spectrum of compound **15** in THF-d_8. Impurities: 0.11 ppm – grease.

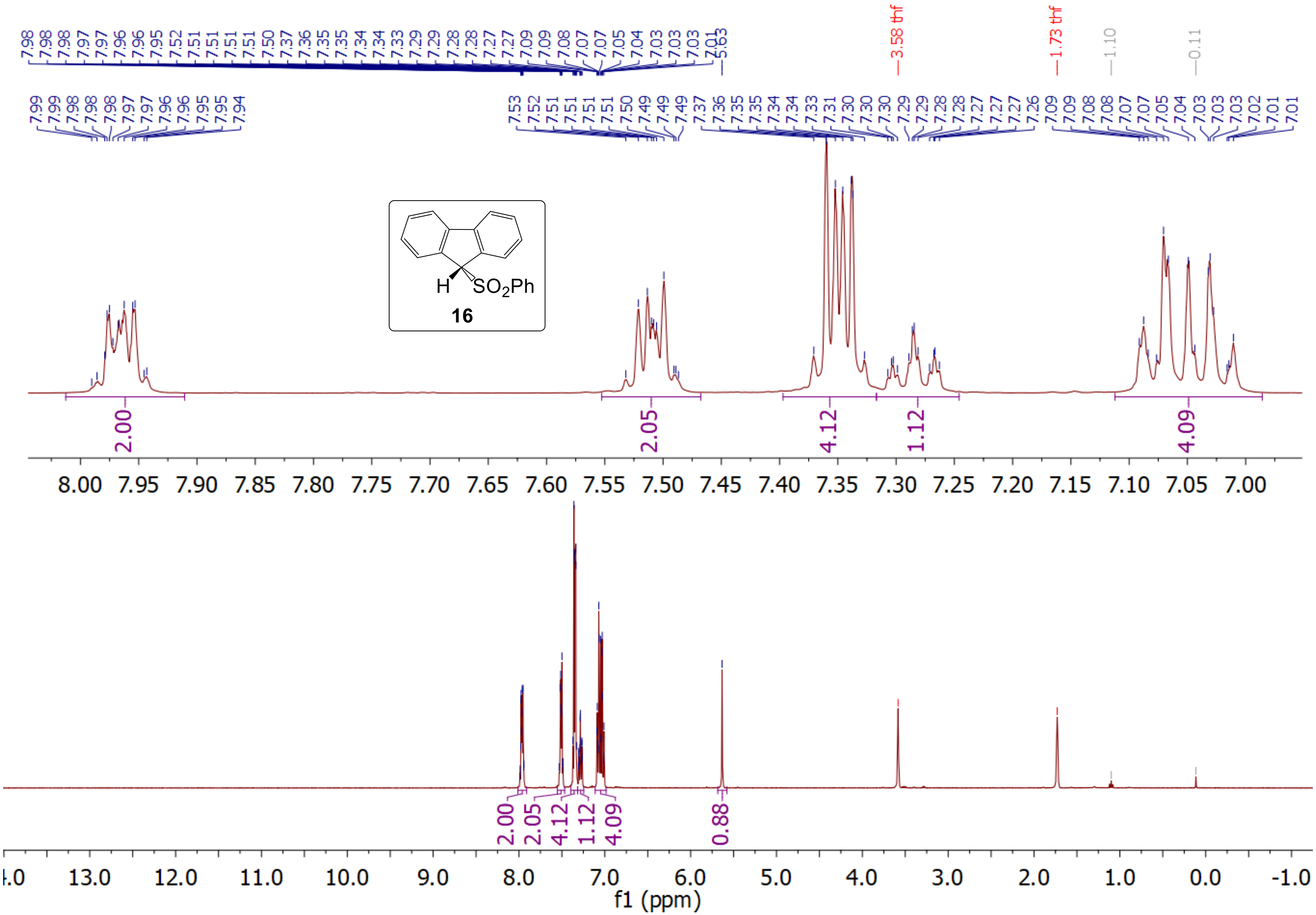

Figure S132. ^{1}H NMR (400 MHz) spectrum of compound **16** in THF-d_8. Impurities: 0.11 ppm – grease, 1.10 – ethanol.

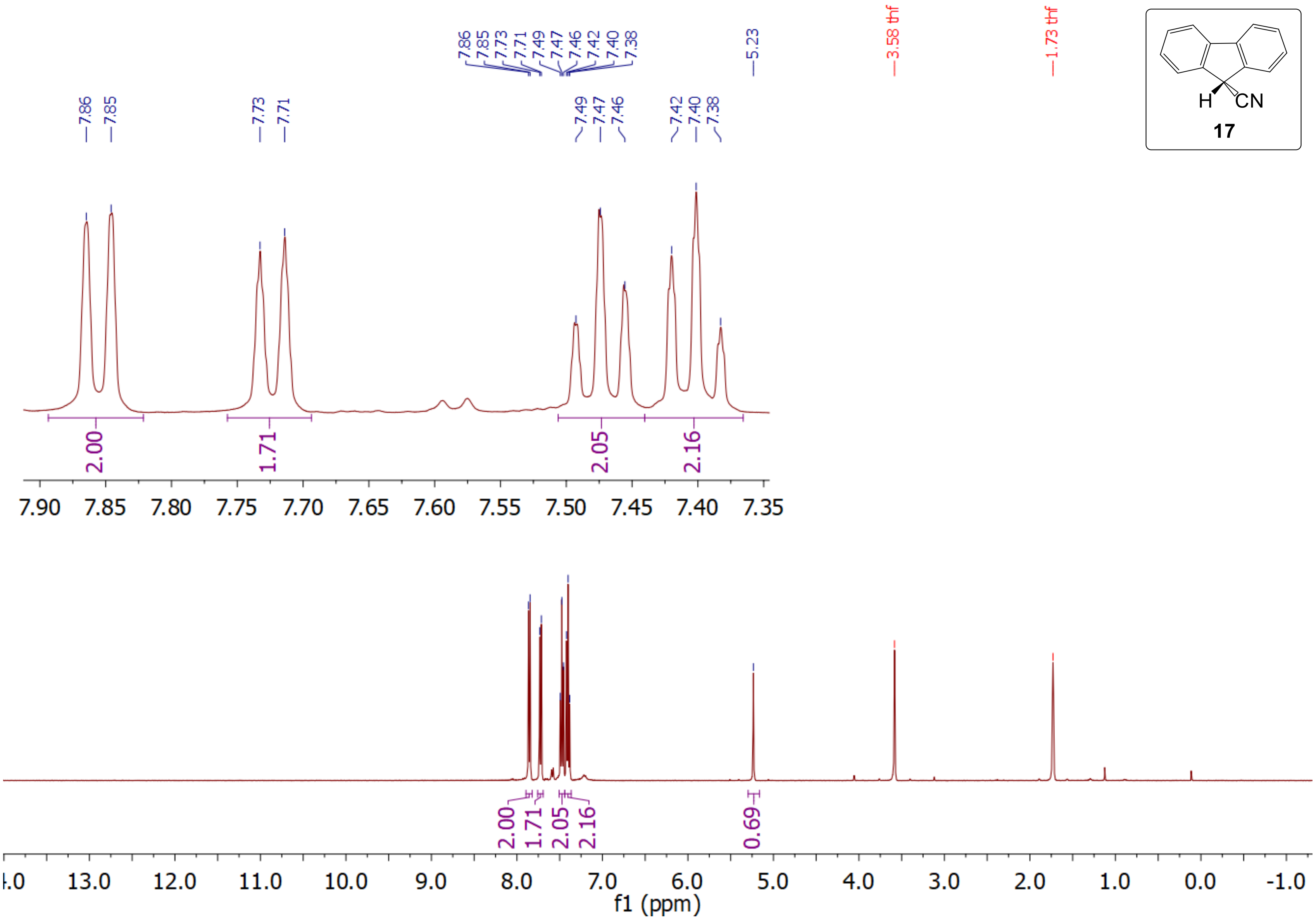

Figure S133. ^{1}H NMR (400 MHz) spectrum of compound **17** in chloroform-*d*. Minor peaks are unidentified impurities.

S8 Computational coordinates

Table S7, TSMPSi (**1**)........ S188
Table S7, $[TSMPSiH]^+$ (**2**)........ S189
Table S7, $[TSMPSiH]^+$·THF (**2·THF**)........ S191
Table S7, (TSMPSi)$Fe(CO)_4$ (**8**)........ S192
Table S7, $[TSMPSiMe]^+OTf^-$ (**9**)........ S194
Table S7, iso-TSMPSi (**10**)........ S196
Section S5.1.1, TSMPGe (**12**)........ S197
Table S8, **A**, X=P^+ (**1**)........ S188
Table S8, **B**, X=P^+ (**2**)........ S189
Table S8, **A**, X=Si........ S198
Table S8, **B**, X=Si........ S200
Table S9, **A1**........ S201
Table S9, **A1**, deprotonated........ S201
Table S9, **A2**........ S202
Table S9, **A2**, deprotonated........ S202
Table S9, **A3**........ S202
Table S9, **A3**, deprotonated........ S203
Table S9, **A4**........ S203
Table S9, **A4**, deprotonated........ S204
Table S9, **A5**........ S205
Table S9, **A5**, deprotonated........ S206
Table S9, **A6**........ S207
Table S9, **A6**, deprotonated........ S208
Table S9, **A7**........ S209
Table S9, **A7**, deprotonated........ S209
Table S9, **A8**........ S209
Table S9, **A8**, deprotonated........ S211
Table S9, **A9**........ S212
Table S9, **A9**, deprotonated........ S214
Table S9, **A10**........ S215
Table S9, **A10**, deprotonated........ S217
Table S9, **A11**........ S218
Table S9, **A11**, deprotonated........ S220
Table S9, **A12**........ S221
Table S9, **A12**, deprotonated........ S223
Table S9, $[TSMPSiH]^+$ (**2**)........ S189
Table S9, **2**, deprotonated, a.k.a. TSMPSi (**1**)........ S188
Table S10, (*N*-Sk)$_3$Si-**R**, **R**=LP........ S224
Table S10, (*N*-Sk)$_3$Si-**R**, **R**=$FeBr_2$(THF)........ S225
Table S10, (*N*-Sk)$_3$Si-**R**, **R**=Me........ S227
Table S10, (*N*-Sk)$_3$Si-**R**, **R**=H........ S229
Table S10, (2-Sk)$_3$Si-Me........ S230
Table S10, anionic R'=SiMe cage, **R**=LP........ S231
Table S10, R'=SiMe cage complex, **R**=$FeBr_2$(THF)........ S233
Table S10, R'=SiMe cage complex, **R**=Me........ S235
Table S10, R'=SiMe cage complex, **R**=H........ S221

Table S10, (2-Sk)$_3$CH…… S236
Table S10, anionic R'=CH cage, **R**=LP…… S220
Table S10, R'=CH cage complex, **R**=FeBr$_2$(THF)…… S237
Table S10, R'=CH cage complex, **R**=Me…… S239
Table S10, R'=CH cage complex, **R**=H…… S218
Table S10, skatole…… S240
Table S11, TSMPSi (**1**)…… S188
Figure S10, TSMPSi (**1**)…… S188
Table S12, **A**…… S241
Table S12, **[A-H$^+$]$^-$ (relaxed)** …… S242
Table S12, **B**…… S244
Table S12, **[B-H$^+$]$^-$ (relaxed)** …… S245
Table S12, **C**…… S246
Table S12, **[C-H$^+$]$^-$ (relaxed)** …… S248
Table S12, **2**, a.k.a. [TSMPSiH]$^+$ (**2**) …… S189
Table S12, **[2-H$^+$]^0 (relaxed)**, a.k.a. TSMPSi (**1**) …… S188
Table S15, L=**I**…… S249
Table S15, L=**II**…… S250
Table S15, L=**III**…… S251
Table S15, L=**IV**…… S253
Table S15, L=**V**…… S254
Table S15, L=**VI**…… S256
Table S15, L=**VII**…… S258
Table S15, L=**VIII**…… S259
Table S15, L=**IX**…… S260
Table S15, L=**X**…… S262
Table S15, L=**XI**…… S263
Table S15, L=**XII**…… S265
Table S15, L=**XIII**…… S266
Table S15, L=**XIV**…… S268
Table S15, L=**XV**…… S269
Table S15, L=**XVI**…… S271
Table S15, L=**XVII**…… S271
Table S15, L=**XVIII**…… S273
Table S15, L=**XIX**…… S275
Table S15, L=TSMPSi (**1**) …… S275
Table S15, L=TSMPGe (**12**) …… S277
Table S16, **A**…… S279
Table S16, **B**, *E*-form…… S280
Table S16, **B**, *Z*-form…… S282
Table S16, **C**…… S283
Table S16, **D**, *E*-form…… S285
Table S16, **D**, *Z*-form…… S287
Chart S4, **Heq1**…… S289
Chart S4, **TS1**…… S290
Chart S4, **Heq2**…… S291
Chart S5, **Heq1'**…… S292
Chart S5, **TS1'**…… S293

Chart S5, **Heq2’**... S295
Figure S22, ***Z*-form**..S296
Figure S22, **TS2**..S297
Figure S22, **Heq2**... S291
Section S5.3.8, opening into an *E*-form: **Heq2**....................... S291
Section S5.3.8, opening into an E-form: **TS**.......................... S298
Section S5.3.8, opening into an *E*-form: ***E*-form**.....................S299
Scheme S9, **Heq1**..S289
Scheme S9, **TS1**..S290
Scheme S9, **Heq2**...S291
Scheme S9, **TS2**.. S300
Scheme S9, ***Z*-form**...S296

Table S7, TSMPSi (**1**)
Level of theory: B3LYP-GD3BJ/6-311++G**
Energy: -1177906.7422050 Kcal/mol

Si	-0.00051	-0.00009	-1.55977
N	0.90016	-1.27915	-0.49976
C	1.65333	-2.34920	-0.94177
C	0.93454	-1.32760	0.89947
C	2.15611	-3.06218	0.18280
C	1.68539	-2.39314	1.36213
N	0.65722	1.41932	-0.49973
C	1.20750	2.60655	-0.94168
C	0.68240	1.47301	0.89954
C	1.57437	3.39801	0.18293
C	1.23034	2.65579	1.36224
N	-1.55837	-0.14038	-0.49939
C	-2.86176	-0.25760	-0.94103
C	-1.61717	-0.14565	0.89990
C	-3.73037	-0.33572	0.18377
C	-2.91531	-0.26247	1.36289
C	1.94253	-2.76107	-2.25016
C	2.95768	-4.20044	-0.01166
C	3.23963	-4.60215	-1.30547
C	2.73429	-3.88566	-2.41215
H	1.55726	-2.21464	-3.10175
H	3.34763	-4.75336	0.83581
H	3.85558	-5.47702	-1.47727
H	2.97267	-4.22510	-3.41362
C	2.15988	4.66107	-0.01149
C	1.41919	3.06317	-2.25005
C	2.36632	5.10639	-1.30528
C	1.99767	4.31096	-2.41201
H	2.44452	5.27487	0.83600

H	2.81634	6.07711	-1.47703
H	2.17216	4.68730	-3.41346
H	1.13803	2.45658	-3.10163
C	-3.36336	-0.30256	-2.24929
C	-4.73324	-0.42553	-2.41093
C	-5.60618	-0.50404	-1.30401
C	-5.11700	-0.46029	-0.01033
H	-2.69764	-0.24266	-3.10102
H	-5.14663	-0.46253	-3.41228
H	-6.67189	-0.59972	-1.47551
H	-5.79065	-0.52080	0.83734
C	-3.44189	-0.30953	2.76232
H	-2.65646	-0.23900	3.51353
H	-4.14391	0.51118	2.94185
H	-3.98667	-1.24200	2.94204
C	1.45388	3.13530	2.76153
H	2.51680	3.32470	2.94319
H	0.92583	4.07778	2.93881
H	1.11477	2.42360	3.51296
C	1.99131	-2.82466	2.76149
H	1.53043	-2.18495	3.51289
H	1.64045	-3.84637	2.93901
H	3.07099	-2.82083	2.94298
P	-0.00002	-0.00017	1.63038
C	0.00045	-0.00037	3.43315
H	-0.43202	-0.93386	3.78993
H	1.02529	0.09170	3.78945
H	-0.59169	0.84092	3.79001

Table S7, [TSMPSiH]$^+$ (**2**)
Level of theory: B3LYP-GD3BJ/6-311++G**
Energy: -1178168.7388729 Kcal/mol

Si	-0.00000	-0.00049	-1.28552
N	-1.36324	-0.78764	-0.50653
C	-2.50461	-1.44525	-0.96002
C	-1.40802	-0.81373	0.91643
C	-3.24703	-1.87368	0.16642
C	-2.53818	-1.46533	1.35392
N	1.36316	-0.78777	-0.50653
C	2.50447	-1.44551	-0.96002
C	1.40793	-0.81387	0.91643
C	3.24685	-1.87401	0.16642
C	2.53803	-1.46559	1.35392

N	0.00008	1.57350	-0.50623
C	0.00015	2.89090	-0.95936
C	0.00008	1.62489	0.91672
C	0.00019	3.74773	0.16733
C	0.00015	2.92934	1.35459
C	-2.93494	-1.69257	-2.26468
C	-4.45267	-2.56860	-0.02002
C	-4.88171	-2.81522	-1.31269
C	-4.12917	-2.38083	-2.42126
H	-2.36734	-1.36487	-3.12721
H	-5.03515	-2.90481	0.82927
H	-5.80849	-3.34934	-1.48004
H	-4.49171	-2.58920	-3.42040
C	4.45241	-2.56905	-0.02002
C	2.93476	-1.69287	-2.26468
C	4.88142	-2.81572	-1.31270
C	4.12893	-2.38125	-2.42126
H	5.03485	-2.90532	0.82927
H	5.80815	-3.34993	-1.48005
H	4.49145	-2.58965	-3.42040
H	2.36720	-1.36511	-3.12721
C	0.00017	3.38759	-2.26388
C	0.00024	4.76600	-2.42006
C	0.00029	5.63459	-1.31125
C	0.00026	5.13936	-0.01871
H	0.00014	2.73242	-3.12659
H	0.00026	5.18444	-3.41908
H	0.00034	6.70432	-1.47829
H	0.00029	5.81171	0.83073
C	0.00017	3.47031	2.74714
H	0.00014	2.69174	3.50768
H	0.87999	4.09824	2.91350
H	-0.87957	4.09833	2.91350
C	3.00664	-1.73637	2.74636
H	3.10885	-2.81238	2.91311
H	3.99116	-1.29000	2.91217
H	2.33329	-1.34561	3.50696
C	-3.00681	-1.73606	2.74636
H	-2.33343	-1.34538	3.50696
H	-3.99129	-1.28960	2.91217
H	-3.10913	-2.81207	2.91311
P	-0.00000	-0.00099	1.66634
C	0.00000	-0.00049	3.46449
H	-0.89180	0.51474	3.81896

H	-0.00005	-1.03006	3.81996
H	0.89185	0.51465	3.81896
H	-0.00000	-0.00031	-2.74533

Table S7, [TSMPSiH]$^+$·THF (**2·THF**)

Level of theory: B3LYP-GD3BJ/6-311++G**

Energy: -1324240.8861323 Kcal/mol

C	-2.81298	-0.07967	1.64557
C	-4.03212	-0.09107	0.91572
C	-5.25988	-0.13063	1.59764
C	-5.25029	-0.15766	2.98008
C	-4.03414	-0.14559	3.69287
C	-2.80922	-0.10687	3.04582
C	-2.33218	-0.02567	-0.54191
C	-3.71002	-0.05607	-0.48442
H	-6.19534	-0.13963	1.05075
H	-6.18443	-0.18818	3.52688
H	-4.05775	-0.16700	4.77582
H	-1.88955	-0.09782	3.61528
P	-1.09316	0.02748	-1.81258
C	-0.04800	-1.37234	-1.43041
C	0.40510	-2.48290	-2.10686
C	1.11486	-2.55572	0.09158
C	1.15451	-3.24930	-1.14427
C	1.72063	-3.07692	1.23829
C	1.83911	-4.47169	-1.23735
C	2.39060	-4.28554	1.12066
H	1.65110	-2.57571	2.19397
C	2.45752	-4.97537	-0.10589
H	1.87668	-5.01423	-2.17449
H	2.86172	-4.71706	1.99569
H	2.98780	-5.91813	-0.15499
C	-0.10864	1.45368	-1.37295
C	0.31557	2.59781	-2.01122
C	1.01968	2.61757	0.19004
C	1.04128	3.35277	-1.02166
C	1.61135	3.11484	1.35446
C	1.69116	4.59656	-1.07223
C	2.24737	4.34490	1.27880
H	1.55522	2.57828	2.29164
C	2.29483	5.07809	0.07661
H	1.71398	5.17155	-1.99029
H	2.70692	4.75887	2.16833

H	2.79866	6.03639	0.06038
Si	0.04694	-0.00391	0.99802
N	0.32539	1.43416	-0.02317
N	0.39077	-1.38430	-0.08232
N	-1.75633	-0.03948	0.74432
C	0.09913	3.05010	-3.41942
H	-0.42721	2.31700	-4.02723
H	-0.47959	3.97827	-3.44112
H	1.05598	3.25693	-3.90681
C	-4.71759	-0.05524	-1.58813
H	-5.34428	-0.95044	-1.53876
H	-5.38369	0.80808	-1.50134
H	-4.26455	-0.02396	-2.57790
C	0.19705	-2.89409	-3.52888
H	-0.36062	-3.83389	-3.58087
H	-0.34590	-2.15327	-4.11227
H	1.15779	-3.06305	-4.02314
C	-1.72302	0.04773	-3.49762
H	-0.88867	0.07757	-4.19641
H	-2.31326	-0.85259	-3.66364
H	-2.34845	0.92981	-3.62925
H	0.05916	-0.03833	2.45699
O	2.12667	0.04171	1.35534
C	2.78242	-0.04062	2.65918
C	3.12608	0.06956	0.27759
C	4.21506	-0.43834	2.33873
H	2.72696	0.94280	3.13066
H	2.23973	-0.76241	3.26772
C	4.45451	0.26195	0.99373
H	3.06508	-0.88565	-0.24390
H	2.85942	0.88234	-0.39373
H	4.29126	-1.52204	2.22523
H	4.90774	-0.12103	3.11829
H	5.28306	-0.16851	0.43129
H	4.65470	1.32530	1.14421

Table S7, (TSMPSi)Fe(CO)$_4$ (**8**)
Level of theory: B3LYP-GD3BJ/6-311++G**
Energy: -2255544.0921790 Kcal/mol

C	0.00000	2.91738	-0.05558
C	0.00000	3.75141	-1.20709
C	0.00000	5.14986	-1.07740
C	0.00000	5.69850	0.19144

C	0.00000	4.86552	1.32723
C	0.00000	3.48423	1.22520
C	0.00000	1.61846	-1.88144
C	0.00000	2.90759	-2.36662
H	0.00000	5.78372	-1.95686
H	0.00000	6.77447	0.31824
H	0.00000	5.31497	2.31330
H	0.00000	2.87085	2.11045
P	0.00000	0.00000	-2.61012
C	-1.40163	-0.80923	-1.88144
C	-2.51805	-1.45379	-2.36662
C	-2.52652	-1.45869	-0.05558
C	-3.24882	-1.87570	-1.20709
C	-3.01743	-1.74211	1.22520
C	-4.45991	-2.57493	-1.07740
C	-4.21367	-2.43276	1.32723
H	-2.48623	-1.43542	2.11045
C	-4.93505	-2.84925	0.19144
H	-5.00885	-2.89186	-1.95686
H	-4.60290	-2.65749	2.31330
H	-5.86687	-3.38724	0.31824
C	1.40163	-0.80923	-1.88144
C	2.51805	-1.45379	-2.36662
C	2.52652	-1.45869	-0.05558
C	3.24882	-1.87570	-1.20709
C	3.01743	-1.74211	1.22520
C	4.45991	-2.57493	-1.07740
C	4.21367	-2.43276	1.32723
H	2.48623	-1.43542	2.11045
C	4.93505	-2.84925	0.19144
H	5.00885	-2.89186	-1.95686
H	4.60290	-2.65749	2.31330
H	5.86687	-3.38724	0.31824
N	1.37210	-0.79218	-0.47119
N	0.00000	1.58436	-0.47119
C	2.95917	-1.70848	-3.77257
H	2.27182	-1.31164	-4.51699
H	3.94001	-1.25877	-3.95554
H	3.06013	-2.78276	-3.95554
C	0.00000	3.41695	-3.77257
H	-0.87988	4.04153	-3.95554
H	0.87988	4.04153	-3.95554
H	0.00000	2.62327	-4.51699
C	-2.95917	-1.70848	-3.77257

H	-3.94001	-1.25877	-3.95554
H	-2.27182	-1.31164	-4.51699
H	-3.06013	-2.78276	-3.95554
C	0.00000	0.00000	-4.41151
H	0.00000	-1.02907	-4.76671
H	-0.89120	0.51454	-4.76671
H	0.89120	0.51454	-4.76671
N	-1.37210	-0.79218	-0.47119
Fe	0.00000	0.00000	2.68762
Si	0.00000	0.00000	0.45738
C	1.54317	0.89095	2.59017
O	2.54015	1.46656	2.54105
C	0.00000	0.00000	4.48502
C	-1.54317	0.89095	2.59017
C	-0.00000	-1.78190	2.59017
O	-0.00000	-2.93311	2.54105
O	-2.54015	1.46656	2.54105
O	0.00000	0.00000	5.63103

Table S7, [TSMPSiMe]$^+$OTf$^-$ (**9**)
Level of theory: B3LYP-GD3BJ/6-311++G**
Energy: -1202865.3240748 Kcal/mol

C	0.85640	2.89754	-0.00000
C	-0.27597	3.74896	-0.00000
C	-0.10432	5.14208	-0.00000
C	1.18222	5.65191	-0.00000
C	2.29770	4.79300	-0.00000
C	2.15511	3.41302	-0.00000
C	-1.01038	1.62427	-0.00000
C	-1.45787	2.92491	-0.00000
H	-0.96155	5.80458	-0.00000
H	1.33863	6.72324	-0.00000
H	3.29333	5.21969	-0.00000
H	3.02754	2.77405	-0.00000
P	-1.75818	0.00055	0.00000
C	-1.01070	-0.81161	1.40624
C	-1.45825	-1.46227	2.53239
C	0.85601	-1.44883	2.50869
C	-0.27641	-1.87479	3.24586
C	2.15470	-1.70682	2.95496
C	-0.10476	-2.57194	4.45199
C	2.29729	-2.39737	4.14975
H	3.02711	-1.38710	2.40169

C	1.18178	-2.82712	4.89338
H	-0.96202	-2.90343	5.02554
H	3.29290	-2.61093	4.51918
H	1.33816	-3.36323	5.82093
C	-1.01070	-0.81161	-1.40624
C	-1.45825	-1.46227	-2.53239
C	0.85601	-1.44883	-2.50869
C	-0.27641	-1.87479	-3.24586
C	2.15470	-1.70682	-2.95496
C	-0.10476	-2.57194	-4.45199
C	2.29729	-2.39737	-4.14975
H	3.02711	-1.38710	-2.40169
C	1.18178	-2.82712	-4.89338
H	-0.96202	-2.90343	-5.02554
H	3.29290	-2.61093	-4.51918
H	1.33816	-3.36323	-5.82093
Si	1.21108	0.00029	-0.00000
N	0.41094	-0.78769	-1.36461
N	0.41123	1.57618	-0.00000
C	-2.85337	-1.73008	-2.99534
H	-3.61080	-1.33497	-2.32121
H	-3.02119	-1.28629	-3.98074
H	-3.02404	-2.80590	-3.09362
C	-2.85285	3.46015	-0.00000
H	-3.02182	4.08750	0.87979
H	-3.02182	4.08750	-0.87979
H	-3.61065	2.67912	-0.00000
C	-2.85337	-1.73008	2.99534
H	-3.02119	-1.28629	3.98074
H	-3.61080	-1.33497	2.32121
H	-3.02404	-2.80590	3.09362
C	-3.55659	0.00167	0.00000
H	-3.91260	-1.02765	0.00000
H	-3.91094	0.51696	0.89173
H	-3.91094	0.51696	-0.89173
C	3.04648	0.00085	-0.00000
H	3.42667	0.51283	-0.88621
H	3.42667	0.51283	0.88621
H	3.42785	-1.02215	0.00000
N	0.41094	-0.78769	1.36461

Table S7, iso-TSMPSi (**10**)
Level of theory: B3LYP-GD3BJ/6-311++G**
Energy: -1177947.6188935 Kcal/mol

C	0.00000	2.90101	-0.74084
C	0.00000	3.73592	0.40845
C	0.00000	5.12828	0.25219
C	0.00000	5.66137	-1.02912
C	0.00000	4.82194	-2.15699
C	0.00000	3.43790	-2.02996
C	0.00000	1.58824	1.10704
C	0.00000	2.87963	1.57020
H	0.00000	5.77786	1.12020
H	0.00000	6.73607	-1.16698
H	0.00000	5.26214	-3.14734
H	0.00000	2.80755	-2.90982
P	0.00000	0.00000	2.02792
C	1.37546	-0.79412	1.10704
C	2.49383	-1.43981	1.57020
C	2.51235	-1.45050	-0.74084
C	3.23540	-1.86796	0.40845
C	2.97730	-1.71895	-2.02996
C	4.44122	-2.56414	0.25219
C	4.17593	-2.41097	-2.15699
H	2.43141	-1.40378	-2.90982
C	4.90289	-2.83068	-1.02912
H	5.00378	-2.88893	1.12020
H	4.55715	-2.63107	-3.14734
H	5.83361	-3.36804	-1.16698
C	-1.37546	-0.79412	1.10704
C	-2.49383	-1.43981	1.57020
C	-2.51235	-1.45050	-0.74084
C	-3.23540	-1.86796	0.40845
C	-2.97730	-1.71895	-2.02996
C	-4.44122	-2.56414	0.25219
C	-4.17593	-2.41097	-2.15699
H	-2.43141	-1.40378	-2.90982
C	-4.90289	-2.83068	-1.02912
H	-5.00378	-2.88893	1.12020
H	-4.55715	-2.63107	-3.14734
H	-5.83361	-3.36804	-1.16698
Si	0.00000	0.00000	-1.08003
N	-1.36751	-0.78953	-0.31112
N	0.00000	1.57906	-0.31112
C	-2.89223	-1.66983	2.99208

H	-2.15931	-1.24668	3.68071
H	-3.86333	-1.21272	3.20787
H	-2.98191	-2.73938	3.20787
C	0.00000	3.33966	2.99208
H	0.88141	3.95210	3.20787
H	-0.88141	3.95210	3.20787
H	0.00000	2.49336	3.68071
C	2.89223	-1.66983	2.99208
H	3.86333	-1.21272	3.20787
H	2.15931	-1.24668	3.68071
H	2.98191	-2.73938	3.20787
C	0.00000	0.00000	-2.92382
H	-0.88617	0.51163	-3.30468
H	0.88617	0.51163	-3.30468
H	-0.00000	-1.02326	-3.30468
N	1.36751	-0.78953	-0.31112

Section S5.1.1, TSMPGe (**12**)
Level of theory: B3LYP-GD3BJ/6-311++G**
Energy: -2299578.0183706 Kcal/mol

C	-0.30850	2.92629	-0.82234
C	-0.39605	3.76109	0.32840
C	-0.54302	5.15089	0.17402
C	-0.59936	5.67670	-1.10459
C	-0.51133	4.83809	-2.23760
C	-0.36626	3.46666	-2.11555
C	-0.17041	1.62806	0.97530
C	-0.30591	2.91248	1.48083
H	-0.61118	5.79918	1.04069
H	-0.71247	6.74526	-1.24468
H	-0.55865	5.28023	-3.22613
H	-0.29954	2.83133	-2.99048
P	0.00040	-0.00072	1.67876
C	1.49649	-0.66723	0.97560
C	2.67708	-1.19078	1.48128
C	2.69008	-1.19677	-0.82190
C	3.45723	-1.53705	0.32895
C	3.18687	-1.41750	-2.11504
C	4.73468	-2.10389	0.17474
C	4.44747	-1.97677	-2.23691
H	2.60297	-1.15870	-2.99008
C	5.21817	-2.31854	-1.10379
H	5.33051	-2.36805	1.04150

H	4.85397	-2.15730	-3.22538
H	6.20039	-2.75428	-1.24377
C	-1.32496	-0.96312	0.97577
C	-2.37035	-1.72136	1.48161
C	-2.38171	-1.73055	-0.82155
C	-3.06111	-2.22296	0.32940
C	-2.82181	-2.04995	-2.11463
C	-4.19249	-3.04345	0.17537
C	-3.93825	-2.85957	-2.23633
H	-2.30477	-1.67505	-2.98968
C	-4.62074	-3.35427	-1.10310
H	-4.72017	-3.42584	1.04220
H	-4.29836	-3.12087	-3.22475
H	-5.49064	-3.98512	-1.24291
Ge	0.00038	-0.00130	-1.60317
N	-1.31652	-0.95777	-0.41949
N	1.48717	-0.66316	-0.41965
N	-0.16995	1.61772	-0.41994
C	-2.77172	-2.01124	2.89378
H	-2.12125	-1.54026	3.62831
H	-3.79156	-1.66290	3.08712
H	-2.75984	-3.08876	3.08771
C	-0.35759	3.40512	2.89292
H	0.44838	4.12109	3.08409
H	-1.30070	3.92569	3.08889
H	-0.26622	2.60736	3.62758
C	3.13006	-1.39076	2.89344
H	4.04903	-0.82899	3.09041
H	2.39117	-1.07618	3.62801
H	3.35329	-2.44558	3.08372
C	0.00038	-0.00056	3.48256
H	0.10581	-1.02371	3.83909
H	0.83373	0.60224	3.83918
H	-0.93849	0.41968	3.83892

Table S8, **A**, X=Si
Level of theory: B3LYP-GD3BJ/6-311++G**
Energy: -1145449.3909182 Kcal/mol

Si	0.00000	-0.00044	-1.45335
N	-1.37096	-0.79295	-0.43864
C	-2.48642	-1.43649	-0.93753
C	-1.45122	-0.83951	0.96427
C	-3.28078	-1.89519	0.15550
C	-2.59936	-1.50212	1.35624

N	1.37165	-0.79177	-0.43864
C	2.48766	-1.43435	-0.93753
C	1.45195	-0.83826	0.96426
C	3.28242	-1.89236	0.15550
C	2.60065	-1.49988	1.35624
N	-0.00068	1.58288	-0.43822
C	-0.00124	2.87080	-0.93672
C	-0.00072	1.67526	0.96469
C	-0.00163	3.78780	0.15655
C	-0.00130	3.00077	1.35707
C	-2.88310	-1.66498	-2.26111
C	-4.47677	-2.58519	-0.09612
C	-4.86483	-2.80855	-1.41065
C	-4.07116	-2.35030	-2.48164
H	-2.27386	-1.31330	-3.08467
H	-5.09060	-2.93968	0.72634
H	-5.78706	-3.34049	-1.61993
H	-4.39502	-2.53676	-3.50031
C	4.47900	-2.58133	-0.09612
C	2.88452	-1.66251	-2.26111
C	4.86725	-2.80436	-1.41065
C	4.07318	-2.34680	-2.48165
H	5.09314	-2.93530	0.72633
H	5.78993	-3.33552	-1.61993
H	4.39720	-2.53300	-3.50032
H	2.27498	-1.31136	-3.08467
C	-0.00143	3.32887	-2.26018
C	-0.00201	4.70047	-2.48040
C	-0.00242	5.61667	-1.40919
C	-0.00223	5.16862	-0.09477
H	-0.00112	2.62556	-3.08386
H	-0.00217	5.07442	-3.49898
H	-0.00287	6.68137	-1.61820
H	-0.00254	5.87730	0.72783
C	-0.00154	3.55468	2.74836
H	-0.00120	2.76138	3.49711
H	0.87908	4.18282	2.93100
H	-0.88271	4.18206	2.93100
C	3.08088	-1.77674	2.74733
H	3.18465	-2.85347	2.92984
H	4.06483	-1.32728	2.92970
H	2.39386	-1.38046	3.49628
C	-3.07934	-1.77940	2.74734
H	-2.39267	-1.38253	3.49629

H	-4.06367	-1.33079	2.92970
H	-3.18219	-2.85622	2.92984
C	0.00000	-0.00105	3.66550
H	-0.88558	0.50975	4.05099
H	0.00044	-1.02334	4.05109
H	0.88515	0.51051	4.05099
Si	0.00000	-0.00102	1.79009

Table S8, **B**, X=Si
Level of theory: B3LYP-GD3BJ/6-311++G**
Energy: -1145787.1183781 Kcal/mol

Si	0.00000	-0.00076	-1.17326
N	-1.37925	-0.79718	-0.44322
C	-2.49885	-1.44205	-0.95521
C	-1.45349	-0.84018	0.98231
C	-3.28083	-1.89285	0.13783
C	-2.60033	-1.50100	1.34928
N	1.37925	-0.79716	-0.44322
C	2.49887	-1.44202	-0.95521
C	1.45350	-0.84016	0.98231
C	3.28085	-1.89281	0.13783
C	2.60035	-1.50096	1.34928
N	-0.00001	1.59167	-0.44271
C	-0.00002	2.88381	-0.95441
C	-0.00001	1.67714	0.98281
C	-0.00002	3.78622	0.13879
C	-0.00002	3.00068	1.35007
C	-2.88652	-1.66494	-2.27736
C	-4.47640	-2.58148	-0.10918
C	-4.86451	-2.80449	-1.42308
C	-4.07594	-2.34991	-2.49527
H	-2.28662	-1.31916	-3.11110
H	-5.08839	-2.93432	0.71336
H	-5.78616	-3.33525	-1.63046
H	-4.40159	-2.53709	-3.51181
C	4.47644	-2.58142	-0.10917
C	2.88655	-1.66490	-2.27736
C	4.86455	-2.80443	-1.42308
C	4.07597	-2.34986	-2.49527
H	5.08843	-2.93425	0.71336
H	5.78620	-3.33517	-1.63046
H	4.40163	-2.53703	-3.51181
H	2.28664	-1.31913	-3.11110

C	-0.00002	3.33114	-2.27649
C	-0.00003	4.70371	-2.49423
C	-0.00004	5.61376	-1.42190
C	-0.00003	5.16597	-0.10805
H	-0.00002	2.63877	-3.11028
H	-0.00004	5.07948	-3.51071
H	-0.00005	6.67734	-1.62911
H	-0.00004	5.87232	0.71453
C	-0.00002	3.57125	2.73310
H	-0.00001	2.79056	3.49387
H	0.88060	4.20038	2.89996
H	-0.88065	4.20037	2.89996
C	3.09502	-1.78605	2.73213
H	3.20093	-2.86318	2.89862
H	4.07964	-1.33679	2.89896
H	2.41852	-1.39680	3.49311
C	-3.09499	-1.78610	2.73213
H	-2.41850	-1.39683	3.49311
H	-4.07962	-1.33685	2.89896
H	-3.20089	-2.86323	2.89862
C	-0.00000	-0.00159	3.69734
H	-0.88520	0.50938	4.08184
H	0.00001	-1.02378	4.08158
H	0.88520	0.50939	4.08184
H	0.00000	-0.00042	-2.63770
Si	-0.00000	-0.00131	1.83449

Table S9, **A1**
Level of theory: B3LYP-GD3BJ/6-311++G**
Energy: -183180.9633241 Kcal/mol

Si	0.00000	0.00000	0.00000
H	0.85669	0.85669	0.85669
H	-0.85669	-0.85669	0.85669
H	-0.85669	0.85669	-0.85669
H	0.85669	-0.85669	-0.85669

Table S9, **A1**, deprotonated
Level of theory: B3LYP-GD3BJ/6-311++G**
Energy: -182802.6855340 Kcal/mol

Si	0.00000	-0.00000	0.14305
H	0.00000	1.31663	-0.66757
H	1.14024	-0.65832	-0.66757
H	-1.14024	-0.65832	-0.66757

Table S9, **A2**
Level of theory: B3LYP-GD3BJ/6-311++G**
Energy: -1048538.5901708 Kcal/mol

Si	0.00000	0.00000	0.50486
Cl	0.00000	1.93309	-0.17722
Cl	1.67411	-0.96655	-0.17722
Cl	-1.67411	-0.96655	-0.17722
H	0.00000	0.00000	1.97024

Table S9, **A2**, deprotonated
Level of theory: B3LYP-GD3BJ/6-311++G**
Energy: -1048205.3774282 Kcal/mol

Si	0.00000	0.00000	0.85921
Cl	-0.00000	1.95528	-0.23586
Cl	1.69332	-0.97764	-0.23586
Cl	-1.69332	-0.97764	-0.23586

Table S9, **A3**
Level of theory: B3LYP-GD3BJ/6-311++G**
Energy: -3326578.0355442 Kcal/mol

Si	-0.00007	-0.00001	-0.95913
H	-0.00018	-0.00012	-2.44104
Si	-1.55880	-1.52108	-0.12050
Si	-0.53802	2.11041	-0.12051
Si	2.09680	-0.58932	-0.12106
Cl	-2.04953	2.95853	-1.22739
Cl	1.11467	3.33441	-0.19110
Cl	3.58657	0.29667	-1.22776
Cl	2.33088	-2.63249	-0.19305
Cl	-1.53674	-3.25449	-1.22683
Cl	-3.44526	-0.70213	-0.19229
Cl	-1.15571	1.92355	1.82997
Cl	-1.08905	-1.96192	1.83033
Cl	2.24423	0.03787	1.82975

Table S9, **A3**, deprotonated
Level of theory: B3LYP-GD3BJ/6-311++G**
Energy: -3326271.9124920 Kcal/mol

Si	-0.00013	0.00004	-1.38877
Si	0.20513	-1.99199	-0.16378
Si	-1.82784	0.81840	-0.16384
Si	1.62261	1.17372	-0.16401
Cl	-3.39132	-0.58169	-0.20943
Cl	-2.60256	2.52307	-1.11725
Cl	1.19232	3.22785	-0.20919
Cl	3.48599	0.99204	-1.11793
Cl	2.19961	-2.64531	-0.20793
Cl	-0.88253	-3.51558	-1.11812
Cl	-1.67513	1.39142	1.84307
Cl	-0.36903	-2.14657	1.84264
Cl	2.04283	0.75463	1.84271

Table S9, **A4**
Level of theory: B3LYP-GD3BJ/6-311++G**
Energy: -952697.9548086 Kcal/mol

Si	0.00073	-0.00044	-0.80306
H	0.00073	-0.00102	-2.30161
Si	-1.47744	-1.66297	-0.04580
Si	-0.70207	2.11043	-0.04566
Si	2.18009	-0.44723	-0.04500
C	-2.57748	2.29460	-0.21782
H	-3.10699	1.55596	0.38985
H	-2.89941	3.28891	0.10899
H	-2.89585	2.16295	-1.25564
C	0.14446	3.48068	-1.03562
H	-0.16208	4.46729	-0.67244
H	1.23254	3.41475	-0.95471
H	-0.11546	3.41656	-2.09583
C	-0.24108	2.29391	1.78079
H	-0.60069	3.25044	2.17384
H	-0.67953	1.49679	2.38658
H	0.84260	2.26299	1.92168
C	-3.08629	-1.61210	-1.03741
H	-3.78860	-2.37078	-0.67622
H	-3.57220	-0.63649	-0.95518
H	-2.90004	-1.80340	-2.09780
C	-0.70068	-3.37964	-0.21927
H	0.20286	-3.47046	0.38946
H	-1.40173	-4.15577	0.10520

H	-0.42632	-3.58785	-1.25710
C	-1.86960	-1.35692	1.78048
H	-2.38287	-0.40206	1.92226
H	-2.52092	-2.14583	2.17058
H	-0.96156	-1.34136	2.38870
C	2.93925	-1.87179	-1.02939
H	3.94761	-2.09880	-0.66757
H	2.33740	-2.78003	-0.94208
H	3.01136	-1.62059	-2.09121
C	3.27967	1.08174	-0.22784
H	3.32168	1.41794	-1.26753
H	2.90882	1.91357	0.37701
H	4.30250	0.86278	0.09619
C	2.10990	-0.92993	1.78368
H	1.53572	-1.84877	1.92975
H	3.11797	-1.10179	2.17482
H	1.64540	-0.14624	2.38760

Table S9, **A4**, deprotonated
Level of theory: B3LYP-GD3BJ/6-311++G**
Energy: -952338.5800416 Kcal/mol

Si	-0.00002	0.00064	-1.21136
Si	-1.78745	1.02313	-0.07541
Si	1.77953	1.03613	-0.07554
Si	0.00763	-2.05896	-0.07566
C	1.65097	2.94170	-0.17543
H	0.79969	3.31255	0.40299
H	2.55811	3.42054	0.21372
H	1.51082	3.26427	-1.21173
C	3.44236	0.60794	-0.91627
H	4.28438	1.11371	-0.42680
H	3.62742	-0.47020	-0.88464
H	3.42433	0.90603	-1.96901
C	2.09135	0.66948	1.77752
H	2.94144	1.25444	2.15257
H	1.21491	0.91280	2.38272
H	2.31798	-0.38902	1.93469
C	-2.24884	2.67685	-0.91641
H	-3.10845	3.15264	-0.42746
H	-1.40816	3.37671	-0.88465
H	-2.49732	2.51167	-1.96924
C	-3.37256	-0.04193	-0.17529
H	-3.26677	-0.96478	0.40261
H	-4.24122	0.50316	0.21440

H	-3.58172	-0.32418	-1.21172
C	-1.62512	1.47577	1.77794
H	-0.81836	2.19770	1.93615
H	-2.55521	1.92337	2.15226
H	-1.40230	0.59378	2.38303
C	-1.19466	-3.28514	-0.91559
H	-1.17778	-4.26693	-0.42554
H	-2.22092	-2.90617	-0.88443
H	-0.92688	-3.41935	-1.96813
C	1.72167	-2.90064	-0.17632
H	2.06956	-2.94157	-1.21310
H	2.46920	-2.34838	0.40074
H	1.68311	-3.92527	0.21388
C	-0.46481	-2.14520	1.77775
H	-1.49408	-1.81061	1.93551
H	-0.38504	-3.17411	2.15259
H	0.18535	-1.50901	2.38271

Table S9, **A5**
Level of theory: B3LYP-GD3BJ/6-311++G**
Energy: -435534.0750266 Kcal/mol

Si	0.00000	0.00000	0.64125
H	0.00000	0.00000	2.12799
N	-0.77435	1.44967	0.10085
N	-0.86827	-1.39544	0.10085
N	1.64263	-0.05423	0.10085
C	-0.58258	-2.70977	0.64793
H	-0.00752	-2.61926	1.57295
H	-1.51097	-3.24812	0.88120
H	0.00000	-3.33420	-0.04713
C	-1.70686	-1.41313	-1.08260
H	-2.67013	-1.89823	-0.87359
H	-1.90776	-0.39342	-1.41458
H	-1.23862	-1.95683	-1.91863
C	-0.37038	2.18476	-1.08260
H	-0.30885	3.26151	-0.87359
H	0.61316	1.84888	-1.41458
H	-1.07536	2.05109	-1.91863
C	-2.05544	1.85942	0.64793
H	-2.26458	1.31614	1.57295
H	-2.05747	2.93260	0.88120
H	-2.88751	1.66710	-0.04713
C	2.07724	-0.77162	-1.08260
H	2.97898	-1.36328	-0.87359

H	1.29459	-1.45546	-1.41458
H	2.31397	-0.09426	-1.91863
C	2.63802	0.85035	0.64793
H	2.88751	1.66710	-0.04713
H	2.27210	1.30312	1.57295
H	3.56844	0.31552	0.88120

Table S9, **A5**, deprotonated
Level of theory: B3LYP-GD3BJ/6-311++G**
Energy: -435144.6608950 Kcal/mol

Si	0.00000	0.00000	0.99469
N	-0.77106	1.42647	0.12424
N	-0.84983	-1.38100	0.12424
N	1.62089	-0.04548	0.12424
C	-0.65303	-2.73186	0.57260
H	-0.18369	-2.72540	1.56261
H	-1.60827	-3.28615	0.65267
H	0.00000	-3.32742	-0.10010
C	-1.57743	-1.29416	-1.11313
H	-2.55375	-1.81162	-1.04856
H	-1.76580	-0.24858	-1.35764
H	-1.04467	-1.74926	-1.97743
C	-0.33206	2.01317	-1.11313
H	-0.29204	3.11742	-1.04856
H	0.66762	1.65352	-1.35764
H	-0.99257	1.77934	-1.97743
C	-2.03934	1.93147	0.57260
H	-2.26842	1.52178	1.56261
H	-2.04175	3.03588	0.65267
H	-2.88163	1.66371	-0.10010
C	1.90949	-0.71901	-1.11313
H	2.84578	-1.30580	-1.04856
H	1.09818	-1.40494	-1.35764
H	2.03724	-0.03008	-1.97743
C	2.69237	0.80039	0.57260
H	2.88163	1.66371	-0.10010
H	2.45211	1.20362	1.56261
H	3.65003	0.25027	0.65267

Table S9, **A6**
Level of theory: B3LYP-GD3BJ/6-311++G**
Energy: -614998.9002905 Kcal/mol

Si	0.00000	0.00000	0.99877
H	0.00000	0.00000	2.48761
C	0.00000	1.77441	0.37997
C	-0.89325	2.71484	0.91634
C	0.84887	2.18495	-0.65722
C	-0.93955	4.01949	0.43255
H	-1.56339	2.42565	1.72020
C	0.80548	3.49004	-1.14593
H	1.55283	1.47933	-1.08487
C	-0.08905	4.40873	-0.60196
H	-1.63538	4.73230	0.86073
H	1.47000	3.78929	-1.94876
H	-0.12303	5.42431	-0.97988
C	1.53669	-0.88721	0.37997
C	2.79774	-0.58384	0.91634
C	1.46779	-1.82762	-0.65722
C	3.95075	-1.19607	0.43255
H	2.88237	0.14111	1.72020
C	2.61972	-2.44258	-1.14593
H	0.50472	-2.08445	-1.08487
C	3.86259	-2.12725	-0.60196
H	4.91598	-0.94987	0.86073
H	2.54662	-3.16770	-1.94876
H	4.75910	-2.60561	-0.97988
C	-1.53669	-0.88721	0.37997
C	-1.90449	-2.13100	0.91634
C	-2.31666	-0.35733	-0.65722
C	-3.01120	-2.82342	0.43255
H	-1.31898	-2.56676	1.72020
C	-3.42520	-1.04746	-1.14593
H	-2.05755	0.60513	-1.08487
C	-3.77355	-2.28148	-0.60196
H	-3.28060	-3.78243	0.86073
H	-4.01662	-0.62159	-1.94876
H	-4.63607	-2.81870	-0.97988

Table S9, **A6**, deprotonated
Level of theory: B3LYP-GD3BJ/6-311++G**
Energy: -614615.2084918 Kcal/mol

Si	0.00000	0.00000	1.39344
C	0.42830	1.66142	0.47745
C	-0.25258	2.83507	0.85568
C	1.39892	1.80402	-0.53023
C	0.00000	4.06822	0.25940
H	-1.00129	2.77307	1.64193
C	1.67570	3.03766	-1.12158
H	1.94507	0.92855	-0.86471
C	0.97514	4.17945	-0.73419
H	-0.55632	4.94703	0.57413
H	2.43408	3.10531	-1.89719
H	1.18476	5.13919	-1.19610
C	1.22469	-1.20163	0.47745
C	2.58153	-1.19879	0.85568
C	0.86287	-2.11351	-0.53023
C	3.52319	-2.03411	0.25940
H	2.90219	-0.51939	1.64193
C	1.79284	-2.97003	-1.12158
H	-0.16838	-2.14875	-0.86471
C	3.13194	-2.93422	-0.73419
H	4.56241	-1.99173	0.57413
H	1.47224	-3.66063	-1.89719
H	3.85829	-3.59563	-1.19610
C	-1.65298	-0.45980	0.47745
C	-2.32895	-1.63628	0.85568
C	-2.26179	0.30949	-0.53023
C	-3.52319	-2.03411	0.25940
H	-1.90090	-2.25368	1.64193
C	-3.46854	-0.06763	-1.12158
H	-1.77668	1.22020	-0.86471
C	-4.10708	-1.24523	-0.73419
H	-4.00609	-2.95530	0.57413
H	-3.90632	0.55532	-1.89719
H	-5.04305	-1.54356	-1.19610

Table S9, **A7**
Level of theory: B3LYP-GD3BJ/6-311++G**
Energy: -257247.6715152 Kcal/mol

Si	0.00000	0.00000	0.38285
H	0.00000	0.00000	1.87516
C	0.00000	1.78423	-0.22477
H	-0.88356	2.32394	0.12777
H	0.88356	2.32394	0.12777
H	0.00000	1.82239	-1.31863
C	1.54519	-0.89211	-0.22477
H	2.45437	-0.39679	0.12777
H	1.57081	-1.92715	0.12777
H	1.57824	-0.91119	-1.31863
C	-1.54519	-0.89211	-0.22477
H	-1.57081	-1.92715	0.12777
H	-2.45437	-0.39679	0.12777
H	-1.57824	-0.91119	-1.31863

Table S9, **A7**, deprotonated
Level of theory: B3LYP-GD3BJ/6-311++G**
Energy: -256856.3108655 Kcal/mol

Si	0.00000	0.00000	0.68219
C	-0.00000	1.70575	-0.28919
H	-0.88342	2.30450	-0.03275
H	0.88342	2.30450	-0.03275
H	-0.00000	1.55112	-1.38289
C	1.47723	-0.85288	-0.28919
H	2.43746	-0.38719	-0.03275
H	1.55404	-1.91731	-0.03275
H	1.34331	-0.77556	-1.38289
C	-1.47723	-0.85288	-0.28919
H	-1.55404	-1.91731	-0.03275
H	-2.43746	-0.38719	-0.03275
H	-1.34331	-0.77556	-1.38289

Table S9, **A8**
Level of theory: B3LYP-GD3BJ/6-311++G**
Energy: -940170.2854508 Kcal/mol

Si	-0.00011	0.00004	-0.73032
N	-1.62566	-0.11200	-0.11503
C	-2.59681	-1.05109	-0.48367
C	-2.20337	0.71158	0.87071
C	-3.76670	-0.79751	0.27322
C	-3.48753	0.33379	1.12888

N	0.90979	-1.35185	-0.11531
C	2.20900	-1.72293	-0.48316
C	0.48463	-2.26507	0.86919
C	2.57383	-2.86346	0.27304
C	1.45383	-3.18832	1.12736
N	0.71566	1.46401	-0.11524
C	0.38654	2.77466	-0.48243
C	1.72070	1.55251	0.86756
C	1.19290	3.66097	0.27255
C	2.03584	2.85354	1.12538
C	-2.51993	-2.10093	-1.39809
C	-4.89076	-1.61331	0.09039
C	-4.82264	-2.65390	-0.82722
C	-3.64934	-2.89569	-1.56220
H	-1.61477	-2.30421	-1.95877
H	-5.79744	-1.43738	0.65813
H	-5.68366	-3.29425	-0.97867
H	-3.62115	-3.71877	-2.26626
C	3.84246	-3.42894	0.09067
C	3.08042	-1.13078	-1.39653
C	4.71022	-2.84902	-0.82593
C	4.33347	-1.71144	-1.56030
H	4.14295	-4.30262	0.65791
H	5.69537	-3.27442	-0.97705
H	5.03265	-1.27499	-2.26358
H	2.80425	-0.24483	-1.95673
C	-0.56328	3.23307	-1.39454
C	-0.68721	4.60856	-1.55833
C	0.11053	5.50373	-0.82513
C	1.04791	5.04235	0.09025
H	-1.19317	2.55080	-1.95376
H	-1.41572	4.99571	-2.26072
H	-0.01385	6.56957	-0.97626
H	1.65502	5.73953	0.65656
C	3.05565	3.36267	2.09311
H	3.55355	2.53977	2.60960
H	3.82394	3.95444	1.58491
H	2.59828	4.00807	2.85016
C	1.38595	-4.32470	2.09679
H	1.51411	-5.28664	1.58986
H	2.17432	-4.24999	2.85293
H	0.42487	-4.34399	2.61428
C	-4.43727	0.96011	2.09916
H	-3.97298	1.80151	2.61715

H	-5.33437	1.33085	1.59271
H	-4.76688	0.23951	2.85476
H	-0.00038	-0.00009	-2.20260
H	2.13526	0.65035	1.28908
H	-0.50346	-2.17208	1.29161
H	-1.62797	1.51975	1.29398

Table S9, **A8**, deprotonated
Level of theory: B3LYP-GD3BJ/6-311++G**
Energy: -939823.4960701 Kcal/mol

Si	0.00014	-0.00018	-1.30352
N	1.58690	-0.01162	-0.30862
C	2.70126	-0.77947	-0.60743
C	1.89583	0.70564	0.83900
C	3.71006	-0.53504	0.37266
C	3.16474	0.42668	1.28973
N	-0.78354	1.37974	-0.30870
C	-0.67592	2.72870	-0.60757
C	-1.55848	1.28865	0.83941
C	-1.39117	3.48019	0.37308
C	-1.95084	2.52709	1.29049
N	-0.80302	-1.36928	-0.30932
C	-2.02575	-1.94943	-0.60766
C	-0.33627	-1.99605	0.83799
C	-2.31885	-2.94521	0.37240
C	-1.21292	-2.95482	1.28898
C	2.90967	-1.68947	-1.64887
C	4.93638	-1.21117	0.28520
C	5.14044	-2.10891	-0.75531
C	4.13453	-2.34353	-1.71324
H	2.13564	-1.87438	-2.38414
H	5.71417	-1.03764	1.02256
H	6.08345	-2.64009	-0.83288
H	4.31730	-3.05172	-2.51442
C	-1.41847	4.88029	0.28576
C	0.00728	3.36406	-1.64954
C	-0.74351	5.50578	-0.75511
C	-0.03823	4.75187	-1.71362
H	-1.95706	5.46722	1.02348
H	-0.75476	6.58804	-0.83261
H	0.48327	5.26420	-2.51510
H	0.55374	2.78606	-2.38521
C	-2.91833	-1.67385	-1.64855
C	-4.09798	-2.40636	-1.71235

C	-4.39810	-3.39481	-0.75453
C	-3.51825	-3.66810	0.28538
H	-2.69117	-0.91113	-2.38379
H	-4.80295	-2.20968	-2.51307
H	-5.33017	-3.94500	-0.83174
H	-3.75715	-4.42851	1.02259
C	-1.05021	-3.84100	2.48392
H	-0.11265	-3.62601	3.00305
H	-1.86759	-3.70797	3.20330
H	-1.03964	-4.90194	2.20511
C	-2.79862	2.82909	2.48621
H	-2.27432	3.47073	3.20505
H	-3.72310	3.35007	2.20809
H	-3.08043	1.90965	3.00576
C	3.85062	1.01075	2.48478
H	3.19513	1.71423	3.00456
H	4.14523	0.23632	3.20365
H	4.76347	1.55147	2.20587
H	0.61555	-1.70665	1.25514
H	-1.78250	0.31955	1.25696
H	1.16875	1.38451	1.25638

Table S9, **A9**
Level of theory: B3LYP-GD3BJ/6-311++G**
Energy: -1388799.3049000 Kcal/mol

C	2.41625	0.61112	3.46738
Si	1.61348	-0.39784	2.10314
C	1.52321	-2.20309	2.60342
Si	2.55344	-0.00042	-0.00063
Si	1.61424	2.02132	-0.70687
C	1.52712	3.35621	0.60763
O	0.03236	0.18629	1.82068
C	-0.86467	0.53416	2.87553
C	-1.74634	1.66962	2.40935
O	-2.36107	1.27655	1.18699
C	-3.36231	2.19155	0.75304
O	0.03181	1.48654	-1.06851
C	-0.86773	2.23003	-1.89101
C	-1.74673	1.26161	-2.64817
O	-2.35859	0.39068	-1.70284
C	-3.34718	-0.45126	-2.28755
C	2.41455	2.69776	-2.26424
Si	1.61332	-1.62326	-1.39678
C	2.41701	-3.30882	-1.20525

O	0.03214	-1.67131	-0.74964
C	-0.86313	-2.76074	-0.97366
C	-1.74448	-2.92351	0.24303
O	-2.36214	-1.66915	0.51047
C	-3.35775	-1.75007	1.52552
C	1.52234	-1.15285	-3.20989
Li	-1.11087	0.00011	-0.00141
H	-4.16510	0.14668	-2.70435
H	-3.72286	-1.09392	-1.49566
H	-2.91483	-1.06499	-3.08663
H	-2.93168	-2.13012	2.46155
H	-4.17302	-2.41216	1.21379
H	-3.73521	-0.74251	1.67885
H	-4.17798	2.24478	1.48250
H	-3.73750	1.82158	-0.19755
H	-2.94264	3.19543	0.61799
H	-0.32070	-3.69630	-1.14272
H	-1.47222	-2.55379	-1.85847
H	-2.50474	-3.69076	0.04770
H	-1.15179	-3.23400	1.11252
H	2.37350	1.68339	3.26129
H	3.47178	0.33392	3.54697
H	1.96295	0.42786	4.44594
H	0.96950	-2.32723	3.53946
H	2.52609	-2.61121	2.75993
H	1.03195	-2.79834	1.83185
H	2.36876	1.98339	-3.08986
H	3.47082	2.90375	-2.06632
H	1.96181	3.63757	-2.59350
H	0.97147	-1.90268	-3.78649
H	2.52510	-1.08004	-3.64111
H	1.02753	-0.18864	-3.33830
H	-0.32919	2.85352	-2.61165
H	-1.47797	2.88416	-1.26148
H	-0.32375	0.85392	3.77178
H	-1.47368	-0.33656	3.13575
H	-2.51076	1.81667	-3.20767
H	-1.15298	0.67382	-3.35930
H	2.37485	-3.66646	-0.17352
H	3.47242	-3.23834	-1.48540
H	1.96415	-4.06500	-1.85310
H	-2.50847	1.88078	3.17053
H	-1.15465	2.57926	2.24754
H	0.97452	4.23021	0.24859

H	2.53057	3.69381	0.88314
H	1.03543	2.98490	1.50842
H	4.04140	-0.00085	-0.00072

Table S9, **A9**, deprotonated
Level of theory: B3LYP-GD3BJ/6-311++G**
Energy: -1388514.7962922 Kcal/mol

C	1.53404	3.31065	0.31700
H	1.05640	3.23600	-0.66207
H	2.53172	3.73525	0.17159
H	0.95711	4.01389	0.92821
C	2.34704	1.91796	2.88207
H	1.85432	2.73618	3.41770
H	3.40342	2.18337	2.78004
H	2.30083	1.01814	3.50178
C	1.53983	-1.93539	2.70567
H	1.06148	-1.05262	3.13471
H	2.53893	-2.02098	3.14306
H	0.96562	-2.81845	3.00818
C	2.34813	-3.45360	0.21207
H	1.85904	-4.32806	0.65394
H	3.40578	-3.49628	0.48839
H	2.29754	-3.53930	-0.87696
C	1.53519	-1.37771	-3.02535
H	1.05833	-2.18888	-2.47148
H	2.53344	-1.71464	-3.31993
H	0.95834	-1.20053	-3.94011
C	2.34443	1.54137	-3.10014
H	1.85324	1.59497	-4.07738
H	3.40163	1.32294	-3.27730
H	2.29497	2.52792	-2.63081
C	-0.84096	1.62358	2.40604
H	-1.44688	2.45053	2.01650
H	-0.26562	2.00192	3.25739
C	-1.73992	0.50860	2.89337
H	-1.14223	-0.27969	3.36867
H	-2.46175	0.89536	3.62730
C	-0.84137	-2.89667	0.20214
H	-1.44663	-2.97508	1.11328
H	-0.26555	-3.82261	0.10195
C	-1.74188	-2.75969	-1.00595
H	-1.14575	-2.77675	-1.92728
H	-2.46406	-3.58838	-1.03755
C	-3.33591	-1.05548	2.11613

H	-2.82976	-1.86530	2.65687
H	-3.74517	-1.44461	1.18671
H	-4.14224	-0.66129	2.74837
C	-3.34048	-1.30429	-1.96728
H	-2.83675	-1.36656	-2.94033
H	-3.75010	-0.30519	-1.83778
H	-4.14623	-2.04950	-1.94052
C	-3.34029	2.35730	-0.14275
H	-2.83612	3.23289	0.28575
H	-3.74700	1.74771	0.66082
H	-4.14832	2.70406	-0.80014
C	-0.84337	1.27142	-2.60807
H	-1.44782	0.51981	-3.12994
H	-0.26851	1.82055	-3.36103
C	-1.74471	2.24938	-1.88653
H	-1.14904	3.05752	-1.44319
H	-2.46799	2.68878	-2.58874
Li	-0.99529	-0.00140	0.00103
O	-0.00018	1.11513	1.38824
O	-0.00137	-1.76043	0.27290
O	-0.00210	0.64456	-1.65895
O	-2.42243	-0.03025	1.76694
O	-2.42410	-1.51471	-0.90730
O	-2.42522	1.54333	-0.85526
Si	2.88604	0.00118	-0.00068
Si	1.65073	1.60967	1.14432
Si	1.65149	-1.79636	0.81911
Si	1.64974	0.18877	-1.96523

Table S9, **A10**
Level of theory: B3LYP-GD3BJ/6-311++G**
Energy: -1760533.7445208 Kcal/mol

C	2.84350	3.28290	1.04602
Si	1.72631	1.79648	1.29775
C	1.46830	1.45084	3.12470
Si	2.47645	-0.08821	0.11701
Si	1.82158	0.06003	-2.13243
C	1.75893	1.83016	-2.75373
O	0.21268	2.10430	0.58220
C	-0.59569	3.23462	0.90773
C	-1.43258	3.63357	-0.28678
O	-2.32126	2.57088	-0.61838
C	-3.15684	2.87994	-1.72935
O	0.23802	-0.56318	-2.09663

C	-0.55529	-0.77509	-3.26374
C	-1.50841	-1.92731	-3.03825
O	-2.40172	-1.60101	-1.97801
C	-3.33863	-2.63894	-1.70866
C	2.88132	-1.00807	-3.25359
Si	1.59108	-2.03929	1.07490
C	2.53748	-2.57382	2.60444
O	0.02351	-1.55409	1.52730
C	-0.88484	-2.39383	2.23899
C	-1.80379	-1.55275	3.09610
O	-2.59059	-0.70974	2.25991
C	-3.49152	0.11005	2.99761
C	1.45186	-3.44802	-0.15765
K	-1.46707	0.06605	-0.07084
H	-3.97530	-2.82942	-2.57975
H	-3.95990	-2.30765	-0.87661
H	-2.82520	-3.56718	-1.43083
H	-2.94847	0.75994	3.69410
H	-4.20623	-0.50004	3.56096
H	-4.03393	0.72469	2.27924
H	-3.79417	3.74444	-1.51293
H	-3.78593	2.00939	-1.91489
H	-2.55764	3.09176	-2.62293
H	-0.35142	-3.09120	2.89485
H	-1.47572	-2.98411	1.52827
H	-2.45592	-2.21305	3.68364
H	-1.21309	-0.94078	3.79036
H	2.93229	3.53969	-0.01256
H	3.84728	3.06171	1.42046
H	2.48480	4.16522	1.58452
H	1.05421	2.32037	3.64465
H	2.41580	1.20013	3.61067
H	0.78571	0.60806	3.25698
H	2.85798	-2.05608	-2.94459
H	3.92161	-0.67202	-3.21366
H	2.56322	-0.95112	-4.29894
H	0.97013	-4.32552	0.28483
H	2.44097	-3.75907	-0.50645
H	0.86961	-3.13104	-1.02598
H	0.06955	-1.01461	-4.13161
H	-1.12068	0.13544	-3.49612
H	0.02050	4.09448	1.19398
H	-1.24853	2.99048	1.75439
H	-2.07334	-2.11591	-3.96117

H	-0.94597	-2.83525	-2.78388
H	2.55373	-1.78321	3.35879
H	3.57380	-2.80389	2.34022
H	2.11245	-3.47361	3.05933
H	-2.00457	4.53862	-0.04151
H	-0.78245	3.85537	-1.14327
H	1.38154	1.88391	-3.77969
H	2.75639	2.27944	-2.74834
H	1.11187	2.43270	-2.11203
H	3.96550	-0.14478	0.18806

Table S9, **A10**, deprotonated
Level of theory: B3LYP-GD3BJ/6-311++G**
Energy: -1760244.4946146 Kcal/mol

C	-3.55008	2.51679	-1.08226
Si	-2.13292	1.27036	-1.25929
C	-1.69832	1.15947	-3.10159
Si	-2.69351	-0.78612	-0.32267
Si	-1.92843	-0.52781	1.86353
C	-2.35425	1.15419	2.62667
O	-0.77088	2.05726	-0.52474
C	-0.33606	3.35933	-0.85956
C	0.43450	3.95703	0.29810
O	1.62048	3.19629	0.52896
C	2.35511	3.65771	1.64990
O	-0.21020	-0.68002	2.02057
C	0.50046	-0.80863	3.23424
C	1.75922	-1.61720	2.99716
O	2.60041	-0.92424	2.07717
C	3.70837	-1.69920	1.65042
C	-2.63688	-1.86284	3.00783
Si	-1.03695	-2.16551	-1.20330
C	-1.55194	-2.87411	-2.88448
O	0.49390	-1.40314	-1.51392
C	1.60318	-2.09601	-2.04860
C	2.55745	-1.12203	-2.70556
O	3.10529	-0.24550	-1.72033
C	3.95786	0.73959	-2.27908
C	-0.62927	-3.63221	-0.07216
K	1.23011	0.48897	0.16164
H	4.38178	-1.92539	2.48722
H	4.24221	-1.11796	0.89868
H	3.37545	-2.64182	1.19756
H	3.42011	1.36350	-3.00439

H	4.82115	0.28071	-2.77797
H	4.31085	1.36863	-1.46106
H	2.69184	4.69236	1.50567
H	3.22657	3.01102	1.75887
H	1.75269	3.60592	2.56595
H	1.29605	-2.82442	-2.81023
H	2.13225	-2.64752	-1.25861
H	3.37031	-1.67325	-3.20021
H	2.02040	-0.53464	-3.46212
H	-3.77185	2.70245	-0.02712
H	-4.45257	2.09805	-1.53628
H	-3.35045	3.47836	-1.56729
H	-1.51974	2.14591	-3.54373
H	-2.51395	0.68912	-3.65841
H	-0.80214	0.54844	-3.24085
H	-2.32355	-2.86004	2.68485
H	-3.72883	-1.83227	2.95513
H	-2.35260	-1.73399	4.05776
H	0.08033	-4.32874	-0.53200
H	-1.53859	-4.19256	0.16336
H	-0.20447	-3.27201	0.86829
H	-0.09166	-1.32315	4.00120
H	0.76769	0.18136	3.63111
H	-1.18124	4.02473	-1.07672
H	0.30485	3.33613	-1.75243
H	2.29305	-1.77472	3.94572
H	1.48901	-2.59569	2.57918
H	-1.71772	-2.07064	-3.60776
H	-2.49641	-3.41413	-2.77312
H	-0.81895	-3.57048	-3.30609
H	0.70325	4.99866	0.07008
H	-0.19066	3.94470	1.20073
H	-2.03241	1.22300	3.67187
H	-3.43438	1.32594	2.59894
H	-1.87242	1.95474	2.05924

Table S9, **A11**
Level of theory: B3LYP-GD3BJ/6-311++G**
Energy: -963322.4293312 Kcal/mol

C	-2.82143	0.69907	-0.80977
C	-3.51937	0.87146	0.41448
C	-4.87815	1.20750	0.38425
C	-5.50619	1.36311	-0.84569
C	-4.79807	1.18823	-2.04649

C	-3.44762	0.85421	-2.04443
C	-1.38515	0.34367	0.90440
C	-2.58109	0.63929	1.49254
H	-5.43233	1.34441	1.30603
H	-6.55745	1.62312	-0.88323
H	-5.31220	1.31560	-2.99182
H	-2.90686	0.72074	-2.97462
C	0.98883	1.02780	0.90459
C	1.84413	1.91431	1.49290
C	2.01615	2.09300	-0.80937
C	2.51517	2.61023	0.41500
C	2.46404	2.55764	-2.04391
C	3.48673	3.61784	0.38505
C	3.42971	3.55905	-2.04568
H	2.07739	2.15700	-2.97422
C	3.93593	4.08385	-0.84475
H	3.88288	4.02857	1.30696
H	3.79742	3.94054	-2.99091
H	4.68762	4.86343	-0.88209
C	0.39434	-1.36990	0.90458
C	0.73615	-2.55333	1.49295
C	0.80500	-2.79171	-0.80932
C	1.00450	-3.48213	0.41508
C	0.98404	-3.41177	-2.04384
C	1.39303	-4.82685	0.38515
C	1.37026	-4.74824	-2.04559
H	0.82938	-2.87690	-2.97416
C	1.57274	-5.44869	-0.84464
H	1.55150	-5.37505	1.30706
H	1.51733	-5.25730	-2.99081
H	1.87341	-6.48907	-0.88195
Si	-0.00050	0.00053	-1.31834
N	0.42861	-1.48902	-0.50503
N	1.07462	1.11721	-0.50503
N	-1.50518	0.37357	-0.50523
C	0.82538	-2.86138	2.95283
H	0.57263	-1.99276	3.56412
H	0.14293	-3.67160	3.22927
H	1.83576	-3.17940	3.22968
C	-2.89291	0.71589	2.95235
H	-3.25281	1.71217	3.22897
H	-3.67415	0.00039	3.22868
H	-2.01468	0.49962	3.56392
C	2.06711	2.14495	2.95276

H	1.84272	3.18026	3.22917
H	1.43753	1.49534	3.56406
H	3.10894	1.95365	3.22961
H	-0.00030	0.00045	-2.78252
C	-0.00079	0.00063	1.43196
H	-0.00082	0.00068	2.51840

Table S9, **A11**, deprotonated
Level of theory: B3LYP-GD3BJ/6-311++G**
Energy: -962992.4486890 Kcal/mol

Si	0.00072	0.00038	-1.58882
N	-0.54040	-1.42929	-0.49788
C	-1.01766	-2.68875	-0.79617
C	-0.49974	-1.32208	0.88686
C	-1.27860	-3.37809	0.42895
C	-0.93658	-2.47525	1.49722
N	1.50936	0.24652	-0.49792
C	2.83866	0.46325	-0.79627
C	1.39618	0.22825	0.88682
C	3.56608	0.58229	0.42883
C	2.61322	0.42704	1.49714
N	-0.96695	1.18370	-0.49786
C	-1.82015	2.22581	-0.79623
C	-0.89455	1.09482	0.88690
C	-2.28746	2.79587	0.42886
C	-1.67611	2.04871	1.49718
C	-1.24851	-3.29649	-2.03261
C	-1.77507	-4.68830	0.38848
C	-2.00314	-5.28910	-0.84513
C	-1.74153	-4.59749	-2.04298
H	-1.04682	-2.76282	-2.95397
H	-1.97939	-5.22824	1.30803
H	-2.38734	-6.30292	-0.88829
H	-1.92788	-5.08811	-2.99254
C	4.94892	0.80790	0.38832
C	3.48038	0.56740	-2.03274
C	5.58322	0.91089	-0.84530
C	4.85351	0.79136	-2.04314
H	5.51871	0.90108	1.30784
H	6.65327	1.08539	-0.88849
H	5.37153	0.87541	-2.99272
H	2.91744	0.47509	-2.95414
C	-2.23198	2.72879	-2.03271
C	-3.11366	3.80503	-2.04314

C	-3.58243	4.37695	-0.84532
C	-3.17536	3.87975	0.38832
H	-1.87042	2.28746	-2.95406
H	-3.44617	4.21103	-2.99273
H	-4.26953	5.21559	-0.88853
H	-3.54144	4.32624	1.30783
C	-1.87004	2.28388	2.96163
H	-1.29067	1.57592	3.55946
H	-1.55776	3.29375	3.25444
H	-2.92196	2.17616	3.25314
C	2.91379	0.47619	2.96163
H	3.62927	-0.30187	3.25460
H	3.34986	1.43944	3.25327
H	2.01029	0.33171	3.55917
C	-1.04438	-2.76011	2.96173
H	-0.72271	-1.90355	3.55933
H	-2.07513	-2.99564	3.25354
H	-0.42441	-3.61628	3.25454
C	0.00070	0.00034	1.42413
H	0.00072	0.00020	2.51190

Table S9, **A12**
Level of theory: B3LYP-GD3BJ/6-311++G**
Energy: -1145787.1183781 Kcal/mol

Si	0.00000	-0.00076	-1.17326
N	-1.37925	-0.79718	-0.44322
C	-2.49885	-1.44205	-0.95521
C	-1.45349	-0.84018	0.98231
C	-3.28083	-1.89285	0.13783
C	-2.60033	-1.50100	1.34928
N	1.37925	-0.79716	-0.44322
C	2.49887	-1.44202	-0.95521
C	1.45350	-0.84016	0.98231
C	3.28085	-1.89281	0.13783
C	2.60035	-1.50096	1.34928
N	-0.00001	1.59167	-0.44271
C	-0.00002	2.88381	-0.95441
C	-0.00001	1.67714	0.98281
C	-0.00002	3.78622	0.13879
C	-0.00002	3.00068	1.35007
C	-2.88652	-1.66494	-2.27736
C	-4.47640	-2.58148	-0.10918
C	-4.86451	-2.80449	-1.42308
C	-4.07594	-2.34991	-2.49527

H	-2.28662	-1.31916	-3.11110
H	-5.08839	-2.93432	0.71336
H	-5.78616	-3.33525	-1.63046
H	-4.40159	-2.53709	-3.51181
C	4.47644	-2.58142	-0.10917
C	2.88655	-1.66490	-2.27736
C	4.86455	-2.80443	-1.42308
C	4.07597	-2.34986	-2.49527
H	5.08843	-2.93425	0.71336
H	5.78620	-3.33517	-1.63046
H	4.40163	-2.53703	-3.51181
H	2.28664	-1.31913	-3.11110
C	-0.00002	3.33114	-2.27649
C	-0.00003	4.70371	-2.49423
C	-0.00004	5.61376	-1.42190
C	-0.00003	5.16597	-0.10805
H	-0.00002	2.63877	-3.11028
H	-0.00004	5.07948	-3.51071
H	-0.00005	6.67734	-1.62911
H	-0.00004	5.87232	0.71453
C	-0.00002	3.57125	2.73310
H	-0.00001	2.79056	3.49387
H	0.88060	4.20038	2.89996
H	-0.88065	4.20037	2.89996
C	3.09502	-1.78605	2.73213
H	3.20093	-2.86318	2.89862
H	4.07964	-1.33679	2.89896
H	2.41852	-1.39680	3.49311
C	-3.09499	-1.78610	2.73213
H	-2.41850	-1.39683	3.49311
H	-4.07962	-1.33685	2.89896
H	-3.20089	-2.86323	2.89862
C	-0.00000	-0.00159	3.69734
H	-0.88520	0.50938	4.08184
H	0.00001	-1.02378	4.08158
H	0.88520	0.50939	4.08184
H	0.00000	-0.00042	-2.63770
Si	-0.00000	-0.00131	1.83449

Table S9, **A12**, deprotonated
Level of theory: B3LYP-GD3BJ/6-311++G**
Energy: -1145449.3909182 Kcal/mol

Si	0.00000	-0.00044	-1.45335
N	-1.37096	-0.79295	-0.43864
C	-2.48642	-1.43649	-0.93753
C	-1.45122	-0.83951	0.96427
C	-3.28078	-1.89519	0.15550
C	-2.59936	-1.50212	1.35624
N	1.37165	-0.79177	-0.43864
C	2.48766	-1.43435	-0.93753
C	1.45195	-0.83826	0.96426
C	3.28242	-1.89236	0.15550
C	2.60065	-1.49988	1.35624
N	-0.00068	1.58288	-0.43822
C	-0.00124	2.87080	-0.93672
C	-0.00072	1.67526	0.96469
C	-0.00163	3.78780	0.15655
C	-0.00130	3.00077	1.35707
C	-2.88310	-1.66498	-2.26111
C	-4.47677	-2.58519	-0.09612
C	-4.86483	-2.80855	-1.41065
C	-4.07116	-2.35030	-2.48164
H	-2.27386	-1.31330	-3.08467
H	-5.09060	-2.93968	0.72634
H	-5.78706	-3.34049	-1.61993
H	-4.39502	-2.53676	-3.50031
C	4.47900	-2.58133	-0.09612
C	2.88452	-1.66251	-2.26111
C	4.86725	-2.80436	-1.41065
C	4.07318	-2.34680	-2.48165
H	5.09314	-2.93530	0.72633
H	5.78993	-3.33552	-1.61993
H	4.39720	-2.53300	-3.50032
H	2.27498	-1.31136	-3.08467
C	-0.00143	3.32887	-2.26018
C	-0.00201	4.70047	-2.48040
C	-0.00242	5.61667	-1.40919
C	-0.00223	5.16862	-0.09477
H	-0.00112	2.62556	-3.08386
H	-0.00217	5.07442	-3.49898
H	-0.00287	6.68137	-1.61820
H	-0.00254	5.87730	0.72783
C	-0.00154	3.55468	2.74836

H	-0.00120	2.76138	3.49711
H	0.87908	4.18282	2.93100
H	-0.88271	4.18206	2.93100
C	3.08088	-1.77674	2.74733
H	3.18465	-2.85347	2.92984
H	4.06483	-1.32728	2.92970
H	2.39386	-1.38046	3.49628
C	-3.07934	-1.77940	2.74734
H	-2.39267	-1.38253	3.49629
H	-4.06367	-1.33079	2.92970
H	-3.18219	-2.85622	2.92984
C	0.00000	-0.00105	3.66550
H	-0.88558	0.50975	4.05099
H	0.00044	-1.02334	4.05109
H	0.88515	0.51051	4.05099
Si	0.00000	-0.00102	1.79009

Table S10, (*N*-Sk)$_3$Si-**R**, **R**=LP
Level of theory: B3LYP-GD3BJ/6-311++G**
Energy: -939823.4963713 Kcal/mol

Si	0.00040	0.00014	-1.30354
N	-1.15122	1.09147	-0.30848
C	-1.44147	2.41324	-0.60732
C	-1.86592	0.77842	0.83968
C	-2.34476	2.92355	0.37330
C	-2.60229	1.84868	1.29071
N	-0.36943	-1.54334	-0.30926
C	-1.37031	-2.45431	-0.60749
C	0.26007	-2.00730	0.83768
C	-1.36042	-3.49250	0.37235
C	-0.29923	-3.17994	1.28856
N	1.52177	0.45111	-0.30875
C	2.81139	0.04084	-0.60742
C	1.60855	1.22799	0.83841
C	3.70537	0.56890	0.37235
C	2.90374	1.33072	1.28901
C	-0.97311	3.22024	-1.64920
C	-2.77926	4.25480	0.28587
C	-2.31599	5.04993	-0.75498
C	-1.42138	4.53445	-1.71337
H	-0.28167	2.82679	-2.38474
H	-3.46572	4.65912	1.02348
H	-2.64243	6.08184	-0.83257

H	-1.07182	5.17658	-2.51480
C	-2.29740	-4.53329	0.28543
C	-2.30490	-2.45032	-1.64800
C	-3.21912	-4.52778	-0.75409
C	-3.22017	-3.49457	-1.71169
H	-2.30421	-5.33050	1.02245
H	-3.95061	-5.32552	-0.83120
H	-3.95223	-3.51143	-2.51207
H	-2.31004	-1.65431	-2.38303
C	3.27567	-0.76975	-1.64835
C	4.63786	-1.03907	-1.71251
C	5.53193	-0.52125	-0.75489
C	5.07539	0.27892	0.28508
H	2.58902	-1.17247	-2.38337
H	5.01883	-1.66393	-2.51328
H	6.58871	-0.75499	-0.83232
H	5.76902	0.67188	1.02213
C	3.39006	2.09002	2.48344
H	2.55815	2.57170	3.00366
H	3.89839	1.43553	3.20216
H	4.10509	2.87357	2.20374
C	0.11493	-3.98072	2.48309
H	-0.70542	-4.09148	3.20286
H	0.43361	-4.99260	2.20384
H	0.94963	-3.50237	3.00195
C	-3.50174	1.89042	2.48609
H	-3.50242	0.92922	3.00649
H	-3.18832	2.65803	3.20432
H	-4.53810	2.11787	2.20742
H	0.71539	1.66611	1.25561
H	1.08654	-1.45335	1.25460
H	-1.79793	-0.21395	1.25720

Table S10, (*N*-Sk)$_3$Si-**R**, **R**=FeBr$_2$(THF)
Level of theory: B3LYP-GD3BJ/6-311++G**
Energy: -5109490.6590556 Kcal/mol

Si	-0.59902	0.04523	0.22090
N	-2.07880	-0.96544	0.40989
C	-2.88685	-1.41785	-0.63053
C	-2.66400	-1.40303	1.59775
C	-3.97613	-2.14325	-0.07304
C	-3.80704	-2.11882	1.35736
N	0.01796	-0.01421	1.93408

C	0.71036	-1.07293	2.51023
C	0.15920	1.07470	2.79291
C	1.28331	-0.63092	3.73623
C	0.91276	0.75140	3.89303
N	-1.32942	1.69895	0.24562
C	-0.67095	2.90405	0.01244
C	-2.61138	2.01859	0.69221
C	-1.55928	3.97139	0.32480
C	-2.79773	3.37442	0.75236
C	-2.76086	-1.23146	-2.00953
C	-4.94028	-2.70231	-0.92404
C	-4.80813	-2.52681	-2.29559
C	-3.72917	-1.79810	-2.82968
H	-1.94256	-0.66438	-2.43718
H	-5.77672	-3.26130	-0.51691
H	-5.54526	-2.95386	-2.96716
H	-3.64786	-1.67185	-3.90323
C	2.04815	-1.52521	4.49964
C	0.88759	-2.37780	2.04569
C	2.23167	-2.82075	4.03187
C	1.65853	-3.23960	2.81617
H	2.49219	-1.20927	5.43823
H	2.82478	-3.52240	4.60869
H	1.82515	-4.25158	2.46585
H	0.46689	-2.70664	1.10450
C	0.63359	3.14668	-0.42429
C	1.03988	4.46959	-0.55090
C	0.17334	5.53668	-0.24911
C	-1.12363	5.29737	0.18566
H	1.30601	2.33816	-0.67670
H	2.04407	4.68153	-0.90116
H	0.52462	6.55666	-0.36337
H	-1.79020	6.12246	0.41521
C	-4.03995	4.10085	1.16117
H	-4.82744	3.39876	1.44567
H	-3.85749	4.76338	2.01552
H	-4.42757	4.72536	0.34775
C	1.27715	1.64777	5.03421
H	0.88457	1.27145	5.98630
H	2.36424	1.73353	5.14826
H	0.87833	2.65372	4.88305
C	-4.69943	-2.76647	2.36836
H	-4.33691	-2.58545	3.38325
H	-5.72509	-2.38413	2.30565

H	-4.75228	-3.85166	2.22175
H	-3.30628	1.22788	0.92787
H	-0.29451	2.01982	2.54028
H	-2.19245	-1.17127	2.53956
Fe	1.00114	-0.50159	-1.57058
Br	0.57731	0.72727	-3.60333
Br	2.00656	-2.71506	-1.54659
O	2.74461	0.51099	-0.77489
C	3.13534	0.26841	0.61020
C	3.91934	0.55325	-1.62952
C	4.64288	0.48263	0.63860
H	2.87194	-0.75964	0.85988
H	2.56870	0.95317	1.23988
C	5.05817	0.02282	-0.76656
H	4.07243	1.59354	-1.93215
H	3.71138	-0.05101	-2.51134
H	4.88134	1.54088	0.78352
H	5.11517	-0.09057	1.43796
H	5.07845	-1.06852	-0.81575
H	6.02999	0.40923	-1.08058

Table S10, (*N*-Sk)$_3$Si-**R**, **R**=Me
Level of theory: B3LYP-GD3BJ/6-311++G**
Energy: -964863.2950221 Kcal/mol

Si	-0.00059	-0.00091	-0.72977
N	-1.62143	-0.04425	-0.07300
C	-2.64276	-0.93995	-0.40865
C	-2.12741	0.79347	0.93839
C	-3.77445	-0.64185	0.39112
C	-3.41715	0.47014	1.24154
N	0.84800	-1.38357	-0.07523
C	2.13615	-1.81635	-0.40890
C	0.37464	-2.24477	0.93216
C	2.44445	-2.94688	0.38861
C	1.30109	-3.19856	1.23529
N	0.77294	1.42463	-0.07384
C	0.50410	2.75676	-0.40730
C	1.75723	1.44484	0.93184
C	1.33142	3.58858	0.38818
C	2.12193	2.72383	1.23359
C	-2.64482	-1.99282	-1.32381
C	-4.93809	-1.40862	0.24716
C	-4.94766	-2.44897	-0.67295

C	-3.81156	-2.73943	-1.44764
H	-1.76990	-2.23803	-1.91383
H	-5.81522	-1.19599	0.84791
H	-5.84002	-3.05174	-0.79433
H	-3.84181	-3.56348	-2.15059
C	3.69229	-3.56767	0.24630
C	3.05010	-1.28680	-1.32010
C	4.59921	-3.05098	-0.66999
C	4.28199	-1.92045	-1.44236
H	3.94730	-4.43456	0.84542
H	5.56891	-3.51965	-0.79002
H	5.01168	-1.53076	-2.14221
H	2.82440	-0.40526	-1.90808
C	-0.41262	3.28403	-1.31705
C	-0.47847	4.66771	-1.43997
C	0.34430	5.50722	-0.66957
C	1.24640	4.97965	0.24529
H	-1.06501	2.64823	-1.90349
H	-1.18158	5.10522	-2.13878
H	0.26627	6.58135	-0.79007
H	1.87152	5.63359	0.84284
C	3.14264	3.16194	2.23475
H	3.59311	2.30426	2.73815
H	3.94691	3.73245	1.75878
H	2.69844	3.80651	3.00038
C	1.17175	-4.29971	2.23869
H	1.26454	-5.28228	1.76453
H	1.95228	-4.23500	3.00390
H	0.20388	-4.26101	2.74235
C	-4.30350	1.13134	2.24817
H	-3.78339	1.94677	2.75462
H	-5.20005	1.54660	1.77631
H	-4.63913	0.42102	3.01092
H	2.12399	0.51514	1.33721
H	-0.61387	-2.09758	1.33766
H	-1.50341	1.57289	1.34617
C	-0.00163	0.00013	-2.59131
H	0.32191	-0.96840	-2.97870
H	-1.00267	0.20456	-2.97724
H	0.67532	0.76525	-2.97752

Table S10, (*N*-Sk)$_3$Si-**R**, **R**=H
Level of theory: B3LYP-GD3BJ/6-311++G**
Energy: -940170.2866430 Kcal/mol

Si	0.00083	-0.00067	-0.73051
N	-1.48579	-0.66747	-0.11480
C	-2.07327	-1.88406	-0.48352
C	-2.31195	-0.09444	0.87163
C	-3.25819	-2.05038	0.27417
C	-3.38607	-0.89289	1.13067
N	1.32216	-0.95403	-0.11527
C	2.66947	-0.85238	-0.48314
C	1.23944	-1.95745	0.86955
C	3.40669	-1.79553	0.27369
C	2.46841	-2.48771	1.12833
N	0.16628	1.62056	-0.11617
C	-0.59670	2.73595	-0.48340
C	1.07764	2.05172	0.86739
C	-0.14796	3.84668	0.27205
C	0.92226	3.38130	1.12524
C	-1.64000	-2.84213	-1.39938
C	-4.03213	-3.20363	0.09090
C	-3.61026	-4.15589	-0.82815
C	-2.42625	-3.97739	-1.56405
H	-0.72097	-2.72016	-1.96103
H	-4.94340	-3.35170	0.65927
H	-4.19776	-5.05372	-0.98003
H	-2.11665	-4.73944	-2.26931
C	4.79267	-1.88681	0.09116
C	3.28173	0.00401	-1.39745
C	5.40567	-1.04343	-0.82664
C	4.65825	-0.10724	-1.56163
H	5.37725	-2.60195	0.65880
H	6.47710	-1.10181	-0.97823
H	5.16286	0.54337	-2.26593
H	2.71584	0.73888	-1.95847
C	-1.64634	2.83680	-1.39576
C	-2.23973	4.08395	-1.55904
C	-1.80232	5.19993	-0.82516
C	-0.76315	5.09206	0.09019
H	-2.00036	1.97874	-1.95558
H	-3.05723	4.19465	-2.26146
H	-2.28884	6.15645	-0.97579
H	-0.43578	5.95637	0.65691
C	1.70188	4.21261	2.09322

H	2.45472	3.61377	2.60942
H	2.21668	5.03453	1.58515
H	1.04899	4.65887	2.85048
C	2.79752	-3.57740	2.09781
H	3.25030	-4.43572	1.59085
H	3.51126	-3.23491	2.85418
H	1.90215	-3.92775	2.61493
C	-4.49252	-0.63394	2.10249
H	-4.34702	0.31572	2.62097
H	-5.46317	-0.59581	1.59741
H	-4.55166	-1.42445	2.85777
H	0.00036	-0.00148	-2.20281
H	1.77845	1.34879	1.28939
H	0.28010	-2.21210	1.29187
H	-2.05207	0.86299	1.29506

Table S10, $(2\text{-Sk})_3$Si-Me
Level of theory: B3LYP-GD3BJ/6-311++G**
Energy: -964832.8833592 Kcal/mol

N	-2.31979	-0.23841	-0.25075
C	-3.45577	0.40052	-0.68246
C	-1.63501	0.56176	0.66442
C	-3.50448	1.65889	-0.02255
C	-2.34239	1.74150	0.82188
N	1.36492	-1.88834	-0.25179
C	1.37994	-3.19163	-0.68328
C	0.33093	-1.69601	0.66508
C	0.31594	-3.86367	-0.02177
C	-0.33607	-2.89893	0.82354
N	0.95364	2.12760	-0.25119
C	2.07545	2.79122	-0.68273
C	1.30332	1.13527	0.66520
C	3.18896	2.20460	-0.02143
C	2.67861	1.15782	0.82361
C	-4.44271	-0.00064	-1.58779
C	-4.57742	2.52663	-0.28439
C	-5.55696	2.13062	-1.18131
C	-5.48853	0.87858	-1.82631
H	-4.39350	-0.96219	-2.08587
H	-4.63931	3.49026	0.20887
H	-6.39105	2.78912	-1.39332
H	-6.26986	0.59810	-2.52298
C	0.10150	-5.22689	-0.28320
C	2.22009	-3.84525	-1.58967

C	0.93345	-5.87669	-1.18122
C	1.98219	-5.19077	-1.82770
H	-0.70101	-5.76286	0.21121
H	0.78065	-6.92841	-1.39292
H	2.61523	-5.72682	-2.52512
H	3.02719	-3.32135	-2.08883
C	2.22244	3.84555	-1.58904
C	3.50709	4.31101	-1.82714
C	4.62499	3.74459	-1.18079
C	4.47724	2.69927	-0.28286
H	1.36559	4.28349	-2.08812
H	3.65555	5.12718	-2.52451
H	5.61257	4.13718	-1.39250
H	5.34224	2.27134	0.21148
C	3.50366	0.27986	1.71422
H	2.91916	-0.54443	2.12680
H	4.34609	-0.15803	1.16927
H	3.92437	0.84412	2.55343
C	-1.50838	-3.17570	1.71448
H	-2.30851	-3.68704	1.16975
H	-1.22925	-3.82201	2.55354
H	-1.93063	-2.25775	2.12722
C	-1.99435	2.89601	1.71105
H	-0.98810	2.80221	2.12335
H	-2.03648	3.84390	1.16504
H	-2.69309	2.97923	2.55040
C	-0.00142	0.00070	3.25022
H	-0.23641	0.99503	3.63724
H	-0.74540	-0.69962	3.63716
H	0.97670	-0.29312	3.63831
Si	-0.00048	0.00043	1.37548
H	0.00473	2.30093	-0.54624
H	-1.99517	-1.14701	-0.54509
H	1.98922	-1.15280	-0.54637

Table S10, anionic R’=SiMe cage, **R**=LP
Level of theory: B3LYP-GD3BJ/6-311++G**
Energy: -1145449.3909182 Kcal/mol

Si	0.00000	-0.00044	-1.45335
N	-1.37096	-0.79295	-0.43864
C	-2.48642	-1.43649	-0.93753
C	-1.45122	-0.83951	0.96427
C	-3.28078	-1.89519	0.15550

C	-2.59936	-1.50212	1.35624
N	1.37165	-0.79177	-0.43864
C	2.48766	-1.43435	-0.93753
C	1.45195	-0.83826	0.96426
C	3.28242	-1.89236	0.15550
C	2.60065	-1.49988	1.35624
N	-0.00068	1.58288	-0.43822
C	-0.00124	2.87080	-0.93672
C	-0.00072	1.67526	0.96469
C	-0.00163	3.78780	0.15655
C	-0.00130	3.00077	1.35707
C	-2.88310	-1.66498	-2.26111
C	-4.47677	-2.58519	-0.09612
C	-4.86483	-2.80855	-1.41065
C	-4.07116	-2.35030	-2.48164
H	-2.27386	-1.31330	-3.08467
H	-5.09060	-2.93968	0.72634
H	-5.78706	-3.34049	-1.61993
H	-4.39502	-2.53676	-3.50031
C	4.47900	-2.58133	-0.09612
C	2.88452	-1.66251	-2.26111
C	4.86725	-2.80436	-1.41065
C	4.07318	-2.34680	-2.48165
H	5.09314	-2.93530	0.72633
H	5.78993	-3.33552	-1.61993
H	4.39720	-2.53300	-3.50032
H	2.27498	-1.31136	-3.08467
C	-0.00143	3.32887	-2.26018
C	-0.00201	4.70047	-2.48040
C	-0.00242	5.61667	-1.40919
C	-0.00223	5.16862	-0.09477
H	-0.00112	2.62556	-3.08386
H	-0.00217	5.07442	-3.49898
H	-0.00287	6.68137	-1.61820
H	-0.00254	5.87730	0.72783
C	-0.00154	3.55468	2.74836
H	-0.00120	2.76138	3.49711
H	0.87908	4.18282	2.93100
H	-0.88271	4.18206	2.93100
C	3.08088	-1.77674	2.74733
H	3.18465	-2.85347	2.92984
H	4.06483	-1.32728	2.92970
H	2.39386	-1.38046	3.49628
C	-3.07934	-1.77940	2.74734

H	-2.39267	-1.38253	3.49629
H	-4.06367	-1.33079	2.92970
H	-3.18219	-2.85622	2.92984
C	0.00000	-0.00105	3.66550
H	-0.88558	0.50975	4.05099
H	0.00044	-1.02334	4.05109
H	0.88515	0.51051	4.05099
Si	0.00000	-0.00102	1.79009

Table S10, R'=SiMe cage complex, **R**=$FeBr_2$(THF)
Level of theory: B3LYP-GD3BJ/6-311++G**
Energy: -5315112.0914705 Kcal/mol

C	0.50357	-1.11335	2.61862
C	1.48143	-1.81841	3.37681
C	1.13324	-2.38927	4.61020
C	-0.16627	-2.24549	5.07640
C	-1.11692	-1.52446	4.32940
C	-0.79685	-0.95140	3.10520
C	2.45304	-1.05923	1.48069
C	2.71109	-1.76415	2.63597
H	1.87259	-2.92931	5.19305
H	-0.45085	-2.67691	6.03014
H	-2.11931	-1.39237	4.72151
H	-1.53471	-0.36436	2.57775
Si	3.40007	-0.56769	-0.05111
C	2.98631	1.24374	-0.21568
C	3.67703	2.42746	-0.35919
C	1.41825	2.87005	-0.30733
C	2.69921	3.47980	-0.41974
C	0.25037	3.63883	-0.33387
C	2.79960	4.87235	-0.55833
C	0.37652	5.01420	-0.47177
H	-0.73010	3.18917	-0.25527
C	1.63763	5.63050	-0.58301
H	3.77157	5.34749	-0.64494
H	-0.52036	5.62255	-0.49505
H	1.69646	6.70835	-0.68973
C	2.32739	-1.34731	-1.36604
C	2.49246	-2.27663	-2.36964
C	0.28739	-1.62200	-2.29349
C	1.20380	-2.47542	-2.97211
C	-1.05396	-1.56246	-2.68353
C	0.75562	-3.29172	-4.02137

C	-1.47319	-2.37956	-3.72561
H	-1.74869	-0.87599	-2.22354
C	-0.58239	-3.24505	-4.38780
H	1.44814	-3.94570	-4.54163
H	-2.50799	-2.32824	-4.04647
H	-0.94417	-3.86656	-5.19990
Si	0.34862	0.19471	0.00720
N	0.97507	-0.94027	-1.29655
N	1.09720	-0.66029	1.44717
C	3.74914	-2.97252	-2.79123
H	4.60917	-2.64407	-2.20707
H	3.66276	-4.05947	-2.67415
H	3.97375	-2.78285	-3.84741
C	4.00484	-2.37209	3.08073
H	4.32127	-1.96964	4.05022
H	3.91556	-3.45847	3.20080
H	4.80734	-2.18284	2.36715
C	5.15774	2.63125	-0.44130
H	5.51622	3.27947	0.36708
H	5.70080	1.68783	-0.37433
H	5.44190	3.11207	-1.38483
C	5.21790	-1.01386	-0.08657
H	5.67249	-0.69818	-1.02845
H	5.74913	-0.52268	0.73193
H	5.35419	-2.09263	0.01525
N	1.59731	1.49898	-0.18241
Fe	-2.06169	0.80273	0.03784
Br	-2.97125	1.57556	-2.06975
Br	-3.02420	1.84778	1.99464
O	-2.85058	-1.17307	0.16817
C	-2.19203	-2.45369	0.35753
C	-4.27958	-1.35684	-0.02881
C	-3.16256	-3.46661	-0.22915
H	-2.02758	-2.60230	1.42733
H	-1.23367	-2.41329	-0.15461
C	-4.52363	-2.85664	0.14025
H	-4.51356	-0.99877	-1.03340
H	-4.79684	-0.73404	0.70181
H	-3.03701	-3.51353	-1.31376
H	-3.01326	-4.46607	0.18337
H	-4.77210	-3.08921	1.17964
H	-5.33925	-3.21015	-0.49326

Table S10, R'=SiMe cage complex, **R**=Me
Level of theory: B3LYP-GD3BJ/6-311++G**
Energy: -1170481.1029988 Kcal/mol

C	0.85657	2.89562	0.00000
C	-0.24642	3.78875	0.00000
C	-0.02140	5.17182	0.00000
C	1.28425	5.64168	0.00000
C	2.36761	4.74624	0.00000
C	2.17076	3.37024	0.00000
C	-1.06866	1.67499	0.00000
C	-1.44940	2.99396	0.00000
H	-0.85623	5.86372	0.00000
H	1.47540	6.70826	0.00000
H	3.37934	5.13493	0.00000
H	3.02028	2.70114	0.00000
Si	-1.91738	-0.00031	0.00000
C	-1.06822	-0.83789	1.45065
C	-1.44868	-1.49764	2.59283
C	0.85726	-1.44768	2.50758
C	-0.24555	-1.89464	3.28109
C	2.17152	-1.68443	2.91869
C	-0.02033	-2.58606	4.47887
C	2.36861	-2.37228	4.11040
H	3.02090	-1.34945	2.33928
C	1.28540	-2.82044	4.88586
H	-0.85510	-2.93235	5.07797
H	3.38040	-2.56616	4.44710
H	1.47675	-3.35361	5.80956
C	-1.06822	-0.83789	-1.45065
C	-1.44868	-1.49764	-2.59283
C	0.85726	-1.44768	-2.50758
C	-0.24555	-1.89464	-3.28109
C	2.17152	-1.68443	-2.91869
C	-0.02033	-2.58606	-4.47887
C	2.36861	-2.37228	-4.11040
H	3.02090	-1.34945	-2.33928
C	1.28540	-2.82044	-4.88586
H	-0.85510	-2.93235	-5.07797
H	3.38040	-2.56616	-4.44710
H	1.47675	-3.35361	-5.80956
Si	1.10580	0.00020	-0.00000
N	0.35720	-0.79806	-1.38230
N	0.35672	1.59618	0.00000
C	-2.83614	-1.77817	-3.07753

H	-3.59123	-1.38329	-2.39800
H	-3.00735	-1.33163	-4.06268
H	-3.00835	-2.85491	-3.17862
C	-2.83696	3.55379	0.00000
H	-3.00878	4.18167	0.88061
H	-3.00878	4.18167	-0.88061
H	-3.59197	2.76779	0.00000
C	-2.83614	-1.77817	3.07753
H	-3.00735	-1.33163	4.06268
H	-3.59123	-1.38329	2.39800
H	-3.00835	-2.85491	3.17862
C	-3.78110	-0.00027	0.00000
H	-4.16582	-1.02225	-0.00000
H	-4.16562	0.51080	0.88512
H	-4.16562	0.51080	-0.88512
C	2.95131	0.00152	0.00000
H	3.33072	0.51363	-0.88606
H	3.33072	0.51363	0.88606
H	3.33276	-1.02109	-0.00000
N	0.35720	-0.79806	1.38230

Table S10, $(2\text{-Sk})_3CH$
Level of theory: B3LYP-GD3BJ/6-311++G**
Energy: -782367.8734604 Kcal/mol

N	-1.97941	-0.64345	-0.38626
C	-3.28493	-0.26813	-0.62200
C	-1.43696	0.16434	0.60191
C	-3.56056	0.83952	0.22497
C	-2.36883	1.09730	0.99476
N	1.54658	-1.39135	-0.38609
C	1.87467	-2.70953	-0.62186
C	0.57562	-1.32579	0.60194
C	1.05328	-3.50230	0.22494
C	0.23392	-2.59938	0.99476
N	0.43237	2.03520	-0.38641
C	1.41057	2.97776	-0.62221
C	0.85991	1.16203	0.60255
C	2.50698	2.66294	0.22570
C	2.13356	1.50264	0.99611
C	-4.23849	-0.79072	-1.49640
C	-4.82952	1.43553	0.17476
C	-5.77930	0.92014	-0.69588
C	-5.48697	-0.18260	-1.52106
H	-4.01469	-1.63798	-2.13450
H	-5.06512	2.28359	0.80790

H	-6.76400	1.37003	-0.74415
H	-6.25029	-0.56346	-2.18928
C	1.17189	-4.89922	0.17470
C	2.80431	-3.27385	-1.49608
C	2.09343	-5.46385	-0.69575
C	2.90224	-4.65910	-1.52074
H	0.55527	-5.52743	0.80774
H	2.19642	-6.54154	-0.74402
H	3.61398	-5.12952	-2.18884
H	3.42613	-2.65629	-2.13411
C	1.43573	4.06420	-1.49740
C	2.58693	4.84090	-1.52195
C	3.68749	4.54292	-0.69588
C	3.65795	3.46339	0.17560
H	0.59060	4.29383	-2.13625
H	2.63948	5.69184	-2.19081
H	4.56974	5.17036	-0.74412
H	4.50971	3.24353	0.80944
C	2.98079	0.80595	2.01416
H	2.46429	-0.05181	2.44893
H	3.91279	0.43534	1.57429
H	3.25713	1.48198	2.82988
C	-0.79359	-2.98552	2.01196
H	-1.58036	-3.60700	1.57127
H	-0.34668	-3.56347	2.82744
H	-1.27836	-2.10966	2.44721
C	-2.18904	2.18005	2.01206
H	-1.18698	2.16363	2.44466
H	-2.33689	3.17203	1.57213
H	-2.91074	2.08029	2.82936
H	-0.51214	2.05087	-0.73882
H	-1.52064	-1.46947	-0.73808
H	2.03255	-0.58086	-0.73753
C	-0.00061	0.00012	1.04625
H	-0.00080	-0.00019	2.14206

Table S10, R'=CH cage complex, **R**=$FeBr_2$(THF)
Level of theory: B3LYP-GD3BJ/6-311++G**
Energy: -5132654.7661781 Kcal/mol

Si	0.36481	0.26216	0.07046
N	1.06826	-0.85202	1.33591
C	0.58280	-1.44969	2.48861
C	2.35703	-1.34578	1.12323
C	1.58412	-2.33564	2.98582
C	2.71343	-2.24767	2.09317
N	0.89268	-0.79771	-1.32798

C	0.26564	-1.34376	-2.43754
C	2.19757	-1.29543	-1.30244
C	1.19325	-2.20737	-3.09301
C	2.42616	-2.15432	-2.34739
N	1.84500	1.29866	-0.01577
C	2.07211	2.66371	-0.00891
C	3.08564	0.66418	-0.10096
C	3.47910	2.87834	-0.08378
C	4.10653	1.57962	-0.14297
C	-0.63386	-1.28178	3.15111
C	1.33389	-3.07431	4.15021
C	0.11293	-2.92090	4.79746
C	-0.85608	-2.02897	4.30342
H	-1.37427	-0.57141	2.80579
H	2.08409	-3.75341	4.54275
H	-0.09299	-3.48537	5.70067
H	-1.79244	-1.90611	4.83637
C	0.79553	-2.89986	-4.24473
C	-1.02341	-1.15020	-2.93704
C	-0.49632	-2.72274	-4.72710
C	-1.39141	-1.85109	-4.08084
H	1.48796	-3.56166	-4.75513
H	-0.81592	-3.25063	-5.61925
H	-2.38496	-1.70510	-4.49067
H	-1.70770	-0.45500	-2.47074
C	1.16987	3.72658	0.04629
C	1.68786	5.01683	0.03568
C	3.07408	5.24860	-0.03085
C	3.97297	4.18981	-0.09250
H	0.10100	3.55446	0.08358
H	1.00702	5.85961	0.07539
H	3.44263	6.26879	-0.03764
H	5.04070	4.37649	-0.14841
C	5.57601	1.31738	-0.23381
H	5.78993	0.24668	-0.27656
H	6.01143	1.77677	-1.12891
H	6.11178	1.72643	0.63081
C	3.68150	-2.90076	-2.66883
H	3.51690	-3.98471	-2.66445
H	4.06360	-2.63725	-3.66195
H	4.47116	-2.68584	-1.94507
C	3.99746	-3.00348	2.22107
H	4.69082	-2.75352	1.41456
H	4.50154	-2.78327	3.16934
H	3.83136	-4.08674	2.18911
C	3.04468	-0.84859	-0.12973
H	4.04888	-1.25868	-0.20470

Fe	-1.97112	1.04726	0.17931
Br	-2.60617	2.21341	-1.83356
Br	-2.98236	1.65003	2.28635
O	-2.83374	-0.87711	-0.11693
C	-2.26447	-2.19646	0.09231
C	-4.24394	-0.97484	-0.45267
C	-3.21964	-3.13545	-0.62774
H	-2.21780	-2.38856	1.16720
H	-1.25771	-2.19278	-0.31899
C	-4.57911	-2.46503	-0.37548
H	-4.36401	-0.56331	-1.45696
H	-4.79800	-0.35815	0.25608
H	-2.98488	-3.15244	-1.69486
H	-3.16512	-4.15437	-0.24042
H	-4.94768	-2.72143	0.62165
H	-5.33989	-2.74978	-1.10457

Table S10, R'=CH cage complex, **R**=Me
Level of theory: B3LYP-GD3BJ/6-311++G**
Energy: -988018.0999756 Kcal/mol

Si	-0.00041	0.00014	-1.22121
N	-1.53945	0.16205	-0.38959
C	-2.88741	0.30407	-0.69351
C	-1.41739	0.14923	1.01806
C	-3.60332	0.37942	0.53142
C	-2.64197	0.27818	1.60848
N	0.62885	-1.41379	-0.38976
C	1.18019	-2.65198	-0.69382
C	0.57920	-1.30176	1.01789
C	1.47324	-3.30966	0.53102
C	1.08022	-2.42664	1.60818
N	0.90939	1.25205	-0.38970
C	1.70659	2.34822	-0.69375
C	0.83760	1.15270	1.01797
C	2.13039	2.93027	0.53111
C	1.56219	2.14837	1.60826
C	-3.53296	0.37199	-1.92696
C	-4.99533	0.52592	0.50270
C	-5.64091	0.59385	-0.72614
C	-4.91662	0.51761	-1.92741
H	-2.98323	0.31413	-2.85898
H	-5.56167	0.58549	1.42538
H	-6.71797	0.70720	-0.76231
H	-5.44426	0.57312	-2.87234
C	2.04256	-4.58833	0.50214
C	1.44399	-3.24493	-1.92734

C	2.30638	-5.18131	-0.72676
C	2.00991	-4.51593	-1.92794
H	2.27431	-5.10860	1.42479
H	2.74687	-6.17069	-0.76304
H	2.22552	-5.00059	-2.87293
H	1.21891	-2.73997	-2.85931
C	2.08811	2.87324	-1.92725
C	2.90654	3.99835	-1.92786
C	3.33530	4.58721	-0.72666
C	2.95375	4.06218	0.50224
H	1.76266	2.42633	-2.85922
H	3.21838	4.42746	-2.87284
H	3.97238	5.46302	-0.76294
H	3.28902	4.52263	1.42486
C	1.75109	2.40657	3.06841
H	1.21873	1.67451	3.67900
H	2.80944	2.36251	3.34544
H	1.38413	3.39995	3.34648
C	1.21043	-2.71872	3.06837
H	0.64311	-3.61297	3.34634
H	2.25438	-2.89769	3.34569
H	0.84334	-1.89122	3.67882
C	-2.95984	0.31159	3.06868
H	-2.05932	0.21808	3.67896
H	-3.45253	1.24916	3.34615
H	-3.63500	-0.50431	3.34664
C	-0.00019	0.00004	1.54621
H	-0.00012	-0.00008	2.63271
C	-0.00038	0.00002	-3.06118
H	-0.60155	-0.82871	-3.44138
H	-0.41738	0.93495	-3.44166
H	1.01791	-0.10638	-3.44134

Table S10, skatole
Level of theory: B3LYP-GD3BJ/6-311++G**
Energy: -253060.4466367 Kcal/mol

N	-0.63582	1.81452	0.00000
C	0.40436	0.90927	0.00000
C	-1.83960	1.12467	-0.00000
C	-0.17330	-0.38929	-0.00000
C	-1.60560	-0.22498	0.00000
C	1.78594	1.10728	0.00000
C	2.59640	-0.02040	0.00000
C	2.04559	-1.31615	-0.00000
C	0.67162	-1.50894	-0.00000

H	2.21406	2.10344	0.00000
H	3.67349	0.09932	0.00000
H	2.70910	-2.17298	-0.00000
H	0.25639	-2.51057	-0.00000
C	-2.62148	-1.32257	0.00000
H	-3.63696	-0.92049	-0.00000
H	-2.51610	-1.96408	-0.88116
H	-2.51610	-1.96407	0.88116
H	-2.77683	1.65928	-0.00000
H	-0.53975	2.81513	0.00000

Table S12, **A**
Level of theory: B3LYP-GD3BJ/6-311++G**
Energy: -940170.2854508 Kcal/mol

Si	-0.00011	0.00004	-0.73032
N	-1.62566	-0.11200	-0.11503
C	-2.59681	-1.05109	-0.48367
C	-2.20337	0.71158	0.87071
C	-3.76670	-0.79751	0.27322
C	-3.48753	0.33379	1.12888
N	0.90979	-1.35185	-0.11531
C	2.20900	-1.72293	-0.48316
C	0.48463	-2.26507	0.86919
C	2.57383	-2.86346	0.27304
C	1.45383	-3.18832	1.12736
N	0.71566	1.46401	-0.11524
C	0.38654	2.77466	-0.48243
C	1.72070	1.55251	0.86756
C	1.19290	3.66097	0.27255
C	2.03584	2.85354	1.12538
C	-2.51993	-2.10093	-1.39809
C	-4.89076	-1.61331	0.09039
C	-4.82264	-2.65390	-0.82722
C	-3.64934	-2.89569	-1.56220
H	-1.61477	-2.30421	-1.95877
H	-5.79744	-1.43738	0.65813
H	-5.68366	-3.29425	-0.97867
H	-3.62115	-3.71877	-2.26626
C	3.84246	-3.42894	0.09067
C	3.08042	-1.13078	-1.39653
C	4.71022	-2.84902	-0.82593
C	4.33347	-1.71144	-1.56030
H	4.14295	-4.30262	0.65791

H	5.69537	-3.27442	-0.97705
H	5.03265	-1.27499	-2.26358
H	2.80425	-0.24483	-1.95673
C	-0.56328	3.23307	-1.39454
C	-0.68721	4.60856	-1.55833
C	0.11053	5.50373	-0.82513
C	1.04791	5.04235	0.09025
H	-1.19317	2.55080	-1.95376
H	-1.41572	4.99571	-2.26072
H	-0.01385	6.56957	-0.97626
H	1.65502	5.73953	0.65656
C	3.05565	3.36267	2.09311
H	3.55355	2.53977	2.60960
H	3.82394	3.95444	1.58491
H	2.59828	4.00807	2.85016
C	1.38595	-4.32470	2.09679
H	1.51411	-5.28664	1.58986
H	2.17432	-4.24999	2.85293
H	0.42487	-4.34399	2.61428
C	-4.43727	0.96011	2.09916
H	-3.97298	1.80151	2.61715
H	-5.33437	1.33085	1.59271
H	-4.76688	0.23951	2.85476
H	-0.00038	-0.00009	-2.20260
H	2.13526	0.65035	1.28908
H	-0.50346	-2.17208	1.29161
H	-1.62797	1.51975	1.29398

Table S12, **[A-H$^+$]$^-$ (relaxed)**
Level of theory: B3LYP-GD3BJ/6-311++G**
Energy: -939823.4960701 Kcal/mol

Si	0.00014	-0.00018	-1.30352
N	1.58690	-0.01162	-0.30862
C	2.70126	-0.77947	-0.60743
C	1.89583	0.70564	0.83900
C	3.71006	-0.53504	0.37266
C	3.16474	0.42668	1.28973
N	-0.78354	1.37974	-0.30870
C	-0.67592	2.72870	-0.60757
C	-1.55848	1.28865	0.83941
C	-1.39117	3.48019	0.37308
C	-1.95084	2.52709	1.29049
N	-0.80302	-1.36928	-0.30932

C	-2.02575	-1.94943	-0.60766
C	-0.33627	-1.99605	0.83799
C	-2.31885	-2.94521	0.37240
C	-1.21292	-2.95482	1.28898
C	2.90967	-1.68947	-1.64887
C	4.93638	-1.21117	0.28520
C	5.14044	-2.10891	-0.75531
C	4.13453	-2.34353	-1.71324
H	2.13564	-1.87438	-2.38414
H	5.71417	-1.03764	1.02256
H	6.08345	-2.64009	-0.83288
H	4.31730	-3.05172	-2.51442
C	-1.41847	4.88029	0.28576
C	0.00728	3.36406	-1.64954
C	-0.74351	5.50578	-0.75511
C	-0.03823	4.75187	-1.71362
H	-1.95706	5.46722	1.02348
H	-0.75476	6.58804	-0.83261
H	0.48327	5.26420	-2.51510
H	0.55374	2.78606	-2.38521
C	-2.91833	-1.67385	-1.64855
C	-4.09798	-2.40636	-1.71235
C	-4.39810	-3.39481	-0.75453
C	-3.51825	-3.66810	0.28538
H	-2.69117	-0.91113	-2.38379
H	-4.80295	-2.20968	-2.51307
H	-5.33017	-3.94500	-0.83174
H	-3.75715	-4.42851	1.02259
C	-1.05021	-3.84100	2.48392
H	-0.11265	-3.62601	3.00305
H	-1.86759	-3.70797	3.20330
H	-1.03964	-4.90194	2.20511
C	-2.79862	2.82909	2.48621
H	-2.27432	3.47073	3.20505
H	-3.72310	3.35007	2.20809
H	-3.08043	1.90965	3.00576
C	3.85062	1.01075	2.48478
H	3.19513	1.71423	3.00456
H	4.14523	0.23632	3.20365
H	4.76347	1.55147	2.20587
H	0.61555	-1.70665	1.25514
H	-1.78250	0.31955	1.25696
H	1.16875	1.38451	1.25638

Table S12, **B**
Level of theory: B3LYP-GD3BJ/6-311++G**
Energy: -1145787.1183781 Kcal/mol

Si	0.00000	-0.00076	-1.17326
N	-1.37925	-0.79718	-0.44322
C	-2.49885	-1.44205	-0.95521
C	-1.45349	-0.84018	0.98231
C	-3.28083	-1.89285	0.13783
C	-2.60033	-1.50100	1.34928
N	1.37925	-0.79716	-0.44322
C	2.49887	-1.44202	-0.95521
C	1.45350	-0.84016	0.98231
C	3.28085	-1.89281	0.13783
C	2.60035	-1.50096	1.34928
N	-0.00001	1.59167	-0.44271
C	-0.00002	2.88381	-0.95441
C	-0.00001	1.67714	0.98281
C	-0.00002	3.78622	0.13879
C	-0.00002	3.00068	1.35007
C	-2.88652	-1.66494	-2.27736
C	-4.47640	-2.58148	-0.10918
C	-4.86451	-2.80449	-1.42308
C	-4.07594	-2.34991	-2.49527
H	-2.28662	-1.31916	-3.11110
H	-5.08839	-2.93432	0.71336
H	-5.78616	-3.33525	-1.63046
H	-4.40159	-2.53709	-3.51181
C	4.47644	-2.58142	-0.10917
C	2.88655	-1.66490	-2.27736
C	4.86455	-2.80443	-1.42308
C	4.07597	-2.34986	-2.49527
H	5.08843	-2.93425	0.71336
H	5.78620	-3.33517	-1.63046
H	4.40163	-2.53703	-3.51181
H	2.28664	-1.31913	-3.11110
C	-0.00002	3.33114	-2.27649
C	-0.00003	4.70371	-2.49423
C	-0.00004	5.61376	-1.42190
C	-0.00003	5.16597	-0.10805
H	-0.00002	2.63877	-3.11028
H	-0.00004	5.07948	-3.51071
H	-0.00005	6.67734	-1.62911
H	-0.00004	5.87232	0.71453
C	-0.00002	3.57125	2.73310

H	-0.00001	2.79056	3.49387
H	0.88060	4.20038	2.89996
H	-0.88065	4.20037	2.89996
C	3.09502	-1.78605	2.73213
H	3.20093	-2.86318	2.89862
H	4.07964	-1.33679	2.89896
H	2.41852	-1.39680	3.49311
C	-3.09499	-1.78610	2.73213
H	-2.41850	-1.39683	3.49311
H	-4.07962	-1.33685	2.89896
H	-3.20089	-2.86323	2.89862
C	-0.00000	-0.00159	3.69734
H	-0.88520	0.50938	4.08184
H	0.00001	-1.02378	4.08158
H	0.88520	0.50939	4.08184
H	0.00000	-0.00042	-2.63770
Si	-0.00000	-0.00131	1.83449

Table S12, **[B-H$^+$]$^-$ (relaxed)**
Level of theory: B3LYP-GD3BJ/6-311++G**
Energy: -1145449.3909182 Kcal/mol

Si	0.00000	-0.00044	-1.45335
N	-1.37096	-0.79295	-0.43864
C	-2.48642	-1.43649	-0.93753
C	-1.45122	-0.83951	0.96427
C	-3.28078	-1.89519	0.15550
C	-2.59936	-1.50212	1.35624
N	1.37165	-0.79177	-0.43864
C	2.48766	-1.43435	-0.93753
C	1.45195	-0.83826	0.96426
C	3.28242	-1.89236	0.15550
C	2.60065	-1.49988	1.35624
N	-0.00068	1.58288	-0.43822
C	-0.00124	2.87080	-0.93672
C	-0.00072	1.67526	0.96469
C	-0.00163	3.78780	0.15655
C	-0.00130	3.00077	1.35707
C	-2.88310	-1.66498	-2.26111
C	-4.47677	-2.58519	-0.09612
C	-4.86483	-2.80855	-1.41065
C	-4.07116	-2.35030	-2.48164
H	-2.27386	-1.31330	-3.08467
H	-5.09060	-2.93968	0.72634

H	-5.78706	-3.34049	-1.61993
H	-4.39502	-2.53676	-3.50031
C	4.47900	-2.58133	-0.09612
C	2.88452	-1.66251	-2.26111
C	4.86725	-2.80436	-1.41065
C	4.07318	-2.34680	-2.48165
H	5.09314	-2.93530	0.72633
H	5.78993	-3.33552	-1.61993
H	4.39720	-2.53300	-3.50032
H	2.27498	-1.31136	-3.08467
C	-0.00143	3.32887	-2.26018
C	-0.00201	4.70047	-2.48040
C	-0.00242	5.61667	-1.40919
C	-0.00223	5.16862	-0.09477
H	-0.00112	2.62556	-3.08386
H	-0.00217	5.07442	-3.49898
H	-0.00287	6.68137	-1.61820
H	-0.00254	5.87730	0.72783
C	-0.00154	3.55468	2.74836
H	-0.00120	2.76138	3.49711
H	0.87908	4.18282	2.93100
H	-0.88271	4.18206	2.93100
C	3.08088	-1.77674	2.74733
H	3.18465	-2.85347	2.92984
H	4.06483	-1.32728	2.92970
H	2.39386	-1.38046	3.49628
C	-3.07934	-1.77940	2.74734
H	-2.39267	-1.38253	3.49629
H	-4.06367	-1.33079	2.92970
H	-3.18219	-2.85622	2.92984
C	0.00000	-0.00105	3.66550
H	-0.88558	0.50975	4.05099
H	0.00044	-1.02334	4.05109
H	0.88515	0.51051	4.05099
Si	0.00000	-0.00102	1.79009

Table S12, **C**
Level of theory: B3LYP-GD3BJ/6-311++G**
Energy: -963322.4293312 Kcal/mol

C	-2.82143	0.69907	-0.80977
C	-3.51937	0.87146	0.41448
C	-4.87815	1.20750	0.38425
C	-5.50619	1.36311	-0.84569

C	-4.79807	1.18823	-2.04649
C	-3.44762	0.85421	-2.04443
C	-1.38515	0.34367	0.90440
C	-2.58109	0.63929	1.49254
H	-5.43233	1.34441	1.30603
H	-6.55745	1.62312	-0.88323
H	-5.31220	1.31560	-2.99182
H	-2.90686	0.72074	-2.97462
C	0.98883	1.02780	0.90459
C	1.84413	1.91431	1.49290
C	2.01615	2.09300	-0.80937
C	2.51517	2.61023	0.41500
C	2.46404	2.55764	-2.04391
C	3.48673	3.61784	0.38505
C	3.42971	3.55905	-2.04568
H	2.07739	2.15700	-2.97422
C	3.93593	4.08385	-0.84475
H	3.88288	4.02857	1.30696
H	3.79742	3.94054	-2.99091
H	4.68762	4.86343	-0.88209
C	0.39434	-1.36990	0.90458
C	0.73615	-2.55333	1.49295
C	0.80500	-2.79171	-0.80932
C	1.00450	-3.48213	0.41508
C	0.98404	-3.41177	-2.04384
C	1.39303	-4.82685	0.38515
C	1.37026	-4.74824	-2.04559
H	0.82938	-2.87690	-2.97416
C	1.57274	-5.44869	-0.84464
H	1.55150	-5.37505	1.30706
H	1.51733	-5.25730	-2.99081
H	1.87341	-6.48907	-0.88195
Si	-0.00050	0.00053	-1.31834
N	0.42861	-1.48902	-0.50503
N	1.07462	1.11721	-0.50503
N	-1.50518	0.37357	-0.50523
C	0.82538	-2.86138	2.95283
H	0.57263	-1.99276	3.56412
H	0.14293	-3.67160	3.22927
H	1.83576	-3.17940	3.22968
C	-2.89291	0.71589	2.95235
H	-3.25281	1.71217	3.22897
H	-3.67415	0.00039	3.22868
H	-2.01468	0.49962	3.56392

C	2.06711	2.14495	2.95276
H	1.84272	3.18026	3.22917
H	1.43753	1.49534	3.56406
H	3.10894	1.95365	3.22961
H	-0.00030	0.00045	-2.78252
C	-0.00079	0.00063	1.43196
H	-0.00082	0.00068	2.51840

Table S12, **[C-H$^+$]$^-$ (relaxed)**
Level of theory: B3LYP-GD3BJ/6-311++G**
Energy: -962992.4486890 Kcal/mol

Si	0.00072	0.00038	-1.58882
N	-0.54040	-1.42929	-0.49788
C	-1.01766	-2.68875	-0.79617
C	-0.49974	-1.32208	0.88686
C	-1.27860	-3.37809	0.42895
C	-0.93658	-2.47525	1.49722
N	1.50936	0.24652	-0.49792
C	2.83866	0.46325	-0.79627
C	1.39618	0.22825	0.88682
C	3.56608	0.58229	0.42883
C	2.61322	0.42704	1.49714
N	-0.96695	1.18370	-0.49786
C	-1.82015	2.22581	-0.79623
C	-0.89455	1.09482	0.88690
C	-2.28746	2.79587	0.42886
C	-1.67611	2.04871	1.49718
C	-1.24851	-3.29649	-2.03261
C	-1.77507	-4.68830	0.38848
C	-2.00314	-5.28910	-0.84513
C	-1.74153	-4.59749	-2.04298
H	-1.04682	-2.76282	-2.95397
H	-1.97939	-5.22824	1.30803
H	-2.38734	-6.30292	-0.88829
H	-1.92788	-5.08811	-2.99254
C	4.94892	0.80790	0.38832
C	3.48038	0.56740	-2.03274
C	5.58322	0.91089	-0.84530
C	4.85351	0.79136	-2.04314
H	5.51871	0.90108	1.30784
H	6.65327	1.08539	-0.88849
H	5.37153	0.87541	-2.99272
H	2.91744	0.47509	-2.95414
C	-2.23198	2.72879	-2.03271

C	-3.11366	3.80503	-2.04314
C	-3.58243	4.37695	-0.84532
C	-3.17536	3.87975	0.38832
H	-1.87042	2.28746	-2.95406
H	-3.44617	4.21103	-2.99273
H	-4.26953	5.21559	-0.88853
H	-3.54144	4.32624	1.30783
C	-1.87004	2.28388	2.96163
H	-1.29067	1.57592	3.55946
H	-1.55776	3.29375	3.25444
H	-2.92196	2.17616	3.25314
C	2.91379	0.47619	2.96163
H	3.62927	-0.30187	3.25460
H	3.34986	1.43944	3.25327
H	2.01029	0.33171	3.55917
C	-1.04438	-2.76011	2.96173
H	-0.72271	-1.90355	3.55933
H	-2.07513	-2.99564	3.25354
H	-0.42441	-3.61628	3.25454
C	0.00070	0.00034	1.42413
H	0.00072	0.00020	2.51190

Table S15, L=**I**

Level of theory: MPW1PW91 functional with SDD basis set (associated with ECP) for Ir and 6-311+G(d,p) for all other atoms; tight geometry optimization and ultrafine integration grid were employed.

Energy: -1636736.7252420 Kcal/mol

Ge	-1.69630	-0.36096	-0.00003
Ir	0.63353	0.30391	0.00002
C	2.47987	-0.58274	1.15953
C	1.67586	-1.66043	0.71794
H	1.17018	-2.37514	1.34830
C	1.67591	-1.66030	-0.71822
H	1.17026	-2.37488	-1.34875
C	2.94353	0.11157	0.00007
H	3.58443	0.98048	0.00018
C	2.47993	-0.58252	-1.15955
H	2.68492	-0.31878	-2.18527
H	2.68479	-0.31921	2.18531
C	-0.03932	1.97039	0.00011
O	-0.48958	3.05579	0.00017
C	-2.78858	0.22574	1.56015
H	-2.79819	1.31679	1.61931

H	-3.82131	-0.13192	1.48065
H	-2.35293	-0.15723	2.48658
C	-2.78857	0.22591	-1.56015
H	-3.82129	-0.13179	-1.48071
H	-2.79820	1.31697	-1.61917
H	-2.35289	-0.15693	-2.48662
C	-1.95656	-2.34296	-0.00014
H	-3.02481	-2.58571	-0.00016
H	-1.50062	-2.79336	-0.88572
H	-1.50063	-2.79346	0.88541

Table S15, L=**II**

Level of theory: MPW1PW91 functional with SDD basis set (associated with ECP) for Ir and 6-311+G(d,p) for all other atoms; tight geometry optimization and ultrafine integration grid were employed.

Energy: -514993.5822180 Kcal/mol

Si	-1.78818	-0.54085	-0.00003
Ir	0.39791	0.29514	0.00003
C	2.33582	-0.43468	1.15745
C	1.61081	-1.56881	0.71717
H	1.16429	-2.32098	1.34842
C	1.61084	-1.56862	-0.71760
H	1.16434	-2.32061	-1.34908
C	2.75390	0.28579	0.00006
H	3.32257	1.20375	0.00020
C	2.33586	-0.43436	-1.15754
H	2.51757	-0.15398	-2.18333
H	2.51750	-0.15459	2.18332
C	-0.43170	1.88907	0.00018
O	-0.98464	2.92607	0.00028
C	-2.86914	-0.06371	1.50357
H	-2.97839	1.02295	1.56699
H	-3.87145	-0.50699	1.44500
H	-2.39780	-0.39765	2.43299
C	-2.86907	-0.06362	-1.50366
H	-3.87139	-0.50690	-1.44516
H	-2.97832	1.02304	-1.56702
H	-2.39770	-0.39750	-2.43307
C	-1.85867	-2.45771	-0.00009
H	-2.89946	-2.80471	-0.00015
H	-1.36571	-2.87416	-0.88376
H	-1.36579	-2.87420	0.88360

Table S15, L=**III**

Level of theory: MPW1PW91 functional with SDD basis set (associated with ECP) for Ir and 6-311+G(d,p) for all other atoms; tight geometry optimization and ultrafine integration grid were employed.

Energy: -1646354.2302055 Kcal/mol

C	0.56405	3.38090	-0.87770
Si	-0.12332	2.05060	0.27338
C	-0.09060	2.77257	2.01658
Si	0.85877	-0.06567	0.00369
Si	-0.21382	-0.80080	-1.94957
C	-0.13053	0.36675	-3.43030
O	-1.79175	1.86459	-0.14154
C	-2.66608	2.96444	-0.26444
C	-3.61053	2.72198	-1.41434
O	-4.27910	1.49912	-1.18367
C	-5.22281	1.20159	-2.18678
O	-1.89471	-0.98696	-1.56602
C	-2.78654	-1.68019	-2.41051
C	-3.74303	-2.48915	-1.57126
O	-4.38007	-1.61565	-0.66026
C	-5.34903	-2.27093	0.12563
C	0.42035	-2.47368	-2.53665
Si	-0.29134	-1.27631	1.65812
C	0.42085	-0.96825	3.37593
O	-1.93013	-0.70787	1.66358
C	-2.82848	-1.07392	2.68764
C	-3.75449	0.07908	2.98052
O	-4.40327	0.44253	1.77959
C	-5.34975	1.46795	1.96556
C	-0.39344	-3.14150	1.41972
Li	-2.83937	0.05812	-0.04220
H	-6.16738	-2.65291	-0.49792
H	-5.73586	-1.54192	0.83426
H	-4.90983	-3.11242	0.67589
H	-4.88919	2.35924	2.41068
H	-6.16639	1.13714	2.62012
H	-5.74697	1.72287	0.98524
H	-6.02322	1.95214	-2.20805
H	-5.64135	0.22470	-1.95497
H	-4.75133	1.16797	-3.17695
H	-2.30222	-1.33620	3.61277
H	-3.41366	-1.94822	2.37876
H	-4.49155	-0.22001	3.74010
H	-3.18603	0.93457	3.36953

H	0.52958	3.07102	-1.92415
H	1.61092	3.56623	-0.62661
H	0.04053	4.33720	-0.78168
H	-0.52183	3.77847	2.04719
H	0.93791	2.84170	2.38041
H	-0.64510	2.14044	2.71333
H	0.24967	-3.26429	-1.80313
H	1.50312	-2.39099	-2.66422
H	-0.00304	-2.78336	-3.49709
H	-0.91725	-3.62057	2.25372
H	0.61000	-3.56921	1.35995
H	-0.91724	-3.39466	0.49519
H	-2.25853	-2.36264	-3.08604
H	-3.34659	-0.96749	-3.02765
H	-2.12537	3.89884	-0.45237
H	-3.23681	3.09118	0.66287
H	-4.48666	-2.97695	-2.21800
H	-3.20033	-3.26815	-1.01983
H	0.33049	0.07729	3.67918
H	1.48887	-1.19911	3.33167
H	-0.02373	-1.59948	4.15124
H	-4.33388	3.54733	-1.48500
H	-3.05289	2.66829	-2.35882
H	-0.59456	-0.07414	-4.31856
H	0.91442	0.57729	-3.67404
H	-0.62624	1.31726	-3.22148
Ir	3.19237	-0.19514	0.02319
C	2.96973	-1.98739	0.09716
C	4.76361	1.10319	1.16831
C	3.86291	1.97650	0.51321
C	3.99954	1.79516	-0.90675
C	4.96288	0.78709	-1.12486
C	5.40573	0.32541	0.15813
O	2.83229	-3.14614	0.14863
H	4.92065	1.02505	2.23196
H	3.23160	2.70383	0.99934
H	3.47747	2.34766	-1.67166
H	5.30166	0.42396	-2.08204
H	6.15767	-0.42991	0.32798

Table S15, L=**IV**

Level of theory: MPW1PW91 functional with SDD basis set (associated with ECP) for Ir and 6-311+G(d,p) for all other atoms; tight geometry optimization and ultrafine integration grid were employed.

Energy: -960387.8389362 Kcal/mol

Ir	-2.02833	-0.11895	-0.56951
C	-3.72783	-1.61284	-0.00950
C	-3.76581	-0.50013	0.87721
H	-3.64167	-0.54785	1.94782
C	-4.11686	0.67224	0.12706
H	-4.28173	1.66103	0.52436
C	-3.96441	-1.11292	-1.31776
H	-3.99127	-1.69975	-2.22301
C	-4.22672	0.29406	-1.22800
H	-4.47021	0.94575	-2.05223
H	-3.54234	-2.63995	0.25999
C	-0.77343	-0.14647	-1.88024
O	0.03470	-0.19525	-2.71871
Si	-0.29893	0.40533	0.80852
N	1.15015	-0.78673	0.96106
N	1.27273	1.17487	0.10561
C	1.23161	-2.24203	1.18595
C	1.05123	-3.00798	-0.12797
H	1.01774	-4.08303	0.06886
H	0.11833	-2.70956	-0.60963
H	1.87883	-2.81962	-0.81393
C	0.07361	-2.58041	2.12672
H	-0.88064	-2.31008	1.66831
H	0.06541	-3.65263	2.33381
H	0.17060	-2.04604	3.07435
C	2.54784	-2.63600	1.85944
H	3.40537	-2.49813	1.20146
H	2.70821	-2.05511	2.77104
H	2.50692	-3.69256	2.13519
C	1.68344	2.50047	-0.38660
C	2.16606	2.45254	-1.83877
H	1.42157	1.97841	-2.47890
H	2.33413	3.47157	-2.19664
H	3.10566	1.90789	-1.93482
C	0.43547	3.38118	-0.30308
H	0.66545	4.38516	-0.66555
H	-0.37462	2.96612	-0.90599
H	0.08494	3.46106	0.72879
C	2.77583	3.08697	0.51297

H	2.44708	3.11230	1.55414
H	3.69862	2.50804	0.45012
H	3.00257	4.10973	0.20144
C	1.94550	0.04161	0.27496
C	3.30423	-0.26107	-0.24226
C	4.42532	-0.10597	0.57179
C	3.45766	-0.70219	-1.55674
C	5.69065	-0.39057	0.07515
H	4.30702	0.23201	1.59447
C	4.72520	-0.99357	-2.04471
H	2.58595	-0.80537	-2.19217
C	5.84209	-0.83784	-1.23218
H	6.55826	-0.26593	0.71217
H	4.83853	-1.33845	-3.06560
H	6.82956	-1.06294	-1.61746
N	-0.50975	1.05474	2.39976
C	-1.72018	1.71643	2.82868
H	-2.13482	1.24878	3.73268
H	-1.53589	2.77520	3.06210
H	-2.46995	1.67420	2.04083
C	0.55986	1.17260	3.36375
H	0.83012	2.22311	3.54649
H	0.26692	0.73889	4.32987
H	1.44937	0.64484	3.01979

Table S15, L=**V**

Level of theory: MPW1PW91 functional with SDD basis set (associated with ECP) for Ir and 6-311+G(d,p) for all other atoms; tight geometry optimization and ultrafine integration grid were employed.

Energy: -1197709.2364486 Kcal/mol

Ir	-0.17088	1.56441	-1.36627
C	-0.54755	3.76516	-0.67110
C	0.58820	3.24334	0.00533
H	0.68464	3.10360	1.07066
C	1.62553	3.01040	-0.95887
H	2.62045	2.65617	-0.74273
C	-0.24084	3.77686	-2.06073
H	-0.90066	4.10371	-2.85024
C	1.11261	3.34102	-2.23197
H	1.63959	3.26320	-3.17028
H	-1.47576	4.07307	-0.21694
C	-1.08063	0.48579	-2.51346
O	-1.66223	-0.18384	-3.26522

N	1.25719	-1.44605	-0.06826
C	2.08204	-1.77903	-1.13651
C	1.60891	-2.29398	0.97239
C	2.93330	-2.84179	-0.73893
C	2.60848	-3.15388	0.62180
N	0.09057	0.13527	1.81031
C	1.19375	0.67506	2.44461
C	-0.93095	0.12466	2.74172
C	0.84354	0.99486	3.78271
C	-0.53124	0.62969	3.94873
N	-1.51931	-1.33913	0.07618
C	-2.85958	-0.98224	0.12981
C	-1.48700	-2.72459	0.07358
C	-3.64525	-2.16176	0.18004
C	-2.73799	-3.26874	0.13448
C	2.16650	-1.23350	-2.41804
C	3.87052	-3.35920	-1.64085
C	3.94747	-2.81830	-2.91241
C	3.10272	-1.76390	-3.29129
H	1.52602	-0.40758	-2.70125
H	4.52660	-4.17178	-1.34563
H	4.66738	-3.20913	-3.62375
H	3.18184	-1.35137	-4.29132
C	1.80638	1.54957	4.63450
C	2.48671	0.89155	1.96458
C	3.08430	1.77320	4.15097
C	3.41925	1.44370	2.82798
H	1.55474	1.79974	5.66019
H	3.83948	2.20333	4.80010
H	4.42935	1.61891	2.47450
H	2.75536	0.62567	0.94986
C	-3.45276	0.28089	0.15641
C	-4.83456	0.35003	0.23663
C	-5.62411	-0.80899	0.29043
C	-5.03934	-2.06328	0.26032
H	-2.84249	1.17325	0.08383
H	-5.31468	1.32252	0.24933
H	-6.70343	-0.71739	0.35114
H	-5.65228	-2.95819	0.29731
C	-3.08985	-4.71777	0.13398
H	-2.19125	-5.33698	0.09304
H	-3.64734	-5.00051	1.03389
H	-3.71380	-4.98192	-0.72689
C	-1.35402	0.76376	5.18530

H	-0.93326	0.18978	6.01842
H	-1.42150	1.80613	5.51593
H	-2.37129	0.40476	5.01629
C	3.25031	-4.18855	1.48238
H	2.80263	-4.20035	2.47824
H	3.14425	-5.19259	1.05701
H	4.32314	-4.00297	1.60367
H	-1.90323	-0.25626	2.46769
H	-0.54058	-3.23904	0.01332
H	1.10657	-2.20521	1.92375
Si	-0.08452	-0.22803	0.02866

Table S15, L=**VI**

Level of theory: MPW1PW91 functional with SDD basis set (associated with ECP) for Ir and 6-311+G(d,p) for all other atoms; tight geometry optimization and ultrafine integration grid were employed.

Energy: -2319442.3593199 Kcal/mol

Ir	-0.01247	1.93006	-1.06863
C	-0.23286	3.95485	0.05296
C	0.84377	3.22904	0.62601
H	0.90399	2.88097	1.64569
C	1.88452	3.09525	-0.35593
H	2.84275	2.62637	-0.20039
C	0.09802	4.18085	-1.31599
H	-0.51295	4.70522	-2.03494
C	1.42582	3.68347	-1.55293
H	1.96786	3.74168	-2.48377
H	-1.14333	4.24802	0.55015
C	-0.98432	1.21524	-2.43264
O	-1.60004	0.78435	-3.31842
N	1.24383	-1.53876	-0.34308
C	2.03619	-1.73355	-1.46054
C	1.54619	-2.55765	0.54019
C	2.83054	-2.89277	-1.25617
C	2.49627	-3.40435	0.03939
N	0.04903	-0.25561	1.89095
C	1.13087	0.11944	2.65682
C	-1.00842	-0.43653	2.75713
C	0.73446	0.16148	4.02095
C	-0.64954	-0.20232	4.05875
N	-1.64048	-1.28592	-0.22216
C	-2.95954	-0.88239	-0.13310
C	-1.65654	-2.64664	-0.45615

C	-3.79477	-2.01833	-0.30103
C	-2.93144	-3.14019	-0.51345
C	2.13229	-0.99175	-2.63918
C	3.72641	-3.30299	-2.25134
C	3.81707	-2.56589	-3.41918
C	3.02644	-1.42076	-3.60650
H	1.53454	-0.09903	-2.77689
H	4.34000	-4.18683	-2.10834
H	4.50540	-2.87277	-4.19959
H	3.11555	-0.85729	-4.52898
C	1.67472	0.50745	4.99955
C	2.44352	0.40268	2.27467
C	2.97191	0.80101	4.61445
C	3.35152	0.74574	3.26332
H	1.39113	0.54389	6.04662
H	3.70881	1.07120	5.36323
H	4.37664	0.96809	2.98824
H	2.74614	0.34111	1.23614
C	-3.49871	0.38480	0.09657
C	-4.87763	0.50343	0.15912
C	-5.71638	-0.61112	-0.00193
C	-5.18544	-1.86835	-0.23309
H	-2.85225	1.24919	0.19505
H	-5.31710	1.48040	0.32870
H	-6.79208	-0.48094	0.05184
H	-5.83784	-2.72641	-0.36043
C	-3.33923	-4.55332	-0.76033
H	-2.46436	-5.19480	-0.88596
H	-3.92986	-4.95685	0.06974
H	-3.95057	-4.64658	-1.66474
C	-1.51825	-0.31221	5.26621
H	-1.14002	-1.05841	5.97393
H	-1.58322	0.63850	5.80701
H	-2.53346	-0.60395	4.98943
C	3.08024	-4.60152	0.71001
H	2.63673	-4.75099	1.69664
H	2.91336	-5.51490	0.12831
H	4.16272	-4.50095	0.84601
H	-1.97394	-0.73102	2.37271
H	-0.72716	-3.18193	-0.57844
H	1.05252	-2.59594	1.49975
Ge	-0.08226	-0.18485	-0.03420

Table S15, L=**VII**

Level of theory: MPW1PW91 functional with SDD basis set (associated with ECP) for Ir and 6-311+G(d,p) for all other atoms; tight geometry optimization and ultrafine integration grid were employed.

Energy: -621503.4140036 Kcal/mol

P	1.38949	-0.20643	-0.01920
Ir	-0.83947	0.19722	-0.08434
C	-2.75603	-0.70345	-1.01935
C	-1.92301	-1.78312	-0.66394
H	-1.51221	-2.51680	-1.33943
C	-1.73997	-1.74788	0.76336
H	-1.18231	-2.46789	1.34320
C	-3.07492	0.01386	0.18337
H	-3.71652	0.87934	0.24453
C	-2.49918	-0.67549	1.29272
H	-2.60185	-0.41412	2.33326
H	-3.08621	-0.45222	-2.01453
C	-0.49844	1.96531	-0.37564
O	-0.29311	3.09284	-0.55380
C	2.40194	0.90738	-1.08805
C	1.80536	-1.92822	-0.57223
C	2.17825	-0.07601	1.64637
C	3.08759	-2.58042	-0.06166
H	3.20033	-3.57225	-0.50706
H	3.98145	-2.00761	-0.30900
H	3.06690	-2.71265	1.02156
C	2.00466	1.29430	2.28965
H	2.41439	1.29627	3.30239
H	2.52072	2.07501	1.72578
H	0.94793	1.56245	2.34262
C	3.91186	0.70821	-1.14047
H	4.18366	-0.24430	-1.59817
H	4.36852	1.49690	-1.74360
H	4.37078	0.75294	-0.15032
H	1.79096	-1.88934	-1.66581
H	0.94663	-2.53888	-0.28746
H	2.16432	1.92193	-0.75846
H	1.96093	0.81607	-2.08429
H	3.23683	-0.34123	1.57119
H	1.69768	-0.84301	2.26125

Table S15, L=**VIII**
Level of theory: MPW1PW91 functional with SDD basis set (associated with ECP) for Ir and 6-311+G(d,p) for all other atoms; tight geometry optimization and ultrafine integration grid were employed.
Energy: -1220871.6920352 Kcal/mol

N	1.07752	0.00006	-1.50400
C	0.93705	0.00011	-2.87585
C	2.44408	0.00005	-1.24592
C	2.22955	0.00012	-3.46656
C	3.18271	0.00008	-2.39848
N	0.90665	1.34112	0.78449
C	0.57610	2.56030	1.34548
C	2.28453	1.22949	0.84700
C	1.77276	3.19059	1.77866
C	2.86050	2.31684	1.44630
N	0.90665	-1.34119	0.78438
C	0.57609	-2.56040	1.34529
C	2.28452	-1.22956	0.84690
C	1.77275	-3.19072	1.77843
C	2.86050	-2.31695	1.44614
C	2.86251	-0.00001	0.20296
H	3.94718	-0.00002	0.28143
C	-0.20437	0.00016	-3.67333
C	2.35854	0.00017	-4.85920
C	1.21741	0.00021	-5.64458
C	-0.05298	0.00020	-5.05180
H	-1.18399	0.00015	-3.22384
H	3.34187	0.00018	-5.31852
H	1.30405	0.00025	-6.72573
H	-0.93650	0.00023	-5.68102
C	1.71198	4.44977	2.38563
C	-0.66149	3.18313	1.49980
C	0.47969	5.06212	2.54374
C	-0.69276	4.43213	2.10068
H	2.61849	4.94143	2.72427
H	0.41800	6.03888	3.01190
H	-1.64696	4.93141	2.23119
H	-1.56320	2.69324	1.15407
C	-0.66150	-3.18325	1.49955
C	-0.69277	-4.43229	2.10034
C	0.47968	-5.06230	2.54338
C	1.71197	-4.44994	2.38532
H	-1.56320	-2.69333	1.15383
H	-1.64697	-4.93158	2.23081

H	0.41798	-6.03909	3.01147
H	2.61847	-4.94163	2.72394
C	4.30622	-2.57524	1.70144
H	4.93183	-1.75650	1.34006
H	4.51117	-2.69450	2.77113
H	4.64582	-3.49109	1.20489
C	4.30623	2.57512	1.70161
H	4.64582	3.49099	1.20511
H	4.51119	2.69431	2.77130
H	4.93184	1.75639	1.34017
C	4.66606	0.00007	-2.54693
H	5.16911	0.00005	-1.57823
H	5.01509	-0.88122	-3.09633
H	5.01510	0.88137	-3.09630
Si	-0.02243	0.00000	-0.02824
Ir	-2.29786	-0.00000	0.07910
C	-4.09937	1.16123	-0.87363
C	-3.25441	0.72001	-1.91644
H	-2.73640	1.35816	-2.61477
C	-3.25441	-0.71985	-1.91651
H	-2.73638	-1.35793	-2.61489
C	-4.57989	0.00000	-0.19329
H	-5.24494	-0.00004	0.65719
C	-4.09936	-1.16116	-0.87373
H	-4.31675	-2.18662	-0.62059
H	-4.31677	2.18666	-0.62040
C	-1.99919	-0.00008	1.87422
O	-1.86379	-0.00012	3.02610

Table S15, L=**IX**

Level of theory: MPW1PW91 functional with SDD basis set (associated with ECP) for Ir and 6-311+G(d,p) for all other atoms; tight geometry optimization and ultrafine integration grid were employed.

Energy: -2342606.5090629 Kcal/mol

N	1.09810	-1.57128	0.00005
C	0.94842	-2.93765	0.00007
C	2.45852	-1.31102	0.00004
C	2.23950	-3.53397	0.00009
C	3.19511	-2.46871	0.00007
N	0.97483	0.79823	-1.38026
C	0.68737	1.39318	-2.58782
C	2.34675	0.80047	-1.23544
C	1.91244	1.78457	-3.19293

C	2.96801	1.39277	-2.30589
N	0.97483	0.79831	1.38022
C	0.68736	1.39332	2.58775
C	2.34675	0.80053	1.23541
C	1.91242	1.78473	3.19286
C	2.96800	1.39288	2.30584
C	2.89154	0.13420	0.00000
H	3.97802	0.18822	0.00001
C	-0.20000	-3.72641	0.00009
C	2.35741	-4.92815	0.00012
C	1.21052	-5.70525	0.00014
C	-0.05750	-5.10561	0.00012
H	-1.17847	-3.27025	0.00008
H	3.33693	-5.39570	0.00014
H	1.29022	-6.78694	0.00017
H	-0.94410	-5.73046	0.00013
C	1.89220	2.41801	-4.44096
C	-0.53609	1.61909	-3.21775
C	0.67508	2.64391	-5.06248
C	-0.52548	2.24517	-4.45431
H	2.81783	2.72638	-4.91681
H	0.64622	3.13377	-6.03006
H	-1.46635	2.43261	-4.96058
H	-1.46068	1.31020	-2.74519
C	-0.53610	1.61926	3.21765
C	-0.52550	2.24541	4.45418
C	0.67505	2.64417	5.06235
C	1.89217	2.41824	4.44085
H	-1.46070	1.31036	2.74510
H	-1.46638	2.43288	4.96044
H	0.64619	3.13408	6.02991
H	2.81780	2.72662	4.91669
C	4.42749	1.59508	2.53382
H	5.02342	1.19804	1.70948
H	4.67679	2.65744	2.63338
H	4.76549	1.09823	3.45019
C	4.42751	1.59496	-2.53387
H	4.76552	1.09806	-3.45020
H	4.67679	2.65732	-2.63349
H	5.02343	1.19799	-1.70949
C	4.67815	-2.62304	0.00007
H	5.18525	-1.65645	0.00004
H	5.02571	-3.17340	0.88145
H	5.02571	-3.17346	-0.88127

Ge	-0.07824	-0.03021	0.00000
Ir	-2.43463	0.10288	-0.00001
C	-4.18466	-0.88403	-1.16454
C	-3.33242	-1.91757	-0.72065
H	-2.80007	-2.60850	-1.35533
C	-3.33241	-1.91753	0.72074
H	-2.80005	-2.60843	1.35545
C	-4.66399	-0.19914	0.00001
H	-5.34535	0.63808	-0.00000
C	-4.18464	-0.88397	1.16459
H	-4.40759	-0.63579	2.18987
H	-4.40761	-0.63590	-2.18982
C	-2.18141	1.90746	-0.00007
O	-2.06923	3.06135	-0.00011

Table S15, L=**X**

Level of theory: MPW1PW91 functional with SDD basis set (associated with ECP) for Ir and 6-311+G(d,p) for all other atoms; tight geometry optimization and ultrafine integration grid were employed.

Energy: -755989.4349576 Kcal/mol

Ir	-1.33219	-0.02840	0.19890
C	-2.34968	1.00486	-1.62637
C	-2.13354	-0.38809	-1.92409
H	-1.52450	-0.76879	-2.72924
C	-2.93469	-1.16520	-1.05085
H	-3.02556	-2.23927	-1.04834
C	-3.24606	1.08274	-0.54333
H	-3.61194	1.98467	-0.07911
C	-3.57196	-0.26023	-0.15226
H	-4.25331	-0.53399	0.63860
H	-1.91986	1.83822	-2.15905
C	-1.01627	-0.13867	1.99824
O	-0.82769	-0.21179	3.13880
Si	0.89226	0.00982	-0.04461
N	2.05621	1.28314	-0.02176
N	2.05949	-1.23272	-0.31122
C	3.35480	0.78599	-0.47162
C	3.40981	-0.69920	-0.14550
H	4.17620	1.29716	0.03246
C	1.87551	-2.69838	-0.16899
C	1.77969	-3.07383	1.31513
H	2.66221	-2.73718	1.86526
H	0.89647	-2.61838	1.76774

H	1.70500	-4.15774	1.43762
C	0.59836	-3.12280	-0.89238
H	-0.27422	-2.61089	-0.48290
H	0.66912	-2.89112	-1.95832
H	0.45321	-4.20052	-0.78251
C	3.04946	-3.44608	-0.80927
H	3.98514	-3.30590	-0.26403
H	2.83145	-4.51593	-0.80973
H	3.19684	-3.13288	-1.84607
C	1.81997	2.74604	-0.07472
C	0.69297	3.12020	0.88689
H	-0.23440	2.60036	0.64179
H	0.96277	2.85886	1.91289
H	0.51409	4.19750	0.84092
C	1.44596	3.16182	-1.50318
H	2.22237	2.87249	-2.21623
H	0.50963	2.68441	-1.80022
H	1.31569	4.24507	-1.57326
C	3.07821	3.50467	0.36103
H	3.89753	3.40951	-0.35468
H	2.84189	4.56736	0.44431
H	3.42358	3.16005	1.33885
H	3.48041	0.93849	-1.55368
H	4.12055	-1.19044	-0.81189
H	3.76288	-0.84378	0.88594

Table S15, L=**XI**

Level of theory: MPW1PW91 functional with SDD basis set (associated with ECP) for Ir and 6-311+G(d,p) for all other atoms; tight geometry optimization and ultrafine integration grid were employed.

Energy: -1515745.0981199 Kcal/mol

Ir	-0.77056	-1.74620	-0.07817
C	-1.95930	-2.55608	1.71783
C	-2.71550	-1.52372	1.07050
H	-3.02680	-0.60097	1.53140
C	-3.09501	-1.98568	-0.22580
H	-3.70258	-1.45669	-0.94201
C	-1.81924	-3.61744	0.79345
H	-1.28762	-4.54044	0.96518
C	-2.51364	-3.26191	-0.40912
H	-2.59616	-3.87497	-1.29311
H	-1.57907	-2.53160	2.72621
C	0.69060	-2.27576	-1.04323

O	1.58058	-2.64917	-1.68149
Si	0.16789	0.28755	-0.05087
C	2.00718	0.66282	0.07716
H	2.01762	1.69946	0.44712
C	-0.70193	1.97032	-0.14317
H	0.06634	2.60855	-0.61169
Si	2.96192	0.89019	-1.57045
Si	2.92372	-0.28225	1.46453
Si	-2.18694	2.09525	-1.34533
Si	-0.95016	2.85416	1.53330
C	1.89855	-0.29974	3.04934
H	1.67885	0.69518	3.44217
H	0.95391	-0.82547	2.88734
H	2.44914	-0.84095	3.82528
C	3.33671	-2.07724	1.10336
H	3.94543	-2.45774	1.93008
H	2.42917	-2.68113	1.05258
H	3.89530	-2.22535	0.17979
C	4.52099	0.65551	1.83487
H	4.32360	1.70055	2.08989
H	5.03093	0.19928	2.68868
H	5.21833	0.64357	0.99375
C	0.71708	3.40001	2.22625
H	1.38558	2.57144	2.46095
H	1.23504	4.06263	1.52663
H	0.55805	3.96433	3.15025
C	-1.81498	1.75056	2.78750
H	-1.29546	0.79938	2.92649
H	-1.85457	2.25309	3.75831
H	-2.84375	1.53642	2.48816
C	-1.93854	4.45420	1.37977
H	-2.95325	4.31812	1.00335
H	-2.01596	4.90235	2.37564
H	-1.43203	5.17805	0.73663
C	-3.84245	1.78701	-0.49720
H	-4.03767	2.47653	0.32627
H	-4.64705	1.91536	-1.22761
H	-3.91590	0.77072	-0.10750
C	-2.19488	3.83185	-2.08506
H	-2.98044	3.90176	-2.84366
H	-2.37413	4.61636	-1.34908
H	-1.24496	4.04829	-2.58283
C	-2.06146	0.91331	-2.80331
H	-1.20090	1.14292	-3.43596

H	-1.97161	-0.12686	-2.48493
H	-2.95884	1.01489	-3.42237
C	1.78491	0.96232	-3.03982
H	1.20516	0.04236	-3.14946
H	1.08659	1.79973	-2.96301
H	2.35826	1.10100	-3.96151
C	3.84657	2.55281	-1.46545
H	4.54833	2.58512	-0.62842
H	4.41228	2.74592	-2.38165
H	3.13710	3.37547	-1.33671
C	4.23972	-0.43833	-1.93686
H	4.73988	-0.18354	-2.87664
H	5.00978	-0.50969	-1.16595
H	3.78048	-1.42025	-2.05959

Table S15, L=**XII**

Level of theory: MPW1PW91 functional with SDD basis set (associated with ECP) for Ir and 6-311+G(d,p) for all other atoms; tight geometry optimization and ultrafine integration grid were employed.

Energy: -908435.0110042 Kcal/mol

P	-0.62643	0.02363	-0.00729
C	-0.99647	1.66486	0.73888
C	-0.74205	2.81977	-0.00637
C	-1.45643	1.79185	2.04850
C	-0.94384	4.07547	0.54920
H	-0.39399	2.73462	-1.02964
C	-1.65458	3.05178	2.60451
H	-1.66527	0.90684	2.63726
C	-1.39888	4.19473	1.85870
H	-0.74769	4.96296	-0.04147
H	-2.01431	3.13633	3.62348
H	-1.55603	5.17525	2.29275
C	-1.61223	-1.14486	1.01963
C	-0.98250	-1.73926	2.11509
C	-2.95283	-1.43784	0.76595
C	-1.68702	-2.59287	2.95461
H	0.06970	-1.53722	2.28463
C	-3.65217	-2.30069	1.60108
H	-3.45294	-1.00838	-0.09279
C	-3.02356	-2.87483	2.69943
H	-1.18572	-3.04919	3.80030
H	-4.68998	-2.52918	1.38755
H	-3.57090	-3.54992	3.34717

C	-1.55902	0.10937	-1.59379
C	-1.06258	-0.57800	-2.70057
C	-2.75228	0.82738	-1.71800
C	-1.75564	-0.56613	-3.90522
H	-0.11826	-1.10299	-2.61203
C	-3.43936	0.84475	-2.92528
H	-3.14041	1.38670	-0.87429
C	-2.94414	0.14415	-4.01969
H	-1.35973	-1.10578	-4.75770
H	-4.36042	1.40972	-3.01198
H	-3.48030	0.15973	-4.96161
Ir	1.58932	-0.40236	-0.11677
C	3.46690	0.54007	-1.10859
C	2.65662	1.59650	-0.64670
H	2.21589	2.37123	-1.25354
C	2.54767	1.48469	0.78429
H	2.00077	2.16051	1.42336
C	3.83094	-0.25243	0.03232
H	4.46507	-1.12543	0.01152
C	3.31864	0.37797	1.21003
H	3.46893	0.05301	2.22666
H	3.74704	0.34748	-2.13179
C	1.23052	-2.17162	-0.41673
O	1.02162	-3.29473	-0.60187

Table S15, L=**XIII**

Level of theory: MPW1PW91 functional with SDD basis set (associated with ECP) for Ir and 6-311+G(d,p) for all other atoms; tight geometry optimization and ultrafine integration grid were employed.

Energy: -1563549.2620193 Kcal/mol

Ir	1.02974	1.74745	-0.15627
C	2.67913	2.80117	1.07181
C	3.09739	1.47092	0.79998
H	3.31651	0.71891	1.53981
C	3.25554	1.32872	-0.61595
H	3.59726	0.44741	-1.13247
C	2.54287	3.46549	-0.18205
H	2.23226	4.48995	-0.31990
C	2.92387	2.56451	-1.22349
H	2.95262	2.78497	-2.27844
H	2.50052	3.22858	2.04511
C	-0.53569	2.61436	-0.51757
O	-1.47128	3.24369	-0.77321

Si	-0.14429	-0.15761	0.02875
C	-0.90018	-2.68352	0.43551
C	-2.04094	-1.98330	0.42501
H	-0.91858	-3.75571	0.61329
H	-2.98742	-2.48784	0.59344
C	0.40510	-1.99269	0.12667
C	-2.01348	-0.48927	0.20853
Si	1.71810	-2.37397	1.48776
Si	0.96062	-2.74753	-1.57072
Si	-2.80084	0.22913	1.82525
Si	-3.07206	-0.03173	-1.36260
C	1.59327	-1.24257	2.98948
H	1.53182	-0.18388	2.72972
H	0.72328	-1.48951	3.59915
H	2.48107	-1.38870	3.61331
C	3.49515	-2.24665	0.86243
H	3.73143	-1.26019	0.46237
H	4.16660	-2.42532	1.70816
H	3.73799	-2.98350	0.09574
C	1.45729	-4.11992	2.15149
H	2.21692	-4.32276	2.91293
H	0.48094	-4.21764	2.63244
H	1.53947	-4.89438	1.38803
C	2.24464	-1.71015	-2.47169
H	3.20516	-1.70191	-1.95381
H	2.40987	-2.13698	-3.46559
H	1.91130	-0.67740	-2.59598
C	1.63519	-4.50127	-1.36457
H	1.85140	-4.89249	-2.36406
H	2.55254	-4.57485	-0.78014
H	0.89155	-5.16444	-0.91534
C	-0.51894	-2.98549	-2.71047
H	-1.21554	-3.71732	-2.29636
H	-1.07821	-2.07448	-2.91677
H	-0.15740	-3.37440	-3.66778
C	-2.86388	2.10063	1.95586
H	-3.32432	2.34616	2.91859
H	-1.85785	2.52448	1.95346
H	-3.44143	2.58990	1.17378
C	-4.54139	-0.48640	2.01329
H	-5.02890	-0.00629	2.86744
H	-5.18253	-0.33522	1.14365
H	-4.50701	-1.55844	2.22308
C	-1.84396	-0.35679	3.33484

H	-2.41989	-0.11419	4.23340
H	-1.67820	-1.43595	3.32580
H	-0.87875	0.14536	3.41902
C	-4.29412	1.38798	-1.14756
H	-4.86887	1.45002	-2.07805
H	-5.00821	1.25389	-0.33429
H	-3.78871	2.34469	-1.01509
C	-2.02619	0.48436	-2.85039
H	-1.08724	-0.05604	-2.97148
H	-2.61769	0.33123	-3.75910
H	-1.78046	1.54577	-2.79275
C	-4.09112	-1.54814	-1.83102
H	-4.78569	-1.83157	-1.03598
H	-4.68588	-1.31513	-2.71946
H	-3.46982	-2.41574	-2.05931

Table S15, L=**XIV**

Level of theory: MPW1PW91 functional with SDD basis set (associated with ECP) for Ir and 6-311+G(d,p) for all other atoms; tight geometry optimization and ultrafine integration grid were employed.

Energy: -725696.2988570 Kcal/mol

P	-1.21649	-0.08960	-0.00602
Ir	1.00934	0.24627	-0.02624
C	2.79375	-0.59219	1.20586
C	1.98931	-1.68544	0.81363
H	1.49286	-2.37303	1.47978
C	2.01253	-1.77366	-0.62273
H	1.52784	-2.53152	-1.21723
C	3.26355	0.03712	0.01018
H	3.91501	0.89651	-0.03217
C	2.82684	-0.73273	-1.11553
H	3.05958	-0.53744	-2.14998
H	3.00157	-0.27918	2.21622
C	0.69918	2.03815	-0.19254
O	0.52046	3.17667	-0.29935
C	-1.85926	-1.79699	2.00588
H	-2.52811	-2.63435	2.22685
H	-0.87549	-2.02247	2.44295
H	-2.25721	-0.91194	2.49931
C	-1.42074	-2.80279	-0.14109
H	-0.46934	-3.21516	0.22027
H	-2.19086	-3.57045	-0.00949
H	-1.32211	-2.59594	-1.20629

C	-1.41557	0.62519	-2.64014
H	-0.38372	0.90284	-2.42749
H	-1.42645	0.00386	-3.54308
H	-1.99774	1.53327	-2.84298
C	-1.48479	1.64473	2.12616
H	-0.41715	1.43260	2.17019
H	-1.62596	2.73056	2.10238
H	-1.95847	1.25917	3.04023
C	-3.24518	-0.73069	-1.82040
H	-3.14365	-1.44951	-2.64351
H	-3.62853	-1.25765	-0.94897
H	-3.98039	0.02233	-2.13411
C	-3.49456	1.19844	0.85490
H	-4.02986	0.58112	1.59115
H	-3.75227	2.24511	1.04468
H	-3.85931	0.94606	-0.13708
N	-2.05707	1.04290	0.93547
N	-1.95949	-0.12708	-1.52634
N	-1.80863	-1.59896	0.57077

Table S15, L=**XV**
Level of theory: MPW1PW91 functional with SDD basis set (associated with ECP) for Ir and 6-311+G(d,p) for all other atoms; tight geometry optimization and ultrafine integration grid were employed.
Energy: -946534.5709042 Kcal/mol

Ir	-0.22978	1.62061	0.00001
C	-2.08112	2.39287	-1.16303
C	-2.49955	1.67029	0.00001
H	-3.11510	0.78404	-0.00000
C	-2.08113	2.39285	1.16306
H	-2.28979	2.12851	2.18694
C	-1.32424	3.50156	-0.72051
H	-0.85437	4.24188	-1.34904
C	-1.32424	3.50154	0.72057
H	-0.85437	4.24185	1.34911
H	-2.28978	2.12856	-2.18691
C	1.57963	1.95399	0.00003
O	2.70107	2.22336	0.00004
Si	0.18643	-0.55849	-0.00001
N	-0.86193	-1.95164	-0.00002
N	1.57141	-1.61160	-0.00002
C	-0.13650	-3.14714	-0.00002
C	1.19542	-2.95876	-0.00002

H	-0.65323	-4.09483	-0.00003
H	1.95405	-3.72656	-0.00003
C	-2.28687	-1.96814	-0.00001
C	-2.97083	-1.97273	-1.22323
C	-2.97083	-1.97276	1.22320
C	-4.36467	-1.98060	-1.20193
C	-4.36467	-1.98062	1.20191
C	-5.05841	-1.98333	-0.00001
H	-4.90767	-1.98477	-2.14073
H	-4.90766	-1.98482	2.14071
H	-6.14228	-1.98926	-0.00001
C	2.95864	-1.26792	-0.00001
C	3.62251	-1.11310	1.22313
C	3.62249	-1.11301	-1.22316
C	4.98133	-0.80674	1.20148
C	4.98131	-0.80665	-1.20150
C	5.65754	-0.65546	-0.00001
H	5.50969	-0.68223	2.14027
H	5.50966	-0.68208	-2.14029
H	6.71443	-0.41509	-0.00001
C	-2.22041	-1.95463	-2.52287
H	-1.63901	-1.03440	-2.62913
H	-1.51287	-2.78424	-2.58891
H	-2.90840	-2.02223	-3.36628
C	-2.22040	-1.95469	2.52284
H	-1.51286	-2.78430	2.58886
H	-1.63900	-1.03446	2.62912
H	-2.90839	-2.02230	3.36626
C	2.88651	-1.25347	-2.52372
H	2.34859	-2.20234	-2.58355
H	2.14609	-0.45704	-2.64236
H	3.57853	-1.19721	-3.36467
C	2.88654	-1.25364	2.52370
H	2.14611	-0.45725	2.64239
H	2.34865	-2.20253	2.58348
H	3.57857	-1.19740	3.36463

Table S15, L=**XVI**
Level of theory: MPW1PW91 functional with SDD basis set (associated with ECP) for Ir and 6-311+G(d,p) for all other atoms; tight geometry optimization and ultrafine integration grid were employed.
Energy: -689117.9345917 Kcal/mol

P	1.37336	-0.14871	-0.09235
Ir	-0.79053	0.20723	-0.05432
C	-2.72745	-0.57905	-1.05538
C	-1.86334	-1.67518	-0.87091
H	-1.43549	-2.28894	-1.64735
C	-1.63496	-1.82523	0.54195
H	-1.03853	-2.59982	0.99886
C	-3.02168	-0.03359	0.24229
H	-3.67438	0.80386	0.43538
C	-2.40105	-0.84983	1.23284
H	-2.48070	-0.73366	2.30131
H	-3.08660	-0.20028	-1.99881
C	-0.49091	2.01829	-0.19684
O	-0.33309	3.15694	-0.28023
C	2.86547	-2.39254	-0.44954
H	2.89079	-3.18096	-1.20041
H	3.84205	-1.90823	-0.39703
H	2.62704	-2.81554	0.52656
C	1.91166	0.35901	2.46004
H	2.30396	-0.18943	3.31538
H	2.41406	1.32692	2.38625
H	0.83599	0.51738	2.57244
C	3.64142	1.00454	-0.91504
H	3.90761	0.29279	-1.70011
H	3.95522	2.00257	-1.21593
H	4.13938	0.73144	0.01868
O	2.22938	1.05659	-0.75087
O	1.85000	-1.48434	-0.87121
O	2.17811	-0.43457	1.30683

Table S15, L=**XVII**
Level of theory: MPW1PW91 functional with SDD basis set (associated with ECP) for Ir and 6-311+G(d,p) for all other atoms; tight geometry optimization and ultrafine integration grid were employed.
Energy: -1230154.2214565 Kcal/mol

Ir	0.16604	-0.93540	-1.69148
C	0.34666	-3.21953	-1.99356
C	-0.80495	-2.95201	-1.21192

H	-0.98108	-3.28896	-0.20223
C	-1.74875	-2.22303	-2.01851
H	-2.73401	-1.91517	-1.70790
C	0.15681	-2.56814	-3.24917
H	0.85349	-2.57503	-4.07352
C	-1.16424	-2.00058	-3.27886
H	-1.61237	-1.48060	-4.11059
H	1.21315	-3.78386	-1.68955
C	1.33223	0.31120	-2.39094
O	2.05649	1.07153	-2.86123
N	-1.14891	1.38927	0.46358
C	-1.84925	2.15792	-0.48137
C	-1.53813	1.84475	1.73350
C	-2.66249	3.06645	0.22980
C	-2.44632	2.84616	1.63600
N	-0.22259	-0.70557	1.64001
C	-1.38604	-1.42405	1.91255
C	0.77138	-1.18565	2.49619
C	-1.09967	-2.32912	2.95833
C	0.28305	-2.15477	3.31502
N	1.48266	1.09254	0.63244
C	2.81158	0.64922	0.56852
C	1.51482	2.42523	1.07014
C	3.63806	1.71517	0.98184
C	2.78905	2.83513	1.28761
C	-1.84533	2.12807	-1.87318
C	-3.48594	3.95584	-0.46587
C	-3.48220	3.92832	-1.84884
C	-2.66957	3.02165	-2.53989
H	-1.22989	1.42129	-2.41187
H	-4.11506	4.65561	0.07240
H	-4.11217	4.61273	-2.40475
H	-2.68050	3.01560	-3.62360
C	-2.11318	-3.16084	3.44438
C	-2.65937	-1.32783	1.35451
C	-3.37530	-3.07816	2.88250
C	-3.64364	-2.16858	1.85051
H	-1.91294	-3.85793	4.25017
H	-4.17053	-3.71721	3.24781
H	-4.64346	-2.11297	1.43622
H	-2.88252	-0.61576	0.57087
C	3.34143	-0.59106	0.22051
C	4.71823	-0.74257	0.28035
C	5.55348	0.30756	0.68157

C	5.02317	1.53585	1.03380
H	2.70186	-1.40021	-0.10615
H	5.15483	-1.69592	0.00664
H	6.62550	0.15286	0.71552
H	5.66968	2.34765	1.34711
C	3.23027	4.18544	1.73765
H	2.37645	4.84594	1.89489
H	3.78857	4.12940	2.67683
H	3.88518	4.65511	0.99800
C	1.02173	-2.87727	4.38901
H	0.57124	-2.70317	5.37072
H	1.01590	-3.95778	4.21750
H	2.06210	-2.55280	4.43633
C	-3.11126	3.56630	2.75814
H	-2.75972	3.20102	3.72394
H	-2.91471	4.64149	2.71257
H	-4.19670	3.43415	2.72484
H	1.76523	-0.77036	2.45571
H	0.59895	2.98297	1.17112
H	-1.12473	1.38304	2.61502
P	0.07298	0.20707	0.18971

Table S15, L=**XVIII**
Level of theory: MPW1PW91 functional with SDD basis set (associated with ECP) for Ir and 6-311+G(d,p) for all other atoms; tight geometry optimization and ultrafine integration grid were employed.
Energy: -1253313.4287027 Kcal/mol

N	1.13945	0.00005	1.39897
C	1.01160	0.00013	2.78550
C	2.51544	0.00003	1.10491
C	2.31575	0.00015	3.33173
C	3.25653	0.00009	2.24405
N	0.85590	-1.28893	-0.80298
C	0.43613	-2.52779	-1.29684
C	2.24430	-1.21945	-0.96188
C	1.58383	-3.18121	-1.80189
C	2.72504	-2.33302	-1.57316
N	0.85593	1.28882	-0.80311
C	0.43618	2.52766	-1.29708
C	2.24432	1.21930	-0.96200
C	1.58390	3.18100	-1.80219
C	2.72509	2.33281	-1.57337
C	2.87337	-0.00005	-0.35822

H	3.95111	-0.00006	-0.49440
C	-0.10787	0.00017	3.60657
C	2.48620	0.00022	4.71806
C	1.36873	0.00027	5.53490
C	0.08435	0.00024	4.98010
H	-1.09943	0.00015	3.18914
H	3.48196	0.00024	5.14669
H	1.48613	0.00032	6.61193
H	-0.77991	0.00028	5.63408
C	1.46330	-4.45052	-2.37258
C	-0.81409	-3.13623	-1.32919
C	0.21715	-5.05084	-2.41838
C	-0.90529	-4.39902	-1.89539
H	2.33570	-4.95868	-2.76761
H	0.10799	-6.03606	-2.85615
H	-1.87065	-4.89057	-1.93027
H	-1.68194	-2.63928	-0.91895
C	-0.81403	3.13611	-1.32949
C	-0.90520	4.39886	-1.89581
C	0.21726	5.05061	-2.41885
C	1.46339	4.45027	-2.37299
H	-1.68189	2.63922	-0.91922
H	-1.87055	4.89042	-1.93074
H	0.10811	6.03580	-2.85670
H	2.33581	4.95838	-2.76806
C	4.13598	2.65076	-1.92972
H	4.81824	1.85174	-1.63567
H	4.24773	2.79859	-3.00793
H	4.47082	3.56992	-1.43978
C	4.13592	-2.65103	-1.92949
H	4.47072	-3.57019	-1.43953
H	4.24768	-2.79887	-3.00770
H	4.81821	-1.85202	-1.63544
C	4.74046	0.00009	2.37530
H	5.23447	0.00002	1.40283
H	5.08884	0.88114	2.92232
H	5.08883	-0.88089	2.92244
Ir	-2.15466	0.00002	0.01205
C	-3.16837	-0.72186	1.97617
C	-3.16835	0.72209	1.97610
H	-2.68669	1.35832	2.70191
C	-3.95802	1.16633	0.89814
H	-4.17534	2.19039	0.64088
C	-3.95805	-1.16618	0.89825

H	-4.17539	-2.19026	0.64109
C	-4.38712	0.00005	0.18230
H	-5.01635	0.00001	-0.69490
H	-2.68672	-1.35803	2.70203
C	-2.00846	-0.00007	-1.82770
O	-1.98493	-0.00013	-2.97584
P	0.05638	-0.00000	0.03473

Table S15, L=**XIX**

Level of theory: MPW1PW91 functional with SDD basis set (associated with ECP) for Ir and 6-311+G(d,p) for all other atoms; tight geometry optimization and ultrafine integration grid were employed.

Energy: -660371.4083410 Kcal/mol

P	1.72791	-0.40847	0.00307
F	2.31078	-1.27319	-1.19469
F	2.27875	-1.35877	1.15030
F	2.86727	0.68224	0.05452
Ir	-0.33896	0.18044	-0.00353
C	-1.97820	-0.88944	-1.21822
C	-1.43538	-1.77690	-0.24206
H	-0.93814	-2.71176	-0.45170
C	-1.77082	-1.28851	1.06902
H	-1.52996	-1.76661	2.00483
C	-2.54240	0.20249	-0.51083
H	-3.02817	1.05850	-0.95315
C	-2.43924	-0.06172	0.90515
H	-2.81177	0.57250	1.69384
H	-1.93591	-1.00952	-2.28817
C	0.15230	1.97233	0.00684
O	0.42461	3.08599	0.00867

Table S15, L=TSMPSi (**1**)

Level of theory: MPW1PW91 functional with SDD basis set (associated with ECP) for Ir and 6-311+G(d,p) for all other atoms; tight geometry optimization and ultrafine integration grid were employed.

Energy: -1435765.2776258 Kcal/mol

N	1.01104	0.00001	-1.54351
C	0.74778	0.00002	-2.89737
C	2.40584	-0.00001	-1.43123
C	1.96794	-0.00000	-3.61301
C	3.02936	-0.00002	-2.65881
N	0.81461	1.38734	0.77812
C	0.35098	2.60458	1.25390

C	2.19577	1.41035	0.97253
C	1.44452	3.34499	1.76387
C	2.62766	2.56990	1.57335
N	0.81457	-1.38736	0.77811
C	0.35090	-2.60458	1.25389
C	2.19574	-1.41042	0.97249
C	1.44443	-3.34504	1.76384
C	2.62760	-2.56999	1.57329
C	-0.47117	0.00007	-3.57761
C	1.96669	-0.00000	-5.01525
C	0.75770	0.00003	-5.67826
C	-0.45003	0.00007	-4.95823
H	-1.39738	0.00010	-3.02917
H	2.89966	-0.00003	-5.56761
H	0.73296	0.00004	-6.76137
H	-1.38891	0.00011	-5.50013
C	1.24707	4.62026	2.31366
C	-0.93834	3.14823	1.26500
C	-0.02797	5.14227	2.33215
C	-1.10516	4.40773	1.80523
H	2.08334	5.18532	2.70985
H	-0.20546	6.12638	2.74958
H	-2.09806	4.84272	1.82238
H	-1.76906	2.58472	0.86116
C	-0.93843	-3.14818	1.26502
C	-1.10529	-4.40768	1.80525
C	-0.02812	-5.14226	2.33214
C	1.24694	-4.62030	2.31363
H	-1.76914	-2.58464	0.86120
H	-2.09821	-4.84263	1.82243
H	-0.20564	-6.12636	2.74958
H	2.08319	-5.18539	2.70979
C	3.99532	-3.01275	1.96797
H	4.75492	-2.24752	1.82053
H	4.01601	-3.28926	3.02577
H	4.29938	-3.89748	1.40017
C	3.99539	3.01261	1.96804
H	4.29960	3.89719	1.40009
H	4.01602	3.28931	3.02580
H	4.75493	2.24726	1.82083
C	4.47543	-0.00007	-3.02237
H	5.13853	0.00025	-2.16035
H	4.72133	-0.87967	-3.62450
H	4.72123	0.87918	-3.62505

Si	-0.08970	0.00001	-0.04841
Ir	-2.35392	0.00003	0.10633
C	-3.45583	0.72057	-1.81578
C	-3.45577	-0.72055	-1.81579
H	-3.00659	-1.35898	-2.56019
C	-4.21423	-1.16189	-0.71044
H	-4.42272	-2.18640	-0.44664
C	-4.21431	1.16183	-0.71041
H	-4.42288	2.18630	-0.44659
C	-4.63503	-0.00006	0.00862
H	-5.23457	-0.00009	0.90624
H	-3.00671	1.35905	-2.56016
C	-1.99888	0.00008	1.89711
O	-1.81871	0.00011	3.03990
P	2.98771	-0.00004	0.25418
C	4.77701	-0.00005	0.41960
H	5.18269	0.89216	-0.05432
H	5.03008	-0.00012	1.47887
H	5.18269	-0.89221	-0.05444

Table S15, L=TSMPGe (**12**)

Level of theory: MPW1PW91 functional with SDD basis set (associated with ECP) for Ir and 6-311+G(d,p) for all other atoms; tight geometry optimization and ultrafine integration grid were employed.

Energy: -2557502.5408736 Kcal/mol

N	1.06528	0.12460	-1.59292
C	0.81369	0.25316	-2.93727
C	2.45200	0.08964	-1.45923
C	2.04230	0.29677	-3.63898
C	3.09197	0.18936	-2.67791
N	0.88693	1.34808	0.92562
C	0.46041	2.51105	1.53169
C	2.26949	1.32113	1.06885
C	1.58399	3.18671	2.06752
C	2.74491	2.41560	1.76114
N	0.85855	-1.50016	0.68849
C	0.40986	-2.73758	1.09911
C	2.23872	-1.51566	0.84945
C	1.51830	-3.50551	1.53331
C	2.69206	-2.71128	1.36737
C	-0.40173	0.33751	-3.62142
C	2.05041	0.42547	-5.03591
C	0.84656	0.50802	-5.70307

C	-0.36878	0.46414	-4.99556
H	-1.33541	0.30524	-3.08290
H	2.98668	0.46141	-5.58155
H	0.83090	0.60843	-6.78170
H	-1.30191	0.53177	-5.54309
C	1.41960	4.40503	2.74446
C	-0.82450	3.05451	1.64967
C	0.15102	4.92960	2.86368
C	-0.95649	4.25626	2.31509
H	2.27494	4.92482	3.16152
H	0.00013	5.86971	3.38103
H	-1.94305	4.69348	2.41878
H	-1.67903	2.53956	1.22959
C	-0.88347	-3.27348	1.11737
C	-1.03931	-4.56325	1.58250
C	0.05276	-5.33125	2.02825
C	1.32933	-4.81322	2.00676
H	-1.72562	-2.68446	0.77708
H	-2.03265	-4.99679	1.60603
H	-0.11649	-6.33929	2.38805
H	2.17253	-5.40592	2.34353
C	4.07111	-3.17343	1.69879
H	4.80062	-2.36633	1.72748
H	4.08317	-3.64980	2.68243
H	4.42398	-3.91877	0.97861
C	4.13120	2.80321	2.15077
H	4.41037	3.75812	1.69518
H	4.20265	2.93327	3.23450
H	4.88231	2.07073	1.86307
C	4.54236	0.20761	-3.02650
H	5.19026	-0.06992	-2.19822
H	4.74523	-0.49189	-3.84178
H	4.85100	1.19950	-3.37159
Ge	-0.14009	0.00593	-0.05534
Ir	-2.48664	0.00678	0.09739
C	-3.50276	0.89048	-1.80074
C	-3.49097	-0.54671	-1.92520
H	-3.00720	-1.11297	-2.70571
C	-4.27512	-1.08957	-0.88648
H	-4.48567	-2.13389	-0.72141
C	-4.29081	1.23240	-0.68319
H	-4.51560	2.22888	-0.33865
C	-4.71600	0.00566	-0.07275
H	-5.34730	-0.07490	0.79891

H	-3.02957	1.59087	-2.47101
C	-2.20117	-0.13271	1.89850
O	-2.06050	-0.22233	3.04255
P	3.01991	-0.04947	0.22763
C	4.81086	-0.08674	0.37764
H	5.22973	0.81604	-0.06327
H	5.07306	-0.13685	1.43329
H	5.19511	-0.96624	-0.13604

Table S16, **A**
Level of theory: B3LYP-GD3BJ/6-311++G**
Energy: -1250582.8988964 Kcal/mol

C	0.00127	2.81368	-1.08439
C	0.00161	3.81392	-0.06209
C	0.00221	5.17897	-0.39872
C	0.00244	5.53632	-1.73381
C	0.00208	4.54626	-2.73973
C	0.00149	3.19497	-2.43863
C	0.00065	1.79398	0.87664
C	0.00120	3.14016	1.19984
H	0.00249	5.93681	0.37723
H	0.00290	6.58254	-2.01629
H	0.00226	4.85194	-3.77991
H	0.00122	2.46187	-3.23131
P	0.00012	0.26196	1.75615
C	1.42622	-0.58829	1.10752
C	2.57674	-1.13205	1.64733
C	2.60105	-1.30373	-0.65130
C	3.34639	-1.58872	0.52460
C	3.09124	-1.63341	-1.92188
C	4.60153	-2.21322	0.42314
C	4.33060	-2.24748	-1.99203
H	2.50817	-1.42813	-2.80966
C	5.08278	-2.53648	-0.83316
H	5.18218	-2.43494	1.31157
H	4.73301	-2.51478	-2.96231
H	6.04865	-3.01718	-0.93417
C	-1.42654	-0.58727	1.10743
C	-2.57735	-1.13032	1.64737
C	-2.60227	-1.30137	-0.65130
C	-3.34761	-1.58606	0.52469
C	-3.09286	-1.63061	-1.92185
C	-4.60323	-2.20961	0.42332

C	-4.33267	-2.24376	-1.99189
H	-2.50976	-1.42568	-2.80968
C	-5.08490	-2.53234	-0.83295
H	-5.18394	-2.43101	1.31180
H	-4.73541	-2.51066	-2.96215
H	-6.05114	-3.01232	-0.93388
Si	-0.00006	-0.21969	-1.36387
N	-1.41921	-0.68182	-0.29225
N	1.41849	-0.68326	-0.29216
N	0.00069	1.56245	-0.49610
C	-3.01517	-1.24737	3.07317
H	-2.22979	-0.99150	3.78227
H	-3.87207	-0.59607	3.27604
H	-3.33193	-2.27103	3.29354
C	0.00153	3.81417	2.53515
H	0.88317	4.45301	2.65164
H	-0.87692	4.45773	2.64949
H	-0.00145	3.10742	3.36469
C	3.01477	-1.24950	3.07301
H	3.87686	-0.60439	3.27357
H	2.23211	-0.98574	3.78221
H	3.32382	-2.27509	3.29549
C	-0.00006	0.41845	3.55200
H	0.00045	-0.57132	4.00506
H	0.89051	0.96543	3.85769
H	-0.89130	0.96442	3.85755
O	-0.00100	-1.66541	-2.27346
C	-0.00173	-2.97892	-1.74429
H	0.89130	-3.51269	-2.08300
H	-0.00813	-2.98657	-0.64791
H	-0.88915	-3.51534	-2.09330
H	0.00071	0.52895	-2.62613

Table S16, **B**, *E*-form
Level of theory: B3LYP-GD3BJ/6-311++G**
Energy: -1250581.0055434 Kcal/mol

C	-4.32081	-0.14966	0.62619
C	-4.06925	-0.12226	-0.79768
C	-5.15331	-0.12518	-1.70440
C	-6.43837	-0.15631	-1.20442
C	-6.68275	-0.18467	0.19568
C	-5.64863	-0.18246	1.10778
C	-2.18763	-0.11257	0.37570

C	-2.66049	-0.09858	-0.95047
H	-4.98023	-0.10192	-2.77578
H	-7.28223	-0.15890	-1.88557
H	-7.70844	-0.20844	0.54770
H	-5.83585	-0.20479	2.17567
P	-0.52790	-0.04966	0.90669
C	0.31156	1.42768	0.33739
C	-0.21844	2.69551	0.27302
C	2.02323	2.77440	-0.26318
C	0.86284	3.56737	-0.11480
C	3.25337	3.32675	-0.62972
C	0.93420	4.94968	-0.34032
C	3.29723	4.69704	-0.85071
H	4.14493	2.71868	-0.72363
C	2.15125	5.50172	-0.70869
H	0.05394	5.57166	-0.22871
H	4.23682	5.15693	-1.13304
H	2.22733	6.56752	-0.88733
C	0.44091	-1.43909	0.31961
C	0.03230	-2.75096	0.25267
C	2.27175	-2.61829	-0.28132
C	1.19047	-3.51684	-0.13632
C	3.54855	-3.05239	-0.64775
C	1.39091	-4.88589	-0.36515
C	3.72045	-4.41198	-0.87184
H	4.37937	-2.36334	-0.73907
C	2.65459	-5.32075	-0.73318
H	0.57253	-5.58768	-0.25599
H	4.69922	-4.78131	-1.15405
H	2.83024	-6.37430	-0.91429
Si	2.73531	0.10632	-0.42360
N	1.81845	-1.32379	0.00009
N	1.69341	1.44238	0.01548
N	-3.16533	-0.14186	1.34275
C	-1.31742	-3.33234	0.53133
H	-1.25038	-4.08549	1.32275
H	-2.04651	-2.58002	0.82672
H	-1.70178	-3.83427	-0.36174
C	-1.86456	-0.03410	-2.21680
H	-0.88728	-0.51552	-2.10840
H	-2.38506	-0.54341	-3.03247
H	-1.68415	0.99821	-2.54188
C	-1.61711	3.14860	0.54965
H	-2.26412	2.34135	0.88777

H	-1.61636	3.93757	1.30808
H	-2.06154	3.57107	-0.35670
C	-0.49119	-0.06309	2.72104
H	0.54525	-0.02222	3.05829
H	-1.05336	0.79673	3.08343
H	-0.97812	-0.97341	3.06899
H	3.11591	0.13129	-1.83986
O	4.15550	0.16477	0.39397
C	4.25412	0.16197	1.82151
H	5.31191	0.20056	2.07827
H	3.74920	1.03524	2.24484
H	3.81644	-0.75053	2.23700

Table S16, **B**, *Z*-form
Level of theory: B3LYP-GD3BJ/6-311++G**
Energy: -1250581.6702956 Kcal/mol

C	-4.30692	-0.24825	0.40017
C	-3.88302	-0.17622	-0.98080
C	-4.84762	-0.17081	-2.01368
C	-6.18345	-0.23705	-1.67666
C	-6.59709	-0.30954	-0.31862
C	-5.68273	-0.31667	0.71353
C	-2.16087	-0.16780	0.41621
C	-2.46691	-0.12409	-0.95752
H	-4.54534	-0.11424	-3.05478
H	-6.93720	-0.23385	-2.45637
H	-7.65750	-0.36008	-0.09628
H	-5.99888	-0.37262	1.74929
P	-0.57731	-0.08128	1.14079
C	0.28124	1.41918	0.67031
C	-0.28356	2.66299	0.50580
C	1.99341	2.80251	0.16037
C	0.79788	3.55748	0.17850
C	3.22757	3.38618	-0.14090
C	0.83873	4.93065	-0.10494
C	3.24157	4.74740	-0.41383
H	4.13973	2.80400	-0.17332
C	2.06129	5.51420	-0.39663
H	-0.07005	5.52073	-0.09684
H	4.18366	5.22881	-0.64798
H	2.11425	6.57352	-0.61721
C	0.48138	-1.43718	0.63682
C	0.10213	-2.74766	0.45893

C	2.37644	-2.55560	0.12383
C	1.30178	-3.47454	0.12873
C	3.68253	-2.95289	-0.17784
C	1.54008	-4.82474	-0.16744
C	3.89263	-4.29520	-0.46349
H	4.50172	-2.24548	-0.20110
C	2.83473	-5.22376	-0.45878
H	0.72537	-5.53915	-0.16880
H	4.89479	-4.63394	-0.69813
H	3.04008	-6.26223	-0.68906
Si	2.85218	0.16874	0.44989
N	1.87738	-1.28625	0.43981
N	1.68315	1.47177	0.46526
N	-3.24827	-0.24103	1.25373
C	-1.25861	-3.35904	0.56979
H	-1.24741	-4.17601	1.29798
H	-2.02141	-2.64043	0.86370
H	-1.56022	-3.78523	-0.39186
C	-1.52092	-0.00586	-2.11210
H	-0.57435	-0.52073	-1.91724
H	-1.94964	-0.44880	-3.01497
H	-1.27933	1.03895	-2.34579
C	-1.71884	3.07072	0.61760
H	-2.35522	2.28141	1.01314
H	-1.81056	3.95058	1.26117
H	-2.11288	3.34354	-0.36634
C	-0.74939	-0.12166	2.94626
H	0.23888	-0.06997	3.40496
H	-1.36474	0.72336	3.25297
H	-1.25754	-1.04500	3.22237
O	3.80130	0.24751	-0.88173
C	3.29374	0.22084	-2.22291
H	2.62056	1.06464	-2.39635
H	4.14687	0.29611	-2.89545
H	2.76396	-0.71624	-2.41414
H	3.78223	0.22210	1.58388

Table S16, **C**
Level of theory: B3LYP-GD3BJ/6-311++G**
Energy: -1396513.9043289 Kcal/mol

C	-0.33907	2.75428	-0.00210
C	-0.08209	3.50887	1.18447
C	-0.33723	4.89106	1.21550

C	-0.82802	5.50639	0.07828
C	-1.06173	4.75952	-1.09649
C	-0.82486	3.39649	-1.15411
C	0.42452	1.36535	1.53707
C	0.42464	2.59940	2.16684
H	-0.14603	5.46617	2.11501
H	-1.02674	6.57175	0.08059
H	-1.42674	5.26915	-1.98134
H	-0.98732	2.84746	-2.06779
P	1.05517	-0.24488	1.92185
C	2.25404	-0.56745	0.65221
C	3.61983	-0.80160	0.62191
C	2.76668	-0.59330	-1.50707
C	3.96322	-0.82395	-0.76932
C	2.77740	-0.54381	-2.90936
C	5.17963	-1.01515	-1.44895
C	3.99018	-0.72962	-3.55017
H	1.86210	-0.37154	-3.45880
C	5.18332	-0.96552	-2.83126
H	6.09778	-1.19350	-0.89992
H	4.02654	-0.69436	-4.63317
H	6.11048	-1.10706	-3.37440
C	-0.31128	-1.31900	1.57461
C	-1.03607	-2.23268	2.31846
C	-1.86617	-2.05464	0.17593
C	-2.05299	-2.70780	1.42951
C	-2.71356	-2.35058	-0.90434
C	-3.09778	-3.63255	1.60250
C	-3.73625	-3.26254	-0.70686
H	-2.54697	-1.89569	-1.86653
C	-3.93678	-3.89936	0.53628
H	-3.24191	-4.12726	2.55678
H	-4.39359	-3.50328	-1.53508
H	-4.74862	-4.60848	0.64826
Si	-0.05806	-0.02931	-1.08794
N	-0.79648	-1.18022	0.27164
N	1.71497	-0.44473	-0.62879
N	-0.04976	1.42616	0.22935
C	-0.87641	-2.67601	3.73927
H	0.08936	-2.40558	4.16316
H	-1.65389	-2.24786	4.38205
H	-0.96597	-3.76371	3.81143
C	0.86814	2.98009	3.54501
H	1.85059	3.46583	3.53306

H	0.16650	3.69154	3.98962
H	0.92696	2.12609	4.21971
C	4.59593	-0.98335	1.74150
H	5.27580	-0.12754	1.81760
H	4.11313	-1.10950	2.70963
H	5.21552	-1.86894	1.57107
C	1.70591	-0.39231	3.59739
H	2.09481	-1.39799	3.74721
H	2.50472	0.33563	3.72994
H	0.90617	-0.19637	4.30958
O	-0.27402	-1.23670	-2.29099
C	0.16892	-2.57742	-2.22060
H	-0.61996	-3.24613	-2.57868
H	1.05472	-2.71241	-2.85011
H	0.42915	-2.87747	-1.20003
H	0.19947	1.00030	-2.11761
O	-2.08761	0.53331	-1.36633
C	-2.60632	0.72544	-2.70731
C	-3.06726	0.92546	-0.36111
C	-3.89424	1.51805	-2.51534
H	-1.84037	1.23335	-3.29228
H	-2.77783	-0.25860	-3.14736
C	-4.37253	1.04136	-1.13596
H	-3.07264	0.15880	0.40922
H	-2.75100	1.87814	0.06445
H	-4.61411	1.32564	-3.31213
H	-3.68219	2.58991	-2.49167
H	-4.85289	0.06265	-1.21248
H	-5.06839	1.73414	-0.66058

Table S16, **D**, *E*-form
Level of theory: B3LYP-GD3BJ/6-311++G**
Energy: -1396512.5825994 Kcal/mol

C	4.60885	0.00249	0.78863
C	4.38941	0.00277	-0.64144
C	5.49488	0.00357	-1.52163
C	6.76868	0.00407	-0.99211
C	6.98117	0.00380	0.41324
C	5.92522	0.00303	1.30061
C	2.48006	0.00151	0.49158
C	2.98381	0.00212	-0.82334
H	5.34813	0.00377	-2.59737
H	7.62775	0.00469	-1.65411

H	7.99860	0.00422	0.78940
H	6.08760	0.00282	2.37293
P	0.79540	0.00023	0.96751
C	-0.05901	-1.44277	0.32620
C	0.38060	-2.74134	0.45002
C	-1.58289	-2.70585	-0.75465
C	-0.59063	-3.56445	-0.22337
C	-2.66653	-3.19788	-1.48882
C	-0.69068	-4.94904	-0.42684
C	-2.74464	-4.57203	-1.67097
H	-3.40958	-2.53633	-1.91660
C	-1.76862	-5.44067	-1.14603
H	0.06294	-5.61906	-0.02989
H	-3.57238	-4.98378	-2.23624
H	-1.86326	-6.50683	-1.31380
C	-0.06117	1.44165	0.32548
C	0.37637	2.74096	0.44891
C	-1.58633	2.70182	-0.75694
C	-0.59573	3.56223	-0.22547
C	-2.67030	3.19180	-1.49199
C	-0.69782	4.94657	-0.42969
C	-2.75043	4.56573	-1.67488
H	-3.41206	2.52887	-1.91990
C	-1.77608	5.43616	-1.14977
H	0.05454	5.61796	-0.03264
H	-3.57847	4.97591	-2.24086
H	-1.87227	6.50208	-1.31815
Si	-2.23083	-0.00247	-0.86004
N	-1.26102	1.38687	-0.41732
N	-1.25943	-1.39022	-0.41587
N	3.43710	0.00169	1.47851
C	1.61479	3.24435	1.12054
H	1.36593	3.79731	2.03258
H	2.30555	2.44135	1.37855
H	2.14687	3.93389	0.45931
C	2.20408	0.00189	-2.10186
H	1.55704	0.88329	-2.18928
H	2.87346	0.00462	-2.96470
H	1.56124	-0.88237	-2.19160
C	1.62008	-3.24256	1.12132
H	2.30965	-2.43840	1.37888
H	1.37240	-3.79564	2.03361
H	2.15300	-3.93145	0.46010
C	0.74019	0.00050	2.77893

H	-0.29954	-0.00283	3.09282
H	1.27458	-0.87976	3.13348
H	1.26851	0.88452	3.13326
O	-2.47745	-0.00330	-2.48632
C	-1.42457	-0.00300	-3.45533
H	-0.79878	-0.89307	-3.34717
H	-1.88575	-0.00484	-4.44234
H	-0.80132	0.88910	-3.34927
H	-3.59695	-0.00314	-0.34112
O	-2.31124	-0.00027	1.95395
C	-2.99256	1.14942	2.47118
C	-2.99729	-1.14716	2.47120
C	-4.48544	0.78084	2.45551
H	-2.64146	1.34867	3.49207
H	-2.73035	1.99656	1.83835
C	-4.48874	-0.77202	2.45803
H	-2.73974	-1.99499	1.83733
H	-2.64564	-1.34890	3.49139
H	-4.96592	1.16606	1.55538
H	-5.00842	1.20057	3.31580
H	-4.97358	-1.15820	1.56065
H	-5.01107	-1.18651	3.32124

Table S16, **D**, *Z*-form
Level of theory: B3LYP-GD3BJ/6-311++G**
Energy: -1396513.1150349 Kcal/mol

C	-4.52070	0.31577	-0.90303
C	-4.27222	0.49624	0.50828
C	-5.35666	0.60864	1.40702
C	-6.64173	0.53655	0.90987
C	-6.88358	0.35678	-0.47910
C	-5.84752	0.24748	-1.38299
C	-2.38366	0.36266	-0.65088
C	-2.86401	0.51629	0.66430
H	-5.18422	0.74858	2.46972
H	-7.48694	0.61884	1.58443
H	-7.90878	0.30548	-0.82962
H	-6.03322	0.11217	-2.44277
P	-0.74089	0.15952	-1.21761
C	-0.00666	-1.39398	-0.70397
C	-0.64946	-2.58120	-0.43810
C	1.63505	-2.90798	-0.38950
C	0.39037	-3.55829	-0.21970

C	2.84843	-3.58551	-0.24328
C	0.36136	-4.92205	0.10577
C	2.79249	-4.93414	0.08429
H	3.79850	-3.08710	-0.39403
C	1.56306	-5.59713	0.25854
H	-0.58343	-5.43643	0.23688
H	3.71599	-5.48906	0.20027
H	1.56065	-6.65103	0.50981
C	0.39227	1.44859	-0.69667
C	0.12529	2.78107	-0.47771
C	2.40299	2.42785	-0.39959
C	1.40257	3.41904	-0.27038
C	3.76043	2.72724	-0.25342
C	1.76975	4.74280	0.01230
C	4.09795	4.04390	0.03065
H	4.52505	1.96933	-0.37318
C	3.11492	5.04285	0.16374
H	1.01532	5.51475	0.10946
H	5.14245	4.30896	0.14475
H	3.41909	6.05966	0.38141
Si	2.62956	-0.32914	-0.48234
N	1.78585	1.20347	-0.66562
N	1.39854	-1.56396	-0.69020
N	-3.36299	0.23212	-1.61022
C	-1.18618	3.50054	-0.47437
H	-1.20057	4.26946	-1.25327
H	-2.02768	2.82954	-0.63226
H	-1.33606	4.00959	0.48281
C	-2.08662	0.69246	1.92862
H	-1.02442	0.50942	1.77005
H	-2.19340	1.70649	2.33503
H	-2.43201	0.00222	2.70673
C	-2.11626	-2.87533	-0.37993
H	-2.71752	-2.12240	-0.88579
H	-2.32083	-3.84749	-0.83635
H	-2.45739	-2.92307	0.65908
C	-0.75839	0.16616	-3.03412
H	0.25080	-0.02523	-3.40031
H	-1.45207	-0.60346	-3.36894
H	-1.11438	1.13752	-3.37590
O	3.74258	-0.46933	-1.69293
C	3.41386	-0.40958	-3.07940
H	4.33518	-0.53681	-3.64745
H	2.71619	-1.20943	-3.34833

H	2.97022	0.55900	-3.33217
H	3.46124	-0.45376	0.71222
O	1.19640	-0.13140	1.84124
C	1.50588	1.01195	2.66065
C	1.00739	-1.30329	2.66489
C	1.07013	0.62959	4.07177
H	2.58391	1.20877	2.60965
H	0.97598	1.87062	2.24704
C	1.35791	-0.87905	4.09312
H	-0.03677	-1.61431	2.56966
H	1.64233	-2.10441	2.28218
H	0.00121	0.81732	4.19984
H	1.61166	1.18256	4.84072
H	0.77376	-1.41882	4.83965
H	2.41737	-1.05930	4.29558

Chart S4, **Heq1**
Level of theory: B3LYP-GD3BJ/6-31+G*
Energy: -887048.3761154 Kcal/mol

C	-1.57857	-2.35193	-0.82396
C	-0.92705	-3.02280	-1.84753
C	0.36584	-1.31904	-1.14685
C	0.31109	-2.35918	-2.06266
P	1.49426	0.00213	-0.77408
C	1.79642	-0.15692	0.94973
C	2.84990	-0.33446	1.83947
C	0.87970	-0.31503	2.94982
C	2.25653	-0.42824	3.12443
C	0.52739	1.48048	-0.88538
C	0.60445	2.67757	-1.58158
C	-1.28583	2.64655	-0.34268
C	-0.54947	3.42465	-1.22316
Si	-1.21149	-0.01403	0.84683
N	-0.63563	1.46067	-0.11647
N	0.58632	-0.14553	1.62627
N	-0.79791	-1.31658	-0.37878
C	2.95480	0.01070	-1.83173
H	3.59867	0.84728	-1.54817
H	3.49863	-0.92888	-1.70396
H	2.64861	0.11954	-2.87526
O	-2.81847	0.33180	0.37936
C	-3.96354	-0.14549	1.04571
H	-4.39949	-0.99689	0.50269

H	-3.73993	-0.47053	2.07454
H	-4.71335	0.65389	1.08839
H	-1.63570	-0.43482	2.21581
H	-0.81395	4.41268	-1.57560
H	-2.24057	2.83373	0.12489
H	0.08621	-0.34606	3.68458
H	2.77323	-0.56275	4.06569
H	-2.54813	-2.53389	-0.38234
H	-1.29762	-3.88728	-2.38190
H	1.06574	-2.60853	-2.79747
H	3.90327	-0.39173	1.59582
H	1.38943	2.97328	-2.26589

Chart S4, **TS1**
Level of theory: B3LYP-GD3BJ/6-31+G*
Energy: -887047.3726902 Kcal/mol

C	1.66641	0.40675	2.50705
C	1.81309	-0.77326	3.22183
C	0.53335	-1.16076	1.41384
C	1.10102	-1.78111	2.51731
P	-0.42155	-1.64447	-0.00000
C	0.53335	-1.16076	-1.41384
C	1.10102	-1.78111	-2.51731
C	1.66641	0.40675	-2.50705
C	1.81309	-0.77326	-3.22183
C	-1.75982	-0.51048	-0.00000
C	-3.14778	-0.56566	-0.00000
C	-2.45286	1.58583	0.00000
C	-3.58725	0.78178	0.00000
Si	0.53890	1.35497	0.00000
N	-1.32280	0.80985	0.00000
N	0.87722	0.18898	-1.40830
N	0.87722	0.18898	1.40830
C	-0.88581	-3.38684	-0.00000
H	-1.47793	-3.60137	-0.89334
H	0.01602	-4.00449	-0.00000
H	-1.47793	-3.60137	0.89334
O	-0.07889	2.92447	0.00000
C	0.69270	4.11063	0.00000
H	0.44569	4.69874	0.89187
H	1.77090	3.89956	0.00000
H	0.44569	4.69874	-0.89187
H	1.96181	1.84953	0.00000

H	-4.61270	1.12749	0.00000
H	-2.36254	2.65977	0.00000
H	2.07277	1.39038	-2.69974
H	2.36825	-0.89838	-4.14192
H	2.07277	1.39038	2.69974
H	2.36825	-0.89838	4.14192
H	1.01722	-2.82760	2.78124
H	1.01722	-2.82760	-2.78124
H	-3.75863	-1.45954	-0.00000

Chart S4, **Heq2**
Level of theory: B3LYP-GD3BJ/6-31+G*
Energy: -887048.3761531 Kcal/mol

C	-0.87969	-0.31323	2.95007
C	-2.25651	-0.42637	3.12461
C	-1.79619	-0.15679	0.94973
C	-2.84977	-0.33357	1.83949
P	-1.49419	0.00152	-0.77423
C	-0.36566	-1.31974	-1.14633
C	-0.31061	-2.36033	-2.06160
C	1.57893	-2.35212	-0.82274
C	0.92765	-3.02358	-1.84607
C	-0.52764	1.47997	-0.88626
C	-0.60466	2.67666	-1.58319
C	1.28560	2.64628	-0.34423
C	0.54924	3.42393	-1.22514
Si	1.21143	-0.01370	0.84699
N	0.63536	1.46057	-0.11735
N	0.79803	-1.31666	-0.37816
N	-0.58618	-0.14491	1.62636
C	-2.95533	0.00930	-1.83107
H	-2.65003	0.11746	-2.87493
H	-3.49907	-0.93017	-1.70219
H	-3.59895	0.84609	-1.54752
O	2.81827	0.33235	0.37934
C	3.96343	-0.14435	1.04604
H	4.71292	0.65533	1.08864
H	3.73974	-0.46920	2.07490
H	4.39974	-0.99570	0.50326
H	1.63580	-0.43366	2.21619
H	0.81373	4.41177	-1.57811
H	2.24035	2.83369	0.12323
H	2.54851	-2.53361	-0.38097

H	1.29840	-3.88825	-2.38000
H	-0.08625	-0.34364	3.68491
H	-2.77332	-0.56020	4.06591
H	-3.90312	-0.39096	1.59579
H	-1.06517	-2.61019	-2.79633
H	-1.38966	2.97200	-2.26764

Chart S5, **Heq1'**
Level of theory: B3LYP-GD3BJ/6-31+G*
Energy: -1250578.5190560 Kcal/mol

C	0.73202	2.64693	-1.05967
C	0.91951	3.64419	-0.05437
C	1.28622	4.95318	-0.41186
C	1.46625	5.25340	-1.74950
C	1.29068	4.26091	-2.73726
C	0.92991	2.96353	-2.41470
C	0.37654	1.71036	0.91534
C	0.68301	3.02586	1.21541
H	1.42894	5.71232	0.34947
H	1.74980	6.25606	-2.04716
H	1.44554	4.52027	-3.77846
H	0.80705	2.22063	-3.18920
P	-0.01916	0.24107	1.82008
C	1.17293	-0.92467	1.20842
C	2.17722	-1.67835	1.78568
C	2.19824	-1.90677	-0.50898
C	2.85165	-2.30914	0.69008
C	2.66344	-2.35082	-1.75546
C	3.96171	-3.16993	0.63899
C	3.76271	-3.19213	-1.77833
H	2.17356	-2.05291	-2.66913
C	4.40910	-3.60587	-0.59430
H	4.46075	-3.48003	1.55038
H	4.13530	-3.54486	-2.73332
H	5.26473	-4.26776	-0.65801
C	-1.59094	-0.27831	1.16173
C	-2.83849	-0.54821	1.68991
C	-2.86812	-0.75246	-0.60606
C	-3.67221	-0.85350	0.56186
C	-3.41300	-0.96467	-1.87831
C	-5.03185	-1.18943	0.45101
C	-4.75640	-1.29276	-1.95996
H	-2.81203	-0.86623	-2.77039

C	-5.56204	-1.40986	-0.80805
H	-5.65457	-1.27014	1.33494
H	-5.20063	-1.46024	-2.93436
H	-6.60852	-1.66915	-0.91632
Si	-0.10505	-0.23803	-1.29105
N	-1.58302	-0.40306	-0.23561
N	1.15735	-1.05196	-0.18797
N	0.39071	1.45219	-0.45456
C	-3.31318	-0.53946	3.10843
H	-2.51390	-0.35819	3.82471
H	-4.07596	0.23135	3.26043
H	-3.77171	-1.49910	3.36611
C	0.77051	3.72080	2.53705
H	1.77493	4.12477	2.70147
H	0.07494	4.56513	2.58139
H	0.54059	3.06419	3.37566
C	2.56082	-1.84304	3.22256
H	3.50154	-1.32666	3.44335
H	1.80587	-1.46696	3.91120
H	2.71370	-2.90032	3.45706
C	-0.00895	0.43984	3.61153
H	-0.24710	-0.51111	4.08466
H	0.98081	0.77036	3.92271
H	-0.75186	1.18627	3.88828
H	-0.37629	0.59309	-2.49365
O	-0.15839	-1.67895	-2.20157
C	-0.47419	-1.79306	-3.56501
H	-1.35855	-2.42545	-3.69383
H	-0.67394	-0.81527	-4.02823
H	0.35827	-2.26164	-4.10378

Chart S5, **TS1’**
Level of theory: B3LYP-GD3BJ/6-31+G*
Energy: -1250577.4409257 Kcal/mol

C	-2.54812	-1.37566	-0.78225
C	-3.26227	-1.92653	0.31765
C	-4.46590	-2.61591	0.09397
C	-4.92597	-2.74708	-1.20494
C	-4.20572	-2.19742	-2.28666
C	-3.01945	-1.50818	-2.09456
C	-1.42454	-0.89785	1.08135
C	-2.52127	-1.62119	1.50940
H	-5.02358	-3.03777	0.92271

H	-5.85144	-3.27619	-1.39906
H	-4.59179	-2.31626	-3.29245
H	-2.47656	-1.08767	-2.93073
P	0.00028	-0.14271	1.83408
C	-0.00245	1.48934	1.16492
C	-0.00462	2.73806	1.75824
C	-0.00491	2.89752	-0.54540
C	-0.00623	3.65837	0.66691
C	-0.00608	3.57353	-1.78004
C	-0.00868	5.06496	0.63644
C	-0.00849	4.95713	-1.77920
H	-0.00511	3.01796	-2.70077
C	-0.00979	5.70754	-0.58473
H	-0.00968	5.63427	1.55940
H	-0.00939	5.47921	-2.72969
H	-0.01168	6.79035	-0.63089
C	1.42761	-0.89310	1.08135
C	2.52674	-1.61278	1.50941
C	2.55280	-1.36710	-0.78224
C	3.26878	-1.91560	0.31766
C	3.02460	-1.49800	-2.09455
C	4.47472	-2.60094	0.09398
C	4.21317	-2.18326	-2.28665
H	2.48031	-1.07929	-2.93072
C	4.93525	-2.73052	-1.20493
H	5.03379	-3.02094	0.92271
H	4.59965	-2.30078	-3.29244
H	5.86249	-3.25653	-1.39905
Si	0.00020	-0.09593	-1.30055
N	1.42467	-0.72735	-0.30976
N	-0.00255	1.54187	-0.23286
N	-1.42214	-0.73214	-0.30977
C	2.92781	-2.02659	2.88961
H	2.23297	-1.67995	3.65333
H	2.98750	-3.11692	2.96766
H	3.91737	-1.63234	3.14206
C	-2.92097	-2.03632	2.88961
H	-3.91205	-1.64578	3.14184
H	-2.97654	-3.12686	2.96782
H	-2.22757	-1.68692	3.65339
C	-0.00530	3.10114	3.21338
H	-0.88997	2.72694	3.73807
H	0.87987	2.72872	3.73847
H	-0.00640	4.18564	3.32823

C	0.00036	-0.19752	3.63521
H	0.88937	0.31084	4.00456
H	-0.89019	0.30817	4.00455
H	0.00192	-1.23443	3.96648
H	0.00230	-1.33088	-2.15795
O	-0.00152	0.90664	-2.64665
C	-0.00079	0.46722	-3.99191
H	0.88773	0.85584	-4.49822
H	0.00111	-0.62680	-4.06100
H	-0.89067	0.85276	-4.49819

Chart S5, **Heq2’**
Level of theory: B3LYP-GD3BJ/6-31+G*
Energy: -1250578.5190434 Kcal/mol

C	2.86719	-0.75513	-0.60569
C	3.67101	-0.85693	0.56234
C	5.03036	-1.19412	0.45168
C	5.56052	-1.41504	-0.80730
C	4.75516	-1.29718	-1.95933
C	3.41206	-0.96783	-1.87787
C	1.59022	-0.27956	1.16189
C	2.83742	-0.55074	1.69026
H	5.65287	-1.27543	1.33570
H	6.60677	-1.67530	-0.91542
H	5.19939	-1.46508	-2.93365
H	2.81131	-0.86884	-2.77003
P	0.01887	0.24144	1.82006
C	-1.17439	-0.92315	1.20834
C	-2.17943	-1.67592	1.78550
C	-2.20017	-1.90483	-0.50911
C	-2.85414	-2.30642	0.68988
C	-2.66536	-2.34874	-1.75562
C	-3.96490	-3.16631	0.63867
C	-3.76529	-3.18919	-1.77862
H	-2.17501	-2.05140	-2.66923
C	-4.41231	-3.60214	-0.59466
H	-4.46443	-3.47584	1.54997
H	-4.13791	-3.54184	-2.73362
H	-5.26846	-4.26335	-0.65845
C	-0.37505	1.71109	0.91520
C	-0.68002	3.02701	1.21502
C	-0.72897	2.64774	-1.06002
C	-0.91540	3.64543	-0.05492

C	-0.92626	2.96443	-2.41513
C	-1.28040	4.95484	-0.41266
C	-1.28535	4.26223	-2.73795
H	-0.80427	2.22131	-3.18954
C	-1.45983	5.25509	-1.75037
H	-1.42228	5.71426	0.34853
H	-1.43973	4.52160	-3.77921
H	-1.74207	6.25805	-2.04824
Si	0.10456	-0.23866	-1.29094
N	-0.38928	1.45266	-0.45464
N	-1.15869	-1.05077	-0.18804
N	1.58239	-0.40434	-0.23546
C	-0.76688	3.72218	2.53657
H	-0.53928	3.06501	3.37536
H	-0.06931	4.56482	2.58153
H	-1.77043	4.12859	2.70018
C	3.31188	-0.54269	3.10886
H	3.76808	-1.50332	3.36695
H	4.07648	0.22634	3.26072
H	2.51295	-0.35924	3.82496
C	-2.56343	-1.84023	3.22232
H	-1.80917	-1.46306	3.91107
H	-3.50471	-1.32461	3.44242
H	-2.71545	-2.89753	3.45732
C	0.00866	0.44028	3.61148
H	-0.98077	0.77194	3.92248
H	0.24568	-0.51090	4.08469
H	0.75233	1.18592	3.88826
H	0.37429	0.59175	-2.49426
O	0.15837	-1.68029	-2.20047
C	0.47291	-1.79512	-3.56411
H	-0.36002	-2.26392	-4.10195
H	0.67232	-0.81759	-4.02805
H	1.35712	-2.42761	-3.69342

Figure S22, ***Z*-form**
Level of theory: B3LYP-GD3BJ/6-31+G*
Energy: -887042.2718100 Kcal/mol

C	-2.00754	2.63762	-0.35192
C	-1.07939	3.49920	-0.89493
C	-0.19857	1.41472	-0.87775
C	0.06946	2.72752	-1.22045
P	0.91631	0.01676	-0.95764

C	-0.15989	-1.41952	-0.87121
C	0.14904	-2.72749	-1.19990
C	-1.90958	-2.70240	-0.28387
C	-0.96232	-3.53678	-0.83581
C	2.15691	0.08181	0.24887
C	3.55661	-0.11683	0.12787
C	2.91280	0.18461	2.26651
C	4.04116	-0.04163	1.44021
Si	-2.32972	-0.02932	0.36347
N	1.76899	0.25738	1.55951
N	-1.43622	-1.39932	-0.29820
N	-1.48712	1.35330	-0.33636
C	1.67682	-0.01078	-2.61203
H	2.32748	-0.88512	-2.69816
H	0.89421	-0.05824	-3.37206
H	2.27725	0.89195	-2.74939
O	-2.41231	-0.01919	1.98528
C	-1.31142	-0.08137	2.92355
H	-1.53100	0.63177	3.72179
H	-1.27578	-1.09357	3.33704
H	-0.35031	0.15829	2.45490
H	-3.71846	-0.04975	-0.10889
H	5.07397	-0.12744	1.75743
H	2.89998	0.30386	3.34554
H	-2.89092	-2.92501	0.11336
H	-1.05413	-4.60659	-0.96525
H	-3.00344	2.83006	0.02411
H	-1.20765	4.56296	-1.04179
H	0.99794	3.09231	-1.63940
H	1.08009	-3.06549	-1.63540
H	4.12668	-0.27898	-0.77950

Figure S22, **TS2**
Level of theory: B3LYP-GD3BJ/6-31+G*
Energy: -887033.5995852 Kcal/mol

C	2.02811	2.36412	0.56514
C	1.25337	3.47257	0.83495
C	-0.09825	1.66740	0.69494
C	-0.09844	3.03191	0.91585
P	-1.42528	0.48809	0.49361
C	-0.78531	-0.98167	1.29420
C	-1.40274	-1.98446	2.01773
C	0.70911	-2.63617	1.56147
C	-0.45121	-3.03047	2.19193

C	-1.65739	0.19222	-1.19647
C	-2.74040	-0.01034	-2.07938
C	-0.75156	-0.54524	-3.01616
C	-2.14342	-0.47197	-3.26529
Si	1.79080	-0.41427	0.24649
N	-0.44791	-0.14792	-1.76523
N	0.52250	-1.38365	1.00177
N	1.21436	1.24892	0.47126
C	-2.89918	1.05308	1.38218
H	-3.69063	0.30682	1.27108
H	-2.67011	1.19514	2.44075
H	-3.24067	1.99483	0.94457
O	2.30509	-0.97418	-1.20093
C	3.63266	-0.83241	-1.69679
H	3.70214	0.07147	-2.31238
H	4.37207	-0.77740	-0.88534
H	3.85012	-1.70541	-2.31791
H	2.98469	-0.48115	1.12116
H	-2.64976	-0.71158	-4.19324
H	0.03099	-0.86650	-3.69557
H	1.66192	-3.13998	1.47310
H	-0.60332	-3.96165	2.72069
H	3.09972	2.27434	0.44861
H	1.61298	4.48388	0.96818
H	-0.96899	3.64505	1.10847
H	-2.42578	-1.97524	2.37028
H	-3.79272	0.16650	-1.89064

Section S5.3.8, opening into an E-form: **TS**
Level of theory: B3LYP-GD3BJ/6-31+G*
Energy: -887036.4371328 Kcal/mol

C	2.45571	1.09378	-2.57926
C	3.23935	-0.08973	-2.56400
C	2.09687	0.17137	-0.66261
C	3.00195	-0.69831	-1.32705
P	1.29207	-0.04768	0.86045
C	0.23785	-1.50091	0.86090
C	0.38366	-2.78243	1.35570
C	-1.51688	-2.70437	0.13640
C	-0.73230	-3.54319	0.89862
C	0.14491	1.29391	1.10825
C	0.22010	2.46845	1.82898
C	-1.74028	2.44273	0.70679
C	-0.97898	3.19452	1.57669

Si	-1.55328	-0.00006	-0.70973
N	-1.06738	1.27103	0.41348
N	-0.93505	-1.44885	0.09900
N	1.75889	1.26367	-1.44314
C	2.37463	-0.20056	2.31595
H	1.78629	-0.37787	3.22029
H	3.06925	-1.02980	2.15191
H	2.95629	0.72013	2.41268
O	-3.18949	-0.08542	-0.74529
C	-4.02247	0.07012	-1.89573
H	-4.65454	0.95267	-1.75774
H	-3.43212	0.18878	-2.81236
H	-4.65709	-0.81611	-1.98780
H	-1.02154	0.16989	-2.06675
H	-1.25251	4.15396	1.99431
H	-2.72143	2.62757	0.29299
H	-2.46189	-2.88335	-0.35652
H	-0.93817	-4.58270	1.11562
H	2.38045	1.82302	-3.38004
H	3.89152	-0.45004	-3.35171
H	3.41236	-1.63110	-0.95674
H	1.19852	-3.13566	1.97383
H	1.04532	2.77687	2.45672

Section S5.3.8, opening into an *E*-form: ***E*-form**
Level of theory: B3LYP-GD3BJ/6-31+G*
Energy: -887040.9585205 Kcal/mol

C	4.59034	-0.06438	0.69954
C	4.54463	-0.49717	-0.65202
C	2.49983	-0.24709	0.20483
C	3.19065	-0.60913	-0.98192
P	0.80968	-0.03903	0.48039
C	-0.16542	-1.34057	-0.27743
C	0.20122	-2.63547	-0.58911
C	-1.95860	-2.35295	-1.18710
C	-0.93453	-3.27503	-1.16410
C	0.09249	1.46228	-0.21275
C	0.71713	2.65903	-0.49473
C	-1.47610	2.88128	-0.99335
C	-0.27661	3.55655	-0.98557
Si	-2.44245	0.30604	-0.36927
N	-1.27237	1.59063	-0.52233
N	-1.50624	-1.15704	-0.64977
N	3.36638	0.09690	1.22857

C	0.47947	0.00272	2.27088
H	-0.56989	0.25594	2.44562
H	0.71781	-0.97425	2.69905
H	1.13864	0.75418	2.71227
O	-3.02610	0.34192	1.16691
C	-3.79063	-0.70995	1.76429
H	-3.97722	-0.42763	2.80250
H	-4.75170	-0.83612	1.25042
H	-3.23600	-1.65528	1.73916
H	-3.50598	0.40285	-1.38247
H	-0.12199	4.57708	-1.30874
H	-2.46006	3.20317	-1.30710
H	-2.97009	-2.43954	-1.56158
H	-0.98598	-4.28988	-1.53465
H	5.47449	0.12480	1.30025
H	5.39050	-0.71034	-1.29574
H	2.75481	-0.90919	-1.92748
H	1.18775	-3.05163	-0.43683
H	1.77515	2.84736	-0.37014

Scheme S9, **TS2**
Level of theory: B3LYP-GD3BJ/6-31+G*
Energy: -887033.5995852 Kcal/mol

C	2.02811	2.36412	0.56514
C	1.25337	3.47257	0.83495
C	-0.09825	1.66740	0.69494
C	-0.09844	3.03191	0.91585
P	-1.42528	0.48809	0.49361
C	-0.78531	-0.98167	1.29420
C	-1.40274	-1.98446	2.01773
C	0.70911	-2.63617	1.56147
C	-0.45121	-3.03047	2.19193
C	-1.65739	0.19222	-1.19647
C	-2.74040	-0.01034	-2.07938
C	-0.75156	-0.54524	-3.01616
C	-2.14342	-0.47197	-3.26529
Si	1.79080	-0.41427	0.24649
N	-0.44791	-0.14792	-1.76523
N	0.52250	-1.38365	1.00177
N	1.21436	1.24892	0.47126
C	-2.89918	1.05308	1.38218
H	-3.69063	0.30682	1.27108
H	-2.67011	1.19514	2.44075
H	-3.24067	1.99483	0.94457

O	2.30509	-0.97418	-1.20093
C	3.63266	-0.83241	-1.69679
H	3.70214	0.07147	-2.31238
H	4.37207	-0.77740	-0.88534
H	3.85012	-1.70541	-2.31791
H	2.98469	-0.48115	1.12116
H	-2.64976	-0.71158	-4.19324
H	0.03099	-0.86650	-3.69557
H	1.66192	-3.13998	1.47310
H	-0.60332	-3.96165	2.72069
H	3.09972	2.27434	0.44861
H	1.61298	4.48388	0.96818
H	-0.96899	3.64505	1.10847
H	-2.42578	-1.97524	2.37028
H	-3.79272	0.16650	-1.89064
